# Global, regional, and national burden of diabetes from 1990 to 2021, with projections of prevalence to 2050: a systematic analysis for the Global Burden of Disease Study 2021

**DOI:** 10.1016/S0140-6736(23)01301-6

**Published:** 2023-07-15

**Authors:** GBD 2021 Diabetes Collaborators, Kanyin Liane Ong, Lauryn K Stafford, Susan A McLaughlin, Edward J Boyko, Stein Emil Vollset, Amanda E Smith, Bronte E Dalton, Joe Duprey, Jessica A Cruz, Hailey Hagins, Paulina A Lindstedt, Amirali Aali, Yohannes Habtegiorgis Abate, Melsew Dagne Abate, Mohammadreza Abbasian, Zeinab Abbasi-Kangevari, Mohsen Abbasi-Kangevari, Samar Abd ElHafeez, Rami Abd-Rabu, Deldar Morad Abdulah, Abu Yousuf Md Abdullah, Vida Abedi, Hassan Abidi, Richard Gyan Aboagye, Hassan Abolhassani, Eman Abu-Gharbieh, Ahmed Abu-Zaid, Tigist Demssew Adane, Denberu Eshetie Adane, Isaac Yeboah Addo, Oyelola A Adegboye, Victor Adekanmbi, Abiola Victor Adepoju, Qorinah Estiningtyas Sakilah Adnani, Rotimi Felix Afolabi, Gina Agarwal, Zahra Babaei Aghdam, Marcela Agudelo-Botero, Constanza Elizabeth Aguilera Arriagada, Williams Agyemang-Duah, Bright Opoku Ahinkorah, Danish Ahmad, Rizwan Ahmad, Sajjad Ahmad, Aqeel Ahmad, Ali Ahmadi, Keivan Ahmadi, Ayman Ahmed, Ali Ahmed, Luai A Ahmed, Syed Anees Ahmed, Marjan Ajami, Rufus Olusola Akinyemi, Hanadi Al Hamad, Syed Mahfuz Al Hasan, Tareq Mohammed Ali AL-Ahdal, Tariq A Alalwan, Ziyad Al-Aly, Mohammad T AlBataineh, Jacqueline Elizabeth Alcalde-Rabanal, Sharifullah Alemi, Hassam Ali, Tahereh Alinia, Syed Mohamed Aljunid, Sami Almustanyir, Rajaa M Al-Raddadi, Nelson Alvis-Guzman, Firehiwot Amare, Edward Kwabena Ameyaw, Sohrab Amiri, Ganiyu Adeniyi Amusa, Catalina Liliana Andrei, Ranjit Mohan Anjana, Adnan Ansar, Golnoosh Ansari, Alireza Ansari-Moghaddam, Anayochukwu Edward Anyasodor, Jalal Arabloo, Aleksandr Y Aravkin, Demelash Areda, Hidayat Arifin, Mesay Arkew, Benedetta Armocida, Johan Ärnlöv, Anton A Artamonov, Judie Arulappan, Raphael Taiwo Aruleba, Ashokan Arumugam, Zahra Aryan, Mulu Tiruneh Asemu, Mohammad Asghari-Jafarabadi, Elaheh Askari, Daniel Asmelash, Thomas Astell-Burt, Mohammad Athar, Seyyed Shamsadin Athari, Maha Moh'd Wahbi Atout, Leticia Avila-Burgos, Ahmed Awaisu, Sina Azadnajafabad, Darshan B B, Hassan Babamohamadi, Muhammad Badar, Alaa Badawi, Ashish D Badiye, Nayereh Baghcheghi, Nasser Bagheri, Sara Bagherieh, Sulaiman Bah, Saeed Bahadory, Ruhai Bai, Atif Amin Baig, Ovidiu Constantin Baltatu, Hamid Reza Baradaran, Martina Barchitta, Mainak Bardhan, Noel C Barengo, Till Winfried Bärnighausen, Mark Thomaz Ugliara Barone, Francesco Barone-Adesi, Amadou Barrow, Hamideh Bashiri, Afisu Basiru, Sanjay Basu, Saurav Basu, Abdul-Monim Mohammad Batiha, Kavita Batra, Mulat Tirfie Bayih, Nebiyou Simegnew Bayileyegn, Amir Hossein Behnoush, Alehegn Bekele Bekele, Melaku Ashagrie Belete, Uzma Iqbal Belgaumi, Luis Belo, Derrick A Bennett, Isabela M Bensenor, Kidanemaryam Berhe, Alemshet Yirga Berhie, Sonu Bhaskar, Ajay Nagesh Bhat, Jasvinder Singh Bhatti, Boris Bikbov, Faiq Bilal, Bagas Suryo Bintoro, Saeid Bitaraf, Veera R Bitra, Vesna Bjegovic-Mikanovic, Virginia Bodolica, Archith Boloor, Michael Brauer, Javier Brazo-Sayavera, Hermann Brenner, Zahid A Butt, Daniela Calina, Luciana Aparecida Campos, Ismael R Campos-Nonato, Yin Cao, Chao Cao, Josip Car, Márcia Carvalho, Carlos A Castañeda-Orjuela, Ferrán Catalá-López, Ester Cerin, Joshua Chadwick, Eeshwar K Chandrasekar, Gashaw Sisay Chanie, Jaykaran Charan, Vijay Kumar Chattu, Kirti Chauhan, Huzaifa Ahmad Cheema, Endeshaw Chekol Abebe, Simiao Chen, Nicolas Cherbuin, Fatemeh Chichagi, Saravana Babu Chidambaram, William C S Cho, Sonali Gajanan Choudhari, Rajiv Chowdhury, Enayet Karim Chowdhury, Dinh-Toi Chu, Isaac Sunday Chukwu, Sheng-Chia Chung, Kaleb Coberly, Alyssa Columbus, Daniela Contreras, Ewerton Cousin, Michael H Criqui, Natália Cruz-Martins, Sarah Cuschieri, Bashir Dabo, Omid Dadras, Xiaochen Dai, Albertino Antonio Moura Damasceno, Rakhi Dandona, Lalit Dandona, Saswati Das, Ana Maria Dascalu, Nihar Ranjan Dash, Mohsen Dashti, Claudio Alberto Dávila-Cervantes, Vanessa De la Cruz-Góngora, Gebiso Roba Debele, Kourosh Delpasand, Fitsum Wolde Demisse, Getu Debalkie Demissie, Xinlei Deng, Edgar Denova-Gutiérrez, Salil V Deo, Emina Dervišević, Hardik Dineshbhai Desai, Aragaw Tesfaw Desale, Anteneh Mengist Dessie, Fikreab Desta, Syed Masudur Rahman Dewan, Sourav Dey, Kuldeep Dhama, Meghnath Dhimal, Nancy Diao, Daniel Diaz, Monica Dinu, Mengistie Diress, Shirin Djalalinia, Linh Phuong Doan, Deepa Dongarwar, Francisco Winter dos Santos Figueiredo, Bruce B Duncan, Siddhartha Dutta, Arkadiusz Marian Dziedzic, Hisham Atan Edinur, Michael Ekholuenetale, Temitope Cyrus Ekundayo, Islam Y Elgendy, Muhammed Elhadi, Waseem El-Huneidi, Omar Abdelsadek Abdou Elmeligy, Mohamed A Elmonem, Destaw Endeshaw, Hawi Leul Esayas, Habitu Birhan Eshetu, Farshid Etaee, Ibtihal Fadhil, Adeniyi Francis Fagbamigbe, Ayesha Fahim, Shahab Falahi, MoezAlIslam Ezzat Mahmoud Faris, Hossein Farrokhpour, Farshad Farzadfar, Ali Fatehizadeh, Ghazal Fazli, Xiaoqi Feng, Tomas Y Ferede, Florian Fischer, David Flood, Ali Forouhari, Roham Foroumadi, Masoumeh Foroutan Koudehi, Abhay Motiramji Gaidhane, Santosh Gaihre, Abduzhappar Gaipov, Yaseen Galali, Balasankar Ganesan, MA Garcia-Gordillo, Rupesh K Gautam, Mesfin Gebrehiwot, Kahsu Gebrekirstos Gebrekidan, Teferi Gebru Gebremeskel, Lemma Getacher, Fataneh Ghadirian, Seyyed-Hadi Ghamari, Mohammad Ghasemi Nour, Fariba Ghassemi, Mahaveer Golechha, Pouya Goleij, Davide Golinelli, Sameer Vali Gopalani, Habtamu Alganeh Guadie, Shi-Yang Guan, Temesgen Worku Gudayu, Rafael Alves Guimarães, Rashid Abdi Guled, Rajeev Gupta, Kartik Gupta, Veer Bala Gupta, Vivek Kumar Gupta, Bishal Gyawali, Rasool Haddadi, Najah R Hadi, Teklehaimanot Gereziher Haile, Ramtin Hajibeygi, Arvin Haj-Mirzaian, Rabih Halwani, Samer Hamidi, Graeme J Hankey, Md Abdul Hannan, Shafiul Haque, Hamid Harandi, Netanja I Harlianto, S M Mahmudul Hasan, Syed Shahzad Hasan, Hamidreza Hasani, Soheil Hassanipour, Mohammed Bheser Hassen, Johannes Haubold, Khezar Hayat, Golnaz Heidari, Mohammad Heidari, Kamran Hessami, Yuta Hiraike, Ramesh Holla, Sahadat Hossain, Md Shakhaoat Hossain, Mohammad-Salar Hosseini, Mehdi Hosseinzadeh, Hassan Hosseinzadeh, Junjie Huang, Md Nazmul Huda, Salman Hussain, Hong-Han Huynh, Bing-Fang Hwang, Segun Emmanuel Ibitoye, Nayu Ikeda, Irena M Ilic, Milena D Ilic, Leeberk Raja Inbaraj, Afrin Iqbal, Sheikh Mohammed Shariful Islam, Rakibul M Islam, Nahlah Elkudssiah Ismail, Hiroyasu Iso, Gaetano Isola, Ramaiah Itumalla, Masao Iwagami, Chidozie C D Iwu, Ihoghosa Osamuyi Iyamu, Assefa N Iyasu, Louis Jacob, Abdollah Jafarzadeh, Haitham Jahrami, Rajesh Jain, Chinwe Jaja, Zahra Jamalpoor, Elham Jamshidi, Balamurugan Janakiraman, Krishnamurthy Jayanna, Sathish Kumar Jayapal, Shubha Jayaram, Ranil Jayawardena, Rime Jebai, Wonjeong Jeong, Yinzi Jin, Mohammad Jokar, Jost B Jonas, Nitin Joseph, Abel Joseph, Charity Ehimwenma Joshua, Farahnaz Joukar, Jacek Jerzy Jozwiak, Billingsley Kaambwa, Ali Kabir, Robel Hussen Kabthymer, Vidya Kadashetti, Farima Kahe, Rohollah Kalhor, Himal Kandel, Shama D Karanth, Ibraheem M Karaye, Samad Karkhah, Patrick DMC Katoto, Navjot Kaur, Sina Kazemian, Sewnet Adem Kebede, Yousef Saleh Khader, Himanshu Khajuria, Amirmohammad Khalaji, Moien AB Khan, Maseer Khan, Ajmal Khan, Saval Khanal, Moawiah Mohammad Khatatbeh, Amir M Khater, Sorour Khateri, Fatemeh khorashadizadeh, Jagdish Khubchandani, Biruk Getahun Kibret, Min Seo Kim, Ruth W Kimokoti, Adnan Kisa, Mika Kivimäki, Ali-Asghar Kolahi, Somayeh Komaki, Farzad Kompani, Hamid Reza Koohestani, Oleksii Korzh, Karel Kostev, Nikhil Kothari, Ai Koyanagi, Kewal Krishan, Yuvaraj Krishnamoorthy, Barthelemy Kuate Defo, Mohammed Kuddus, Md Abdul Kuddus, Rakesh Kumar, Harish Kumar, Satyajit Kundu, Maria Dyah Kurniasari, Ambily Kuttikkattu, Carlo La Vecchia, Tea Lallukka, Bagher Larijani, Anders O Larsson, Kamaluddin Latief, Basira Kankia Lawal, Thao Thi Thu Le, Trang Thi Bich Le, Shaun Wen Huey Lee, Munjae Lee, Wei-Chen Lee, Paul H Lee, Sang-woong Lee, Seung Won Lee, Samson Mideksa Legesse, Jacopo Lenzi, Yongze Li, Ming-Chieh Li, Stephen S Lim, Lee-Ling Lim, Xuefeng Liu, Chaojie Liu, Chun-Han Lo, Graciliana Lopes, Stefan Lorkowski, Rafael Lozano, Giancarlo Lucchetti, Azzam A Maghazachi, Phetole Walter Mahasha, Soleiman Mahjoub, Mansour Adam Mahmoud, Razzagh Mahmoudi, Marzieh Mahmoudimanesh, Anh Tuan Mai, Azeem Majeed, Pantea Majma Sanaye, Konstantinos Christos Makris, Kashish Malhotra, Ahmad Azam Malik, Iram Malik, Tauqeer Hussain Mallhi, Deborah Carvalho Malta, Abdullah A Mamun, Borhan Mansouri, Hamid Reza Marateb, Parham Mardi, Santi Martini, Miquel Martorell, Roy Rillera Marzo, Reza Masoudi, Sahar Masoudi, Elezebeth Mathews, Andrea Maugeri, Giampiero Mazzaglia, Teferi Mekonnen, Mahboobeh Meshkat, Tomislav Mestrovic, Junmei Miao Jonasson, Tomasz Miazgowski, Irmina Maria Michalek, Le Huu Nhat Minh, GK Mini, J Jaime Miranda, Reza Mirfakhraie, Erkin M Mirrakhimov, Mohammad Mirza-Aghazadeh-Attari, Awoke Misganaw, Kebede Haile Misgina, Manish Mishra, Babak Moazen, Nouh Saad Mohamed, Esmaeil Mohammadi, Mohsen Mohammadi, Abdollah Mohammadian-Hafshejani, Marita Mohammadshahi, Alireza Mohseni, Hoda Mojiri-forushani, Ali H Mokdad, Sara Momtazmanesh, Lorenzo Monasta, Md Moniruzzaman, Ute Mons, Fateme Montazeri, AmirAli Moodi Ghalibaf, Yousef Moradi, Maryam Moradi, Mostafa Moradi Sarabi, Negar Morovatdar, Shane Douglas Morrison, Jakub Morze, Elias Mossialos, Ebrahim Mostafavi, Ulrich Otto Mueller, Francesk Mulita, Admir Mulita, Efrén Murillo-Zamora, Kamarul Imran Musa, Julius C Mwita, Shankar Prasad Nagaraju, Mohsen Naghavi, Firzan Nainu, Tapas Sadasivan Nair, Hastyar Hama Rashid Najmuldeen, Vinay Nangia, Shumaila Nargus, Abdallah Y Naser, Hasan Nassereldine, Zuhair S Natto, Javaid Nauman, Biswa Prakash Nayak, Rawlance Ndejjo, Hadush Negash, Ruxandra Irina Negoi, Hau Thi Hien Nguyen, Dang H Nguyen, Phat Tuan Nguyen, Van Thanh Nguyen, Hien Quang Nguyen, Robina Khan Niazi, Yeshambel T Nigatu, Dina Nur Anggraini Ningrum, Muhammad A Nizam, Lawrence Achilles Nnyanzi, Mamoona Noreen, Jean Jacques Noubiap, Ogochukwu Janet Nzoputam, Chimezie Igwegbe Nzoputam, Bogdan Oancea, Nkechi Martina Odogwu, Oluwakemi Ololade Odukoya, Vivek Anand Ojha, Hassan Okati-Aliabad, Akinkunmi Paul Okekunle, Osaretin Christabel Okonji, Patrick Godwin Okwute, Isaac Iyinoluwa Olufadewa, Obinna E Onwujekwe, Michal Ordak, Alberto Ortiz, Uchechukwu Levi Osuagwu, Abderrahim Oulhaj, Mayowa O Owolabi, Alicia Padron-Monedero, Jagadish Rao Padubidri, Raffaele Palladino, Demosthenes Panagiotakos, Songhomitra Panda-Jonas, Ashok Pandey, Anamika Pandey, Seithikurippu R Pandi-Perumal, Anca Mihaela Pantea Stoian, Shahina Pardhan, Tarang Parekh, Utsav Parekh, Maja Pasovic, Jay Patel, Jenil R Patel, Uttam Paudel, Veincent Christian Filipino Pepito, Marcos Pereira, Norberto Perico, Simone Perna, Ionela-Roxana Petcu, Fanny Emily Petermann-Rocha, Vivek Podder, Maarten J Postma, Ghazaleh Pourali, Naeimeh Pourtaheri, Elton Junio Sady Prates, Mirza Muhammad Fahd Qadir, Ibrahim Qattea, Pourya Raee, Ibrar Rafique, Mehran Rahimi, Mahban Rahimifard, Vafa Rahimi-Movaghar, Md Obaidur Rahman, Muhammad Aziz Rahman, Mohammad Hifz Ur Rahman, Mosiur Rahman, Md Mosfequr Rahman, Mohamed Rahmani, Shayan Rahmani, Vahid Rahmanian, Setyaningrum Rahmawaty, Niloufar Rahnavard, Bibek Rajbhandari, Pradhum Ram, Sheena Ramazanu, Juwel Rana, Nemanja Rancic, Muhammad Modassar Ali Nawaz Ranjha, Chythra R Rao, Deepthi Rapaka, Drona Prakash Rasali, Sina Rashedi, Vahid Rashedi, Ahmed Mustafa Rashid, Mohammad-Mahdi Rashidi, Zubair Ahmed Ratan, Salman Rawaf, Lal Rawal, Elrashdy Moustafa Mohamed Redwan, Giuseppe Remuzzi, Kannan RR Rengasamy, Andre M N Renzaho, Luis Felipe Reyes, Nima Rezaei, Nazila Rezaei, Mohsen Rezaeian, Hossein Rezazadeh, Seyed Mohammad Riahi, Yohanes Andy Rias, Muhammad Riaz, Daniela Ribeiro, Mónica Rodrigues, Jefferson Antonio Buendia Rodriguez, Leonardo Roever, Peter Rohloff, Gholamreza Roshandel, Abazar Roustazadeh, Godfrey M Rwegerera, Aly M A Saad, Maha Mohamed Saber-Ayad, Siamak Sabour, Leila Sabzmakan, Basema Saddik, Erfan Sadeghi, Umar Saeed, Sahar Saeedi Moghaddam, Sare Safi, Sher Zaman Safi, Amene Saghazadeh, Narjes Saheb Sharif-Askari, Fatemeh Saheb Sharif-Askari, Amirhossein Sahebkar, Soumya Swaroop Sahoo, Harihar Sahoo, KM Saif-Ur-Rahman, Mirza Rizwan Sajid, Sarvenaz Salahi, Saina Salahi, Mohamed A Saleh, Mohammad Amin Salehi, Joshua A Salomon, Juan Sanabria, Rama Krishna Sanjeev, Francesco Sanmarchi, Milena M Santric-Milicevic, Made Ary Sarasmita, Saman Sargazi, Brijesh Sathian, Thirunavukkarasu Sathish, Monika Sawhney, Markus P Schlaich, Maria Inês Schmidt, Art Schuermans, Abdul-Aziz Seidu, Nachimuthu Senthil Kumar, Sadaf G Sepanlou, Yashendra Sethi, Allen Seylani, Maryam Shabany, Tahereh Shafaghat, Melika Shafeghat, Mahan Shafie, Nilay S Shah, Samiah Shahid, Masood Ali Shaikh, Mohd Shanawaz, Mohammed Shannawaz, Sadaf Sharfaei, Bereket Beyene Shashamo, Rahman Shiri, Aminu Shittu, K M Shivakumar, Siddharudha Shivalli, Parnian Shobeiri, Fereshteh Shokri, Kerem Shuval, Migbar Mekonnen Sibhat, Luís Manuel Lopes Rodrigues Silva, Colin R Simpson, Jasvinder A Singh, Paramdeep Singh, Surjit Singh, Md Shahjahan Siraj, Anna Aleksandrovna Skryabina, Abdullah Al Mamun Sohag, Hamidreza Soleimani, Solikhah Solikhah, Mohammad Sadegh Soltani-Zangbar, Ranjani Somayaji, Reed J D Sorensen, Antonina V Starodubova, Sujata Sujata, Muhammad Suleman, Jing Sun, Johan Sundström, Rafael Tabarés-Seisdedos, Seyyed Mohammad Tabatabaei, Seyed-Amir Tabatabaeizadeh, Mohammad Tabish, Majid Taheri, Ensiyeh Taheri, Elahe Taki, Jacques JL Lukenze Tamuzi, Ker-Kan Tan, Nathan Y Tat, Birhan Tsegaw Taye, Worku Animaw Temesgen, Mohamad-Hani Temsah, Riki Tesler, Pugazhenthan Thangaraju, Kavumpurathu Raman Thankappan, Rajshree Thapa, Samar Tharwat, Nihal Thomas, Jansje Henny Vera Ticoalu, Amir Tiyuri, Marcello Tonelli, Marcos Roberto Tovani-Palone, Domenico Trico, Indang Trihandini, Jaya Prasad Tripathy, Samuel Joseph Tromans, Guesh Mebrahtom Tsegay, Abdul Rohim Tualeka, Derara Girma Tufa, Stefanos Tyrovolas, Sana Ullah, Era Upadhyay, Seyed Mohammad Vahabi, Asokan Govindaraj Vaithinathan, Rohollah Valizadeh, Kim Robin van Daalen, Priya Vart, Shoban Babu Varthya, Tommi Juhani Vasankari, Siavash Vaziri, Madhur verma Verma, Georgios-Ioannis Verras, Danh Cao Vo, Birhanu Wagaye, Yasir Waheed, Ziyue Wang, Yanqing Wang, Cong Wang, Fang Wang, Gizachew Tadesse Wassie, Melissa Y Wei Wei, Abrha Hailay Weldemariam, Ronny Westerman, Nuwan Darshana Wickramasinghe, YiFan Wu, Ratna DWI Wulandari, Juan Xia, Hong Xiao, Suowen Xu, Xiaoyue Xu, Dereje Y Yada, Lin Yang, Hiroshi Yatsuya, Metin Yesiltepe, Siyan Yi, Hunachew Kibret Yohannis, Naohiro Yonemoto, Yuyi You, Sojib Bin Zaman, Nelson Zamora, Iman Zare, Kourosh Zarea, Armin Zarrintan, Mikhail Sergeevich Zastrozhin, Naod Gebrekrstos Zeru, Zhi-Jiang Zhang, Chenwen Zhong, Jingjing Zhou, Magdalena Zielińska, Yossef Teshome Zikarg, Sanjay Zodpey, Mohammad Zoladl, Zhiyong Zou, Alimuddin Zumla, Yves Miel H Zuniga, Dianna J Magliano, Christopher J L Murray, Simon I Hay, Theo Vos

## Abstract

**Background:**

Diabetes is one of the leading causes of death and disability worldwide, and affects people regardless of country, age group, or sex. Using the most recent evidentiary and analytical framework from the Global Burden of Diseases, Injuries, and Risk Factors Study (GBD), we produced location-specific, age-specific, and sex-specific estimates of diabetes prevalence and burden from 1990 to 2021, the proportion of type 1 and type 2 diabetes in 2021, the proportion of the type 2 diabetes burden attributable to selected risk factors, and projections of diabetes prevalence through 2050.

**Methods:**

Estimates of diabetes prevalence and burden were computed in 204 countries and territories, across 25 age groups, for males and females separately and combined; these estimates comprised lost years of healthy life, measured in disability-adjusted life-years (DALYs; defined as the sum of years of life lost [YLLs] and years lived with disability [YLDs]). We used the Cause of Death Ensemble model (CODEm) approach to estimate deaths due to diabetes, incorporating 25 666 location-years of data from vital registration and verbal autopsy reports in separate total (including both type 1 and type 2 diabetes) and type-specific models. Other forms of diabetes, including gestational and monogenic diabetes, were not explicitly modelled. Total and type 1 diabetes prevalence was estimated by use of a Bayesian meta-regression modelling tool, DisMod-MR 2.1, to analyse 1527 location-years of data from the scientific literature, survey microdata, and insurance claims; type 2 diabetes estimates were computed by subtracting type 1 diabetes from total estimates. Mortality and prevalence estimates, along with standard life expectancy and disability weights, were used to calculate YLLs, YLDs, and DALYs. When appropriate, we extrapolated estimates to a hypothetical population with a standardised age structure to allow comparison in populations with different age structures. We used the comparative risk assessment framework to estimate the risk-attributable type 2 diabetes burden for 16 risk factors falling under risk categories including environmental and occupational factors, tobacco use, high alcohol use, high body-mass index (BMI), dietary factors, and low physical activity. Using a regression framework, we forecast type 1 and type 2 diabetes prevalence through 2050 with Socio-demographic Index (SDI) and high BMI as predictors, respectively.

**Findings:**

In 2021, there were 529 million (95% uncertainty interval [UI] 500–564) people living with diabetes worldwide, and the global age-standardised total diabetes prevalence was 6·1% (5·8–6·5). At the super-region level, the highest age-standardised rates were observed in north Africa and the Middle East (9·3% [8·7–9·9]) and, at the regional level, in Oceania (12·3% [11·5–13·0]). Nationally, Qatar had the world's highest age-specific prevalence of diabetes, at 76·1% (73·1–79·5) in individuals aged 75–79 years. Total diabetes prevalence—especially among older adults—primarily reflects type 2 diabetes, which in 2021 accounted for 96·0% (95·1–96·8) of diabetes cases and 95·4% (94·9–95·9) of diabetes DALYs worldwide. In 2021, 52·2% (25·5–71·8) of global type 2 diabetes DALYs were attributable to high BMI. The contribution of high BMI to type 2 diabetes DALYs rose by 24·3% (18·5–30·4) worldwide between 1990 and 2021. By 2050, more than 1·31 billion (1·22–1·39) people are projected to have diabetes, with expected age-standardised total diabetes prevalence rates greater than 10% in two super-regions: 16·8% (16·1–17·6) in north Africa and the Middle East and 11·3% (10·8–11·9) in Latin America and Caribbean. By 2050, 89 (43·6%) of 204 countries and territories will have an age-standardised rate greater than 10%.

**Interpretation:**

Diabetes remains a substantial public health issue. Type 2 diabetes, which makes up the bulk of diabetes cases, is largely preventable and, in some cases, potentially reversible if identified and managed early in the disease course. However, all evidence indicates that diabetes prevalence is increasing worldwide, primarily due to a rise in obesity caused by multiple factors. Preventing and controlling type 2 diabetes remains an ongoing challenge. It is essential to better understand disparities in risk factor profiles and diabetes burden across populations, to inform strategies to successfully control diabetes risk factors within the context of multiple and complex drivers.

**Funding:**

Bill & Melinda Gates Foundation.

**Research in context**

**Evidence before this study**

The Global Burden of Diseases, Injuries, and Risk Factors Study (GBD) generates publicly available estimates of total (inclusive of type 1 and type 2) diabetes deaths, prevalence, years of life lost (YLLs), years lived with disability (YLDs), and disability-adjusted life-years (DALYs) at the global, super-region, region, and country and territory levels. Since GBD 2017, type-specific estimates have also been produced. The International Diabetes Federation (IDF) generates worldwide estimates of diabetes deaths and prevalence for type 1 diabetes in people aged 19 years or younger and for total diabetes in those aged 20–79 years, with the most recent estimates produced in 2021, and has projected the future prevalence of total diabetes through 2045. The NCD Risk Factor Collaboration (NCD-RisC) published global estimates in 2016 that focused on total diabetes prevalence in individuals aged 18 years and older and projected the probability that diabetes would not continue to increase by 2025. In the present study, we estimated non-fatal outcomes due to diabetes by conducting systematic reviews in PubMed from Jan 1, 1990, to Oct 16, 2018 (see appendix section 4.1.1), carrying out opportunistic searches from Jan 1, 1990, to Dec 31, 2021, and incorporating data shared by country collaborators and WHO in addition to insurance claims data. To estimate diabetes risk relative to risk factor exposure, separate systematic reviews were done for each risk factor by accessing various databases (PubMed, Embase, and Web of Science) with endpoints ranging from 2019 to 2022 (see appendix sections 5.1.1–5.1.6).

**Added value of this study**

Global estimates are essential to policy makers, health-care professionals, health researchers, and individuals with diabetes, but only GBD data and methods are exhaustive across diabetes type, age, and sex, for 204 countries and territories; explicitly quantify the proportion of the diabetes burden attributable to specific risk factors; predict diabetes prevalence to 2050; and are designed to capture both undiagnosed and diagnosed cases. Various research groups have made use of publicly available GBD data to report on the diabetes burden and risk factors and produce short-term forecasts. Our study, as part of the larger GBD analytical enterprise, leverages the newest available data and methods. We apply and detail the updated GBD analytical and evidentiary framework to generate comprehensive, type-specific estimates of diabetes burden for all regions of the world, across the human lifespan, for males and females separately and combined. We also quantify the proportion of type 2 diabetes attributable to 16 selected risk factors concurrently to highlight the main drivers of diabetes. The continued global spread of diabetes presents a massive public health challenge. The location-specific and population-specific data we present on the likely trajectory of diabetes in the coming decades are crucial to inform policy makers and public health professionals as they prepare to address the impending threat to the communities they serve.

**Implications of all the available evidence**

Policy makers and public health officials worldwide are increasingly concerned by soaring diabetes prevalence rates and their implications for health-care systems and societies. At the current pace, we project that more than 1·31 billion people will be living with diabetes by 2050, most of whom will have type 2 diabetes. Addressing escalating challenges to diabetes prevention and barriers to managing the disease and its complications will become a requisite component of health-care provision worldwide. There is an urgent need to tackle adverse trends in the prevalence of risk factors for type 2 diabetes, particularly obesity. Without new and far-reaching approaches targeting not only risk factors but also the social and logistical barriers that limit access to treatment and medical attention, diabetes will continue to exert increasingly negative effects on the quality of life of individuals, health of populations, and the strength of global economies for decades to come.

## Introduction

Diabetes is a serious, chronic disease characterised by elevated blood glucose concentrations related to the effects of abnormal β-cell biology on insulin action.^1^, ^2^, ^3^, ^4^, ^5^ According to estimates from the Global Burden of Diseases, Injuries, and Risk Factors Study (GBD) 2019, diabetes was the eighth leading cause of death and disability combined in the world, with nearly 460 million people across every country and age group living with the disease in 2019.^6^ Diabetes represents a substantial burden to health-care systems,^2^, ^7^, ^8^, ^9^ with estimates by the International Diabetes Federation (IDF) indicating that 537 million people worldwide had diabetes in 2021, resulting in health expenditures of US$966 billion globally, forecast to reach more than $1054 billion by 2045.^4^, ^10^ The 2016 NCD Risk Factor Collaboration (NCD-RisC) Study projected that the probability of meeting global targets to halt the rising diabetes prevalence by 2025 was lower than 1% for women and even lower for men.^11^ Diabetes is also a major risk factor for ischaemic heart disease and stroke,^12^ which were estimated by GBD 2019 to be the first and second leading causes, respectively, of the global disease burden.^6^

Type 1 and type 2 diabetes are the most common forms of the disease and are diagnosed through well established criteria.^1^, ^2^, ^4^ Type 1 diabetes often develops during childhood, while type 2 diabetes has a strong genetic component and a robust association with obesity and a sedentary lifestyle.^10^, ^13^ Although prevention and management approaches differ between diabetes types, there are well established strategies to reduce the disease burden, including limiting risk factors for type 2 diabetes,^2^ increasing access to treatment such as insulin,^14^ and enhancing the health-system infrastructure.^2^, ^14^, ^15^ However, social determinants of health have led to considerable disparities across populations in risk factor profiles, access to screening and treatment, and available health services.^16^, ^17^, ^18^, ^19^ Hence, the burden of diabetes-related deaths and disability, as well as their drivers, varies widely.^14^, ^20^, ^21^, ^22^, ^23^, ^24^, ^25^, ^26^ The *Lancet* Commission on diabetes published in 2020 highlights the unequal burden of the disease on people in low-income and middle-income countries (LMICs), reporting that 80% of diabetes cases occur in LMICs.^2^ The *Lancet* Commission noted that, in addition to underfunded and ill-prepared health-care systems, LMICs are beset by socioeconomic challenges such as poor nutrition, poverty, and physical inactivity, and emphasised the pressing need for accurate, focused data to guide the development of effective programmes targeting these factors. It was further argued as imperative to accurately identify and characterise the populations at highest risk—defined by their demographic features and exposure to key risk factors—in addition to forecasting how the diabetes burden is expected to increase along these dimensions in the future.

In response to this need and in support of recent calls to action sounded by the global community, as embodied in initiatives such as the 2020 *Lancet* Commission on diabetes and the 2021 WHO Global Diabetes Compact, our work applies and explicates the newly updated methodological framework of GBD to generate estimates of total diabetes and type-specific (type 1 and type 2) diabetes prevalence and burden from 1990 to 2021. This approach allows us to break down these estimates with a high degree of granularity by location, age, and sex, and to present a more holistic picture of the landscape of diabetes—including drivers of the disease and how they have changed over time, as well as forecasting global and location-specific diabetes prevalence through 2050.

This manuscript was produced as part of the GBD Collaborator Network and in accordance with the GBD Protocol.^27^

## Methods

### Overview

To obtain the data used in models, GBD conducts systematic reviews and opportunistic searches, and utilises data shared by country collaborators and WHO. Data seeking is iterative and continuously in process in order to identify new sources. Information on data seeking efforts conducted for GBD iterations through GBD 2019 has been published previously^6^, ^28^, ^29^ and is provided in the appendix (section 4.1.1). For this study, we identified 27 193 data sources to which we applied the methodological and evidentiary framework provided by GBD. The present analysis does not reflect the potential impact of the COVID-19 pandemic on diabetes prevalence and burden since these data were not available at the time of the analysis.

We report primarily on diabetes prevalence and burden because these metrics are particularly salient for characterising type 2 diabetes and capturing aspects of the rapid global rise of diabetes; however, we also provide mortality estimates in the appendix (table S24). Moreover, mortality data were included in the calculation of our principal metrics: prevalence (via cause-specific mortality rates used in the compartmental disease modelling process) and years of life lost (YLLs; via measures of expected age of mortality), and, by extension, disability-adjusted life-years (DALYs), which are the sum of YLLs and years lived with disability (YLDs).

We report many estimates generated as age-standardised results (ie, extrapolated to a hypothetical population with a standardised age structure) to allow comparison of estimates made in populations with different age structures. The standard population was calculated with the non-weighted mean of the age-specific population proportional distributions for all national locations with populations greater than 5 million in 2019 from GBD 2019.

This study complies with the Guidelines for Accurate and Transparent Health Estimates Reporting (GATHER) statement (appendix table S1).^30^

### Mortality

There were 25 666 location-years of death data included in the fatal model (appendix section 3.1). We used vital registration data and verbal autopsy^6^ data coded as diabetes since 1980 (appendix section 3.2.1). Codes for causes that either did not lead to death or were intermediate causes, but for which diabetes could have been the underlying cause—referred to as garbage codes^31^—were eligible for inclusion in the diabetes model^6^ (appendix section 3.2.1). Approximately 11·1% of deaths coded to diabetes were reassigned from garbage codes.

More than 50% of deaths coded to diabetes did not specify a type. We developed a log-linear regression model to predict the type-specific proportion of deaths among those coded to unspecified diabetes. The model was informed by data that specified the diabetes type and, for a given country, included two parameters: first, country-years in which more than 50% of deaths due to diabetes were coded as being due to type 1 or type 2 diabetes; and second, for country-years with type-specific coding, those in which 70% or more of type-specific deaths for people older than 25 years were coded as type 2 diabetes. We included prevalence of obesity as a covariate and redistributed deaths accordingly to type 1 or type 2 diabetes. More details are provided in the appendix (section 3.2.3).

We ran separate mortality models for type 1 diabetes, type 2 diabetes, and total diabetes. We assumed that all deaths due to diabetes in people younger than 15 years were from type 1 diabetes. We used the Cause of Death Ensemble model (CODEm),^6^ a highly automated analytical tool that selects an ensemble of mixed-effects or spatiotemporal Gaussian regression models of mortality rates or cause fractions with varying combinations of predictive covariates. Ensembles were selected on the basis of out-of-sample predictive validity testing. We included 19 covariates, six associated with type 1 diabetes and 13 associated with type 2 diabetes, which were selected on the basis of known or postulated relationships with development or management of diabetes (appendix section 3.3).

### Non-fatal outcomes

The reference case definition for diabetes was a fasting plasma glucose (FPG) concentration of 7 mmol/L (126 mg/dL) or greater, or a person using insulin or diabetes medication. We included any population-representative source that provided individual-level data or reported the prevalence or incidence of diabetes defined by the source's glucose threshold from tests of FPG, glycated haemoglobin (HbA_1c_), oral glucose tolerance (OGTT), or post-prandial glucose (PPG), or any population-representative source that reported mean FPG and uncertainty around the estimate.^32^ We also used insurance claims data from the USA and Taiwan (province of China), since these were locations for which we had access to insurance claims. We included studies reporting the incidence of type 1 diabetes. We incorporated data found through systematic reviews conducted from Jan 1, 1990, to Oct 16, 2018 (appendix section 4.1.1),^6^, ^28^, ^29^ and carried out opportunistic literature searches from Jan 1, 1990, to Dec 31, 2021. Between 2020 and 2021, we reviewed all data provided by GBD collaborators through the Global Health Data Exchange (GHDx) and prospectively sought individual-level data from the WHO STEPwise Approach to NCD Risk Factor Surveillance (STEPS) surveys (appendix section 4.1.1). There were 1527 location-years of data from 172 countries (84·3% of the 204 countries and territories included in GBD) used in the diabetes modelling process (appendix section 4.1.1).

Where possible, we used individual-level data from surveys that collected glucose measurements to calculate age-sex-year-location-specific prevalence estimates and used the information included in the survey metadata to inform how the sampling strategy, sampling frame, and sampling weights were incorporated into the estimates and uncertainty.

We used the meta-regression Bayesian, regularised, trimmed (MR-BRT)^33^ tool to generate coefficients that were used to adjust estimates from studies that did not define diabetes with the reference definition (appendix section 4.2.2). For data from people aged younger than 15 years, we assumed that all diabetes cases were type 1 diabetes and that all patients had sought hospital care due to their insulin dependence. We also converted population-level mean FPG estimates to diabetes prevalence estimates (appendix section 4.2.2).

We ran separate non-fatal models for type 1 diabetes and total diabetes. We used a hierarchical Bayesian meta-regression modelling tool, DisMod-MR 2.1,^6^ to estimate prevalence due to diabetes from 1990 to 2021. Differential equations in DisMod-MR 2.1 produce a consistent set of estimates based on data on prevalence, incidence, and cause-specific mortality rates generated from the fatal modelling process. In the type 1 diabetes model, we included three predictive covariates: proportion of livebirths in women aged 35 years and older and maternal education (years per capita) as predictors of type 1 diabetes incidence; and the Healthcare Access and Quality Index (HAQ Index)^34^ as a predictor of type 1 diabetes excess mortality rate. We assumed there was no remission (ie, no cure). In the total diabetes model, we included two predictive covariates—prevalence of obesity and year as predictors of diabetes prevalence—and assumed that annual remission could be no more than 1% (appendix sections 4.3.1 and 4.3.2). Because most data sources in adults reported prevalence of total diabetes or did not use a robust strategy to exclude people with type 1 diabetes, we were not confident in the accuracy of the data available that were labelled as type 2 diabetes. As an alternative, we subtracted the year-age-sex-location-specific estimates of type 1 diabetes from total diabetes to produce estimates of type 2 diabetes (appendix section 4.3.3).

### YLLs, YLDs, and DALYs

The methods for calculating YLLs, YLDs, and DALYs have been described elsewhere,^6^ but in brief, YLLs were the product of the number of deaths and standard life expectancy at each age of death,^35^ and YLDs were the product of the prevalence of each sequela and its corresponding disability weight.^36^ We included estimates for four diabetic sequelae for each type of diabetes: neuropathy, diabetic foot, lower limb amputation, and vision loss due to retinopathy. Each sequela had separate disability weights that were used to calculate YLDs (appendix sections 4.2.4, 4.3.4, and 4.4). YLDs were corrected for comorbidities with all other causes of ill health, assuming independence and a multiplicative function. DALYs were the sum of YLLs and YLDs.

### Risk-attributable burden

We modelled 16 detailed risk factors for diabetes: ambient particulate matter pollution, household air pollution from solid fuels, smoking, second-hand smoke, high alcohol use, high body-mass index (BMI), diet low in fruits, diet low in vegetables, diet low in whole grains, diet high in red meat, diet high in processed meat, diet high in sugar-sweetened beverages, diet low in fibre, low physical activity, high air temperature, and low air temperature (appendix section 5.1). These risk factors fall into six categories: environmental or occupational, tobacco use, high alcohol use, high BMI, dietary risks, and low physical activity. All risk factors have been shown to be associated with type 2 diabetes, but high air temperature and low air temperature are the only risk factors associated with type 1 diabetes.^28^

To quantify the relationship between each risk factor and diabetes, we carried out meta-analyses following the comparative risk assessment approach, a framework used by GBD since 2002 that is predicated on a causal web of hierarchically organised, potentially overlapping health risks.^29^, ^37^ For each risk factor analysed here, we estimated the relative risk of diabetes as a function of risk exposure, using the following methods, which have been extensively detailed elsewhere.^33^, ^38^ In brief, we did a literature review of studies that estimated diabetes risk relative to risk factor exposure and extracted data to input into a set of flexible meta-regression procedures using regularised splines to estimate risk functions, as an alternative to imposing a log-linear risk–outcome relationship. Accuracy was further improved by using a robust likelihood-based approach—least-trimmed squares—to detect and trim 10% of the outlying data, testing and adjusting for bias related to study design, and integrating over exposure ranges to account for inconsistency in exposure levels between data sources.

Following methods established previously,^28^ we used DisMod-MR 2.1 or spatiotemporal Gaussian process regression to estimate exposure distributions for each risk factor by age, sex, year, and location, and further determined the theoretical minimum risk exposure level (TMREL), the counterfactual level of exposure that would minimise the risk of diabetes, on the basis of epidemiological evidence. Exposure, relative risk estimates, and TMRELs were used to calculate population attributable fractions (PAFs) for each risk factor by location, age, sex, and year. PAFs quantify the proportional reduction in diabetes that would occur if exposure to the given risk factor was reduced to the TMREL. PAFs were multiplied by metrics of disease burden—in this instance, DALYs—to estimate the risk-attributable burden.

### Forecasting

We used forecasted Socio-demographic Index (SDI)^39^ as a predictor in a regression model to estimate the prevalence of type 1 diabetes and forecasted BMI as the predictor for estimating the prevalence of type 2 diabetes. These metrics were forecast through 2050, by age, sex, year, and location.^40^ For each location (*l*), age (*a*), sex (*s*), and year (*y*), we logit-transformed the GBD 2019 diabetes prevalence estimates *logit*(*Y*_l,a,s,y_) and used a fixed coefficient (β_1_) on SDI only for type 1 diabetes (equation 1)

$$E[\log it(Y_{l,a,s,\gamma })]=\beta_{1}SDI+\alpha_{l,a,s}$$

and BMI for type 2 diabetes (equation 2) over time, and a random intercept (α).

$$E[\log it(Y_{l,a,s,\gamma })]=\beta_{1}BMI+\alpha_{l,a,s}$$

We computed the difference between the GBD estimates in rate space and the forecasted estimates for 2021 and shifted the forecasting trend through 2050 to align with that of GBD. To calculate the number of cases, we used the forecasted population multiplied by the corresponding predicted prevalence. Population forecasts are described by Vollset and colleagues.^40^

### Uncertainty and presentation of results

At each modelling step described above, parameter uncertainty was incorporated by randomly drawing 100 samples from each age-sex-location-year-specific parameter distribution and propagating this uncertainty forward through each subsequent step of the analysis. Likewise, 95% uncertainty intervals (UIs) for final estimates were calculated by generating 100 random draws from the estimate distribution and taking the 2·5th and 97·5th percentile values across the 100 draws.

All count data reported are presented to three significant figures, while rates and percentages are presented to one decimal place.

### Geographical locations reported

Diabetes estimates were generated for 204 countries and territories that are grouped on the basis of epidemiological patterns into seven super-regions, with these super-regions further grouped into 21 regions based on geographical and epidemiological similarity (see appendix section 7, table S18, for the full GBD location hierarchy).

### Code availability

All codes used for these analyses are publicly available online. Analyses were carried out with R (version 4.2.2).

### Role of the funding source

The funder of the study had no role in study design, data collection, data analysis, data interpretation, or the writing of the report.

## Results

### Total diabetes prevalence

**Figure 1:**
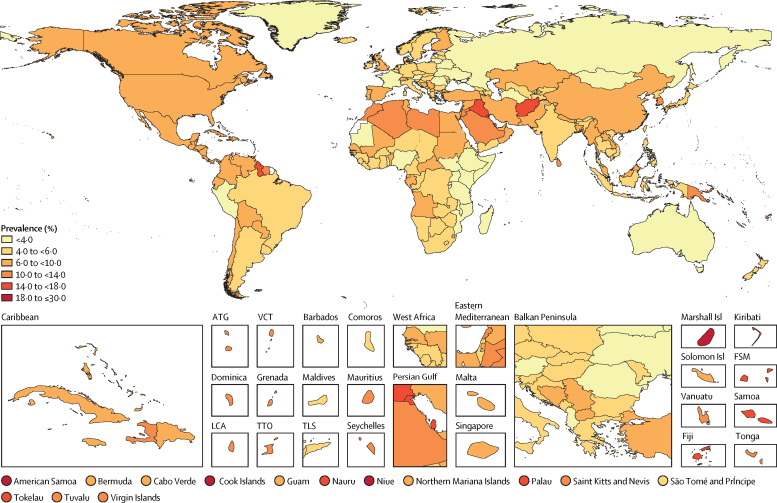
Age-standardised total diabetes prevalence rates in 2021 ATG=Antigua and Barbuda. VCT=Saint Vincent and the Grenadines. LCA=Saint Lucia. TTO=Trinidad and Tobago. Isl=Islands. FSM=Federated States of Micronesia. TLS=Timor-Leste.

In 2021, there were 529 million (95% UI 500–564) people of all ages, worldwide, living with diabetes, yielding a global age-standardised prevalence of 6·1% (5·8–6·5; figure 1). To facilitate comparison, we re-stratified our results into age groups reported by IDF and NCD-RisC.^4^, ^11^ We estimated there were 485 million (456–517) adults aged 20–79 years with diabetes in 2021 (for comparison with the 2021 IDF estimate of 537 million in the same age group), and 321 million (304–341) people aged 18 years and older with diabetes in 2010 (for rough comparison with the 2014 NCD-RisC estimate of 422 million in the same age group; appendix table S20). Age-standardised total diabetes prevalence rates varied at the super-region level; north Africa and the Middle East had an age-standardised total diabetes prevalence rate of 9·3% (8·7–9·9), with country-specific rates of more than 10% in 11 countries in this region: Iraq (15·3%; 14·3–16·2), Kuwait (15·2%; 14·1–16·3), Qatar (15·1%; 14·0–16·2), Bahrain (15·0%; 14·1–15·8), Afghanistan (14·6%; 13·5–15·5), Morocco (13·8%; 12·7–14·7), Jordan (13·5%; 12·6–14·5), Saudi Arabia (11·3%; 10·6–12·0), Lebanon (11·1%; 10·3–11·8), Libya (10·6%; 9·9–11·6), and Algeria (10·0%; 9·3–10·7). Oceania had the highest regional age-standardised prevalence, at 12·3% (11·5–13·0), where 15 of 18 countries and territories had a prevalence greater than 10%; age-standardised prevalence rates were greater than 20% in the Marshall Islands, at 22·2% (20·7–23·9), and American Samoa, at 21·4% (19·9–22·7). Eastern sub-Saharan Africa had the lowest regional diabetes prevalence, at 2·9% (2·7–3·1). The age-standardised diabetes prevalence exceeded 10% in 43 countries and territories in 2021 (figure 1).

### Sex-specific total diabetes prevalence

The global age-standardised total diabetes prevalence was higher in males than in females (6·5% [95% UI 6·2–7·0] *vs* 5·8% [5·4–6·1]), with a male-to-female sex ratio of 1·14 (1·13–1·15). The ratio varied geographically, from 1·26 (1·24–1·28) in the high-income super-region to 0·96 (0·94–0·97) in the Latin America and Caribbean super-region. At the regional level, the age-standardised diabetes prevalence in males was 1·40 (1·30–1·48) times higher than in females in central sub-Saharan Africa, but prevalence among females was more than 10% higher than in males in central Latin America, southern sub-Saharan Africa, and the Caribbean. In 64 (31·4%) countries and territories, age-standardised diabetes prevalence was lower in males than in females, and in six countries—Azerbaijan, Haiti, Laos, Mauritania, Zimbabwe, and Belize—prevalence in males was more than 20% lower than in females. Of the 140 (68·6%) countries and territories where diabetes was more prevalent in males than in females, in three countries—Angola, Uganda, and Gabon—the male prevalence was more than 50% higher than the female prevalence (appendix figure S24).

### Age-specific total diabetes prevalence

**Figure 2:**
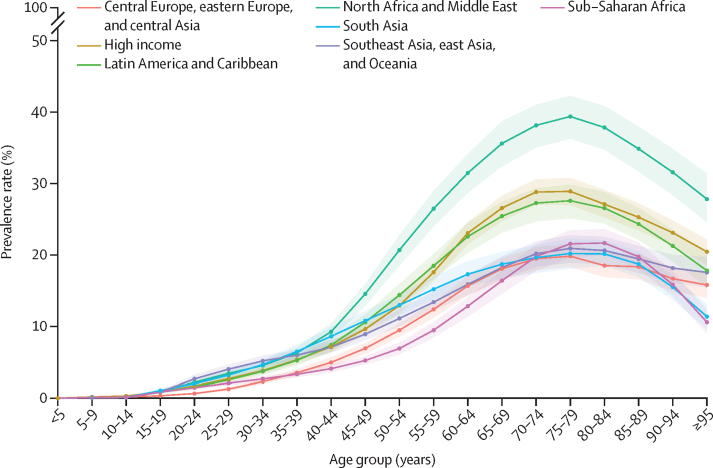
Prevalence of total diabetes by age and GBD super-region in 2021 The shaded areas represent 95% uncertainty intervals. GBD=Global Burden of Diseases, Injuries, and Risk Factors Study.

Globally, total diabetes prevalence exceeded 20% in every age group between 65–95 years but was less than 1% in age groups younger than 20 years. Global diabetes prevalence peaked between ages 75–79 years, at 24·4% (95% UI 22·3–26·2). In this age group, prevalence was highest in the north Africa and Middle East super-region, at 39·4% (36·3–42·3), and lowest in central Europe, eastern Europe, and central Asia, at 19·8% (18·3–21·6; figure 2). At the regional level, Oceania had the highest age-specific total diabetes prevalence in the world, at 43·0% (40·7–45·9) in people aged 75–79 years. The highest country-level prevalence was found in Qatar, at 76·1% (73·1–79·5) in people aged 75–79 years. In people aged 30–34 years, ten countries and territories—all in Oceania—had an age-specific prevalence that exceeded 10%: Marshall Islands (19·5% [16·8–23·1]), American Samoa (17·3% [14·8–20·3]), Cook Islands (15·1% [12·9–17·9]), Niue (14·9% [12·8–17·4]), Palau (13·6% [11·9–15·9]), Tokelau (13·1% [11·4–15·2]), Samoa (12·9% [11·0–15·1]), Nauru (12·1% [10·5–13·8]), Federated States of Micronesia (10·6% [9·2–12·2]), and Kiribati (10·4% [9·0–11·8]; appendix figure S23).

### Type-specific diabetes prevalence

Type 2 diabetes cases made up 96·0% (95% UI 95·1–96·8) of all diabetes cases. More than 90% of the age-standardised diabetes prevalence rate in every super-region was due to type 2 diabetes. In two regions, less than 90% of diabetes cases were due to type 2 diabetes: 86·4% (83·3–89·4) in Australasia and 89·3% (87·0–91·5) in western Europe. Type 2 diabetes prevalence made up more than 80% of total diabetes cases in all 204 countries and territories and more than 90% of total diabetes cases in 183 (89·7%) countries and territories. There was no difference in type-specific breakdown by sex.

### Total diabetes burden: YLLs, YLDs, and DALYs

Globally, there were 37·8 million (95% UI 35·4–40·2) total diabetes-related YLLs and 41·4 million (29·5–55·4) YLDs, yielding 79·2 million (67·8–92·5) DALYs due to diabetes in 2021 (table; appendix table S21). Type 2 diabetes made up 94·0% (93·6–94·6) of diabetes YLLs, 96·6% (96·0–97·3) of YLDs, and 95·4% (94·9–95·9) of DALYs. The global age-standardised diabetes DALY rate was 915·0 (782·6–1067·5) per 100 000, the YLL rate was 437·4 (409·2–464·1) per 100 000, and the YLD rate was 477·6 (340·7–637·4) per 100 000.

**Table:** DALY counts and age-standardised DALY rates per 100 000 population and the corresponding percentage change in DALY counts and age-standardised DALY rates between 1990 and 2021 for diabetes globally, in 21 GBD regions and 204 countries

		**DALY count in 2021 (thousands)**	**Percentage change in DALY count, 1990–2021 (%)**	**Age-standardised DALY rate in 2021 (per 100 000)**	**Percentage change in age-standardised DALY rate, 1990–2021 (%)**
**Global**		**79 200 (67 800 to 92 500)**	**189·8% (171·1 to 203·4)**	**915·0 (782·6 to 1067·4)**	**38·6% (29·7 to 45·3)**
**Central Europe, eastern Europe, and central Asia**		**4370 (3670 to 5230)**	**126·9% (119·3 to 132·4)**	**700·0 (588·3 to 840·2)**	**70·8% (65·4 to 75·1)**
Central Asia		800 (675 to 970)	236·0% (211·0 to 257·1)	923·6 (780·8 to 1119·9)	96·6% (82·6 to 108·9)
	Armenia	32·3 (27·0 to 39·3)	52·3% (38·8 to 67·0)	771·2 (646·7 to 942·3)	4·5% (−4·7 to 14·9)
	Azerbaijan	97·7 (77·0 to 122)	249·3% (196·6 to 319·7)	870·6 (689·0 to 1090·4)	72·0% (45·0 to 108·0)
	Georgia	49·4 (40·1 to 60·8)	57·6% (42·9 to 78·0)	903·5 (732·2 to 1121·7)	81·4% (65·6 to 104·6)
	Kazakhstan	144 (111 to 180)	142·5% (118·1 to 164·1)	750·2 (581·8 to 932·6)	70·4% (53·5 to 85·2)
	Kyrgyzstan	30·3 (23·7 to 38·0)	199·3% (172·3 to 226·4)	560·7 (436·3 to 696·9)	76·5% (61·8 to 91·6)
	Mongolia	17·2 (13·6 to 21·1)	337·2% (268·5 to 419·9)	564·3 (448·0 to 688·7)	76·7% (48·4 to 112·3)
	Tajikistan	52·6 (43·1 to 64·1)	237·8% (182·8 to 307·7)	801·9 (659·3 to 955·8)	63·2% (36·7 to 96·7)
	Turkmenistan	44·3 (35·8 to 53·2)	357·2% (286·9 to 446·2)	929·3 (757·9 to 1110·5)	112·3% (80·2 to 153·5)
	Uzbekistan	341 (292 to 411)	479·8% (418·0 to 538·7)	1147·1 (980·2 to 1375·1)	150·9% (124·3 to 175·8)
Central Europe		1550 (1250 to 1890)	73·7% (63·6 to 82·0)	748·1 (598·1 to 913·2)	23·0% (15·3 to 29·4)
	Albania	17·8 (13·7 to 23·2)	147·8% (119·1 to 175·6)	417·5 (323·5 to 544·8)	26·9% (12·3 to 40·5)
	Bosnia and Herzegovina	75·2 (61·2 to 91·0)	171·1% (134·0 to 204·2)	1253·6 (1022·6 to 1527·9)	88·3% (62·4 to 111·5)
	Bulgaria	115 (89·8 to 138)	35·5% (23·2 to 48·7)	868·1 (675·6 to 1051·7)	24·6% (13·5 to 36·2)
	Croatia	62·7 (49·6 to 75·9)	73·0% (58·1 to 84·2)	743·1 (580·3 to 903·3)	29·7% (18·3 to 37·8)
	Czechia	171 (136 to 211)	106·7% (87·4 to 125·6)	829·8 (659·6 to 1029·7)	35·7% (22·9 to 47·6)
	Hungary	136 (109 to 170)	55·8% (43·0 to 69·8)	747·5 (594·9 to 932·2)	22·5% (12·9 to 32·3)
	Montenegro	8·79 (7·12 to 10·7)	118·1% (95·9 to 139·0)	896·2 (728·1 to 1091·2)	39·7% (26·3 to 52·0)
	North Macedonia	41·7 (32·6 to 51·1)	153·4% (116·8 to 190·3)	1268·1 (1000·4 to 1554·4)	46·4% (26·1 to 68·5)
	Poland	520 (427 to 632)	76·6% (66·0 to 86·6)	764·5 (626·8 to 926·1)	13·5% (6·0 to 20·7)
	Romania	167 (130 to 208)	47·7% (32·0 to 61·6)	482·9 (374·8 to 607·4)	18·8% (5·3 to 30·8)
	Serbia	161 (127 to 197)	73·7% (56·3 to 95·3)	1020·2 (794·1 to 1249·1)	24·1% (11·2 to 40·2)
	Slovakia	52·0 (40·1 to 64·2)	62·0% (43·5 to 79·3)	565·3 (438·2 to 700·8)	4·8% (−7·5 to 15·4)
	Slovenia	22·1 (17·5 to 27·6)	68·7% (54·6 to 82·3)	540·8 (423·5 to 678·6)	0·0% (−9·2 to 8·5)
Eastern Europe		2020 (1730 to 2370)	153·9% (144·0 to 165·1)	596·8 (508·3 to 706·5)	104·6% (97·4 to 112·7)
	Belarus	52·3 (41·0 to 65·9)	59·8% (44·9 to 73·7)	353·4 (281·1 to 444·3)	36·7% (25·0 to 48·6)
	Estonia	14·5 (11·9 to 18·0)	130·0% (115·0 to 147·7)	635·2 (517·4 to 798·5)	96·5% (84·4 to 110·4)
	Latvia	23·8 (19·8 to 28·8)	105·3% (90·9 to 119·9)	702·4 (588·1 to 852·5)	104·7% (89·8 to 120·0)
	Lithuania	26·9 (22·3 to 33·3)	132·9% (111·9 to 149·3)	551·1 (454·0 to 687·3)	107·7% (90·0 to 123·5)
	Moldova	36·6 (29·3 to 46·3)	95·9% (81·0 to 112·7)	668·0 (537·5 to 847·3)	62·0% (49·9 to 75·0)
	Russia	1580 (1370 to 1830)	206·7% (191·9 to 225·4)	671·2 (583·8 to 780·4)	131·4% (121·6 to 145·0)
	Ukraine	285 (217 to 354)	42·8% (29·1 to 56·9)	409·4 (312·0 to 504·9)	38·4% (24·3 to 52·3)
**High income**		**12 800 (10 200 to 15 700)**	**114·7% (94·6 to 132·5)**	**676·9 (536·0 to 839·8)**	**31·6% (19·1 to 43·4)**
Australasia		226 (183 to 288)	140·8% (122·2 to 164·8)	469·2 (378·1 to 602·4)	15·3% (5·5 to 27·1)
Australia		188 (152 to 240)	148·1% (126·5 to 175·4)	462·3 (373·1 to 597·4)	17·8% (7·1 to 32·0)
New Zealand		38·6 (30·8 to 46·9)	111·0% (91·4 to 126·4)	503·2 (399·3 to 616·2)	4·6% (−5·1 to 13·6)
High-income Asia Pacific		2340 (1780 to 2980)	133·5% (110·4 to 154·9)	642·5 (487·3 to 829·0)	29·3% (14·9 to 42·3)
	Brunei	8·37 (7·00 to 9·97)	213·7% (155·8 to 259·6)	2279·7 (1946·8 to 2686·7)	−7·7% (−23·2 to 5·8)
	Japan	1400 (1050 to 1800)	99·3% (83·1 to 118·5)	512·8 (381·4 to 665·9)	21·4% (8·4 to 34·5)
	Singapore	56·7 (40·2 to 78·0)	179·9% (133·2 to 221·4)	661·1 (467·9 to 910·6)	−21·2% (−34·5 to −9·3)
	South Korea	879 (670 to 1130)	215·6% (172·8 to 257·4)	966·4 (737·7 to 1251·9)	16·4% (0·0 to 33·5)
High-income North America		5470 (4400 to 6580)	170·0% (147·2 to 188·3)	928·6 (744·8 to 1122·5)	53·6% (40·6 to 63·6)
	Canada	435 (335 to 562)	205·2% (168·7 to 246·4)	668·1 (519·4 to 859·5)	49·9% (32·1 to 69·8)
	Greenland	0·361 (0·282 to 0·435)	136·5% (91·6 to 180·5)	492·5 (386·3 to 591·1)	21·3% (−0·3 to 44·5)
	USA	5040 (4060 to 6010)	168·1% (145·4 to 185·2)	958·5 (770·8 to 1150·9)	53·9% (41·0 to 63·6)
Southern Latin America		648 (524 to 802)	105·9% (86·1 to 126·9)	762·3 (617·3 to 946·0)	12·7% (1·6 to 24·4)
	Argentina	426 (350 to 522)	81·8% (62·6 to 99·7)	780·1 (639·2 to 957·3)	8·0% (−3·5 to 18·7)
	Chile	183 (146 to 233)	205·6% (172·5 to 248·0)	725·1 (577·6 to 923·6)	24·1% (10·7 to 41·0)
	Uruguay	38·7 (32·3 to 47·0)	90·9% (76·4 to 111·7)	753·0 (623·6 to 919·4)	42·7% (31·5 to 58·3)
Western Europe		4070 (3280 to 5030)	62·6% (47·1 to 77·4)	511·8 (402·0 to 648·3)	13·2% (1·6 to 25·9)
	Andorra	0·724 (0·579 to 0·941)	212·5% (148·7 to 282·8)	510·6 (404·9 to 665·7)	22·8% (−2·3 to 50·4)
	Austria	66·6 (54·3 to 79·9)	49·2% (33·0 to 65·7)	402·0 (322·2 to 492·3)	3·5% (−9·2 to 16·9)
	Belgium	96·7 (73·1 to 129)	61·1% (41·8 to 80·6)	494·3 (372·4 to 669·1)	19·6% (4·3 to 35·0)
	Cyprus	17·2 (14·2 to 21·5)	58·3% (36·0 to 79·9)	873·5 (722·7 to 1091·7)	−39·6% (−48·3 to −31·4)
	Denmark	46·1 (38·3 to 55·6)	76·9% (59·3 to 94·3)	440·8 (359·4 to 538·1)	24·0% (11·3 to 37·0)
	Finland	55·7 (41·6 to 72·8)	101·8% (87·3 to 116·7)	577·7 (427·5 to 756·2)	39·3% (28·1 to 50·6)
	France	426 (345 to 524)	90·3% (74·4 to 108·1)	351·7 (278·2 to 445·7)	25·0% (13·1 to 37·8)
	Germany	804 (662 to 966)	56·8% (40·0 to 73·5)	482·1 (390·3 to 593·3)	15·1% (2·3 to 28·9)
	Greece	102 (77·2 to 132)	88·6% (77·0 to 101·5)	534·8 (399·5 to 703·1)	42·6% (32·1 to 52·7)
	Iceland	2·10 (1·57 to 2·72)	188·7% (156·8 to 211·8)	408·8 (303·2 to 540·4)	55·7% (38·0 to 68·4)
	Ireland	27·7 (21·4 to 36·3)	81·2% (58·9 to 104·1)	386·6 (295·5 to 505·8)	0·8% (−11·6 to 13·9)
	Israel	81·9 (67·3 to 98·8)	159·1% (141·9 to 176·2)	690·7 (567·3 to 839·3)	4·7% (−2·4 to 11·7)
	Italy	665 (557 to 792)	31·0% (20·5 to 42·4)	521·1 (422·4 to 637·8)	−11·5% (−20·3 to −2·5)
	Luxembourg	4·30 (3·31 to 5·48)	109·4% (81·7 to 129·5)	440·0 (336·1 to 565·0)	11·9% (−3·5 to 23·1)
	Malta	6·28 (5·03 to 7·94)	127·8% (96·4 to 163·9)	738·0 (585·7 to 953·5)	13·0% (−3·7 to 32·2)
	Monaco	0·283 (0·214 to 0·359)	129·5% (104·8 to 154·5)	375·5 (275·0 to 478·4)	77·1% (61·5 to 94·7)
	Netherlands	137 (111 to 171)	32·3% (17·1 to 51·2)	445·2 (355·9 to 563·0)	−16·5% (−26·3 to −3·8)
	Norway	36·8 (29·4 to 45·5)	54·1% (46·2 to 61·4)	433·2 (339·1 to 545·4)	7·0% (1·0 to 12·8)
	Portugal	157 (124 to 199)	68·2% (48·4 to 90·8)	736·1 (573·1 to 952·9)	6·9% (−7·6 to 22·3)
	San Marino	0·232 (0·179 to 0·300)	167·6% (132·4 to 204·5)	413·3 (313·5 to 540·1)	47·9% (27·4 to 65·7)
	Spain	554 (423 to 729)	62·8% (43·1 to 82·2)	650·1 (491·1 to 868·0)	1·3% (−12·3 to 14·6)
	Sweden	84·0 (68·2 to 102)	58·2% (45·1 to 70·5)	465·0 (365·4 to 577·8)	15·9% (5·9 to 25·1)
	Switzerland	89·2 (68·0 to 118)	75·4% (53·4 to 97·7)	578·8 (435·7 to 770·7)	12·7% (−1·5 to 26·2)
	UK	601 (458 to 764)	92·4% (70·3 to 111·6)	580·3 (431·2 to 751·1)	53·5% (34·0 to 70·1)
**Latin America and Caribbean**		**9160 (7850 to 10 600)**	**193·1% (177·6 to 205·7)**	**1446·1 (1240·9 to 1673·7)**	**10·1% (4·1 to 15·0)**
Andean Latin America		582 (473 to 707)	290·7% (245·4 to 344·7)	962·1 (782·4 to 1166·8)	40·5% (24·1 to 60·0)
	Bolivia	142 (115 to 177)	257·4% (198·9 to 343·6)	1482·2 (1205·0 to 1828·0)	27·0% (6·5 to 57·4)
	Ecuador	206 (168 to 252)	359·9% (300·1 to 413·6)	1257·7 (1027·0 to 1532·8)	57·6% (37·0 to 76·0)
	Peru	233 (183 to 290)	264·7% (208·3 to 330·1)	678·6 (532·1 to 844·7)	34·8% (13·5 to 58·7)
Caribbean		924 (774 to 1140)	124·6% (105·5 to 146·5)	1722·1 (1442·8 to 2116·7)	12·9% (3·2 to 23·9)
	Antigua and Barbuda	2·36 (2·01 to 2·82)	122·3% (104·1 to 142·7)	2202·5 (1875·0 to 2613·0)	6·6% (−1·9 to 16·3)
	The Bahamas	7·60 (6·26 to 9·55)	166·7% (130·4 to 206·8)	1759·3 (1452·3 to 2185·9)	2·8% (−10·7 to 17·8)
	Barbados	9·94 (8·16 to 12·1)	61·1% (38·7 to 86·2)	2015·7 (1652·9 to 2474·1)	−7·9% (−20·6 to 6·8)
	Belize	6·79 (5·71 to 8·08)	343·1% (303·1 to 388·5)	2082·4 (1767·0 to 2459·3)	35·5% (24·4 to 48·1)
	Bermuda	1·15 (0·906 to 1·41)	68·5% (45·8 to 89·5)	928·4 (732·2 to 1144·6)	−14·4% (−26·1 to −3·1)
	Cuba	149 (116 to 196)	68·8% (50·4 to 88·4)	806·9 (626·1 to 1061·3)	−5·0% (−15·6 to 6·4)
	Dominica	2·35 (1·95 to 2·77)	60·0% (42·9 to 77·2)	2592·0 (2151·2 to 3058·7)	23·4% (10·5 to 37·1)
	Dominican Republic	159 (126 to 192)	304·1% (244·9 to 357·3)	1566·3 (1244·2 to 1882·6)	67·1% (42·6 to 89·0)
	Grenada	3·40 (2·91 to 4·01)	94·6% (74·3 to 115·1)	2908·0 (2508·7 to 3409·0)	16·6% (5·2 to 27·8)
	Guyana	24·4 (19·2 to 29·3)	108·5% (79·0 to 139·9)	3477·6 (2755·8 to 4160·9)	25·6% (8·3 to 43·2)
	Haiti	242 (196 to 320)	142·2% (91·1 to 198·8)	2931·0 (2369·9 to 3870·4)	6·9% (−15·6 to 30·8)
	Jamaica	65·4 (53·5 to 77·9)	90·0% (58·7 to 123·0)	2115·9 (1729·3 to 2520·6)	9·5% (−8·7 to 28·4)
	Puerto Rico	123 (99·5 to 154)	94·1% (76·1 to 112·1)	1934·2 (1541·0 to 2440·5)	9·9% (−0·2 to 22·0)
	Saint Kitts and Nevis	1·49 (1·22 to 1·81)	78·1% (50·3 to 103·0)	2031·5 (1681·0 to 2430·3)	−11·9% (−23·4 to −1·9)
	Saint Lucia	5·27 (4·28 to 6·35)	107·4% (85·5 to 132·6)	2309·0 (1874·8 to 2774·3)	−19·1% (−27·7 to −9·0)
	Saint Vincent and the Grenadines	3·86 (3·24 to 4·57)	85·5% (63·5 to 107·5)	2732·3 (2301·1 to 3218·6)	−4·2% (−15·2 to 6·8)
	Suriname	14·0 (11·0 to 16·7)	243·1% (194·0 to 289·5)	2140·5 (1695·3 to 2537·5)	44·4% (23·5 to 64·1)
	Trinidad and Tobago	67·3 (54·7 to 80·9)	100·5% (73·5 to 132·4)	3468·0 (2824·5 to 4171·5)	−10·8% (−22·8 to 3·3)
	Virgin Islands	3·59 (2·76 to 4·41)	146·7% (107·8 to 182·0)	2082·7 (1591·3 to 2557·8)	27·3% (5·9 to 44·1)
Central Latin America		4810 (4120 to 5540)	222·5% (202·5 to 239·1)	1865·9 (1601·9 to 2146·4)	13·9% (6·7 to 20·1)
	Colombia	470 (362 to 590)	180·0% (148·7 to 207·6)	841·3 (647·5 to 1057·6)	−2·6% (−14·5 to 7·4)
	Costa Rica	59·1 (46·2 to 74·5)	334·4% (301·0 to 362·0)	1074·4 (841·0 to 1353·2)	47·4% (36·0 to 58·1)
	El Salvador	99·5 (81·8 to 119)	269·1% (224·2 to 328·5)	1625·6 (1333·0 to 1942·8)	93·2% (70·3 to 125·1)
	Guatemala	277 (233 to 323)	737·3% (662·0 to 836·9)	2377·2 (2006·3 to 2777·2)	212·5% (182·1 to 250·2)
	Honduras	100 (80·4 to 126)	454·8% (386·5 to 533·7)	1434·0 (1156·8 to 1787·1)	85·0% (63·4 to 111·4)
	Mexico	3160 (2720 to 3530)	192·5% (173·7 to 206·9)	2451·3 (2122·5 to 2733·0)	5·3% (−1·4 to 10·5)
	Nicaragua	76·5 (63·0 to 95·4)	329·5% (283·2 to 379·5)	1498·1 (1244·5 to 1854·1)	47·1% (30·3 to 64·3)
	Panama	56·2 (45·0 to 67·2)	337·9% (289·3 to 388·2)	1265·1 (1010·5 to 1510·6)	56·3% (38·3 to 74·7)
	Venezuela	502 (402 to 605)	316·5% (256·3 to 384·2)	1597·3 (1280·8 to 1922·2)	39·1% (19·0 to 62·0)
Tropical Latin America		2850 (2460 to 3290)	165·0% (153·9 to 177·1)	1092·4 (945·9 to 1261·3)	−0·6% (−5·1 to 3·9)
	Brazil	2740 (2370 to 3160)	159·7% (149·2 to 172·1)	1075·2 (931·4 to 1239·0)	−2·7% (−7·1 to 2·1)
	Paraguay	110 (89·2 to 136)	429·0% (348·8 to 553·8)	1831·6 (1487·5 to 2246·3)	107·8% (75·9 to 157·6)
**North Africa and Middle East**		**6650 (5330 to 8120)**	**348·3% (296·1 to 389·1)**	**1338·3 (1087·5 to 1632·2)**	**67·5% (48·0 to 82·5)**
North Africa and Middle East		6650 (5330 to 8120)	348·3% (296·1 to 389·1)	1338·3 (1087·5 to 1632·2)	67·5% (48·0 to 82·5)
	Afghanistan	366 (282 to 462)	327·2% (250·3 to 393·8)	2099·1 (1634·4 to 2633·2)	93·7% (59·7 to 122·1)
	Algeria	437 (320 to 545)	481·2% (417·5 to 544·4)	1148·4 (855·6 to 1413·8)	101·2% (80·0 to 121·4)
	Bahrain	37·5 (30·6 to 45·2)	693·4% (565·3 to 819·2)	3125·4 (2614·7 to 3660·0)	21·9% (4·0 to 42·8)
	Egypt	1220 (993 to 1440)	386·2% (306·5 to 462·3)	1713·4 (1406·1 to 2009·2)	122·4% (86·5 to 156·0)
	Iran	780 (631 to 947)	426·2% (363·8 to 464·6)	961·3 (786·6 to 1158·7)	82·1% (58·8 to 95·7)
	Iraq	608 (453 to 754)	369·3% (286·7 to 449·5)	2193·8 (1688·9 to 2691·6)	45·9% (21·7 to 70·0)
	Jordan	148 (117 to 184)	491·2% (391·1 to 606·2)	1792·3 (1459·9 to 2220·5)	1·6% (−16·3 to 22·0)
	Kuwait	62·2 (46·2 to 80·4)	682·3% (600·7 to 758·0)	1666·7 (1268·4 to 2159·2)	60·8% (44·7 to 77·9)
	Lebanon	80·8 (64·1 to 101)	177·6% (132·1 to 219·9)	1481·5 (1174·1 to 1856·7)	18·5% (−0·5 to 37·6)
	Libya	84·9 (67·4 to 108)	560·4% (464·1 to 655·3)	1392·5 (1122·5 to 1767·4)	123·5% (88·7 to 154·7)
	Morocco	559 (438 to 693)	451·5% (389·5 to 516·7)	1592·8 (1247·8 to 1970·3)	137·6% (110·1 to 161·2)
	Oman	39·6 (32·9 to 47·6)	302·2% (205·7 to 377·3)	1656·7 (1410·7 to 1957·2)	30·7% (−1·7 to 57·6)
	Palestine	47·1 (40·0 to 55·8)	278·8% (210·9 to 343·0)	1782·5 (1530·8 to 2084·5)	29·4% (5·3 to 51·9)
	Qatar	34·2 (26·3 to 44·2)	1235·6% (946·6 to 1505·7)	2217·1 (1780·0 to 2815·6)	6·4% (−18·6 to 27·2)
	Saudi Arabia	391 (306 to 489)	541·8% (375·2 to 679·2)	1456·8 (1179·8 to 1781·1)	64·3% (21·1 to 98·2)
	Sudan	225 (172 to 279)	281·2% (216·0 to 341·0)	989·8 (784·3 to 1227·1)	81·0% (52·5 to 106·7)
	Syria	147 (116 to 185)	239·1% (179·9 to 299·4)	1090·0 (864·6 to 1369·0)	48·9% (23·0 to 75·6)
	Tunisia	152 (114 to 192)	451·4% (379·7 to 525·9)	1111·2 (836·7 to 1392·4)	116·2% (86·2 to 145·3)
	Türkiye	1010 (833 to 1250)	184·1% (136·4 to 236·1)	1074·0 (888·7 to 1319·3)	10·8% (−7·9 to 31·1)
	United Arab Emirates	82·2 (62·2 to 107)	1161·8% (863·5 to 1360·7)	1486·3 (1176·6 to 1815·6)	10·8% (−15·9 to 31·8)
	Yemen	132 (101 to 173)	337·6% (264·5 to 424·4)	800·4 (616·8 to 1059·2)	59·1% (31·5 to 89·3)
**South Asia**		**18 000 (15 500 to 20 500)**	**267·0% (230·1 to 299·6)**	**1153·4 (999·6 to 1306·6)**	**44·6% (30·0 to 58·2)**
South Asia		18 000 (15 500 to 20 500)	267·0% (230·1 to 299·6)	1153·4 (999·6 to 1306·6)	44·6% (30·0 to 58·2)
	Bangladesh	1650 (1350 to 2060)	282·6% (226·1 to 342·0)	1148·7 (939·1 to 1425·3)	37·8% (18·1 to 61·4)
	Bhutan	6·50 (5·16 to 7·83)	210·3% (152·9 to 284·2)	1061·5 (851·2 to 1278·8)	41·6% (17·3 to 75·5)
	India	13 900 (11 900 to 15 800)	262·9% (225·9 to 304·6)	1106·2 (952·1 to 1250·3)	44·0% (28·6 to 61·8)
	Nepal	304 (245 to 377)	285·3% (224·2 to 373·6)	1240·2 (1009·1 to 1516·9)	63·5% (37·0 to 102·0)
	Pakistan	2070 (1650 to 2420)	283·3% (232·6 to 347·4)	1604·6 (1301·4 to 1859·8)	80·1% (57·6 to 110·9)
**Southeast Asia, east Asia, and Oceania**		**20 800 (17 600 to 24 300)**	**187·7% (165·6 to 206·9)**	**735·6 (621·3 to 861·9)**	**25·3% (15·2 to 33·8)**
East Asia		12 400 (9900 to 15 000)	171·1% (144·8 to 190·5)	592·5 (472·1 to 720·7)	24·0% (9·3 to 35·3)
	China	11 700 (9310 to 14 200)	172·9% (145·5 to 194·1)	581·5 (460·5 to 707·6)	25·2% (9·7 to 37·0)
	North Korea	257 (205 to 326)	176·8% (127·3 to 229·0)	764·9 (611·9 to 962·9)	41·4% (16·1 to 66·3)
	Taiwan (province of China)	406 (337 to 489)	124·7% (104·9 to 143·5)	1002·2 (832·0 to 1210·2)	−8·4% (−17·1 to −0·1)
Oceania		308 (269 to 355)	213·9% (151·7 to 270·2)	3577·0 (3157·0 to 4120·5)	22·5% (−1·4 to 43·4)
	American Samoa	2·24 (1·92 to 2·62)	208·3% (159·3 to 263·7)	4307·8 (3692·1 to 4989·9)	49·8% (26·4 to 75·9)
	Cook Islands	1·03 (0·868 to 1·18)	91·1% (56·2 to 120·5)	4029·3 (3361·3 to 4643·7)	−2·0% (−19·6 to 12·9)
	Federated States of Micronesia	3·19 (2·55 to 3·85)	138·3% (93·8 to 194·0)	3933·7 (3207·9 to 4681·1)	50·1% (22·6 to 85·2)
	Fiji	59·9 (49·1 to 72·5)	182·8% (118·8 to 252·7)	7333·9 (6066·7 to 8776·7)	38·5% (7·5 to 71·5)
	Guam	2·54 (2·06 to 3·03)	137·3% (110·1 to 163·5)	1289·1 (1045·8 to 1545·5)	0·3% (−12·3 to 11·4)
	Kiribati	4·42 (3·61 to 5·50)	166·2% (112·0 to 238·9)	5510·6 (4508·6 to 6709·3)	36·3% (6·5 to 69·5)
	Marshall Islands	2·45 (1·89 to 3·17)	293·0% (206·7 to 367·4)	5750·8 (4384·5 to 7411·2)	69·8% (32·2 to 101·0)
	Nauru	0·272 (0·220 to 0·341)	68·7% (35·2 to 117·5)	4870·4 (4039·5 to 5855·2)	38·6% (12·5 to 78·5)
	Niue	0·0887 (0·0723 to 0·105)	64·6% (29·8 to 92·5)	4095·0 (3321·0 to 4823·2)	62·3% (27·9 to 89·6)
	Northern Mariana Islands	1·38 (1·17 to 1·70)	228·5% (159·0 to 283·2)	2199·4 (1871·3 to 2680·6)	14·1% (−8·5 to 34·0)
	Palau	0·891 (0·763 to 1·08)	227·0% (169·0 to 309·2)	3726·9 (3210·3 to 4536·0)	43·4% (19·2 to 78·6)
	Papua New Guinea	187 (157 to 225)	239·8% (143·5 to 337·0)	3062·0 (2597·2 to 3685·0)	18·6% (−14·6 to 52·3)
	Samoa	5·48 (4·61 to 6·57)	152·3% (109·8 to 200·3)	3390·8 (2876·6 to 4052·4)	44·0% (20·0 to 70·2)
	Solomon Islands	13·6 (11·0 to 16·9)	268·7% (154·5 to 421·5)	3473·1 (2878·9 to 4232·1)	46·6% (2·6 to 97·3)
	Tokelau	0·0503 (0·0410 to 0·0600)	49·1% (25·5 to 79·2)	3345·0 (2747·6 to 3948·8)	31·9% (11·1 to 56·3)
	Tonga	2·99 (2·44 to 3·46)	90·0% (55·1 to 130·2)	3640·0 (2966·9 to 4203·8)	35·5% (10·8 to 63·6)
	Tuvalu	0·357 (0·300 to 0·422)	93·5% (65·7 to 128·5)	3259·8 (2755·1 to 3847·4)	27·5% (9·7 to 49·6)
	Vanuatu	6·01 (5·05 to 6·97)	301·3% (216·9 to 400·6)	3006·9 (2550·6 to 3504·3)	45·7% (16·0 to 80·1)
Southeast Asia		8100 (7220 to 9290)	216·6% (185·8 to 244·3)	1220·7 (1084·5 to 1393·3)	31·8% (19·3 to 43·3)
	Cambodia	160 (130 to 199)	252·3% (173·7 to 337·9)	1205·1 (982·9 to 1497·6)	35·1% (5·7 to 66·9)
	Indonesia	2570 (2190 to 2960)	224·0% (181·4 to 258·5)	1067·0 (913·7 to 1215·4)	48·7% (29·7 to 64·4)
	Laos	69·4 (56·8 to 86·4)	159·1% (105·5 to 232·1)	1399·1 (1145·8 to 1723·4)	20·1% (−3·5 to 52·0)
	Malaysia	318 (258 to 382)	224·1% (186·5 to 257·5)	1073·7 (879·7 to 1284·2)	7·3% (−5·2 to 19·7)
	Maldives	3·22 (2·67 to 3·91)	188·6% (135·5 to 235·2)	867·6 (730·0 to 1043·3)	−21·9% (−35·1 to −9·8)
	Mauritius	65·1 (59·2 to 72·6)	340·4% (317·6 to 360·7)	3480·5 (3163·3 to 3879·7)	87·5% (78·0 to 96·5)
	Myanmar	1000 (832 to 1200)	119·9% (66·5 to 179·8)	1996·5 (1650·3 to 2388·4)	11·0% (−14·7 to 40·8)
	Philippines	1190 (1080 to 1310)	268·2% (244·3 to 296·0)	1357·9 (1234·4 to 1488·3)	39·9% (31·5 to 50·7)
	Seychelles	1·82 (1·44 to 2·31)	347·5% (295·4 to 398·0)	1524·8 (1215·2 to 1928·2)	114·8% (91·9 to 138·8)
	Sri Lanka	529 (416 to 646)	301·7% (230·8 to 378·2)	1952·5 (1540·5 to 2378·8)	62·1% (34·1 to 91·5)
	Thailand	1070 (847 to 1290)	248·5% (187·6 to 317·9)	996·4 (790·8 to 1194·2)	23·8% (2·0 to 49·2)
	Timor-Leste	9·40 (7·69 to 11·5)	350·7% (255·7 to 461·0)	1051·4 (861·6 to 1284·4)	69·5% (35·9 to 110·2)
	Viet Nam	1090 (913 to 1300)	217·6% (154·4 to 281·3)	1118·3 (935·5 to 1329·5)	33·1% (6·4 to 58·8)
**Sub-Saharan Africa**		**7560 (6720 to 8730)**	**175·6% (145·4 to 200·4)**	**1387·6 (1247·6 to 1589·2)**	**21·4% (7·9 to 31·6)**
Central sub-Saharan Africa		1060 (887 to 1270)	195·0% (138·8 to 260·6)	1631·3 (1376·3 to 1914·5)	14·7% (−6·8 to 39·8)
	Angola	235 (191 to 292)	262·9% (175·0 to 356·7)	1650·6 (1379·4 to 2022·0)	15·8% (−11·8 to 45·6)
	Central African Republic	57·6 (45·8 to 71·2)	137·2% (97·2 to 194·9)	2120·9 (1666·3 to 2574·1)	16·4% (−3·3 to 40·9)
	Congo (Brazzaville)	61·8 (51·3 to 74·7)	194·8% (132·9 to 269·1)	1954·0 (1656·2 to 2322·6)	9·1% (−10·8 to 33·9)
	Democratic Republic of the Congo	670 (554 to 803)	184·1% (115·7 to 259·5)	1548·7 (1287·0 to 1836·5)	14·7% (−11·5 to 45·7)
	Equatorial Guinea	11·7 (9·02 to 15·7)	239·8% (153·6 to 347·4)	1903·0 (1507·2 to 2541·0)	21·1% (−6·2 to 57·5)
	Gabon	26·8 (21·2 to 34·4)	166·8% (115·3 to 242·9)	2245·1 (1805·7 to 2846·3)	31·1% (5·9 to 65·6)
Eastern sub-Saharan Africa		2390 (2170 to 2720)	112·9% (93·2 to 140·7)	1197·0 (1080·8 to 1351·0)	−5·4% (−14·4 to 6·3)
	Burundi	69·9 (54·8 to 96·1)	87·2% (49·5 to 135·8)	1248·1 (972·5 to 1719·7)	−10·7% (−29·9 to 15·4)
	Comoros	7·38 (5·70 to 9·02)	143·7% (79·3 to 203·7)	1369·6 (1049·7 to 1675·2)	10·9% (−18·4 to 39·6)
	Djibouti	9·15 (7·24 to 12·2)	445·9% (327·7 to 596·2)	1289·2 (1051·9 to 1683·3)	35·7% (5·1 to 71·2)
	Eritrea	54·0 (41·8 to 68·4)	218·7% (159·8 to 288·4)	1606·5 (1256·2 to 2033·8)	16·5% (−3·3 to 36·7)
	Ethiopia	573 (502 to 639)	31·3% (10·8 to 58·7)	1125·9 (989·3 to 1258·3)	−38·8% (−48·0 to −27·5)
	Kenya	254 (221 to 302)	284·1% (224·3 to 367·9)	987·6 (865·7 to 1167·0)	38·8% (16·7 to 67·8)
	Madagascar	144 (116 to 179)	159·9% (110·5 to 221·8)	1051·9 (849·7 to 1309·9)	16·4% (−4·9 to 44·4)
	Malawi	113 (92·0 to 135)	118·1% (80·9 to 162·7)	1284·3 (1038·3 to 1531·5)	12·7% (−5·6 to 35·9)
	Mozambique	204 (160 to 249)	175·0% (113·9 to 240·1)	1476·8 (1183·7 to 1765·7)	41·2% (11·5 to 73·7)
	Rwanda	79·1 (57·6 to 106)	54·4% (19·1 to 90·6)	1126·8 (801·4 to 1517·9)	−26·1% (−42·4 to −10·2)
	Somalia	142 (111 to 179)	225·8% (162·6 to 304·4)	1631·2 (1318·2 to 2027·8)	15·1% (−5·9 to 40·9)
	South Sudan	71·7 (57·2 to 95·4)	125·8% (72·3 to 209·8)	1553·8 (1246·4 to 2083·2)	33·3% (1·3 to 82·9)
	Tanzania	322 (270 to 382)	157·5% (115·3 to 210·6)	1090·2 (913·7 to 1291·3)	12·6% (−4·8 to 37·5)
	Uganda	214 (167 to 286)	185·4% (105·5 to 261·7)	1239·5 (959·8 to 1659·6)	21·4% (−11·9 to 53·1)
	Zambia	131 (105 to 162)	177·0% (110·8 to 250·6)	1499·3 (1194·8 to 1858·4)	10·5% (−13·5 to 36·6)
Southern sub-Saharan Africa		1290 (1190 to 1410)	260·9% (231·1 to 288·7)	2128·5 (1978·7 to 2333·3)	73·3% (59·6 to 86·1)
	Botswana	25·0 (21·4 to 28·9)	191·9% (119·0 to 273·4)	1690·0 (1443·9 to 1932·7)	16·8% (−10·8 to 49·3)
	Eswatini	20·8 (16·4 to 27·4)	244·5% (170·7 to 376·3)	3334·2 (2669·6 to 4350·2)	67·0% (32·3 to 129·0)
	Lesotho	36·2 (28·2 to 44·8)	205·6% (134·9 to 330·9)	2711·4 (2145·2 to 3314·6)	131·9% (80·9 to 220·4)
	Namibia	28·3 (22·2 to 35·6)	153·7% (99·6 to 217·1)	1901·2 (1501·7 to 2375·0)	27·3% (0·7 to 57·1)
	South Africa	1030 (937 to 1140)	271·1% (239·4 to 298·5)	2150·8 (1962·4 to 2367·3)	71·5% (57·6 to 84·8)
	Zimbabwe	145 (120 to 179)	258·5% (180·1 to 353·4)	1899·2 (1570·7 to 2329·5)	96·8% (55·1 to 145·3)
Western sub-Saharan Africa		2820 (2340 to 3340)	213·3% (166·3 to 259·3)	1245·7 (1058·9 to 1460·1)	33·8% (14·6 to 51·2)
	Benin	82·6 (67·5 to 101)	311·2% (234·0 to 382·9)	1344·0 (1096·9 to 1632·3)	53·0% (25·9 to 76·3)
	Burkina Faso	128 (105 to 157)	176·5% (111·4 to 239·5)	1119·2 (915·3 to 1359·2)	16·3% (−9·8 to 39·2)
	Cabo Verde	6·20 (4·80 to 7·34)	418·7% (360·6 to 489·5)	1316·5 (1029·0 to 1552·2)	160·2% (132·2 to 194·7)
	Cameroon	223 (168 to 285)	326·9% (242·2 to 462·6)	1532·2 (1181·5 to 1947·8)	44·5% (14·1 to 89·2)
	Chad	87·3 (70·0 to 109)	282·3% (217·7 to 353·9)	1227·8 (977·1 to 1518·7)	69·1% (39·6 to 101·6)
	Côte d'Ivoire	179 (145 to 219)	295·2% (214·4 to 377·1)	1366·9 (1127·7 to 1673·7)	45·6% (17·0 to 78·5)
	The Gambia	16·0 (13·1 to 20·2)	376·0% (282·7 to 471·2)	1407·0 (1130·9 to 1778·1)	73·5% (39·4 to 105·8)
	Ghana	283 (229 to 351)	380·0% (278·8 to 499·8)	1502·0 (1222·6 to 1838·6)	82·2% (43·4 to 129·8)
	Guinea	80·5 (65·3 to 98·3)	164·9% (110·2 to 232·3)	1267·2 (1043·5 to 1556·9)	50·7% (20·2 to 88·7)
	Guinea-Bissau	15·6 (13·0 to 19·0)	157·0% (111·1 to 218·8)	1747·0 (1463·8 to 2118·2)	36·7% (11·8 to 69·5)
	Liberia	36·3 (27·8 to 46·2)	228·6% (162·6 to 289·8)	1427·7 (1112·3 to 1804·9)	54·0% (24·9 to 86·3)
	Mali	176 (147 to 213)	241·1% (182·5 to 295·4)	1679·9 (1415·5 to 2038·6)	48·7% (24·1 to 71·6)
	Mauritania	24·8 (19·2 to 31·6)	170·0% (119·1 to 249·5)	1065·7 (830·4 to 1351·9)	25·5% (1·9 to 61·6)
	Niger	100 (79·4 to 131)	294·3% (224·2 to 375·0)	1015·3 (812·7 to 1335·0)	40·7% (18·9 to 68·3)
	Nigeria	1140 (917 to 1380)	154·5% (106·4 to 213·1)	1103·6 (914·0 to 1298·0)	16·0% (−6·0 to 40·9)
	São Tomé and Príncipe	1·17 (0·885 to 1·46)	201·2% (161·9 to 252·6)	911·6 (711·8 to 1136·5)	65·2% (48·7 to 89·5)
	Senegal	138 (113 to 167)	270·8% (212·3 to 337·6)	1618·7 (1325·9 to 1973·4)	59·3% (33·4 to 87·1)
	Sierra Leone	50·6 (41·3 to 63·4)	222·9% (164·9 to 291·0)	1151·4 (950·3 to 1419·0)	55·6% (29·9 to 93·5)
	Togo	47·9 (38·1 to 61·5)	343·6% (270·5 to 446·9)	1089·9 (870·3 to 1387·9)	51·7% (25·6 to 83·5)

Data in parentheses are 95% uncertainty intervals. Count data are presented to three significant figures, and percentages and rates are presented to 1 decimal place. GBD=Global Burden of Diseases, Injuries, and Risk Factors Study. DALY=disability-adjusted life-year.

The age-standardised DALY rate was more than 1000 per 100 000 in four GBD super-regions: Latin America and Caribbean, sub-Saharan Africa, north Africa and the Middle East, and south Asia. Regionally, the age-standardised DALY rate varied from a high of 3577·0 (95% UI 3157·0–4120·5) per 100 000 in Oceania to a low of 511·8 (402·0–648·3) per 100 000 in western Europe. At the country level, Fiji had the highest age-standardised DALY rate, at 7333·9 (6066·7–8776·7) per 100 000. In 47 (23·0%) countries and territories, age-standardised DALY rates were greater than 2000 per 100 000 (table).

### Type 2 diabetes risk factors

In 2021, 58·9 million (95% UI 44·2–73·9) DALYs or 76·5% (58·0–87·5) of DALYs due to type 2 diabetes were attributable to risk factors. Of the 16 risk factors we analysed, high BMI was the primary risk factor for type 2 diabetes worldwide, accounting for 52·2% (25·5–71·8) of global type 2 diabetes DALYs. Among the other risk factor groups, dietary risks combined accounted for 25·7% (8·6–40·7), environmental or occupational risks combined accounted for 19·6% (12·7–26·5), tobacco use accounted for 12·1% (4·5–20·9), low physical activity accounted for 7·4% (3·0–11·2), and alcohol use accounted for 1·8% (0·3–3·9) of type 2 diabetes DALYs.

High BMI contributed more than 60% of type 2 diabetes DALYs in three super-regions: north Africa and the Middle East; Latin America and Caribbean; and central Europe, eastern Europe, and central Asia. Among the 21 regions analysed, the proportion of type 2 diabetes DALYs due to high BMI ranged from 68·0% (95% UI 37·8–85·8) in north Africa and the Middle East to 39·5% (17·1–58·4) in south Asia. High BMI contributed more than 60% of DALYs in 11 other regions: central Latin America, central Asia, southern Latin America, eastern Europe, southern sub-Saharan Africa, high-income North America, Australasia, tropical Latin America, central Europe, Andean Latin America, and Oceania. In south Asia, high BMI contributed less than 40% of type 2 diabetes DALYs. High BMI contributed more than 50% of DALYs in 167 (81·9%) countries and territories.

**Figure 3:**
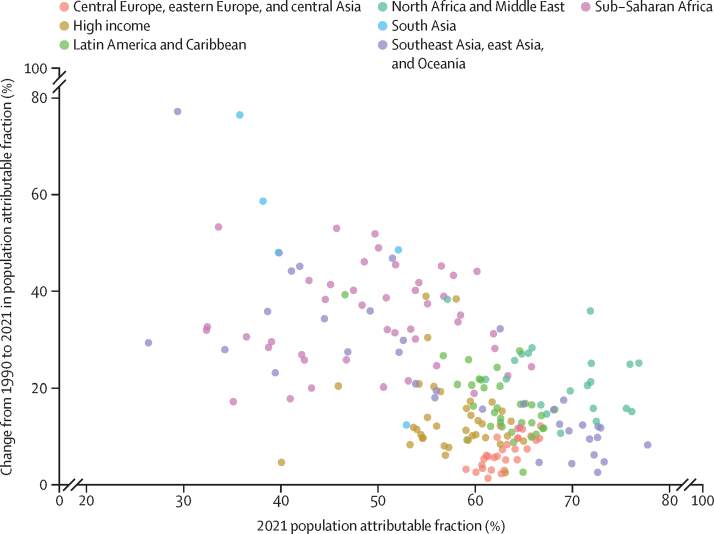
Change from 1990 to 2021 in population attributable fraction for high BMI in relation to type 2 diabetes, by GBD super-region BMI=body-mass index. GBD=Global Burden of Diseases, Injuries, and Risk Factors Study.

The proportion of global type 2 diabetes DALYs attributable to high BMI increased by 24·3% (95% UI 18·5 to 30·4), from 42·2% (19·8 to 59·9) in 1990 to 52·2% (25·5 to 71·8) in 2021. Although there were increases in every super-region, the largest change occurred in south Asia, with an increase of 58·0% (44·0 to 75·4). At the regional level, the increase in type 2 diabetes DALYs attributable to high BMI between 1990 and 2021 was greater than 45% in south Asia (58·0%; 44·0 to 75·4), central sub-Saharan Africa (48·8%; 35·8 to 61·2), and east Asia (45·7%; 33·5 to 57·3). Over this period, the proportion of DALYS due to high BMI increased in every country and territory, ranging from an increase of 77·2% (52·2 to 107·9) in Viet Nam to 1·3% (–1·5 to 4·1) in Czechia (figure 3).

### Diabetes prevalence over time: 1990 to 2021, and forecasts to 2050

Between 1990 and 2021, the global age-standardised prevalence of diabetes increased by 90·5% (95% UI 85·8–93·6), from 3·2% (3·0–3·5) to 6·1% (5·8–6·5; appendix table S22). This increase exceeded 100% in two super-regions: north Africa and the Middle East (161·5%; 154·3–168·7) and the high-income super-region (114·8%; 109·6–119·7). Six regions (north Africa and the Middle East, high-income North America, central Asia, Oceania, Andean Latin America, and southern Latin America) showed a prevalence increase of more than 100% from 1990 to 2021, while six additional regions (western Europe, southern sub-Saharan Africa, eastern Europe, south Asia, high-income Asia Pacific, and central sub-Saharan Africa) showed an increase of more than 90%. The age-standardised diabetes prevalence increased by more than 100% in 97 (47·5%) of 204 countries and territories and by more than 200% in three countries and one territory: Egypt (284·3%; 262·7–305·9), Greenland (263·6%; 236·8–296·3), Timor-Leste (225·3%; 206·7–243·7), and Seychelles (211·5%; 193·5–230·7). The age-standardised diabetes prevalence increased by less than 30% in only two countries: Mexico (19·7% [16·7–22·4]) and the Philippines (29·1% [24·3–33·8]; appendix table S22).

Between 2021 and 2050, the global age-standardised total diabetes prevalence is expected to increase by 59·7% (95% UI 54·7–66·0), from 6·1% (5·8–6·5) to 9·8% (9·4–10·2), resulting in 1·31 billion (1·22–1·39) people living with diabetes in 2050, or an annualised rate of change of 3·31%. Of this increase, 49·6% is driven by trends in obesity, and the remaining 50·4% is driven by demographic shifts. The age-standardised diabetes prevalence is projected to be higher than 10% in two super-regions: north Africa and the Middle East (16·8% [16·1–17·6]) and Latin America and Caribbean (11·3% [10·8–11·9]). The age-standardised diabetes prevalence rate is projected to exceed 10% in 89 (43·6%) countries and territories and to surpass 20% in 24 (11·8%) countries and territories. Every country and territory in three regions—Oceania, north Africa and the Middle East, and central Latin America—is projected to have a diabetes prevalence rate exceeding 10% by 2050. In 13 of 18 countries and territories in Oceania, ten of 21 countries in north Africa and the Middle East, and one country in the Caribbean, the diabetes prevalence will be greater than 20% by 2050. There are no countries and territories where diabetes prevalence rates are expected to decrease (appendix table S23).

**Figure 4:**
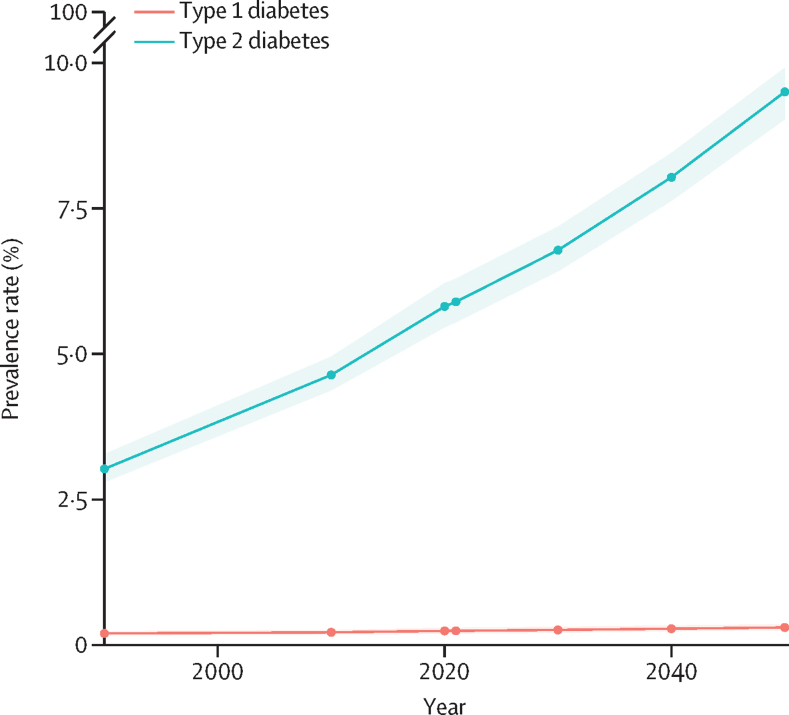
Global age-standardised prevalence of type 1 and type 2 diabetes from 1990 through 2050 forecasts The shaded area represents 95% uncertainty intervals. Total diabetes is the sum of type 1 and type 2 diabetes.

The projected increase in total diabetes prevalence is expected to be driven by type 2 diabetes. The age-standardised global prevalence of type 2 diabetes is projected to rise by 61·2% (95% UI 56·2–68·1), from 5·9% (5·5–6·3) in 2021 to 9·5% (9·0–9·9) in 2050, affecting more than 1·27 billion (1·19–1·35) people. This varies by super-region, from 82·7% (76·8–90·5) in north Africa and the Middle East to 30·3% (27·3–33·0) in the high-income region. Age-standardised type 2 diabetes prevalence will increase by more than 70% in six regions: north Africa and the Middle East (82·7%; 76·8–90·5), east Asia (80·1%; 72·1–89·2), central sub-Saharan Africa (79·9%; 72·4–89·9), southern sub-Saharan Africa (74·7%; 67·9–83·7), central Latin America (74·7%; 68·5–80·2), and Australasia (71·9%; 63·6–81·8). The age-standardised type 2 diabetes prevalence is projected to increase by more than 100% in 11 countries in three regions: seven countries (Oman, United Arab Emirates, Syria, Iran, Libya, Sudan, and Saudi Arabia) in north Africa and the Middle East, two countries (Kenya and Tanzania) in eastern sub-Saharan Africa, and two countries (Zimbabwe and Botswana) in southern sub-Saharan Africa. The age-standardised global prevalence of type 1 diabetes is expected to increase by 23·9% (95% UI 17·8–32·4), from 0·2% (0·2–0·3) in 2021 to 0·3% (0·3–0·4) in 2050 (figure 4; appendix figure S26).

## Discussion

The international community has become increasingly aware that diabetes is a monumental global health threat posing increasing challenges to public health and health-care systems worldwide. WHO has identified diabetes as one of three target diseases in its *WHO Global Action Plan for the Prevention and Control of NCDs,*^41^ and the WHO Global Diabetes Compact was established in 2021 to improve access to health care for, and work closely with, those living with diabetes. The UN uses diabetes treatment as an indicator of countries' health-care systems when assessing universal health coverage objectives^42^ and has established a target of reducing rates of premature death due to diabetes and other non-communicable diseases by a third by 2030, as detailed in Goal 3 of the UN Sustainable Development Goals.^43^ To drive improvements in diabetes prevention and care, the *Lancet* Commission on diabetes^2^ called for an increased reliance on high-quality data, with a focus on LMICs, to allow policy makers to better understand risks and define needs.

To contribute to this undertaking, as part of GBD we generated estimates of diabetes prevalence and burden, stratified by geographical and demographic factors; examined the contribution of leading risk factors; and forecast location-specific diabetes prevalence in 2050. There were an estimated 529 million people living with diabetes in 2021, a number we project will more than double to about 1·31 billion by 2050. The global age-standardised diabetes prevalence rate in 2021 was 6·1%, with highs of 9·3% in the north Africa and Middle East super-region and 12·3% in the Oceania region. Diabetes was especially prevalent in people aged 65 years and older in every location, but in some locations the prevalence rates were high even in younger adults, exceeding 10% among those aged 30–34 years in ten countries, all in Oceania.

Because diabetes prevalence rates are driven almost entirely by type 2 diabetes, which accounted for more than 96% of diabetes cases worldwide in 2021, much of the following discussion will focus on type 2 diabetes.

Our estimates showed high BMI to be the primary risk factor for type 2 diabetes, contributing more than 50% of global DALYs in 2021. The association between high BMI and type 2 diabetes has intensified in recent decades, with the proportion of global type 2 diabetes DALYs attributable to high BMI growing by nearly 25% between 1990 and 2021. By 2050, we project a global increase in age-standardised type 2 diabetes prevalence of more than 60%, with increases of more than 70% in six regions: north Africa and the Middle East, east Asia, central sub-Saharan Africa, southern sub-Saharan Africa, central Latin America, and Australasia.

Major behavioural shifts and changes in food systems contributing to high BMI include greater availability of shelf-stable and high-calorie products; limited financial and proximal access to healthy food options; increased consumption of ultra-processed foods^44^ and fat, sugar, and animal products; and reductions in physical activity related to global work and transportation trends.^45^ Particularly in low-income and middle-income populations, the shift away from a traditional diet to an industrialised one has been abrupt and is associated with considerable increases in nutrition-related non-communicable diseases such as type 2 diabetes.^46^ In some instances, high type 2 diabetes prevalence rates might also be partly associated with a population-specific genetic disposition to developing diabetes.^47^ A high diabetes burden in LMICs is also related to economic and sociopolitical challenges, including limited health spending on diabetes^10^ and inadequate or incomplete coverage for pharmacological treatment. Fewer than one in ten people with diabetes in LMICs receive coverage for comprehensive diabetes treatment; Oceania, for example, has the lowest medication coverage in the world despite its very high prevalence rates.^16^

The regional variation in sex differences in age-standardised diabetes prevalence rates revealed by our estimates is probably also related to variation in patterns in and the impact of obesity on type 2 diabetes, socioeconomic factors, and biological and hormonal differences.^48^ Evidence suggests that males might develop type 2 diabetes at lower BMI thresholds and might be more insulin resistant than females.^49^ Moreover, in a study of diabetes treatment coverage in LMICs, females had better treatment coverage than males.^16^ Conversely, obesity tends to be more common in females,^48^ and diabetes treatments rarely account for reported differences in the risk of developing diabetes at different ages between males and females, which are likely to be due to the impact of a combination of genetic, hormonal, and psychosocial dimensions.^50^

Although obesity is theoretically reversible^28^, ^51^, ^52^ and addressing it could provide the biggest opportunity to limit the advance of diabetes, current trends suggest that obesity rates are likely to continue to climb.^45^ Various interventions and policies to address obesity have been developed and studied,^53^, ^54^, ^55^, ^56^ but no programme to date has shown long-term, sustained, population-level reductions in obesity.^57^ This is probably because no strategy has attempted to deal with the multiple factors that potentially contribute to obesity. Creating change that relies on behavioural and structural shifts in interconnected, complex, and dynamic systems requires a multifaceted, long-term approach with contributions from policy makers, regulators, educators, public health officials, and the medical community.^58^ This is clearly not a simple challenge.

In 2022, the WHO Global Diabetes Compact outlined five diabetes targets to reach by 2030,^59^ focused on addressing metabolic risks, access to medication, and diagnosis. Although 77 countries, representing every region and socioeconomic level, have created recommendations, guidelines, and targets to monitor and control diabetes in their populations,^60^, ^61^, ^62^ preparedness varies considerably between countries. A survey of 160 WHO member states revealed that approximately 60% have conducted national surveys of blood glucose concentrations, 50% have a diabetes registry, and 80% have an action plan in place.^63^ Ultimately, effective testing, diagnosis, treatment, and diabetes control are lacking, particularly in LMICs.^17^ As our forecasts suggest that nearly 50% of the increases in diabetes prevalence will be due to changing demographic profiles, countries will need to invest in health systems to handle the surge in expected patients.

The outlook for a healthy future is further marred by the lack of sustained progress in strategies designed to remediate diabetes.^64^ Interventions that have yielded successful results for more than 2 years in people with type 2 diabetes involve bodyweight loss through aggressive control of calorie intake and physical activity or bariatric surgery.^65^, ^66^, ^67^, ^68^ Both options involve close oversight and are unlikely to be scalable at a population level globally. Pharmacological agents such as SGLT2 inhibitors and GLP1 agonists have shown some promising results in weight reduction and cardiovascular protection in individuals with type 2 diabetes,^69^, ^70^ but the viability of these interventions at the population level remains unclear. Moreover, disparities in medication coverage remain widespread.^2^, ^16^ Early diagnosis, patient education, and regular visits to health-care providers can offer clinicians, public health professionals, and policy makers opportunities for potentially effective early intervention through pharmacological approaches and other strategies such as lifestyle changes.^71^, ^72^ Evidence from studies done in China, Finland, and the USA suggests that these interventions can prevent or at least delay the onset of type 2 diabetes.^73^, ^74^, ^75^ Few countries have health-care systems, however, that are positioned to take a proactive approach or possess the infrastructure to prioritise early interventions. Developing and implementing strategies that will have long-lasting impacts at the population level remains a persistent challenge.

Although this study does not explicitly report the impact of diabetes on diseases such as chronic kidney disease, ischaemic heart disease, and cancer, since these relationships are captured in the GBD risk factor framework via high fasting plasma glucose, the impact of diabetes extends beyond the results presented here. Strategies and policies aimed at mitigating the diabetes burden should also consider the additional nuance that diabetes can lead to irreversible microvascular damage and increase the risk of morbidity and mortality due to other infectious and non-communicable diseases.^28^, ^51^, ^52^ Furthermore, efforts that succeed in halting the rise in diabetes could mitigate or delay associated health complications if implemented early.^76^, ^77^, ^78^, ^79^, ^80^ These are important considerations given that in many places in the world, increases in disability due to diabetes have outpaced diabetes mortality.^81^, ^82^

In addition to the estimates presented here, previous studies have reported global diabetes estimates from earlier rounds of GBD, and two other organisations, the IDF and NCD-RisC, have also generated global and multi-country estimates of diabetes for specific age groups.^4^, ^11^ Differences between estimates produced by GBD and IDF or NCD-RisC are likely to be due to differences in methods, case inclusion criteria, and data sources used. For example, our GBD analysis deliberately excluded sources that rely on self-reported diabetes data because we assumed that reporting bias would change over time and across location (eg, due to variability in diagnostic and screening efficacy), thus making bias adjustment challenging. Although we omitted these data sources, which were included in the IDF and NCD-RisC models, we were still able to incorporate data expressly gathered in 172 countries, exceeding the 144 locations with data in the IDF analysis and 146 locations with data in the NCD-RisC analysis. Moreover, our modelling approach—which used a Bayesian meta-regression tool, MR-BRT,^28^, ^33^ to develop coefficients to adjust non-reference case definitions, as well as DisMod-MR, which allows us to estimate prevalence by taking into account diabetes mortality—is unique to GBD. Other differences include our estimates for locations not reported by IDF (Cook Islands, Niue, and Tokelau) or NCD-RisC (Guam, Monaco, Northern Mariana Islands, San Marino, South Sudan, and Virgin Islands; appendix table S19). Our estimates covered the entire age spectrum, whereas IDF only reported total diabetes estimates for people aged 20–79 years and type 1 diabetes estimates for those younger than 19 years, and NCD-RisC reported estimates of total diabetes for individuals aged 18 years and older. Finally, we projected type-specific and total diabetes through 2050 for every age group, while IDF and NCD-RisC did not make projections as far out and did not generate forecasts for the entire population.

Despite methodological and reporting differences, our estimates of particularly high total diabetes prevalence rates in the north Africa and the Middle East super-region are supported by similar estimates reported in the 2021 IDF Atlas.^4^ Similar to many other regions, north Africa and the Middle East has experienced a rise in obesity due to rapid urbanisation and a concomitant rise in sedentary lifestyles and unhealthy eating patterns.^46^, ^83^

Our study has several strengths. The robust location-specific, age-specific, and sex-specific diabetes estimates produced by our analysis are largely due to our ability to leverage the rigorous evidentiary and methodological framework provided by the larger GBD enterprise. GBD integrates all available data, critically examines and standardises differences in methods, and draws upon the expertise of a network comprising more than 9000 researchers located in more than 160 countries to generate estimates of mortality and morbidity associated with 370 diseases and injuries worldwide. The GBD approach entails routinely updating systematic reviews of the peer-reviewed literature and seeking input from the global collaborator network to exhaustively identify studies that meet our inclusion criteria. We regularly present our results to and solicit feedback from in-country and topic experts to address data and methodological questions and concerns. Moreover, in keeping with established GBD methods, we report estimates of YLLs, YLDs, and DALYs that provide useful details about health loss associated with diabetes, in addition to estimating disease prevalence.

There are also limitations to our analysis. As discussed above, we deliberately excluded studies that identify people with diabetes by self-report status that is not validated by blood glucose tests; despite this, we were able to include data from 172 countries and territories, covering more than 80% of countries and territories reported in GBD and representing each of the 21 GBD regions. Additionally, our estimates and forecasts do not reflect a potential impact of the COVID-19 pandemic on diabetes prevalence and burden in 2020 and 2021. At the time of this analysis, these data were not available; the figures reported here are estimates of diabetes burden during a non-pandemic period. Data on the effects of the COVID-19 pandemic will be integrated into our models as they become available, allowing us to evaluate the impact of COVID-19 on the diabetes burden between 2020 and 2023, as well as on longer time trends.

Other limitations of our study include, first, that ICD coding practices documenting the underlying cause of death can vary. Studies that estimated the concordance between death certificates and patient records found disagreements with the reported underlying cause of death. These results vary by location and time but suggest that diabetes as a cause of death might be over-coded or under-coded depending on the location.^84^, ^85^, ^86^ Second, many deaths that are coded as being caused by diabetes do not specify the diabetes type. We sought to reallocate these deaths to either type 1 or type 2 diabetes using previously available information about type-specific distribution and deaths. However, this process relied on data from high-income and high-middle-income locations that might not have been valid for dissimilar locations. Third, although clinical diagnostic criteria for diabetes require more than one abnormal glucose concentration in the absence of symptoms,^1^ very few population-based epidemiological studies require these diagnostic criteria. Relying on a single glucose test could overestimate diabetes prevalence; however, the magnitude of error depends on the distribution of blood sugar concentrations as the reliability of a single glucose test increases with higher blood sugar values.^87^ Fourth, approximately half the data sources included in our analysis did not contain information on the method of blood collection, which can affect reported glucose concentrations by up to 20%.^88^, ^89^, ^90^ We plan to address this issue in upcoming GBD cycles. Fifth, our analysis did not estimate gestational diabetes explicitly since this diagnosis is captured in another GBD disease category, “Other maternal disorders”, nor did our study include rarer forms of diabetes such as monogenic diabetes,^1^ due to the paucity of relevant data. Sixth, GBD currently assumes that all individuals younger than 15 years have type 1 diabetes. Although there is growing evidence of type 2 diabetes occurring in younger individuals in many parts of the world,^91^, ^92^ we do not have population-based studies that distinguish type 1 diabetes from type 2 diabetes among people younger than 15 years across time, age, and location. We are monitoring the literature to determine the viability of addressing this concern. Seventh, GBD generates risk factor analyses for modifiable risk–outcome pairs. Each risk factor is identified by a review of the literature, and we regularly revisit the possibility of adding additional risk factors. The risk–outcome association is quantified through a rules-based assessment of existing evidence. Although our forecasts do not incorporate all known risk factors, here we capture the principal driver of type 2 diabetes, high BMI. In future rounds of GBD, we will revisit this strategy and consider adding additional risk factor covariates, such as low physical activity and smoking, to the model. Under the GBD's comparative risk assessment framework, we do not currently capture risk factors that have been reported in the literature for type 1 diabetes.^93^, ^94^ However, we believe that SDI is a good proxy for better access to care leading to a lower case fatality rate, and to increases in type 1 diabetes prevalence. Moreover, as with other autoimmune diseases, we found a positive correlation between economic development and the incidence of type 1 diabetes. Nevertheless, we acknowledge that additional research focused on risk factors for type 1 diabetes is needed. Finally, due to limitations inherent in the available literature and the comparative risk factor framework, we were unable to account for cohort effects apart from those pertaining to tobacco exposure.

The granular location-specific, age-specific, and sex-specific epidemiological diabetes data provided in the present analysis, along with our type-specific evaluation of diabetes risk factors and projections of diabetes prevalence in 2050—including which regions and countries or territories are likely to be most affected—are essential to policy makers, who must plan for an expansion of health-service capacity to manage diabetes cases and to maximise evidence-based prevention strategies. Our estimates should also serve as a rallying call to galvanise increased research funding to identify and develop more effective measures to prevent diabetes that are economically and behaviourally sustainable at a population level across the world.

Despite a well described understanding of the main drivers of diabetes, general consensus on what needs to change to reduce the diabetes burden, and widespread buy-in from international and national health organisations, diabetes prevalence continues to increase in every country and territory, age group, and in both males and females. Diabetes was already a substantial concern in 2021 and is set to become an even greater public health issue over the coming three decades, with no effective mitigation strategy currently in place. We need to urgently identify solutions that will limit population increases in risk factors for diabetes, otherwise the advance of the disease is likely to continue unabated. At the same time, we must enhance and expand access to better diabetes care to limit the complications associated with the disease. Differences between type 1 and type 2 diabetes with respect to risk factor profiles and underlying pathophysiology highlight the necessity to report type-specific diabetes both separately and together, given that hyperglycaemia leads to similar complications for both types. Although type 2 diabetes can in some cases be prevented and management of hyperglycaemia has the potential to improve outcomes, the disease continues to be a major public health problem due to high rates of obesity that place an increasing burden on individuals and health-care systems alike.

## Data sharing

Citations for the data used in these analyses are provided in the appendix (tables S25 and S26), with further information available on the Global Health Data Exchange website.

## Declaration of interests

J Ärnlöv reports payment or honoraria for lectures, presentations, speakers bureaus, manuscript writing, or educational events from AstraZeneca and Novartis; and participation on a Data Safety Monitoring Board or Advisory Board with AstraZeneca, Astella, and Boehringer Ingelheim; all outside the submitted work. S Bhaskar reports leadership or fiduciary role in other board, society, committee or advocacy group, paid or unpaid, with Rotary Club of Sydney, Australia as Board Director, with Rotary District 9675, Australia as Chair of Diversity Equity & Inclusion, and with Global Health & Migration, Global Health Hub Germany as Founding Member and Chair; all outside the submitted work. E J Boyko reports grants or contracts from the U.S. Department of Veteran Affairs; payment or honoraria for lectures, presentations, speakers bureaus, manuscript writing, or educational events from the Korean Diabetes Association, The Diabetes Association of the ROC (Taiwan; province of China), and the American Diabetes Association; support for attending meetings and/or travel from the Korean Diabetes Association, The Diabetes Association of the R.O.C. (Taiwan; province of China), and the International Society for the Diabetic Foot; all outside the submitted work. R M Islam reports support for attending meetings and/or travel from Lawley Pharmaceuticals for conference attendance outside the submitted work. N E Ismail reports unpaid leadership or fiduciary roles in board, society, committee, or advocacy groups with the Malaysian Academy of Pharmacy as Council Member and Bursar outside the submitted work. K Krishan reports non-financial support from UGC Centre of Advanced Study, CAS II, Department of Anthropology, Panjab University, Chandigarh, India, outside the submitted work. S Lorkowski reports grants or contracts paid to his institution from Akcea Therapeutics Germany; consulting fees from Danone, Novartis Pharma, Swedish Orphan Biovitrum (SOBI), and Upfield; payment or honoraria for lectures, presentations, speakers bureaus, manuscript writing, or educational events from Akcea Therapeutics Germany, AMARIN Germany, Amedes Holding, AMGEN, Berlin-Chemie, Boehringer Ingelheim Pharma, Daiichi Sankyo Deutschland, Danone, Hubert Burda Media Holding, Janssen-Cilag, Lilly Deutschland, Novartis Pharma, Novo Nordisk Pharma, Roche Pharma, Sanofi-Aventis, and SYNLAB Holding Deutschland & SYNLAB Akademie; support for attending meetings and/or travel from AMGEN; and participation on a data safety monitoring board or advisory board with Akcea Therapeutics Germany, AMGEN, Daiichi Sankyo Deutschland, Novartis Pharma, and Sanofi-Aventis; all outside the submitted work. A Ortiz has received grants from Sanofi; consultancy or speaker fees or travel support from Advicciene, Astellas, Astrazeneca, Amicus, Amgen, Boehringer Ingelheim, Fresenius Medical Care, GSK, Bayer, Sanofi-Genzyme, Menarini, Mundipharma, Kyowa Kirin, Lilly, Alexion, Freeline, Idorsia, Chiesi, Otsuka, Novo-Nordisk, Sysmex, and Vifor Fresenius Medical Care Renal Pharma and is Director of the Catedra Mundipharma-UAM of diabetic kidney disease and the Catedra Astrazeneca-UAM of chronic kidney disease and electrolytes; leadership or fiduciary roles in board, society, committee, or advocacy groups, paid or unpaid with the European Renal Association; and stock or stock options from Telara Farma; all outside the submitted work. V C F Pepito reports grants or contracts from Sanofi Consumer Healthcare to do research on self-care in the Philippines and from International Initiative for Impact Evaluation (3ie) to propose research on primary care benefit packages in the Philippines; all outside the submitted work. M J Postma reports stock or stock options from Health-Ecore, Zeist (NL) (25%) and PAG BV, Groningen (NL) (100%) outside the submitted work. D P Rasali reports an unpaid leadership or fiduciary role in a board, society, committee, or advocacy group with Emotional Well Being Institute Canada as Director. L R Reyes reports grants or contracts from Merck and Pfizer; consulting fees, payment or honoraria for lectures, presentations, speakers' bureaus, manuscript writing or educational events, and payments for expert testimony from Merck, Pfizer and GSK; support for attending meetings and/or travel from GSK; and participation on a data safety monitoring board or advisory board with Merck; all outside the submitted work. M P Schlaich reports consulting fees, payment, or honoraria for lectures, presentations, speakers' bureaus, manuscript writing or educational events, paid to himself, and support for attending meetings and/or travel paid to his institution, from Medtronic and Abbot; and leadership or fiduciary roles in board, society, committee, or advocacy groups, paid or unpaid with the World Hypertension League as Director; all outside the submitted work. C R Simpson reports research grants paid to his institution from Ministry of Business, Innovation and Employment (MBIE) (New Zealand), Health Research Council of New Zealand, Ministry of Health (New Zealand), Medical Research Council (UK), Health Data Research UK, and Chief Scientist Office (UK); all outside the submitted work. J A Singh reports consulting fees from Crealta/Horizon, Medisys, Fidia, PK Med, Two Labs, Adept Field Solutions, Clinical Care Options, Clearview Healthcare Partners, Putnam Associates, Focus Forward, Navigant cCnsulting, Spherix, MedIQ, Jupiter Life Science, UBM LLC, Trio Health, Medscape, WebMD, Practice Point communications, the National Institutes of Health and the American College of Rheumatology; payment or honoraria for lectures, presentations, speakers bureaus, manuscript writing, or educational events from the speaker's bureau of Simply Speaking; support for attending meetings and/or travel from the steering committee of OMERACT; participation on a Data Safety Monitoring Board or Advisory Board as a member of the FDA Arthritis Advisory Committee; leadership or fiduciary roles in board, society, committee, or advocacy groups, paid or unpaid with OMERACT as a steering committee member, with Veterans Affairs of Rheumatology Field Advisory Committee as a chair, and with UAB Cochrane Musculoskeletal Group Satellite Center on Network Meta-analysis as the editor and director; and stock or stock options in TPT Global Tech, Vaxart pharmaceuticals, Atyu biopharma, Adaptimmune Therapeutics, GeoVax Labs, Pieris Pharmaceuticals, Enzolytics, Seres Therapeutics, Tonix Pharmaceuticals and Charlotte's Web Holdings, with previously owned stock options in Amarin, Viking, and Moderna pharmaceuticals; all outside the submitted work. J Sundström reports stock or stock options from Anagram kommunikation AB and Symptoms Europe AB, outside the submitted work. D Trico reports payment or honoraria for lectures, presentations, speakers' bureaus, manuscript writing, or educational events and support for attending meetings and/or travel from AstraZeneca, Eli Lilly, and Novo Nordisk; participation on a data safety monitoring board or advisory board with Amarin; and receipt of equipment, materials, drugs, medical writing, gifts or other services to their institution from PharmaNutra and Abbott; all outside the submitted work. M Zielińska reports other financial or non-financial interests as an AstraZeneca employee outside the submitted work. A Z reports other financial or non-financial interests in the Pan African Network for Rapid Research, Response, and Preparedness for Infectious Diseases Epidemics Consortium (PANDORA-ID-NET), European and Developing Countries Clinical Trials Partnership the EU Horizon 2020 Framework Programme (EDCTP-RIA2016E-1609). Sir Zumla is a UK-NIHR Senior Investigator, and a Mahathir Science Award, Sir Patrick Manson Medal, and EU-EDCTP Pascoal Mocumbi Prize laureate; all outside the submitted work.
