
JOURNAL INFORMATION
==============================
NLM Title Abbreviation: Lancet
Journal ID: Lancet
NLM Journal ID: 2985213R

Lancet (London, England)

ISSN: 0140-6736
EISSN: 1474-547X

ARTICLE INFORMATION
==============================
PMCID: PMC11790340
PMID: 39826967
DOI: 10.1016/S0140-6736(25)00053-4
Article ID: S0140-6736(25)00053-4
Article version: 1
Subjects: Department of Error

Department of Error

Print publication date: 2025 Jan 18
Volume: 405
Issue: 10474
First page: 202
Last page: 202
Copyright: © 2025 The Author(s). Published by Elsevier Ltd.
Copyright year: 2025
Copyright holder: Elsevier Ltd
License: This is an open access article under the CC BY license (http://creativecommons.org/licenses/by/4.0/).
License URL: https://creativecommons.org/licenses/by/4.0/

Erratum for: Global, regional, and national burden of diabetes from 1990 to 2021, with projections of prevalence to 2050: a systematic analysis for the Global Burden of Disease Study 2021 402 10397 15 7 2023 203 234 Lancet (London, England) 10.1016/S0140-6736(23)01301-6 PMC10364581 37356446

==============================
GBD 2021 Diabetes Collaborators. Global, regional, and national burden of diabetes from 1990 to 2021, with projections of prevalence to 2050: a systematic analysis for the Global Burden of Disease Study 2021. Lancet 2023; 402: 203–34—In this Article, the map in figure 1 has been updated to correct boundary lines and shading in selected regions. This correction has been made to the online version as of Jan 16, 2025.

