# Supplementary material for: Global, regional, and national burden of diabetes from 1990 to 2021, with projections of prevalence to 2050: a systematic analysis for the Global Burden of Disease Study 2021
Source: Lancet. 2023 Jul 15;402(10397):203–34. doi: 10.1016/S0140-6736(23)01301-6 (PMC10364581; doi:10.1016/S0140-6736(23)01301-6)
Supplement: Supplementary appendix [file mmc1.pdf]

THE LANCET

Supplementary appendix

This appendix formed part of the original submission and has been peer reviewed. We post it as supplied by the authors.

Supplement to: GBD 2021 Diabetes Collaborators. Global, regional, and national burden of diabetes from 1990 to 2021, with projections of prevalence to 2050: a systematic analysis for the Global Burden of Disease Study 2021. *Lancet* 2023; published online June 22. https://doi.org/10.1016/S0140-6736(23)01301-6.

Appendix 1: Supplementary methods and results to "Global, regional, and national burden of diabetes from 1990 to 2021, with projections of prevalence to 2050: a systematic analysis for the Global Burden of Disease Study 2021"

This appendix provides supplemental figures and more detailed results for "Global, regional, and national burden of diabetes from 1990 to 2021, with projections of prevalence to 2050: a systematic analysis for the Global Burden of Disease Study 2021"

Portions of this appendix have been reproduced or adapted from GBD 2019 Diseases and Injuries Collaborators.[1] References are provided for reproduced sections.

Table of Contents

List of figures and tables 3
Figures 3
Tables 3
Section 1. Statement of GATHER compliance 5
Section 2. Flowchart[1] 6
Section 3. Fatal[1] 6
Section 3.1. Sources 6
Section 3.2 Data processing 7
Section 3.2.1. Case definitions 7
Section 3.2.2. Total diabetes 8
Section 3.2.3. Type 1 & type 2 diabetes 8
Section 3.3. Modelling strategies 10
Section 3.3.1. Total diabetes 10
Section 3.3.2. Type 1 & type 2 diabetes 13
Section 4. Nonfatal[1] 17
Section 4.1. Sources 17
Section 4.1.1. Total diabetes & type 1 diabetes 17
Section 4.1.2. Diabetic outcomes 21
Section 4.2 Data processing 27
Section 4.2.1. Case definitions 27
Section 4.2.2. Total diabetes 28
Section 4.2.3. Type 1 diabetes 30
Section 4.2.4. Diabetic outcomes 30
Section 4.3. Modelling strategies 31
Section 4.3.1. Total diabetes 31
Section 4.3.2. Type 1 diabetes 31
Section 4.3.3. Type 2 diabetes 32
Section 4.3.4. Diabetic outcomes 32
Section 4.4 Disability weights 33
Section 4.4.1. Diabetic outcomes 33
Section 5. Risk factors[1] 34
Section 5.1 Attributable risk methods 34

Section 5.1.1. High alcohol use 35
Section 5.1.2. Dietary risks 36
Section 5.1.3. Environmental/occupational risks 36
Section 5.1.4. Low physical activity 38
Section 5.1.5. Tobacco 38
Section 5.1.6. High body-mass index 39
Section 6. References 40
Section 7. Additional figures and tables 41
Figure S20. Total diabetes age-standardised prevalence for both sexes combined in 1990 (A) and 2050 (B) 41
Figure S21. Type 1 diabetes age-standardised prevalence for both sexes combined in 2021 (A) and 2050 (B) 43
Figure S22. Type 2 diabetes age-standardised prevalence for both sexes combined in 2021 (A) and 2050 (B) 45
Figure S23. Total diabetes age-specific prevalence by sex in 2021 by super-region, region, and country 47
Figure S24. Sex ratio (males-to-females) of age-standardised total diabetes prevalence in 204 locations by GBD region in 2021 ... 54
Figure S25. Change from 1990 to 2021 in population attributable fraction for five risk factor groups in relation to type 2 diabetes 54
Figure S26. Global number of people with type 1 diabetes and type 2 diabetes from 1990 through 2050 forecasts 55
Table S18. Global Burden of Disease location hierarchy 55
Table S19. Countries estimated by the Global Burden of Diseases, Injuries, and Risk Factors Study (GBD), the International Diabetes Federation (IDF)[11], and the NCD Risk Factor Collaboration (NCD-RisC)[12] 64
Table S20. Number of people with diabetes estimated by the Global Burden of Diseases, Injuries, and Risk Factors Study (GBD), the International Diabetes Federation (IDF)[11], and the NCD Risk Factor Collaboration (NCD-RisC)[12] 69
Table S21. YLLs and YLDs counts and age-standardised rates per 100,000 population and the corresponding percentage change in YLLs and YLDs counts and age-standardised rates between 1990 and 2021 for diabetes globally, in 21 Global Burden of Disease regions and all countries 69
Table S22. Prevalence counts and age-standardised rates per 100,000 population and the corresponding percentage change between 1990 and 2021 for diabetes globally, in 21 Global Burden of Disease regions and all countries 78
Table S23. Prevalence counts and age-standardised rates per 100,000 population and the corresponding percentage change between 2021 and 2050 for diabetes globally, in 21 Global Burden of Disease regions and all countries 87
Table S24. Death counts and age-standardised rates per 100,000 population and the corresponding percentage change between 1990 and 2021 for diabetes globally, in 21 Global Burden of Disease regions and all countries 95
Table S25. Total diabetes and type 1 diabetes nonfatal input data source citations 104
Table S26. Diabetes relative risk input data source citations 129
Section 8. Contributions 143
Managing the overall research enterprise 143
Writing the first draft of the manuscript 144
Primary responsibility for applying analytical methods to produce estimates 144
Primary responsibility for seeking, cataloguing, extracting, or cleaning data; designing or coding figures and tables 144
Providing data or critical feedback on data sources 144
Developing methods or computational machinery 145
Providing critical feedback on methods or results 145
Drafting the work or revising it critically for important intellectual content 147
Managing the estimation or publications process 148

List of figures and tables

Figures and tables are available throughout the text and in section 7.

Figures

Figure S1. Diabetes fatal and nonfatal flowchart
Figure S2. Sources used in fatal total diabetes estimation
Figure S3. Redistribution of ICD-10 garbage codes in both sexes and all ages, globally in 2015
Figure S4. Proportion of type-specific vs. unspecified type diabetes data by age, globally for both sexes in 2015
Figure S5. Redistribution of unspecified diabetes type data by age, globally among males in 2015
Figure S6. Redistribution of unspecified diabetes type data by age, globally among females in 2015
Figure S7. Covariate influence plot for fatal diabetes 0-14 years model
Figure S8. Covariate influence plot for fatal diabetes 15-95+ years model
Figure S9. Covariate influence plot for fatal type 1 diabetes model
Figure S10. Covariate influence plot for fatal type 2 diabetes model
Figure S11. PRISMA diagram of GBD 2019 nonfatal diabetes systematic review
Figure S12. Diagram of data source updates in the nonfatal total diabetes model since GBD 2019
Figure S13. Sources used in nonfatal total diabetes estimation
Figure S14. Sources used in nonfatal type 1 diabetes estimation
Figure S15. Sources used in nonfatal diabetic neuropathy estimation
Figure S16. Sources used in nonfatal diabetic foot estimation
Figure S17. Sources used in nonfatal amputation due to diabetes estimation
Figure S18. Sources used in nonfatal low vision due to diabetic retinopathy estimation
Figure S19. Sources used in nonfatal blindness due to diabetic retinopathy estimation
Figure S20: Total diabetes age-standardised prevalence for both sexes combined in 1990 (A) and 2050 (B)
Figure S21. Type 1 diabetes age-standardised prevalence for both sexes combined in 2021 (A) and 2050 (B)
Figure S22. Type 2 diabetes age-standardised prevalence for both sexes combined in 2021 (A) and 2050 (B)
Figure S23. Total diabetes age-specific prevalence by sex in 2021 by super-region, region, and country
Figure S24. Sex ratio (males-to-females) of age-standardised total diabetes prevalence in 204 locations by GBD region in 2021
Figure S25. Change from 1990 to 2021 in population attributable fraction for five risk factor groups in relation to type 2 diabetes
Figure S26. Global number of people with type 1 diabetes and type 2 diabetes from 1990 through 2050 forecasts

Tables

Table S1. GATHER checklist
Table S2. ICD-9 and ICD-10 fatal diabetes codes
Table S3. ICD-9 and ICD-10 fatal diabetes garbage codes coded to unspecified diabetes
Table S4. Covariates used in total diabetes fatal modelling
Table S5. Covariates used in type 1 diabetes and type 2 diabetes fatal modelling
Table S6. Case definitions for nonfatal diabetes and diabetic outcomes
Table S7. MR-BRT crosswalk adjustment factors for total diabetes
Table S8. MR-BRT crosswalk adjustment factors for type 1 diabetes
Table S9. MR-BRT crosswalk adjustment factors for low vision due to diabetes
Table S10. MR-BRT crosswalk adjustment factors for blindness due to diabetes
Table S11. Covariates used in total diabetes nonfatal modelling
Table S12. Covariates used in type 1 diabetes nonfatal modelling
Table S13. Covariates used in diabetic foot nonfatal modelling
Table S14. Covariates used in amputation due to diabetes nonfatal modelling
Table S15. Covariates used in vision impairment due to diabetes nonfatal modelling
Table S16. Severity levels and disability weights for diabetic outcomes
Table S17. Theoretical minimum risk exposure levels
Table S18. Global Burden of Disease location hierarchy
Table S19. Countries estimated by the Global Burden of Diseases, Injuries, and Risk Factors Study (GBD), the International Diabetes Federation (IDF), and the NCD Risk Factor Collaboration (NCD-RisC)
Table S20. Number of people with diabetes estimated by the Global Burden of Diseases, Injuries, and Risk Factors Study (GBD), the International Diabetes Federation (IDF), and the NCD Risk Factor Collaboration (NCD-RisC)
Table S21. YLLs and YLDs counts and age-standardised rates per 100,000 population and the corresponding percentage change in

YLLs and YLDs counts and age-standardised rates between 1990 and 2021 for diabetes globally, in 21 Global Burden of Disease regions and all countries
Table S22. Prevalence counts and age-standardised rates per 100,000 population and the corresponding percentage change between 1990 and 2021 for diabetes globally, in 21 Global Burden of Disease regions and all countries
Table S23. Prevalence counts and age-standardised rates per 100,000 population and the corresponding percentage change between 2021 and 2050 for diabetes globally, in 21 Global Burden of Disease regions and all countries
Table S24. Death counts and age-standardised rates per 100,000 population and the corresponding percentage change between 1990 and 2021 for diabetes globally, in 21 Global Burden of Disease regions and all countries
Table S25. Total diabetes and type 1 diabetes nonfatal input data source citations
Table S26. Diabetes relative risk input data source citations

Section 1. Statement of GATHER compliance

This study complies with the Guidelines for Accurate and Transparent Health Estimates Reporting (GATHER) recommendations. Below is the GATHER checklist.[2]

Table S1. GATHER checklist

#	GATHER checklist item	Description of compliance	Reference
**Objectives and funding**			
**1**	Define the indicator(s), populations (including age, sex, and geographic entities), and time period(s) for which estimates were made.	Narrative provided in paper and appendix describing indicators, definitions, populations, and time periods	Main text (Methods) and Appendix (Sections 3-4)
**2**	List the funding sources for the work.	Funding sources listed in paper	Summary (Funding)
**Data Inputs**			
For all data inputs from multiple sources that are synthesized as part of the study:			
**3**	Describe how the data were identified and how the data were accessed.	Narrative description of data seeking methods provided	Main text (Methods) and Appendix (Sections 3-4)
**4**	Specify the inclusion and exclusion criteria. Identify all ad-hoc exclusions.	Narrative about inclusion and exclusion criteria provided; ad hoc exclusions in appendix supplementary methods	Main text (Methods) and Appendix (Sections 3-4)
**5**	Provide information on all included data sources and their main characteristics. For each data source used, report reference information or contact name/institution, population represented, data collection method, year(s) of data collection, sex and age range, diagnostic criteria or measurement method, and sample size, as relevant.	An interactive, online data source tool that provides metadata for data sources by component, geography, cause, risk, or impairment has been developed, and data source citations provided	Appendix (Sections 3-4) and citations in Appendix (Section 7) with additional information about these sources available at https://ghdx.healthdata.org/
**6**	Identify and describe any categories of input data that have potentially important biases (e.g., based on characteristics listed in item 5).	Summary of known biases included in appendix supplementary methods	Appendix (Section 4)
For data inputs that contribute to the analysis but were not synthesized as part of the study:			
**7**	Describe and give sources for any other data inputs.	Included in online data source tool	Global Health Data Exchange (https://ghdx.healthdata.org/)
For all data inputs:			
**8**	Provide all data inputs in a file format from which data can be efficiently extracted (e.g., a spreadsheet rather than a PDF), including all relevant meta-data listed in item 5. For any data inputs that cannot be shared because of ethical or legal reasons, such as third-party ownership, provide a contact name or the name of the institution that retains the right to the data.	Downloads of input data available through online data tools; input data not available in tools will be made available upon request	Global Health Data Exchange (https://ghdx.healthdata.org/)
**Data analysis**			
**9**	Provide a conceptual overview of the data analysis method. A diagram may be helpful.	Flow diagram of methodological process provided, as well as narrative descriptions of modelling process	Main text (Methods) and Appendix (Sections 2-4)
**10**	Provide a detailed description of all steps of the analysis, including mathematical formulae. This description should cover, as relevant, data cleaning, data pre-processing, data adjustments and weighting of data sources, and mathematical or statistical model(s).	Flow diagram and detailed methods write-up covering all data extraction, processing, and modelling processes provided	Main text (Methods) and Appendix (Sections 2-4)
**11**	Describe how candidate models were evaluated and how the final model(s) were selected.	Provided in methodological write-up	Appendix (Sections 2-4)
**12**	Provide the results of an evaluation of model performance, if done, as well as the results of any relevant sensitivity analysis.	Provided in methodological write-up	Appendix (Sections 2-4)
**13**	Describe methods for calculating uncertainty of the estimates. State which sources of uncertainty were, and were not, accounted for in the uncertainty analysis.	Provided in main text methods narrative description and appendix methodological write-up	Main text (Methods) and Appendix (Section 4)
**14**	State how analytic or statistical source code used to generate estimates can be accessed.	Remote code repository for access to analytic code provided	Remote code repository
**Results and Discussion**			
**15**	Provide published estimates in a file format from which data can be efficiently extracted.	Published estimates not available in main text or appendix will be made available upon request.	Main text (Methods, Results and Discussion), Appendix (Section 7)
**16**	Report a quantitative measure of the uncertainty of the estimates (e.g. uncertainty intervals).	Uncertainty provided with all results	Main text (Methods), Appendix (Section 4)

**17**	Interpret results in light of existing evidence. If updating a previous set of estimates, describe the reasons for changes in estimates.	Discussion of results and methodological changes between GBD rounds provided in manuscript narrative and appendix	Main text (Methods, Results and Discussion) and Appendix (Sections 2-5)
**18**	Discuss limitations of the estimates. Include a discussion of any modelling assumptions or data limitations that affect interpretation of the estimates.	Discussion of limitations, including modelling assumptions and data limitations, included in manuscript narrative and appendix	Main text (Methods and Discussion) and Appendix (Sections 2-4)

Section 2. Flowchart[1]

Below is a flowchart of the Global Burden of Disease (GBD) diabetes fatal and nonfatal modelling process. Red numbers in shape corners indicate relevant appendix sections for processes.

Figure S1. Diabetes fatal and nonfatal flowchart

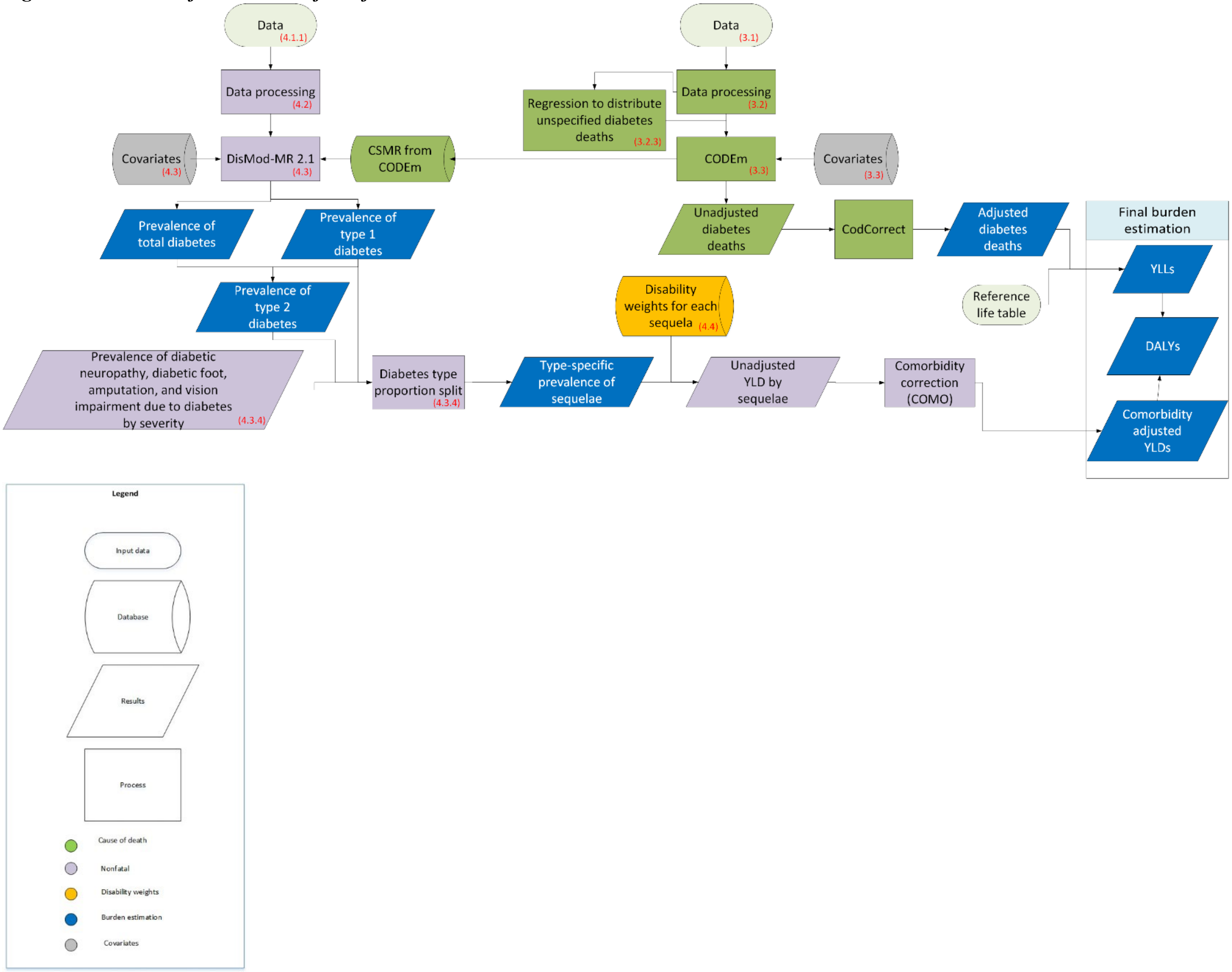

Section 3. Fatal[1]

Section 3.1. Sources

All source count maps below reflect subnational-level location-year counts for countries where GBD estimates at the subnational-level, and national-level location-year counts for countries where GBD does not estimate at the subnational-level. Location-year counts for sources representative at the national-level only for countries where GBD estimates at the subnational-level are not reflected in the maps.

*Figure S2. Sources used in fatal total diabetes estimation**

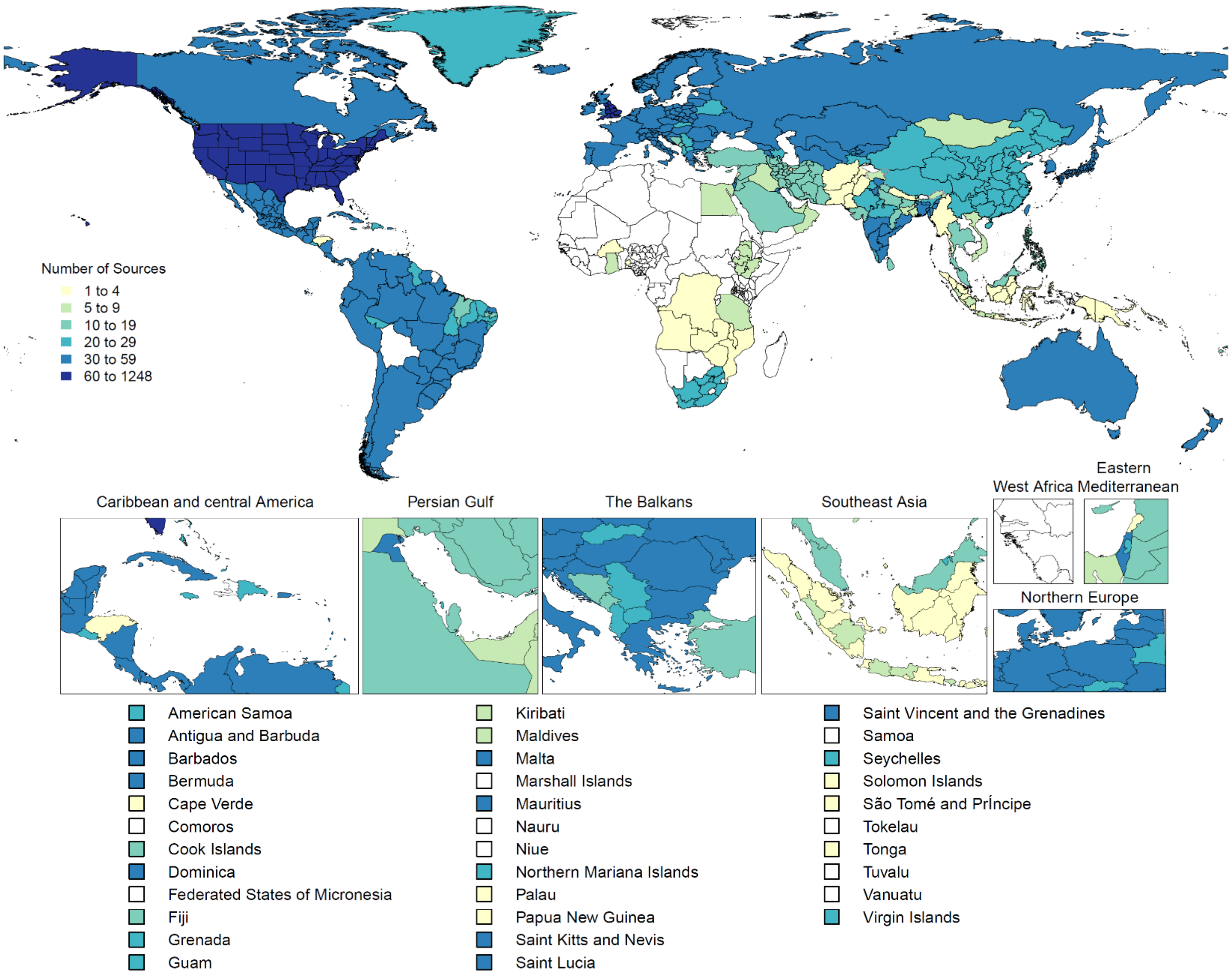

**Also representative of fatal type 1 diabetes and fatal type 2 diabetes estimation source location-years. Note that this map does not reflect subnational sources for Russia, which range from 15 to 32 location-years.*

Section 3.2 Data processing

Section 3.2.1. Case definitions

Below are the International Classification of Diseases (ICD)-9 and ICD-10 codes used in the diabetes fatal models.

Table S2. ICD-9 and ICD-10 fatal diabetes codes

	**ICD-9**	**ICD-10**
**Type 1 diabetes**	250.01, 250.03, 250.11, 250.13, 250.21, 250.23, 250.31, 250.33, 250.51, 250.53, 250.61, 250.63, 250.71, 250.73, 250.81, 250.83, 250.91, 250.93, 775.1	E10, P70.2
**Type 2 diabetes**	250.00, 250.02, 250.10, 250.12, 250.20, 250.22, 250.30, 250.32, 250.50, 250.52, 250.60, 250.62, 250.70, 250.72, 250.80, 250.82, 250.90, 250.92	E11
**Unspecified diabetes type**	250.0, 250.09, 250.1, 250.19, 250.2, 250.29, 250.3, 250.39, 250.4, 250.49, 250.5, 250.59, 250.6, 250.69, 250.7, 250.79, 250.8, 250.89, 250.9, 250.99, 357.2, 362.0, 362.01, 362.02, 362.03, 362.04, 362.05, 362.06, 790.2, 790.21, 790.22	E12, E13, E14, R73

ICD-9 and ICD-10 codes that are either intermediate causes of death where diabetes may be the underlying cause or do not lead directly to death are referred to as garbage codes and may be redistributed to the diabetes fatal model.

Figure S3. Redistribution of ICD-10 garbage codes in both sexes and all ages, globally in 2015

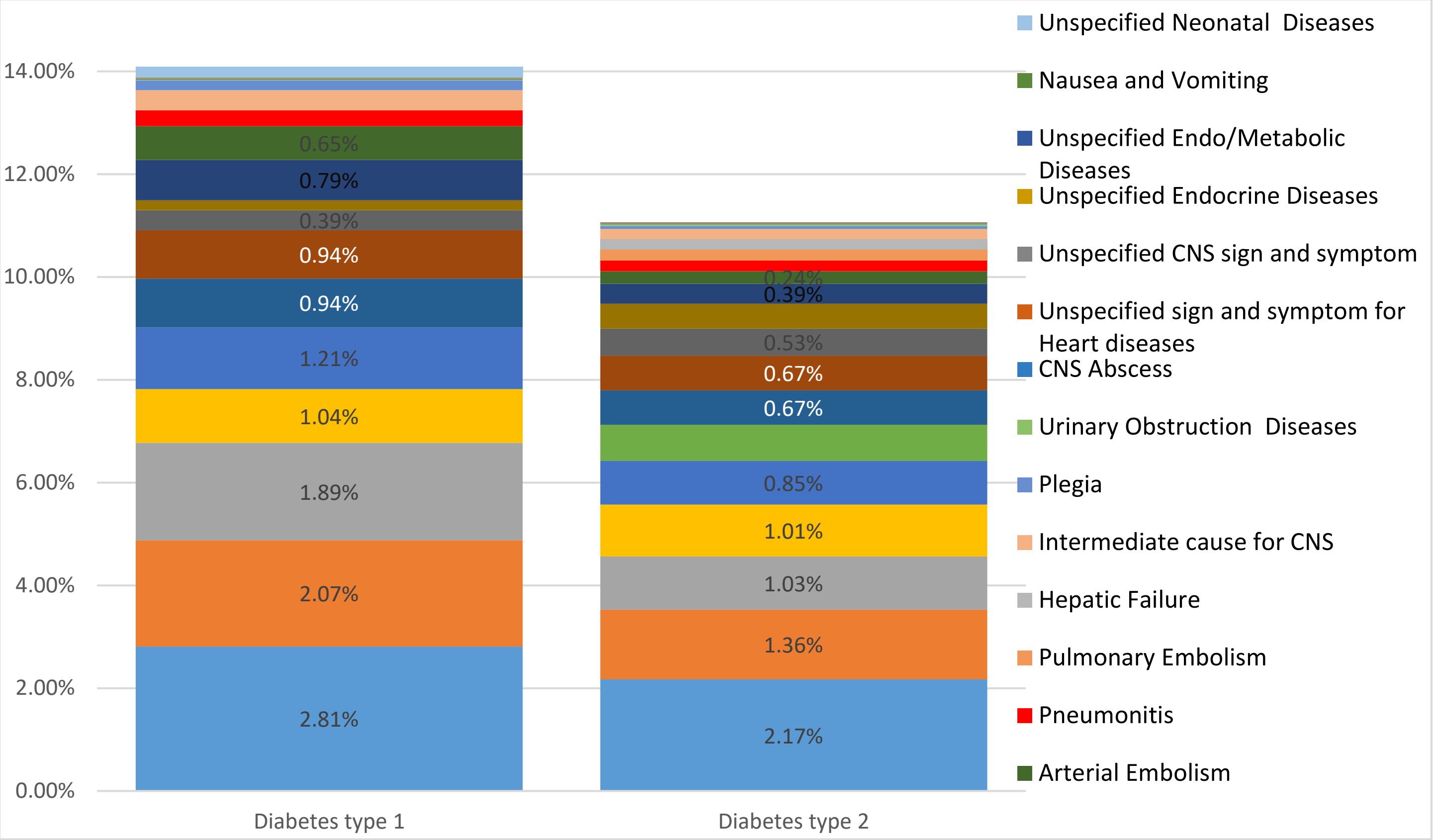

Table S3. ICD-9 and ICD-10 fatal diabetes garbage codes coded to unspecified diabetes

	**ICD-9**	**ICD-10**
**Unspecified diabetes type**	250.0, 250.09, 250.1, 250.19, 250.2, 250.29, 250.3, 250.39, 250.4, 250.49, 250.5, 250.59, 250.6, 250.69, 250.7, 250.79, 250.8, 250.89, 250.9, 250.99, 357.2, 362.0, 362.01, 362.02, 362.03, 362.04, 362.05, 362.06, 790.2, 790.21, 790.22	E12, E13, E14, R73

Section 3.2.2. Total diabetes

Verbal autopsy and vital registration data were used as inputs into the total diabetes model.

Verbal autopsy data: We outliered data points from sources where there were zero deaths estimated in an age group as this was not realistic for deaths due to diabetes and we determined that these data sources were unreliable.

Vital registration data: We outliered all data from the India Medical Certification of Cause of Death report since the source of the data was unreliable according to expert opinion. We also outliered ICD9BTL data points that were inconsistent with the rest of the data series and created unlikely time trends.

Section 3.2.3. Type 1 & type 2 diabetes

Type-specific diabetes mortality was estimated using deaths from vital registration data. There were two unique data manipulation steps that occurred in order to prepare the data as part of the modelling process.

3. We assumed that all deaths <15 years were due to type 1 regardless of the ICD code assigned to the death. We imposed 100% attribution of diabetes deaths in <15 years to type 1 diabetes.
4. ICD diabetes data were reported as type 1, type 2, or unspecified. We assumed that all deaths in persons >50 years was unspecified regardless of the ICD code assigned to the death because we found an unreasonably high proportion of deaths

due to diabetes were assigned to type 1 diabetes. We developed a regression to estimate the fraction of unspecified diabetes that was type 1 and type 2. We only used data from 703 country-years to inform the regression. This is because these country-years had more than 50% of the deaths typed to type 1 or type 2 AND at least 70% of type-specific deaths in people >25 years were coded to type 2. Since there was a separate regression to estimate the proportion of type 1 diabetes and type 2 diabetes, we scaled the predicted proportions to one. These scaled proportions were then applied to number of deaths coded to unspecified diabetes in each location, year, and sex where ICD data was reported.

Regression equations:

Type 1:

$$logit\left(\frac{number\ type\ 1\ DM}{number\ total\ DM}\right) \sim logit\left(\frac{number\ unspecified\ DM}{number\ total\ DM}\right) + \beta_1 age\ group + \beta_2 age\text{-}st\ prev\ obesity * age\ group + age\text{-}st\ prev\ obesity$$

Type 2:

$$logit\left(\frac{number\ type\ 2\ DM}{number\ total\ DM}\right) \sim logit\left(\frac{number\ unspecified\ DM}{number\ total\ DM}\right) + \beta_1 age\ group + \beta_2 age\text{-}st\ prev\ obesity * age\ group + age\text{-}st\ prev\ obesity$$

Figure S4. Proportion of type-specific vs. unspecified type diabetes data by age, globally for both sexes in 2015

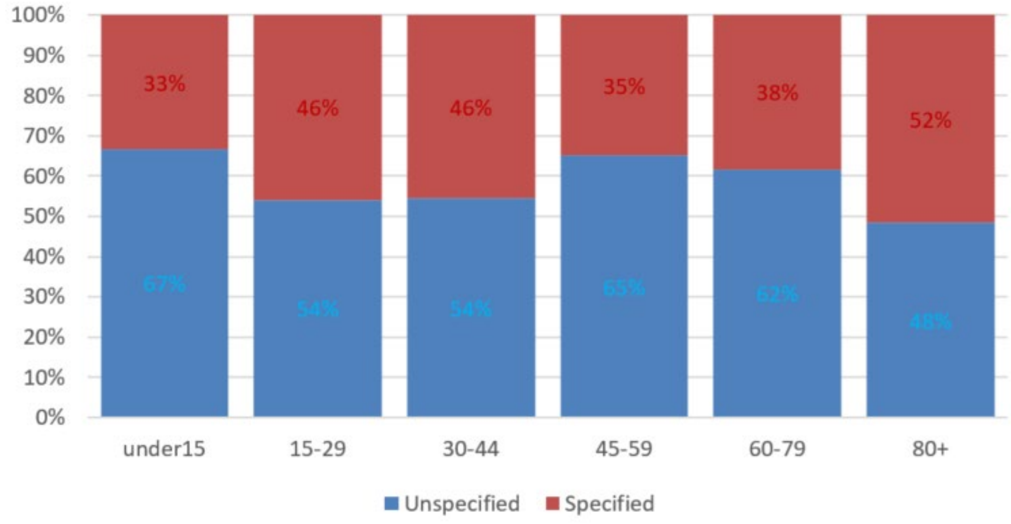

Figure S5. Redistribution of unspecified diabetes type data by age, globally among males in 2015

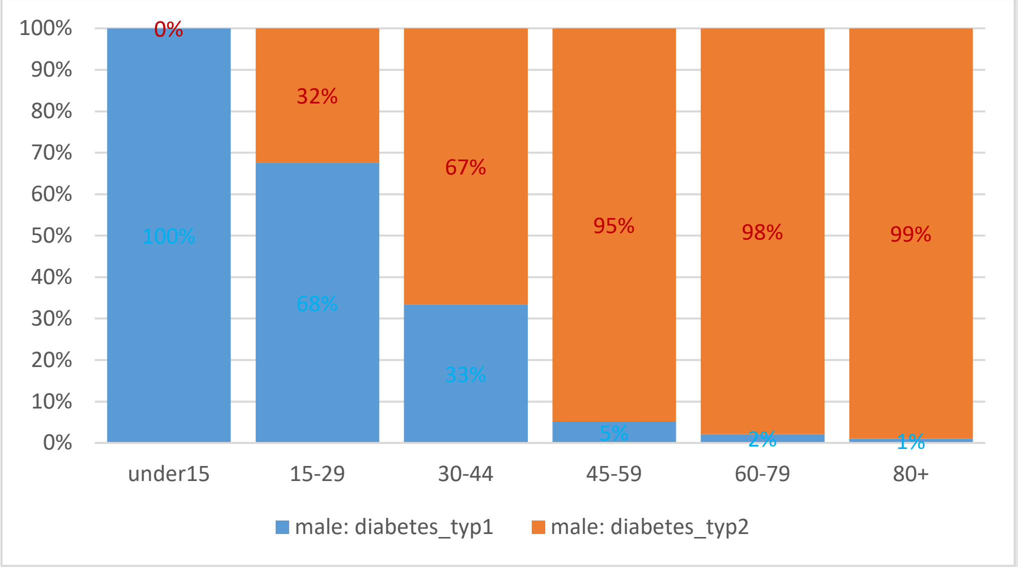

Figure S6. Redistribution of unspecified diabetes type data by age, globally among females in 2015

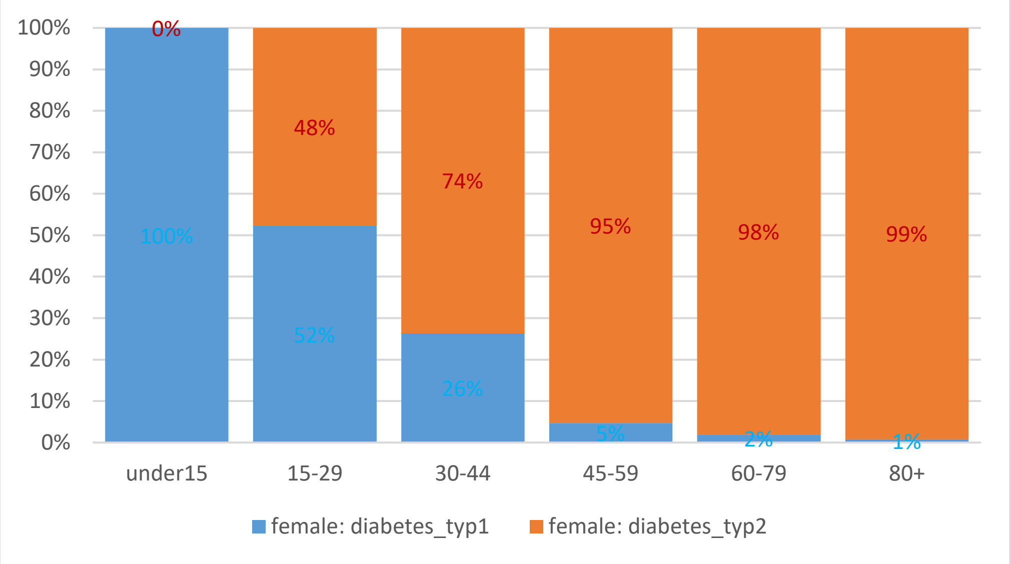

Section 3.3. Modelling strategies

Section 3.3.1. Total diabetes

The Cause of Death Ensemble model (CODEm)[1] was used for deaths due to diabetes estimation. Additional information on CODEm methods can be found in appendix 1, section 3 of the reference article.

For total diabetes estimates, we used two models to estimate overall diabetes deaths with different age restrictions. This is because deaths in younger age groups are almost exclusively due to type 1 diabetes, while deaths in older ages are primarily due to type 2 diabetes. This allowed us to select predictive covariates that are specific to the pathophysiology of type 1 and type 2 diabetes. We set the younger age model from 0-14 years and the older age model from 15-95+ years. We determined the age threshold based on evidence of the onset age of type 2 diabetes occurring at younger ages.

Covariates

The following table lists the covariates included in the total diabetes CODEm models. This requires that the covariate selected for the model must have a directional relationship with diabetes deaths. Covariate directions were selected based on the strength of the evidence.

Table S4. Covariates used in total diabetes fatal modelling

Model	Level	Covariate	Direction
0-14 years	1	Healthcare Access and Quality Index	-
	2	Latitude	+
	2	Percentage of births occurring in women >35 years old	+
	2	Percentage of births occurring in women >40 years old	+
	3	Socio-demographic Index	-
	3	Education years per capita	-
15+ years	1	Age-standardised mean fasting plasma glucose (mmol/L)	+
	1	Age-standardised prevalence of diabetes	+
	1	Mean BMI	+
	1	Prevalence of obesity	+
	2	Mean cholesterol	+
	2	Mean systolic blood pressure	+
	2	Age- and sex-specific summary exposure variable for low fruit	-
	2	Unadjusted grams of sugar	+
	2	Age- and sex-specific summary exposure variable for low vegetables	-
	2	Age- and sex-specific summary exposure variable for alcohol use	+
	3	Healthcare Access and Quality Index	-
	3	Education years per capita	-

	3	Lag-distributed income per capita	+

The following plots show the influence of each covariate on the four CODEm models* (male global, male data rich, female global, and female data rich). A positive standardised beta (to the right) means that the covariate was associated with increased death. A negative standardised beta (to the left) means the covariate was associated with decreased death.

*Note that the data rich CODEm models include only locations that meet high quality data criteria. Global models include all locations, and the two models are hybridized to produce the final CODEm model for a cause of death. The following locations are included in data rich models: Taiwan (Province of China), Armenia, Georgia, Kazakhstan, Kyrgyzstan, Turkmenistan, Uzbekistan, Bulgaria, Croatia, Czechia, Hungary, Poland, Romania, Slovenia, Belarus, Estonia, Latvia, Lithuania, Republic of Moldova, Russian Federation, Ukraine, Japan, Singapore, Australia, New Zealand, Austria, Belgium, Denmark, Finland, France, Germany, Greece, Iceland, Ireland, Israel, Italy, Luxembourg, Malta, Netherlands, Norway, Portugal, Spain, Sweden, Switzerland, United Kingdom, Argentina, Chile, Uruguay, Canada, United States of America, Antigua and Barbuda, Bahamas, Barbados, Belize, Cuba, Grenada, Jamaica, Saint Lucia, Saint Vincent and the Grenadines, Trinidad and Tobago, Ecuador, Colombia, Costa Rica, Guatemala, Mexico, Panama, Venezuela (Bolivarian Republic of), Brazil, Kuwait, Mauritius, Bermuda, Puerto Rico, Saint Kitts and Nevis.

Figure S7. Covariate influence plot for fatal diabetes 0-14 years model

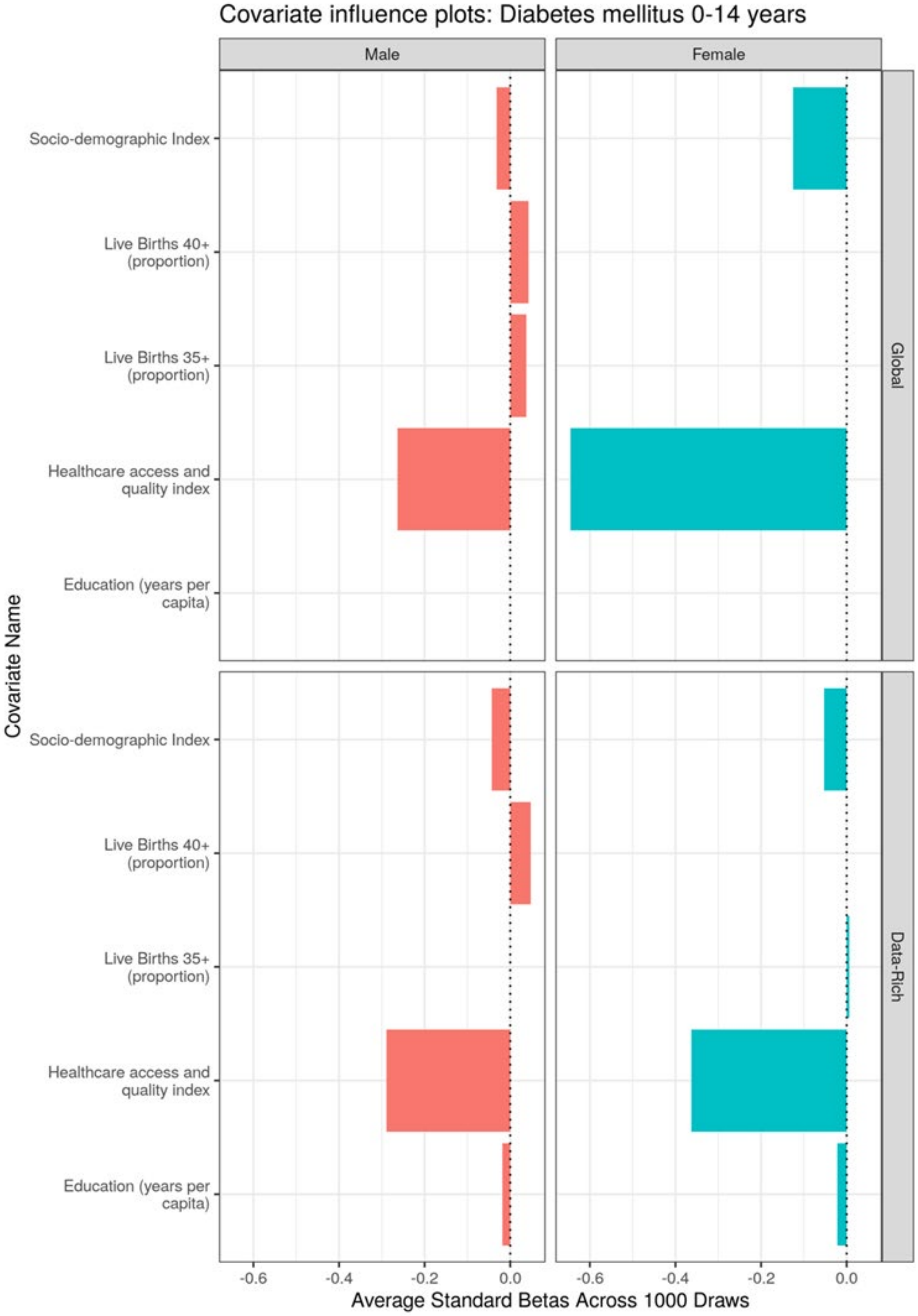

Figure S8. Covariate influence plot for fatal diabetes 15-95+ years model

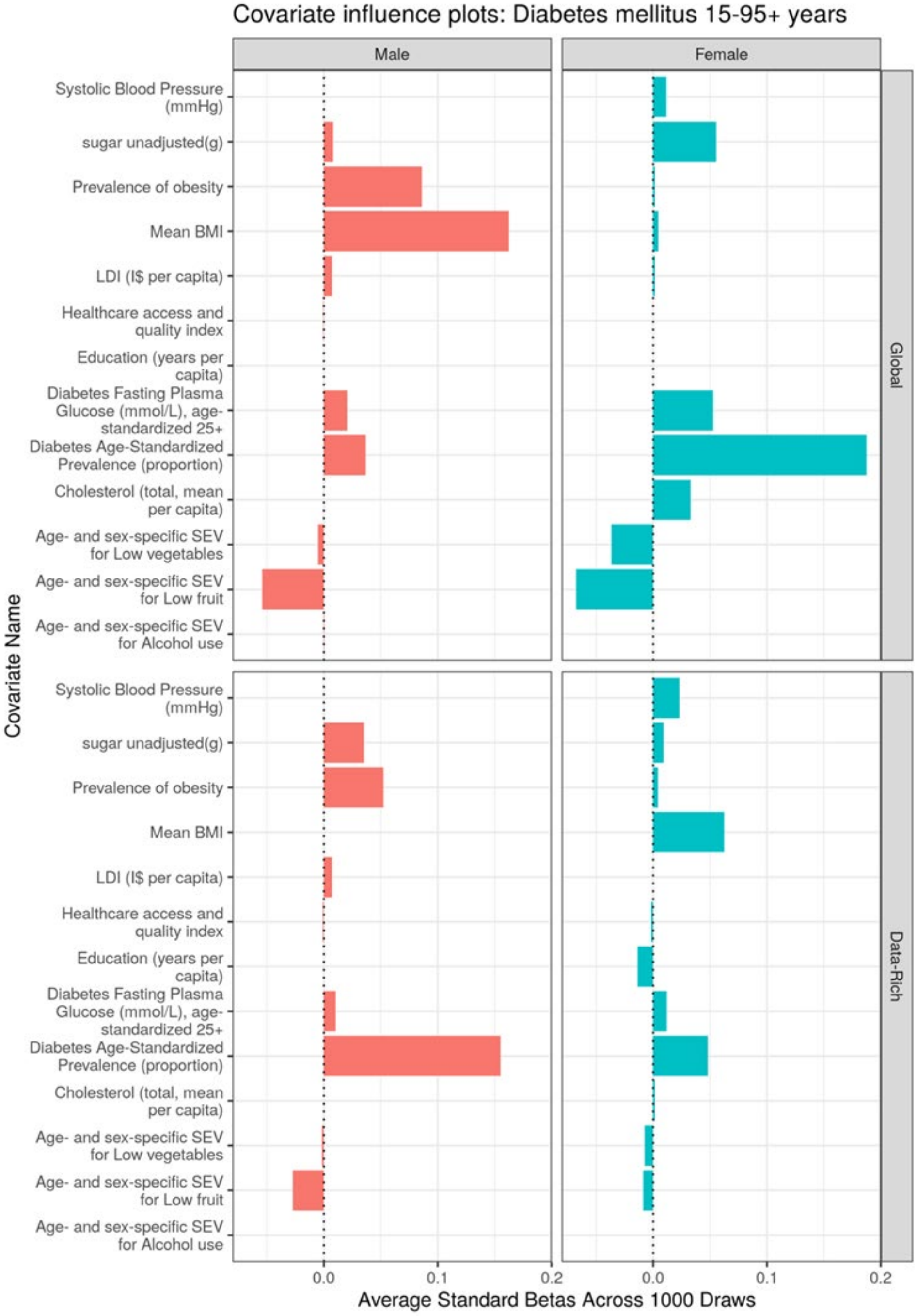

Section 3.3.2. Type 1 & type 2 diabetes

Deaths in younger age groups are almost exclusively due to type 1 diabetes, while deaths in older ages are primarily due to type 2 diabetes. To account for this age pattern, we set the age range of the type 1 diabetes CODEm model to 0-95+ years and the age range of the type 2 diabetes CODEm model to 15-95+ years.

Covariates

The following are the covariates included in the type 1 and type 2 diabetes CODEm models. We selected the same covariates for the type 1 diabetes model as the 0-14 year diabetes total model and the type 2 diabetes model as the 15-95+ year diabetes total model. Covariate directions were selected based on the strength of the evidence.

Table S5. Covariates used in type 1 diabetes and type 2 diabetes fatal modelling

Model	Level	Covariate	Direction
Type 1	1	Healthcare Access and Quality Index	-

	2	Latitude	+
	2	Percentage of births occurring in women >35 years old	+
	2	Percentage of births occurring in women >40 years old	+
	3	Socio-demographic Index	-
	3	Education years per capita	-
Type 2	1	Age-standardised mean fasting plasma glucose (mmol/L)	+
	1	Age-standardised prevalence of diabetes	+
	1	Mean BMI	+
	1	Prevalence of obesity	+
	2	Mean cholesterol	+
	2	Mean systolic blood pressure	+
	2	Age- and sex-specific summary exposure variable for low fruit	-
	2	Unadjusted grams of sugar	+
	2	Age- and sex-specific summary exposure variable for low vegetables	-
	2	Age- and sex-specific summary exposure variable for alcohol use	+
	3	Healthcare Access and Quality Index	-
	3	Education years per capita	-
	3	Lag-distributed income per capita	+

The following plots show the influence of each covariate on the four CODEm models (male global, male data rich, female global, and female data rich). A positive standardised beta (to the right) means that the covariate was associated with increased death. A negative standardised beta (to the left) means the covariate was associated with decreased death.

Figure S9. Covariate influence plot for fatal type 1 diabetes model

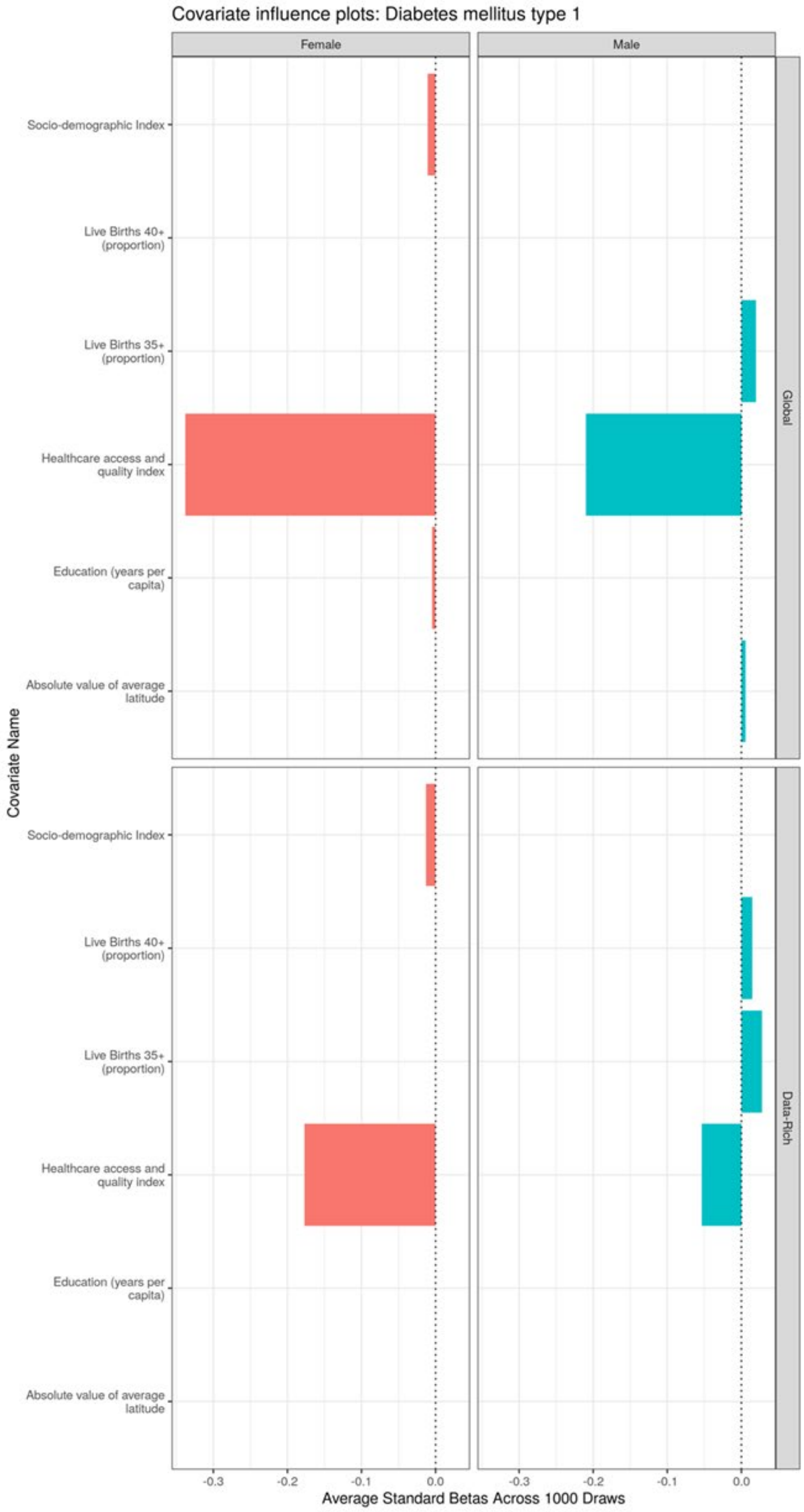

Figure S10. Covariate influence plot for fatal type 2 diabetes model

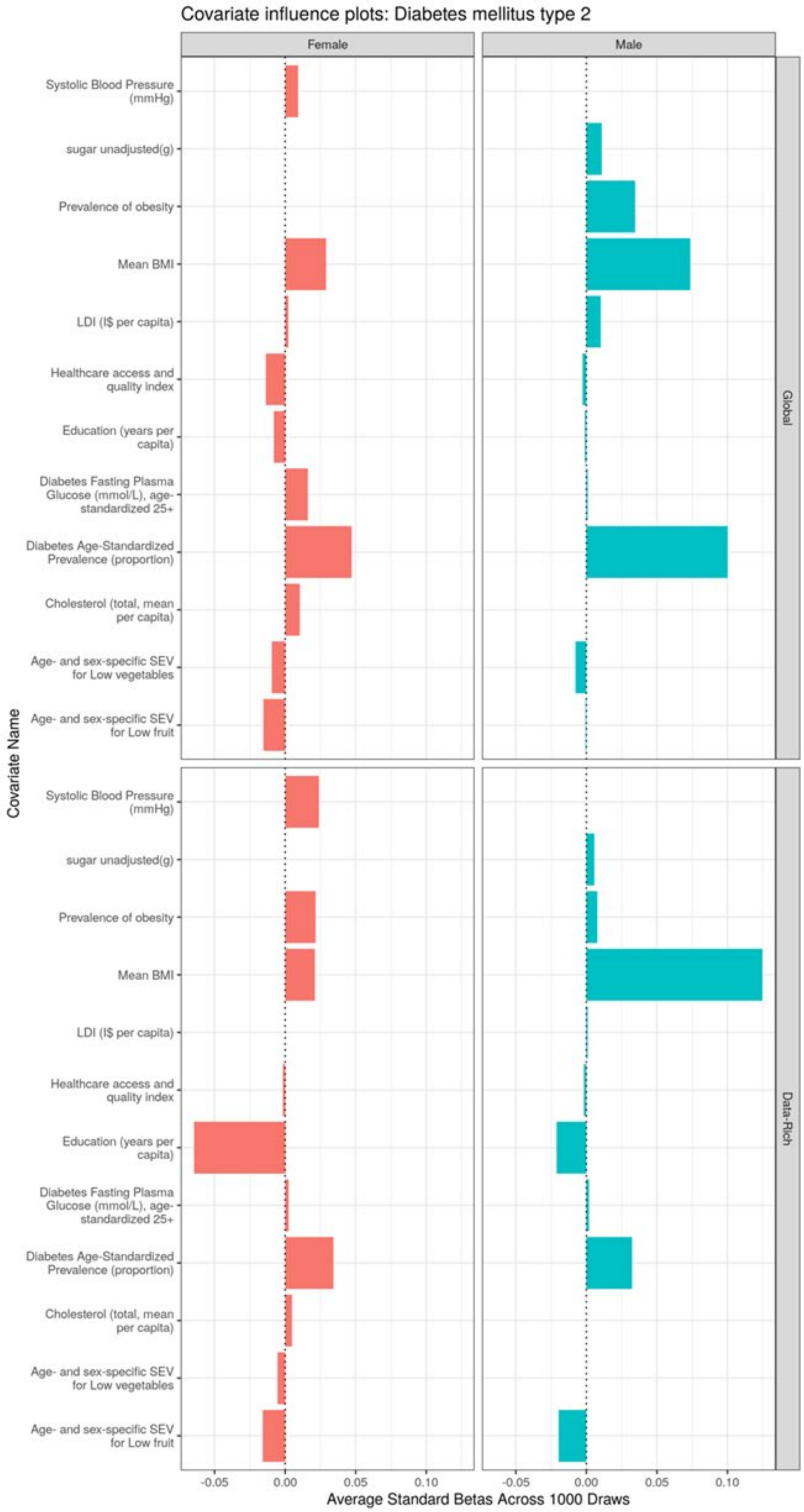

Section 4. Nonfatal[1]

Section 4.1. Sources

Section 4.1.1. Total diabetes & type 1 diabetes

1. Details on systematic reviews conducted before GBD 2019 for diabetes can be found in previous GBD capstones:
   a. GBD 2015: GBD 2015 Disease and Injury Incidence and Prevalence Collaborators. Global, regional, and national incidence, prevalence, and years lived with disability for 310 diseases and injuries, 1990-2015: a systematic analysis for the Global Burden of Disease Study 2015. Lancet. 2016 Oct 8;388(10053):1545-1602. doi: 10.1016/S0140-6736(16)31678-6. Erratum in: Lancet. 2017 Jan 7;389(10064):e1. PMID: 27733282; PMCID: PMC5055577.
   b. GBD 2016: GBD 2016 Disease and Injury Incidence and Prevalence Collaborators. Global, regional, and national incidence, prevalence, and years lived with disability for 328 diseases and injuries for 195 countries, 1990-2016: a systematic analysis for the Global Burden of Disease Study 2016. Lancet. 2017 Sep 16;390(10100):1211-1259. doi: 10.1016/S0140-6736(17)32154-2. Erratum in: Lancet. 2017 Oct 28;390(10106):e38. PMID: 28919117; PMCID: PMC5605509.
   c. GBD 2017: GBD 2017 Disease and Injury Incidence and Prevalence Collaborators. Global, regional, and national incidence, prevalence, and years lived with disability for 354 diseases and injuries for 195 countries and territories, 1990-2017: a systematic analysis for the Global Burden of Disease Study 2017. Lancet. 2018 Nov 10;392(10159):1789-1858. doi: 10.1016/S0140-6736(18)32279-7. Epub 2018 Nov 8. Erratum in: Lancet. 2019 Jun 22;393(10190):e44. PMID: 30496104; PMCID: PMC6227754.
2. A systematic review of the literature was done for GBD 2019 with the following search terms in PubMed:

   Diabetes search string: (diabetes[TI] AND (prevalence[TIAB] OR incidence[TIAB])) OR ('Diabetes Mellitus'[MeSH Terms] AND 'epidemiology'[MeSH Terms]) OR (diabetes[TI] AND 'epidemiology'[MeSH Terms]) NOT gestational[All Fields] NOT ('neoplasms'[MeSH Terms] OR 'neoplasms'[All Fields] OR 'cancer'[All Fields]) NOT ('mice'[MeSH Terms] OR 'mice'[All Fields]) NOT ('schizophrenia'[MeSH Terms] OR 'schizophrenia'[All Fields]) NOT ('emigrants and immigrants'[MeSH Terms] OR ('emigrants'[All Fields] AND 'immigrants'[All Fields]) OR 'emigrants and immigrants'[All Fields] OR 'immigrants'[All Fields]) NOT ('pregnancy'[MeSH Terms] OR 'pregnancy'[All Fields] OR 'gestation'[All Fields]) NOT ('rats'[MeSH Terms] OR 'rats'[All Fields] OR 'rat'[All Fields]) NOT ('kidney'[MeSH Terms] OR 'kidney'[All Fields]) NOT renal[All Fields] NOT ('vitamins'[Pharmacological Action] OR 'vitamins'[MeSH Terms] OR 'vitamins'[All Fields] OR 'vitamin'[All Fields])

   And

   Fasting plasma glucose (FPG) search string: ((“glucose”[Mesh] OR “hyperglycemia”[Mesh] OR “prediabetic state”[Mesh]) AND "Geographic Locations"[Mesh] NOT "United States"[Mesh]) AND ("humans"[Mesh] AND "adult"[MeSH]) AND ("Data Collection" [Mesh] OR "Health Services Research"[Mesh] OR "Population Surveillance"[Mesh] OR "Vital statistics"[Mesh] OR "Population"[Mesh] OR "Epidemiology"[Mesh] OR surve*[TiAb]) NOT Comment[ptyp] NOT Case Reports[ptyp]) NOT "hospital"[TiAb]

   Search date: October 17, 2018

   The search took place for the following dates: 10/15/2017-10/16/2018. The number of studies returned was 717, and the number of studies extracted was 21.

Figure S11. PRISMA diagram of GBD 2019 nonfatal diabetes systematic review

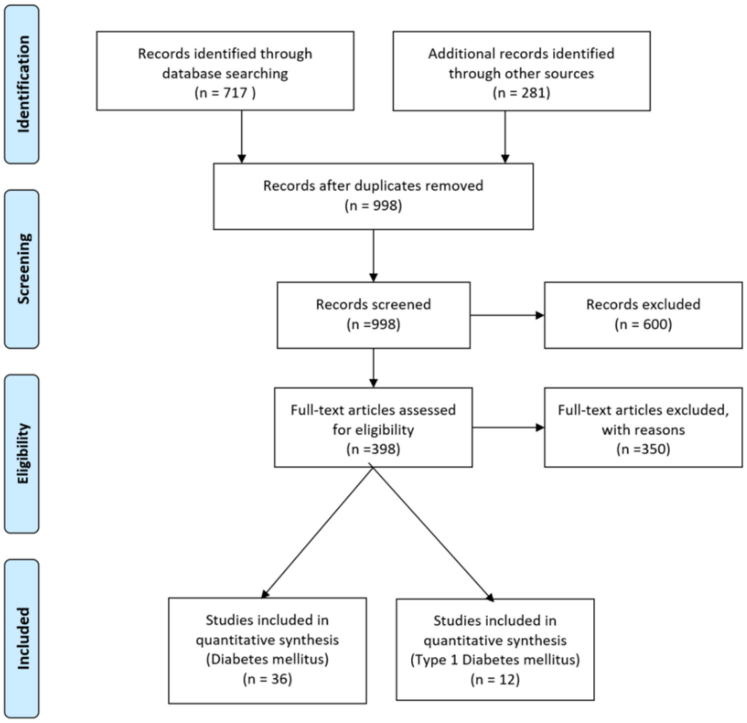

3. Collaborator-provided sources that were either shared directly with us or were identified through searching the Global Health Data Exchange (GHDx) were reviewed for inclusion since GBD 2019.
   a. 115 new sources were included in the total diabetes model since GBD 2019.
      i. Of these new sources, four were also included in the type 1 diabetes model as they were specific to type 1 diabetes mellitus.

4. No systematic review was conducted for the overall diabetes mellitus model since GBD 2019. In place of a systematic review, an "audit" of the current data in the total diabetes mellitus model was undertaken. The audit process involved returning to each data source to re-evaluate inclusion into the model, and to re-check data extractions for those sources that remain eligible for inclusion. GBD 2019 sources (excluding those specific to type 1 diabetes mellitus) and 111 new sources were included in the audit (the four new sources specific to type 1 diabetes mellitus were not included). Main exclusion reasons include duplicative studies, not population representative, and self-report of diabetes status.

Updates 3 and 4 listed above are summarized in the diagram below.

Figure S12. Diagram of data source updates in the nonfatal total diabetes model since GBD 2019

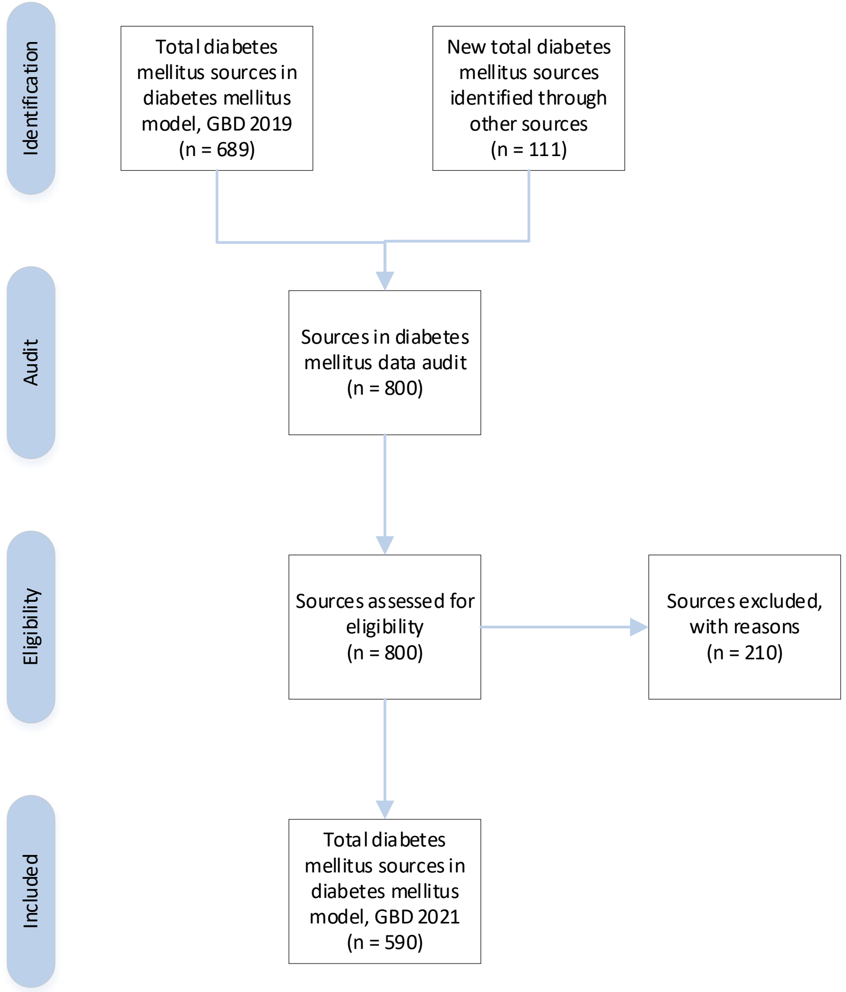

All source count maps below reflect subnational-level location-year counts for countries where GBD estimates at the subnational-level, and national-level location-year counts for countries where GBD does not estimate at the subnational-level. Location-year counts for sources representative at the national-level only for countries where GBD estimates at the subnational-level are not reflected in the maps.

*Figure S13. Sources used in nonfatal total diabetes estimation**

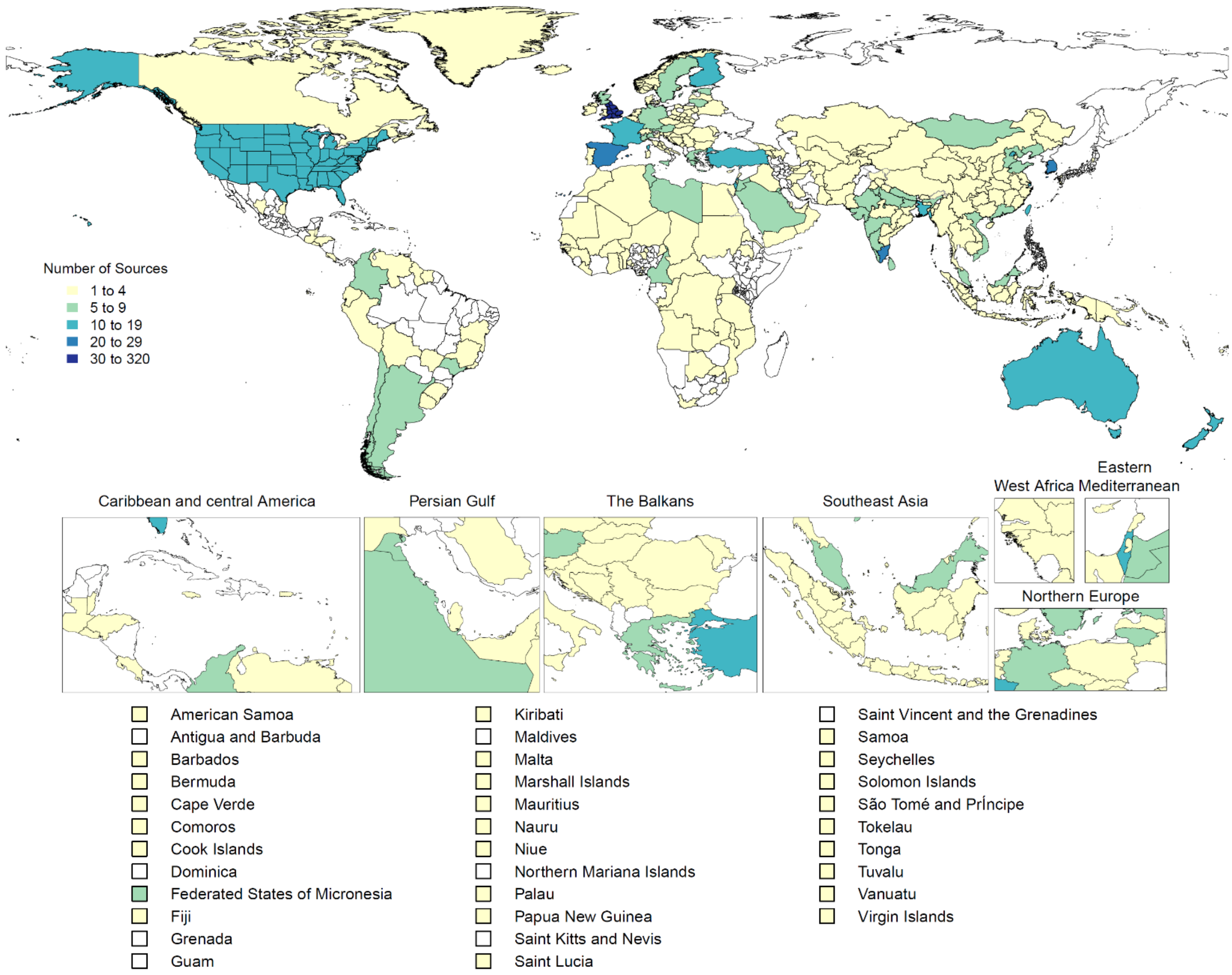

**Only source location-years in the total diabetes nonfatal model. Maps of source location-years in the complication models are below. Note that this map does not reflect subnational sources for Russia, which include 2 location-years in Moscow City, 1 location-year in Karelia, and 2 location-years in Bashkortostan.*

*Figure S14. Sources used in nonfatal type 1 diabetes estimation**

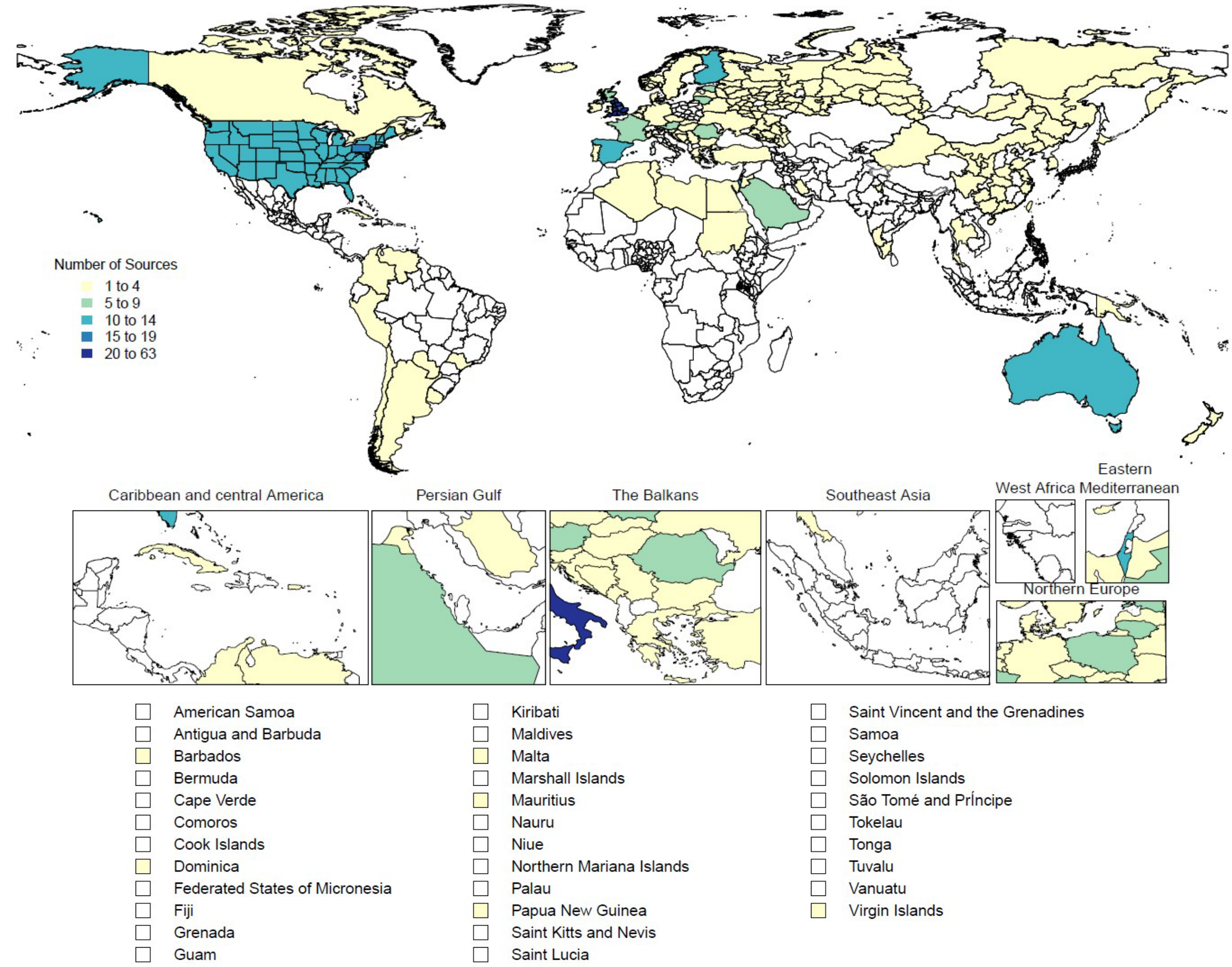

**Only source location-years in the type 1 diabetes nonfatal model. Maps of source location-years in the complication models are below.*

Section 4.1.2. Diabetic outcomes

Diabetic neuropathy, foot ulcer, and amputation due to diabetes

1. The most recent systematic reviews for diabetic neuropathy, diabetic foot ulcer, and amputation due to diabetes were undertaken for GBD 2017. Below are the search terms used in PubMed:

   Diabetic neuropathy: ("Diabetes Mellitus"[MeSH Terms] OR ("diabetes"[All Fields] AND "mellitus"[All Fields]) OR "Diabetes Mellitus"[All Fields]) AND neuropathy[All Fields] AND (proportion OR prevalence OR incidence) NOT gestational NOT cancer NOT mice NOT schizophrenia NOT immigrants NOT gestation NOT rat NOT kidney NOT renal NOT vitamin

   - Dates: 12/31/16-10/17/2017
   - Number of studies returned: 170
   - Number of studies extracted: 1

   Foot ulcer: ((("Diabetes Mellitus"[MeSH Terms] OR ("diabetes"[All Fields] AND "mellitus"[All Fields]) OR "Diabetes Mellitus"[All Fields] OR "diabetes"[All Fields]) AND ("foot"[MeSH Terms] OR "foot"[All Fields]) AND ("ulcer"[MeSH

Terms] OR "ulcer"[All Fields])) NOT ("neoplasms"[MeSH Terms] OR "neoplasms"[All Fields] OR "cancer"[All Fields]) NOT ("mice"[MeSH Terms] OR "mice"[All Fields]) NOT ("emigrants and immigrants"[MeSH Terms] OR ("emigrants"[All Fields] AND "immigrants"[All Fields]) OR "emigrants and immigrants"[All Fields] OR "immigrants"[All Fields]) NOT ("pregnancy"[MeSH Terms] OR "pregnancy"[All Fields] OR "gestation"[All Fields]) NOT ("vitamins"[Pharmacological Action] OR "vitamins"[MeSH Terms] OR "vitamins"[All Fields] OR "vitamin"[All Fields]) NOT renal[All Fields] NOT ("kidney"[MeSH Terms] OR "kidney"[All Fields]) AND (proportion[All Fields] OR "incidence"[All Fields] OR "prevalence"[All Fields]) NOT ("schizophrenia"[MeSH Terms] OR "schizophrenia"[All Fields]) NOT ("rats"[MeSH Terms] OR "rats"[All Fields] OR "rat"[All Fields]))

- Dates: 12/31/16-10/17/2017
- Number of studies returned: 48
- Number of studies extracted: 0

Amputation due to diabetes: (‘Diabetes Mellitus’[MeSH Terms] OR (‘diabetes’[All Fields] AND ‘mellitus’[All Fields]) OR ‘Diabetes Mellitus’[All Fields]) AND ‘amputation’[All Fields] AND (proportion OR prevalence OR incidence) NOT gestational NOT cancer NOT mice NOT schizophrenia NOT immigrants NOT gestation NOT rat NOT kidney NOT renal NOT vitamin

- Dates of search: 12/31/16-10/17/2017
- Number of studies returned: 16
- Number of studies extracted: 1

Figure S15. Sources used in nonfatal diabetic neuropathy estimation

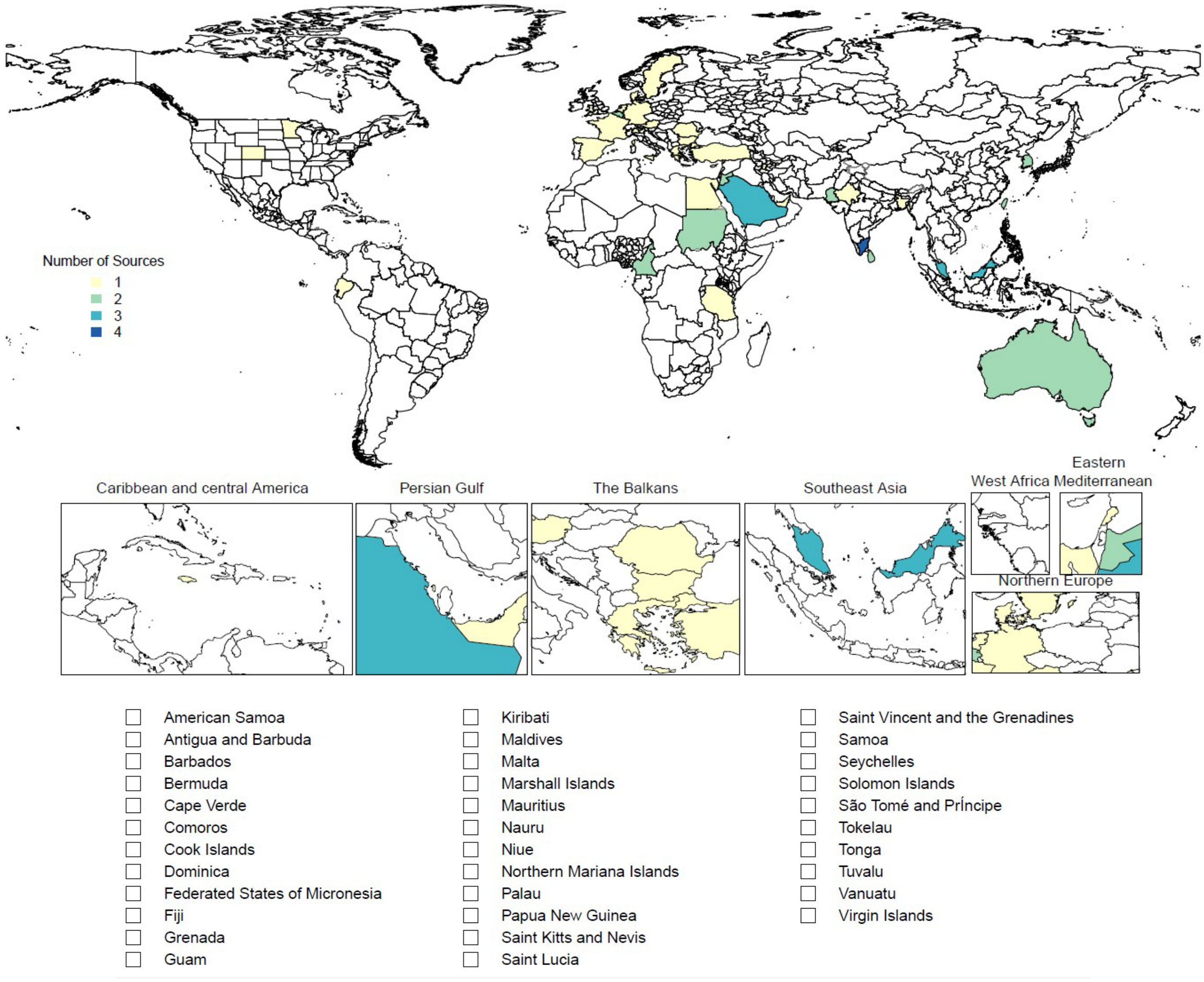

Figure S16. Sources used in nonfatal diabetic foot estimation

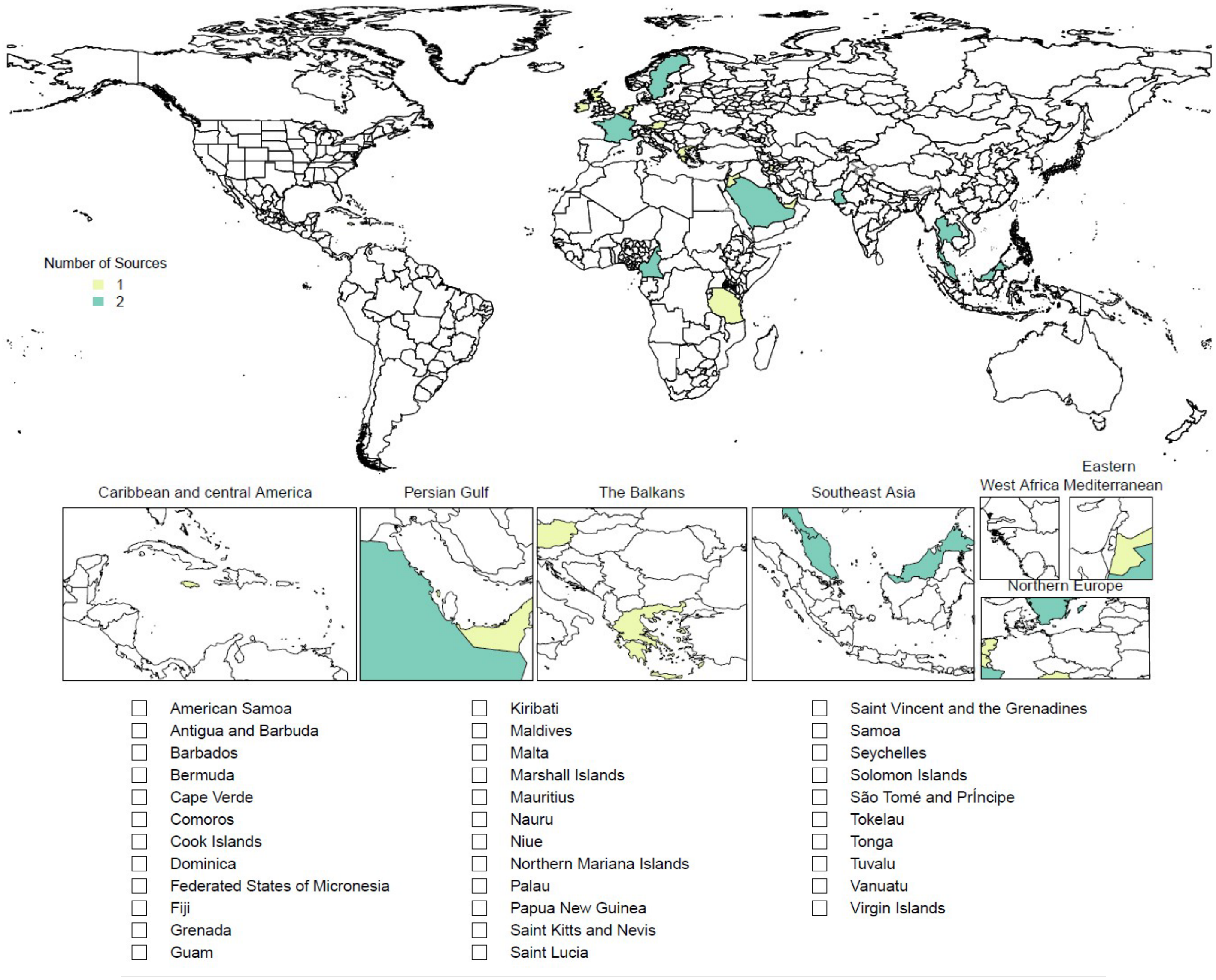

Figure S17. Sources used in nonfatal amputation due to diabetes estimation

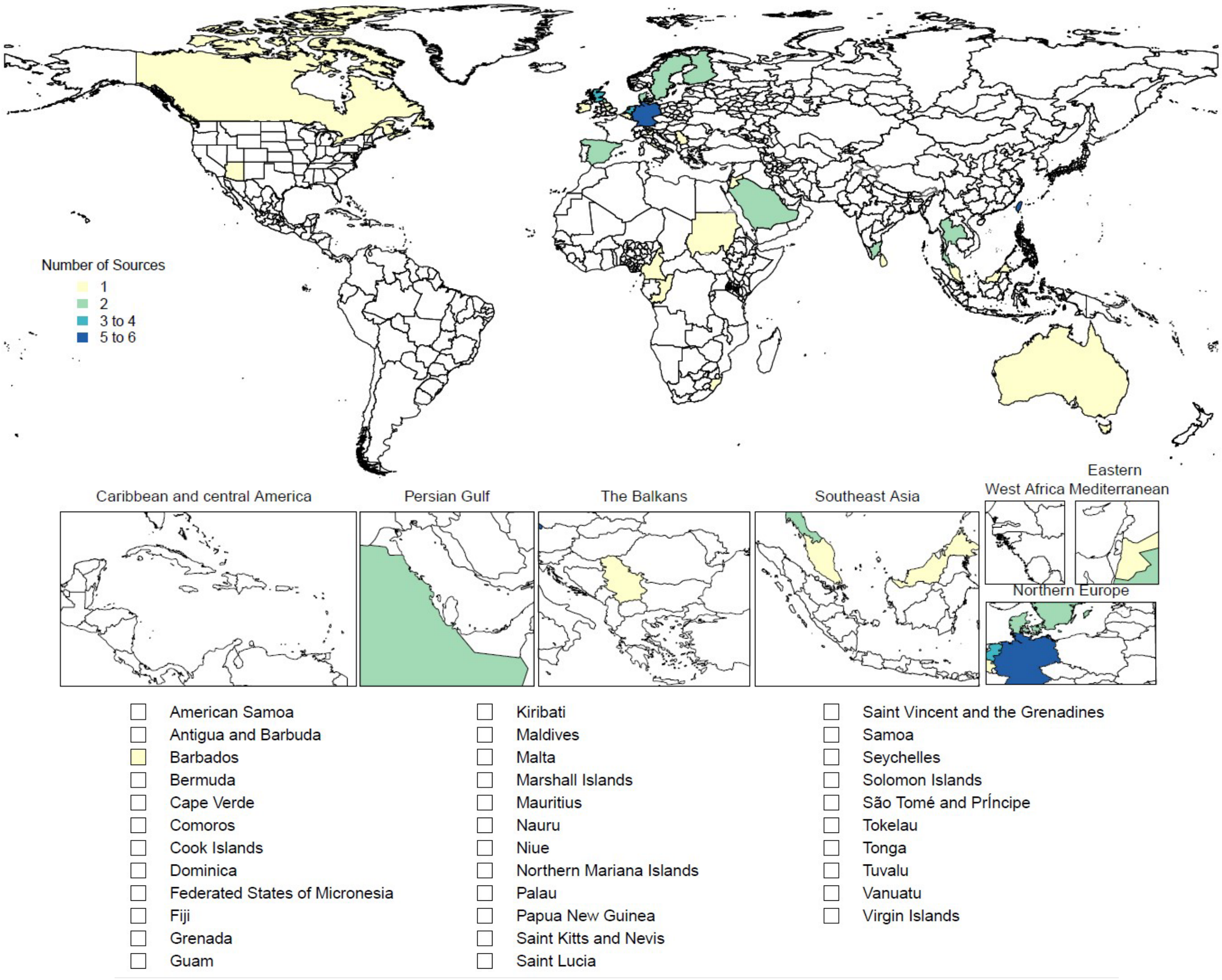

Vision impairment due to diabetes

1. In GBD 2019, we added literature sources from a systematic review conducted by collaborators in the Vision Loss Expert Group where all screened abstracts were sent to regional expert groups to assess data quality for inclusion. Data from 95 of these literature sources that matched GBD inclusion criteria were newly added to vision models (all-cause and cause-specific).
2. The Vision Loss Expert Group also provided additional data provided by principle investigators for existing studies, 51 new Rapid assessment of avoidable blindness (RAAB) surveys, and 5-year disaggregated data for 151 RAAB surveys (previously only data for combined ages 50–-99 were available), which better informed the age pattern for vision impairment in estimates.

Figure S18. Sources used in nonfatal low vision due to diabetic retinopathy estimation

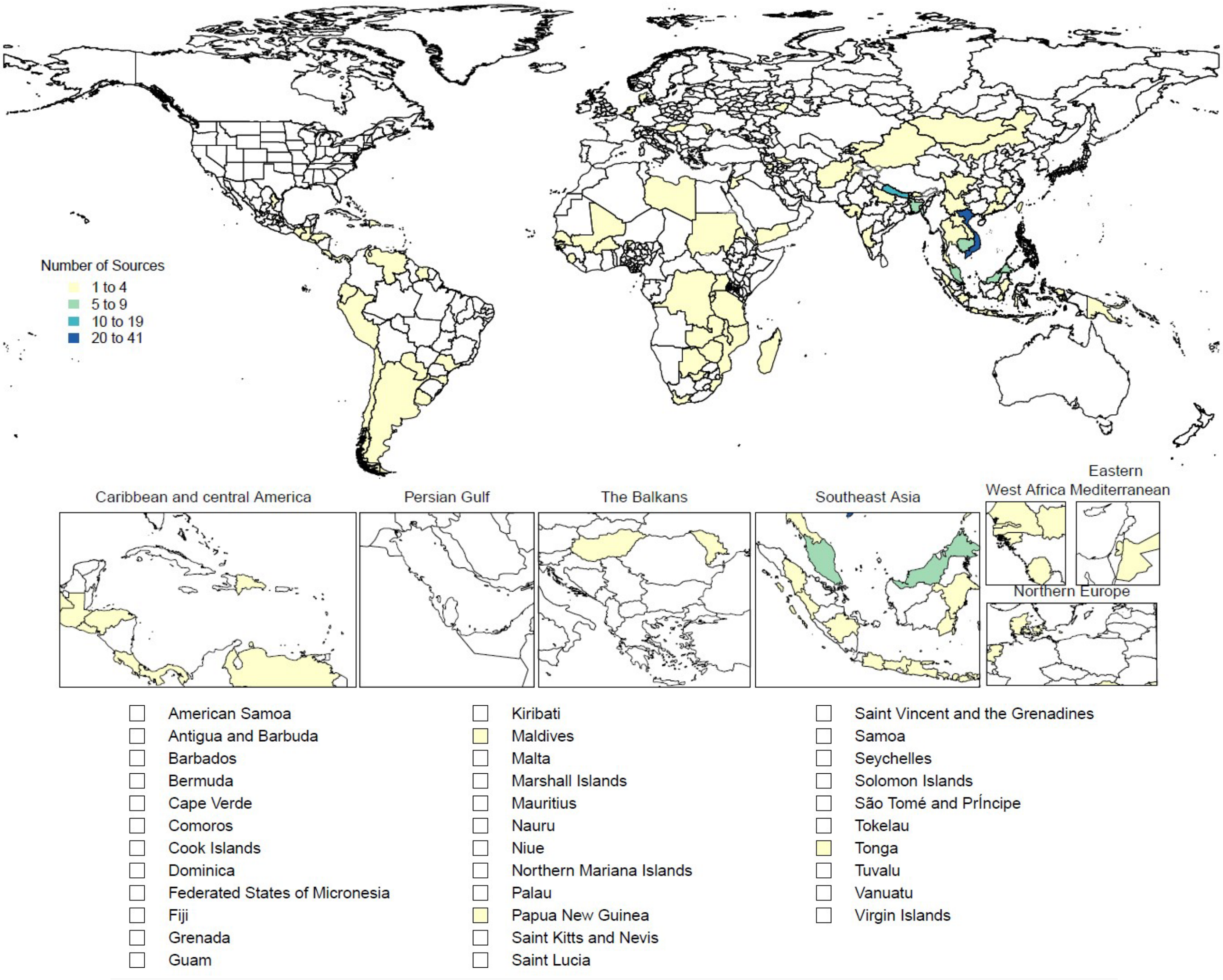

Figure S19. Sources used in nonfatal blindness due to diabetic retinopathy estimation

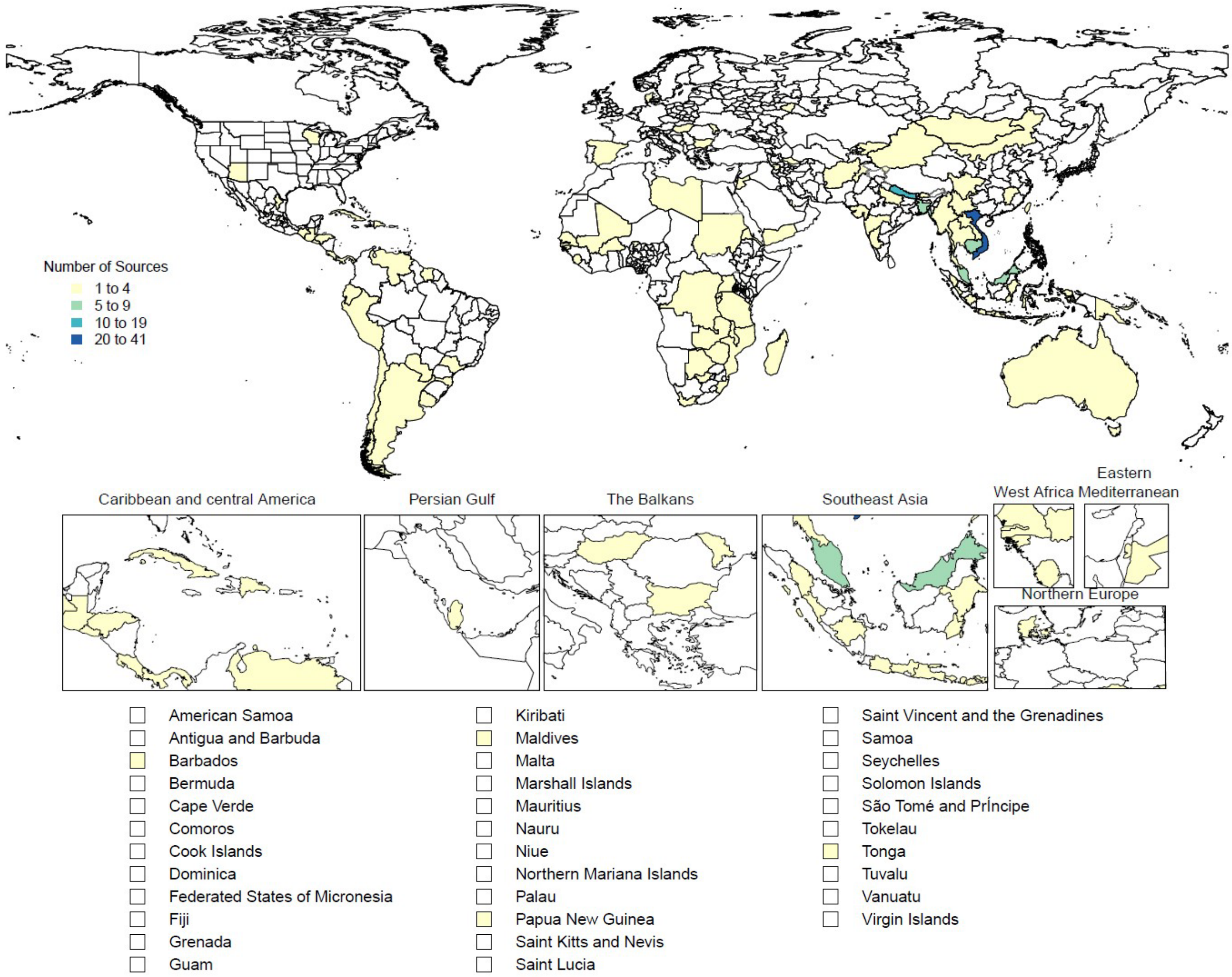

Section 4.2 Data processing

Section 4.2.1. Case definitions

Reference and alternative case definitions and diagnostic criteria are presented in the tables below. The below quantities of interest and case definitions are limited to those for which we model. For example, type 2 diabetes is not included in the table below because it is not modelled; rather, it was calculated by subtracting estimated type 1 diabetes from estimated total diabetes for each sex, age, location, and year.

Table S6. Case definitions for nonfatal diabetes and diabetic outcomes

Quantity of interest	Reference or Alternative	Definition
Diabetes mellitus	Reference	Fasting plasma glucose (FPG) greater than or equal to 126 mg/dl (7 mmol/L) or current treatment (insulin or drugs)
Diabetes mellitus	Alternative	FPG greater than a threshold not equal to 126 mg/dl (7mmol/L) or current treatment (insulin or drugs)
Diabetes mellitus	Alternative	Blood glucose tests measured from glycated hemoglobin (HbA1c) or current treatment (insulin or drugs)
Diabetes mellitus	Alternative	Blood glucose tests measured from oral glucose tolerance test (OGTT) or current treatment (insulin or drugs)

Diabetes mellitus	Alternative	Blood glucose tests measured from post-prandial glucose test (PPG) or current treatment (insulin or drugs)
Diabetes mellitus	Alternative	Combination of non-FPG blood glucose test(s) and FPG or current treatment (insulin or drugs)
Diabetes mellitus	Alternative	Blood glucose tests measured from FPG, HbA1c, OGTT, or PPG (no treatment)
Diabetes mellitus type 1	Reference	Cases of physician diagnosed type 1 diabetes, or type 1 diabetes cases in a diabetic registry or hospital, or any case of diabetes in persons <15 years who are on insulin
Diabetes mellitus type 1	Alternative	Cases of type 1 diabetes determined by c-peptide, islet cell autoantibodies (ICA), Glutamic Acid Decarboxylase Autoantibodies (GADA)
Diabetes mellitus type 1	Alternative	Cases of type 1 diabetes found using pharmacy data, diabetic camps, or another alternative data collection system that is not a registry
Neuropathy	Reference	People with diabetes mellitus who have diabetic neuropathy determined by microfilament test
Neuropathy	Alternative	People with diabetes mellitus who have diabetic neuropathy determined by a test that is not a microfilament test
Diabetic foot	Reference	People with diabetes mellitus who have diabetic foot, which is a poorly healing ulcer
Amputations due to diabetes mellitus	Reference	People with diabetes mellitus who have a lower limb amputation
Amputations due to diabetes mellitus	Alternative	People with diabetes mellitus who have a specific part of the lower limb amputated (eg., toes only, feet only, below ankle only)
Low vision due to diabetic retinopathy	Reference	Low vision (presenting visual acuity of <6/18 ≥3/60 in the better eye using the Snellen chart) from damage to the retina caused by damaged blood vessels due to diabetes. Presenting vision is measured using any corrective lenses currently in use.
Low vision due to diabetic retinopathy	Alternative	Low vision (presenting visual acuity of <6/18 ≥3/60 in the better eye using the Snellen chart) from damage to the retina caused by damaged blood vessels due to diabetes, as measured by Rapid Assessment of Avoidable Blindness (RAAB) surveys.
Blindness due to diabetic retinopathy	Reference	Blindness (acuity in the better eye of <3/60 or <10% visual field around central fixation point) from damage to the retina caused by damaged blood vessels that can leak blood into the retina and cause scarring. Presenting vision is measured using any corrective lenses currently in use.
Blindness due to diabetic retinopathy	Alternative	Blindness (acuity in the better eye of <3/60 or <10% visual field around central fixation point) from damage to the retina caused by damaged blood vessels that can leak blood into the retina and cause scarring as measured by Rapid Assessment of Avoidable Blindness (RAAB) surveys.

Section 4.2.2. Total diabetes

We performed several processing steps to the total diabetes data in order to address sampling and measurement inconsistencies that ensured the data are comparable across data sources and between high fasting plasma glucose (FPG) modelling efforts.

1. *Small sample size*: Data with a sample size of 10 or less were outliered prior to modelling.
2. *Mean FPG processing*: We used an ensemble distribution to estimate the prevalence of diabetes based on mean FPG for sources where data on prevalence of diabetes were not available, but there was data on mean FPG. Essentially, we constructed a distribution based on unit-level data available in 31 different countries. Then we predicted out the prevalence of diabetes by age and sex. This provided the conversion of mean FPG to prevalence of diabetes defined as FPG >126 mg/dL (7 mmol/L). Because this definition is not consistent with our reference case definition (which also includes those on treatment), we then applied an adjustment to adjust these data points to the reference case definition. For information on how these adjustments were made, please see the section "Age splitting and bias adjustments" below.
3. *Age splitting and bias adjustments*: Reported estimates of prevalence were split by age and sex where possible. First, if studies reported prevalence for broad age groups by sex, and also by specific age groups but for both sexes combined, age-specific estimates were split by sex using the sex ratio from within the study. Second, input data reporting prevalence for both sexes that could not be split using a within-study ratio were split using a sex ratio derived from a meta-analysis of existing sex-specific data using the meta-regression—Bayesian, regularized, trimmed (MR-BRT)[3] method. The female to male ratio for diabetes was 0.85 (0.61-1.09). Finally, after the application of bias adjustments, where studies reported estimates across age groups spanning 25 years or more, these were split into five-year age groups using Disease model—Bayesian meta-regression (DisMod-MR)[1] 2.1 from a model that contained the subset of data with age range less than 25 years. Additional information on DisMod-MR 2.1 methods can be found in appendix 1, section 4.5 of the reference article.

We also adjusted estimates from alternative case definitions to the reference case definition. Ratios were constructed between alternative case definitions and the reference case definition using data from surveys that measured glucose level based on different glucose tests on a single person or between survey and the insurance claims data. However, we assumed that claims data in persons <15 years are type 1 diabetes and that 100% of people with diabetes are captured in this age group. Thus, we only adjusted the claims data in persons >15 years. We used MR-BRT analysis to adjust for bias due to commercial insurance or use of alternative case definitions. We performed this analysis in logit-space due to the high prevalence of diabetes (from simulations we learned that for prevalence greater than 50% the log ratio method is biased).

The process of adjusting for non-reference data using MR-BRT with the logit-transformation method is described below:

1. Identify data points with overlapping year, age, sex, and location between alternative case definition and reference case definition
2. Logit transform overlapping data points of alternative and reference case definitions
3. Convert overlapping data points into a difference in logit space using the following equation:
   logit(alternative)-logit(reference)
4. Use the delta method to compute standard errors of overlapping data points in logit space, then calculate standard error of logit difference using the following equation:
   √((variance of alternative)+(variance of reference))
5. Using MR-BRT, conduct a random effects meta-regression to obtain the pooled logit difference of alternative to reference
6. Apply the pooled logit difference to all data points of alternative case definitions using the following equation:
   New estimate = inverse.logit((logit(alternative))-(pooled logit difference))
7. Calculate new standard errors using the delta method, accounting for gamma (between-study heterogeneity)

Table S7. MR-BRT crosswalk adjustment factors for total diabetes

Data input	Reference or alternative case definition	Gamma	Beta Coefficient, Logit (95% UI)*	Adjustment factor (95% UI)**
FPG > 126 mg/dl (7 mmol/L) or Tx	Ref	--	---	---
HbA1c > 6.5%	Alt	0.41	-0.30 (-1.11 – 0.51)	0.74 (0.33 – 1.66)
HbA1c > 6.4% or Tx	Alt	0.31	0.06 (-0.56 – 0.67)	1.06 (0.57 – 1.96)
HbA1c > 6%	Alt	0.57	0.70 (-0.43 – 1.82)	2.01 (0.65 – 6.20)
HbA1c > 6.5% or Tx	Alt	0.29	-0.08 (-0.65 – 0.49)	0.92 (0.52 – 1.63)
FPG > 100 mg/dl (5.6 mmol/L) or Tx	Alt	0.28	1.61 (1.06 – 2.15)	4.98 (2.89 – 8.58)
FPG > 100 mg/dl (5.6 mmol/L)	Alt	0.27	1.55 (1.01 – 2.09)	4.72 (2.76 – 8.08)
FPG > 110 mg/dl (6.1 mmol/L) or Tx	Alt	0.13	0.69 (0.44 – 0.93)	1.99 (1.55 – 2.54)
FPG > 110 mg/dl (6.1 mmol/L)	Alt	0.16	0.59 (0.27 – 0.90)	1.8 (1.31 – 2.47)
FPG > 115 mg/dl (6.4 mmol/L) or Tx	Alt	0.08	0.38 (0.22 – 0.53)	1.46 (1.25 – 1.70)
FPG > 120 mg/dl (6.7 mmol/L)	Alt	0.13	-0.003 (-0.26 – 0.25)	0.997 (0.77 – 1.29)
FPG > 121 mg/dl (6.7 mmol/L)	Alt	0.11	-0.04 (-0.26 – 0.18)	0.96 (0.77 – 1.20)
FPG > 126 mg/dl (7 mmol/L)	Alt	0.14	-0.25 (-0.51 – 0.02)	0.78 (0.60 – 1.02)
FPG > 140 mg/dl (7.8 mmol/L) or Tx	Alt	0.10	-0.27 (-0.48 - -0.07)	0.76 (0.62 – 0.93)
FPG > 144 mg/dl (8 mmol/L) or Tx	Alt	0.12	-0.33 (-0.56 - -0.09)	0.72 (0.57 – 0.91)
OGTT > 180 mg/dl (10 mmol/L) or Tx	Alt	0.17	0.82 (0.45 – 1.19)	2.28 (1.57 – 3.30)
OGTT > 200 mg/dl (11.1 mmol/L)	Alt	0.17	0.41 (0.04 – 0.77)	1.5 (1.04 – 2.15)
OGTT > 200 mg/dl (11.1 mmol/L) or Tx	Alt	0.17	0.41 (0.04 – 0.78)	1.5 (1.04 – 2.18)
FPG > 110 mg/dl (6.1 mmol/L) or OGTT > 200 mg/dl (11.1 mmol/L)	Alt	0.24	1.59 (1.08 – 2.11)	4.92 (2.94 – 8.24)
FPG > 126 mg/dl (7 mmol/L) or OGTT > 200 mg/dl (11.1 mmol/L)	Alt	0.10	0.62 (0.40 – 0.83)	1.85 (1.49 – 2.30)
FPG > 126 mg/dl (7 mmol/L) or OGTT > 200 mg/dl (11.1 mmol/L) or Tx	Alt	0.10	0.62 (0.40 – 0.85)	1.86 (1.49 – 2.33)
FPG > 126 mg/dl (7 mmol/L) or OGTT > 220 mg/dl (12.2 mmol/L)	Alt	0.07	0.36 (0.20 – 0.53)	1.44 (1.22 – 1.70)
FPG > 144 mg/dl (8 mmol/L) or OGTT > 200 mg/dl (11.1 mmol/L) or Tx	Alt	0.17	0.43 (0.06 – 0.80)	1.53 (1.06 – 2.22)
FPG > 140 mg/dl (7.8 mmol/L) or OGTT > 200 mg/dl (11.1 mmol/L) or Tx	Alt	0.18	0.43 (0.06 – 0.81)	1.54 (1.06 – 2.24)
FPG > 140 mg/dl (7.8 mmol/L) or OGTT > 200 mg/dl (11.1 mmol/L)	Alt	0.17	0.43 (0.06 – 0.80)	1.53 (1.06 – 2.22)
FPG > 126 mg/dl (7 mmol/L) or OGTT > 200 mg/dl (11.1 mmol/L) or HbA1c > 6.1%	Alt	0.48	1.30 (0.30 – 2.30)	3.67 (1.35 – 10.00)
US claims	Alt	0.15	-0.62 (-0.92 - -0.31)	0.54 (0.40 – 0.73)
Taiwan claims	Alt	0.38	0.15 (-0.63 – 0.93)	1.16 (0.53 – 2.53)

**MR-BRT crosswalk adjustments can be interpreted as the factor the alternative case definition is adjusted by to reflect what it would have been had it been measured using the reference case definition. If the log/logit beta coefficient is negative, then the alternative is adjusted up to the reference. If the log/logit beta coefficient is positive, then the alternative is adjusted down to the reference.*
***The adjustment factor column is the exponentiated beta coefficient. For log beta coefficients, this is the relative rate between the two case definitions. For logit beta coefficients, this is the relative odds between the two case definitions.*

Section 4.2.3. Type 1 diabetes

We used data that reported incidence, standardised mortality ratio, and prevalence data in claims data for persons <15 years for type 1 diabetes. We decided to not include reported type 1 diabetes prevalence in non-claims sources because we found that their estimates of prevalence and incidence were inconsistent. We decided to trust the incidence data and thus, had to exclude the prevalence data from the model. Similarly, we did not include prevalence of type 1 diabetes in people >15 years from claims sources, because of poor reporting on type of diabetes.

Based on the assumption that claims data in persons <15 years are type 1 diabetes and that 100% of people with diabetes are captured in this age group, we make no adjustments to data in these ages. Claims data are reported as prevalence.

There are a number of different sources and ascertainment methods that were used to identify people with type 1 diabetes. The majority of data reported in the literature are from a diabetic registry, hospital discharge data review, physician interview, or insulin use. We assumed that there is no systematic bias between these sources and considered sources identified through these methods as reference. For the other sources that use alternative ascertainment techniques (eg., pharmacy reports, diabetic camps, school reports), there was not a sufficient amount of data to perform an analysis on each individual type, and the model had relatively few data points in locations where these approaches were used. Therefore, we collapsed all alternative sources and treated the estimates from these sources as defined as an alternative case definition.

Table S8. MR-BRT crosswalk adjustment factors for type 1 diabetes

Data input	Reference or alternative case definition	Beta Coefficient, Logit (95% UI)*	Adjustment factor (95% UI)**
Cases of physician diagnosed type 1 diabetes, or type 1 diabetes cases in a diabetic registry or hospital, or any case of diabetes in persons <15 years who are on insulin	Ref	---	---
Ascertainment through pharmacy, schools, diabetic camps	Alt	-0.11 (-0.22 – 0.10)	0.90 (0.80 – 1.10)

**MR-BRT crosswalk adjustments can be interpreted as the factor the alternative case definition is adjusted by to reflect what it would have been had it been measured using the reference case definition. If the log/logit beta coefficient is negative, then the alternative is adjusted up to the reference. If the log/logit beta coefficient is positive, then the alternative is adjusted down to the reference.*
***The adjustment factor column is the exponentiated beta coefficient. For log beta coefficients, this is the relative rate between the two case definitions. For logit beta coefficients, this is the relative odds between the two case definitions.*

Section 4.2.4. Diabetic outcomes

Diabetic neuropathy, foot ulcer, and amputation due to diabetes

All input data and sources were reviewed for GBD 2019 for diabetic neuropathy, foot ulcer, and amputation due to diabetes. We found that nearly all sources reported estimates in age ranges that exceed 50 years. We identified a single study for each outcome that reported estimates in age range <25 years. We applied this age pattern to the remaining data points.

Due to a lack of data in the diabetic outcome models, no adjustments were undertaken for alternative case definitions, and therefore all case definitions were treated as reference.

Vision impairment due to diabetes

Data on overall vision impairment came from surveys measuring visual acuity in representative population-based studies, either from publications in peer-reviewed and grey literature or surveys for which we had the unit record data. Data were excluded if no test was used of visual acuity that can be converted to the Snellen scale, and if a study did not assess “presenting” or “best-corrected” vision. Presenting vision is the visual acuity as measured with the glasses used by an individual. Best-corrected vision is with the best possible correction for refractive error, regardless of the strength of glasses used by an individual. A subset of these studies that reported vision impairment by cause were used to estimate the prevalence of vision impairment due to diabetic retinopathy.

Several adjustments were made to data extracted from the original data sources.

1. Where studies only reported "both" sex data, a meta-regression in MR-BRT was used to split these datapoints into sex-specific datapoints.
2. Where studies reported visual acuity spanning multiple thresholds (eg, <6/60, rather than separate severe and blind estimates), we applied a logit-difference adjustment meta-regression, using data from studies reporting vision impairment by both severity levels.
3. Some studies reporting all-cause visual acuity provided best-corrected visual acuity, but not presenting visual acuity. We crosswalked these datapoints using a logit-difference meta-regression. This gave us predicted presenting vision impairment datapoints for studies not explicitly reporting presenting visual acuity.
4. Where datapoints spanned more than 25 years of age, we age-split using an algorithm that applies the age pattern of the super-region (from a DisMod-MR 2.1 model that only contains data with age groups that span fewer than 25 years) to split the data to five-year age groups.

Non-reference case definitions were adjusted to reference (full visual acuity exam, presenting vision) using MR-BRT. Betas and exponentiated values, which can be interpreted as an odds ratio, are shown in the tables below for each adjustment for alternative case definitions.

Table S9. MR-BRT crosswalk adjustment factors for low vision due to diabetes

Data input	Reference or alternative case definition	Gamma	Beta Coefficient, Logit (95% CI)*	Adjustment factor**
Does not use rapid methodology	Ref	---	---	---
Uses rapid methodology	Alt	0.70	0.12 (-0.03 – 0.34)	01.13

Table S10. MR-BRT crosswalk adjustment factors for blindness due to diabetes

Data input	Reference or alternative case definition	Beta Coefficient, Logit (95% CI)*	Adjustment factor**
Does not use rapid methodology	Ref	---	---
Uses rapid methodology	Alt	0.06 (-0.03 – 0.15)	01.06

**MR-BRT crosswalk adjustments can be interpreted as the factor the alternative case definition is adjusted by to reflect what it would have been had it been measured using the reference case definition. If the log/logit beta coefficient is negative, then the alternative is adjusted up to the reference. If the log/logit beta coefficient is positive, then the alternative is adjusted down to the reference.*
***The adjustment factor column is the exponentiated beta coefficient. For log beta coefficients, this is the relative rate between the two case definitions. For logit beta coefficients, this is the relative odds between the two case definitions.*

Section 4.3. Modelling strategies

Section 4.3.1. Total diabetes

Below are the DisMod-MR 2.1 model parameters and estimates for the total diabetes model:

1. We set a value prior of 0 for remission for ages 0 to 14
2. We set a value prior of a maximum value of 0.01 for remission for ages 15 to 100
3. We set a value prior of a maximum value of 0.15 for excess mortality for all ages
4. We set a value prior of 0 for incidence for ages 0 to 1
5. We set a value prior of a maximum value of 0.0008 for incidence for ages 1 to 15
6. We set a value prior of a maximum value of 0.1 for incidence for ages 15 to 100

After modelling, we replaced the total diabetes estimates for less than 15 years with the estimates from the type 1 diabetes mellitus model for each age, sex, location, and year within this age range. This was to ensure that the <15 years estimates for total diabetes mellitus and type 1 diabetes mellitus were equivalent, because we assume type 2 diabetes mellitus cannot occur before 15 years.

Covariates

Table S11. Covariates used in total diabetes nonfatal modelling

Covariate	Type	Parameter	Exponentiated beta (95% Uncertainty Interval)
Prevalence of obesity (age-standardised)	Country-level	Prevalence	1.47 (1.32 – 1.63)
Year	Country-level	Prevalence	1.04 (1.04 – 1.04)

Section 4.3.2. Type 1 diabetes

Below are the DisMod-MR 2.1 model parameters and estimates for the type 1 diabetes model:

7. We set a value prior of 0 for remission for all ages

8. We set a value prior of a maximum value of 0.002 for excess mortality for ages 0 to 19
9. We set a value prior of a maximum value of 0 for incidence for ages 0 to 1
10. We set a value prior of a maximum value of 0.0006 for incidence for ages 1 to 20
11. We set a value prior of a maximum value of 0.00033 for incidence for ages 65 to 100

Covariates

Table S12. Covariates used in type 1 diabetes nonfatal modelling

Covariate	Type	Parameter	Exponentiated beta (95% Uncertainty Interval)
Proportion of live births in women 35+ years	Country-level	Incidence	14.66 (11.28 – 19.14)
Maternal education (years per capita)	Country-level	Incidence	1.09 (1.08 – 1.10)
Healthcare access and quality index	Country-level	Excess mortality rate	0.98 (0.98 – 0.98)

Section 4.3.3. Type 2 diabetes

We found that the diagnostic criteria in the methodological sections of papers that report estimates of type 2 diabetes are not sufficiently specific for GBD. Thus, we calculated estimates of type 2 diabetes by subtracting the estimates of type 1 diabetes from estimates of total diabetes for each age, sex, and location from 1990 to 2021.

Section 4.3.4. Diabetic outcomes

Models

The diabetic outcomes estimated in GBD include diabetic neuropathy, diabetic foot ulcer, amputation due to diabetes, moderate vision impairment due to diabetes, severe vision impairment due to diabetes, and blindness due to diabetes.

We estimated amputation due to diabetes, diabetic neuropathy, and diabetic foot for type 1 diabetes and type 2 diabetes using DisMod-MR 2.1. We then multiplied all proportion draws from neuropathy/foot/amputation models by the total diabetes model so that all estimates were in the same population-space.

Below are the DisMod-MR 2.1 model parameters and estimates for these models:

Diabetic neuropathy

1. We set a value prior on the proportion of 0 from ages 0 to 1

Diabetic foot ulcer

2. We set a value prior on the proportion of 0 from ages 0 to 10

Amputation due to diabetes

12. We set a value prior of 0 for incidence for ages 0 to 15
13. We set a value prior of 0 for remission for all ages

The prevalence of vision impairment due to diabetic retinopathy was modelled in two steps. In the first step, we estimated the total prevalence of all-cause presenting vision impairment (the "envelopes") using DisMod-MR 2.1 by severity, including: moderate vision impairment (visual acuity of <6/18 ≥6/60), severe vision impairment (visual acuity of of <6/60 ≥3/60), and blindness (visual acuity of <3/60).

In the second step, we estimated the prevalence of vision impairment due to specific causes, including diabetic retinopathy. Two DisMod-MR 2.1 models for diabetic retinopathy were run: one for the combined category of moderate and severe vision impairment due to diabetes, and one for blindness due to diabetes. Moderate and severe vision impairment due to diabetes were modelled together because input data were mostly available for the aggregate. The minimum age for vision impairment due to diabetes in DisMod-MR 2.1 models was set to 20 years.

We split the moderate plus severe vision estimates for each cause into moderate and severe using the ratio of the presenting moderate and severe vision impairment envelopes. We scaled the sum of cause-specific vision impairment prevalence to the total prevalence of the vision impairment envelopes for each of the three severity levels, resulting in prevalence of vision impairment due to each cause of vision impairment, including diabetes, by severity.

Covariates

Note: No covariates were used in the diabetic neuropathy model.

Table S13. Covariates used in diabetic foot nonfatal modelling

Covariate	Type	Parameter	Exponentiated beta (95% Uncertainty Interval)
Healthcare access and quality index	Country-level	Proportion	0.99 (0.99 – 1.00)

Table S14. Covariates used in amputation due to diabetes nonfatal modelling

Covariate	Type	Parameter	Exponentiated beta (95% Uncertainty Interval)
Healthcare access and quality index	Country-level	Prevalence	1.00 (0.99 – 1.02)

Table S15. Covariates used in vision impairment due to diabetes nonfatal modelling

Model	Covariate	Type	Parameter	Exponentiated beta (95% Uncertainty Interval)
Vision impairment due to diabetes mellitus	Socio-demographic Index	Country-level	Prevalence	0.18 (0.14 – 0.29)
Vision impairment due to diabetes mellitus	Diabetes age-standard prevalence (proportion)	Country-level	Prevalence	2.05 (1.56 – 2.70)
Blindness due to diabetes mellitus	Socio-demographic Index	Country-level	Prevalence	0.17 (0.14 – 0.24)
Blindness due to diabetes mellitus	Diabetes age- standard prevalence (proportion)	Country-level	Prevalence	52.12 (48.23 – 54.49)

Post-processing pipeline

Estimates produced by the diabetic neuropathy, diabetic foot, amputation to diabetes, and vision impairment due to diabetes by severity were run through a splitting process to attribute burden to type 1 diabetes or type 2 diabetes.

First, we ensured that the sum of the prevalence for neuropathy due to diabetes, moderate vision impairment due to diabetes, severe vision impairment due to diabetes, and blindness due to diabetes did not exceed 90% of the prevalence of total diabetes. If the sum exceeded 90% then we rescaled the individual outcomes to 90%. This treats vision impairment and neuropathy as mutually exclusive categories by assuming a patient will not have both simultaneously. From here, we calculated uncomplicated diabetes as the remainder of diabetes cases exclusive of neuropathy and vision impairment.

We performed the same check to ensure that the prevalence of amputation due to diabetes and prevalence of foot ulcer due to diabetes did not exceed 90% of the prevalence of neuropathy due to diabetes. This treats foot ulcer and amputation as mutually exclusive categories by assuming a patient will not have both simultaneously.

In addition, we estimated the prevalence of amputation due to diabetes by splitting into with and without treatment using scaled health systems access (HSA) values. For diabetic amputation, we calculated a distribution of treated versus untreated amputation, defined as receiving a prosthesis or not. We first rescaled the estimates to be between 0 and 0.9, under the assumption that 10% of amputees will not receive a prosthetic, even in high income countries. We based this assumption on the retrospective study by Moore et al[4], which found that about 80% of patients following major lower extremity amputation were fitted with prostheses in the authors' institutions from 1978 to 1986 in the United States. We then performed a population-weighted average of this country-specific value to obtain a proxy for the proportion of amputees that receive a prosthetic by super region. Because these are rough estimates based on large assumptions, we applied confidence intervals of +/- 50% of the value to reflect our uncertainty.

Section 4.4 Disability weights

Section 4.4.1. Diabetic outcomes

We determined the disability weights for each diabetic outcome from the GBD disability weight survey[5]. The table below illustrates the severity levels, lay descriptions, and associated disability weights (DW) applicable for outcomes related to type 1 diabetes and type 2 diabetes:

Table S16. Severity levels and disability weights for diabetic outcomes

Severity level	Lay description	DW (95% CI)
Uncomplicated diabetes mellitus	Has a chronic disease that requires medication every day and causes some worry, but minimal interference with daily activities	0.049 (0.031 – 0.072)

Diabetic neuropathy	Has pain, tingling, and numbness in the arms, legs, hands, and feet. The person sometimes gets cramps and muscle weakness.	0.133 (0.089 – 0.187)
Diabetic neuropathy with diabetic foot	Has a sore on the foot that is swollen and causes some difficulty in walking.	[a]
Diabetic neuropathy with treated amputation	Has lost part of one leg, leaving pain and tingling in the stump. The person has an artificial leg that helps in moving around.	[a]
Diabetic neuropathy with untreated amputation	Has lost part of one leg, leaving pain and tingling in the stump. The person does not have an artificial leg, has frequent sores, and uses crutches.	[a]
Moderate vision impairment due to diabetes mellitus	Has vision problems that make it difficult to recognize faces or objects across a room.	0.031 (0.019 – 0.049)
Severe vision impairment due to diabetes mellitus	Has severe vision loss, which causes difficulty in daily activities, some emotional impact (for example worry), and some difficulty going outside the home without assistance.	0.184 (0.125 – 0.259)
Blindness due to diabetes mellitus	Is completely blind, which causes great difficulty in some daily activities, worry and anxiety, and great difficulty going outside the home without assistance.	0.187 (0.124 – 0.26)

[a] *The disability weights are produced from a combination of two health states: neuropathy and diabetic foot/amputation*

Section 5. Risk factors[1]

Section 5.1 Attributable risk methods

Relative risk analyses were conducted for each of the selected risk factors with diabetes as the outcome. For each risk-outcome pair, diabetes risk as a function of risk factor exposure relative to diabetes risk at the theoretical minimum exposure level (TMREL) was estimated via meta-regression on data identified through systematic literature review. Risk exposure prevalence estimates were generated using Bayesian meta-regresson models, spatiotemporal Gaussian process regression and DisMod-MR 2.1, and TMRELs derived from epidemiological evidence.

Meta-regressions were conducted using the pipeline instantiated in the meta-regression—Bayesian, regularised, trimmed (MR-BRT) tool. Full technical details underlying these methods are provided in Zheng et al.,[3] but in brief, relative risk curves were modeled using a non-linear mixed effect model that allowed for: imposing ß-spline shape constraints that did not assume a log-linear relationship between diabetes and risk exposure, using an ensemble approach to select optimal spline knot placement, accounting for relative risk data over varying range exposures, and applying likelihood-based trimming to identify and remove outliers. A generalized Lasso approach was used to select potential bias covariates, which were then included in the model to adjust for bias. Further explication of these methods is provided in Zheng et al.[6] and GBD 2019 Risk Factor Collaborators.[7]

Details on systematic reviews conducted before GBD 2019 for risk factors can be found in previous GBD capstones:

- GBD 2015: GBD 2015 Risk Factors Collaborators. Global, regional, and national comparative risk assessment of 79 behavioural, environmental and occupational, and metabolic risks or clusters of risks, 1990-2015: a systematic analysis for the Global Burden of Disease Study 2015. Lancet. 2016 Oct 8;388(10053):1659-1724. doi: 10.1016/S0140-6736(16)31679-8. Erratum in: Lancet. 2017 Jan 7;389(10064):e1. PMID: 27733284; PMCID: PMC5388856.
- GBD 2016: GBD 2016 Risk Factors Collaborators. Global, regional, and national comparative risk assessment of 84 behavioural, environmental and occupational, and metabolic risks or clusters of risks, 1990-2016: a systematic analysis for the Global Burden of Disease Study 2016. Lancet. 2017 Sep 16;390(10100):1345-1422. doi: 10.1016/S0140-6736(17)32366-8. Erratum in: Lancet. 2017 Oct 14;390(10104):1736. Erratum in: Lancet. 2017 Oct 28;390(10106):e38. PMID: 28919119; PMCID: PMC5614451.
- GBD 2017: GBD 2017 Risk Factor Collaborators. Global, regional, and national comparative risk assessment of 84 behavioural, environmental and occupational, and metabolic risks or clusters of risks for 195 countries and territories, 1990-

2017: a systematic analysis for the Global Burden of Disease Study 2017. Lancet. 2018 Nov 10;392(10159):1923-1994. doi: 10.1016/S0140-6736(18)32225-6. Epub 2018 Nov 8. Erratum in: Lancet. 2019 Jan 12;393(10167):132. Erratum in: Lancet. 2019 Jun 22;393(10190):e44. PMID: 30496105; PMCID: PMC6227755.

Full details on GBD 2019 modelling strategies, input data, case/exposure definitions, and relative risk estimates are provided in the Supplementary Appendix 1 of GBD 2019 Risk Factor Collaborators,[7] but subsequently, the analysis pipeline for many risk-outcome pairs has been updated with new or updated systematic reviews, inclusion of new input data, revised TMRELs (see Table S17 below), refinements to incorporation of mediation factors, and calculation of revised relative risk curves. Some of these updates are described in Lescinsky et al. 2022 (red meat),[8] Stanaway et al 2022 (vegetables),[9] and Dai et al. 2022 (smoking),[10] and further details are given below per risk factor, particularly in regards to data updates since GBD 2019. Updated data source citations for diabetes relative risk estimates are provided in Table S26.

Table S17. Theoretical minimum risk exposure levels

**Risk Factor**	**TMREL**
Diet low in fruits	340-350 g/day
Diet low in vegetables	306-372 g/day
Diet low in whole grains	160-210 g/day
Diet high in red meat	0-200 g/day
Diet high in processed meat	0 g/day
Diet high in sugar-sweetened beverages	0 g/day
Diet low in dietary fibre	22-25 g/day
Low physical activity	3600-4400 MET-minutes/week
High body-mass index (BMI)	20-21 kg/m2
High fasting plasma glucose (FPG)	4.8-5.4 mmol/L
High alcohol use	*
Smoking	0
Second-hand smoke	0
Ambient particulate matter pollution (PM 2.5)	2.5-5.9 μg/m3 PM2.5
Household air pollution (HAP) from solid fuels	2.5-5.9 μg/m3 PM2.5
High air temperature	**
Low air temperature	**

* Alcohol consumption that is equivalent to that of a non-drinker by age-sex-location. For more information see: DOI:https://doi.org/10.1016/S0140-6736(22)00847-9
** Temperature that minimizes temperature-attributable deaths by year-location-temperature zone. For more information see: DOI: https://doi.org/10.1016/S0140-6736(21)01700-1

Section 5.1.1. High alcohol use

We defined exposure as the grams per day of pure alcohol consumed among current drinkers. We constructed this exposure using the indicators outlined below:

1. Current drinkers, defined as the proportion of individuals who have consumed at least one alcoholic beverage (or some approximation) in a 12-month period.
2. Alcohol consumption (in grams per day), defined as grams of alcohol consumed by current drinkers, per day, over a 12-month period.
3. Alcohol litres per capita (LPC) stock, defined in LPC of pure alcohol, over a 12-month period.

We also used three additional indicators to adjust alcohol exposure estimates to account for different types of bias:

1. Number of tourists within a location, defined as the total amount of visitors to a location within a 12-month period.
2. Tourists' duration of stay, defined as the number of days resided in a hosting country.
3. Unrecorded alcohol stock, defined as a percentage of the total alcohol stock produced outside established markets.

A systematic review of the literature was performed to extract data on our primary indicators. The Global Health Exchange (GHDx), IHME's online database of health-related data, was searched for population survey data containing participant-level information from which we could formulate the required alcohol use indicators on current drinkers and alcohol consumption. Data sources were included if they captured a sample representative of the geographical location under study. We documented relevant survey variables from each data source in a spreadsheet and extracted using STATA 13.1 and R 3.3. A total of 6926 potential data sources were available in the GHDx, of which 5764 have been screened and 1206 accepted.

Since GBD 2019, we undertook an effort to update the type 2 diabetes risk curve. We refined the search strings to capture a larger number of studies than was identified by previous search strings. Studies published between 01/01/1970 and 12/31/2019 were reviewed. Of those articles captured, cohort and case-control studies were included if they reported an association between alcohol use and type 2 diabetes, a continuous dose for alcohol consumption, and effect size (relative risk, hazard ratio, or odds ratio) with uncertainty. Information on study type, confounders controlled for, sample representativeness, and measurement of exposure and outcomes was also extracted.

Section 5.1.2. Dietary risks

Below are the dietary risk factor exposure definitions:

- Diet low in fruit: Average daily consumption (in grams per day) of fruit including fresh, frozen, cooked, canned, or dried fruit, excluding fruit juices and salted or pickled fruits
- Diet low in vegetables: Average daily consumption (in grams per day) of vegetables, including fresh, frozen, cooked, canned, or dried vegetables and excluding legumes and salted or pickled vegetables, juices, nuts and seeds, and starchy vegetables such as potatoes or corn
- Diet low in whole grains: Average daily consumption (in grams per day) of whole grains (bran, germ, and endosperm in their natural proportion) from breakfast cereals, bread, rice, pasta, biscuits, muffins, tortillas, pancakes, and other sources
- Diet low in fiber: Average daily consumption (in grams per day) of fiber from all sources including fruits, vegetables, grains, legumes, and pulses
- Diet high in red meat: Average daily consumption (in grams per day) of unprocessed red meat including pork and bovine meats such as beef, pork, lamb, and goat, but excluding all processed meats, poultry, fish, and eggs
- Diet high in processed meat: Average daily consumption (in grams per day) of meat preserved by smoking, curing, salting, or addition of chemical preservatives
- Diet high in sugar-sweetened beverages: Average daily consumption (in grams per day) of beverages with ≥50 kcal per 226.8 gram serving, including carbonated beverages, sodas, energy drinks, and fruit drinks, but excluding 100% fruit and vegetable juices

The dietary data that we use in the exposure models come from multiple sources, including nationally and subnationally representative nutrition surveys using 24-hour dietary recall methodology, food frequency questionnaires (FFQ), household budget surveys (HBS), accounts of national sales from the Euromonitor ("sales"), and food availability data from the Food and Agriculture Organization of the United Nations (FAO). We did not make any updates to the exposure data sources used in the dietary risk factor models since GBD 2019. In GBD 2019, we had included new dietary recall sources from a literature search of PubMed and updates to yearly known survey series from the IHME GHDx in our models.

Since GBD 2019, we conducted two additional systematic reviews that examine the health effects of vegetable and red meat consumption on various outcomes, including type 2 diabetes.

Section 5.1.3. Environmental/occupational risks

High/low air temperature

We assess the daily exposure to non-optimal temperature, which is defined for high temperatures (above the TMREL) and low temperatures (below TMREL). TMRELs for non-optimal temperature exposure vary by year and location and reflect location-specific adaptation to temperature as well as specific composition of causes of death.

Household air pollution

Exposure to household air pollution from solid fuels (HAP) is estimated from both the proportion of individuals using solid cooking fuels and the level of exposure to particulate matter less than 2.5 micrometers in diameter (PM2.5) air pollution for these individuals. Solid fuels in our analysis include wood, coal/charcoal, dung, and agricultural residues.

As in GBD 2019, household air pollution was modelled at the individual level using a three-step modelling strategy implementing linear regression, spatiotemporal regression, and Gaussian Process process Regression regression (GPR). Since GBD 2019, we updated the HAP proportion model to disaggregate estimates of solid fuel use to estimate the proportion of individuals using each of the following component fuel type categories: 1) coal or charcoal, 2) crop residue, 3) dung, and 4) wood. With this strategy, we can more finely characterize characterise individual exposure to PM2.5 due to solid fuel use by applying fuel-specific mapping values to fuel-specific proportion estimates. This change addresses an important limitation in our model, in that it previously assumed equal PM2.5 exposure for all solid fuel categories. Fuel type-specific estimates were generated by first using ST-GPR to generate location- and year-specific estimates for coal, crop, dung, and wood. ST-GPR was also used to create estimates for the parent solid fuel category, as in GBD 2019. The first step of the ST-GPR modelling process is a mixed-effect linear regression of logit-transformed proportion of individuals using solid cooking fuels. For each of the linear models, maternal education and the proportion of population living in urban areas were used as covariates. These models also included nested random effects by GBD region and GBD super-region.

The four fuel-type-specific proportion estimates were then squeezed to the estimates for the overall proportion of individuals using solid fuel for cooking. For each location and year, we used the following formula, where $prop_{coal}$, $prop_{crop}$, $prop_{dung,}$ $prop_{wood}$, and $prop_{solid}$ indicate the proportion of individuals using coal, crop, dung, wood, or any type of solid fuel, respectively.

$$\text{Let } prop_{total} = prop_{coal} + prop_{wood} + prop_{crop} + prop_{dung}$$

$$S = prop_{total} \,/\, prop_{solid}$$

For each fuel category, with coal shown below as an example, the adjusted (squeezed) proportion is calculated as

$$prop_{coal}{}' = prop_{coal} \,/\, S$$

In preliminary model iterations, we mapped mixed fuel strings (eg, "wood and agricultural residues") to the category associated with highest $PM_{2.5}$ exposure to avoid underestimating HAP exposure. However, fuel-specific ST-GPR models were unstable with this approach. We therefore excluded mixed-fuel string studies from final estimates for fuel-specific proportions, though we retained these studies when modelling the proportion of overall solid fuel use.

Relative risk for HAP and type 2 diabetes is calculated jointly with ambient particulate matter pollution.

Ambient particulate matter pollution

Exposure to ambient particulate matter pollution is defined as the population-weighted annual average mass concentration of particles with an aerodynamic diameter less than 2.5 micrometers ($PM_{2.5}$) in a cubic meter of air. This measurement is reported in $\mu g/m^3$. Ambient air pollution exposure estimates use input data from multiple sources. These include satellite observations of aerosols in the atmosphere, ground monitor measurements, chemical transport model simulations, population estimates, and land-use data.

Ground monitor measurements were updated to include more recent measurements from sites included in GBD 2019 and additional measurements from new monitors. New data were added to the database from several sources, including the European Environment Agency, United States Environmental Protection Agency, and the OpenAQ database. The complete, updated dataset included measurements of PM_{10} and $PM_{2.5}$ concentrations between 2018 and 2020 from 18,406 ground monitors from 120 countries, primarily from the USA, China, European countries, and USA embassies and consulates. Annual averages were excluded if they were based on less than 75% coverage within a year unless there was already sufficient data within the country of interest (monitor density greater than 0.1). If information on coverage was not available, data were included. For sites with PM_{10} measurements only, these observations were converted from PM_{10} to $PM_{2.5}$ measurements using a hierarchy of conversion factors ($PM_{2.5}/PM_{10}$ ratios): (i) where possible, a "local" conversion factor was used, constructed as the ratio of the average measurements (of $PM_{2.5}$ and PM_{10}) from within 50 km of the location of the PM_{10} measurement, and within the same country, if such measurements were available; (ii) where local information was not sufficient to construct a conversion factor, a country-wide conversion factor was used; and (ii) where appropriate information within a country did not exist, a region-level factor was used. In each case, to avoid the possible effects of outliers in the measured $PM_{2.5}$ and PM_{10} data, extreme values of the ratios were excluded. These extreme values were defined as those greater/lesser than the 95th and 5th quantiles of the empirical distributions of conversion factors. As with the GBD 2013, 2015, 2016, 2017, and 2019 databases, in addition to values of $PM_{2.5}$ and whether they were direct measurements or conversions from PM_{10}, the updated database also included additional information (where available) concerning the ground measurements, such as monitor geo-coordinates and monitor site type.

Global satellite-derived estimates (V4.GL.03.NoGWR) used as inputs to DIMAQ2 for 1998–2019 and for January to August 2020 are used at 0.1^{o} x 0.1^{o} resolution (~11 x 11 km resolution at the equator). The algorithm uses aerosol optical depth (AOD) from several updated satellite products (MAIAC, MODIS, and MISR). Ground-based observations from a global sunphotometer network (AERONET version 3) are used to combine different AOD information sources. The GEOS-Chem chemical transport model was used

for geophysical relationships between surface $PM_{2.5}$ and AOD. Since GBD 2019, an additional update to biomass burning emissions from 2015 to 2020 was made. This update allows for time-varying biomass burning emissions in the simulation for those years, where they had previously been unavailable after 2014. Given lags in releases of available meteorological information used in the GEOS Chem simulations, for September to December 2020, the estimates incorporate satellite retrievals from 2020, but GEOS-Chem simulated values for 2019 as well as biomass burning emissions from 2019. Further, satellite retrievals for all of 2020 were limited to MODIS DT, DB, and MAIAC. We included MISR inputs for January to June 2020 only, as this product was not available past June when the satellite-based estimates were generated.

Estimates of the sum of particulate sulfate, nitrate, ammonium, and organic carbon and the compositional concentrations of mineral dust simulated using the GEOS-Chem chemical transport model, and a measure combining elevation and the distance to the nearest urban land surface were available for 2000–2020 for each 0.1o × 0.1o grid cell.

We obtained a comprehensive, high-resolution gridded population dataset from the Gridded Population of the World (GPW) database. Estimates for 2000, 2005, 2010, 2015, and 2020 were available from the GPW version 4, with estimates for 1990 and 1995 obtained from the GPW version 3. These data are provided on a $0.0083^{o} \times 0.0083^{o}$ resolution. Aggregation to each $0.1^{o} \times 0.1^{o}$ grid cell was accomplished by summing the central 12 × 12 population cells. Populations estimates for 2001–2004, 2006–2009, 2011–2014, and 2016–2019 were obtained by interpolation using natural splines with knots placed at 2000, 2005, 2010, 2015, and 2020. This was performed for each grid cell.

Section 5.1.4. Low physical activity

Low physical activity is defined as objectively measured, total physical activity less than 3600 to 4400 MET-minutes per week. We assess physical activity performed by adults older than 25 years of age, for duration of at least ten minutes at a time, across all domains of life (leisure/recreation, work, household and transport). We use frequency, duration, and intensity of activity to calculate total metabolic equivalent (MET)-minutes per week. MET is the ratio of the working metabolic rate to the resting metabolic rate. One MET is equivalent to 1 kcal/kg/hour and is equal to the energy cost of sitting quietly. A MET is also defined as the oxygen uptake in ml/kg/min with one MET equal to the oxygen cost of sitting quietly, around 3.5 ml/kg/min.

We included surveys of the general adult population that captured self-reported physical activity in all domains of life (leisure/recreation, work/household, and transport), where random sampling was used. Data were primarily derived from two standardised questionnaires: The Global Physical Activity Questionnaire (GPAQ) and the International Physical Activity Questionnaire (IPAQ), although we included other survey instruments that asked about intensity, frequency, and duration of physical activity performed across all activity domains.

Since GBD 2019, we conducted an updated systematic review for studies published before December 31, 2019, evaluating the relationship between physical activity and risk of diabetes. We included prospective cohort studies that assessed total physical activity or leisure-time physical activity as the exposure variable and diabetes as an outcome. Further, we only included studies that reported risk estimates (relative risk, hazard ratio, or odds ratio) with confidence intervals, standard errors, or enough information to quantify uncertainty. In addition, we only included studies that reported the frequency and duration of activity achieved, excluding studies that reported physical activity using categorical or custom component scores. Below are the PubMed search strings used:

physical activity[Title/Abstract] AND type 2 diabetes[Title/Abstract] AND "humans"[MeSH Terms] AND English[lang] AND ("2014/10/01"[PDAT] : "2019/12/31" [PDAT])

physical activity[Title/Abstract] AND noninsulin dependent diabetes mellitus [Title/Abstract] AND "humans"[MeSH Terms] AND English[lang] AND ("2014/10/01"[PDAT] : "2019/12/31" [PDAT])

physical activity[Title/Abstract] AND niddm[Title/Abstract] AND "humans"[MeSH Terms] AND English[lang] AND ("2014/10/01"[PDAT] : "2019/12/31" [PDAT])

Section 5.1.5. Tobacco

Smoking

We estimated the prevalence of current smoking and the prevalence of former smoking using data from cross-sectional nationally representative household surveys. We defined current smokers as individuals who currently use any smoked tobacco product on a daily or occasional basis. We defined former smokers as individuals who quit using all smoked tobacco products for at least six

months, where possible, or according to the definition used by the given survey. Our survey data extraction method for smoking exposure has not changed from previous GBD cycles.

Since GBD 2019, we undertook an effort to improve our relative risk curves by refining our search strings to capture a larger number of studies than was identified by previous search strings. Studies published between 01/01/1970 and 12/31/2019 were reviewed. Of those articles captured, prospective cohort and case-control studies were included if they reported the effect sizes (relative risk, hazard ratio, or odds ratio) of an association between a continuous or categorical dose for smoked tobacco consumption and a GBD outcome with uncertainty. Information on study design, confounders controlled for, sample representativeness, and measurement of exposure and outcomes was also extracted.

Second-hand smoke

We define secondhand smoke exposure as current exposure to secondhand tobacco smoke at home or at work. We use household composition as a proxy for household secondhand smoke exposure and make the assumption that all persons living with a daily smoker are exposed to tobacco smoke. We use surveys to estimate the proportion of the population exposed to secondhand smoke at work. We only consider non-smokers to be exposed to secondhand smoke. Non-smokers are defined as all persons who are not daily smokers. Ex-smokers and occasional smokers are considered non-smokers in this analysis. Exposure is evaluated for both children and adults.

To calculate the proportion of non-smokers who live with at least one daily smoker, two types of data were used: 1) unit record data on household composition, which included the ages and sexes of all persons living in the same household, and 2) GBD daily smoking estimates for each location, year, sex, and age group. Major survey series with a household composition module – including the Demographic Health Surveys (DHS), the Multiple Indicator Cluster Surveys (MICS), and the Living Standards Measurement Surveys (LSMS) – and national and subnational censuses, which included those captured in the Integrated Public Use Microdata Series (IPUMS) project, were used. To calculate the proportion of the population exposed to secondhand smoke at work, by age and sex, we used cross-sectional surveys that ask respondents about self-reported occupational secondhand smoke exposure. Sources include the Global Adult Tobacco Surveys (GATS), Eurobarometer Surveys, WHO Stepwise Approach to NCD Risk Factor Surveillance (STEPS) Surveys, and other regional and national survey series.

We updated our systematic review since GBD 2019 by searching the Global Health Data Exchange (GHDx) using the keywords "environmental tobacco smoke", for workplace exposure, and "family composition", for identifying household composition modules. We prioritised extraction of surveys used for estimating exposure at the workplace and of new household modules for filing in location and time gaps. Sources that reported exposure to secondhand smoke in a setting other than the workplace were not used. Due to the type of analysis performed, we restricted our data sources to those with available microdata (tabulated data-only sources were excluded). Given the nature of the data used in our models (microdata), no crosswalk for case definition adjustment or age and sex splitting processes were required.

Section 5.1.6. High body-mass index

High body-mass index (BMI) for adults (ages 20+) is defined as BMI greater than 20 to 23 kg/m^2. High BMI for children and adolescents (ages 2–19) is defined as being overweight or obese based on International Obesity Task Force standards.

Since GBD 2019, new data were added from sources included in the annual GHDx update of known survey series for our exposure models. We included representative studies providing data on mean BMI or prevalence of overweight or obesity among adults or children. For adults, studies were included if they defined overweight as BMI ≥25 kg/m^2 and obesity as BMI ≥30 kg/m^2, or if estimates using those cutoffs could be back-calculated from reported categories. For children (children ages 2–19), studies were included if they used International Obesity Task Force (IOTF) standards to define overweight and obesity thresholds.[1] We only included studies reporting data collected after January 1, 1980. Studies were excluded if they used non-random samples (eg, case-control studies or convenience samples), conducted among specific subpopulations (eg, pregnant women, racial or ethnic minorities, immigrants, or individuals with specific diseases), used alternative methods to assess adiposity (eg, waist circumference, skin-fold thickness, or hydrodensitometry), had sample sizes of less than 20 per age-sex group, or provided inadequate information on any of the inclusion criteria. We also excluded review articles and non-English-language articles.

Since GBD 2019, we did not conduct an updated systematic review to identify new relative risk data sources.

Section 6. References

1. GBD 2019 Diseases and Injuries Collaborators. Global burden of 369 diseases and injuries in 204 countries and territories, 1990-2019: a systematic analysis for the Global Burden of Disease Study 2019. *Lancet* 2020; **396**(10258): 1204-22.
2. Stevens GA, Alkema L, Black RE, et al. Guidelines for accurate and transparent health estimates reporting: the GATHER statement. *PLoS Med* 2016; **13**(6): e1002056.
3. Zheng P, Barber, R., Sorensen, R. J., Murray, C. J., & Aravkin, A. Y. . Trimmed constrained mixed effects models: formulations and algorithms. *J Comput Graph Stat* 2021: 1-13.
4. Moore TJ, Barron JE, Hutchinson 3rd F, Golden CA, Ellis CL, Humphries DA. Prosthetic usage following major lower extremity amputation. *Clinical orthopaedics and related research*. 1989 Jan 1(238):219-24.
5. Salomon JA, Haagsma JA, Davis A, et al. Disability weights for the Global Burden of Disease 2013 study. *Lancet Glob Health* 2015; **3**(11): e712-23.
6. Zheng P, Afshin A, Biryukov S, et al. The Burden of Proof studies: assessing the evidence of risk. *Nat Med* 2022; **28**(10): 2038-44.
7. GBD 2019 Risk Factors Collaborators. Global burden of 87 risk factors in 204 countries and territories, 1990-2019: a systematic analysis for the Global Burden of Disease Study 2019. *Lancet* 2020; **396**(10258): 1223-49.
8. Lescinsky H, Afshin A, Ashbaugh C, Bisignano C, Brauer M, Ferrara G, Hay SI, He J, Iannucci V, Marczak LB, McLaughlin SA. Health effects associated with consumption of unprocessed red meat: a Burden of Proof study. *Nature Medicine*. 2022 Oct;**28**(10):2075-82.
9. Stanaway JD, Afshin A, Ashbaugh C, Bisignano C, Brauer M, Ferrara G, Garcia V, Haile D, Hay SI, He J, Iannucci V. Health effects associated with vegetable consumption: a Burden of Proof study. *Nature Medicine*. 2022 Oct;**28**(10):2066-74.
10. Dai X, Gil GF, Reitsma MB, Ahmad NS, Anderson JA, Bisignano C, Carr S, Feldman R, Hay SI, He J, Iannucci V. Health effects associated with smoking: a Burden of Proof study. *Nature Medicine*. 2022 Oct;**28**(10):2045-55.
11. International Diabetes Federation. IDF Diabetes Atlas, 10th edition. Brussels, Belgium, 2021.
12. NCD Risk Factor Collaboration. Worldwide trends in diabetes since 1980: a pooled analysis of 751 population-based studies with 4.4 million participants. *Lancet* 2016; **387**(10027): 1513-30.

Section 7. Additional figures and tables

Figure S20. Total diabetes age-standardised prevalence for both sexes combined in 1990 (A) and 2050 (B)

A.

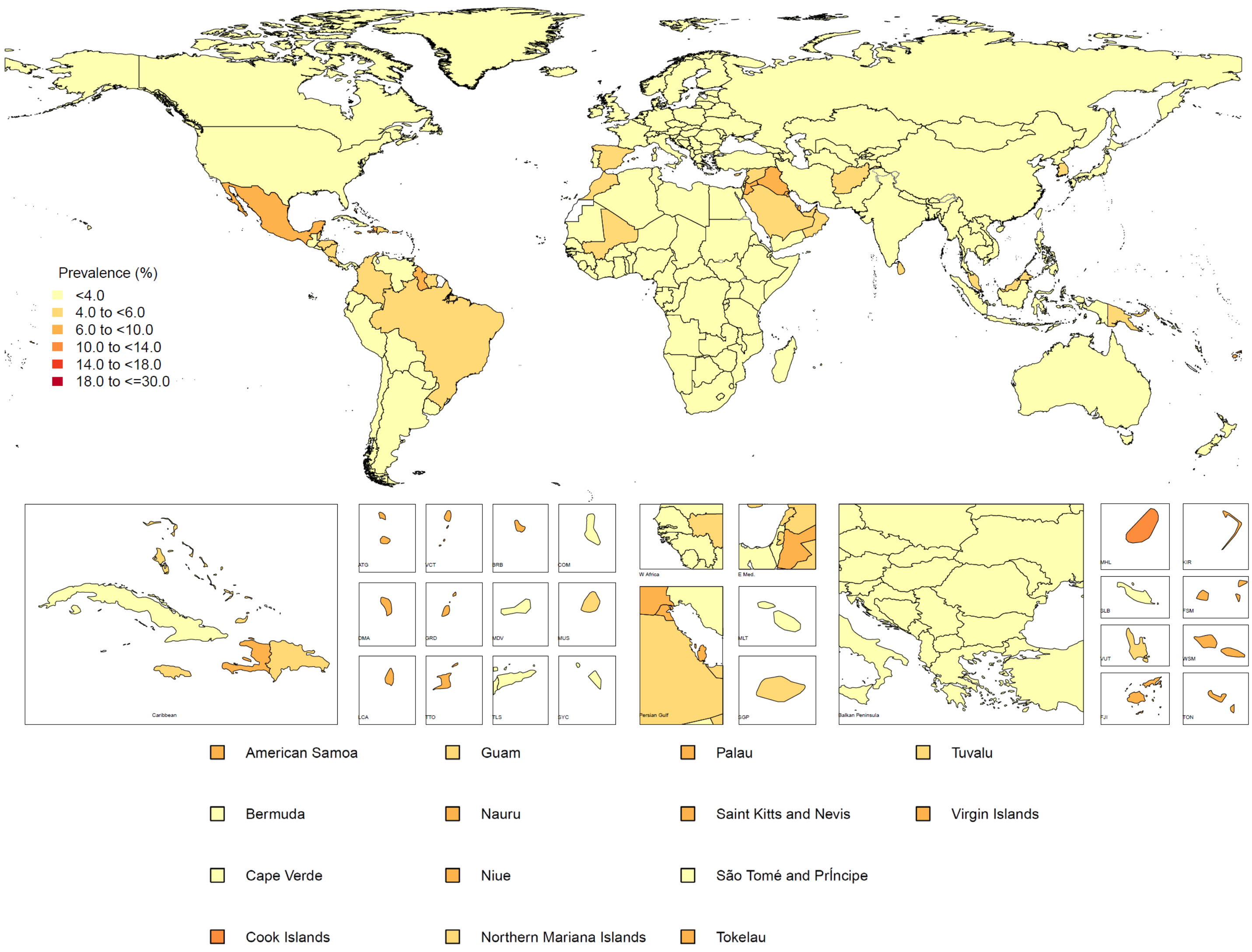

B.

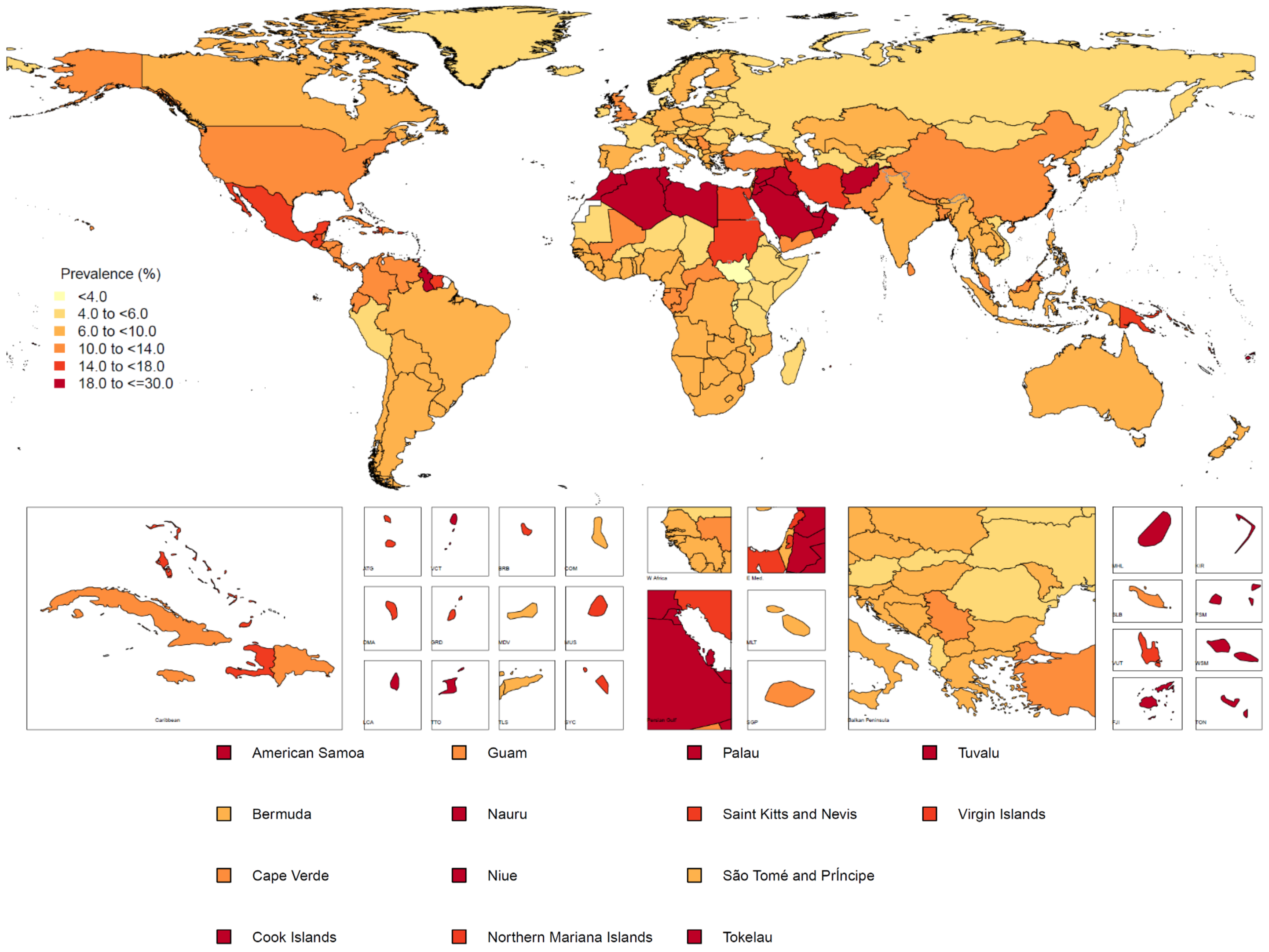
Prevalence (%)
<4.0
4.0 to <6.0
6.0 to <10.0
10.0 to <14.0
14.0 to <18.0
18.0 to <=30.0
American Samoa
Guam
Palau
Tuvalu
Bermuda
Nauru
Saint Kitts and Nevis
Virgin Islands
Cape Verde
Niue
São Tomé and Prĺncipe
Cook Islands
Northern Mariana Islands
Tokelau

Figure S21. Type 1 diabetes age-standardised prevalence for both sexes combined in 2021 (A) and 2050 (B)

A.

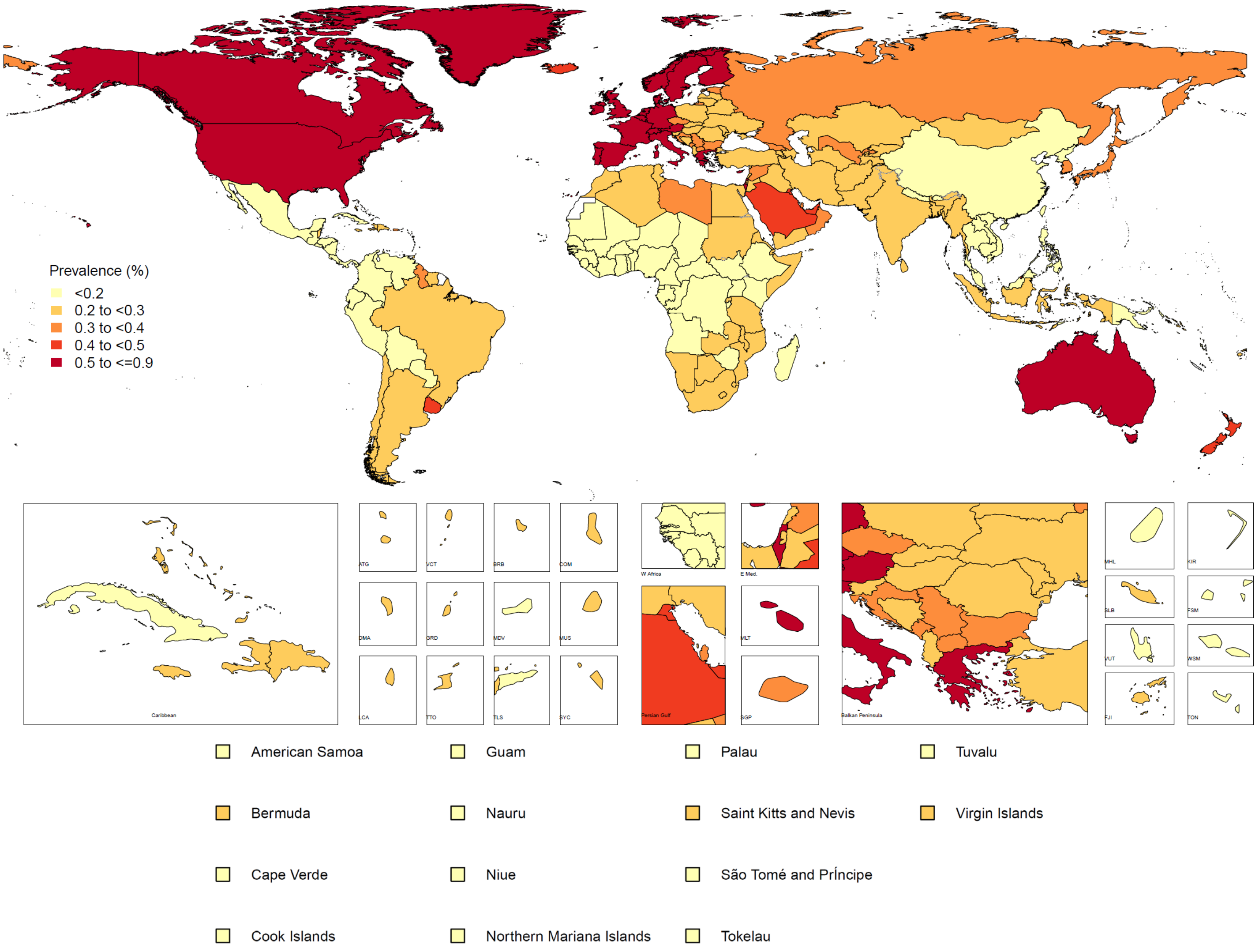

B.

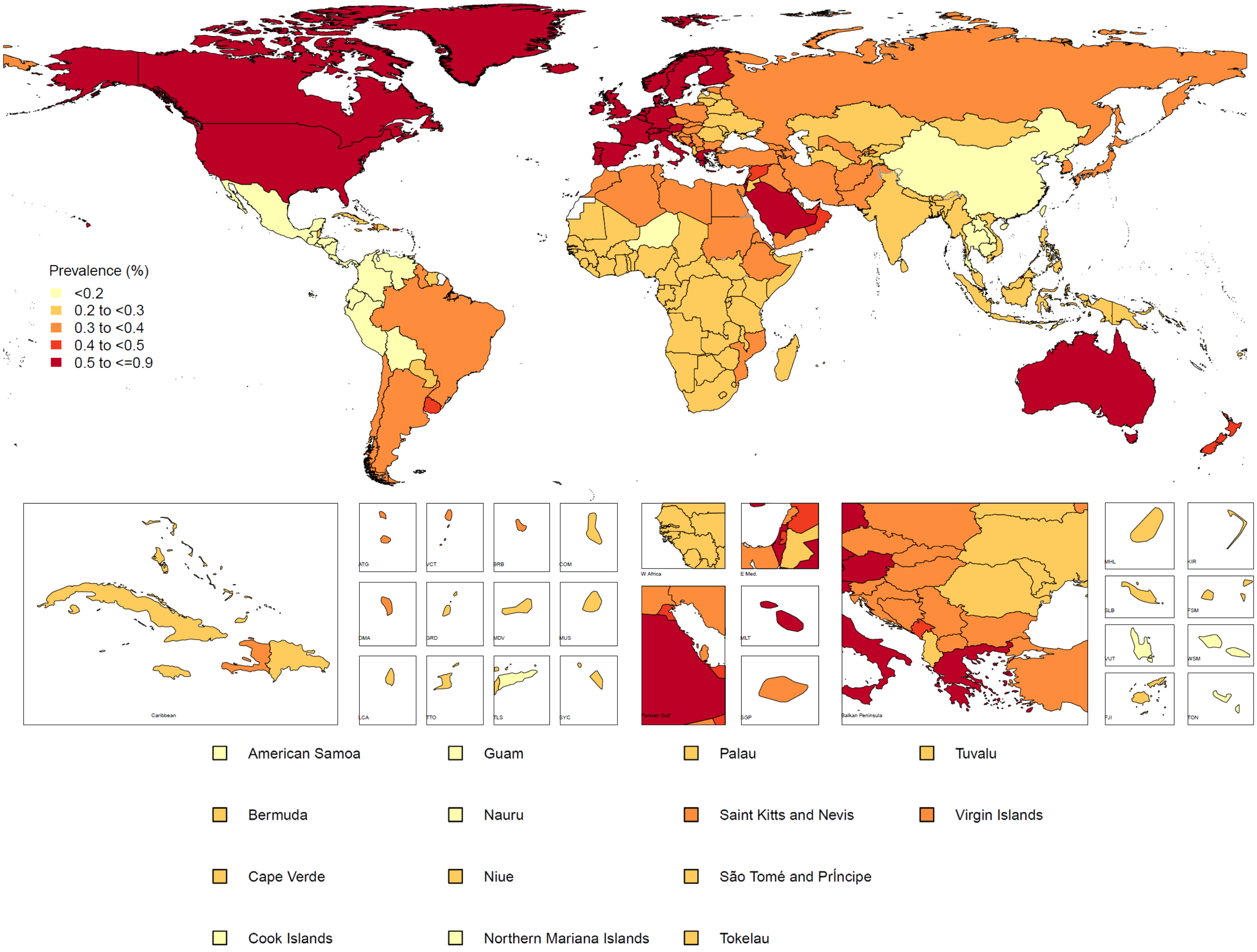
Prevalence (%)
<0.2
0.2 to <0.3
0.3 to <0.4
0.4 to <0.5
0.5 to <=0.9
Caribbean
ATG
VCT
BRB
COM
DMA
GRD
MDV
MUS
LCA
TTO
TLS
SYC
W Africa
E Med.
Persian Gulf
MLT
SGP
Balkan Peninsula
MHL
KIR
SLB
FSM
VUT
WSM
FJI
TON
American Samoa
Guam
Palau
Tuvalu
Bermuda
Nauru
Saint Kitts and Nevis
Virgin Islands
Cape Verde
Niue
São Tomé and Prĺncipe
Cook Islands
Northern Mariana Islands
Tokelau

Figure S22. Type 2 diabetes age-standardised prevalence for both sexes combined in 2021 (A) and 2050 (B)

A.

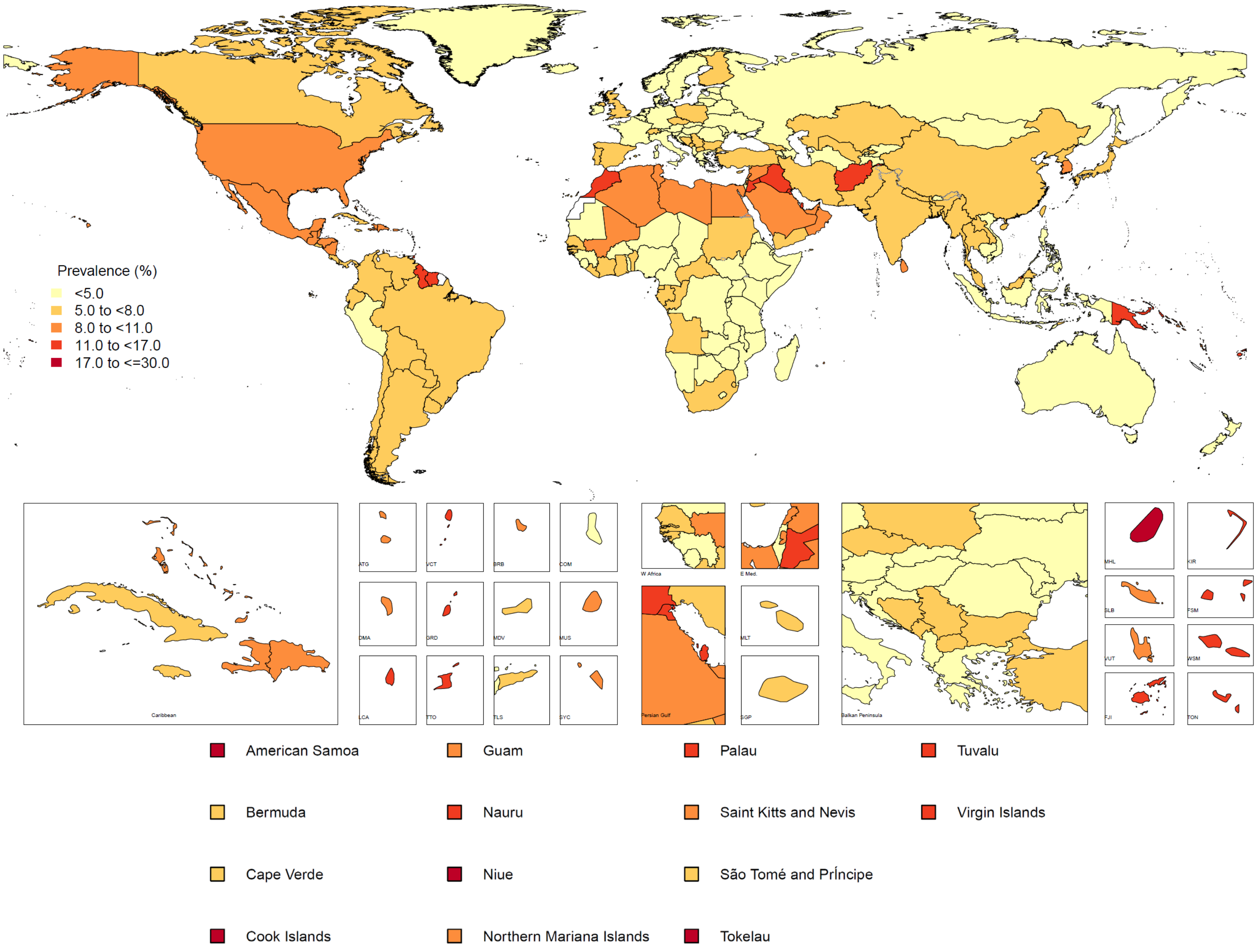

B.

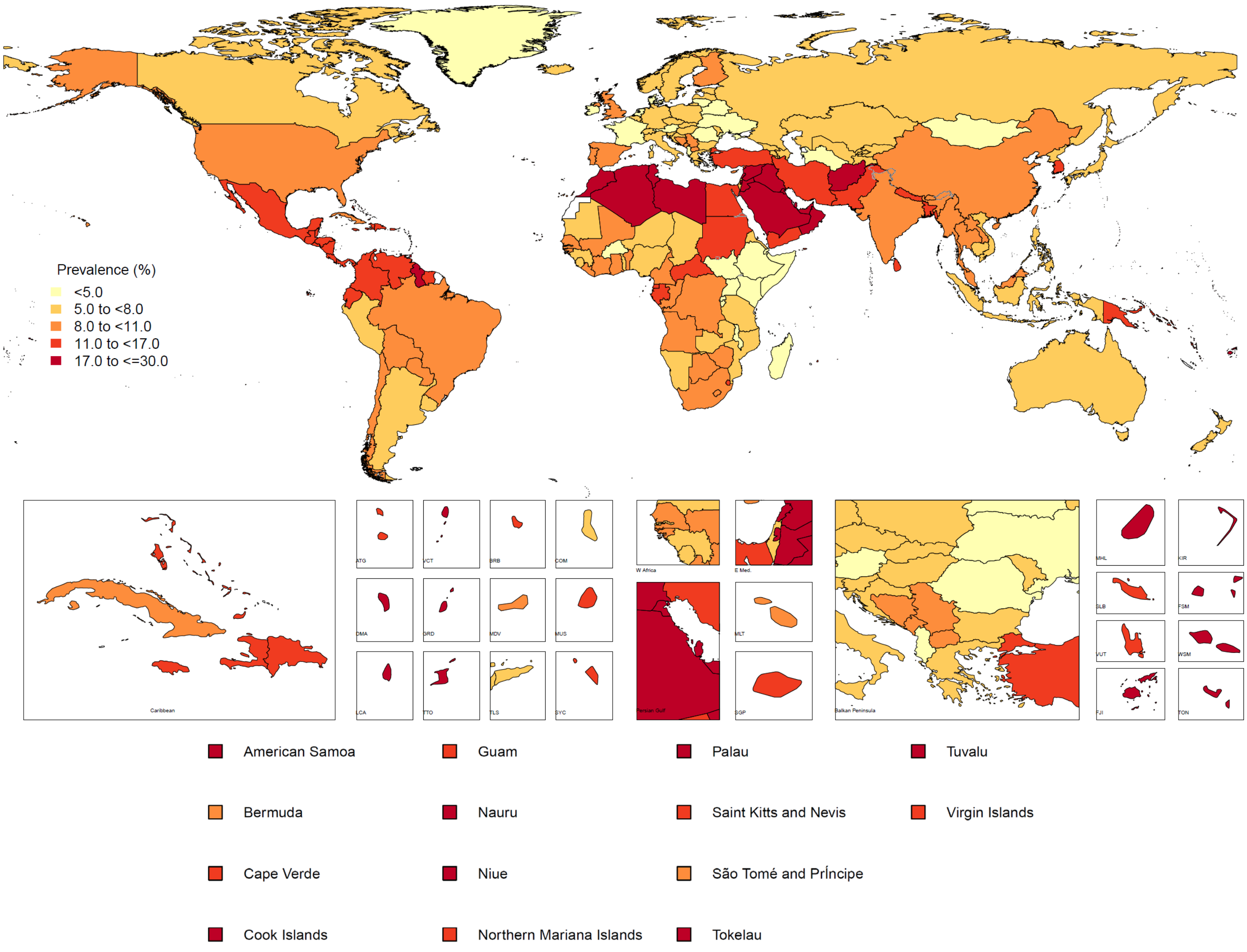
Prevalence (%)
<5.0
5.0 to <8.0
8.0 to <11.0
11.0 to <17.0
17.0 to <=30.0
Caribbean
ATG
VCT
BRB
COM
DMA
GRD
MDV
MUS
LCA
TTO
TLS
SYC
W Africa
E Med.
Persian Gulf
MLT
SGP
Balkan Peninsula
MHL
KIR
SLB
FSM
VUT
WSM
FJI
TON
American Samoa
Guam
Palau
Tuvalu
Bermuda
Nauru
Saint Kitts and Nevis
Virgin Islands
Cape Verde
Niue
São Tomé and Príncipe
Cook Islands
Northern Mariana Islands
Tokelau

Figure S23. Total diabetes age-specific prevalence by sex in 2021 by super-region, region, and country

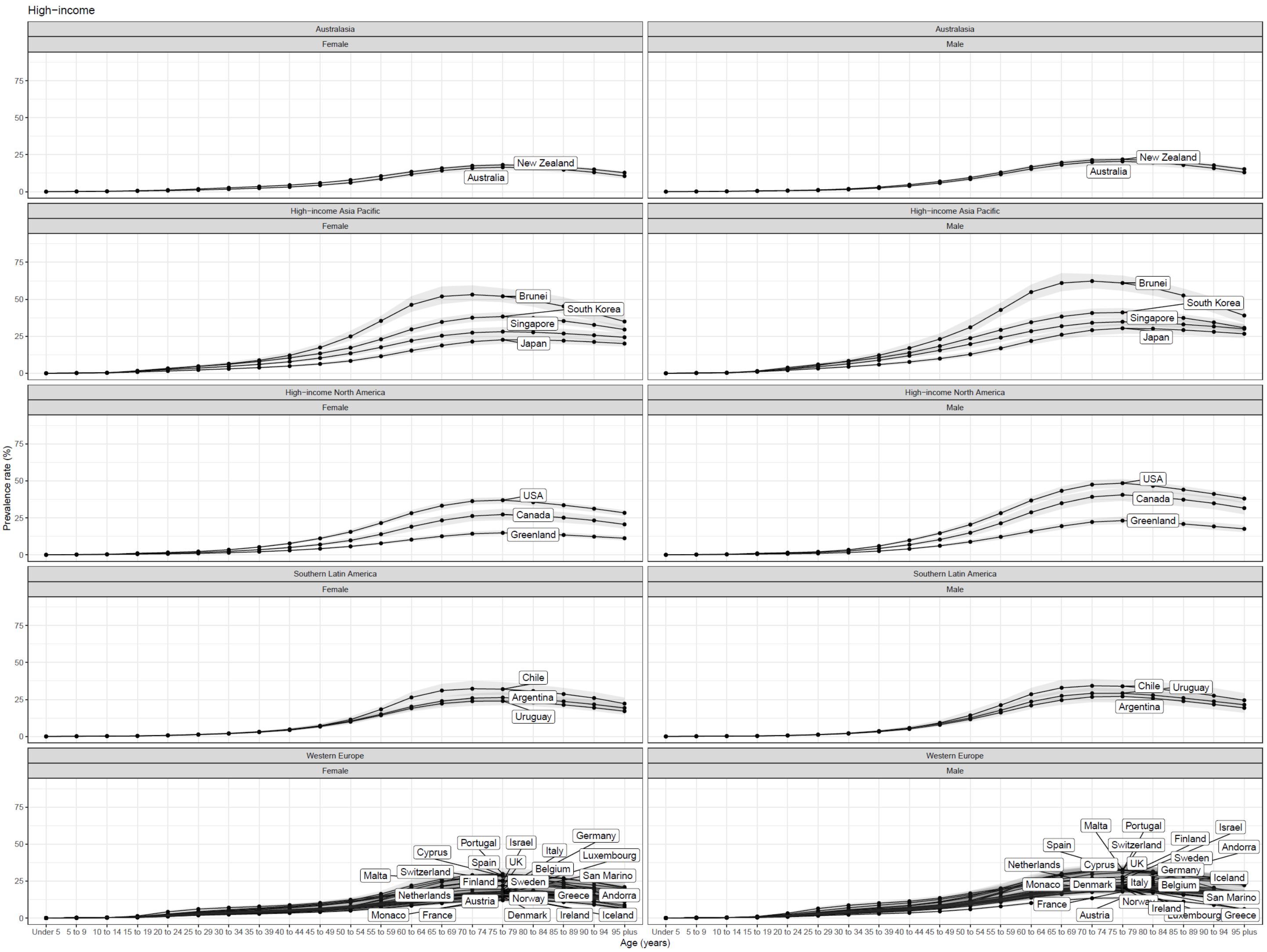

Central Europe, Eastern Europe, and Central Asia

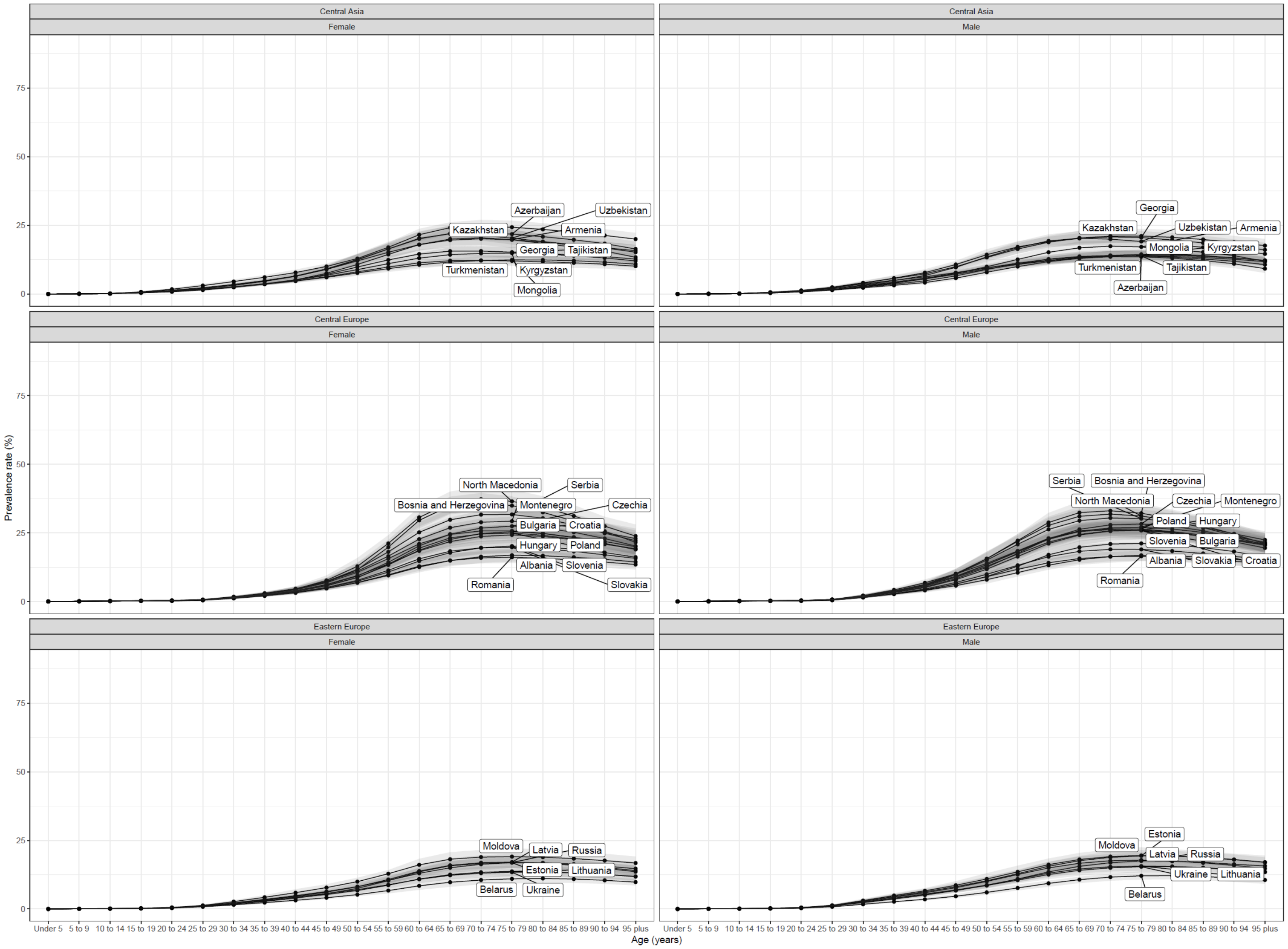

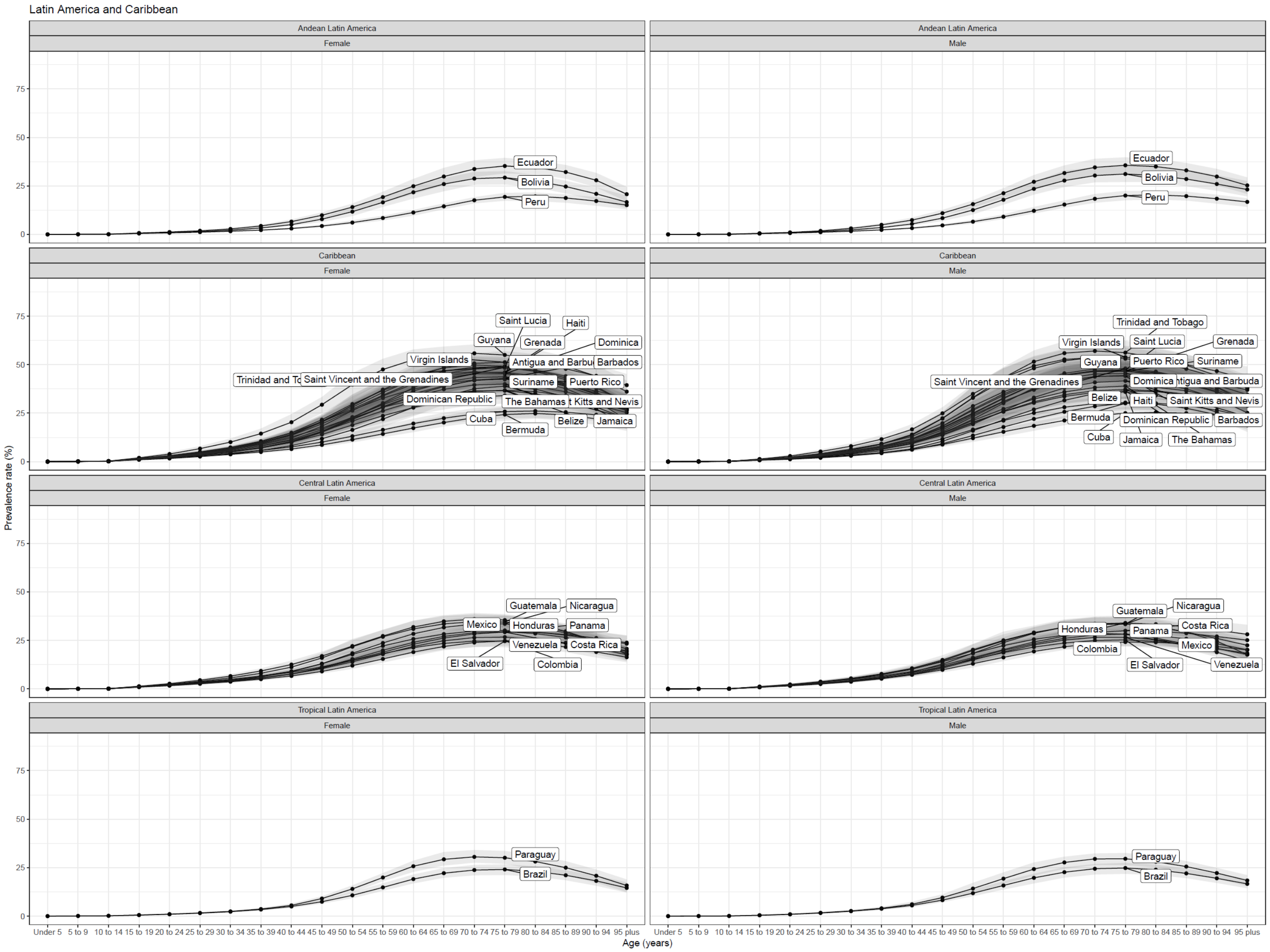

Latin America and Caribbean
Andean Latin America
Female
Male
Ecuador
Bolivia
Peru
Caribbean
Saint Lucia
Haiti
Guyana
Grenada
Dominica
Virgin Islands
Antigua and Barbu
Barbados
Trinidad and To
Saint Vincent and the Grenadines
Suriname
Puerto Rico
Dominican Republic
The Bahamas
t Kitts and Nevis
Cuba
Belize
Jamaica
Bermuda
Trinidad and Tobago
tigua and Barbuda
Saint Kitts and Nevis
Central Latin America
Guatemala
Nicaragua
Mexico
Honduras
Panama
Venezuela
Costa Rica
El Salvador
Colombia
Tropical Latin America
Paraguay
Brazil
Prevalence rate (%)
Age (years)

North Africa and Middle East

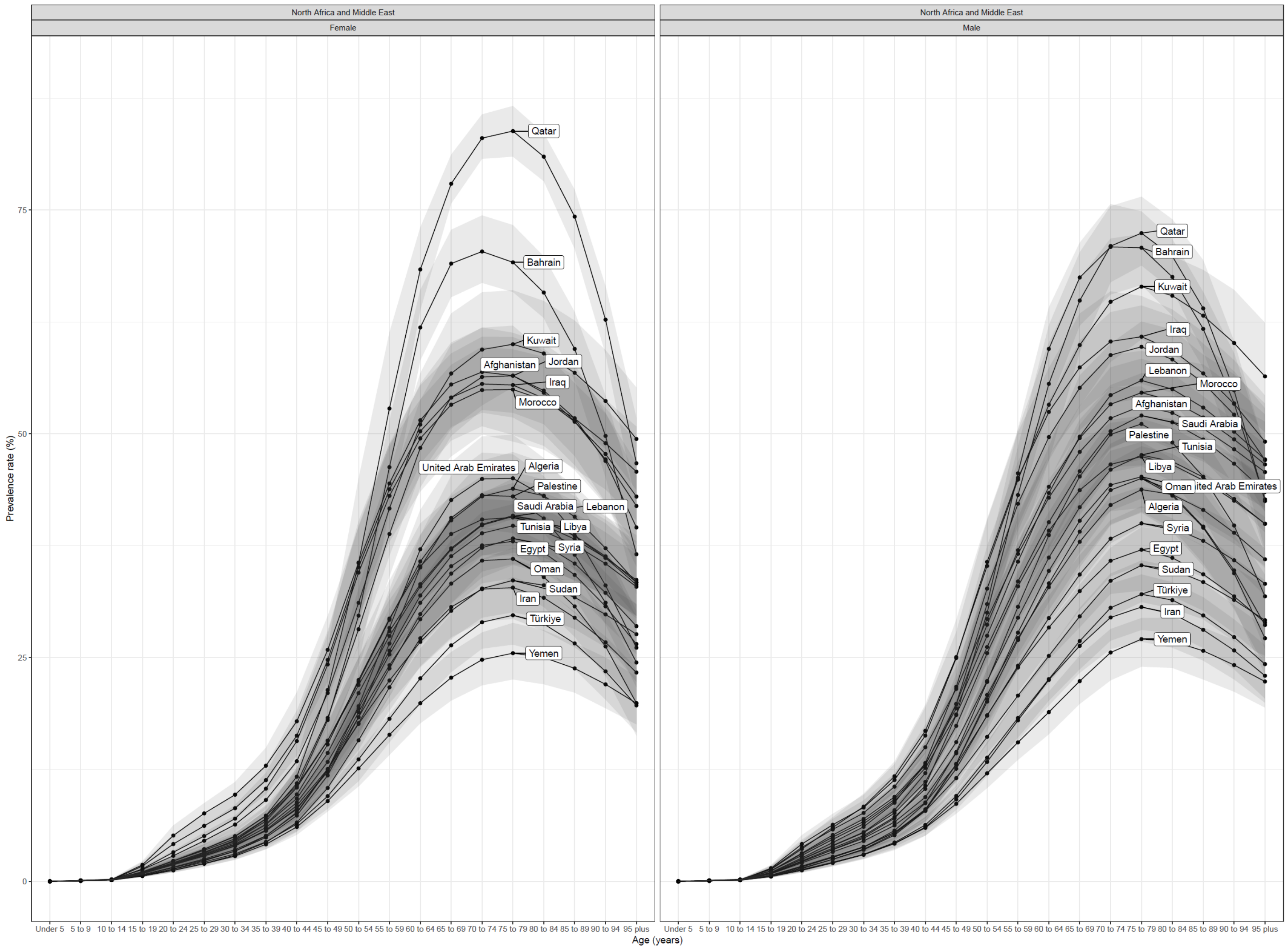

Southeast Asia, East Asia, and Oceania

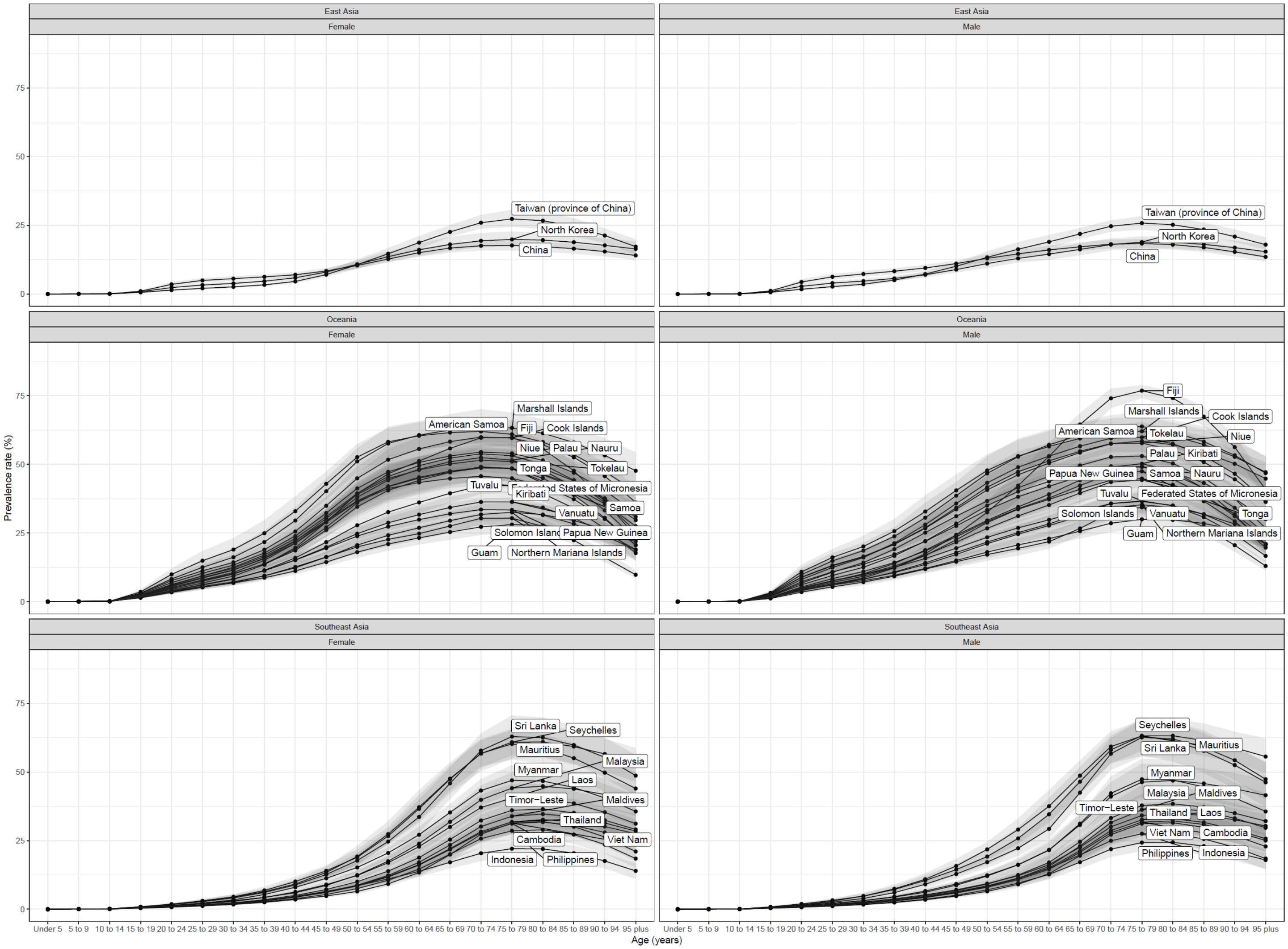

South Asia

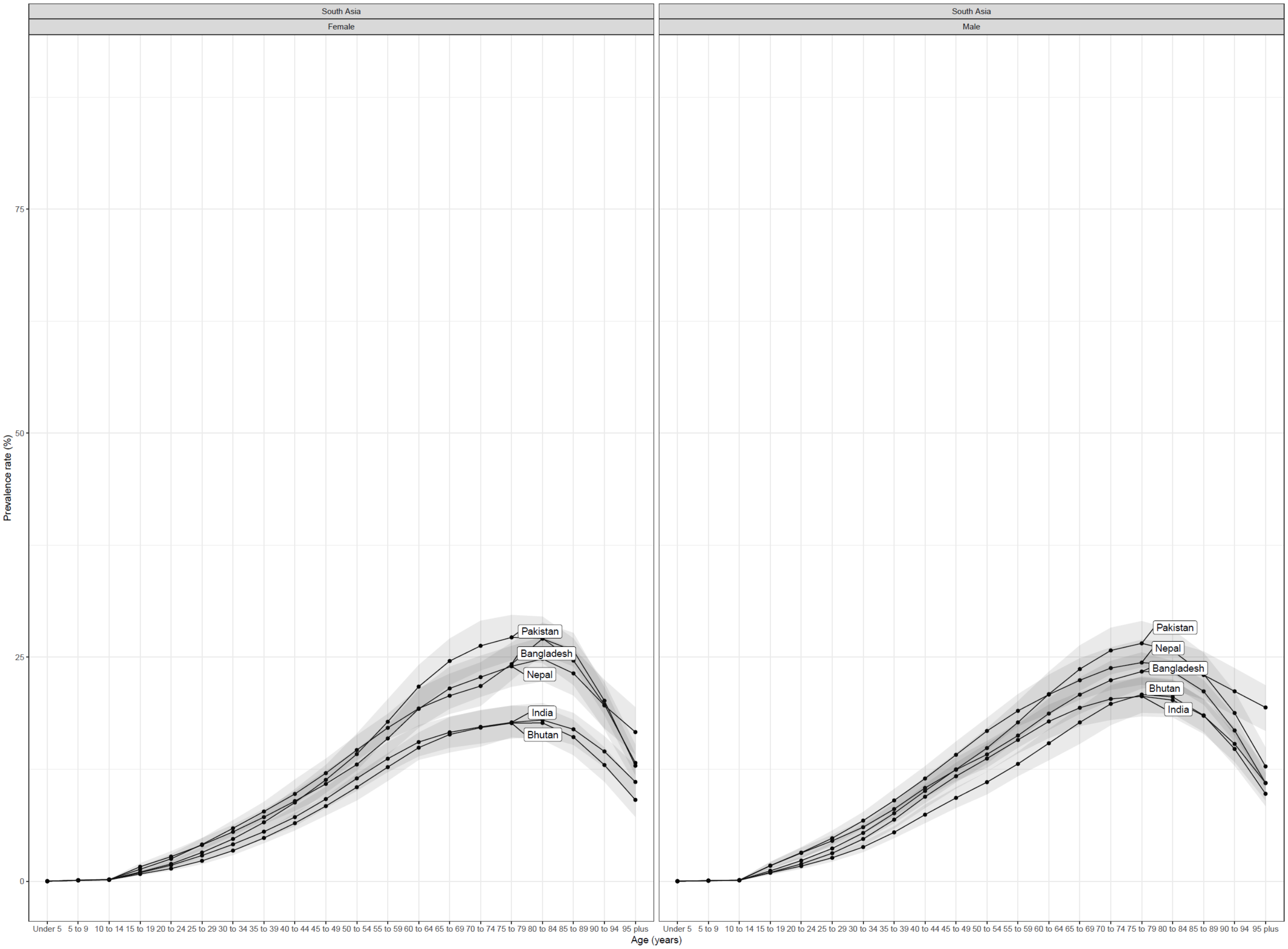
South Asia
Female
South Asia
Male
Prevalence rate (%)
75
50
25
0
Pakistan
Bangladesh
Nepal
India
Bhutan
Pakistan
Nepal
Bangladesh
Bhutan
India
Under 5
5 to 9
10 to 14
15 to 19
20 to 24
25 to 29
30 to 34
35 to 39
40 to 44
45 to 49
50 to 54
55 to 59
60 to 64
65 to 69
70 to 74
75 to 79
80 to 84
85 to 89
90 to 94
95 plus
Age (years)

Sub−Saharan Africa

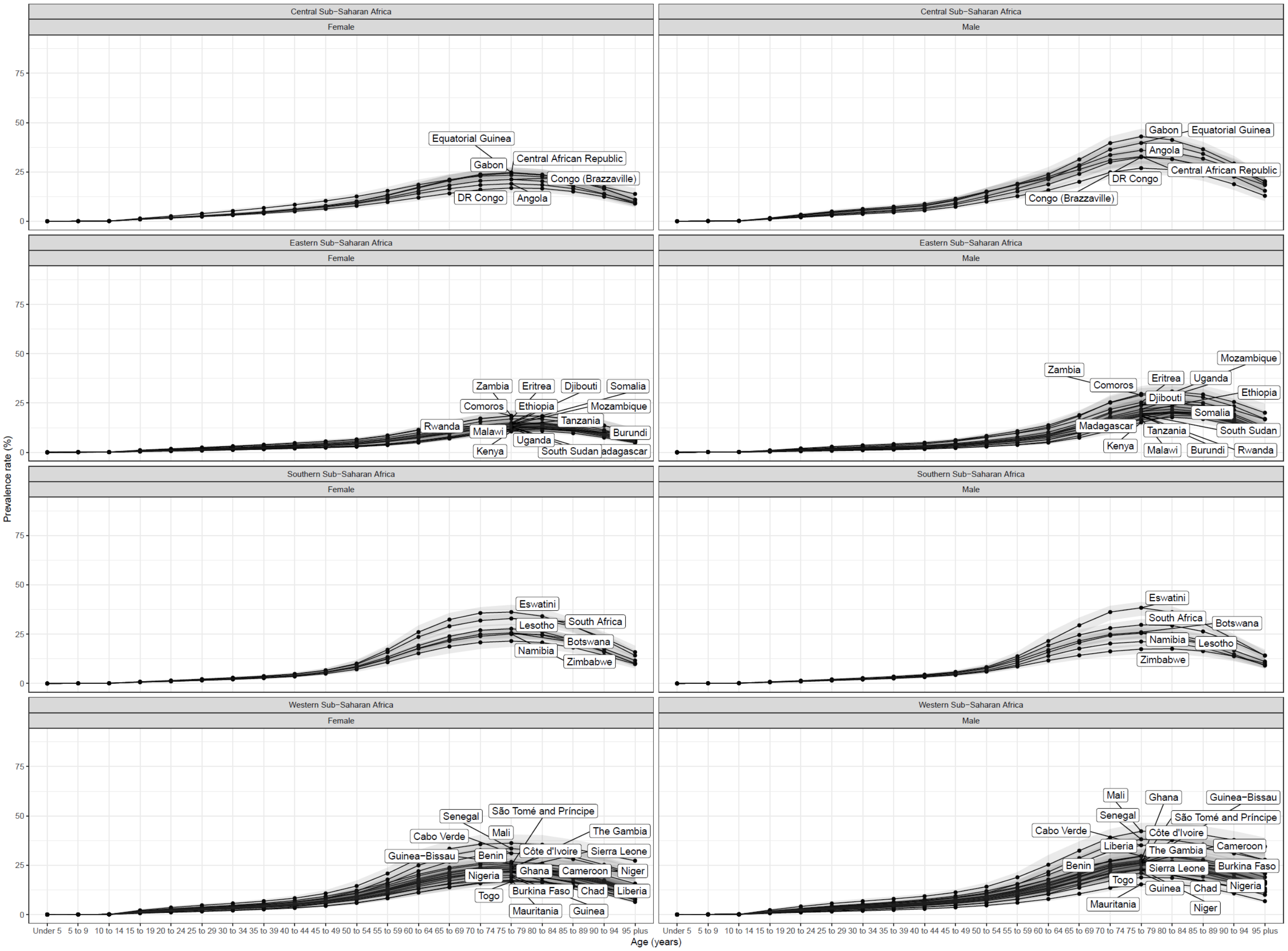

Figure S24. Sex ratio (males-to-females) of age-standardised total diabetes prevalence in 204 locations by GBD region in 2021

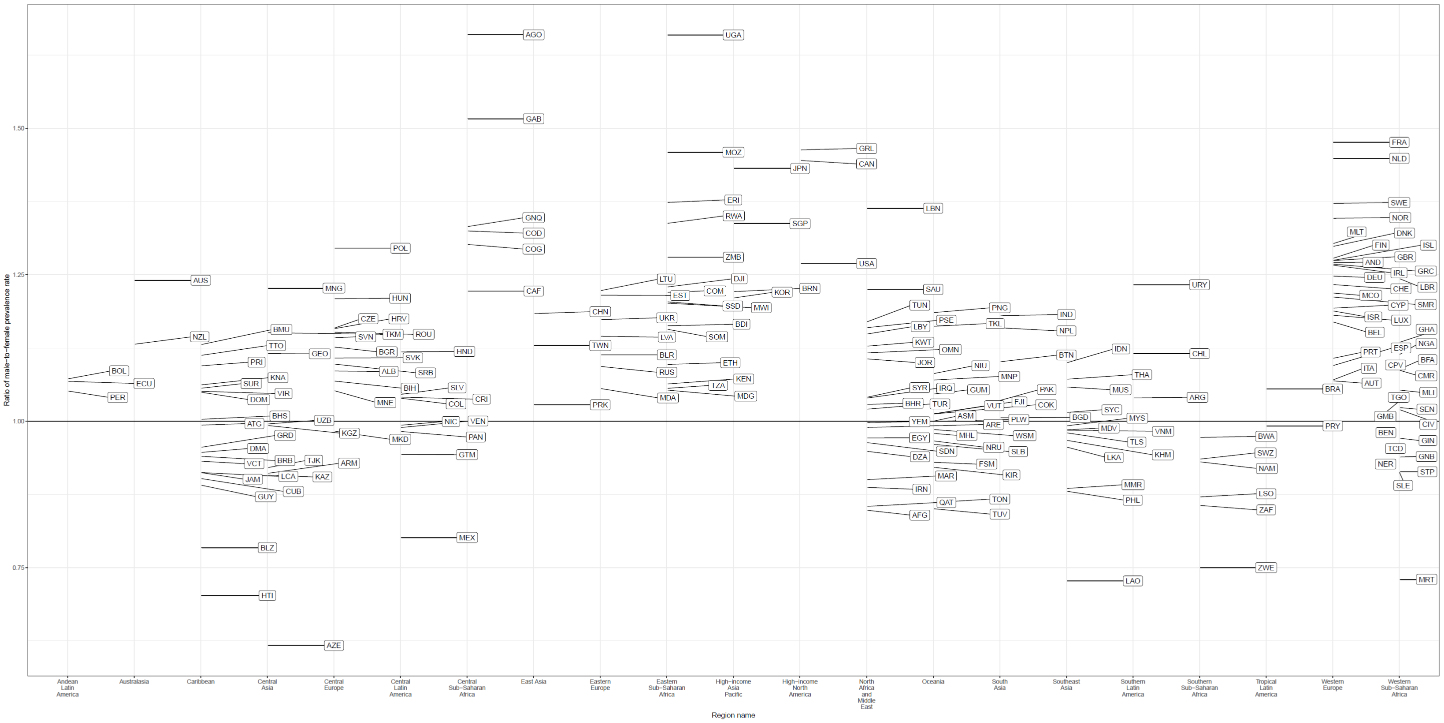

Figure S25. Change from 1990 to 2021 in population attributable fraction for five risk factor groups in relation to type 2 diabetes

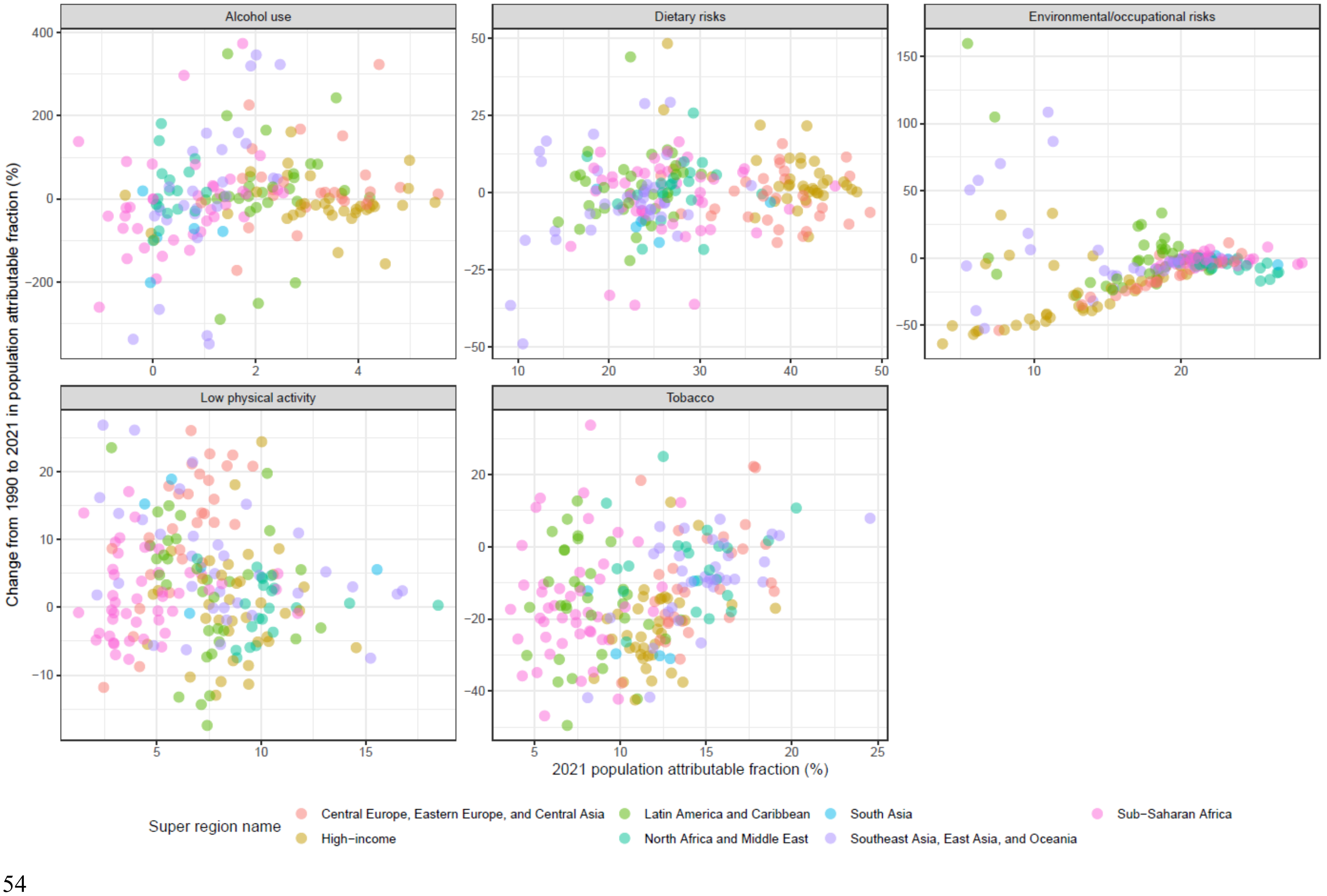

Figure S26. Global number of people with type 1 diabetes and type 2 diabetes from 1990 through 2050 forecasts

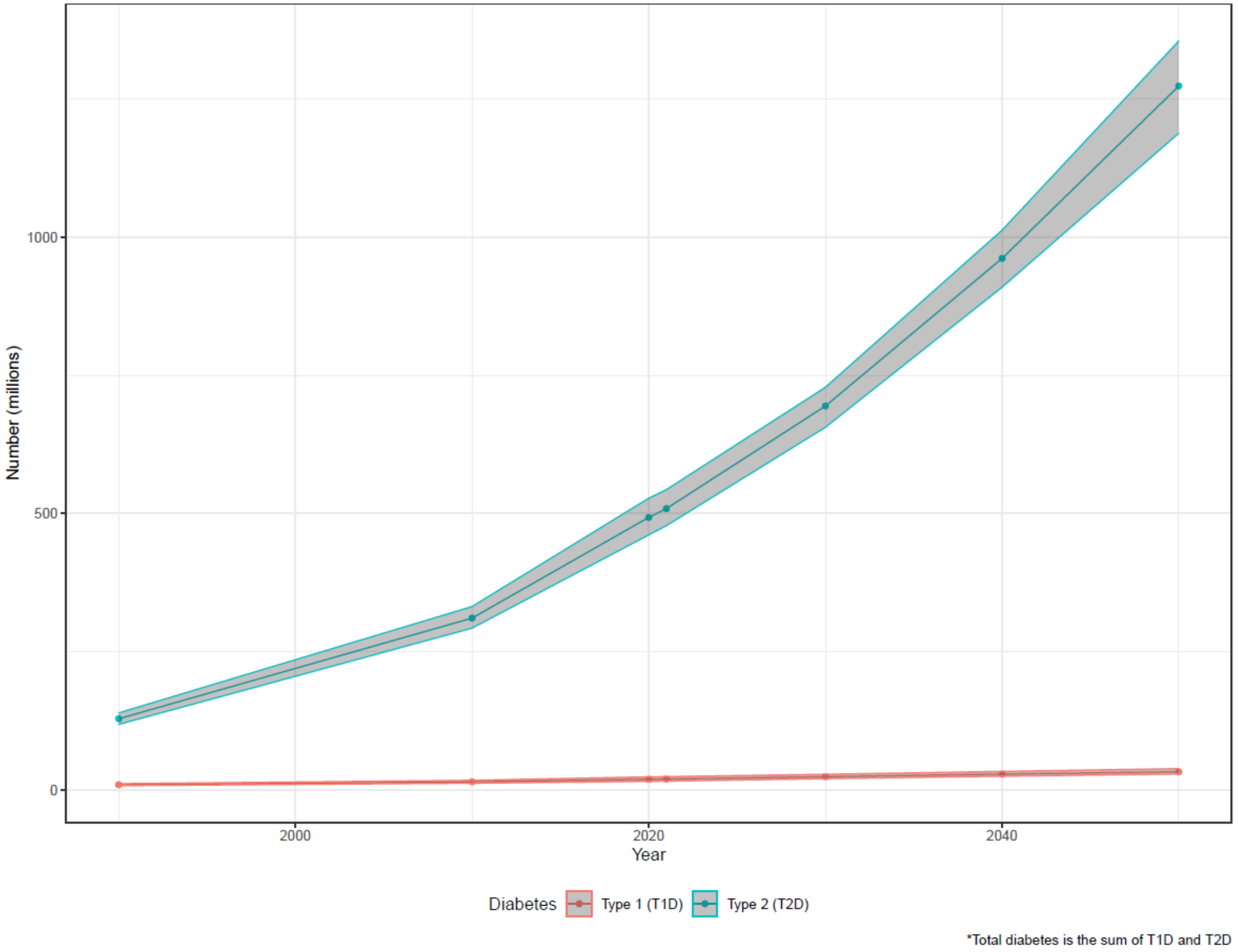

Table S18. Global Burden of Disease location hierarchy

Location	Level
**Global**	**0**
**Low SDI**	**1**
**Low-middle SDI**	**1**
**Middle SDI**	**1**
**High-middle SDI**	**1**
**High SDI**	**1**
**Central Europe, Eastern Europe, and Central Asia**	**1**
**Central Asia**	**2**

Armenia	3
Azerbaijan	3
Georgia	3
Kazakhstan	3
Kyrgyzstan	3
Mongolia	3
Tajikistan	3
Turkmenistan	3
Uzbekistan	3
**Central Europe**	**2**
Albania	3
Bosnia and Herzegovina	3
Bulgaria	3
Croatia	3
Czechia	3
Hungary	3
Montenegro	3
North Macedonia	3
Poland	3
Romania	3
Serbia	3
Slovakia	3
Slovenia	3
**Eastern Europe**	**2**
Belarus	3
Estonia	3
Latvia	3

Lithuania	3
Moldova	3
Russia	3
Ukraine	3
**High income**	**1**
**Australasia**	**2**
Australia	3
New Zealand	3
**High-income Asia Pacific**	**2**
Brunei	3
Japan	3
Singapore	3
South Korea	3
**High-income North America**	**2**
Canada	3
Greenland	3
USA	3
**Southern Latin America**	**2**
Argentina	3
Chile	3
Uruguay	3
**Western Europe**	**2**
Andorra	3
Austria	3
Belgium	3
Cyprus	3
Denmark	3

Finland	3
France	3
Germany	3
Greece	3
Iceland	3
Ireland	3
Israel	3
Italy	3
Luxembourg	3
Malta	3
Monaco	3
Netherlands	3
Norway	3
Portugal	3
San Marino	3
Spain	3
Sweden	3
Switzerland	3
UK	3
**Latin America and Caribbean**	**1**
**Andean Latin America**	**2**
Bolivia	3
Ecuador	3
Peru	3
**Caribbean**	**2**
Antigua and Barbuda	3
Barbados	3

Belize	3
Bermuda	3
The Bahamas	3
Cuba	3
Dominica	3
Dominican Republic	3
Grenada	3
Guyana	3
Haiti	3
Jamaica	3
Puerto Rico	3
Saint Kitts and Nevis	3
Saint Lucia	3
Saint Vincent and the Grenadines	3
Suriname	3
Trinidad and Tobago	3
Virgin Islands	3
**Central Latin America**	**2**
Venezuela	3
Colombia	3
Costa Rica	3
El Salvador	3
Guatemala	3
Honduras	3
Mexico	3
Nicaragua	3
Panama	3

**Tropical Latin America**	**2**
Brazil	3
Paraguay	3
**North Africa and Middle East**	**1**
**North Africa and Middle East**	**2**
Afghanistan	3
Algeria	3
Bahrain	3
Egypt	3
Iraq	3
Iran	3
Jordan	3
Kuwait	3
Lebanon	3
Libya	3
Morocco	3
Palestine	3
Oman	3
Qatar	3
Saudi Arabia	3
Sudan	3
Syria	3
Tunisia	3
Türkiye	3
United Arab Emirates	3
Yemen	3
**South Asia**	**1**

**South Asia**	**2**
Bangladesh	3
Bhutan	3
India	3
Nepal	3
Pakistan	3
**Southeast Asia, East Asia, and Oceania**	**1**
**East Asia**	**2**
China	3
North Korea	3
Taiwan (Province of China)	3
**Oceania**	**2**
American Samoa	3
Cook Islands	3
Federated States of Micronesia	3
Fiji	3
Guam	3
Kiribati	3
Marshall Islands	3
Nauru	3
Niue	3
Northern Mariana Islands	3
Palau	3
Papua New Guinea	3
Samoa	3
Solomon Islands	3
Tokelau	3

Tonga	3
Tuvalu	3
Vanuatu	3
**Southeast Asia**	**2**
Cambodia	3
Indonesia	3
Laos	3
Malaysia	3
Maldives	3
Mauritius	3
Myanmar	3
Philippines	3
Sri Lanka	3
Seychelles	3
Thailand	3
Timor-Leste	3
Vietnam	3
**Sub-Saharan Africa**	**1**
**Central sub-Saharan Africa**	**2**
Angola	3
Central African Republic	3
Congo (Brazzaville)	3
DR Congo	3
Equatorial Guinea	3
Gabon	3
**Eastern sub-Saharan Africa**	**2**
Burundi	3

Comoros	3
Djibouti	3
Eritrea	3
Ethiopia	3
Kenya	3
Madagascar	3
Malawi	3
Mozambique	3
Rwanda	3
Somalia	3
South Sudan	3
Tanzania	3
Uganda	3
Zambia	3
**Southern sub-Saharan Africa**	**2**
Botswana	3
Eswatini	3
Lesotho	3
Namibia	3
South Africa	3
Zimbabwe	3
**Western sub-Saharan Africa**	**2**
Benin	3
Burkina Faso	3
Cameroon	3
Cape Verde	3
Chad	3

Côte d'Ivoire	3
The Gambia	3
Ghana	3
Guinea	3
Guinea-Bissau	3
Liberia	3
Mali	3
Mauritania	3
Niger	3
Nigeria	3
São Tomé and Príncipe	3
Senegal	3
Sierra Leone	3
Togo	3

Table S19. Countries estimated by the Global Burden of Diseases, Injuries, and Risk Factors Study (GBD), the International Diabetes Federation (IDF)[11], and the NCD Risk Factor Collaboration (NCD-RisC)[12]

**Country**	**GBD**	**IDF**	**NCD-RisC**
Afghanistan	Yes	Yes	Yes
Albania	Yes	Yes	Yes
Algeria	Yes	Yes	Yes
American Samoa	Yes	Yes	Yes
Andorra	Yes	Yes	Yes
Angola	Yes	Yes	Yes
Antigua and Barbuda	Yes	Yes	Yes
Argentina	Yes	Yes	Yes
Armenia	Yes	Yes	Yes
**Aruba**	**No**	**Yes**	**No**
Australia	Yes	Yes	Yes
Austria	Yes	Yes	Yes
Azerbaijan	Yes	Yes	Yes
Bahrain	Yes	Yes	Yes
Bangladesh	Yes	Yes	Yes
Barbados	Yes	Yes	Yes
Belarus	Yes	Yes	Yes
Belgium	Yes	Yes	Yes

Belize	Yes	Yes	Yes
Benin	Yes	Yes	Yes
Bermuda	Yes	Yes	Yes
Bhutan	Yes	Yes	Yes
Bolivia	Yes	Yes	Yes
Bosnia and Herzegovina	Yes	Yes	Yes
Botswana	Yes	Yes	Yes
Brazil	Yes	Yes	Yes
**British Virgin Islands**	**No**	**Yes**	**No**
Brunei	Yes	Yes	Yes
Bulgaria	Yes	Yes	Yes
Burkina Faso	Yes	Yes	Yes
Burundi	Yes	Yes	Yes
Cambodia	Yes	Yes	Yes
Cameroon	Yes	Yes	Yes
Canada	Yes	Yes	Yes
Cape Verde	Yes	Yes	Yes
**Cayman Islands**	**No**	**Yes**	**No**
Central African Republic	Yes	Yes	Yes
Chad	Yes	Yes	Yes
**Channel Islands**	**No**	**Yes**	**No**
Chile	Yes	Yes	Yes
China	Yes	Yes	Yes
Colombia	Yes	Yes	Yes
Comoros	Yes	Yes	Yes
Congo (Brazzaville)	Yes	Yes	Yes
**Cook Islands**	**Yes**	**No**	**Yes**
Costa Rica	Yes	Yes	Yes
Côte d'Ivoire	Yes	Yes	Yes
Croatia	Yes	Yes	Yes
Cuba	Yes	Yes	Yes
**Curaçao**	**No**	**Yes**	**No**
Cyprus	Yes	Yes	Yes
Czechia	Yes	Yes	Yes
Denmark	Yes	Yes	Yes
Djibouti	Yes	Yes	Yes
Dominica	Yes	Yes	Yes
Dominican Republic	Yes	Yes	Yes
DR Congo	Yes	Yes	Yes
Ecuador	Yes	Yes	Yes
Egypt	Yes	Yes	Yes
El Salvador	Yes	Yes	Yes
Equatorial Guinea	Yes	Yes	Yes
Eritrea	Yes	Yes	Yes
Estonia	Yes	Yes	Yes
Eswatini	Yes	Yes	Yes

Ethiopia	Yes	Yes	Yes
**Faroe Islands**	**No**	**Yes**	**No**
Federated States of Micronesia	Yes	Yes	Yes
Fiji	Yes	Yes	Yes
Finland	Yes	Yes	Yes
France	Yes	Yes	Yes
**French Polynesia**	**No**	**Yes**	**Yes**
Gabon	Yes	Yes	Yes
Georgia	Yes	Yes	Yes
Germany	Yes	Yes	Yes
Ghana	Yes	Yes	Yes
Greece	Yes	Yes	Yes
Greenland	Yes	Yes	Yes
Grenada	Yes	Yes	Yes
**Guam**	**Yes**	**Yes**	**No**
Guatemala	Yes	Yes	Yes
Guinea	Yes	Yes	Yes
Guinea-Bissau	Yes	Yes	Yes
Guyana	Yes	Yes	Yes
Haiti	Yes	Yes	Yes
Honduras	Yes	Yes	Yes
**Holy See**	**No**	**Yes**	**No**
**Hong Kong Special Administrative Region of China**	**No**	**Yes**	**Yes**
Hungary	Yes	Yes	Yes
Iceland	Yes	Yes	Yes
India	Yes	Yes	Yes
Indonesia	Yes	Yes	Yes
Iran	Yes	Yes	Yes
Iraq	Yes	Yes	Yes
Ireland	Yes	Yes	Yes
**Isle of Man**	**No**	**Yes**	**No**
Israel	Yes	Yes	Yes
Italy	Yes	Yes	Yes
Jamaica	Yes	Yes	Yes
Japan	Yes	Yes	Yes
Jordan	Yes	Yes	Yes
Kazakhstan	Yes	Yes	Yes
Kenya	Yes	Yes	Yes
Kiribati	Yes	Yes	Yes
Kuwait	Yes	Yes	Yes
Kyrgyzstan	Yes	Yes	Yes
Laos	Yes	Yes	Yes
Latvia	Yes	Yes	Yes
Lebanon	Yes	Yes	Yes
Lesotho	Yes	Yes	Yes
Liberia	Yes	Yes	Yes

Libya	Yes	Yes	Yes
**Liechtenstein**	**No**	**Yes**	**No**
Lithuania	Yes	Yes	Yes
Luxembourg	Yes	Yes	Yes
**Macao Special Administrative Region of China**	**No**	**Yes**	**No**
Madagascar	Yes	Yes	Yes
Malawi	Yes	Yes	Yes
Malaysia	Yes	Yes	Yes
Maldives	Yes	Yes	Yes
Mali	Yes	Yes	Yes
Malta	Yes	Yes	Yes
Marshall Islands	Yes	Yes	Yes
Mauritania	Yes	Yes	Yes
Mauritius	Yes	Yes	Yes
**Mayotte**	**No**	**Yes**	**No**
Mexico	Yes	Yes	Yes
Moldova	Yes	Yes	Yes
**Monaco**	**Yes**	**Yes**	**No**
Mongolia	Yes	Yes	Yes
Montenegro	Yes	Yes	Yes
Morocco	Yes	Yes	Yes
Mozambique	Yes	Yes	Yes
Myanmar	Yes	Yes	Yes
Namibia	Yes	Yes	Yes
Nauru	Yes	Yes	Yes
Nepal	Yes	Yes	Yes
Netherlands	Yes	Yes	Yes
**New Caledonia**	**No**	**Yes**	**No**
New Zealand	Yes	Yes	Yes
Nicaragua	Yes	Yes	Yes
Niger	Yes	Yes	Yes
Nigeria	Yes	Yes	Yes
**Niue**	**Yes**	**No**	**Yes**
North Korea	Yes	Yes	Yes
North Macedonia	Yes	Yes	Yes
**Northern Mariana Islands**	**Yes**	**Yes**	**No**
Norway	Yes	Yes	Yes
Oman	Yes	Yes	Yes
Pakistan	Yes	Yes	Yes
Palau	Yes	Yes	Yes
Palestine	Yes	Yes	Yes
Panama	Yes	Yes	Yes
Papua New Guinea	Yes	Yes	Yes
Paraguay	Yes	Yes	Yes
Peru	Yes	Yes	Yes
Philippines	Yes	Yes	Yes

Poland	Yes	Yes	Yes
Portugal	Yes	Yes	Yes
Puerto Rico	Yes	Yes	Yes
Qatar	Yes	Yes	Yes
Romania	Yes	Yes	Yes
Russia	Yes	Yes	Yes
Rwanda	Yes	Yes	Yes
Saint Kitts and Nevis	Yes	Yes	Yes
Saint Lucia	Yes	Yes	Yes
Saint Vincent and the Grenadines	Yes	Yes	Yes
Samoa	Yes	Yes	Yes
**San Marino**	**Yes**	**Yes**	**No**
São Tomé and Príncipe	Yes	Yes	Yes
Saudi Arabia	Yes	Yes	Yes
Senegal	Yes	Yes	Yes
Serbia	Yes	Yes	Yes
Seychelles	Yes	Yes	Yes
Sierra Leone	Yes	Yes	Yes
Singapore	Yes	Yes	Yes
Slovakia	Yes	Yes	Yes
Slovenia	Yes	Yes	Yes
Solomon Islands	Yes	Yes	Yes
Somalia	Yes	Yes	Yes
South Africa	Yes	Yes	Yes
South Korea	Yes	Yes	Yes
**South Sudan**	**Yes**	**Yes**	**No**
Spain	Yes	Yes	Yes
Sri Lanka	Yes	Yes	Yes
Sudan	Yes	Yes	Yes
Suriname	Yes	Yes	Yes
Sweden	Yes	Yes	Yes
Switzerland	Yes	Yes	Yes
Syria	Yes	Yes	Yes
Taiwan (province of China)	Yes	Yes	Yes
Tajikistan	Yes	Yes	Yes
Tanzania	Yes	Yes	Yes
Thailand	Yes	Yes	Yes
The Bahamas	Yes	Yes	Yes
The Gambia	Yes	Yes	Yes
Timor-Leste	Yes	Yes	Yes
Togo	Yes	Yes	Yes
**Tokelau**	**Yes**	**No**	**Yes**
Tonga	Yes	Yes	Yes
Trinidad and Tobago	Yes	Yes	Yes
Tunisia	Yes	Yes	Yes
Türkiye	Yes	Yes	Yes

Turkmenistan	Yes	Yes	Yes
Tuvalu	Yes	Yes	Yes
Uganda	Yes	Yes	Yes
UK	Yes	Yes	Yes
Ukraine	Yes	Yes	Yes
United Arab Emirates	Yes	Yes	Yes
**United States Virgin Islands**	**Yes**	**Yes**	**No**
Uruguay	Yes	Yes	Yes
USA	Yes	Yes	Yes
Uzbekistan	Yes	Yes	Yes
Vanuatu	Yes	Yes	Yes
Venezuela	Yes	Yes	Yes
Vietnam	Yes	Yes	Yes
Yemen	Yes	Yes	Yes
Zambia	Yes	Yes	Yes
Zimbabwe	Yes	Yes	Yes

Table S20. Number of people with diabetes estimated by the Global Burden of Diseases, Injuries, and Risk Factors Study (GBD), the International Diabetes Federation (IDF)[11], and the NCD Risk Factor Collaboration (NCD-RisC)[12]

Age Range	GBD	IDF	NCD-RisC
20-79 years*	485 million (456-517)	537 million	--
18+ years**	321 million (304-341)	--	422 million

**2021 estimates from GBD and IDF Atlas 2021*
***2010 estimates from GBD and 2014 estimates from NCD-RisC*

Table S21. YLLs and YLDs counts and age-standardised rates per 100,000 population and the corresponding percentage change in YLLs and YLDs counts and age-standardised rates between 1990 and 2021 for diabetes globally, in 21 Global Burden of Disease regions and all countries
95% confidence intervals in parentheses

Location	YLLs 2021, number (thousands)	YLLs percent change 1990 - 2021, number (%)	YLLs 2021, rate (per 100,000)	YLLs percent change 1990 - 2021, rate (%)	YLDs 2021, number (thousands)	YLDs percent change 1990 - 2021, number (%)	YLDs 2021, rate (per 100,000)	YLDs percent change 1990 - 2021, rate (%)
**Global**	**37800 (35400–40200)**	**126·0 (108·9–140·1)**	**437·4 (409·2–464·1)**	**6·9 (-1·2–13·4)**	**41400 (29500–55400)**	**292·3 (280·9–299·8)**	**477·6 (340·7–637·4)**	**91·0 (86·0–94·5)**
**Central Europe, eastern Europe, and central Asia**	**1900 (1810–2000)**	**103·4 (93·6–113·3)**	**301·2 (286·5–315·7)**	**51·7 (44·2–59·0)**	**2470 (1760–3330)**	**149·3 (144·5–154·1)**	**398·8 (285·0–538·7)**	**89·0 (86·0–92·3)**
Central Asia	394 (348–448)	183·8 (147·6–219·4)	465·5 (413·4–525·5)	71·3 (49·4–92·4)	407 (280–555)	310·2 (292·0–327·0)	458·1 (317·3–626·5)	132·0 (121·5–141·8)
Armenia	14·7 (13·0–16·4)	7·1 (-7·2–20·8)	352·8 (314·0–391·6)	-26·4 (-36·3–-17·0)	17·6 (11·8–24·6)	136·9 (116·8–156·9)	418·4 (283·8–587·7)	62·9 (52·3–74·9)
Azerbaijan	49·8 (35·9–65·7)	173·5 (90·2–283·3)	450·3 (324·9–595·4)	37·9 (-4·2–94·2)	47·9 (33·5–66·5)	397·5 (358·3–432·8)	420·3 (298·6–583·4)	137·1 (119·0–153·5)
Georgia	23·3 (20·6–26·6)	25·2 (3·5–48·7)	413·2 (368·1–471·8)	41·0 (17·2–67·2)	26·1 (17·8–36·6)	106·4 (90·9–121·8)	490·3 (337·1–685·8)	140·7 (123·2–159·2)
Kazakhstan	39·5 (33·2–46·6)	49·9 (25·0–81·4)	212·9 (179·1–248·9)	9·1 (-8·7–31·9)	105 (72·5–141)	219·0 (189·3–249·4)	537·3 (373·9–724·0)	120·9 (100·3–142·9)
Kyrgyzstan	11·3 (9·94–13·1)	124·7 (94·2–160·3)	211·2 (187·1–243·0)	35·9 (18·8–57·2)	19·1 (12·9–26·7)	274·0 (242·2–314·3)	349·5 (238·6–485·2)	116·4 (98·3–140·5)
Mongolia	6·70 (5·47–8·19)	203·2 (121·6–315·5)	231·9 (188·7–284·7)	30·5 (-3·9–85·1)	10·5 (7·49–14·4)	518·6 (473·9–567·9)	332·5 (238·2–455·0)	138·2 (124·2–151·6)
Tajikistan	27·8 (20·6–35·0)	156·7 (83·0–243·9)	445·4 (334·5–551·0)	31·3 (-4·5–73·6)	24·8 (17·2–34·8)	426·6 (385·8–460·9)	356·5 (249·9–496·7)	136·2 (118·5–151·4)

Turkmenistan	28·3 (21·9–35·8)	328·4 (227·0–445·4)	593·2 (462·9–749·9)	99·5 (52·5–153·6)	16·0 (11·0–23·6)	420·9 (374·5–464·9)	336·1 (233·1–490·9)	140·6 (118·0–160·6)
Uzbekistan	192 (159–226)	423·5 (338·7–520·4)	655·0 (544·7–766·3)	131·2 (94·9–175·1)	149 (100–202)	575·5 (517·1–631·3)	492·1 (334·0–678·4)	183·8 (158·9–208·4)
Central Europe	602 (567–646)	29·8 (21·5–38·3)	279·8 (262·5–300·0)	-11·3 (-17·2–-5·5)	949 (658–1290)	122·0 (115·8–127·7)	468·3 (328·4–635·1)	60·8 (56·9–63·9)
Albania	4·14 (3·36–5·38)	70·3 (30·8–128·2)	94·1 (76·3–122·4)	-16·9 (-35·7–10·5)	13·7 (9·65–19·1)	189·2 (167·5–211·3)	323·3 (230·1–451·0)	51·0 (40·6–61·4)
Bosnia and Herzegovina	38·0 (30·6–46·7)	152·1 (93·6–209·0)	622·0 (505·3–764·0)	68·8 (29·4–107·3)	37·2 (25·1–49·6)	195·2 (168·0–219·6)	631·5 (426·0–848·2)	113·6 (97·3–129·8)
Bulgaria	48·3 (42·9–53·9)	-2·6 (-16·4–12·0)	358·2 (317·2–402·7)	-12·3 (-24·5–0·3)	66·8 (44·9–90·6)	89·8 (74·0–108·0)	509·9 (339·6–692·3)	77·9 (65·9–94·6)
Croatia	25·2 (22·3–28·4)	44·9 (23·5–63·8)	277·0 (245·6–313·0)	-0·3 (-14·8–13·2)	37·5 (25·6–51·0)	99·8 (82·8–111·4)	466·1 (319·1–638·6)	58·6 (47·5–68·8)
Czechia	72·3 (64·0–83·3)	63·6 (44·0–86·7)	328·9 (289·7–376·9)	1·7 (-10·8–15·9)	98·8 (66·4–137)	157·3 (137·0–179·6)	500·9 (343·6–692·3)	74·8 (62·8–89·1)
Hungary	52·0 (46·5–56·9)	18·3 (4·1–32·0)	273·3 (246·2–299·6)	-10·5 (-21·3–-0·3)	84·3 (56·9–118)	94·2 (78·9–108·4)	474·2 (324·3–653·4)	56·3 (45·1–67·1)
Montenegro	3·54 (2·94–4·18)	86·9 (49·2–125·8)	358·1 (299·8–418·9)	18·2 (-5·6–42·4)	5·25 (3·58–7·10)	146·5 (129·4–169·1)	538·1 (371·2–733·6)	59·4 (47·9–71·8)
North Macedonia	20·3 (16·2–25·1)	112·7 (61·0–178·8)	630·8 (505·4–771·1)	24·0 (-4·6–61·1)	21·4 (14·5–30·0)	211·6 (184·1–232·4)	637·3 (433·3–896·1)	79·1 (64·9–91·6)
Poland	187 (172–200)	23·0 (13·0–33·0)	267·4 (246·2–287·5)	-22·5 (-28·9–-16·0)	334 (242–440)	134·7 (125·6–142·9)	497·1 (359·5–651·9)	52·0 (46·8–56·5)
Romania	55·2 (49·0–61·3)	2·0 (-8·4–16·2)	153·3 (136·3–170·6)	-20·9 (-28·9–-9·9)	112 (75·0–154)	90·5 (70·6–105·9)	329·5 (220·8–457·6)	56·0 (39·8–67·8)
Serbia	72·2 (62·0–86·2)	42·0 (12·1–70·1)	435·7 (373·4–515·5)	-5·4 (-25·8–13·3)	89·3 (59·5–122)	113·0 (91·6–138·2)	584·4 (387·1–798·1)	62·6 (47·7–80·7)
Slovakia	17·6 (14·2–21·6)	5·3 (-16·3–30·5)	187·6 (151·9–230·8)	-32·8 (-46·4–-16·6)	34·4 (23·7–47·4)	124·8 (105·4–144·0)	377·7 (263·6–517·1)	46·0 (33·5–58·6)
Slovenia	7·06 (6·16–8·12)	14·9 (0·8–36·3)	162·3 (141·6–187·6)	-36·2 (-44·1–-24·3)	15·0 (10·5–20·4)	117·4 (99·9–135·3)	378·5 (266·7–515·9)	32·9 (22·5–43·1)
Eastern Europe	908 (845–968)	172·6 (153·5–192·7)	263·9 (246·5–281·0)	115·3 (100·3–130·4)	1110 (804–1470)	140·1 (133·5–146·6)	333·0 (240·1–444·8)	96·5 (91·3–100·6)
Belarus	16·7 (13·7–19·3)	10·7 (-7·6–33·2)	118·9 (97·4–138·1)	-0·4 (-17·0–20·8)	35·6 (24·8–48·1)	103·1 (84·3–116·8)	234·6 (163·7–318·6)	69·3 (55·8–81·4)
Estonia	6·00 (5·33–6·72)	120·8 (95·3–152·7)	258·2 (230·7–289·2)	81·9 (61·8–107·4)	8·47 (5·87–11·7)	137·5 (121·6–153·4)	377·1 (258·4–522·0)	108·4 (96·1–119·6)
Latvia	11·4 (9·79–12·5)	92·8 (67·6–118·2)	332·7 (285·4–367·9)	87·5 (63·0–112·8)	12·4 (8·82–17·2)	118·9 (103·2–139·4)	369·7 (265·2–513·1)	123·8 (110·7–143·0)
Lithuania	11·8 (10·2–13·2)	137·8 (106·4–172·1)	239·7 (208·8–268·4)	108·6 (81·6–138·0)	15·1 (10·6–20·8)	129·6 (110·3–151·1)	311·3 (218·2–432·2)	107·3 (88·7–124·1)
Moldova	13·2 (11·9–15·1)	50·1 (32·9–70·8)	245·2 (221·7–280·6)	27·5 (13·7–44·2)	23·4 (16·1–32·7)	137·7 (120·3–158·1)	422·8 (293·4–590·4)	92·8 (78·4–107·9)
Russia	785 (726–839)	271·9 (246·0–296·6)	328·3 (304·6–351·0)	176·0 (157·1–194·5)	793 (576–1040)	160·6 (152·6–167·5)	343·0 (247·9–453·7)	99·9 (94·8–104·1)
Ukraine	63·9 (48·1–83·5)	-24·3 (-44·5–-0·2)	95·5 (71·7–124·6)	-24·7 (-44·5–-1·3)	221 (160–292)	93·1 (82·4–105·4)	313·9 (224·3–412·8)	86·7 (76·9–98·2)
**High income**	**3650 (3340–3800)**	**9·3 (4·8–12·1)**	**180·5 (168·4–186·6)**	**-36·7 (-38·1–-35·4)**	**9100 (6530–12200)**	**253·3 (239·0–263·0)**	**496·4 (357·4–664·5)**	**118·2 (110·6–124·6)**
Australasia	78·7 (71·6–84·0)	51·7 (41·8–63·6)	153·9 (142·3–164·2)	-31·3 (-35·7–-26·4)	148 (104–210)	252·8 (220·5–290·0)	315·4 (223·9–448·3)	73·4 (58·6–91·8)
Australia	66·9 (60·6–71·7)	57·8 (47·6–70·6)	154·9 (142·7–165·8)	-29·4 (-34·0–-24·1)	121 (84·2–173)	265·4 (222·3–309·6)	307·4 (216·7–440·1)	78·8 (59·2–101·7)
New Zealand	11·8 (10·9–12·7)	24·2 (11·9–35·7)	147·4 (137·3–157·9)	-40·5 (-46·2–-34·9)	26·9 (19·0–35·4)	206·2 (181·1–229·8)	355·8 (252·6–467·8)	53·6 (40·5–65·3)
High-income Asia Pacific	342 (298–374)	-19·9 (-28·3–-11·4)	80·6 (72·3–88·0)	-62·2 (-65·0–-58·2)	2000 (1440–2650)	250·0 (233·1–270·4)	561·9 (406·8–751·5)	100·0 (91·4–110·2)

Brunei	3·95 (3·40–4·50)	89·4 (52·3–124·4)	1201·8 (1049·3–1377·9)	-39·7 (-50·0–-29·0)	4·41 (3·05–6·09)	671·9 (616·0–720·3)	1077·9 (737·6–1493·2)	128·3 (110·9–143·1)
Japan	146 (129–157)	-41·4 (-45·7–-37·8)	45·5 (42·3–47·6)	-69·8 (-70·7–-68·7)	1250 (908–1650)	178·8 (163·5–197·0)	467·3 (336·3–621·5)	73·4 (65·5–81·6)
Singapore	3·10 (2·88–3·29)	-64·8 (-67·7–-62·2)	37·5 (34·8–39·9)	-90·4 (-91·1–-89·6)	53·6 (37·0–74·9)	374·3 (339·5–412·9)	623·6 (429·3–873·6)	40·4 (29·5–50·4)
South Korea	189 (158–216)	13·3 (-1·1–32·8)	199·4 (167·1–227·2)	-60·8 (-65·6–-54·2)	690 (483–939)	525·1 (484·8–574·0)	767·1 (540·5–1039·4)	141·9 (128·1–159·0)
High-income North America	1660 (1570–1730)	44·4 (40·3–48·9)	283·8 (270·4–294·3)	-16·5 (-19·0–-14·2)	3810 (2740–4960)	339·3 (309·5–361·4)	644·9 (461·3–841·4)	145·6 (129·5–158·7)
Canada	115 (106–123)	32·0 (23·5–42·2)	174·3 (163·7–185·0)	-35·6 (-39·4–-31·3)	321 (222–448)	482·2 (439·8–539·3)	493·8 (347·8–685·6)	185·4 (162·6–212·4)
Greenland	0·150 (0·122–0·174)	23·3 (0·6–49·2)	205·6 (171·5–239·8)	-37·4 (-48·1–-25·6)	0·212 (0·149–0·286)	583·8 (518·1–651·3)	286·8 (203·0–388·0)	274·9 (246·0–305·6)
USA	1550 (1460–1610)	45·4 (41·3–49·6)	295·6 (281·4–306·7)	-15·1 (-17·7–-12·7)	3500 (2520–4510)	331·4 (300·2–356·0)	662·9 (474·9–857·2)	143·3 (126·9–157·2)
Southern Latin America	266 (252–280)	26·2 (19·3–33·2)	309·8 (294·0–324·8)	-31·9 (-35·6–-28·3)	381 (262–534)	271·7 (237·6–293·1)	452·4 (312·4–634·8)	106·3 (88·2–118·2)
Argentina	196 (185–206)	21·4 (13·4–29·0)	354·9 (336·6–372·7)	-28·7 (-33·2–-24·5)	230 (153–324)	217·5 (184·4–238·9)	425·3 (285·0–599·9)	90·8 (71·8–103·7)
Chile	53·3 (49·6–56·3)	46·6 (35·4–58·7)	210·1 (195·3–221·8)	-41·4 (-45·8–-36·6)	130 (92·6–181)	456·8 (417·3–499·5)	515·1 (368·4–716·0)	130·6 (115·2–147·8)
Uruguay	17·6 (16·1–18·7)	29·8 (18·4–41·8)	327·0 (301·9–348·0)	-6·1 (-13·3–2·3)	21·1 (14·8–29·7)	215·7 (193·6–237·9)	426·0 (300·5–591·3)	138·9 (121·7–155·4)
Western Europe	1300 (1140–1370)	-13·3 (-19·1–-9·5)	135·6 (122·5–142·1)	-48·0 (-50·2–-46·1)	2770 (1990–3760)	178·4 (169·6–187·5)	376·2 (269·6–516·6)	98·6 (93·1–105·2)
Andorra	0·244 (0·178–0·318)	87·7 (21·1–159·5)	163·1 (118·9–212·8)	-33·1 (-56·7–-7·3)	0·480 (0·338–0·657)	380·8 (349·5–414·8)	347·5 (245·3–475·7)	106·1 (93·9–119·4)
Austria	29·5 (26·2–31·6)	-8·7 (-15·8–-2·0)	155·8 (141·2–165·4)	-43·0 (-47·0–-39·0)	37·1 (25·2–50·0)	203·4 (186·8–226·6)	246·2 (169·8–334·4)	116·1 (103·8–133·5)
Belgium	21·9 (19·4–23·2)	-30·9 (-35·3–-25·8)	93·6 (85·1–98·9)	-54·9 (-57·0–-51·9)	74·9 (51·0–106)	166·1 (148·5–189·3)	400·6 (278·5–572·7)	96·7 (85·3–112·3)
Cyprus	8·40 (7·33–9·78)	5·8 (-12·9–22·5)	432·3 (380·4–499·5)	-60·9 (-67·3–-54·2)	8·77 (6·11–12·2)	204·5 (186·2–220·5)	441·2 (307·2–615·1)	30·7 (22·2–37·8)
Denmark	21·5 (19·6–23·0)	19·9 (9·3–29·1)	181·2 (166·2–192·3)	-23·8 (-30·1–-18·1)	24·6 (17·1–33·8)	204·6 (188·6–223·9)	259·6 (180·4–361·7)	122·8 (110·9–137·0)
Finland	9·95 (8·92–10·7)	-7·0 (-13·5–0·6)	86·6 (80·3–92·4)	-43·8 (-47·3–-39·7)	45·8 (32·0–62·4)	172·5 (160·5–192·7)	491·0 (344·2–673·3)	90·0 (81·8–101·2)
France	177 (154–189)	31·5 (20·5–40·5)	120·6 (110·3–127·6)	-26·1 (-31·8–-21·4)	249 (177–345)	180·2 (157·0–203·0)	231·1 (160·9–323·6)	96·7 (82·4–111·9)
Germany	320 (277–343)	-12·9 (-19·6–-6·4)	165·2 (147·9–175·1)	-43·3 (-47·2–-39·7)	484 (343–654)	237·0 (214·6–260·0)	316·9 (225·5–432·0)	151·3 (134·4–169·0)
Greece	27·8 (25·5–30·2)	34·7 (24·5–48·5)	118·9 (111·4–127·7)	-13·5 (-19·3–-5·6)	74·3 (50·5–104)	122·7 (106·5–137·2)	415·9 (286·3–582·1)	76·0 (65·5–87·5)
Iceland	0·428 (0·381–0·469)	36·4 (23·7–51·1)	73·1 (65·7–79·9)	-33·3 (-39·4–-26·3)	1·67 (1·17–2·32)	307·3 (274·6–328·6)	335·7 (236·3–472·0)	121·4 (104·2–133·5)
Ireland	6·47 (5·79–7·14)	-24·1 (-31·6–-16·0)	81·9 (73·7–90·2)	-60·5 (-64·2–-56·6)	21·3 (15·1–29·8)	216·7 (195·3–237·6)	304·7 (215·7–427·1)	74·7 (63·6–85·6)
Israel	37·2 (32·6–39·7)	91·8 (77·3–108·0)	297·6 (263·8–317·8)	-26·4 (-31·5–-20·0)	44·7 (31·5–61·6)	267·9 (238·5–286·3)	393·1 (277·7–544·1)	54·7 (42·2–63·2)
Italy	262 (228–281)	-19·8 (-25·6–-15·1)	168·1 (149·7–178·3)	-53·8 (-56·2–-51·6)	403 (296–530)	124·5 (112·3–136·6)	353·0 (254·9–471·7)	58·4 (51·2–65·6)
Luxembourg	1·11 (0·981–1·22)	-3·0 (-14·3–6·8)	102·4 (91·6–112·6)	-52·2 (-57·5–-47·5)	3·19 (2·27–4·43)	253·8 (233·1–271·7)	337·6 (239·6–468·8)	90·2 (79·2–100·1)
Malta	2·36 (2·09–2·62)	22·1 (10·1–36·9)	242·6 (216·8–268·9)	-47·0 (-52·2–-40·7)	3·92 (2·73–5·55)	380·1 (348·2–412·4)	495·3 (351·6–703·3)	157·0 (143·3–171·5)

Monaco	0·0637 (0·0534–0·0764)	35·8 (3·7–80·6)	67·4 (56·0–81·6)	-1·8 (-23·6–30·7)	0·219 (0·155–0·299)	189·7 (170·9–211·0)	308·1 (214·3–419·2)	116·7 (103·7–131·2)
Netherlands	46·8 (42·5–50·4)	-30·2 (-36·2–-24·6)	132·4 (121·8–142·1)	-60·9 (-63·8–-57·9)	90·6 (63·8–123)	148·3 (115·0–183·6)	312·7 (220·5–432·0)	62·7 (43·1–86·5)
Norway	9·75 (8·88–10·4)	0·3 (-4·8–5·8)	103·3 (95·5–109·3)	-34·2 (-37·3–-30·9)	27·1 (19·5–36·0)	91·8 (86·3–98·1)	329·8 (234·3–441·0)	33·7 (29·5–37·5)
Portugal	53·8 (47·1–57·6)	-9·8 (-19·6–-2·3)	205·8 (184·7–218·7)	-52·2 (-56·1–-48·7)	103 (72·2–147)	209·4 (187·0–233·4)	530·3 (374·5–756·1)	107·9 (95·3–121·0)
San Marino	0·0537 (0·0346–0·0731)	42·4 (-5·4–102·9)	78·1 (49·9–107·1)	-33·0 (-55·8–-3·6)	0·178 (0·129–0·247)	267·1 (247·8–285·8)	335·2 (239·0–462·9)	107·8 (98·0–117·5)
Spain	117 (101–127)	-31·0 (-37·4–-25·6)	108·9 (96·9–117·3)	-65·1 (-67·6–-62·9)	437 (305–616)	158·8 (140·8–173·8)	541·2 (381·6–762·9)	65·9 (54·6–76·1)
Sweden	27·7 (24·2–31·1)	3·8 (-9·4–17·2)	128·7 (114·2–142·9)	-32·2 (-40·3–-24·3)	56·2 (40·9–75·2)	114·5 (104·0–128·0)	336·3 (241·2–450·5)	60·2 (52·8–69·3)
Switzerland	16·6 (14·7–18·2)	-37·2 (-42·4–-32·2)	88·5 (80·2–96·3)	-65·1 (-67·8–-62·6)	72·6 (51·3–101)	201·1 (183·1–223·6)	490·3 (346·5–682·7)	90·9 (79·4–104·0)
UK	101 (94·3–105)	-35·4 (-38·0–-33·5)	83·6 (79·2–86·6)	-53·6 (-55·0–-52·3)	500 (356–666)	223·9 (212·0–238·5)	496·8 (346·5–671·2)	153·5 (145·5–165·7)
**Latin America and Caribbean**	**5140 (4840–5540)**	**151·5 (135·9–169·1)**	**815·9 (767·1–878·9)**	**-6·5 (-12·3–0·0)**	**4020 (2820–5380)**	**272·8 (264·0–281·5)**	**630·1 (441·8–842·2)**	**43·5 (40·1–46·1)**
Andean Latin America	307 (259–375)	204·9 (153·6–273·7)	512·1 (433·3–623·8)	9·4 (-9·0–33·6)	275 (186–378)	474·6 (442·6–510·7)	450·0 (303·7–620·7)	109·1 (98·2–123·5)
Bolivia	88·5 (72·2–114)	187·8 (120·6–292·4)	936·8 (768·1–1190·0)	3·1 (-19·9–39·7)	53·9 (37·8–74·6)	499·5 (466·5–538·5)	545·4 (386·2–754·2)	112·6 (101·4–126·0)
Ecuador	96·7 (76·2–120)	237·9 (163·4–318·5)	597·2 (471·5–737·7)	15·8 (-9·5–42·9)	109 (73·7–150)	580·9 (508·9–649·6)	660·4 (444·3–905·6)	135·7 (111·4–159·6)
Peru	122 (94·2–161)	196·5 (116·0–296·1)	357·0 (276·8–469·7)	9·0 (-19·9–45·2)	111 (75·8–154)	391·4 (356·1–434·3)	321·7 (219·2–446·6)	84·1 (72·2–100·8)
Caribbean	464 (401–545)	65·8 (46·4–94·8)	866·3 (747·5–1016·0)	-16·9 (-26·7–-2·5)	460 (307–642)	251·8 (241·1–264·1)	855·8 (572·2–1195·2)	78·1 (72·9–83·5)
Antigua and Barbuda	1·25 (1·14–1·33)	65·4 (51·1–77·4)	1191·0 (1091·0–1261·1)	-18·5 (-25·3–-12·3)	1·11 (0·743–1·59)	263·6 (235·6–298·6)	1011·6 (677·5–1439·4)	68·1 (52·3–83·9)
The Bahamas	3·53 (2·85–4·50)	82·3 (45·6–133·9)	836·7 (678·1–1062·2)	-29·2 (-43·1–-9·6)	4·07 (2·77–5·77)	349·8 (320·3–376·9)	922·5 (629·1–1299·5)	75·6 (63·1–88·4)
Barbados	5·37 (4·27–6·93)	18·2 (-10·4–53·4)	1068·5 (849·9–1379·2)	-33·0 (-49·0–-12·7)	4·57 (3·04–6·36)	182·4 (164·3–211·7)	947·3 (630·2–1322·6)	60·3 (51·1–75·9)
Belize	3·99 (3·63–4·48)	263·1 (224·7–307·3)	1260·3 (1138·5–1411·6)	13·9 (1·9–28·0)	2·79 (1·83–3·89)	549·5 (486·9–603·8)	822·2 (544·5–1146·4)	91·3 (73·9–110·4)
Bermuda	0·448 (0·382–0·538)	-3·8 (-18·2–16·5)	338·4 (289·1–406·4)	-54·8 (-61·9–-45·4)	0·699 (0·486–0·973)	227·7 (199·2–251·7)	590·0 (415·6–817·7)	77·7 (62·0–90·9)
Cuba	40·7 (35·4–46·0)	-19·5 (-31·0–-9·0)	211·1 (183·5–237·8)	-56·9 (-62·9–-51·4)	108 (73·3–153)	190·7 (169·5–212·4)	595·8 (401·5–841·4)	67·2 (54·9–78·9)
Dominica	1·40 (1·23–1·63)	32·6 (14·2–60·3)	1525·3 (1338·8–1779·8)	2·8 (-11·9–24·3)	0·950 (0·645–1·35)	131·0 (114·1–149·7)	1066·7 (720·7–1510·2)	73·6 (61·2–86·6)
Dominican Republic	70·8 (56·0–89·7)	212·6 (141·0–299·1)	705·3 (555·4–896·7)	30·4 (-0·3–66·1)	88·4 (59·4–123)	432·1 (396·1–467·1)	861·0 (581·8–1200·4)	119·1 (104·6–133·0)
Grenada	2·06 (1·82–2·26)	56·6 (36·6–78·3)	1800·4 (1603·1–1963·8)	-3·1 (-15·0–10·3)	1·34 (0·868–1·85)	211·8 (185·0–238·6)	1107·5 (721·6–1521·9)	74·8 (61·6–88·7)
Guyana	13·7 (10·8–17·4)	71·3 (32·6–116·2)	1997·4 (1587·7–2508·6)	4·1 (-18·2–29·8)	10·7 (7·19–14·9)	189·9 (175·3–210·4)	1480·1 (1001·4–2057·5)	74·6 (65·2–86·3)
Haiti	150 (113–218)	94·4 (41·8–160·9)	1875·6 (1441·8–2706·4)	-11·5 (-35·3–17·3)	92·5 (61·8–129)	305·5 (280·7–328·2)	1055·4 (713·0–1462·8)	70·8 (60·7–80·2)

Jamaica	41·4 (32·0–51·0)	57·7 (21·8–100·4)	1338·7 (1035·3–1649·3)	-8·9 (-29·9–15·8)	24·0 (16·4–33·3)	194·6 (174·8–215·2)	777·2 (532·2–1077·7)	68·3 (57·4–81·0)
Puerto Rico	58·2 (49·5–67·5)	42·3 (17·4–67·7)	865·8 (735·5–1003·5)	-23·5 (-37·3–-9·7)	65·2 (43·7–92·2)	189·5 (172·2–213·0)	1068·4 (719·1–1509·0)	71·3 (61·7–86·2)
Saint Kitts and Nevis	0·740 (0·584–0·874)	18·5 (-8·2–42·0)	1053·9 (850·6–1226·5)	-37·9 (-50·5–-26·8)	0·754 (0·524–1·04)	253·3 (223·6–284·5)	977·6 (686·1–1352·7)	61·4 (48·5–72·4)
Saint Lucia	2·61 (2·16–3·12)	44·5 (21·6–72·2)	1153·9 (958·6–1381·4)	-43·4 (-52·3–-32·5)	2·66 (1·76–3·69)	263·4 (236·3–288·2)	1155·2 (763·0–1595·7)	42·4 (32·1–51·5)
Saint Vincent and the Grenadines	2·17 (1·95–2·48)	40·9 (24·3–60·8)	1551·6 (1395·4–1759·9)	-26·6 (-35·2–-16·5)	1·69 (1·13–2·41)	214·3 (192·2–237·3)	1180·7 (791·6–1675·3)	61·2 (50·2–72·3)
Suriname	6·53 (5·24–8·09)	157·5 (100·1–216·2)	1007·6 (813·2–1246·5)	8·2 (-15·9–33·6)	7·45 (5·16–10·2)	388·1 (355·7–435·0)	1132·9 (780·5–1540·3)	107·3 (94·5–125·0)
Trinidad and Tobago	42·2 (32·9–53·4)	61·5 (24·8–105·3)	2167·4 (1693·5–2738·4)	-28·6 (-44·8–-9·7)	25·1 (17·0–35·0)	238·7 (212·1–264·8)	1300·6 (876·3–1812·0)	53·8 (41·7–65·1)
Virgin Islands	1·37 (1·08–1·72)	67·3 (21·6–116·1)	800·6 (636·5–1002·5)	-16·1 (-38·1–7·8)	2·22 (1·55–3·05)	253·5 (222·7–280·2)	1282·1 (904·9–1769·9)	90·0 (75·8–104·4)
Central Latin America	2880 (2640–3130)	196·6 (169·9–223·2)	1124·1 (1030·5–1223·4)	2·9 (-6·4–12·1)	1930 (1340–2590)	271·5 (260·7–282·8)	741·8 (515·0–995·0)	36·2 (32·5–40·0)
Colombia	157 (134–184)	76·9 (51·6–111·1)	279·9 (239·2–328·2)	-40·7 (-49·2–-29·3)	313 (215–428)	299·1 (261·7–338·7)	561·4 (385·7–769·4)	44·7 (31·6–57·8)
Costa Rica	21·0 (18·8–23·6)	238·5 (202·8–280·2)	382·4 (342·4–429·8)	11·1 (-0·6–24·6)	38·2 (26·1–53·7)	416·3 (381·7–451·2)	692·0 (473·8–975·4)	80·7 (68·2–92·2)
El Salvador	61·9 (53·0–78·2)	255·4 (195·8–352·1)	1011·4 (866·9–1280·9)	85·0 (54·2–134·2)	37·7 (25·9–49·9)	295·2 (269·6–323·0)	614·1 (422·7–813·5)	109·2 (96·6–124·9)
Guatemala	176 (151–202)	817·7 (694·1–969·6)	1532·0 (1319·4–1755·7)	251·4 (204·1–310·7)	101 (67–139)	625·6 (575·8–686·2)	845·2 (566·6–1155·4)	159·9 (142·4–181·2)
Honduras	44·1 (33·5–57·8)	404·7 (278·3–571·5)	661·9 (505·3–865·9)	74·7 (31·6–130·9)	56·4 (38·5–77·8)	503·8 (469·1–544·8)	772·1 (532·7–1067·9)	95·5 (83·6–109·7)
Mexico	2050 (1860–2220)	180·4 (151·7–202·7)	1607·0 (1457·9–1737·3)	-0·6 (-10·8–7·3)	1110 (781–1470)	218·2 (210·6–226·4)	844·4 (597·0–1122·0)	18·7 (15·8–21·7)
Nicaragua	35·6 (30·3–42·7)	252·1 (191·3–329·8)	726·8 (619·3–871·5)	24·0 (0·9–51·3)	40·8 (26·8–58·1)	433·9 (400·4–481·1)	771·2 (517·7–1094·4)	79·2 (68·2–93·6)
Panama	26·6 (21·6–31·4)	274·7 (200·1–354·0)	599·4 (486·4–707·3)	31·2 (4·7–59·0)	29·6 (20·3–41·6)	417·7 (379·5–463·3)	665·8 (455·6–934·2)	89·2 (75·0–105·4)
Venezuela	303 (242–384)	276·2 (202·7–380·1)	972·3 (779·7–1227·3)	23·2 (-1·2–57·2)	199 (135–277)	398·9 (361·6–446·5)	625·0 (426·0–866·7)	74·6 (60·0–89·1)
Tropical Latin America	1490 (1420–1550)	115·2 (107·4–122·2)	577·2 (548·3–600·9)	-19·6 (-22·2–-17·2)	1360 (969–1790)	256·4 (243·5–270·3)	515·2 (368·6–680·5)	35·7 (30·6–40·4)
Brazil	1420 (1350–1470)	108·8 (101·9–115·3)	562·5 (534·2–583·6)	-22·1 (-24·5–-19·9)	1320 (942–1740)	253·4 (240·4–267·2)	512·7 (367·0–677·1)	34·5 (29·5–39·2)
Paraguay	71·8 (57·0–92·3)	447·8 (320·6–644·5)	1207·3 (960·5–1547·2)	117·3 (67·0–195·6)	38·6 (25·9–53·3)	399·3 (357·4–440·6)	624·3 (419·9–860·4)	92·4 (75·6–109·0)
**North Africa and Middle East**	**2760 (2420–3120)**	**197·1 (147·7–233·2)**	**592·8 (522·1–670·0)**	**16·7 (-2·0–31·3)**	**3890 (2700–5340)**	**606·9 (585·1–626·2)**	**745·5 (522·9–1020·7)**	**158·0 (150·5–165·1)**
North Africa and Middle East	2760 (2420–3120)	197·1 (147·7–233·2)	592·8 (522·1–670·0)	16·7 (-2·0–31·3)	3890 (2700–5340)	606·9 (585·1–626·2)	745·5 (522·9–1020·7)	158·0 (150·5–165·1)
Afghanistan	159 (121–205)	196·5 (119·7–285·0)	977·7 (754·6–1242·6)	48·9 (7·7–89·6)	207 (141–285)	553·2 (496·3–615·7)	1121·3 (788·3–1556·8)	165·1 (149·4–178·3)
Algeria	124 (97·8–150)	291·8 (202·8–405·0)	351·7 (278·5–427·7)	41·0 (6·1–78·6)	314 (217–431)	624·7 (566·5–686·8)	796·8 (547·0–1096·7)	149·6 (129·8–170·6)

Bahrain	18·2 (15·2–21·2)	433·2 (324·7–567·0)	1877·8 (1572·0–2130·2)	-5·1 (-22·6–21·3)	19·3 (13·2–26·1)	1390·4 (1290·5–1492·7)	1247·6 (864·6–1678·1)	115·2 (101·4–130·7)
Egypt	697 (560–856)	264·0 (192·0–348·5)	1020·7 (830·4–1242·3)	73·3 (41·3–110·4)	522 (345–720)	787·5 (735·1–843·2)	692·7 (472·8–953·5)	284·9 (262·0–306·6)
Iran	280 (251–301)	272·8 (206·5–322·4)	368·9 (329·2–397·3)	32·2 (8·1–49·2)	500 (358–649)	588·6 (565·7–610·8)	592·4 (426·6–760·9)	139·8 (132·0–146·2)
Iraq	253 (182–320)	210·5 (122·3–298·7)	1007·8 (734·3–1249·9)	5·4 (-22·3–33·2)	355 (243–495)	645·7 (586·9–699·6)	1186·0 (823·6–1641·2)	118·8 (102·9–134·8)
Jordan	56·8 (45·0–69·9)	238·2 (146·5–339·2)	775·8 (625·4–940·2)	-36·8 (-53·0–-18·6)	91·5 (64·4–125)	1022·8 (951·5–1089·4)	1016·5 (724·1–1373·3)	91·9 (79·7–102·8)
Kuwait	12·7 (10·7–15·1)	324·4 (249·4–411·8)	465·7 (390·6–550·9)	3·5 (-13·7–24·5)	49·4 (33·4–66·9)	904·6 (832·2–975·8)	1201·0 (828·5–1636·4)	105·8 (91·4–122·8)
Lebanon	27·6 (20·4–33·6)	54·8 (8·6–99·7)	509·6 (376·1–618·3)	-34·6 (-53·8–-15·2)	53·2 (37·5–71·8)	378·0 (341·1–414·9)	971·9 (685·5–1309·0)	109·4 (92·8–125·7)
Libya	31·2 (22·1–41·3)	408·9 (246·6–604·6)	547·3 (391·7–715·0)	80·9 (24·8–144·9)	53·8 (37·3–74·9)	706·5 (652·0–757·7)	845·2 (595·0–1173·6)	166·3 (149·6–184·5)
Morocco	172 (125–211)	302·0 (175·5–436·5)	514·2 (379·5–620·6)	82·7 (29·3–135·3)	387 (263–527)	564·6 (522·9–599·6)	1078·6 (745·9–1466·1)	178·6 (160·4–194·7)
Oman	19·0 (15·5–22·5)	182·7 (90·7–277·9)	949·0 (794·6–1113·8)	4·2 (-29·1–38·1)	20·6 (13·9–28·2)	574·4 (534·3–621·7)	707·7 (490·3–972·9)	102·7 (90·1–114·4)
Palestine	23·9 (20·7–27·0)	166·7 (93·8–227·1)	994·9 (865·8–1120·0)	-1·3 (-28·3–21·9)	23·1 (16·6–31·6)	582·2 (527·7–625·7)	787·6 (559·4–1068·9)	116·9 (101·1–132·1)
Qatar	11·5 (8·49–15·1)	612·1 (365·1–885·6)	999·4 (778·4–1280·9)	-33·2 (-54·8–-9·3)	22·7 (15·5–31·5)	2352·6 (2188·2–2599·2)	1217·7 (835·8–1654·4)	111·1 (96·8–129·8)
Saudi Arabia	162 (127–204)	382·5 (185·3–582·9)	672·9 (548·7–794·2)	30·6 (-18·7–76·4)	229 (158–311)	752·3 (684·8–825·0)	783·9 (546·6–1066·6)	114·8 (97·9–135·6)
Sudan	91·0 (70·1–121)	170·5 (89·8–277·6)	423·0 (329·0–551·7)	40·5 (2·4–87·5)	134 (92·4–183)	433·0 (392·5–470·1)	566·8 (403·0–776·0)	132·3 (115·7–153·7)
Syria	50·7 (37·7–67·0)	119·1 (51·5–224·5)	399·3 (301·5–524·3)	0·6 (-30·1–47·6)	96·2 (66·8–133)	383·7 (355·9–416·4)	690·8 (483·7–952·7)	109·2 (97·6–122·0)
Tunisia	44·3 (30·1–56·7)	285·6 (155·9–437·2)	331·9 (228·2–421·9)	51·6 (1·6–107·3)	107 (76·8–151)	575·0 (518·3–618·5)	779·3 (558·0–1084·2)	166·0 (145·7–182·7)
Türkiye	449 (339–549)	72·7 (23·7–119·1)	489·8 (370·1–599·6)	-31·9 (-50·0–-13·7)	562 (393–791)	492·2 (456·3–544·0)	584·2 (411·0–823·2)	136·2 (120·5–157·4)
United Arab Emirates	25·0 (18·7–31·8)	534·7 (290·6–709·9)	769·6 (606·4–946·0)	-18·4 (-45·6–1·9)	57·2 (37·9–81·6)	2153·9 (1973·5–2330·0)	716·7 (507·6–1014·0)	82·0 (69·3–95·2)
Yemen	52·4 (37·9–76·8)	195·7 (115·0–311·3)	337·4 (244·1–496·8)	17·4 (-12·0–59·9)	79·3 (56·0–112)	547·7 (519·0–579·9)	463·0 (329·2–653·9)	116·6 (106·9–127·6)
**South Asia**	**10100 (8950–11000)**	**213·9 (167·0–261·2)**	**675·5 (602·4–733·6)**	**24·0 (6·2–43·6)**	**7900 (5620–10400)**	**369·6 (357·6–379·5)**	**477·9 (342·0–632·0)**	**89·9 (84·7–94·2)**
South Asia	10100 (8950–11000)	213·9 (167·0–261·2)	675·5 (602·4–733·6)	24·0 (6·2–43·6)	7900 (5620–10400)	369·6 (357·6–379·5)	477·9 (342·0–632·0)	89·9 (84·7–94·2)
Bangladesh	816 (679–1000)	179·8 (115·2–249·8)	600·8 (502·2–734·0)	2·2 (-21·3–28·8)	838 (597–1160)	501·4 (459·9–540·0)	547·9 (388·1–755·6)	125·2 (112·5–138·2)
Bhutan	3·70 (2·72–4·72)	166·5 (86·7–266·0)	638·0 (472·2–809·8)	22·2 (-11·2–69·3)	2·80 (1·95–3·88)	300·7 (279·4–329·9)	423·5 (296·2–587·2)	87·9 (76·0–98·9)
India	7920 (6940–8740)	216·0 (160·0–274·7)	653·8 (576·1–720·5)	25·5 (3·9–50·0)	6010 (4270–7860)	353·2 (341·4–364·7)	452·4 (323·1–592·2)	83·7 (78·1–88·7)
Nepal	153 (118–191)	221·5 (136·5–349·1)	660·6 (514·9–814·5)	38·2 (3·1–94·2)	151 (106–212)	387·7 (356·2–420·5)	579·7 (405·0–806·6)	108·8 (95·3–121·0)
Pakistan	1170 (1000–1450)	231·2 (169·6–322·8)	971·8 (830·0–1188·6)	62·3 (34·0–104·3)	902 (637–1200)	385·7 (365·4–407·8)	632·8 (448·8–829·0)	118·3 (109·3–127·5)

**Southeast Asia, east Asia, and Oceania**	**9080 (8290–10100)**	**124·1 (99·8–153·3)**	**319·4 (291·9–353·1)**	**-5·6 (-15·5–7·0)**	**11700 (8310–15400)**	**270·7 (255·9–286·2)**	**416·2 (295·2–546·3)**	**68·1 (61·0–75·8)**
East Asia	4030 (3330–4720)	90·8 (48·6–127·0)	184·5 (152·9–215·7)	-20·0 (-37·3–-5·4)	8360 (5920–11000)	242·1 (227·5–258·7)	407·9 (287·2–539·3)	66·2 (57·4–77·4)
China	3720 (3010–4380)	92·8 (46·4–132·6)	176·1 (143·0–206·9)	-19·2 (-38·3–-3·1)	8010 (5680–10500)	240·5 (225·1–257·3)	405·4 (285·8–537·0)	65·6 (56·6–77·5)
North Korea	116 (87·2–145)	112·2 (49·3–189·8)	345·8 (260·8–430·8)	5·5 (-24·5–41·8)	141 (95·3–195)	274·4 (253·7–293·7)	419·1 (285·5–581·5)	98·8 (87·8–111·0)
Taiwan (province of China)	192 (178–204)	52·7 (41·4–63·5)	468·9 (435·2–496·1)	-39·7 (-43·9–-35·5)	214 (148–295)	292·5 (266·7–318·0)	533·3 (365·6–737·3)	69·9 (60·6–79·4)
Oceania	214 (179–254)	165·0 (99·0–224·6)	2579·5 (2191·2–3053·7)	5·6 (-20·0–28·7)	94·2 (64·3–128)	445·3 (415·5–468·3)	997·5 (690·1–1349·3)	111·1 (100·8–120·0)
American Samoa	1·33 (1·13–1·59)	158·2 (101·0–231·9)	2593·1 (2201·4–3067·3)	22·1 (-4·1–55·6)	0·910 (0·621–1·24)	333·9 (311·4–359·9)	1714·7 (1175·9–2327·4)	129·8 (117·0–143·3)
Cook Islands	0·651 (0·545–0·772)	53·3 (15·7–90·7)	2469·9 (2051·0–2932·7)	-24·8 (-43·1–-6·8)	0·381 (0·260–0·517)	233·9 (212·4–256·5)	1559·4 (1057·7–2122·8)	90·0 (77·3–103·2)
Federated States of Micronesia	2·21 (1·69–2·93)	108·8 (58·0–176·5)	2781·4 (2166·0–3606·4)	31·9 (1·5–73·6)	0·985 (0·697–1·35)	252·0 (232·4–272·6)	1152·3 (818·6–1556·0)	127·1 (115·4–137·8)
Fiji	48·3 (38·4–60·5)	163·0 (92·2–241·2)	5972·2 (4822·9–7407·9)	28·5 (-5·6–65·4)	11·6 (7·78–16·0)	313·8 (281·0–347·1)	1361·7 (913·2–1859·2)	111·5 (97·1–128·9)
Guam	1·18 (1·04–1·32)	68·8 (42·7–98·1)	590·9 (520·0–655·8)	-34·2 (-43·4–-23·4)	1·35 (0·921–1·85)	270·6 (246·2–300·4)	698·1 (476·3–955·8)	81·5 (71·2–94·3)
Kiribati	3·39 (2·53–4·37)	142·6 (77·4–226·5)	4340·2 (3297·3–5381·7)	25·6 (-7·0–64·3)	1·03 (0·683–1·40)	294·8 (269·1–316·9)	1170·4 (786·8–1561·7)	100·6 (89·4–111·5)
Marshall Islands	1·63 (1·09–2·17)	262·0 (150·2–361·8)	3981·4 (2738·6–5293·3)	56·0 (8·9–95·7)	0·825 (0·557–1·12)	373·0 (343·7–401·9)	1769·4 (1202·7–2389·2)	112·3 (101·1–125·2)
Nauru	0·190 (0·143–0·244)	48·9 (12·0–109·0)	3581·6 (2781·8–4489·0)	25·0 (-3·7–71·2)	0·0820 (0·0558–0·112)	147·6 (132·2–163·2)	1288·7 (873·9–1732·4)	101·4 (91·8–113·6)
Niue	0·0577 (0·0443–0·0737)	45·0 (0·9–83·1)	2620·6 (2016·1–3334·3)	41·2 (-1·7–78·2)	0·0309 (0·0213–0·0421)	121·4 (109·3–134·9)	1474·4 (1015·7–2005·3)	122·3 (110·4–134·9)
Northern Mariana Islands	0·886 (0·696–1·06)	192·6 (109·9–267·0)	1401·6 (1121·8–1660·7)	-7·7 (-30·4–14·3)	0·491 (0·336–0·677)	326·6 (284·0–371·3)	797·8 (545·1–1095·2)	96·0 (83·3–110·0)
Palau	0·552 (0·444–0·710)	179·9 (105·7–290·6)	2363·2 (1930·5–2993·6)	22·3 (-7·1–69·1)	0·339 (0·224–0·467)	355·0 (323·5–396·4)	1363·8 (910·3–1839·7)	106·2 (92·6–122·9)
Papua New Guinea	123 (95·5–155)	175·2 (79·6–282·6)	2119·0 (1670·9–2625·3)	-1·0 (-33·8–37·4)	63·6 (43·5–87·1)	535·9 (497·7–573·8)	942·9 (653·9–1276·4)	118·4 (105·6–131·6)
Samoa	3·33 (2·59–4·13)	112·4 (58·8–173·1)	2123·4 (1667·1–2606·5)	22·2 (-8·1–57·1)	2·15 (1·41–2·95)	260·0 (232·6–280·6)	1267·3 (837·8–1720·5)	107·8 (94·6–120·7)
Solomon Islands	10·6 (8·14–13·6)	240·1 (124·0–415·0)	2757·6 (2157·7–3486·6)	35·3 (-9·1–91·4)	3·00 (2·03–4·20)	442·3 (407·0–480·9)	715·5 (491·2–978·9)	122·5 (108·3–139·0)
Tokelau	0·0292 (0·0234–0·0374)	20·9 (-10·4–57·4)	1922·0 (1539·2–2427·4)	6·6 (-20·5–38·7)	0·0211 (0·0143–0·0284)	121·6 (105·1–141·0)	1423·0 (961·0–1912·2)	95·8 (81·1–112·8)
Tonga	2·03 (1·64–2·53)	66·2 (26·0–117·4)	2494·9 (2019·3–3106·4)	18·5 (-10·0–54·2)	0·961 (0·650–1·30)	173·8 (159·0–188·3)	1145·1 (776·7–1550·6)	98·2 (87·2–108·7)
Tuvalu	0·254 (0·196–0·322)	66·1 (32·9–104·4)	2339·5 (1820·8–2933·0)	10·1 (-11·4–35·2)	0·103 (0·0710–0·142)	227·8 (203·5–246·0)	920·3 (635·8–1258·1)	114·5 (97·3–127·2)
Vanuatu	4·09 (3·27–4·96)	247·0 (155·1–379·6)	2108·7 (1691·1–2538·8)	26·5 (-4·7–70·2)	1·92 (1·31–2·62)	509·5 (468·2–548·8)	898·3 (616·3–1227·3)	128·9 (114·4–142·7)
Southeast Asia	4850 (4420–5350)	160·9 (127·8–194·9)	731·2 (669·7–805·7)	8·8 (-4·7–22·7)	3250 (2280–4360)	366·4 (347·3–383·4)	489·5 (341·2–649·2)	93·2 (86·6–99·3)
Cambodia	98·9 (73·2–129)	169·5 (92·8–251·4)	746·2 (555·9–962·4)	3·7 (-24·8–34·0)	60·7 (43·2–83·3)	613·8 (557·9–671·2)	459·0 (328·0–621·7)	168·1 (149·1–188·8)

Indonesia	1550 (1290–1780)	178·1 (128·7–223·6)	649·8 (543·4–739·0)	29·5 (6·5–50·7)	1030 (728–1340)	333·4 (317·6–348·3)	417·2 (297·7–542·0)	94·3 (88·3–100·8)
Laos	41·8 (32·4–54·0)	94·2 (39·8–179·7)	842·4 (663·6–1080·5)	-9·3 (-33·2–29·0)	27·6 (18·7–37·8)	431·9 (395·2–465·4)	556·8 (381·7–753·1)	139·2 (124·0–155·6)
Malaysia	131 (119–144)	113·2 (85·7–140·5)	443·1 (402·6–489·0)	-30·6 (-39·9–-21·7)	186 (130–249)	415·7 (377·5–447·4)	630·6 (441·9–845·4)	75·4 (63·4–86·6)
Maldives	1·32 (1·04–1·57)	53·3 (14·7–92·8)	372·3 (293·6–435·8)	-56·1 (-67·3–-44·5)	1·90 (1·28–2·61)	658·1 (606·0–724·3)	495·3 (341·9–667·6)	91·3 (76·9–104·3)
Mauritius	46·2 (43·2–48·3)	320·4 (297·5–346·0)	2462·6 (2313·9–2569·7)	79·7 (70·4–90·4)	18·9 (13·0–26·0)	399·1 (367·1–435·0)	1017·9 (696·9–1398·3)	109·8 (97·9–126·0)
Myanmar	640 (511–807)	71·9 (21·3–137·9)	1274·4 (1029·1–1597·5)	-12·6 (-37·4–20·5)	364 (253–495)	341·4 (310·5–369·5)	722·1 (500·0–988·9)	116·0 (100·5–129·8)
Philippines	846 (803–894)	277·0 (242·2–318·4)	956·4 (911·2–1011·7)	43·7 (30·9–59·3)	348 (247–445)	248·9 (235·7–262·9)	401·5 (288·7–516·2)	31·9 (26·7–36·8)
Seychelles	0·616 (0·543–0·700)	180·0 (134·9–230·7)	524·7 (460·7–592·9)	35·8 (14·6–60·3)	1·20 (0·832–1·64)	551·1 (501·4–591·5)	1000·1 (695·6–1367·1)	211·7 (191·1–231·0)
Sri Lanka	261 (182–351)	210·1 (106·0–331·9)	967·4 (683·4–1297·7)	25·1 (-16·0–72·4)	269 (186–375)	467·1 (398·3–538·3)	985·1 (684·4–1378·8)	129·8 (101·7–159·8)
Thailand	544 (439–680)	150·5 (86·9–249·5)	506·0 (408·8–632·2)	-11·1 (-34·2–24·6)	528 (367–733)	491·5 (437·4–546·6)	490·4 (340·6–679·6)	111·1 (93·3–126·4)
Timor-Leste	4·64 (3·61–6·26)	201·9 (114·1–315·3)	517·0 (403·6–689·3)	13·2 (-18·3–54·8)	4·76 (3·36–6·56)	779·7 (717·7–867·3)	534·4 (379·7–735·1)	230·8 (210·3–251·7)
Viet Nam	676 (542–826)	164·2 (90·0–241·1)	702·0 (567·3–845·1)	11·3 (-19·4–45·0)	413 (288–575)	379·8 (340·3–416·4)	416·3 (290·7–572·4)	100·5 (83·6–117·7)
**Sub-Saharan Africa**	**5250 (4700–5840)**	**136·0 (104·6–160·4)**	**993·6 (896·2–1085·4)**	**7·2 (-6·3–18·2)**	**2310 (1610–3150)**	**346·7 (335·4–357·6)**	**393·9 (275·6–524·0)**	**83·4 (78·5–88·5)**
Central sub-Saharan Africa	713 (564–848)	143·2 (88·0–215·0)	1148·8 (922·9–1358·4)	-1·5 (-24·1–23·9)	350 (236–494)	425·2 (402·3–447·0)	482·5 (325·1–675·3)	89·9 (82·4–97·8)
Angola	146 (111–186)	189·7 (101·0–300·8)	1083·3 (835·9–1360·5)	-3·0 (-31·9–32·0)	89·7 (60·9–125)	524·8 (492·7–563·2)	567·2 (391·0–786·0)	87·0 (77·3–97·5)
Central African Republic	38·4 (28·5–48·2)	95·1 (38·1–159·1)	1479·9 (1118·4–1797·2)	-0·5 (-26·4–27·5)	19·2 (13·1–27·0)	318·0 (293·8–344·0)	641·0 (431·8–880·8)	91·5 (79·6–103·3)
Congo (Brazzaville)	43·4 (34·9–52·8)	144·1 (86·5–230·4)	1425·1 (1192·6–1704·5)	-6·9 (-25·5–21·8)	18·3 (12·6–25·6)	489·0 (451·8–534·8)	528·9 (360·9–738·7)	105·6 (92·3–120·5)
DR Congo	460 (343–564)	137·9 (75·2–222·0)	1112·2 (827·9–1352·6)	-0·2 (-26·1–32·4)	210 (141–300)	399·1 (371·7–427·6)	436·5 (291·5–616·3)	86·9 (76·7–97·3)
Equatorial Guinea	7·55 (5·36–10·8)	165·7 (76·3–278·2)	1315·0 (968·2–1825·7)	0·7 (-29·7–40·0)	4·10 (2·85–5·57)	605·1 (565·2–642·3)	588·1 (403·6–785·7)	123·5 (111·0–134·6)
Gabon	18·7 (13·5–24·4)	127·7 (70·4–212·1)	1616·4 (1197·8–2059·8)	14·7 (-12·3–53·4)	8·06 (5·63–11·0)	348·6 (316·6–376·6)	628·8 (434·8–851·3)	109·2 (95·4–122·0)
Eastern sub-Saharan Africa	1780 (1580–2040)	84·1 (61·5–115·6)	913·4 (808·0–1037·1)	-16·2 (-26·2–-1·7)	604 (421–817)	296·4 (283·2–307·5)	283·6 (201·1–378·6)	61·6 (56·5–66·6)
Burundi	52·8 (39·5–78·0)	61·8 (20·2–114·5)	965·2 (689·7–1429·5)	-21·1 (-42·5–7·3)	17·1 (11·7–24·0)	262·2 (241·4–291·1)	282·9 (197·3–394·1)	61·6 (51·5–74·0)
Comoros	4·98 (3·47–6·58)	101·7 (39·1–169·1)	942·5 (665·6–1244·7)	-6·2 (-35·4–26·9)	2·40 (1·66–3·33)	334·1 (310·7–362·1)	427·2 (296·2–588·9)	87·0 (77·1–99·0)
Djibouti	6·78 (5·00–9·52)	388·7 (257·8–571·7)	987·6 (759·0–1352·3)	24·4 (-5·7–66·5)	2·37 (1·67–3·35)	727·1 (674·4–780·2)	301·6 (212·7–420·4)	94·7 (83·8–106·8)
Eritrea	39·5 (28·6–52·4)	174·4 (104·6–253·1)	1218·5 (900·3–1557·6)	3·6 (-17·9–27·4)	14·5 (9·74–19·7)	468·8 (429·5–503·8)	388·0 (266·2–531·9)	90·8 (78·2–104·1)
Ethiopia	393 (348–453)	3·7 (-14·8–29·5)	798·1 (704·3–909·3)	-50·0 (-58·8–-37·7)	179 (124–236)	217·8 (206·7–228·0)	327·8 (234·1–423·4)	36·2 (31·6–41·3)

Kenya	197 (165–235)	273·4 (202·1–383·3)	785·5 (666·7–935·2)	37·0 (11·3–72·6)	56·9 (39·7–74·0)	330·2 (316·7–342·0)	202·1 (143·0–262·1)	47·5 (42·9–51·2)
Madagascar	105 (79·9–138)	126·8 (73·3–203·7)	785·4 (606·0–1014·7)	4·8 (-21·1–39·0)	38·8 (26·5–54·2)	329·1 (299·5–359·4)	266·5 (185·5–366·7)	72·8 (61·4–83·5)
Malawi	94·4 (72·7–113)	104·8 (63·0–153·1)	1080·6 (838·6–1285·4)	7·7 (-13·1–33·0)	19·0 (13·3–26·6)	224·0 (204·8–245·7)	203·8 (143·2–286·7)	50·7 (41·3–62·4)
Mozambique	160 (118–199)	147·6 (86·7–214·7)	1164·0 (899·8–1434·9)	29·7 (-2·7–64·9)	44·2 (30·8–60·7)	359·9 (332·7–400·6)	312·8 (217·6–430·4)	112·6 (99·3–133·9)
Rwanda	62·3 (38·3–88·7)	34·9 (-4·4–75·2)	903·4 (558·5–1293·9)	-34·2 (-52·4–-16·4)	16·8 (11·6–23·2)	228·1 (210·2–246·2)	223·4 (153·7–309·3)	45·1 (36·7–53·9)
Somalia	114 (85·7–150)	197·7 (127·1–289·2)	1327·1 (1027·7–1718·1)	6·8 (-15·9–35·4)	28·4 (19·8–39·4)	425·7 (391·7–456·4)	304·0 (211·9–423·3)	74·9 (60·9–86·0)
South Sudan	58·8 (43·4–79·9)	114·0 (54·8–209·7)	1280·0 (966·3–1764·7)	26·6 (-8·2–83·1)	12·9 (8·97–17·8)	206·5 (189·0–223·0)	273·8 (190·3–374·8)	79·3 (70·3–89·8)
Tanzania	244 (199–304)	123·2 (81·4–182·8)	834·2 (687·6–1033·1)	-0·4 (-19·7–26·7)	78·1 (53·5–109)	396·1 (365·0–423·2)	256·0 (175·7–354·2)	97·6 (84·8–110·5)
Uganda	163 (120–235)	156·1 (75·8–240·6)	965·4 (688·8–1391·9)	11·3 (-22·3–47·6)	51·1 (35·3–70·0)	351·1 (327·3–373·6)	274·2 (190·6–383·1)	79·4 (68·6–90·3)
Zambia	88·5 (66·7–116)	128·1 (64·0–199·6)	1058·2 (824·3–1343·4)	-4·7 (-29·3–24·1)	42·0 (28·8–58·3)	410·0 (377·9–439·1)	441·1 (307·5–612·8)	80·0 (67·9–90·9)
Southern sub-Saharan Africa	949 (890–1010)	244·7 (211·8–280·7)	1595·2 (1496·6–1688·2)	66·4 (50·8–83·2)	338 (238–438)	317·4 (298·7–336·3)	533·3 (379·7–688·5)	98·8 (88·8–106·1)
Botswana	17·4 (14·5–20·7)	144·2 (74·5–228·1)	1232·5 (1034·9–1443·7)	1·3 (-26·0–34·9)	7·64 (5·21–10·6)	436·5 (394·6–467·0)	457·4 (311·7–629·1)	102·7 (87·0–115·1)
Eswatini	16·7 (12·6–22·7)	227·8 (142·7–382·9)	2727·0 (2092·5–3644·2)	58·8 (20·9–130·1)	4·04 (2·74–5·56)	344·4 (315·1–381·3)	607·3 (406·4–829·9)	121·1 (106·3–139·6)
Lesotho	29·6 (22·3–38·5)	196·4 (118·2–343·2)	2251·7 (1724·9–2864·7)	126·7 (70·5–229·7)	6·52 (4·48–8·88)	259·3 (228·5–281·1)	459·7 (314·1–620·3)	164·3 (141·4–180·2)
Namibia	22 (16·9–29·1)	134·1 (73·6–207·3)	1513·0 (1169·2–1979·3)	19·5 (-9·9–53·9)	6·29 (4·33–8·73)	261·5 (237·9–286·7)	388·2 (265·5–536·9)	71·5 (61·0–83·6)
South Africa	752 (706–797)	255·5 (219·3–290·5)	1590·5 (1495·0–1682·7)	65·0 (49·3–81·4)	279 (198–361)	322·7 (299·4–341·6)	560·3 (399·9–722·4)	93·9 (82·8–101·2)
Zimbabwe	111 (84·6–142)	253·6 (158·5–380·2)	1490·4 (1149·9–1861·9)	94·7 (45·5–157·8)	34·3 (23·5–48·1)	279·8 (255·6–311·2)	408·8 (285·1–575·6)	106·9 (92·6–125·9)
Western sub-Saharan Africa	1800 (1480–2120)	164·2 (112·0–216·7)	825·8 (697·5–956·4)	16·5 (-6·4–37·2)	1020 (714–1400)	368·8 (355·4–381·6)	419·9 (295·4–567·4)	90·1 (84·1–95·6)
Benin	44·2 (35·0–55·0)	214·6 (129·6–289·9)	763·1 (619·1–939·8)	24·5 (-8·0–51·3)	38·4 (26·3–52·5)	541·1 (501·2–584·7)	580·9 (402·3–791·3)	120·2 (107·0–135·6)
Burkina Faso	81·2 (66·4–99·0)	115·3 (60·5–173·6)	723·5 (590·0–874·6)	-7·6 (-32·4–17·0)	46·5 (32·1–63·7)	455·6 (430·3–496·1)	395·7 (274·8–543·9)	122·9 (110·9–139·1)
Cabo Verde	3·13 (2·54–3·82)	434·6 (317·1–571·5)	690·7 (561·0–841·9)	182·5 (123·5–261·2)	3·08 (2·14–4·17)	405·1 (373·4–439·1)	625·9 (435·3–845·0)	140·1 (124·4–157·2)
Cameroon	150 (114–212)	266·5 (177·9–429·1)	1080·8 (835·9–1482·6)	28·6 (-2·7–82·6)	72·3 (50·2–100)	555·8 (511·5–604·5)	451·4 (310·0–613·4)	107·7 (92·7–125·0)
Chad	55·1 (42·0–76·5)	240·3 (165·1–337·3)	792·6 (595·0–1108·2)	54·7 (19·1–97·6)	32·2 (22·5–45·0)	387·7 (357·6–415·2)	435·2 (304·6–592·1)	104·9 (92·5–116·1)
Côte d'Ivoire	108 (80·5–142)	230·4 (136·3–332·9)	873·7 (672·9–1105·0)	26·0 (-3·7–65·8)	71·2 (49·1–98·0)	470·4 (434·4–501·6)	493·2 (345·6–672·0)	103·4 (88·1–117·3)
The Gambia	10·3 (7·65–13·1)	326·5 (211·1–449·3)	939·4 (686·3–1195·1)	59·1 (18·2–103·1)	5·72 (3·99–7·90)	508·3 (475·3–546·8)	467·6 (329·9–640·8)	114·3 (102·5–127·8)

Ghana	185 (144–228)	341·1 (203·3–503·6)	1024·6 (808·4–1253·5)	72·6 (19·1–139·4)	98·3 (66·7–135)	481·8 (449·1–518·3)	477·4 (322·6–654·7)	109·3 (96·1–123·7)
Guinea	53·9 (42·8–70·6)	132·9 (69·4–218·0)	873·0 (698·6–1138·6)	36·7 (0·6–86·4)	26·6 (18·3–36·7)	273·7 (248·6–296·8)	394·2 (276·3–540·6)	98·0 (84·6–111·3)
Guinea-Bissau	10·4 (8·31–13·0)	118·4 (62·3–193·2)	1218·2 (986·4–1520·2)	21·1 (-9·0–61·2)	5·20 (3·69–7·19)	303·2 (285·0–330·8)	528·8 (376·1–722·6)	96·8 (87·6–109·5)
Liberia	22·1 (15·8–30·9)	169·2 (96·0–257·4)	919·8 (675·7–1286·1)	33·8 (0·2–76·7)	14·2 (9·49–19·3)	404·4 (367·2–454·6)	507·9 (355·1–680·9)	113·2 (100·1–128·1)
Mali	89·2 (72·7–114)	164·4 (107·1–239·5)	898·3 (734·3–1132·5)	21·1 (-4·8–53·4)	86·4 (59·6–119)	390·5 (356·9–416·2)	781·7 (544·9–1086·9)	102·7 (89·4–113·4)
Mauritania	17·0 (12·6–22·8)	140·6 (79·7–237·0)	755·4 (559·3–1004·1)	14·5 (-13·7–58·7)	7·75 (5·44–10·6)	273·5 (253·0–297·3)	310·3 (215·2–426·9)	65·6 (55·1–77·2)
Niger	55·4 (40·3–76·9)	210·7 (129·2–321·9)	581·4 (425·0–805·5)	16·5 (-12·0–51·7)	44·8 (30·2–60·7)	495·8 (458·5–529·8)	434·0 (307·5–587·8)	95·8 (82·1–108·9)
Nigeria	771 (570–935)	119·4 (59·3–189·6)	767·8 (595·4–915·3)	2·5 (-24·4–32·8)	374 (263–504)	284·4 (273·8–295·9)	335·7 (239·6–442·5)	67·6 (62·2–72·1)
São Tomé and Príncipe	0·460 (0·356–0·557)	112·7 (66·2–181·2)	393·9 (315·3–465·8)	30·1 (2·9–62·1)	0·705 (0·486–0·972)	317·7 (289·0–346·3)	517·7 (359·8–703·1)	109·5 (97·7–124·3)
Senegal	79·7 (60·8–103)	216·8 (139·8–306·6)	968·6 (753·9–1242·1)	41·0 (5·2–78·1)	58·3 (40·9–78·8)	388·3 (358·7–424·0)	650·1 (458·4–875·4)	99·3 (86·8–116·0)
Sierra Leone	29·3 (22·7–38·3)	166·1 (101·8–256·9)	698·6 (558·1–902·2)	34·5 (2·2–82·6)	21·3 (14·4–29·5)	361·6 (335·9–389·4)	452·9 (316·4–625·6)	106·8 (94·2–117·9)
Togo	32·6 (24·1–43·4)	300·0 (203·0–434·8)	767·3 (577·5–996·1)	40·4 (9·0–80·9)	15·3 (10·8–21·0)	481·8 (449·4–520·8)	322·6 (224·7–437·4)	88·4 (78·3–101·2)

Table S22. Prevalence counts and age-standardised rates per 100,000 population and the corresponding percentage change between 1990 and 2021 for diabetes globally, in 21 Global Burden of Disease regions and all countries

95% confidence intervals in parentheses

Location	Prevalence 1990, number (thousands)	Prevalence 2021, number (thousands)	Prevalence percent change 1990 - 2021, number (%)	Prevalence 1990, rate (per 100,000)	Prevalence 2021, rate (per 100,000)	Prevalence percent change 1990 - 2021, rate (%)
**Global**	**139000 (130000–150000)**	**529000 (500000–564000)**	**280·7 (272·0–287·0)**	**3223·9 (3029·5–3476·9)**	**6138·6 (5800·3–6536·5)**	**90·5 (85·8–93·6)**
**Central Europe, eastern Europe, and central Asia**	**11200 (10500–12000)**	**27500 (25700–29600)**	**145·3 (140·7–149·3)**	**2408·9 (2252·9–2571·8)**	**4548·4 (4251·4–4891·2)**	**88·8 (86·1–91·2)**
Central Asia	1190 (1120–1300)	4790 (4510–5100)	301·8 (286·1–314·1)	2309·0 (2160·4–2515·7)	5314·8 (4994·7–5661·3)	130·3 (120·7–137·9)
Armenia	86·7 (80·4–96·6)	197 (180–217)	127·9 (113·6–140·9)	2948·0 (2727·4–3280·9)	4793·6 (4364·2–5274·2)	62·7 (52·1–72·2)
Azerbaijan	116 (107–125)	555 (511–601)	379·8 (345·5–412·0)	2060·9 (1907·8–2246·7)	4837·1 (4456·9–5226·8)	134·9 (118·3–151·4)
Georgia	146 (134–157)	294 (271–319)	102·2 (87·8–116·1)	2367·8 (2185·6–2550·1)	5703·4 (5244·6–6187·3)	141·0 (125·5–156·4)
Kazakhstan	394 (356–443)	1230 (1150–1310)	213·4 (185·9–241·6)	2858·9 (2580·8–3214·1)	6304·8 (5887·5–6702·8)	121·0 (101·4–141·6)
Kyrgyzstan	61·9 (56·2–66·9)	228 (211–248)	269·1 (242·6–306·3)	1900·9 (1721·5–2052·9)	4070·1 (3748·7–4425·6)	114·3 (97·6–136·2)
Mongolia	21·4 (19·8–22·9)	129 (119–139)	502·8 (463·6–537·7)	1661·0 (1524·0–1786·7)	3975·8 (3689·5–4263·9)	139·5 (124·7–152·9)
Tajikistan	58·2 (54·4–62·8)	299 (276–324)	414·3 (383·1–447·0)	1777·3 (1653·7–1921·8)	4120·8 (3757·8–4460·6)	132·0 (117·2–146·1)
Turkmenistan	38·2 (35·8–40·8)	192 (175–210)	402·2 (359·9–433·4)	1644·4 (1529·3–1761·0)	3956·6 (3605·9–4358·7)	140·7 (117·9–156·5)
Uzbekistan	272 (252–294)	1750 (1620–1900)	544·2 (499·8–593·7)	2043·2 (1896·6–2224·5)	5683·3 (5270·3–6176·2)	178·4 (156·7–201·5)

Central Europe	4690 (4390–5010)	10200 (9540–10900)	117·4 (112·7–121·4)	3217·9 (3017·2–3431·4)	5147·7 (4833·5–5516·0)	60·0 (56·5–62·7)
Albania	53·9 (48·4–58·0)	148 (131–165)	174·9 (157·1–194·1)	2374·1 (2133·4–2564·5)	3586·5 (3176·5–3956·0)	51·1 (41·6–61·1)
Bosnia and Herzegovina	140 (128–151)	398 (369–424)	184·7 (164·9–205·7)	3247·3 (2960·4–3512·8)	6883·0 (6397·5–7325·6)	112·2 (96·5–125·5)
Bulgaria	384 (349–421)	717 (671–767)	86·7 (73·1–105·2)	3180·9 (2912·4–3466·6)	5614·2 (5266·4–5983·8)	76·8 (64·7–93·1)
Croatia	205 (190–223)	402 (370–439)	96·6 (82·3–107·2)	3234·8 (2999·2–3515·0)	5122·7 (4720·5–5633·1)	58·5 (47·7–67·6)
Czechia	421 (388–460)	1060 (970–1150)	152·5 (134·3–168·8)	3175·9 (2925·4–3460·8)	5501·2 (5040·8–5924·7)	73·4 (61·2–85·3)
Hungary	478 (437–515)	908 (829–994)	90·0 (76·2–103·1)	3384·5 (3103·1–3641·0)	5225·2 (4750·0–5697·5)	54·5 (43·4–64·7)
Montenegro	23·5 (21·1–25·9)	56·6 (51·8–61·9)	141·2 (124·3–160·2)	3712·5 (3337·1–4092·5)	5909·5 (5425·4–6435·0)	59·3 (48·6–71·4)
North Macedonia	75·4 (69·1–81·8)	230 (210–248)	205·1 (183·5–223·5)	3894·4 (3561·9–4230·2)	6941·9 (6370·6–7480·5)	78·4 (65·9–89·5)
Poland	1560 (1420–1710)	3590 (3280–3950)	129·9 (121·6–137·2)	3598·7 (3285·2–3942·0)	5455·6 (5018·4–5991·8)	51·6 (46·5–55·7)
Romania	651 (587–737)	1210 (1110–1320)	86·1 (66·6–100·1)	2355·0 (2122·0–2663·3)	3646·1 (3355·0–3955·3)	55·1 (39·1–67·0)
Serbia	457 (413–501)	958 (880–1040)	109·8 (90·7–134·8)	3946·3 (3585·0–4307·9)	6388·5 (5879·4–6988·0)	62·1 (48·9–80·0)
Slovakia	169 (154–184)	374 (340–408)	121·1 (103·4–139·3)	2868·6 (2606·9–3118·5)	4176·7 (3821·8–4541·9)	45·8 (33·5–57·4)
Slovenia	75·8 (69·4–82·3)	162 (149–178)	114·2 (98·6–128·0)	3140·3 (2882·2–3404·8)	4193·9 (3853·9–4575·2)	33·6 (24·1–42·1)
Eastern Europe	5330 (4960–5780)	12500 (11500–13600)	134·7 (128·0–139·9)	1975·9 (1840·6–2138·2)	3854·8 (3555·3–4183·3)	95·1 (90·1–99·7)
Belarus	201 (185–222)	402 (362–436)	100·1 (83·5–111·5)	1613·4 (1488·0–1776·1)	2727·3 (2467·4–2960·2)	69·2 (55·5–79·8)
Estonia	41·0 (37·4–44·9)	94·7 (85·8–104)	130·9 (117·1–143·6)	2112·4 (1931·5–2303·4)	4360·5 (3952·3–4757·0)	106·5 (94·9–117·4)
Latvia	65·1 (59·2–70·8)	139 (129–153)	112·8 (98·8–127·7)	1928·9 (1758·8–2087·3)	4274·4 (3973·0–4664·4)	121·7 (108·7–135·8)
Lithuania	76·0 (68·8–83·1)	170 (156–185)	123·5 (105·9–138·1)	1750·0 (1588·7–1916·6)	3626·3 (3340·5–3921·2)	107·4 (91·3–121·4)
Moldova	114 (105–126)	264 (239–286)	133·2 (118·3–151·6)	2525·6 (2325·6–2798·7)	4876·2 (4419·5–5254·8)	93·2 (81·3–108·5)
Russia	3510 (3260–3830)	8930 (8230–9780)	154·0 (146·5–160·4)	1996·9 (1854·1–2166·4)	3957·5 (3650·7–4329·2)	98·2 (93·3–101·9)
Ukraine	1320 (1210–1430)	2510 (2270–2720)	90·4 (80·2–100·2)	1977·0 (1821·1–2141·1)	3672·8 (3309·7–3985·5)	85·8 (76·2–95·9)
**High income**	**35300 (33400–37700)**	**120000 (114000–126000)**	**239·5 (231·1–247·2)**	**3149·2 (2989·0–3365·7)**	**6761·3 (6464·4–7081·6)**	**114·8 (109·6–119·7)**
Australasia	500 (467–533)	1780 (1640–1900)	256·0 (240·9–272·2)	2190·0 (2048·2–2337·2)	3907·5 (3626·0–4147·8)	78·5 (71·0–86·1)
Australia	392 (361–423)	1450 (1320–1570)	271·5 (251·6–295·4)	2052·7 (1895·9–2222·3)	3801·5 (3474·1–4071·6)	85·3 (75·1–97·0)
New Zealand	109 (99·4–120)	326 (314–340)	200·7 (176·9–219·9)	2886·3 (2636·9–3191·3)	4444·7 (4288·9–4640·8)	54·2 (41·4–64·0)
High-income Asia Pacific	7760 (7260–8380)	24800 (23400–26300)	219·5 (209·4–228·4)	3828·5 (3580·3–4124·3)	7311·9 (6898·8–7715·4)	91·1 (84·4–96·8)
Brunei	7·70 (7·26–8·12)	58·4 (55·0–62·2)	659·3 (618·2–703·9)	5932·6 (5594·6–6213·4)	13904·0 (13054·6–14873·9)	134·5 (119·2–148·4)

Japan	6080 (5610–6630)	15000 (13700–16200)	146·6 (138·7–155·0)	3683·8 (3395·4–4004·9)	5897·9 (5446·9–6377·4)	60·1 (55·0–65·4)
Singapore	150 (140–160)	696 (646–743)	363·2 (329·9–395·9)	5749·7 (5331·9–6131·9)	8225·0 (7626·3–8753·1)	43·1 (32·0–52·6)
South Korea	1520 (1400–1640)	9030 (8530–9420)	494·8 (462·3–530·3)	4225·2 (3888·7–4550·4)	10346·1 (9782·9–10807·5)	145·1 (132·4–160·9)
High-income North America	11700 (10900–12700)	50300 (48000–52700)	328·6 (309·1–347·9)	3594·1 (3338·3–3901·0)	8748·7 (8405·1–9168·4)	143·6 (132·7–154·7)
Canada	737 (695–787)	4170 (3770–4560)	466·7 (426·0–517·2)	2336·4 (2196·3–2491·1)	6627·4 (6010·8–7170·3)	183·8 (163·4–208·4)
Greenland	0·454 (0·422–0·492)	2·83 (2·58–3·04)	524·1 (462·1–577·8)	1063·8 (999·1–1147·5)	3865·5 (3560·9–4149·8)	263·6 (236·8–296·3)
USA	11000 (10200–11900)	46300 (44000–48700)	321·3 (300·6–340·7)	3733·2 (3456·6–4065·1)	9001·1 (8619·8–9462·1)	141·3 (129·6–152·7)
Southern Latin America	1330 (1250–1420)	4910 (4500–5290)	269·2 (236·7–291·0)	2837·8 (2672·4–3025·9)	5871·8 (5387·7–6325·8)	106·9 (89·2–119·4)
Argentina	936 (882–993)	2970 (2700–3230)	217·2 (184·8–238·1)	2878·4 (2713·4–3050·5)	5519·2 (5013·0–5992·6)	91·8 (72·7–104·4)
Chile	307 (284–334)	1670 (1530–1820)	443·8 (409·8–486·3)	2899·5 (2681·3–3174·4)	6675·4 (6126·1–7277·6)	130·4 (116·3–148·8)
Uruguay	86·3 (79·2–93·6)	271 (247–293)	214·7 (193·4–237·1)	2325·1 (2133·5–2526·6)	5568·0 (5099·4–5998·6)	139·7 (123·6–156·2)
Western Europe	14000 (13200–14700)	38100 (35800–40600)	172·4 (165·7–180·3)	2706·5 (2550·4–2851·4)	5378·6 (5028·3–5715·0)	98·7 (93·6–104·8)
Andorra	1·43 (1·32–1·54)	6·61 (6·09–7·23)	363·6 (338·1–395·1)	2382·8 (2199·5–2572·9)	4919·8 (4568·1–5331·3)	106·6 (96·2–119·8)
Austria	150 (141–160)	450 (421–481)	199·5 (182·7–217·5)	1433·8 (1347·5–1530·0)	3106·4 (2907·9–3328·4)	116·7 (104·8–127·7)
Belgium	338 (311–364)	887 (794–978)	162·5 (146·9–182·8)	2509·8 (2311·1–2697·7)	4959·2 (4470·8–5488·2)	97·6 (86·8–111·1)
Cyprus	38·7 (36·5–40·9)	119 (111–127)	207·5 (193·6–224·3)	4548·0 (4281·4–4802·6)	6080·2 (5693·1–6498·0)	33·7 (27·2–41·0)
Denmark	128 (120–136)	380 (356–412)	197·2 (184·7–215·6)	1874·0 (1753·2–2001·8)	4118·3 (3833·2–4449·3)	119·8 (111·5–134·4)
Finland	237 (222–258)	629 (590–675)	164·9 (155·9–179·2)	3716·4 (3477·1–4019·9)	7040·1 (6569·6–7535·2)	89·5 (81·5–101·0)
France	1390 (1310–1480)	3800 (3490–4120)	173·5 (155·7–189·9)	1864·6 (1743·0–1988·0)	3633·7 (3350·6–3989·1)	94·9 (82·6–106·0)
Germany	2130 (2010–2260)	7050 (6530–7690)	231·0 (211·1–251·3)	1907·3 (1800·4–2034·8)	4781·7 (4430·9–5241·8)	150·8 (135·4–165·7)
Greece	403 (372–436)	886 (807–960)	119·9 (106·7–131·7)	2921·2 (2694·1–3170·6)	5238·9 (4787·1–5716·0)	79·4 (69·0–88·9)
Iceland	5·83 (5·35–6·33)	23·0 (21·3–24·8)	294·1 (268·7–316·4)	2161·1 (1978·7–2336·9)	4740·9 (4388·3–5105·4)	119·5 (105·3–130·8)
Ireland	94·7 (87·7–103)	293 (269–317)	210·3 (192·0–230·4)	2476·9 (2291·1–2696·1)	4313·0 (3946·0–4640·2)	74·2 (64·4–85·6)
Israel	168 (158–179)	607 (561–659)	262·2 (238·6–282·1)	3506·0 (3305·5–3750·4)	5404·8 (4975·0–5878·1)	54·2 (43·6–63·2)
Italy	2580 (2390–2800)	5170 (4690–5740)	99·8 (92·0–112·1)	3228·5 (2991·9–3487·8)	4711·1 (4339·8–5151·7)	45·9 (40·7–52·3)
Luxembourg	12·6 (11·5–13·5)	44·0 (40·8–47·9)	250·4 (231·5–270·0)	2507·5 (2311·5–2690·4)	4759·3 (4423·0–5174·4)	89·8 (80·0–100·2)
Malta	11·3 (10·5–12·0)	52·6 (48·3–57·4)	367·4 (342·4–392·7)	2671·0 (2491·2–2852·3)	6903·2 (6363·2–7584·1)	158·5 (145·2–172·9)
Monaco	1·03 (0·950–1·11)	2·95 (2·72–3·22)	186·4 (169·7–204·1)	2029·6 (1875·3–2189·6)	4376·4 (4043·3–4749·7)	115·7 (103·6–127·8)
Netherlands	450 (416–483)	1160 (1060–1270)	157·7 (138·2–183·3)	2412·6 (2230·3–2580·0)	4169·6 (3829·6–4531·4)	72·9 (61·3–88·2)

Norway	197 (184–215)	373 (348–404)	89·2 (83·4–94·4)	3520·2 (3282·1–3811·4)	4689·7 (4343·5–5057·2)	33·2 (29·5–36·3)
Portugal	459 (430–489)	1390 (1280–1510)	201·8 (183·1–222·5)	3569·2 (3337·7–3791·7)	7417·3 (6844·0–8033·9)	107·8 (95·6–121·1)
San Marino	0·677 (0·626–0·727)	2·44 (2·24–2·68)	260·0 (245·6–276·0)	2288·3 (2113·8–2451·0)	4749·2 (4369·4–5179·8)	107·6 (100·2–116·1)
Spain	2070 (1930–2230)	5320 (4990–5620)	156·9 (140·7–171·5)	4066·4 (3811·6–4365·7)	6859·2 (6411·7–7251·6)	68·8 (58·5–78·4)
Sweden	390 (357–421)	831 (752–903)	113·2 (103·3–124·1)	3199·2 (2930·1–3442·3)	5100·6 (4665·7–5520·3)	59·4 (52·3–67·4)
Switzerland	334 (310–362)	990 (902–1070)	196·4 (180·1–215·6)	3620·2 (3353·6–3925·2)	6906·5 (6302·6–7433·7)	90·9 (80·1–101·6)
UK	2360 (2190–2540)	7570 (7040–8080)	220·8 (210·8–233·0)	3052·0 (2830·7–3285·1)	7751·5 (7183·4–8262·7)	154·1 (145·0–164·8)
**Latin America and Caribbean**	**11900 (11200–12700)**	**43000 (40200–46200)**	**261·0 (252·3–268·2)**	**4667·7 (4391·4–5001·2)**	**6745·2 (6305·5–7228·1)**	**44·5 (41·4–47·2)**
Andean Latin America	560 (526–600)	3090 (2890–3280)	451·7 (434·2–474·5)	2414·8 (2265·3–2611·1)	5010·8 (4688·3–5333·7)	107·6 (99·9–116·7)
Bolivia	105 (96·9–113)	611 (563–662)	481·4 (456·7–519·2)	2877·4 (2647·4–3107·9)	6084·9 (5601·3–6613·7)	111·5 (102·1–124·6)
Ecuador	187 (173–203)	1220 (1130–1310)	553·8 (514·6–599·8)	3131·1 (2879·3–3413·9)	7322·8 (6733·2–7833·3)	134·1 (119·7–150·8)
Peru	266 (246–291)	1250 (1150–1360)	370·0 (341·9–410·1)	1968·1 (1805·0–2168·4)	3592·0 (3301·1–3901·9)	82·7 (71·6–97·0)
Caribbean	1370 (1300–1460)	4740 (4450–5070)	245·4 (235·5–257·4)	4943·9 (4683·3–5272·0)	8883·6 (8321·1–9487·1)	79·7 (74·9–85·4)
Antigua and Barbuda	3·17 (2·94–3·47)	11·4 (10·3–12·3)	258·2 (229·3–293·1)	6153·5 (5665·0–6761·7)	10382·6 (9459·6–11241·9)	68·9 (54·0–84·2)
The Bahamas	9·68 (8·92–10·4)	42·1 (38·2–45·2)	334·7 (307·9–360·5)	5396·9 (4944·9–5859·9)	9560·2 (8697·4–10280·5)	77·2 (64·5–88·6)
Barbados	16·4 (15·1–17·7)	45·7 (42·6–48·7)	178·9 (162·5–202·9)	6014·5 (5520·8–6518·6)	9743·9 (9022·1–10404·4)	62·2 (52·7–75·5)
Belize	4·61 (4·28–4·94)	29·6 (27·2–32·6)	541·8 (490·9–593·7)	4409·1 (4066·1–4747·6)	8481·5 (7760·1–9378·2)	92·5 (76·5–109·4)
Bermuda	2·21 (2·03–2·38)	6·97 (6·44–7·64)	215·4 (189·0–240·0)	3422·7 (3143·4–3685·9)	6109·9 (5687·7–6683·9)	78·7 (64·5–92·8)
Cuba	388 (357–419)	1080 (996–1160)	179·5 (159·3–201·6)	3680·2 (3373·7–3975·6)	6174·4 (5704·5–6627·8)	67·9 (55·9–80·0)
Dominica	4·21 (3·94–4·54)	9·62 (8·73–10·4)	128·5 (113·1–143·7)	6297·8 (5894·4–6842·4)	10976·4 (9964·2–11891·4)	74·4 (61·9–86·5)
Dominican Republic	181 (168–196)	929 (846–1010)	413·5 (380·1–446·6)	4074·4 (3743·5–4437·4)	8951·5 (8168·4–9739·0)	119·8 (104·4–133·7)
Grenada	4·45 (4·12–4·77)	13·7 (12·5–15·0)	207·7 (186·0–230·0)	6508·8 (5965·1–6996·1)	11385·1 (10473·4–12427·8)	75·0 (61·8–87·3)
Guyana	40·2 (36·5–43·6)	114 (106–122)	182·8 (167·9–201·4)	8800·4 (7991·7–9639·1)	15515·3 (14486·0–16602·0)	76·5 (66·5–88·5)
Haiti	246 (226–263)	998 (907–1090)	305·9 (282·0–329·5)	6414·6 (5860·2–6897·2)	10966·8 (9908·6–12102·6)	71·0 (60·5–81·0)
Jamaica	83·4 (78·3–89·1)	244 (223–264)	192·2 (171·9–212·4)	4663·0 (4359·6–4988·8)	7891·5 (7239·7–8538·9)	69·3 (57·9–80·7)
Puerto Rico	230 (213–249)	650 (599–711)	182·3 (165·2–201·8)	6368·7 (5883·8–6890·1)	10991·1 (10090·4–12111·1)	72·6 (63·1–85·8)
Saint Kitts and Nevis	2·21 (2·04–2·36)	7·68 (6·91–8·40)	248·3 (221·9–272·5)	6239·9 (5733·1–6756·1)	10054·4 (9148·3–10952·0)	61·2 (48·1–72·3)
Saint Lucia	7·64 (7·11–8·17)	27·2 (25·2–28·8)	256·0 (229·5–278·6)	8270·6 (7679·0–8894·9)	11903·5 (11093·6–12610·6)	44·1 (33·6–53·9)

Saint Vincent and the Grenadines	5·60 (5·22–5·97)	17·1 (15·8–18·3)	206·3 (184·9–224·9)	7470·9 (6941·8–7984·0)	12148·7 (11253·9–12951·7)	62·7 (52·0–72·4)
Suriname	16·2 (14·7–17·4)	77·0 (70·7–83·8)	377·4 (343·4–419·2)	5643·5 (5123·8–6087·8)	11794·7 (10854·0–12780·7)	109·3 (95·3–127·3)
Trinidad and Tobago	76·2 (70·7–81·1)	254 (234–272)	233·8 (208·9–260·1)	8525·4 (7902·2–9046·4)	13331·5 (12356·9–14290·5)	56·5 (44·5–68·7)
Virgin Islands	6·51 (5·93–7·11)	22·1 (20·3–24·1)	239·7 (216·7–262·8)	6923·7 (6315·3–7561·8)	13195·8 (12185·8–14235·0)	90·7 (77·2–103·1)
Central Latin America	5850 (5500–6250)	20900 (19600–22400)	257·9 (249·3–267·0)	5833·7 (5491·4–6249·0)	8009·9 (7516·3–8579·2)	37·3 (33·8–40·4)
Colombia	893 (818–984)	3380 (3140–3610)	279·3 (251·0–312·5)	4203·1 (3858·4–4645·5)	6108·6 (5673·3–6529·8)	45·6 (33·3–57·8)
Costa Rica	83·5 (77·3–91·5)	414 (388–447)	396·3 (364·3–424·1)	4136·2 (3806·6–4546·4)	7544·8 (7067·4–8138·9)	82·6 (72·2–94·0)
El Salvador	109 (101–119)	412 (374–453)	277·5 (255·7–301·5)	3226·1 (2972·1–3539·4)	6678·9 (6063·1–7353·7)	107·1 (95·5–121·5)
Guatemala	161 (151–173)	1130 (1030–1220)	602·2 (553·5–649·8)	3580·6 (3348·2–3853·7)	9212·1 (8429·5–9898·0)	157·4 (140·8–175·2)
Honduras	107 (98·5–116)	636 (587–692)	494·2 (459·0–531·0)	4299·9 (3920·0–4648·7)	8416·2 (7701·7–9210·2)	95·9 (84·0–108·8)
Mexico	3890 (3610–4170)	11900 (11000–12900)	206·7 (199·5–214·5)	7575·7 (7032·0–8178·8)	9065·2 (8395·4–9841·6)	19·7 (16·7–22·4)
Nicaragua	87·4 (81·2–93·7)	451 (414–485)	415·8 (385·4–452·0)	4628·7 (4288·4–4985·2)	8295·7 (7657·4–8906·5)	79·3 (68·7–92·8)
Panama	64·1 (59·0–68·9)	319 (291–348)	397·3 (365·8–434·9)	3796·8 (3485·8–4088·0)	7171·9 (6542·4–7834·5)	89·0 (76·5–104·1)
Venezuela	453 (417–484)	2130 (1950–2320)	371·4 (329·7–411·6)	3866·4 (3578·7–4127·3)	6771·7 (6216·9–7356·3)	75·3 (61·1–90·3)
Tropical Latin America	4150 (3870–4460)	14300 (13100–15600)	245·0 (233·7–258·8)	4003·2 (3726·9–4321·1)	5451·0 (4997·9–5950·1)	36·2 (31·6–40·8)
Brazil	4060 (3790–4380)	13900 (12700–15200)	242·0 (230·6–255·7)	4016·9 (3731·9–4340·2)	5423·7 (4966·1–5929·5)	35·0 (30·4–39·6)
Paraguay	84·9 (77·3–92·4)	414 (383–442)	388·5 (353·6–424·5)	3445·0 (3120·6–3762·1)	6607·9 (6133·3–7053·0)	92·1 (77·3–105·9)
**North Africa and Middle East**	**7050 (6600–7490)**	**49900 (47000–53000)**	**608·2 (590·2–626·3)**	**3553·3 (3291·9–3790·3)**	**9288·7 (8742·9–9864·2)**	**161·5 (154·3–168·7)**
North Africa and Middle East	7050 (6600–7490)	49900 (47000–53000)	608·2 (590·2–626·3)	3553·3 (3291·9–3790·3)	9288·7 (8742·9–9864·2)	161·5 (154·3–168·7)
Afghanistan	411 (375–452)	2920 (2690–3110)	611·7 (559·2–652·0)	5405·3 (4946·9–5925·6)	14609·5 (13535·9–15523·2)	170·5 (152·4–183·7)
Algeria	561 (514–605)	4030 (3730–4300)	618·3 (570·4–671·5)	3993·1 (3620·8–4318·4)	10044·5 (9335·0–10739·5)	151·9 (133·0–172·6)
Bahrain	16·6 (15·2–17·8)	241 (223–258)	1350·3 (1253·7–1439·0)	6880·0 (6376·2–7360·0)	14986·1 (14117·7–15828·7)	118·0 (104·2–131·5)
Egypt	744 (690–801)	6500 (6000–7070)	774·0 (725·3–826·0)	2184·2 (2018·0–2350·9)	8390·9 (7720·9–9158·4)	284·3 (262·7–305·9)
Iran	887 (826–975)	5960 (5440–6490)	572·3 (550·0–591·8)	2880·3 (2660·5–3175·7)	6923·7 (6313·6–7525·6)	140·4 (133·2–145·8)
Iraq	631 (580–674)	4790 (4450–5090)	659·0 (607·6–713·3)	6878·3 (6263·4–7403·7)	15298·1 (14317·4–16224·4)	122·6 (108·3–139·0)
Jordan	111 (103–118)	1260 (1170–1350)	1031·4 (967·2–1093·7)	6860·0 (6352·4–7270·6)	13464·4 (12577·3–14491·0)	96·4 (84·9–107·1)
Kuwait	67·3 (61·8–73·1)	665 (604–725)	889·3 (826·7–956·3)	7270·2 (6782·2–7868·7)	15200·7 (14080·9–16270·5)	109·2 (95·2–125·4)

Lebanon	127 (117–138)	612 (569–649)	380·7 (348·7–415·6)	5221·3 (4763·5–5670·6)	11124·1 (10344·9–11825·3)	113·2 (98·3–129·2)
Libya	86·7 (79·2–93·9)	699 (647–762)	707·7 (658·3–760·9)	3945·8 (3602·2–4272·4)	10632·9 (9856·1–11633·6)	169·7 (152·7–186·1)
Morocco	759 (696–826)	5000 (4640–5340)	559·7 (521·9–595·3)	4868·2 (4433·8–5352·0)	13761·7 (12748·7–14709·2)	183·0 (164·2–198·5)
Oman	41·5 (38·4–44·1)	281 (260–313)	578·1 (537·5–628·8)	4407·1 (4043·3–4696·8)	8934·8 (8281·8–9770·5)	102·8 (90·8–114·7)
Palestine	43·1 (40·4–46·0)	300 (279–322)	595·9 (546·6–643·0)	4450·2 (4134·9–4766·0)	9776·8 (9116·0–10494·5)	119·8 (104·3–134·6)
Qatar	12·7 (11·7–13·8)	308 (277–340)	2324·4 (2145·2–2569·0)	7037·4 (6538·3–7561·7)	15139·8 (13988·7–16209·3)	115·3 (102·4–137·1)
Saudi Arabia	407 (375–431)	3510 (3250–3750)	762·0 (706·8–819·2)	5253·3 (4765·2–5579·2)	11315·6 (10569·2–12019·2)	115·5 (102·1–130·4)
Sudan	364 (337–390)	1960 (1810–2090)	437·8 (407·6–473·3)	3402·2 (3084·9–3693·2)	7873·0 (7233·0–8443·7)	131·6 (117·1–148·5)
Syria	263 (242–283)	1220 (1130–1310)	365·4 (341·2–389·8)	4152·4 (3771·4–4484·8)	8718·2 (8086·9–9298·5)	110·1 (98·8–122·0)
Tunisia	204 (189–221)	1360 (1230–1490)	563·4 (512·7–602·0)	3663·1 (3364·8–3958·6)	9842·8 (8954·1–10778·4)	168·8 (148·6–185·1)
Türkiye	1110 (1040–1160)	6390 (5870–6970)	476·2 (440·2–520·6)	2781·5 (2599·1–2930·5)	6626·9 (6104·8–7227·0)	138·3 (122·3–156·9)
United Arab Emirates	37·3 (34·0–40·6)	822 (739–897)	2102·4 (1947·5–2277·9)	5155·0 (4741·9–5596·9)	9504·3 (8726·4–10270·1)	84·5 (72·0–95·3)
Yemen	165 (152–178)	1070 (980–1140)	548·2 (520·0–579·6)	2719·9 (2506·9–2936·0)	5878·4 (5384·5–6351·8)	116·2 (106·3–125·7)
**South Asia**	**22500 (20800–24400)**	**103000 (95300–111000)**	**356·7 (345·7–366·1)**	**3173·6 (2967·1–3456·8)**	**6067·5 (5642·9–6599·1)**	**91·2 (86·4–95·6)**
South Asia	22500 (20800–24400)	103000 (95300–111000)	356·7 (345·7–366·1)	3173·6 (2967·1–3456·8)	6067·5 (5642·9–6599·1)	91·2 (86·4–95·6)
Bangladesh	1940 (1820–2070)	11000 (10400–11800)	470·1 (436·2–499·8)	3135·6 (2925·6–3360·9)	7084·3 (6698·9–7586·4)	126·0 (112·2–138·6)
Bhutan	9·40 (8·57–9·98)	35·6 (33·2–37·8)	278·4 (260·1–301·7)	2777·5 (2546·9–2981·0)	5189·4 (4822·5–5541·9)	86·9 (77·4–99·1)
India	17900 (16500–19500)	78800 (72700–85900)	339·7 (328·3–350·3)	3152·8 (2935·7–3447·9)	5819·0 (5381·5–6350·4)	84·6 (79·7–89·4)
Nepal	412 (381–446)	1950 (1820–2070)	374·3 (347·5–405·1)	3475·6 (3224·0–3721·9)	7280·9 (6765·6–7746·3)	109·6 (98·3–123·2)
Pakistan	2200 (2040–2410)	10800 (9950–11800)	392·6 (373·3–415·3)	3265·7 (3025·5–3595·1)	7144·2 (6622·0–7791·0)	118·8 (110·5–128·8)
**Southeast Asia, east Asia, and Oceania**	**45000 (41300–48800)**	**159000 (150000–170000)**	**254·1 (240·2–264·9)**	**3401·6 (3135·7–3706·9)**	**5773·6 (5416·9–6159·3)**	**69·8 (62·6–78·1)**
East Asia	36600 (33400–39800)	122000 (114000–131000)	233·7 (218·3–247·8)	3572·8 (3282·0–3916·0)	6139·5 (5688·0–6597·7)	72·0 (62·5–83·4)
China	35500 (32400–38600)	118000 (110000–127000)	232·7 (217·0–247·2)	3590·8 (3293·7–3939·4)	6157·7 (5697·9–6625·2)	71·6 (62·0–83·5)
North Korea	501 (462–545)	1800 (1630–1960)	260·5 (242·1–283·7)	2732·7 (2520·1–2968·2)	5430·6 (4925·5–5915·8)	98·8 (89·3–111·1)
Taiwan (province of China)	621 (575–662)	2270 (2130–2430)	266·0 (240·8–288·5)	3479·5 (3244·5–3699·9)	5853·6 (5504·9–6247·7)	68·3 (59·2–78·1)
Oceania	228 (213–243)	1220 (1140–1310)	436·1 (406·5–460·3)	5819·0 (5413·1–6213·6)	12255·9 (11524·9–13025·5)	110·7 (100·3–119·3)
American Samoa	2·73 (2·55–2·97)	11·3 (10·5–12·0)	315·5 (292·0–339·3)	9088·8 (8460·6–9780·2)	21350·9 (19873·5–22715·4)	135·0 (123·9–147·1)

Cook Islands	1·43 (1·33–1·55)	4·54 (4·18–4·84)	216·5 (195·1–236·0)	10012·4 (9262·1–10808·6)	19310·3 (17832·0–20652·1)	93·0 (80·1–105·4)
Federated States of Micronesia	3·69 (3·46–3·94)	12·4 (11·7–13·3)	236·5 (218·5–254·3)	6278·9 (5836·9–6716·4)	14162·3 (13369·6–15007·3)	125·7 (113·6–135·8)
Fiji	36·1 (33·3–39·3)	140 (130–150)	288·3 (258·9–317·6)	7749·1 (7141·7–8333·9)	16262·0 (15192·5–17352·6)	110·1 (97·8–126·7)
Guam	4·78 (4·39–5·18)	16·4 (15·2–17·9)	244·0 (221·6–266·2)	4700·4 (4350·8–5103·2)	8639·3 (7990·9–9401·1)	83·9 (73·8–95·6)
Kiribati	3·44 (3·20–3·72)	13·2 (12·2–14·1)	283·7 (262·7–303·1)	7255·3 (6733·0–7737·7)	14377·8 (13466·0–15325·9)	98·3 (86·7–108·7)
Marshall Islands	2·34 (2·17–2·52)	10·8 (9·98–11·7)	361·1 (330·3–390·6)	10267·8 (9472·6–11039·4)	22173·9 (20687·2–23875·3)	116·1 (103·8–129·3)
Nauru	0·447 (0·412–0·487)	1·10 (1·03–1·18)	146·5 (131·1–159·9)	7866·8 (7278·0–8422·0)	15952·0 (14972·0–16853·4)	102·9 (92·9–114·4)
Niue	0·170 (0·158–0·180)	0·374 (0·346–0·399)	119·5 (106·5–131·4)	8130·6 (7534·0–8601·5)	18316·2 (16927·9–19688·6)	125·4 (112·3–138·4)
Northern Mariana Islands	1·59 (1·47–1·74)	5·84 (5·20–6·33)	266·9 (230·3–296·3)	4994·7 (4638·9–5362·1)	9830·9 (8899·3–10641·6)	96·9 (85·4–108·7)
Palau	0·959 (0·875–1·04)	4·14 (3·89–4·37)	331·9 (303·5–364·8)	8119·5 (7401·4–8771·6)	16965·0 (15938·4–17864·3)	109·2 (96·8–125·2)
Papua New Guinea	133 (124–143)	839 (779–906)	529·3 (491·7–565·5)	5365·6 (4966·4–5771·9)	11656·2 (10837·9–12448·3)	117·4 (104·0–128·8)
Samoa	7·65 (7·06–8·21)	27·4 (25·4–29·8)	258·0 (235·0–278·9)	7472·5 (6948·2–8020·1)	15753·5 (14655·1–17091·8)	110·9 (98·2–122·9)
Solomon Islands	7·38 (6·89–7·92)	39·5 (36·3–42·7)	436·1 (401·7–471·5)	3992·4 (3737·2–4262·7)	8811·0 (8215·4–9406·7)	120·8 (107·9–136·8)
Tokelau	0·117 (0·105–0·126)	0·258 (0·238–0·275)	121·7 (104·0–138·9)	8923·9 (8042·7–9658·1)	17608·4 (16240·6–18760·7)	97·6 (82·2–112·7)
Tonga	4·38 (4·06–4·69)	11·8 (11·1–12·5)	170·0 (156·6–185·2)	6980·5 (6510·2–7489·5)	13908·2 (13021·0–14762·3)	99·4 (90·0–110·3)
Tuvalu	0·392 (0·365–0·419)	1·27 (1·18–1·33)	223·7 (206·1–241·0)	5239·6 (4891·3–5566·7)	11242·8 (10480·3–11812·3)	114·7 (102·6–126·3)
Vanuatu	4·15 (3·83–4·49)	24·7 (23·0–26·3)	495·9 (456·5–531·6)	4818·0 (4499·8–5181·2)	11093·3 (10361·1–11842·0)	130·4 (116·5–143·7)
Southeast Asia	8210 (7660–8780)	36100 (33700–38600)	340·1 (327·0–350·5)	2833·2 (2618·1–3020·1)	5378·7 (5000·7–5734·8)	89·9 (84·7–95·0)
Cambodia	103 (95·0–110)	687 (632–726)	566·1 (519·7–620·2)	1951·3 (1813·0–2081·2)	5070·2 (4668·0–5409·6)	160·0 (143·6–180·2)
Indonesia	2800 (2610–3040)	11500 (10600–12600)	309·9 (294·3–325·3)	2400·7 (2211·6–2636·6)	4584·4 (4171·9–4972·2)	91·0 (85·3–97·5)
Laos	61·6 (57·6–65·9)	317 (294–338)	415·8 (388·7–445·0)	2625·8 (2422·5–2807·1)	6137·7 (5676·9–6584·8)	133·9 (120·4–149·3)
Malaysia	445 (412–488)	2220 (2090–2400)	399·2 (367·0–428·5)	4225·2 (3885·9–4581·3)	7416·0 (6945·0–7981·5)	75·6 (63·7–86·3)
Maldives	2·96 (2·71–3·22)	22·4 (20·6–24·5)	657·1 (607·2–723·6)	2872·5 (2581·4–3146·3)	5447·7 (4935·5–6004·7)	89·8 (76·5–104·0)
Mauritius	43·2 (40·4–47·1)	205 (189–220)	374·8 (346·2–406·5)	5369·7 (5019·1–5840·0)	11230·0 (10378·0–12024·1)	109·2 (97·0–124·1)
Myanmar	969 (890–1040)	4080 (3850–4350)	321·9 (297·4–343·0)	3768·1 (3445·1–4050·1)	7994·7 (7473·1–8523·7)	112·4 (100·2–123·5)
Philippines	1210 (1120–1290)	3940 (3660–4290)	225·9 (212·9–238·7)	3427·6 (3171·0–3721·8)	4423·8 (4070·2–4785·7)	29·1 (24·3–33·8)

Seychelles	2·06 (1·88–2·21)	13·2 (12·1–14·3)	543·6 (501·3–592·7)	3543·5 (3228·3–3807·0)	11029·9 (10182·1–11808·3)	211·5 (193·5–230·7)
Sri Lanka	526 (480–571)	2770 (2590–2990)	428·0 (397·6–460·7)	4550·5 (4105·6–4985·6)	10297·2 (9694·7–11089·3)	126·6 (111·9–139·8)
Thailand	1050 (970–1120)	5760 (5180–6320)	448·3 (405·9–488·7)	2612·8 (2402·1–2808·8)	5447·8 (4917·5–5948·0)	108·6 (91·7–122·5)
Timor-Leste	6·68 (6·22–7·13)	53·7 (49·0–57·6)	705·1 (656·1–748·5)	1815·1 (1690·9–1967·9)	5902·3 (5336·6–6332·5)	225·3 (206·7–243·7)
Viet Nam	981 (894–1050)	4530 (4290–4760)	362·2 (330·4–389·8)	2292·0 (2071·4–2487·8)	4518·3 (4259·8–4781·9)	97·4 (82·6–112·3)
**Sub-Saharan Africa**	**6050 (5630–6440)**	**26900 (25300–28700)**	**345·4 (336·2–354·0)**	**2323·4 (2154·5–2484·2)**	**4247·5 (3947·0–4530·7)**	**82·9 (78·6–86·5)**
Central sub-Saharan Africa	784 (726–845)	4120 (3790–4420)	426·0 (405·2–446·2)	2766·9 (2566·3–2970·8)	5263·8 (4847·9–5638·8)	90·3 (84·5–97·2)
Angola	170 (156–184)	1060 (980–1150)	525·9 (490·1–557·8)	3282·9 (3014·0–3517·1)	6198·2 (5686·2–6707·5)	88·9 (79·1–98·3)
Central African Republic	54·6 (50·5–58·8)	230 (212–247)	320·8 (301·6–344·3)	3679·1 (3369·9–3946·8)	7086·4 (6557·0–7569·2)	92·8 (82·8–104·4)
Congo (Brazzaville)	36·3 (32·8–39·5)	209 (192–226)	476·0 (443·8–512·4)	2788·8 (2510·2–3012·8)	5709·0 (5223·8–6130·5)	104·9 (93·0–118·7)
DR Congo	496 (457–536)	2480 (2260–2650)	400·2 (375·1–426·9)	2547·6 (2350·1–2749·9)	4760·7 (4339·0–5125·6)	86·9 (78·2–95·6)
Equatorial Guinea	6·74 (6·19–7·18)	49·8 (46·0–53·8)	639·5 (601·7–673·7)	2881·4 (2667·9–3078·4)	6377·6 (5764·2–6841·7)	121·4 (109·1–130·8)
Gabon	20·3 (18·7–21·9)	91·0 (84·8–97·7)	347·8 (320·1–378·2)	3251·2 (3008·1–3500·3)	6789·1 (6359·8–7259·5)	109·0 (95·5–121·6)
Eastern sub-Saharan Africa	1740 (1620–1850)	6790 (6360–7210)	289·6 (280·4–299·5)	1823·6 (1694·0–1949·7)	2921·9 (2716·9–3097·0)	60·3 (55·9–64·4)
Burundi	53·6 (49·5–57·4)	192 (179–206)	259·1 (241·8–282·0)	1821·5 (1674·4–1942·7)	2921·5 (2697·2–3118·5)	60·4 (51·2–71·0)
Comoros	6·10 (5·66–6·53)	25·5 (23·6–27·3)	317·5 (295·1–341·0)	2363·8 (2182·7–2547·9)	4399·8 (4056·4–4702·9)	86·2 (75·9–97·8)
Djibouti	3·42 (3·17–3·62)	25·8 (23·9–27·7)	654·0 (613·6–694·8)	1600·4 (1473·1–1718·4)	3085·3 (2819·3–3283·1)	92·9 (82·4–102·1)
Eritrea	29·9 (27·4–31·9)	165 (154–177)	452·9 (427·6–482·3)	2132·0 (1960·8–2296·4)	4064·6 (3772·8–4362·4)	90·8 (78·7–103·5)
Ethiopia	642 (594–691)	2010 (1870–2150)	213·5 (204·2–223·7)	2493·8 (2299·3–2714·7)	3354·5 (3130·5–3611·9)	34·5 (30·4–38·8)
Kenya	151 (140–160)	622 (580–664)	312·9 (302·0–324·8)	1401·7 (1289·4–1506·6)	2046·5 (1908·0–2200·3)	46·0 (41·5–49·2)
Madagascar	103 (95·7–111)	434 (404–468)	320·7 (300·0–340·1)	1611·9 (1473·8–1733·6)	2751·5 (2530·9–2959·3)	70·8 (61·5–80·1)
Malawi	67·6 (63·0–72·3)	215 (202–229)	218·9 (202·9–238·1)	1409·8 (1309·7–1502·5)	2104·1 (1956·5–2253·9)	49·3 (41·6–59·3)
Mozambique	109 (99·6–116)	506 (462–552)	365·8 (342·7–404·3)	1542·4 (1413·2–1647·4)	3274·8 (2956·8–3557·7)	112·4 (101·5–132·4)
Rwanda	59·2 (55·5–63·6)	187 (172–199)	215·5 (200·3–233·2)	1608·5 (1487·5–1714·3)	2313·1 (2125·1–2474·9)	43·9 (36·3–51·8)
Somalia	62·4 (57·3–66·9)	328 (303–352)	425·1 (399·3–455·3)	1808·4 (1663·0–1953·1)	3155·2 (2902·3–3382·0)	74·6 (64·3–83·5)
South Sudan	48·4 (44·5–52·2)	143 (132–153)	195·4 (184·4–207·7)	1602·4 (1471·2–1731·5)	2856·3 (2622·5–3062·1)	78·3 (70·6–87·2)
Tanzania	182 (168–193)	862 (812–916)	374·4 (346·1–395·8)	1356·1 (1259·4–1434·8)	2625·1 (2427·3–2803·0)	93·7 (82·7–104·3)
Uganda	129 (119–137)	580 (538–617)	348·7 (327·7–374·6)	1582·6 (1449·6–1696·5)	2823·4 (2587·4–3019·2)	78·5 (68·2–90·3)

Zambia	95·1<br>(88·3–101)	485<br>(451–524)	409·7<br>(381·7–433·1)	2549·2<br>(2368·1–2726·0)	4619·2<br>(4277·1–4943·7)	81·3<br>(70·0–91·1)
Southern sub-Saharan Africa	912<br>(852–982)	3640<br>(3410–3910)	299·5<br>(283·4–314·0)	2852·3<br>(2652·9–3094·8)	5602·3<br>(5225·5–6018·8)	96·5<br>(87·4–103·2)
Botswana	16·2<br>(15·0–17·5)	85·2<br>(79·3–90·3)	426·9<br>(396·9–454·3)	2395·5<br>(2202·4–2607·3)	4869·4<br>(4521·4–5107·2)	103·4<br>(90·1–115·0)
Eswatini	10·4<br>(9·63–11·2)	44·9<br>(41·6–48·0)	332·8<br>(306·2–359·8)	2895·6<br>(2675·7–3169·6)	6412·2<br>(5938·6–6822·8)	121·7<br>(107·5–136·7)
Lesotho	20·2<br>(18·3–21·8)	72·1<br>(67·1–77·5)	257·6<br>(231·4–280·6)	1847·9<br>(1683·9–2006·6)	4883·5<br>(4547·2–5255·8)	164·6<br>(143·6–183·6)
Namibia	19·4<br>(17·8–21·1)	69·7<br>(64·1–75·5)	260·4<br>(241·2–280·3)	2391·8<br>(2193·3–2622·5)	4124·2<br>(3726·6–4500·1)	72·6<br>(62·6–84·0)
South Africa	742<br>(691–806)	2980<br>(2790–3210)	302·3<br>(281·0–318·1)	3076·4<br>(2863·5–3372·3)	5878·3<br>(5464·8–6315·5)	91·2<br>(81·1–98·3)
Zimbabwe	104<br>(94·4–112)	387<br>(359–414)	271·9<br>(253·1–299·6)	2101·9<br>(1901·9–2261·5)	4347·0<br>(3997·4–4644·8)	106·9<br>(95·6–123·8)
Western sub-Saharan Africa	2610<br>(2420–2780)	12400<br>(11600–13200)	374·6<br>(363·2–385·2)	2467·6<br>(2275·2–2643·6)	4678·0<br>(4348·9–4982·2)	89·6<br>(84·8–93·7)
Benin	72·5<br>(66·1–77·6)	478<br>(448–509)	558·9<br>(525·5–598·7)	2963·2<br>(2669·0–3166·1)	6577·3<br>(6146·8–7059·6)	122·1<br>(110·2–135·9)
Burkina Faso	99·8<br>(91·7–107)	565<br>(533–598)	466·0<br>(441·2–497·1)	1986·5<br>(1833·7–2132·2)	4407·0<br>(4106·3–4711·6)	122·0<br>(110·8–134·2)
Cabo Verde	6·99<br>(6·48–7·50)	35·7<br>(32·7–38·1)	410·4<br>(382·6–445·4)	2921·5<br>(2675·7–3136·4)	6994·7<br>(6489·1–7487·7)	139·6<br>(125·7–156·3)
Cameroon	133<br>(123–141)	888<br>(829–946)	570·0<br>(527·6–610·6)	2415·5<br>(2222·8–2581·3)	5034·1<br>(4724·3–5359·5)	108·6<br>(95·2–122·6)
Chad	78·8<br>(73·1–84·8)	399<br>(369–428)	406·3<br>(376·0–433·0)	2386·4<br>(2207·7–2567·3)	4890·2<br>(4488·1–5242·8)	105·0<br>(94·0–114·5)
Côte d'Ivoire	159<br>(146–170)	876<br>(814–937)	452·5<br>(423·7–487·3)	2752·1<br>(2539·6–2957·0)	5529·9<br>(5115·1–5915·8)	101·1<br>(88·6–113·4)
The Gambia	11·6<br>(10·7–12·4)	70·0<br>(64·4–74·7)	502·9<br>(471·2–539·9)	2431·1<br>(2239·8–2609·1)	5234·4<br>(4814·3–5635·1)	115·4<br>(103·9–128·3)
Ghana	207<br>(190–224)	1190<br>(1090–1270)	472·7<br>(447·0–504·3)	2563·4<br>(2313·4–2763·9)	5344·7<br>(4889·2–5715·4)	108·6<br>(98·1–120·0)
Guinea	83·6<br>(76·6–89·7)	323<br>(300–344)	286·4<br>(262·9–304·9)	2228·0<br>(2033·0–2378·1)	4413·4<br>(4077·7–4732·3)	98·2<br>(86·1–109·5)
Guinea-Bissau	15·9<br>(14·6–17·2)	65·1<br>(60·6–69·5)	309·5<br>(293·2–333·9)	3041·8<br>(2770·1–3270·7)	5962·0<br>(5551·9–6312·1)	96·1<br>(87·4–107·2)
Liberia	33·1<br>(30·6–35·4)	177<br>(162–188)	435·1<br>(405·4–474·8)	2697·3<br>(2489·9–2889·9)	5797·3<br>(5333·9–6181·3)	115·1<br>(104·0–129·9)
Mali	210<br>(193–225)	1060<br>(973–1140)	405·6<br>(375·8–431·5)	4331·6<br>(3968·9–4655·4)	8781·8<br>(8029·8–9442·1)	102·8<br>(91·2–113·8)
Mauritania	24·5<br>(22·6–26·1)	91·0<br>(85·8–96·2)	271·2<br>(252·0–290·8)	2082·2<br>(1907·3–2226·9)	3440·9<br>(3228·6–3656·7)	65·4<br>(56·1–74·7)
Niger	93·0<br>(86·4–100)	550<br>(508–588)	491·8<br>(458·5–521·7)	2486·9<br>(2290·4–2669·5)	4856·0<br>(4445·8–5255·5)	95·3<br>(83·4–106·6)
Nigeria	1150<br>(1070–1240)	4490<br>(4180–4790)	290·3<br>(280·6–300·3)	2227·4<br>(2065·6–2428·0)	3713·2<br>(3446·3–4005·0)	66·7<br>(61·9–70·5)
São Tomé and Príncipe	1·97<br>(1·82–2·13)	8·60<br>(7·96–9·18)	336·6<br>(312·3–367·5)	2758·9<br>(2533·4–2968·6)	5862·5<br>(5435·4–6259·9)	112·6<br>(101·6–125·4)
Senegal	143<br>(132–153)	683<br>(643–724)	378·3<br>(354·1–405·9)	3630·1<br>(3336·0–3889·3)	7160·4<br>(6728·4–7607·4)	97·4<br>(86·1–111·6)
Sierra Leone	54·7<br>(50·3–59·5)	262<br>(239–282)	380·1<br>(354·3–408·8)	2456·0<br>(2250·5–2667·8)	5101·6<br>(4660·1–5466·3)	107·8<br>(96·3–120·4)
Togo	33·0<br>(30·4–35·5)	185<br>(172–195)	460·9<br>(436·3–494·6)	1921·8<br>(1752·0–2059·4)	3601·6<br>(3350·9–3815·0)	87·5<br>(79·0–97·0)

Table S23. Prevalence counts and age-standardised rates per 100,000 population and the corresponding percentage change between 2021 and 2050 for diabetes globally, in 21 Global Burden of Disease regions and all countries

95% confidence intervals in parentheses

Location	Prevalence 2021, number (thousands)	Prevalence 2050, number (thousands)	Prevalence percent change 2021 - 2050, number (%)	Prevalence 2021, rate (per 100,000)	Prevalence 2050, rate (per 100,000)	Prevalence percent change 2021 - 2050, rate (%)
**Global**	**529000 (500000–564000)**	**1310000 (1220000–1390000)**	**147·0 (131·1–164·7)**	**6138·6 (5800·3–6536·5)**	**9802·0 (9382·0–10207·0)**	**59·7 (54·7–66·0)**
**Central Europe, eastern Europe, and central Asia**	**27500 (25700–29600)**	**43300 (39400–47900)**	**57·5 (45·7–69·2)**	**4548·4 (4251·4–4891·2)**	**6280·7 (5951·6–6615·2)**	**38·1 (34·4–41·7)**
Central Asia	4790 (4510–5100)	10500 (9120–12000)	119·6 (90·4–149·5)	5314·8 (4994·7–5661·3)	6892·6 (6511·5–7253·8)	29·7 (26·6–33·1)
Armenia	197 (180–217)	322 (251–418)	63·4 (28·6–112·2)	4793·6 (4364·2–5274·2)	6028·4 (5589·8–6514·1)	25·8 (22·0–28·8)
Azerbaijan	555 (511–601)	1230 (1040–1390)	121·1 (95·7–157·2)	4837·1 (4456·9–5226·8)	6660·2 (6264·5–7092·5)	37·8 (33·9–42·5)
Georgia	294 (271–319)	336 (240–478)	14·2 (-13·9–60·8)	5703·4 (5244·6–6187·3)	6860·9 (6393·6–7365·1)	20·3 (17·8–22·7)
Kazakhstan	1230 (1150–1310)	2440 (1880–3110)	98·4 (54·2–154·1)	6304·8 (5887·5–6702·8)	8203·1 (7773·8–8650·1)	30·1 (27·0–33·1)
Kyrgyzstan	228 (211–248)	587 (458–750)	157·4 (105·6–233·5)	4070·1 (3748·7–4425·6)	5867·7 (5494·8–6269·6)	44·3 (38·4–49·0)
Mongolia	129 (119–139)	207 (181–241)	60·7 (40·6–89·1)	3975·8 (3689·5–4263·9)	4568·7 (4279·8–4862·1)	14·9 (13·0–16·8)
Tajikistan	299 (276–324)	982 (763–1250)	228·4 (154·2–321·3)	4120·8 (3757·8–4460·6)	5650·6 (5309·5–5981·4)	37·2 (33·9–41·5)
Turkmenistan	192 (175–210)	434 (347–527)	126·7 (83·0–185·2)	3956·6 (3605·9–4358·7)	4826·5 (4453·6–5233·0)	22·0 (19·4–24·7)
Uzbekistan	1750 (1620–1900)	4070 (3270–5090)	132·6 (84·4–188·6)	5683·3 (5270·3–6176·2)	7479·6 (7018·6–7938·6)	31·7 (28·1–34·9)
Central Europe	10200 (9540–10900)	14200 (13000–15300)	38·8 (30·2–48·1)	5147·7 (4833·5–5516·0)	7014·8 (6643·2–7395·8)	36·3 (32·3–39·9)
Albania	148 (131–165)	215 (174–267)	45·1 (20·8–79·3)	3586·5 (3176·5–3956·0)	4609·5 (4159·7–4996·0)	28·6 (24·9–33·3)
Bosnia and Herzegovina	398 (369–424)	536 (434–662)	34·8 (6·2–70·2)	6883·0 (6397·5–7325·6)	9101·9 (8663·2–9623·5)	32·3 (29·7–35·7)
Bulgaria	717 (671–767)	736 (640–835)	2·7 (-9·6–14·0)	5614·2 (5266·4–5983·8)	6807·4 (6451·7–7184·1)	21·3 (19·1–23·4)
Croatia	402 (370–439)	552 (471–642)	37·1 (21·3–57·8)	5122·7 (4720·5–5633·1)	7505·4 (7062·4–8062·0)	46·6 (41·2–51·3)
Czechia	1060 (970–1150)	1620 (1400–1840)	52·9 (31·4–70·3)	5501·2 (5040·8–5924·7)	7472·7 (7009·8–7897·4)	35·9 (32·5–39·4)
Hungary	908 (829–994)	1300 (1110–1490)	43·2 (25·5–61·9)	5225·2 (4750·0–5697·5)	7765·7 (7252·3–8330·0)	48·7 (42·7–54·4)
Montenegro	56·6 (51·8–61·9)	93·1 (72·5–117)	64·7 (31·3–102·4)	5909·5 (5425·4–6435·0)	8646·9 (8021·6–9211·5)	46·4 (41·6–52·3)
North Macedonia	230 (210–248)	365 (308–421)	58·8 (34·5–86·5)	6941·9 (6370·6–7480·5)	9497·4 (8860·8–10045·7)	36·9 (33·5–41·0)
Poland	3590 (3280–3950)	4840 (4380–5340)	34·9 (26·6–45·0)	5455·6 (5018·4–5991·8)	6751·2 (6271·4–7313·0)	23·8 (21·2–26·4)
Romania	1210 (1110–1320)	1600 (1290–1870)	32·0 (12·8–51·4)	3646·1 (3355·0–3955·3)	5269·9 (4937·8–5584·0)	44·6 (38·8–50·1)
Serbia	958 (880–1040)	1470 (1230–1750)	53·3 (28·6–84·0)	6388·5 (5879·4–6988·0)	10294·5 (9713·0–10863·9)	61·3 (54·3–66·8)

Slovakia	374 (340–408)	590 (525–654)	58·1 (40·9–72·4)	4176·7 (3821·8–4541·9)	5716·0 (5385·5–6102·3)	36·9 (32·7–40·9)
Slovenia	162 (149–178)	257 (222–295)	58·2 (40·1–76·4)	4193·9 (3853·9–4575·2)	6103·3 (5725·2–6560·7)	45·6 (39·6–50·1)
Eastern Europe	12500 (11500–13600)	18600 (16400–20900)	49·0 (33·9–63·7)	3854·8 (3555·3–4183·3)	5356·2 (4971·1–5724·0)	39·0 (34·8–44·4)
Belarus	402 (362–436)	687 (586–808)	70·8 (42·6–96·6)	2727·3 (2467·4–2960·2)	4427·2 (4108·6–4708·5)	62·5 (54·2–71·7)
Estonia	94·7 (85·8–104)	125 (99·2–151)	32·3 (8·7–62·3)	4360·5 (3952·3–4757·0)	5696·0 (5260·9–6111·4)	30·7 (27·1–35·3)
Latvia	139 (129–153)	151 (127–181)	8·7 (-4·8–26·9)	4274·4 (3973·0–4664·4)	5520·2 (5181·7–5916·6)	29·2 (25·5–32·0)
Lithuania	170 (156–185)	201 (169–233)	18·7 (3·4–36·5)	3626·3 (3340·5–3921·2)	4836·7 (4541·9–5147·3)	33·5 (28·9–37·9)
Moldova	264 (239–286)	413 (334–511)	56·4 (31·1–89·7)	4876·2 (4419·5–5254·8)	6865·2 (6386·4–7324·6)	40·9 (35·8–45·9)
Russia	8930 (8230–9780)	14100 (12400–15900)	58·5 (42·0–73·9)	3957·5 (3650·7–4329·2)	5568·3 (5172·2–5926·4)	40·8 (36·0–46·7)
Ukraine	2510 (2270–2720)	2910 (2380–3520)	15·9 (-3·0–32·3)	3672·8 (3309·7–3985·5)	4769·1 (4368·2–5128·9)	29·9 (26·5–33·8)
**High income**	**120000 (114000–126000)**	**177000 (169000–187000)**	**47·7 (42·4–53·0)**	**6761·3 (6464·4–7081·6)**	**8704·0 (8367·5–9063·6)**	**28·8 (26·1–31·2)**
Australasia	1780 (1640–1900)	4220 (3750–4750)	137·2 (109·9–160·3)	3907·5 (3626·0–4147·8)	6396·5 (6010·3–6829·7)	63·8 (56·4–70·7)
Australia	1450 (1320–1570)	3640 (3150–4100)	150·7 (118·4–180·3)	3801·5 (3474·1–4071·6)	6386·1 (5954·2–6859·6)	68·1 (59·3–76·6)
New Zealand	326 (314–340)	578 (502–646)	77·2 (56·6–99·3)	4444·7 (4288·9–4640·8)	6281·6 (6037·8–6528·7)	41·3 (36·8–45·2)
High-income Asia Pacific	24800 (23400–26300)	29900 (28300–31600)	20·7 (17·3–25·0)	7311·9 (6898·8–7715·4)	8576·3 (8143·1–8980·4)	17·3 (15·4–19·2)
Brunei	58·4 (55·0–62·2)	157 (133–184)	168·3 (127·6–211·8)	13904·0 (13054·6–14873·9)	18914·5 (18002·3–19835·4)	36·1 (33·0–39·0)
Japan	15000 (13700–16200)	16000 (14700–17300)	6·9 (4·6–9·5)	5897·9 (5446·9–6377·4)	6703·6 (6259·4–7170·1)	13·7 (12·2–15·9)
Singapore	696 (646–743)	1610 (1390–1780)	131·4 (95·9–156·8)	8225·0 (7626·3–8753·1)	12185·9 (11524·1–12860·7)	48·2 (42·9–53·6)
South Korea	9030 (8530–9420)	12100 (11500–12900)	34·1 (27·8–41·8)	10346·1 (9782·9–10807·5)	11687·1 (11111·7–12134·5)	13·0 (11·8–14·0)
High-income North America	50300 (48000–52700)	70400 (66100–75400)	40·0 (32·6–47·8)	8748·7 (8405·1–9168·4)	10524·9 (10162·5–10963·8)	20·3 (18·7–22·1)
Canada	4170 (3770–4560)	5820 (5230–6270)	39·6 (29·0–49·9)	6627·4 (6010·8–7170·3)	7905·4 (7258·3–8431·8)	19·3 (16·8–21·9)
Greenland	2·83 (2·58–3·04)	4·66 (4·23–5·12)	64·7 (51·1–80·6)	3865·5 (3560·9–4149·8)	4827·2 (4502·1–5137·3)	24·9 (22·5–27·7)
USA	46300 (44000–48700)	64700 (60600–69400)	39·9 (32·4–48·3)	9001·1 (8619·8–9462·1)	10842·0 (10443·8–11299·9)	20·5 (18·8–22·1)
Southern Latin America	4910 (4500–5290)	10600 (9850–11500)	116·7 (92·9–136·8)	5871·8 (5387·7–6325·8)	8591·8 (8102·8–9073·8)	46·4 (41·3–51·4)
Argentina	2970 (2700–3230)	6660 (6010–7320)	124·8 (95·1–152·0)	5519·2 (5013·0–5992·6)	8258·5 (7784·6–8792·4)	49·8 (43·8–55·5)
Chile	1670 (1530–1820)	3540 (3290–3870)	112·8 (97·4–126·5)	6675·4 (6126·1–7277·6)	9440·9 (8917·0–10049·2)	41·5 (37·4–45·8)
Uruguay	271 (247–293)	416 (365–469)	53·4 (37·6–74·2)	5568·0 (5099·4–5998·6)	7302·3 (6852·6–7729·1)	31·2 (27·1–35·3)
Western Europe	38100 (35800–40600)	61700 (58300–65900)	62·3 (54·8–71·7)	5378·6 (5028·3–5715·0)	7376·6 (6981·8–7761·6)	37·2 (32·6–42·2)

Andorra	6·61 (6·09–7·23)	11·5 (10·4–12·6)	73·9 (60·7–90·0)	4919·8 (4568·1–5331·3)	6581·5 (6147·6–7013·4)	33·8 (29·6–39·7)
Austria	450 (421–481)	864 (761–969)	92·2 (70·7–112·1)	3106·4 (2907·9–3328·4)	4385·3 (4144·0–4643·3)	41·2 (36·0–45·9)
Belgium	887 (794–978)	1560 (1440–1690)	76·3 (64·8–89·2)	4959·2 (4470·8–5488·2)	7049·6 (6508·3–7648·8)	42·2 (37·2–47·5)
Cyprus	119 (111–127)	302 (244–344)	153·9 (108·6–191·8)	6080·2 (5693·1–6498·0)	9184·3 (8668·5–9727·8)	51·1 (46·3–56·9)
Denmark	380 (356–412)	601 (554–652)	58·4 (48·0–69·0)	4118·3 (3833·2–4449·3)	5581·0 (5277·0–5968·2)	35·6 (31·6–39·4)
Finland	629 (590–675)	898 (811–977)	42·9 (31·3–53·4)	7040·1 (6569·6–7535·2)	9248·4 (8743·4–9779·4)	31·4 (28·2–35·4)
France	3800 (3490–4120)	6730 (6070–7440)	77·2 (61·0–95·8)	3633·7 (3350·6–3989·1)	5472·5 (5117·6–5873·2)	50·7 (44·8–56·5)
Germany	7050 (6530–7690)	10400 (9320–11600)	47·1 (32·0–62·1)	4781·7 (4430·9–5241·8)	6231·6 (5821·4–6684·6)	30·4 (26·9–33·5)
Greece	886 (807–960)	1370 (1190–1600)	55·3 (37·7–77·5)	5238·9 (4787·1–5716·0)	7657·4 (7178·8–8201·5)	46·3 (40·4–52·1)
Iceland	23·0 (21·3–24·8)	40·8 (35·6–47·1)	77·9 (51·7–106·0)	4740·9 (4388·3–5105·4)	5776·6 (5361·5–6129·7)	21·9 (19·6–25·0)
Ireland	293 (269–317)	584 (474–687)	99·3 (62·9–137·3)	4313·0 (3946·0–4640·2)	5533·1 (5171·3–5892·4)	28·3 (25·0–31·9)
Israel	607 (561–659)	1240 (1030–1470)	104·2 (67·7–149·1)	5404·8 (4975·0–5878·1)	6770·8 (6309·3–7249·1)	25·3 (22·8–28·3)
Italy	5170 (4690–5740)	8470 (7570–9360)	64·1 (48·9–79·8)	4711·1 (4339·8–5151·7)	7021·2 (6543·8–7487·5)	49·1 (44·0–55·8)
Luxembourg	44·0 (40·8–47·9)	120 (102–135)	173·8 (135·6–207·4)	4759·3 (4423·0–5174·4)	6989·0 (6550·8–7423·3)	46·9 (41·9–53·2)
Malta	52·6 (48·3–57·4)	89·3 (78·7–100)	69·8 (53·0–87·8)	6903·2 (6363·2–7584·1)	9952·9 (9313·2–10633·8)	44·3 (39·6–49·4)
Monaco	2·95 (2·72–3·22)	3·06 (2·78–3·44)	3·6 (-2·3–11·1)	4376·4 (4043·3–4749·7)	5610·7 (5214·6–5950·9)	28·3 (24·6–32·7)
Netherlands	1160 (1060–1270)	1740 (1610–1890)	50·6 (40·8–58·5)	4169·6 (3829·6–4531·4)	5592·5 (5196·0–5994·8)	34·2 (29·7–38·5)
Norway	373 (348–404)	688 (619–750)	84·5 (67·3–103·4)	4689·7 (4343·5–5057·2)	5847·5 (5489·0–6235·4)	24·7 (22·2–28·6)
Portugal	1390 (1280–1510)	1960 (1650–2290)	41·4 (21·9–67·0)	7417·3 (6844·0–8033·9)	9831·4 (9238·1–10528·2)	32·6 (29·6–36·5)
San Marino	2·44 (2·24–2·68)	4·25 (3·73–4·86)	74·4 (54·1–95·1)	4749·2 (4369·4–5179·8)	6762·3 (6325·3–7187·4)	42·5 (37·6–48·5)
Spain	5320 (4990–5620)	8510 (7020–9680)	60·1 (39·7–80·5)	6859·2 (6411·7–7251·6)	9005·8 (8492·7–9481·9)	31·3 (28·3–34·8)
Sweden	831 (752–903)	1310 (1170–1460)	57·7 (43·7–71·6)	5100·6 (4665·7–5520·3)	6557·7 (6059·8–7017·5)	28·6 (25·4–32·8)
Switzerland	990 (902–1070)	1470 (1260–1620)	48·1 (32·1–63·0)	6906·5 (6302·6–7433·7)	8005·4 (7326·1–8498·6)	15·9 (14·2–18·0)
UK	7570 (7040–8080)	12800 (11500–14200)	68·5 (52·4–87·0)	7751·5 (7183·4–8262·7)	10464·3 (9820·5–11036·7)	35·1 (31·8–39·8)
**Latin America and Caribbean**	**43000 (40200–46200)**	**121000 (112000–130000)**	**180·4 (159·8–202·7)**	**6745·2 (6305·5–7228·1)**	**11317·5 (10767·3–11850·0)**	**67·9 (62·3–74·2)**
Andean Latin America	3090 (2890–3280)	9450 (8480–10300)	206·4 (177·0–233·9)	5010·8 (4688·3–5333·7)	8075·7 (7677·9–8465·9)	61·2 (56·0–67·3)
Bolivia	611 (563–662)	1810 (1620–2000)	196·9 (165·0–235·0)	6084·9 (5601·3–6613·7)	9419·6 (8795·8–9955·6)	54·9 (49·0–62·3)
Ecuador	1220 (1130–1310)	3620 (3170–4020)	196·3 (163·2–232·6)	7322·8 (6733·2–7833·3)	11935·1 (11334·4–12575·1)	63·1 (57·3–70·1)

Peru	1250 (1150–1360)	4010 (3520–4490)	221·5 (184·4–258·9)	3592·0 (3301·1–3901·9)	5773·3 (5424·0–6092·0)	60·8 (55·0–67·9)
Caribbean	4740 (4450–5070)	9580 (8560–10800)	102·2 (78·8–126·8)	8883·6 (8321·1–9487·1)	13104·0 (12474·2–13818·2)	47·6 (42·8–52·0)
Antigua and Barbuda	11·4 (10·3–12·3)	25·4 (20·1–30·6)	123·9 (82·5–181·6)	10382·6 (9459·6–11241·9)	16377·1 (15394·7–17415·7)	57·9 (51·9–64·0)
The Bahamas	42·1 (38·2–45·2)	96·1 (75·3–122)	128·6 (79·6–183·3)	9560·2 (8697·4–10280·5)	15013·3 (14123·5–15819·8)	57·1 (51·6–63·0)
Barbados	45·7 (42·6–48·7)	76·1 (65·5–86·3)	66·5 (43·7–92·1)	9743·9 (9022·1–10404·4)	14970·4 (14200·7–15662·0)	53·7 (48·8–58·6)
Belize	29·6 (27·2–32·6)	83·3 (63·6–102)	182·6 (108·4–250·7)	8481·5 (7760·1–9378·2)	11578·9 (10907·0–12481·2)	36·6 (32·2–41·0)
Bermuda	6·97 (6·44–7·64)	12·0 (10·9–13·0)	72·3 (59·4–84·7)	6109·9 (5687·7–6683·9)	9824·0 (9209·0–10496·5)	60·9 (54·6–68·1)
Cuba	1080 (996–1160)	1960 (1710–2260)	80·9 (60·9–107·9)	6174·4 (5704·5–6627·8)	10680·2 (10085·7–11231·8)	73·1 (66·0–80·9)
Dominica	9·62 (8·73–10·4)	18·4 (16·6–20·6)	91·3 (72·0–112·0)	10976·4 (9964·2–11891·4)	17311·7 (16240·3–18294·6)	57·9 (52·0–64·1)
Dominican Republic	929 (846–1010)	1790 (1590–1980)	92·9 (73·4–117·0)	8951·5 (8168·4–9739·0)	11994·6 (11093·7–12928·3)	34·1 (30·9–38·1)
Grenada	13·7 (12·5–15·0)	27·5 (21·1–35·2)	100·6 (54·8–157·4)	11385·1 (10473·4–12427·8)	17735·5 (16660·8–18748·8)	55·9 (50·2–61·2)
Guyana	114 (106–122)	198 (161–236)	74·6 (43·9–113·3)	15515·3 (14486·0–16602·0)	20024·9 (19031·7–21140·2)	29·1 (26·5–31·8)
Haiti	998 (907–1090)	2790 (2150–3550)	179·6 (112·2–255·8)	10966·8 (9908·6–12102·6)	15880·4 (14745·7–16971·7)	44·9 (40·2–50·4)
Jamaica	244 (223–264)	598 (496–718)	145·7 (104·0–191·7)	7891·5 (7239·7–8538·9)	12680·3 (12004·4–13375·7)	60·8 (54·9–67·4)
Puerto Rico	650 (599–711)	839 (705–979)	29·1 (11·4–50·4)	10991·1 (10090·4–12111·1)	14126·4 (13240·5–15320·2)	28·6 (25·6–31·5)
Saint Kitts and Nevis	7·68 (6·91–8·40)	16·0 (13·1–18·7)	108·8 (79·1–146·3)	10054·4 (9148·3–10952·0)	15820·4 (14866·2–16977·1)	57·5 (52·1–64·7)
Saint Lucia	27·2 (25·2–28·8)	59·7 (47·6–69·6)	120·0 (77·1–156·5)	11903·5 (11093·6–12610·6)	18063·2 (17198·3–18811·4)	51·8 (47·7–56·1)
Saint Vincent and the Grenadines	17·1 (15·8–18·3)	29·3 (25·3–35·0)	70·6 (48·4–98·3)	12148·7 (11253·9–12951·7)	18112·6 (17207·2–19070·6)	49·2 (44·6–53·7)
Suriname	77·0 (70·7–83·8)	162 (122–210)	109·8 (60·9–179·6)	11794·7 (10854·0–12780·7)	17001·8 (16035·1–18000·0)	44·2 (39·8–48·5)
Trinidad and Tobago	254 (234–272)	456 (378–556)	79·9 (47·7–123·8)	13331·5 (12356·9–14290·5)	19178·7 (18122·7–20195·3)	43·9 (40·1–48·3)
Virgin Islands	22·1 (20·3–24·1)	25·3 (20·4–31·2)	14·4 (-4·2–37·9)	13195·8 (12185·8–14235·0)	16791·7 (15745·4–17881·4)	27·3 (24·7–30·2)
Central Latin America	20900 (19600–22400)	63300 (58500–70400)	202·8 (175·6–236·7)	8009·9 (7516·3–8579·2)	13912·6 (13261·6–14517·5)	73·8 (67·8–79·2)
Colombia	3380 (3140–3610)	11500 (10400–12700)	239·6 (206·7–273·1)	6108·6 (5673·3–6529·8)	11770·7 (11229·4–12257·6)	92·8 (84·7–101·8)
Costa Rica	414 (388–447)	1070 (939–1210)	157·7 (124·6–195·1)	7544·8 (7067·4–8138·9)	11286·5 (10727·4–11977·9)	49·7 (44·3–54·2)
El Salvador	412 (374–453)	1070 (890–1250)	160·7 (111·8–214·9)	6678·9 (6063·1–7353·7)	11233·1 (10594·6–11961·5)	68·4 (61·0–76·2)

Guatemala	1130 (1030–1220)	4700 (3780–5500)	316·0 (236·3–388·6)	9212·1 (8429·5–9898·0)	15736·0 (14899·1–16530·4)	70·9 (63·8–77·4)
Honduras	636 (587–692)	2190 (1890–2420)	244·4 (189·2–297·7)	8416·2 (7701·7–9210·2)	13082·1 (12329·7–13848·0)	55·6 (50·2–62·1)
Mexico	11900 (11000–12900)	34900 (30500–39300)	193·0 (158·4–229·0)	9065·2 (8395·4–9841·6)	15404·2 (14642·2–16283·8)	70·0 (64·1–76·3)
Nicaragua	451 (414–485)	1420 (1170–1680)	215·7 (159·8–277·0)	8295·7 (7657·4–8906·5)	13525·1 (12782·5–14293·1)	63·2 (57·9–70·2)
Panama	319 (291–348)	1120 (967–1270)	252·4 (197·2–301·7)	7171·9 (6542·4–7834·5)	12861·7 (12171·0–13573·2)	79·5 (71·2–87·6)
Venezuela	2130 (1950–2320)	5240 (3590–6830)	145·8 (72·7–224·3)	6771·7 (6216·9–7356·3)	11192·3 (10511·0–11881·2)	65·4 (58·4–72·6)
Tropical Latin America	14300 (13100–15600)	38300 (34700–42300)	168·2 (140·6–198·9)	5451·0 (4997·9–5950·1)	8927·9 (8390·0–9535·4)	63·9 (57·3–71·7)
Brazil	13900 (12700–15200)	37200 (33600–41200)	168·4 (139·9–200·3)	5423·7 (4966·1–5929·5)	8922·8 (8370·4–9547·2)	64·6 (57·9–72·7)
Paraguay	414 (383–442)	1080 (924–1260)	160·3 (118·6–202·3)	6607·9 (6133·3–7053·0)	9394·9 (8867·3–9932·4)	42·2 (38·4–47·2)
**North Africa and Middle East**	**49900 (47000–53000)**	**181000 (166000–195000)**	**262·7 (233·4–294·6)**	**9288·7 (8742·9–9864·2)**	**16841·7 (16069·0–17554·6)**	**81·4 (75·6–88·6)**
North Africa and Middle East	49900 (47000–53000)	181000 (166000–195000)	262·7 (233·4–294·6)	9288·7 (8742·9–9864·2)	16841·7 (16069·0–17554·6)	81·4 (75·6–88·6)
Afghanistan	2920 (2690–3110)	9520 (6100–14200)	226·3 (109·5–382·2)	14609·5 (13535·9–15523·2)	19625·3 (18449·5–20548·3)	34·4 (31·3–38·1)
Algeria	4030 (3730–4300)	14500 (12700–16500)	259·5 (212·2–313·7)	10044·5 (9335·0–10739·5)	18484·0 (17702·4–19300·7)	84·1 (76·5–94·1)
Bahrain	241 (223–258)	893 (676–1050)	271·2 (186·1–347·5)	14986·1 (14117·7–15828·7)	23605·4 (22748·9–24585·0)	57·6 (53·2–62·5)
Egypt	6500 (6000–7070)	22800 (20300–25100)	251·8 (210·2–295·2)	8390·9 (7720·9–9158·4)	15178·4 (14289·8–16045·7)	81·1 (73·2–91·8)
Iran	5960 (5440–6490)	27000 (22300–31900)	353·6 (264·3–455·0)	6923·7 (6313·6–7525·6)	15847·3 (15039·5–16675·8)	129·2 (117·3–144·6)
Iraq	4790 (4450–5090)	11400 (9220–14100)	138·9 (92·2–194·4)	15298·1 (14317·4–16224·4)	18384·2 (17436·9–19372·2)	20·2 (18·4–22·3)
Jordan	1260 (1170–1350)	3870 (3030–4810)	208·2 (144·7–286·8)	13464·4 (12577·3–14491·0)	20020·8 (19172·8–21123·5)	48·8 (44·4–53·6)
Kuwait	665 (604–725)	2230 (1640–2680)	235·3 (159·9–322·3)	15200·7 (14080·9–16270·5)	23496·6 (22310·3–24842·2)	54·7 (49·4–61·7)
Lebanon	612 (569–649)	1550 (1240–1960)	153·6 (101·4–229·2)	11124·1 (10344·9–11825·3)	17596·2 (16745·3–18487·1)	58·3 (52·1–65·1)
Libya	699 (647–762)	2930 (2230–3680)	319·9 (223·6–437·7)	10632·9 (9856·1–11633·6)	23471·6 (22285·7–24673·2)	121·0 (110·0–132·5)
Morocco	5000 (4640–5340)	10700 (9510–11800)	113·4 (92·7–136·1)	13761·7 (12748·7–14709·2)	20050·2 (18901·2–21031·5)	45·8 (41·6–51·3)
Oman	281 (260–313)	2480 (2020–2840)	785·5 (587·7–933·6)	8934·8 (8281·8–9770·5)	21076·4 (20156·8–22185·5)	136·1 (121·2–150·4)
Palestine	300 (279–322)	1460 (1080–1820)	387·2 (250·8–510·0)	9776·8 (9116·0–10494·5)	17650·3 (16897·2–18425·7)	80·7 (73·9–88·9)
Qatar	308 (277–340)	1860 (1400–2280)	505·7 (368·9–651·8)	15139·8 (13988·7–16209·3)	23716·1 (22516·8–24896·1)	56·7 (51·7–62·4)

Saudi Arabia	3510 (3250–3750)	16500 (12400–20100)	370·5 (262·1–479·4)	11315·6 (10569·2–12019·2)	22581·9 (21614·5–23577·6)	99·7 (91·3–109·4)
Sudan	1960 (1810–2090)	11300 (9160–14200)	479·8 (365·3–661·4)	7873·0 (7233·0–8443·7)	16793·2 (15913·9–17598·0)	113·5 (103·5–124·6)
Syria	1220 (1130–1310)	4300 (3100–6030)	251·6 (155·1–386·1)	8718·2 (8086·9–9298·5)	20309·4 (19288·9–21297·9)	133·2 (122·3–146·2)
Tunisia	1360 (1230–1490)	4080 (3330–4750)	201·0 (148·4–253·7)	9842·8 (8954·1–10778·4)	18824·4 (17767·0–20027·0)	91·4 (82·1–102·6)
Türkiye	6390 (5870–6970)	20900 (18600–23300)	228·2 (186·4–275·6)	6626·9 (6104·8–7227·0)	12154·2 (11471·0–12815·2)	83·6 (73·3–91·6)
United Arab Emirates	822 (739–897)	4770 (3290–6360)	480·6 (316·1–650·0)	9504·3 (8726·4–10270·1)	21986·0 (20964·7–23078·4)	131·6 (120·0–150·6)
Yemen	1070 (980–1140)	5690 (4450–7180)	433·9 (301·6–583·1)	5878·4 (5384·5–6351·8)	11474·1 (10743·9–12204·2)	95·3 (85·2–107·0)
**South Asia**	**103000 (95300–111000)**	**321000 (284000–352000)**	**213·0 (176·9–250·0)**	**6067·5 (5642·9–6599·1)**	**9951·4 (9460·6–10524·0)**	**64·1 (57·8–72·5)**
South Asia	103000 (95300–111000)	321000 (284000–352000)	213·0 (176·9–250·0)	6067·5 (5642·9–6599·1)	9951·4 (9460·6–10524·0)	64·1 (57·8–72·5)
Bangladesh	11000 (10400–11800)	28600 (24000–33000)	159·5 (118·8–199·7)	7084·3 (6698·9–7586·4)	11411·6 (10928·0–11994·4)	61·2 (55·5–67·9)
Bhutan	35·6 (33·2–37·8)	124 (92·2–156)	249·9 (163·4–345·7)	5189·4 (4822·5–5541·9)	8987·6 (8449·0–9476·6)	73·3 (66·9–82·2)
India	78800 (72700–85900)	242000 (212000–270000)	207·7 (166·7–249·6)	5819·0 (5381·5–6350·4)	9388·5 (8884·7–9930·2)	61·5 (55·4–69·5)
Nepal	1950 (1820–2070)	5180 (4360–6060)	165·3 (122·8–212·4)	7280·9 (6765·6–7746·3)	11264·8 (10684·5–11823·4)	54·8 (49·5–62·1)
Pakistan	10800 (9950–11800)	44900 (38700–50800)	315·0 (248·5–375·2)	7144·2 (6622·0–7791·0)	12652·1 (12009·8–13347·5)	77·2 (69·4–86·7)
**Southeast Asia, east Asia, and Oceania**	**159000 (150000–170000)**	**354000 (332000–378000)**	**122·0 (109·0–139·7)**	**5773·6 (5416·9–6159·3)**	**9623·2 (9137·6–10121·8)**	**66·8 (60·6–74·8)**
East Asia	122000 (114000–131000)	264000 (245000–288000)	116·8 (101·0–138·5)	6139·5 (5688·0–6597·7)	11002·3 (10371·4–11606·3)	79·3 (71·5–88·0)
China	118000 (110000–127000)	257000 (238000–279000)	117·8 (101·1–140·1)	6157·7 (5697·9–6625·2)	11100·4 (10424·7–11733·0)	80·4 (72·7–89·5)
North Korea	1800 (1630–1960)	2700 (2250–3190)	49·5 (24·5–75·0)	5430·6 (4925·5–5915·8)	6466·1 (5923·6–6969·4)	19·1 (17·1–21·7)
Taiwan (province of China)	2270 (2130–2430)	4960 (4410–5550)	118·3 (98·9–144·8)	5853·6 (5504·9–6247·7)	10555·8 (10108·8–10985·3)	80·4 (73·8–87·0)
Oceania	1220 (1140–1310)	3260 (2980–3620)	167·3 (141·6–203·7)	12255·9 (11524·9–13025·5)	15986·1 (15309·2–16772·6)	30·5 (27·9–33·9)
American Samoa	11·3 (10·5–12·0)	22·8 (20·9–24·6)	101·4 (82·1–115·3)	21350·9 (19873·5–22715·4)	27225·5 (25716·2–28676·0)	27·5 (25·5–29·8)
Cook Islands	4·54 (4·18–4·84)	6·72 (6·24–7·15)	48·1 (41·5–57·1)	19310·3 (17832·0–20652·1)	25382·0 (23916·6–26808·4)	31·5 (29·0–34·5)
Federated States of Micronesia	12·4 (11·7–13·3)	25·5 (19·3–36·5)	105·8 (56·0–195·9)	14162·3 (13369·6–15007·3)	21749·5 (20891·0–22689·8)	53·6 (50·3–58·2)
Fiji	140 (130–150)	283 (222–364)	102·2 (53·8–170·1)	16262·0 (15192·5–17352·6)	23427·8 (22364·3–24582·9)	44·1 (41·2–48·1)
Guam	16·4 (15·2–17·9)	29·8 (22·8–37·4)	81·3 (43·1–126·5)	8639·3 (7990·9–9401·1)	11370·1 (10653·9–12192·4)	31·7 (28·5–35·2)
Kiribati	13·2 (12·2–14·1)	34·7 (25·9–43·3)	163·5 (93·0–241·3)	14377·8 (13466·0–15325·9)	22591·9 (21528·6–23536·9)	57·2 (53·1–62·2)

Marshall Islands	10·8 (9·98–11·7)	23·4 (20·8–25·8)	117·1 (93·4–140·4)	22173·9 (20687·2–23875·3)	28937·9 (27240·7–30712·5)	30·5 (28·0–33·2)
Nauru	1·10 (1·03–1·18)	3·05 (2·77–3·36)	177·8 (152·6–214·7)	15952·0 (14972·0–16853·4)	22218·8 (21142·3–23287·0)	39·3 (36·1–42·8)
Niue	0·374 (0·346–0·399)	0·624 (0·569–0·668)	66·9 (56·2–80·0)	18316·2 (16927·9–19688·6)	27476·8 (25902·1–28868·0)	50·1 (46·2–54·6)
Northern Mariana Islands	5·84 (5·20–6·33)	10·4 (9·68–11·3)	78·1 (62·0–95·4)	9830·9 (8899·3–10641·6)	16795·2 (15835·0–17636·4)	71·0 (63·6–79·3)
Palau	4·14 (3·89–4·37)	5·43 (4·92–5·97)	31·4 (20·7–42·8)	16965·0 (15938·4–17864·3)	23019·2 (21994·4–23985·6)	35·7 (33·1–39·4)
Papua New Guinea	839 (779–906)	2400 (2200–2710)	186·0 (158·0–235·3)	11656·2 (10837·9–12448·3)	15221·1 (14364·1–16007·1)	30·6 (27·8–34·0)
Samoa	27·4 (25·4–29·8)	51·8 (44·9–60·3)	89·1 (70·7–123·9)	15753·5 (14655·1–17091·8)	21614·3 (20406·1–22990·9)	37·3 (34·0–41·3)
Solomon Islands	39·5 (36·3–42·7)	107 (86·1–128)	169·7 (125·9–226·2)	8811·0 (8215·4–9406·7)	13821·0 (13171·7–14565·4)	56·9 (52·2–63·1)
Tokelau	0·258 (0·238–0·275)	0·511 (0·475–0·552)	97·8 (83·9–111·0)	17608·4 (16240·6–18760·7)	24883·4 (23368·9–26080·6)	41·4 (37·9–45·5)
Tonga	11·8 (11·1–12·5)	27·6 (21·3–35·8)	133·4 (87·8–212·5)	13908·2 (13021·0–14762·3)	21232·8 (20347·2–22102·0)	52·7 (47·8–58·0)
Tuvalu	1·27 (1·18–1·33)	3·68 (3·39–4·17)	190·7 (164·7–224·3)	11242·8 (10480·3–11812·3)	21112·9 (20108·7–22132·9)	87·9 (81·8–95·4)
Vanuatu	24·7 (23·0–26·3)	75·7 (58·9–93·3)	206·9 (133·1–287·1)	11093·3 (10361·1–11842·0)	16622·9 (15787·8–17340·7)	49·9 (46·1–55·0)
Southeast Asia	36100 (33700–38600)	86000 (80200–92200)	138·2 (122·7–157·1)	5378·7 (5000·7–5734·8)	7282·5 (6882·4–7637·1)	35·4 (31·9–39·3)
Cambodia	687 (632–726)	1820 (1410–2330)	164·7 (107·7–235·5)	5070·2 (4668·0–5409·6)	6589·7 (6170·2–6974·3)	30·0 (26·5–34·2)
Indonesia	11500 (10600–12600)	27600 (25200–30500)	140·5 (117·6–169·9)	4584·4 (4171·9–4972·2)	6279·9 (5814·6–6692·2)	37·1 (32·6–42·3)
Laos	317 (294–338)	1050 (880–1270)	232·3 (178·8–294·3)	6137·7 (5676·9–6584·8)	8810·7 (8233·3–9291·9)	43·6 (39·7–48·7)
Malaysia	2220 (2090–2400)	6150 (5070–7270)	177·2 (133·5–225·2)	7416·0 (6945·0–7981·5)	10415·9 (9884·1–10986·8)	40·5 (36·5–44·8)
Maldives	22·4 (20·6–24·5)	121 (102–138)	444·3 (353·2–552·7)	5447·7 (4935·5–6004·7)	9828·8 (9252·3–10493·5)	80·6 (71·2–91·1)
Mauritius	205 (189–220)	359 (306–419)	75·4 (52·4–105·9)	11230·0 (10378·0–12024·1)	15027·3 (14134·1–15810·1)	33·9 (30·9–36·7)
Myanmar	4080 (3850–4350)	7900 (7140–8680)	93·5 (73·5–113·4)	7994·7 (7473·1–8523·7)	9522·7 (8992·8–10100·1)	19·1 (17·4–21·1)
Philippines	3940 (3660–4290)	12000 (10500–13100)	204·1 (162·3–238·2)	4423·8 (4070·2–4785·7)	6302·4 (5876·6–6697·5)	42·6 (37·7–47·9)
Seychelles	13·2 (12·1–14·3)	29·2 (22·9–35·1)	120·2 (77·2–172·7)	11029·9 (10182·1–11808·3)	17042·7 (16165·7–18006·5)	54·6 (49·2–59·9)
Sri Lanka	2770 (2590–2990)	4870 (4220–5500)	75·6 (55·9–99·7)	10297·2 (9694·7–11089·3)	12668·0 (11987·2–13488·0)	23·1 (21·0–24·9)
Thailand	5760 (5180–6320)	13200 (11600–14900)	128·9 (99·4–159·1)	5447·8 (4917·5–5948·0)	9012·8 (8446·1–9516·0)	65·6 (59·1–73·0)
Timor-Leste	53·7 (49·0–57·6)	129 (94·4–173)	140·5 (73·3–218·0)	5902·3 (5336·6–6332·5)	7180·3 (6533·0–7621·0)	21·7 (19·3–24·6)
Viet Nam	4530 (4290–4760)	10800 (9340–12400)	138·1 (110·5–179·7)	4518·3 (4259·8–4781·9)	5853·4 (5578·9–6133·4)	29·6 (27·1–32·7)

**Sub-Saharan Africa**	**26900 (25300–28700)**	**110000 (97600–122000)**	**308·8 (259·2–371·1)**	**4247·5 (3947·0–4530·7)**	**6648·5 (6232·5–6991·2)**	**56·6 (50·8–64·2)**
Central sub-Saharan Africa	4120 (3790–4420)	19600 (15700–24900)	376·4 (271·9–500·7)	5263·8 (4847·9–5638·8)	9411·6 (8929·0–9883·1)	78·9 (71·6–88·6)
Angola	1060 (980–1150)	4260 (3270–5280)	302·2 (217·2–402·1)	6198·2 (5686·2–6707·5)	9621·4 (8984·0–10183·3)	55·3 (49·7–62·2)
Central African Republic	230 (212–247)	734 (547–1030)	220·1 (142·5–364·6)	7086·4 (6557·0–7569·2)	12930·4 (12257·8–13694·9)	82·6 (74·7–93·5)
Congo (Brazzaville)	209 (192–226)	909 (729–1110)	335·3 (263·1–458·2)	5709·0 (5223·8–6130·5)	11163·0 (10634·1–11726·2)	95·7 (86·9–107·5)
DR Congo	2480 (2260–2650)	13100 (8960–17900)	428·4 (253·6–607·6)	4760·7 (4339·0–5125·6)	8974·3 (8503·7–9407·8)	88·7 (80·3–100·1)
Equatorial Guinea	49·8 (46·0–53·8)	279 (187–364)	460·5 (266·6–627·0)	6377·6 (5764·2–6841·7)	12160·1 (11477·9–12777·8)	90·8 (82·0–102·5)
Gabon	91·0 (84·8–97·7)	336 (262–407)	269·2 (187·9–353·4)	6789·1 (6359·8–7259·5)	11767·4 (11116·0–12347·9)	73·4 (66·2–82·8)
Eastern sub-Saharan Africa	6790 (6360–7210)	33100 (29700–36900)	387·4 (332·4–470·6)	2921·9 (2716·9–3097·0)	4767·2 (4437·2–5041·1)	63·2 (54·8–72·4)
Burundi	192 (179–206)	723 (449–958)	276·7 (135·9–420·6)	2921·5 (2697·2–3118·5)	3613·5 (3380·3–3841·0)	23·7 (21·2–27·0)
Comoros	25·5 (23·6–27·3)	83·5 (65·1–105)	228·0 (161·8–309·1)	4399·8 (4056·4–4702·9)	7653·9 (7234·8–8075·9)	74·1 (66·9–84·3)
Djibouti	25·8 (23·9–27·7)	89·5 (64·8–120)	247·3 (156·9–365·3)	3085·3 (2819·3–3283·1)	4250·7 (3966·8–4494·0)	37·8 (34·0–42·5)
Eritrea	165 (154–177)	561 (404–782)	239·5 (147·7–377·1)	4064·6 (3772·8–4362·4)	5639·3 (5339·1–5952·2)	38·8 (34·3–43·4)
Ethiopia	2010 (1870–2150)	6660 (5110–8350)	231·1 (157·6–321·3)	3354·5 (3130·5–3611·9)	4037·7 (3780·5–4291·1)	20·4 (17·4–23·2)
Kenya	622 (580–664)	4490 (3900–5290)	621·7 (517·1–759·5)	2046·5 (1908·0–2200·3)	4628·8 (4350·7–4912·3)	126·4 (110·5–148·8)
Madagascar	434 (404–468)	1920 (1470–2260)	342·9 (231·8–433·8)	2751·5 (2530·9–2959·3)	4192·7 (3914·7–4430·0)	52·5 (46·5–59·8)
Malawi	215 (202–229)	1490 (938–2110)	593·1 (327·3–905·9)	2104·1 (1956·5–2253·9)	4063·9 (3867·0–4291·4)	93·3 (83·7–106·1)
Mozambique	506 (462–552)	2950 (2010–3940)	484·9 (298·1–713·3)	3274·8 (2956·8–3557·7)	6329·6 (5979·4–6691·0)	93·5 (82·9–104·8)
Rwanda	187 (172–199)	853 (579–1160)	356·8 (224·1–539·2)	2313·1 (2125·1–2474·9)	3699·1 (3451·2–3899·5)	60·0 (52·7–67·2)
Somalia	328 (303–352)	1360 (833–2250)	316·6 (158·4–576·4)	3155·2 (2902·3–3382·0)	5029·9 (4728·2–5323·5)	59·5 (52·8–68·0)
South Sudan	143 (132–153)	571 (329–768)	300·1 (128·1–441·8)	2856·3 (2622·5–3062·1)	3442·6 (3218·0–3662·5)	20·6 (18·2–24·1)
Tanzania	862 (812–916)	5420 (4370–6580)	528·8 (396·4–680·8)	2625·1 (2427·3–2803·0)	5846·7 (5540·2–6198·2)	122·9 (109·5–137·9)
Uganda	580 (538–617)	3830 (3090–4810)	560·7 (434·0–746·6)	2823·4 (2587·4–3019·2)	4905·0 (4616·4–5169·9)	73·9 (65·4–84·3)
Zambia	485 (451–524)	2040 (1760–2390)	321·1 (260·6–407·5)	4619·2 (4277·1–4943·7)	7741·7 (7269·5–8142·6)	67·7 (60·1–76·3)
Southern sub-Saharan Africa	3640 (3410–3910)	11900 (10400–13500)	226·7 (180·0–275·6)	5602·3 (5225·5–6018·8)	9661·3 (9178·5–10209·2)	72·6 (65·9–80·9)
Botswana	85·2 (79·3–90·3)	410 (322–492)	381·9 (269·8–494·9)	4869·4 (4521·4–5107·2)	9774·9 (9344·0–10251·5)	100·9 (91·4–111·7)
Eswatini	44·9 (41·6–48·0)	180 (135–228)	301·7 (198·9–404·8)	6412·2 (5938·6–6822·8)	11520·8 (10971·6–12090·0)	79·8 (72·2–87·8)
Lesotho	72·1 (67·1–77·5)	228 (190–271)	216·6 (162·6–285·2)	4883·5 (4547·2–5255·8)	8948·0 (8503·8–9428·5)	83·3 (75·3–92·6)

Namibia	69·7<br>(64·1–75·5)	311<br>(239–401)	346·9<br>(235·0–491·1)	4124·2<br>(3726·6–4500·1)	7755·1<br>(7248·8–8181·0)	88·2<br>(78·7–99·7)
South Africa	2980<br>(2790–3210)	8650<br>(7410–10100)	190·0<br>(148·3–233·2)	5878·3<br>(5464·8–6315·5)	9706·7<br>(9220·3–10254·0)	65·2<br>(58·8–72·1)
Zimbabwe	387<br>(359–414)	2120<br>(1660–2680)	447·0<br>(325·5–595·8)	4347·0<br>(3997·4–4644·8)	9050·3<br>(8566·8–9521·4)	108·4<br>(97·0–122·1)
Western sub-Saharan Africa	12400<br>(11600–13200)	45500<br>(40300–49700)	267·3<br>(220·6–317·4)	4678·0<br>(4348·9–4982·2)	7035·5<br>(6623·8–7355·6)	50·5<br>(45·2–56·6)
Benin	478<br>(448–509)	1600<br>(1410–1800)	236·2<br>(186·4–274·1)	6577·3<br>(6146·8–7059·6)	9084·9<br>(8559·5–9597·7)	38·2<br>(34·3–43·2)
Burkina Faso	565<br>(533–598)	1760<br>(1520–2000)	211·3<br>(170·8–265·3)	4407·0<br>(4106·3–4711·6)	5188·9<br>(4885·2–5502·5)	17·8<br>(15·5–19·8)
Cabo Verde	35·7<br>(32·7–38·1)	113<br>(84·8–142)	218·7<br>(137·7–309·8)	6994·7<br>(6489·1–7487·7)	11910·7<br>(11371·3–12502·9)	70·4<br>(63·3–78·9)
Cameroon	888<br>(829–946)	3690<br>(2700–4870)	316·7<br>(201·8–446·4)	5034·1<br>(4724·3–5359·5)	8713·6<br>(8336·0–9143·3)	73·2<br>(66·6–82·1)
Chad	399<br>(369–428)	1290<br>(956–1630)	224·7<br>(140·0–304·8)	4890·2<br>(4488·1–5242·8)	5781·2<br>(5330·8–6137·2)	18·3<br>(16·2–20·8)
Côte d'Ivoire	876<br>(814–937)	3380<br>(2640–4040)	285·9<br>(199·2–369·8)	5529·9<br>(5115·1–5915·8)	8698·2<br>(8209·6–9171·4)	57·4<br>(52·3–63·8)
The Gambia	70·0<br>(64·4–74·7)	242<br>(183–329)	246·5<br>(159·4–366·2)	5234·4<br>(4814·3–5635·1)	7915·1<br>(7404·6–8434·7)	51·3<br>(46·4–57·0)
Ghana	1190<br>(1090–1270)	4750<br>(3680–5970)	300·6<br>(202·0–403·8)	5344·7<br>(4889·2–5715·4)	9541·1<br>(8985·2–9965·2)	78·7<br>(70·9–88·6)
Guinea	323<br>(300–344)	1170<br>(847–1550)	262·9<br>(159·0–391·8)	4413·4<br>(4077·7–4732·3)	6825·8<br>(6510·2–7166·1)	54·7<br>(50·0–61·8)
Guinea-Bissau	65·1<br>(60·6–69·5)	205<br>(156–263)	215·8<br>(135·5–312·3)	5962·0<br>(5551·9–6312·1)	8624·5<br>(8171·1–9052·4)	44·7<br>(40·9–50·6)
Liberia	177<br>(162–188)	732<br>(509–949)	314·3<br>(193·3–432·3)	5797·3<br>(5333·9–6181·3)	9547·2<br>(9083·4–10003·4)	64·8<br>(58·0–73·6)
Mali	1060<br>(973–1140)	2440<br>(1930–2940)	130·2<br>(82·0–180·4)	8781·8<br>(8029·8–9442·1)	10728·6<br>(9954·5–11401·3)	22·2<br>(19·8–25·3)
Mauritania	91·0<br>(85·8–96·2)	380<br>(327–428)	318·2<br>(268·6–375·3)	3440·9<br>(3228·6–3656·7)	5892·6<br>(5620·4–6186·2)	71·3<br>(64·5–80·0)
Niger	550<br>(508–588)	1310<br>(1110–1500)	138·5<br>(105·3–173·1)	4856·0<br>(4445·8–5255·5)	5256·7<br>(4850·6–5651·1)	8·3<br>(7·3–9·4)
Nigeria	4490<br>(4180–4790)	18900<br>(16300–21700)	321·4<br>(254·5–390·2)	3713·2<br>(3446·3–4005·0)	6017·9<br>(5657·2–6346·4)	62·2<br>(55·4–69·9)
São Tomé and Príncipe	8·60<br>(7·96–9·18)	29·4<br>(23·6–37·6)	242·5<br>(175·1–336·0)	5862·5<br>(5435·4–6259·9)	9481·5<br>(8876·6–9939·8)	61·8<br>(55·2–69·6)
Senegal	683<br>(643–724)	2020<br>(1630–2340)	196·2<br>(136·4–247·0)	7160·4<br>(6728·4–7607·4)	9377·5<br>(8919·2–9854·9)	31·0<br>(28·2–34·3)
Sierra Leone	262<br>(239–282)	718<br>(536–899)	173·8<br>(107·9–241·6)	5101·6<br>(4660·1–5466·3)	6901·2<br>(6409·5–7262·4)	35·3<br>(31·5–39·9)
Togo	185<br>(172–195)	739<br>(560–933)	300·7<br>(211·7–416·5)	3601·6<br>(3350·9–3815·0)	6179·0<br>(5846·7–6459·1)	71·6<br>(65·0–79·6)

Table S24. Death counts and age-standardised rates per 100,000 population and the corresponding percentage change between 1990 and 2021 for diabetes globally, in 21 Global Burden of Disease regions and all countries

95% confidence intervals in parentheses

Location	Deaths 2021, number (thousands)	Deaths percent change 1990 - 2021, number (%)	Deaths 2021, rate (per 100,000)	Deaths percent change 1990 - 2021, rate (%)
**Global**	**1700**<br>**(1570–1790)**	**152·7**<br>**(135·0–167·9)**	**19·9**<br>**(18·3–21·1)**	**8·6**<br>**(1·2–14·7)**
**Central Europe, eastern Europe, and central Asia**	**92·5**<br>**(87·5–97·3)**	**151·8**<br>**(140·2–163·8)**	**14·1**<br>**(13·3–14·8)**	**78·3**<br>**(70·0–86·5)**

Central Asia	14·4 (12·7–16·2)	202·1 (163·8–240·1)	19·3 (17·2–21·5)	91·4 (68·5–115·2)
Armenia	0·656 (0·584–0·729)	30·4 (13·8–47·5)	15·6 (13·9–17·2)	-19·1 (-29·4–-8·5)
Azerbaijan	1·84 (1·33–2·43)	201·5 (111·2–320·4)	18·4 (13·4–24·2)	52·1 (7·7–114·3)
Georgia	1·08 (0·944–1·22)	50·2 (26·6–76·1)	18·0 (15·8–20·5)	57·0 (32·2–83·9)
Kazakhstan	1·58 (1·33–1·84)	70·2 (41·9–106·9)	9·3 (7·8–10·9)	26·6 (6·1–54·1)
Kyrgyzstan	0·387 (0·343–0·443)	128·4 (96·8–162·9)	8·2 (7·3–9·3)	45·1 (22·7–66·8)
Mongolia	0·221 (0·178–0·271)	212·7 (130·1–351·7)	9·0 (7·3–11·2)	38·7 (0·4–103·6)
Tajikistan	0·940 (0·704–1·17)	169·8 (95·1–261·6)	18·7 (14·3–22·8)	56·1 (15·8–102·5)
Turkmenistan	0·930 (0·728–1·17)	333·6 (231·4–450·4)	21·4 (17·0–26·9)	96·7 (51·6–148·2)
Uzbekistan	6·75 (5·62–7·90)	471·2 (380·6–581·8)	25·4 (21·3–29·6)	153·1 (112·6–201·4)
Central Europe	33·1 (30·8–35·4)	66·4 (56·3–77·0)	14·3 (13·4–15·3)	3·7 (-2·5–10·0)
Albania	0·225 (0·187–0·285)	120·8 (75·8–191·1)	5·0 (4·1–6·3)	-8·4 (-27·0–20·8)
Bosnia and Herzegovina	2·08 (1·68–2·51)	243·6 (166·5–316·1)	33·9 (27·6–40·8)	102·3 (57·6–148·6)
Bulgaria	2·46 (2·17–2·73)	15·7 (0·3–34·0)	16·6 (14·7–18·5)	-10·6 (-22·2–2·9)
Croatia	1·57 (1·39–1·77)	100·5 (70·1–128·5)	15·9 (14·1–17·9)	22·6 (4·6–39·9)
Czechia	4·49 (3·98–5·18)	113·6 (87·0–142·3)	19·2 (17·0–22·0)	25·7 (10·3–41·7)
Hungary	2·83 (2·53–3·12)	44·8 (28·5–59·4)	13·6 (12·2–15·0)	1·1 (-10·1–11·1)
Montenegro	0·181 (0·150–0·211)	121·3 (75·8–172·5)	18·4 (15·4–21·5)	34·6 (7·6–64·8)
North Macedonia	1·03 (0·828–1·26)	157·6 (97·8–231·0)	34·9 (28·4–41·8)	50·5 (17·5–89·8)
Poland	10·3 (9·27–11·0)	64·0 (51·2–77·9)	13·5 (12·3–14·5)	-5·2 (-12·8–2·7)
Romania	2·79 (2·48–3·09)	30·2 (17·4–47·7)	7·1 (6·3–7·9)	-10·2 (-19·0–1·6)
Serbia	3·86 (3·31–4·67)	70·7 (35·9–109·1)	21·7 (18·6–26·1)	-0·1 (-21·6–22·6)
Slovakia	0·881 (0·726–1·07)	17·7 (-5·6–44·0)	9·1 (7·5–11·1)	-27·3 (-41·7–-10·8)
Slovenia	0·426 (0·374–0·484)	45·7 (27·8–70·2)	8·5 (7·4–9·7)	-29·6 (-37·7–-17·5)
Eastern Europe	45·0 (41·9–48·1)	272·8 (246·1–299·7)	12·4 (11·5–13·2)	183·1 (164·0–203·3)
Belarus	0·732 (0·605–0·838)	28·6 (9·5–52·8)	4·7 (3·8–5·3)	4·7 (-10·9–25·2)
Estonia	0·309 (0·274–0·347)	202·3 (165·8–248·0)	11·1 (9·9–12·5)	115·9 (91·8–147·3)
Latvia	0·570 (0·501–0·627)	159·7 (128·6–193·0)	14·0 (12·1–15·3)	120·6 (94·1–149·0)
Lithuania	0·570 (0·496–0·638)	211·6 (175·8–255·3)	9·8 (8·6–11·0)	137·1 (107·5–170·3)

Moldova	0·544 (0·488–0·622)	73·3 (54·3–96·9)	9·7 (8·7–11·1)	34·5 (20·5–51·8)
Russia	39·8 (36·9–42·6)	410·8 (371·3–444·6)	16·0 (14·8–17·2)	266·3 (240·1–290·2)
Ukraine	2·45 (1·87–3·22)	-15·0 (-36·6–11·6)	3·3 (2·5–4·3)	-19·9 (-40·1–4·7)
**High income**	**214 (187–226)**	**25·3 (17·6–29·4)**	**8·9 (7·9–9·3)**	**-37·3 (-39·5–-35·7)**
Australasia	4·97 (4·36–5·37)	86·7 (72·4–103·7)	8·7 (7·7–9·3)	-25·9 (-31·1–-20·0)
Australia	4·31 (3·76–4·65)	93·4 (78·4–111·6)	8·9 (7·8–9·5)	-24·9 (-30·3–-18·8)
New Zealand	0·662 (0·592–0·717)	52·4 (37·0–67·9)	7·5 (6·8–8·1)	-32·8 (-39·4–-26·2)
High-income Asia Pacific	21·5 (17·7–23·9)	16·1 (1·7–28·5)	4·0 (3·4–4·4)	-58·9 (-62·6–-54·9)
Brunei	0·150 (0·130–0·171)	97·9 (61·9–133·3)	62·7 (55·0–71·7)	-37·1 (-48·8–-26·1)
Japan	10·2 (8·43–11·3)	-15·5 (-24·9–-9·0)	2·3 (2·0–2·4)	-69·8 (-71·2–-68·3)
Singapore	0·168 (0·151–0·181)	-54·0 (-58·3–-49·9)	2·1 (1·8–2·2)	-88·7 (-89·7–-87·7)
South Korea	11·0 (8·98–12·5)	83·6 (54·7–118·5)	11·7 (9·5–13·3)	-46·7 (-54·6–-37·3)
High-income North America	81·5 (74·1–85·9)	50·4 (45·5–55·5)	12·4 (11·4–13·1)	-18·6 (-20·8–-16·0)
Canada	6·60 (5·85–7·16)	45·5 (36·4–58·6)	8·6 (7·7–9·3)	-39·2 (-42·7–-34·2)
Greenland	0·00641 (0·00532–0·00766)	45·8 (20·1–76·7)	9·7 (8·2–11·6)	-35·0 (-47·3–-22·1)
USA	74·9 (68·2–78·9)	50·8 (46·2–55·7)	12·9 (11·8–13·5)	-16·4 (-18·7–-13·9)
Southern Latin America	13·6 (12·6–14·4)	40·1 (31·4–47·9)	15·3 (14·2–16·2)	-29·8 (-33·8–-25·9)
Argentina	9·58 (8·95–10·2)	29·1 (20·0–36·8)	16·9 (15·8–17·9)	-29·1 (-33·7–-25·0)
Chile	3·01 (2·74–3·22)	89·8 (74·2–110·1)	11·7 (10·6–12·5)	-30·7 (-35·9–-23·5)
Uruguay	0·980 (0·877–1·05)	45·4 (31·0–58·5)	16·3 (14·7–17·4)	-4·6 (-12·6–3·7)
Western Europe	92·7 (78·2–99·5)	7·9 (-1·2–13·2)	8·2 (7·0–8·7)	-43·3 (-46·8–-41·0)
Andorra	0·0153 (0·0113–0·0193)	153·7 (70·5–244·0)	9·5 (7·0–12·1)	-28·2 (-51·6–-3·5)
Austria	2·06 (1·77–2·24)	11·8 (2·2–21·7)	9·6 (8·3–10·3)	-35·8 (-40·6–-30·4)
Belgium	1·49 (1·25–1·62)	-21·3 (-28·4–-14·8)	5·4 (4·6–5·8)	-55·5 (-58·1–-52·3)
Cyprus	0·551 (0·477–0·628)	18·2 (-3·1–38·9)	31·0 (27·2–35·0)	-58·6 (-65·3–-51·2)
Denmark	1·42 (1·25–1·54)	58·6 (42·2–71·5)	10·8 (9·6–11·7)	-0·7 (-10·3–7·0)
Finland	0·613 (0·530–0·665)	10·1 (1·2–19·7)	4·4 (3·9–4·8)	-43·8 (-47·4–-39·5)
France	13·2 (11·0–14·2)	58·3 (42·2–69·4)	7·2 (6·2–7·7)	-23·9 (-30·3–-19·3)
Germany	21·8 (18·1–23·8)	2·5 (-7·0–11·0)	9·6 (8·1–10·4)	-40·5 (-44·7–-36·2)

Greece	1·78 (1·56–1·96)	50·9 (37·3–68·9)	6·3 (5·7–6·9)	-20·4 (-26·4–-12·4)
Iceland	0·0280 (0·0240–0·0309)	59·0 (44·3–75·1)	4·3 (3·8–4·7)	-27·3 (-34·0–-19·9)
Ireland	0·438 (0·373–0·484)	-5·4 (-15·9–5·8)	5·3 (4·5–5·8)	-54·1 (-58·7–-49·1)
Israel	2·48 (2·08–2·68)	150·2 (127·3–169·3)	18·3 (15·6–19·8)	-14·0 (-21·0–-7·3)
Italy	19·5 (16·2–21·2)	5·7 (-3·3–12·8)	10·5 (9·0–11·3)	-48·1 (-51·4–-45·2)
Luxembourg	0·0738 (0·0625–0·0822)	16·8 (2·7–29·7)	6·2 (5·3–6·9)	-47·6 (-53·9–-41·9)
Malta	0·153 (0·131–0·170)	45·3 (28·9–63·0)	14·2 (12·3–15·8)	-45·6 (-51·2–-39·3)
Monaco	0·00434 (0·00347–0·00515)	47·4 (14·9–95·8)	3·8 (3·1–4·5)	2·2 (-21·2–35·6)
Netherlands	3·23 (2·78–3·54)	-16·5 (-23·9–-9·6)	8·3 (7·2–9·0)	-56·8 (-60·7–-53·4)
Norway	0·641 (0·560–0·694)	19·6 (12·2–26·5)	5·8 (5·1–6·2)	-23·7 (-27·5–-19·5)
Portugal	3·96 (3·35–4·31)	29·5 (12·2–42·1)	13·0 (11·2–14·0)	-41·8 (-48·2–-36·5)
San Marino	0·00390 (0·00251–0·00521)	84·6 (28·4–151·7)	4·8 (3·1–6·4)	-26·2 (-48·9–2·1)
Spain	9·38 (7·75–10·3)	-6·0 (-16·0–1·4)	7·2 (6·2–7·9)	-60·6 (-63·9–-57·6)
Sweden	2·00 (1·68–2·27)	29·7 (12·4–47·0)	7·8 (6·7–8·8)	-19·9 (-30·3–-10·1)
Switzerland	1·23 (1·04–1·35)	-24·1 (-31·2–-17·3)	5·6 (4·8–6·1)	-61·9 (-65·1–-58·8)
UK	6·64 (5·96–7·00)	-23·6 (-27·3–-21·0)	4·6 (4·2–4·9)	-50·7 (-52·6–-49·3)
**Latin America and Caribbean**	**225 (210–242)**	**178·6 (163·2–198·3)**	**36·6 (34·2–39·4)**	**-6·5 (-11·7–-0·2)**
Andean Latin America	14·2 (12·3–17·1)	254·8 (200·9–328·3)	24·4 (21·2–29·2)	17·7 (0·1–41·8)
Bolivia	3·88 (3·23–4·86)	225·4 (158·7–340·3)	44·9 (37·6–55·7)	12·6 (-9·1–49·4)
Ecuador	4·52 (3·64–5·50)	290·4 (211·0–376·2)	29·4 (23·9–35·7)	27·2 (1·9–54·6)
Peru	5·84 (4·64–7·52)	253·3 (165·4–364·0)	17·0 (13·6–22·0)	17·1 (-12·0–55·3)
Caribbean	20·5 (17·8–23·6)	77·2 (58·3–104·5)	37·8 (32·9–43·6)	-17·6 (-26·2–-5·0)
Antigua and Barbuda	0·0583 (0·0532–0·0616)	61·0 (47·4–74·4)	60·3 (54·9–63·9)	-12·3 (-19·5–-5·1)
The Bahamas	0·146 (0·119–0·184)	95·3 (59·3–146·8)	37·8 (31·1–47·4)	-26·1 (-39·4–-7·2)
Barbados	0·296 (0·241–0·370)	26·1 (-3·1–58·4)	57·2 (46·7–71·5)	-25·7 (-42·7–-6·7)
Belize	0·160 (0·143–0·178)	249·2 (211·2–292·9)	56·2 (50·2–62·4)	13·0 (0·6–27·3)
Bermuda	0·0259 (0·0221–0·0309)	17·8 (2·2–42·3)	17·7 (15·1–21·2)	-52·5 (-59·0–-42·5)
Cuba	2·09 (1·82–2·38)	-7·1 (-19·6–5·4)	10·3 (8·9–11·6)	-53·8 (-60·1–-47·8)
Dominica	0·0713 (0·0635–0·0819)	31·4 (15·7–56·0)	76·5 (67·9–87·9)	5·3 (-6·7–24·7)

Dominican Republic	2·88<br>(2·26–3·66)	247·5<br>(166·2–339·1)	30·6<br>(24·0–38·6)	29·0<br>(-1·0–62·6)
Grenada	0·0968<br>(0·0870–0·104)	47·9<br>(31·0–67·9)	92·9<br>(84·3–99·7)	9·3<br>(-2·7–23·3)
Guyana	0·530<br>(0·423–0·661)	82·5<br>(44·7–124·1)	87·7<br>(70·8–107·5)	8·3<br>(-12·8–30·0)
Haiti	5·54<br>(4·29–8·00)	106·0<br>(51·0–172·6)	82·8<br>(64·7–118·0)	-6·7<br>(-31·5–21·9)
Jamaica	2·17<br>(1·71–2·62)	75·4<br>(38·8–118·9)	68·2<br>(53·5–82·6)	0·1<br>(-21·2–25·5)
Puerto Rico	3·20<br>(2·68–3·70)	64·3<br>(37·1–92·6)	40·5<br>(34·3–46·9)	-26·7<br>(-39·2–-14·6)
Saint Kitts and Nevis	0·0323<br>(0·0260–0·0375)	10·3<br>(-11·5–29·7)	53·7<br>(44·8–61·5)	-32·5<br>(-43·6–-22·3)
Saint Lucia	0·122<br>(0·104–0·144)	52·6<br>(30·8–80·1)	56·0<br>(47·9–66·1)	-42·5<br>(-50·6–-32·2)
Saint Vincent and the Grenadines	0·0982<br>(0·0894–0·110)	40·2<br>(24·5–57·7)	73·7<br>(67·2–82·2)	-27·4<br>(-35·2–-18·8)
Suriname	0·268<br>(0·217–0·334)	168·5<br>(107·2–233·8)	43·3<br>(35·0–53·8)	9·1<br>(-15·2–36·1)
Trinidad and Tobago	1·90<br>(1·51–2·38)	82·0<br>(43·5–129·4)	98·6<br>(78·4–123·0)	-24·5<br>(-40·2–-5·2)
Virgin Islands	0·0676<br>(0·0544–0·0835)	98·7<br>(47·9–154·1)	38·7<br>(31·9–47·7)	-15·8<br>(-36·9–7·1)
Central Latin America	121<br>(112–132)	219·6<br>(193·5–246·0)	48·9<br>(45·1–53·3)	0·8<br>(-7·4–9·0)
Colombia	7·47<br>(6·49–8·64)	115·3<br>(87·8–153·8)	13·0<br>(11·2–15·0)	-39·2<br>(-47·4–-28·4)
Costa Rica	1·04<br>(0·942–1·17)	312·4<br>(274·9–363·6)	19·1<br>(17·3–21·5)	28·3<br>(16·9–44·0)
El Salvador	2·70<br>(2·30–3·39)	309·8<br>(244·2–417·9)	43·0<br>(36·7–54·2)	91·4<br>(61·7–142·5)
Guatemala	6·69<br>(5·80–7·68)	970·6<br>(832·1–1150·6)	63·9<br>(55·7–73·1)	242·9<br>(196·9–298·5)
Honduras	1·82<br>(1·42–2·38)	478·8<br>(345·8–660·4)	30·0<br>(23·7–39·0)	90·6<br>(48·5–147·2)
Mexico	85·6<br>(78·0–92·4)	196·9<br>(167·2–219·2)	70·3<br>(64·2–75·8)	-2·5<br>(-12·2–4·7)
Nicaragua	1·46<br>(1·24–1·74)	292·4<br>(220·8–378·9)	33·9<br>(28·9–40·6)	33·7<br>(9·1–61·4)
Panama	1·28<br>(1·05–1·50)	320·7<br>(240·2–404·0)	28·4<br>(23·3–33·3)	34·8<br>(8·7–61·4)
Venezuela	13·4<br>(11·0–16·7)	322·6<br>(242·8–436·7)	44·6<br>(36·7–55·6)	28·3<br>(3·6–63·2)
Tropical Latin America	68·4<br>(62·8–71·8)	153·1<br>(143·5–162·6)	27·2<br>(24·9–28·6)	-17·0<br>(-19·7–-14·4)
Brazil	65·2<br>(59·8–68·4)	146·2<br>(137·0–155·8)	26·5<br>(24·2–27·9)	-19·5<br>(-22·3–-16·8)
Paraguay	3·23<br>(2·57–4·07)	486·4<br>(350·6–686·0)	57·1<br>(45·6–71·9)	119·1<br>(68·4–191·6)
**North Africa and Middle East**	**117<br>(103–133)**	**229·6<br>(179·8–270·6)**	**29·0<br>(25·5–32·6)**	**20·7<br>(3·4–35·7)**
North Africa and Middle East	117<br>(103–133)	229·6<br>(179·8–270·6)	29·0<br>(25·5–32·6)	20·7<br>(3·4–35·7)
Afghanistan	5·04<br>(3·88–6·38)	178·8<br>(104·8–251·1)	41·1<br>(32·2–52·1)	58·5<br>(17·7–104·0)
Algeria	5·71<br>(4·50–6·97)	383·0<br>(270·5–512·0)	19·0<br>(15·3–23·3)	47·3<br>(8·5–78·3)

Bahrain	0·739 (0·617–0·851)	466·7 (354·5–620·3)	109·9 (92·2–124·8)	6·4 (-12·0–34·1)
Egypt	27·9 (22·8–33·9)	318·2 (242·5–406·0)	48·7 (40·2–57·6)	95·2 (62·0–132·8)
Iran	13·5 (11·8–14·5)	375·2 (288·6–436·3)	19·4 (16·8–21·1)	37·3 (12·1–55·9)
Iraq	10·4 (7·66–12·7)	235·3 (150·5–322·5)	49·6 (37·8–59·3)	17·6 (-11·1–47·9)
Jordan	2·45 (1·97–2·98)	272·9 (179·3–382·6)	41·1 (33·3–49·1)	-32·2 (-47·4–-11·6)
Kuwait	0·592 (0·501–0·703)	438·6 (352·2–543·2)	26·9 (22·4–31·8)	19·6 (0·7–42·8)
Lebanon	1·37 (0·977–1·69)	84·2 (33·5–135·2)	25·4 (18·1–31·3)	-31·6 (-49·8–-12·7)
Libya	1·25 (0·902–1·61)	422·9 (258·0–606·5)	24·7 (17·9–31·9)	79·8 (24·0–140·6)
Morocco	7·16 (5·32–8·58)	360·8 (232·1–485·9)	24·2 (18·3–29·1)	95·9 (43·3–139·6)
Oman	0·746 (0·622–0·871)	199·2 (104·6–295·2)	50·2 (42·2–58·5)	19·0 (-16·2–58·4)
Palestine	1·06 (0·918–1·19)	162·6 (94·6–221·6)	54·1 (46·5–60·7)	2·7 (-23·5–25·4)
Qatar	0·433 (0·328–0·564)	652·0 (398·0–935·4)	57·0 (45·5–70·4)	-29·5 (-50·5–-6·8)
Saudi Arabia	5·22 (4·21–6·32)	327·6 (162·6–485·4)	30·6 (24·9–35·8)	30·3 (-15·7–70·2)
Sudan	3·43 (2·70–4·41)	202·2 (119·5–294·3)	19·2 (15·2–24·1)	51·4 (13·2–93·3)
Syria	2·17 (1·64–2·86)	147·2 (72·0–255·4)	20·5 (15·9–25·7)	10·4 (-21·7–56·1)
Tunisia	2·06 (1·48–2·60)	341·8 (198·2–479·5)	16·4 (12·0–20·7)	54·7 (4·4–97·1)
Türkiye	23·3 (17·8–28·4)	106·7 (53·2–154·3)	26·4 (20·1–32·2)	-26·0 (-44·6–-8·7)
United Arab Emirates	0·844 (0·646–1·07)	546·9 (305·5–725·2)	43·7 (34·6–53·2)	-5·2 (-36·8–15·3)
Yemen	1·93 (1·41–2·83)	240·8 (154·8–363·4)	15·4 (11·5–22·0)	24·8 (-6·5–66·8)
**South Asia**	**444 (396–480)**	**278·2 (224·5–338·8)**	**33·8 (30·3–36·6)**	**31·3 (12·8–52·1)**
South Asia	444 (396–480)	278·2 (224·5–338·8)	33·8 (30·3–36·6)	31·3 (12·8–52·1)
Bangladesh	42·8 (35·6–51·7)	263·6 (179·1–360·5)	35·8 (29·9–43·0)	17·0 (-8·1–49·2)
Bhutan	0·176 (0·131–0·224)	253·1 (159·9–392·3)	34·0 (25·6–43·0)	41·3 (6·3–100·3)
India	348 (309–381)	292·4 (224·8–369·4)	32·2 (28·7–35·2)	37·2 (14·8–65·5)
Nepal	6·76 (5·34–8·24)	288·7 (195·4–443·4)	33·6 (26·9–41·1)	53·4 (14·4–114·3)
Pakistan	45·9 (39·2–55·9)	209·6 (156·4–289·6)	47·7 (40·7–57·5)	65·4 (38·2–106·8)
**Southeast Asia, east Asia, and Oceania**	**403 (367–444)**	**167·3 (137·3–205·1)**	**14·8 (13·4–16·2)**	**-1·3 (-11·7–12·9)**
East Asia	197 (162–229)	146·4 (92·9–191·6)	9·3 (7·7–10·7)	-9·8 (-28·8–6·0)
China	181 (147–214)	150·1 (91·2–199·9)	8·8 (7·1–10·3)	-8·6 (-29·6–8·9)

North Korea	4·85 (3·78–5·96)	140·6 (75·2–208·8)	14·8 (11·6–18·3)	5·1 (-21·9–32·7)
Taiwan (province of China)	10·7 (9·39–11·3)	100·4 (83·7–115·2)	25·0 (22·1–26·6)	-32·5 (-37·5–-27·8)
Oceania	7·46 (6·35–8·83)	171·1 (105·2–232·1)	111·3 (95·6–129·4)	8·2 (-17·0–31·4)
American Samoa	0·0534 (0·0456–0·0622)	183·0 (122·6–259·1)	114·9 (98·3–132·7)	22·4 (-3·6–52·4)
Cook Islands	0·0302 (0·0258–0·0352)	75·7 (34·7–113·8)	114·8 (97·6–135·0)	-22·6 (-39·9–-7·2)
Federated States of Micronesia	0·0772 (0·0601–0·100)	106·2 (58·5–170·6)	118·7 (93·8–148·4)	36·2 (5·8–76·8)
Fiji	1·85 (1·49–2·28)	189·4 (112·6–271·2)	268·5 (221·4–325·3)	35·9 (1·3–72·0)
Guam	0·0478 (0·0421–0·0530)	79·3 (54·4–106·6)	24·1 (21·2–26·6)	-44·3 (-51·3–-35·5)
Kiribati	0·116 (0·0880–0·145)	143·3 (79·2–218·1)	185·0 (143·9–221·6)	31·5 (-1·5–63·6)
Marshall Islands	0·0549 (0·0375–0·0727)	241·0 (136·5–323·4)	166·9 (117·4–221·9)	54·2 (9·7–86·7)
Nauru	0·00576 (0·00441–0·00731)	44·3 (9·7–99·8)	146·5 (116·5–181·2)	25·4 (-0·7–67·8)
Niue	0·00263 (0·00204–0·00323)	41·9 (4·5–76·3)	120·7 (93·8–147·7)	47·3 (8·0–83·2)
Northern Mariana Islands	0·0337 (0·0268–0·0397)	242·2 (154·3–322·6)	64·4 (52·2–74·2)	-5·7 (-26·2–15·9)
Palau	0·0210 (0·0170–0·0266)	179·7 (113·6–288·2)	108·0 (88·8–133·6)	29·0 (0·6–75·0)
Papua New Guinea	4·11 (3·23–5·10)	174·6 (83·1–284·6)	90·1 (72·5–111·9)	1·2 (-30·9–36·3)
Samoa	0·133 (0·106–0·160)	114·3 (63·8–174·7)	94·0 (76·2–111·8)	22·6 (-5·7–53·4)
Solomon Islands	0·332 (0·259–0·421)	227·4 (119·1–366·5)	111·9 (91·2–138·2)	33·9 (-6·8–77·8)
Tokelau	0·00122 (0·000998–0·00154)	18·0 (-9·4–51·4)	85·2 (69·4–106·0)	9·9 (-15·2–40·6)
Tonga	0·0894 (0·0737–0·110)	88·7 (45·7–145·9)	113·2 (93·6–138·6)	23·8 (-4·2–58·6)
Tuvalu	0·0101 (0·00805–0·0124)	79·4 (44·8–120·8)	101·8 (83·0–123·3)	15·4 (-6·2–39·4)
Vanuatu	0·152 (0·123–0·181)	261·2 (176·2–380·4)	89·9 (74·9–106·1)	26·7 (-0·3–65·5)
Southeast Asia	199 (183–219)	192·7 (155·6–228·5)	33·7 (30·8–36·9)	15·9 (1·4–30·0)
Cambodia	3·89 (2·92–5·06)	211·8 (128·2–304·7)	33·4 (25·5–42·9)	15·0 (-13·6–49·7)
Indonesia	60·0 (50·1–67·9)	215·4 (159·2–266·7)	29·6 (24·9–33·2)	44·0 (17·8–70·2)
Laos	1·57 (1·24–2·00)	112·4 (57·8–199·8)	37·3 (29·8–47·0)	0·4 (-23·8–38·0)
Malaysia	5·31 (4·76–5·86)	123·8 (91·1–154·3)	19·9 (17·9–22·1)	-28·6 (-39·0–-18·3)
Maldives	0·0563 (0·0445–0·0660)	93·4 (46·0–148·0)	19·2 (15·4–22·7)	-46·9 (-59·5–-32·0)
Mauritius	1·95 (1·84–2·04)	403·4 (374·3–436·1)	106·7 (100·1–112·0)	101·2 (89·9–113·6)
Myanmar	25·8 (21·0–32·1)	99·7 (43·7–175·8)	58·1 (47·8–71·9)	-1·7 (-29·1–34·1)

Philippines	31·9 (30·4–33·8)	310·5 (273·1–354·4)	41·4 (39·5–44·3)	42·1 (29·0–56·8)
Seychelles	0·0266 (0·0233–0·0301)	180·8 (141·8–237·3)	25·1 (22·1–28·2)	49·4 (30·1–80·5)
Sri Lanka	12·3 (8·79–16·2)	249·7 (136·1–369·5)	48·9 (35·2–64·1)	25·2 (-14·6–64·7)
Thailand	24·3 (19·4–29·7)	201·2 (119·6–315·0)	22·4 (17·9–27·4)	-8·0 (-33·5–27·2)
Timor-Leste	0·187 (0·146–0·250)	278·2 (172·4–423·3)	23·4 (18·3–30·8)	21·4 (-9·3–67·3)
Viet Nam	31·3 (25·5–37·0)	176·3 (102·8–258·8)	36·6 (29·9–42·9)	19·2 (-10·8–54·9)
**Sub-Saharan Africa**	**201 (182–219)**	**155·1 (123·0–180·7)**	**47·9 (43·7–51·9)**	**16·0 (1·4–27·1)**
Central sub-Saharan Africa	25·7 (20·6–30·3)	154·5 (96·5–218·4)	52·9 (42·8–61·9)	1·8 (-21·5–23·3)
Angola	5·09 (3·91–6·43)	209·4 (117·4–322·6)	49·8 (39·6–60·6)	3·3 (-24·7–36·5)
Central African Republic	1·25 (0·944–1·53)	93·0 (42·6–148·5)	64·1 (49·0–77·0)	3·0 (-20·5–28·3)
Congo (Brazzaville)	1·57 (1·31–1·87)	151·2 (100·1–227·7)	65·4 (56·3–77·8)	-1·2 (-18·2–24·4)
DR Congo	16·7 (12·5–20·3)	149·6 (85·0–231·8)	51·4 (39·0–61·6)	1·7 (-24·6–32·0)
Equatorial Guinea	0·281 (0·207–0·385)	183·7 (99·8–298·0)	63·2 (47·1–82·8)	14·2 (-17·6–55·8)
Gabon	0·735 (0·545–0·926)	129·5 (76·7–204·5)	75·6 (56·8–93·0)	20·5 (-4·9–57·2)
Eastern sub-Saharan Africa	65·0 (57·7–73·5)	101·7 (78·2–135·1)	43·2 (38·2–48·6)	-8·3 (-18·5–5·3)
Burundi	1·88 (1·34–2·77)	62·2 (19·7–117·1)	45·8 (31·6–66·2)	-13·6 (-36·5–14·1)
Comoros	0·211 (0·150–0·274)	126·3 (59·5–205·3)	45·3 (32·6–58·4)	-0·8 (-28·4–29·2)
Djibouti	0·248 (0·190–0·341)	478·5 (332·6–678·2)	48·5 (38·7–64·2)	32·3 (-1·4–68·4)
Eritrea	1·32 (0·969–1·69)	204·5 (138·1–276·7)	56·4 (42·4–69·8)	13·1 (-6·2–33·1)
Ethiopia	15·1 (13·3–17·2)	25·4 (3·7–56·2)	39·2 (34·5–44·5)	-41·4 (-51·5–-28·9)
Kenya	7·55 (6·42–8·97)	278·1 (207·0–376·7)	38·5 (32·9–45·7)	38·9 (12·2–76·0)
Madagascar	3·54 (2·72–4·58)	128·7 (71·4–201·6)	36·3 (28·4–46·1)	12·0 (-13·5–45·1)
Malawi	3·43 (2·69–4·03)	122·5 (79·9–173·6)	49·3 (39·6–58·1)	10·2 (-9·5–32·2)
Mozambique	5·30 (4·11–6·51)	151·8 (90·7–217·2)	50·8 (39·9–61·8)	30·3 (-1·1–60·3)
Rwanda	2·38 (1·48–3·42)	53·2 (11·4–95·6)	44·2 (28·4–62·6)	-24·8 (-43·9–-5·6)
Somalia	3·62 (2·79–4·72)	203·3 (136·0–285·5)	59·3 (46·9–74·4)	11·6 (-10·3–39·6)
South Sudan	2·07 (1·57–2·85)	109·3 (51·5–200·8)	58·2 (44·3–79·4)	28·2 (-5·6–77·3)
Tanzania	9·14 (7·53–11·2)	147·3 (102·4–211·6)	39·4 (32·5–47·7)	4·5 (-13·0–31·4)
Uganda	6·05 (4·36–8·56)	160·9 (81·4–246·0)	47·0 (33·9–64·1)	16·1 (-17·4–51·9)

Zambia	3·07 (2·40–3·89)	141·2 (79·9–209·8)	48·9 (39·0–59·1)	-0·2 (-24·8–24·6)
Southern sub-Saharan Africa	41·1 (38·5–43·3)	270·7 (234·3–307·0)	77·6 (72·3–81·9)	76·8 (60·2–93·4)
Botswana	0·728 (0·612–0·853)	153·0 (85·5–234·3)	61·6 (51·6–71·7)	6·8 (-20·6–39·9)
Eswatini	0·668 (0·519–0·894)	229·2 (151·5–374·7)	126·0 (100·3–164·1)	58·2 (21·8–124·9)
Lesotho	1·18 (0·913–1·50)	182·7 (113·5–305·8)	103·1 (80·2–128·8)	123·6 (67·8–212·0)
Namibia	0·972 (0·754–1·25)	142·3 (85·4–204·7)	73·7 (57·6–92·9)	23·0 (-3·0–54·5)
South Africa	33·2 (31·1–35·0)	292·1 (254·3–328·5)	77·7 (72·8–82·5)	79·3 (62·8–95·7)
Zimbabwe	4·38 (3·40–5·49)	239·0 (151·6–347·3)	69·4 (54·5–84·6)	85·8 (39·3–136·3)
Western sub-Saharan Africa	69·4 (59·0–80·0)	173·9 (121·7–220·7)	40·8 (35·1–46·0)	23·6 (1·8–41·9)
Benin	1·67 (1·36–2·06)	218·2 (138·4–287·5)	36·9 (30·4–44·2)	32·2 (4·1–63·1)
Burkina Faso	2·85 (2·34–3·43)	114·1 (56·5–167·4)	33·7 (28·0–40·3)	-5·5 (-31·6–17·1)
Cabo Verde	0·156 (0·128–0·187)	483·1 (364·1–615·0)	36·3 (29·9–43·4)	223·7 (160·8–304·3)
Cameroon	5·60 (4·35–7·63)	273·0 (182·5–428·7)	51·4 (40·9–67·7)	31·8 (-0·4–86·7)
Chad	1·96 (1·48–2·71)	220·4 (144·4–304·8)	36·9 (28·0–49·5)	58·4 (20·3–96·8)
Côte d'Ivoire	3·93 (3·05–4·97)	268·4 (180·8–384·7)	42·1 (34·1–52·4)	31·4 (3·2–74·9)
The Gambia	0·410 (0·305–0·518)	385·0 (259·6–519·5)	45·1 (34·0–56·7)	64·2 (25·4–109·8)
Ghana	7·19 (5·70–8·79)	391·4 (240·0–580·5)	49·0 (39·5–59·6)	78·4 (26·6–143·9)
Guinea	2·12 (1·71–2·74)	140·5 (79·4–224·9)	41·6 (33·9–53·1)	43·4 (8·4–92·0)
Guinea-Bissau	0·362 (0·294–0·451)	128·0 (72·3–202·2)	56·0 (46·6–68·1)	31·7 (2·1–72·9)
Liberia	0·821 (0·608–1·14)	161·2 (96·1–244·6)	43·9 (32·7–59·1)	38·0 (6·6–79·8)
Mali	3·43 (2·84–4·28)	182·9 (124·2–255·4)	43·8 (36·9–53·4)	29·1 (4·7–60·4)
Mauritania	0·750 (0·559–0·978)	171·7 (110·0–273·4)	38·7 (29·3–49·8)	25·9 (-0·6–71·3)
Niger	1·98 (1·45–2·72)	258·9 (169·5–369·8)	28·3 (20·9–38·2)	27·5 (-0·4–60·5)
Nigeria	30·5 (24·1–36·1)	124·7 (68·0–187·4)	39·2 (32·0–45·6)	10·8 (-15·2–36·5)
São Tomé and Príncipe	0·0182 (0·0148–0·0213)	117·8 (73·3–169·0)	18·9 (15·8–22·2)	31·8 (8·3–57·9)
Senegal	3·30 (2·58–4·20)	259·7 (167·6–356·4)	48·2 (37·8–60·5)	52·4 (16·7–91·6)
Sierra Leone	1·12 (0·913–1·45)	164·1 (105·1–258·9)	33·5 (27·7–42·9)	39·2 (9·4–87·7)
Togo	1·19 (0·904–1·55)	333·6 (236·2–460·1)	36·5 (28·3–45·6)	45·8 (14·4–82·9)

Table S25. Total diabetes and type 1 diabetes nonfatal input data source citations

Citation	Model
Aamodt G, Stene LC, Njolstad PR, Sovik O, Joner G. Spatiotemporal trends and age-period-cohort modeling of the incidence of type 1 diabetes among children aged Diabetes Care. 2007; 30(4): 884-9.	Total diabetes, type 1 diabetes
Abdella N, Al Arouj M, Al Nakhi A, Al Assoussi A, Moussa M. Non-insulin-dependent diabetes in Kuwait: prevalence rates and associated risk factors. Diabetes Res Clin Pract. 1998; 42(3): 187-96.	Total diabetes
Abdul-Rasoul M, Al-Qattan H, Al-Haj A, Habib H, Ismael A. Incidence and seasonal variation of Type 1 diabetes in children in Farwania area, Kuwait (1995-1999). Diabetes Res Clin Pract. 2002; 56(2): 153-7.	Total diabetes, type 1 diabetes
Abebe SM, Berhane Y, Worku A, Assefa A. Diabetes mellitus in North West Ethiopia: a community based study. BMC Public Health. 2014; 97.	Total diabetes
Abu Sham'a R a. H, Darwazah AK, Kufri FH, Yassin IH, Torok NI. MetS and cardiovascular risk factors among Palestinians of East Jerusalem. East Mediterr Health J. 2009; 15(6): 1464–73.	Total diabetes
Achutha Menon Center for Health Sciences Studies, Sree Chitra Tirunal Institute for Medical Sciences and Technology (India), Kerala Directorate of Health Services (India), World Health Organization (WHO). India - Kerala Noncommunicable Disease Risk Factors Survey 2016-2017.	Total diabetes
Adegoke OA, Adedoyin RA, Balogun MO, Adebayo RA, Bisiriyu LA, Salawu AA. Prevalence of metabolic syndrome in a rural community in Nigeria. Metab Syndr Relat Disord. 2010; 8(1): 59–62.	Total diabetes
Adeleke SI, Asani MO, Belonwu RO, Gwarzo GD, Farouk ZL. Childhood diabetes mellitus in Kano, North West, Nigeria. Niger J Med. 2010; 19(2): 145-7.	Total diabetes
Administrative Department of Science, Technology, and Innovation (Colombia), Center for Development Projects, Pontifical Xavierian University, Ministry of Social Protection (Colombia), Specialized Information Systems. Colombia National Health Survey 2007-2008.	Total diabetes
Aga Khan University, All India Institute of Medical Sciences, New Delhi (AIIMS), Centre for Chronic Disease Control (India), Emory University (United States), Madras Diabetes Research Foundation, National Institute of Mental Health and Neurosciences (India), Public Health Foundation of India. Center for Cardio-Metabolic Risk Reduction in South Asia Surveillance Study 2010-2011.	Total diabetes
Aga Khan University, All India Institute of Medical Sciences, New Delhi (AIIMS), Madras Diabetes Research Foundation, Public Health Foundation of India, Rollins School of Public Health, Emory University (United States). Center for Cardiometabolic Risk Reduction in South Asia Surveillance Study First Cohort Follow-up 2013.	Total diabetes
Agency of Health Research and Development (Indonesia). Indonesia Basic Health Research 2007-2008.	Total diabetes
Ajlouni K, Jaddou H, Batieha A. Diabetes and impaired glucose tolerance in Jordan: prevalence and associated risk factors. J Intern Med. 1998; 244(4): 317-23.	Total diabetes
Ajlouni K, Qusous Y, Khawaldeh AK, Jaddou H, Batiehah A, Ammari F, Zaheri M, Mashal A. Incidence of insulin-dependent diabetes mellitus in Jordanian children aged 0-14 y during 1992-1996. Acta Paediatr Suppl. 1999; 88(427): 11-3.	Total diabetes, type 1 diabetes
Akesen E, Turan S, Güran T, Atay Z, Save D, Bereket A. Prevalence of type 1 diabetes mellitus in 6-18-yr-old school children living in Istanbul, Turkey. Pediatr Diabetes. 2011; 12(6): 567-71.	Total diabetes, type 1 diabetes
Al Zenki S, Al Omirah H, Al Hooti S, Al Hamad N, Jackson RT, Rao A, Al Jahmah N, Al Obaid I, Al Ghanim J, Al Somaie M, Zaghloul S, Al Othman A. High prevalence of metabolic syndrome among Kuwaiti adults--a wake-up call for public health intervention. Int J Environ Res Public Health. 2012; 9(5): 1984-96.	Total diabetes
Alarouj M, Bennakhi A, Alnesef Y, Sharifi M, Elkum N. Diabetes and associated cardiovascular risk factors in the State of Kuwait: the first national survey. Int J Clin Pract. 2013; 67(1): 89-96.	Total diabetes
Alberts M, Urdal P, Steyn K, Stensvold I, Tverdal A, Nel JH, Steyn NP. Prevalence of cardiovascular diseases and associated risk factors in a rural black population of South Africa. Eur J Prev Cardiol. 2005; 12(4): 347-54.	Total diabetes
Al-Daghri NM, Al-Attas OS, Alokail MS, Alkharfy KM, Yousef M, Sabico SL, Chrousos GP. Diabetes mellitus type 2 and other chronic non-communicable diseases in the central region, Saudi Arabia (Riyadh cohort 2): a decade of an epidemic. BMC Med. 2011; 76.	Total diabetes, type 1 diabetes
Al-Habori M, Al-Mamari M, Al-Meeri A. Type II diabetes mellitus and impaired glucose tolerance in Yemen: prevalence, associated metabolic changes and risk factors. Diabetes Res Clin Pract. 2004; 65(3): 275-81.	Total diabetes
Al-Herbish AS, El-Mouzan MI, Al-Salloum AA, Al-Qurachi MM, Al-Omar AA. Prevalence of type 1 diabetes mellitus in Saudi Arabian children and adolescents. Saudi Med J. 2008; 29(9): 1285-8.	Total diabetes
Alikor CA, Emem-Chioma PC. EPIDEMIOLOGY OF DIABETES AND IMPAIRED FASTING GLUCOSE IN A RURAL COMMUNITY OF NIGERIAN NIGER DELTA REGION. Niger J Med. 2015; 24(2): 114–24.	Total diabetes
Allal-Elasmi M, Feki M, Zayani Y, Hsairi M, Haj Taieb S, Jemaa R, Sanhaji H, Omar S, Mebazaa A, Kaabachi N. Prehypertension among adults in Great Tunis region (Tunisia): A population-based study. Pathol Biol. 2012; 60(3): 174–9.	Total diabetes
Allal-Elasmi M, Haj Taieb S, Hsairi M, Zayani Y, Omar S, Sanhaji H, Jemaa R, Feki M, Elati J, Mebazaa A, Kaabachi N. The metabolic syndrome: prevalence, main characteristics and association with socio-economic status in adults living in Great Tunis. Diabetes Metab. 2010; 36(3): 204–8.	Total diabetes
Al-Lawati JA, Mohammed AJ, Al-Hinai HQ, Jousilahti P. Prevalence of the metabolic syndrome among Omani adults. Diabetes Care. 2003; 26(6): 1781-5.	Total diabetes
Al-Mendalawi MD, Abduljabbar MA, Aljubeh JM, Amalraj A, Cherian MP. Incidence trends of childhood type 1 diabetes in eastern Saudi Arabia. Saudi Med J. 2010; 31(9): 1074-1075.	Total diabetes, type 1 diabetes
Al-Nozha MM, Al-Maatouq MA, Al-Mazrou YY, Al-Harthi SS, Arafah MR, Khalil MZ, Khan NB, Al-Khadra A, Al-Marzouki K, Nouh MS, Abdullah M, Attas O, Al-Shahid MS, Al-Mobeireek A. Diabetes mellitus in Saudi Arabia. Saudi Med J. 2004; 25(11): 1603-10.	Total diabetes
Al-Rubeaan K, Al-Manaa H, Khoja T, Ahmad N, Al-Sharqawi A, Siddiqui K, AlNaqeb D, Aburisheh K, Youssef A, Al-Batil A, Al-Otaibi M, Ghamdi AA. The Saudi Abnormal Glucose Metabolism and Diabetes Impact Study (SAUDI-DM). Ann Saudi Med. 2014; 34(6): 465–75.	Type 1 diabetes
Altobelli E, Chiarelli F, Valenti M, Verrotti A, Tumini S, Di Orio F. Incidence of insulin-dependent diabetes mellitus (0-14 years) in the Abruzzo Region, Italy, 1990-1995: results from a population-based register. J Pediatr Endocrinol Metab. 1998; 11(4): 555-62.	Total diabetes, type 1 diabetes
American University of Beirut, World Health Organization (WHO). Lebanon STEPS Noncommunicable Disease Risk Factors Survey 2008-2009.	Total diabetes
Amini M, Afshin-Nia F, Bashardoost N, Aminorroaya A, Shahparian M, Kazemi M. Prevalence and risk factors of diabetes mellitus in the Isfahan city population (aged 40 or over) in 1993. Diabetes Res Clin Pract. 1997; 38(3): 185-90.	Total diabetes
Amoah AGB, Owusu SK, Adjei S. Diabetes in Ghana: a community based prevalence study in Greater Accra. Diabetes Res Clin Pract. 2002; 56(3): 197-205.	Total diabetes

Analytical and Information Center of the Ministry of Health of Uzbekistan, Macro International, Inc, Ministry of Macroeconomics and Statistics (Uzbekistan). Uzbekistan Special Demographic and Health Survey 2002. Fairfax, United States of America: ICF International.	Total diabetes
Anokute CC. Epidemiologic studies of diabetes mellitus in Saudi Arabia--Part I--Screening of 3158 males in King Saud University. J R Soc Health. 1990; 110(6): 201-3.	Total diabetes
Arab M. Diabetes mellitus in Egypt. World Health Stat Q. 1992; 45(4): 334-7.	Total diabetes
Aregbesola A, Voutilainen S, Virtanen JK, Mursu J, Tuomainen TP. Gender difference in type 2 diabetes and the role of body iron stores. Ann Clin Biochem. 2017; 54(1): 113-120.	Total diabetes
Arpi ML, Fichera G, Mancuso M, Lucenti C, Italia S, Tomaselli L, Motta RM, Mazza A, Vigneri R, Purrello F, Squatrito S. A ten-year (1989-1998) perspective study of the incidence of Type 1 diabetes in the district of Catania (Sicily) in a 0-14 year age group. J Endocrinol Invest. 2002; 25(5): 414-9.	Total diabetes, type 1 diabetes
Arpi ML, Italia S, Motta RM, Raimondo M, Mancuso M, Tomaselli L, Squatrito S, Vigneri R, Purrello F. Incidence of type I diabetes in the district of Catania, Sicily. Acta Diabetol. 1994; 31(1): 37-9.	Total diabetes, type 1 diabetes
Aschner P, King H, Triana de Torrado M, Rodriguez BM. Glucose intolerance in Colombia. A population-based survey in an urban community. Diabetes Care. 1993; 16(1): 90-3.	Total diabetes
Asfour MG, Lambourne A, Soliman A, Al-Behlani S, Al-Asfoor D, Bold A, Mahtab H, King H. High prevalence of diabetes mellitus and impaired glucose tolerance in the Sultanate of Oman: results of the 1991 national survey. Diabet Med. 1995; 12(12): 1122-5.	Total diabetes
Asmar R, Vol S, Pannier B, Brisac AM, Tichet J, El Hasnaoui A. High blood pressure and associated cardiovascular risk factors in France. J Hypertens. 2001; 19(10): 1727-32.	Total diabetes
Aspray TJ, Mugusi F, Rashid S, Whiting D, Edwards R, Alberti KG, Unwin NC, Essential Non-Communicable Disease Health Intervention Project. Rural and urban differences in diabetes prevalence in Tanzania: the role of obesity, physical inactivity and urban living. Trans R Soc Trop Med Hyg. 2000; 94(6): 637-44.	Total diabetes
Associates for Community and Population Research (ACPR), International Centre for Diarrhoeal Disease Research, Bangladesh (ICDDR,B), MEASURE Evaluation Project, Carolina Population Center, University of North Carolina, National Institute of Population Research and Training (NIPORT). Bangladesh Urban Health Survey 2006.	Total diabetes
Australian Agency for International Development (AusAID), Department of Health (Tokelau), Fiji School of Medicine, World Health Organization (WHO). Tokelau STEPS Noncommunicable Disease Risk Factors Survey 2005.	Total diabetes
Australian Agency for International Development (AusAID), Fiji School of Medicine, Menzies Center for Population Health Research, University of Tasmania (Australia), Ministry of Health (Fiji), World Health Organization (WHO). Fiji STEPS Noncommunicable Disease Risk Factors Survey 2002.	Total diabetes
Australian Institute of Health and Welfare, National Diabetes Register (NDR) (Australia). Australia Incidence of Insulin-Treated Diabetes 2014. Canberra, Australia: Australian Institute of Health and Welfare, 2016.	Total diabetes, type 1 diabetes
Australian Institute of Health and Welfare, National Diabetes Register (NDR) (Australia). Australia Incidence of Insulin-Treated Diabetes 2015. Canberra, Australia: Australian Institute of Health and Welfare, 2017.	Total diabetes, type 1 diabetes
Azerbaijan Ministry of Health, World Health Organization (WHO). Azerbaijan STEPS Noncommunicable Disease Risk Factors Survey 2017.	Total diabetes
Azimi-Nezhad M, Ghayour-Mobarhan M, Parizadeh MR, Safarian M, Esmaeili H, Parizadeh SM, Khodaee G, Hosseini J, Abasalti Z, Hassankhani B, Ferns G. Prevalence of type 2 diabetes mellitus in Iran and its relationship with gender, urbanisation, education, marital status and occupation. Singapore Med J. 2008; 49(7): 571-6.	Total diabetes
Azizi F, Rahmani M, Emami H, Mirmiran P, Hajipour R, Madjid M, Ghanbili J, Ghanbarian A, Mehrabi Y, Saadat N, Salehi P, Mortazavi N, Heydarian P, Sarbazi N, Allahverdian S, Saadati N, Ainy E, Moeini S. Cardiovascular risk factors in an Iranian urban population: Tehran lipid and glucose study (phase 1). Soz Praventivmed. 2002; 47(6): 408-26.	Total diabetes
Bahíllo MP, Hermoso F, Ochoa C, García-Fernández JA, Rodrigo J, Marugán JM, de la Torre S, Manzano F, Lema T, García-Velázquez J, Castilla-León Childhood Type 1 Diabetes Epidemiology Study Group. Incidence and prevalence of type 1 diabetes in children aged Pediatr Diabetes. 2007; 8(6): 369-73.	Total diabetes, type 1 diabetes
Baker IDI Heart and Diabetes Institute, Imperial College London, Ministry of Health and Quality of Life (Mauritius), National Public Health Institute (Finland), Umeå University. Mauritius Noncommunicable Disease Survey 2009.	Total diabetes
Balagopal P, Kamalamma N, Patel TG, Misra R. A community-based participatory diabetes prevention and management intervention in rural India using community health workers. Diabetes Educ. 2012; 38(6): 822-34.	Total diabetes
Bald, NM, Diallo I, Bald, MD, Barry IS, Kaba L, Diallo MM, Kak, A, Camara A, Bah D, Barry MM, Sangar,-Bah M, Maugendre D. Diabetes and impaired fasting glucose in rural and urban populations in Futa Jallon (Guinea): prevalence and associated risk factors. Diabetes Metab. 2007; 33(2): 114-20.	Total diabetes
Baragou S, Djibril M, Atta B, Damorou F, Pio M, Balogou A. Prevalence of cardiovascular risk factors in an urban area of Togo: a WHO STEPS-wise approach in Lome, Togo. Cardiovasc J Afr. 2012; 23(6): 309-12.	Total diabetes
Barat P, Valade A, Brosselin P, Alberti C, Maurice-Tison S, L,vy-Marchal C. The growing incidence of type 1 diabetes in children: the 17-year French experience in Aquitaine. Diabetes Metab. 2008; 34(6 Pt 1): 601-5.	Total diabetes, type 1 diabetes
Barbagallo CM, Cavera G, Sapienza M, Noto D, Cefalù AB, Pagano M, Montalto G, Notarbartolo A, Averna MR. Prevalence of overweight and obesity in a rural southern Italy population and relationships with total and cardiovascular mortality: the Ventimiglia di Sicilia project. Int J Obes (Lond). 2001; 25(2): 185-90.	Total diabetes
Barcelo A, Daroca MC, Ribera R, Duarte E, Zapata A, Vohra M. Diabetes in Bolivia. Rev Panam Salud Publica. 2001; 10(5): 318-23.	Total diabetes
Basit A, Hydrie MZ, Ahmed K, Hakeem R. Prevalence of diabetes, impaired fasting glucose and associated risk factors in a rural area of Baluchistan province according to new ADA criteria. J Pak Med Assoc. 2002; 52(8): 357-60.	Total diabetes
Battelino T, Krzisnik C. Incidence of type 1 diabetes mellitus in children in Slovenia during the years 1988-1995. Acta Diabetol. 1998; 35(2): 112-4.	Total diabetes, type 1 diabetes
Bautista LE, Oróstegui M, Vera LM, Prada GE, Orozco LC, Herrán OF. Prevalence and impact of cardiovascular risk factors in Bucaramanga, Colombia: results from the Countrywide Integrated Noncommunicable Disease Intervention Programme (CINDI/CARMEN) baseline survey. Eur J Cardiovasc Prev Rehabil. 2006; 13(5): 769-75.	Total diabetes
Bayram F, Kocer D, Gundogan K, Kaya A, Demir O, Coskun R, Sabuncu T, Karaman A, Cesur M, Rizzo M, Toth PP, Gedik V. Prevalence of dyslipidemia and associated risk factors in Turkish adults. J Clin Lipidol. 2014; 8(2): 206–16.	Total diabetes

Belfki H, Ben Ali S, Aounallah-Skhiri H, Traissac P, Bougatef S, Maire B, Delpeuch F, Achour N, Ben Romdhane H. Prevalence and determinants of the metabolic syndrome among Tunisian adults: results of the Transition and Health Impact in North Africa (TAHINA) project. Public Health Nutr. 2013; 16(4): 582–90.	Total diabetes
Ben Khalifa F, Mekaouar A, Taktak S, Hamhoum M, Jebara H, Kodia A, Zouari B, Chakroun M. A five-year study of the incidence of insulin-dependent diabetes mellitus in young Tunisians (preliminary results). Diabetes Metab. 1997; 23(5): 395-401.	Total diabetes, type 1 diabetes
Bendas A, Rothe U, Kiess W, Kapellen TM, Stange T, Manuwald U, Salzsieder E, Holl RW, Schoffer O, Stahl-Pehe A, Giani G, Ehehalt S, Neu A, Rosenbauer J. Trends in Incidence Rates during 1999-2008 and Prevalence in 2008 of Childhood Type 1 Diabetes Mellitus in Germany--Model-Based National Estimates. PLoS One. 2015; 10(7): e0132716.	Total diabetes, type 1 diabetes
Bengtsson H, Bergqvist D, Ekberg O, Janzon L. A population based screening of abdominal aortic aneurysms (AAA). Eur J Vasc Surg. 1991; 5(1): 53-7.	Total diabetes
Berhan Y, Waernbaum I, Lind T, Mollsten A, Dahlquist G. Thirty years of prospective nationwide incidence of childhood type 1 diabetes: the accelerating increase by time tends to level off in Sweden. Diabetes. 2011; 60(2): 577-81.	Total diabetes, type 1 diabetes
Bermuda Diabetes Association, Bermuda Hospitals Board (BHB), Caribbean Public Health Agency (CARPHA), Ministry of Health, Seniors and Environment (Bermuda), World Health Organization (WHO). Bermuda STEPS Noncommunicable Disease Risk Factors Survey 2013-2014.	Total diabetes
Bernabe-Ortiz A, Carrillo-Larco RM, Gilman RH, Checkley W, Smeeth L, Miranda JJ. Contribution of modifiable risk factors for hypertension and type-2 diabetes in Peruvian resource-limited settings. J Epidemiol Community Health. 2016; 70(1): 49-55.	Total diabetes
Bharati DR, Pal R, Kar S, Rekha R, Yamuna TV, Basu M. Prevalence and determinants of diabetes mellitus in Puducherry, South India. J Pharm Bioallied Sci. 2011; 3(4): 513-8.	Total diabetes
Bidulescu A, Ferguson TS, Hambleton I, Younger-Coleman N, Francis D, Bennett N, Griswold M, Fox E, MacLeish M, Wilks R, Harris EN, Sullivan LW. Educational health disparities in hypertension and diabetes mellitus among African descent populations in the Caribbean and the USA: a comparative analysis from the Spanish town cohort (Jamaica) and the Jackson heart study (USA). Int J Equity Health. 2017; 16(1): 33.	Total diabetes
Binh TQ, Phuong PT, Nhung BT, Tung DD. Metabolic syndrome among a middle-aged population in the Red River Delta region of Vietnam. BMC Endocr Disord. 2014; 14: 77.	Total diabetes
Bizzarri C, Patera PI, Arnaldi C, Petrucci S, Bitti MLM, Scrocca R, Manfrini S, Portuesi R, Buzzetti R, Cappa M, Pozzilli P, Immunotherapy Diabetes (IMDIAB) Group. Incidence of type 1 diabetes has doubled in Rome and the Lazio region in the 0- to 14-year age-group: a 6-year prospective study (2004-2009). Diabetes Care. 2010; 33(11): e140.	Total diabetes, type 1 diabetes
Bjerregaard-Andersen M, Hansen L, da Silva LI, Joaquím LC, Hennild DE, Christiansen L, Aaby P, Benn CS, Christensen K, Sodemann M, Jensen DM, Beck-Nielsen H. Risk of metabolic syndrome and diabetes among young twins and singletons in Guinea-bissau. Diabetes Care. 2013; 36(11): 3549-56.	Total diabetes
Bo S, Durazzo M, Guidi S, Carello M, Sacerdote C, Silli B, Rosato R, Cassader M, Gentile L, Pagano G. Dietary magnesium and fiber intakes and inflammatory and metabolic indicators in middle-aged subjects from a population-based cohort. Am J Clin Nutr. 2006; 84(5): 1062-9.	Total diabetes
Borissova AM, Shinkov A, Kovatcheva R, Vlahov J, Dakovska L, Todorov T. Changes in the prevalence of diabetes mellitus in bulgaria (2006-2012). Clin Med Insights Endocrinol Diabetes. 2015; 8: 41-5.	Total diabetes
Boronat M, Saavedra P, Perez-Martin N, Lopez-Madrazo MJ, Rodriguez-Perez C, Novoa FJ. High levels of lipoprotein(a) are associated with a lower prevalence of diabetes with advancing age: results of a cross-sectional epidemiological survey in Gran Canaria, Spain. Cardiovasc Diabetol. 2012; 11: 81.	Total diabetes
Bratina NU, Tahirovic H, Battelino T, Krzisnik C. Incidence of childhood-onset Type I diabetes in Slovenia and the Tuzia region (Bosnia and Herzegovina) in the period 1990-1998. Diabetologia. 2001; B27-31.	Total diabetes, type 1 diabetes
Bruno G, Maule M, Biggeri A, Ledda A, Mannu C, Merletti F, Songini M, Sardinian Group for Diabetes Epidemiology. More than 20 years of registration of type 1 diabetes in Sardinian children: temporal variations of incidence with age, period of diagnosis, and year of birth. Diabetes. 2013; 62(10): 3542-6.	Total diabetes, type 1 diabetes
Bruno G, Maule M, Merletti F, Novelli G, Falorni A, Iannilli A, Iughetti L, Altobelli E, d'Annunzio G, Piffer S, Pozzilli P, Iafusco D, Songini M, Roncarolo F, Toni S, Carle F, Cherubini V. Age-period-cohort analysis of 1990-2003 incidence time trends of childhood diabetes in Italy: the RIDI study. Diabetes. 2010; 59(9): 2281-7.	Total diabetes, type 1 diabetes
Bruno G, Merletti F, Biggeri A, Cerutti F, Grosso N, De Salvia A, Vitali E, Pagano G. Increasing trend of type I diabetes in children and young adults in the province of Turin (Italy). Analysis of age, period and birth cohort effects from 1984 to 1996. Diabetologia. 2001; 44(1): 22-5.	Total diabetes, type 1 diabetes
Bruno G, Merletti F, De Salvia A, Lezo A, Arcari R, Pagano G. Comparison of incidence of insulin-dependent diabetes mellitus in children and young adults in the Province of Turin, Italy, 1984-91. Piedmont Study Group for Diabetes Epidemiology. Diabet Med. 1997; 14(11): 964-9.	Total diabetes, type 1 diabetes
Bruno G, Novelli G, Panero F, Perotto M, Monasterolo F, Bona G, Perino A, Rabbone I, Cavallo-Perin P, Cerutti F, Piedmont Study Group for Diabetes Epidemiology. The incidence of type 1 diabetes is increasing in both children and young adults in Northern Italy: 1984-2004 temporal trends. Diabetologia. 2009; 52(12): 2531-5.	Total diabetes, type 1 diabetes
Bruno G, Runzo C, Cavallo-Perin P, Merletti F, Rivetti M, Pinach S, Novelli G, Trovati M, Cerutti F, Pagano G, Piedmont Study Group for Diabetes Epidemiology. Incidence of type 1 and type 2 diabetes in adults aged 30-49 years: the population-based registry in the province of Turin, Italy. Diabetes Care. 2005; 28(11): 2613-9.	Total diabetes, type 1 diabetes
Bureau of Health Promotion, Department of Health (Taiwan), Department of Health (Taiwan), Population Studies Center, University of Michigan, Union Clinical Laboratory (UCL) (Taiwan), iSTAT Healthcare Consulting (Taiwan). Taiwan Social Environment and Biomarkers of Aging Study (SEBAS) 2006-2007.	Total diabetes
Burke JP, Williams K, Haffner SM, Villalpando CG, Stern MP. Elevated incidence of type 2 diabetes in San Antonio, Texas, compared with that of Mexico City, Mexico. Diabetes Care. 2001; 24(9): 1573-8.	Total diabetes
Bustos P, da Silva AAM, Amigo H, Bettiol H, Barbieri MA. Metabolic syndrome in young adults from two socioeconomic Latin American settings. Nutr Metab Cardiovasc Dis. 2007; 17(8): 581-9.	Total diabetes
Cabrera de León A, Rodríguez Pérez Mdel C, Almeida González D, Domínguez Coello S, Aguirre Jaime A, Brito Díaz B, González Hernández A, Pérez Méndez LI, grupo CDC. Presentación de la cohorte "CDC de Canarias": objetivos, diseño y resultados preliminares. Rev Esp Salud Publica. 2008; 82(5): 519-534.	Total diabetes
Cai L, Liu A, Zhang L, Li S, Wang P. Prevalence, awareness, treatment, and control of hypertension among adults in Beijing, China. Clin Exp Hypertens. 2012; 34(1): 45–52.	Total diabetes
Calori G, Gallus G, Garancini P, Repetto F, Micossi P. Identification of the cohort of type 1 diabetes presenting in Lombardy in 1983-84: a validated assessment. Diabet Med. 1990; 7(7): 595-9.	Total diabetes, type 1 diabetes
Campos H, Mata L, Siles X, Vives M, Ordovas JM, Schaefer EJ. Prevalence of cardiovascular risk factors in rural and urban Costa Rica. Circulation. 1992; 85(2): 648-58.	Total diabetes

Cardwell CR, Carson DJ, Patterson CC. Secular trends, disease maps and ecological analyses of the incidence of childhood onset Type 1 diabetes in Northern Ireland, 1989-2003. Diabet Med. 2007; 24(3): 289-95.	Total diabetes, type 1 diabetes
Caribbean Epidemiology Centre (CAREC), Central Statistical Office (Trinidad and Tobago), Ministry of Health (Trinidad and Tobago), Pan American Health Organization (PAHO), University of the West Indies. Trinidad and Tobago STEPS Noncommunicable Disease Risk Factors Survey 2011.	Total diabetes
Caribbean Epidemiology Centre (CAREC), Ministry of Health and Social Development (Virgin Islands, British), Pan American Health Organization (PAHO), World Health Organization (WHO). Virgin Islands, British STEPS Noncommunicable Disease Risk Factors Survey 2009.	Total diabetes
Carolina Population Center, University of North Carolina at Chapel Hill, Chinese Center for Disease Control and Prevention (CCDC). China Health and Nutrition Survey. Chapel Hill, United States of America: Carolina Population Center, University of North Carolina at Chapel Hill.	Total diabetes
Carrasco E, P‚rez-Bravo F, Dorman J, Mondrag¢n A, Santos JL. Increasing incidence of type 1 diabetes in population from Santiago of Chile: trends in a period of 18 years (1986-2003). Diabetes Metab Res Rev. 2006; 22(1): 34-7.	Total diabetes, type 1 diabetes
Carrasco E, Perez-Bravo F, Santos JL, Lopez G, Calvillan M, Wolff C, Garcia de los Rios M. One of the lowest validated incidence rates of insulin dependent diabetes mellitus in the Americas: Santiago, Chile. Diabetes Res Clin Pract. 1996; S153-7.	Total diabetes, type 1 diabetes
Casiglia E, Tikhonoff V, Mazza A, Piccoli A, Pessina AC. Pulse pressure and coronary mortality in elderly men and women from general population. J Hum Hypertens. 2002; 16(9): 611-20.	Total diabetes
Casu A, Pascutto C, Bernardinelli L, Songini M. Type 1 diabetes among sardinian children is increasing: the Sardinian diabetes register for children aged 0-14 years (1989-1999). Diabetes Care. 2004; 27(7): 1623-9.	Total diabetes, type 1 diabetes
Center for Research and Teaching in Economics (CIDE) (Mexico), Duke University, Ibero-American University, National Institute of Public Health (Mexico), University of California, Los Angeles (UCLA). Mexico Family Life Survey 2008-2013.	Total diabetes
Center for Strategic Studies, University of Jordan, Ministry of Health (Jordan), World Health Organization (WHO). Jordan STEPS Noncommunicable Disease Risk Factors Survey 2019. WHO NCD Microdata Repository, 2021.	Total diabetes
Centers for Disease Control and Prevention (CDC), Institute of Nutrition of Central America and Panama, Pan American Health Organization (PAHO). El Salvador - Santa Tecla Diabetes, Hypertension, and Chronic Disease Risk Factors Survey 2004.	Total diabetes
Centers for Disease Control and Prevention (CDC), Institute of Nutrition of Central America and Panama, Pan American Health Organization (PAHO). Guatemala - Villa Nueva Diabetes, Hypertension, and Chronic Disease Risk Factors Survey 2002-2003.	Total diabetes
Centers for Disease Control and Prevention (CDC), Ministry of Health (Jordan), World Health Organization (WHO). Jordan STEPS Noncommunicable Disease Risk Factors Survey 2007.	Total diabetes
Centers for Disease Control and Prevention (CDC), Ministry of Health (Rwanda), National Institute of Statistics of Rwanda, Rwanda Biomedical Center (RBC), World Health Organization (WHO). Rwanda STEPS Noncommunicable Disease Risk Factor Survey 2012-2013.	Total diabetes
Central Organization for Statistics and Information Technology (Iraq), Ministry of Health (Iraq), World Health Organization (WHO). Iraq STEPS Noncommunicable Disease Risk Factors Survey 2006.	Total diabetes
Centre for Chronic Disease Control (India), Indian Council of Medical Research (ICMR). India Diet and Lifestyle Interventions for Hypertension Risk reduction through Anganwandi Workers and Accredited Social Health Activists Baseline Study 2013-2014.	Total diabetes
Centre for Chronic Disease Control (India). India Prevalence of Coronary Heart Disease and its Risk Factors in Residents of Urban and Rural Areas of NCR Survey 2010-2012.	Total diabetes
Centre for Physical Activity and Health, University of Sydney (Australia), Department of Health and Social Affairs (Micronesia), Fiji School of Medicine, Micronesia Human Resources Development Center, Pohnpei State Department of Health Services, World Health Organization (WHO). Micronesia - Pohnpei STEPS Noncommunicable Disease Risk Factors Survey 2002.	Total diabetes
Centre for Physical Activity and Health, University of Sydney (Australia), Ministry of Health (Nauru), World Health Organization (WHO). Nauru STEPS Noncommunicable Disease Risk Factors Survey 2004.	Total diabetes
Chahkandi T, Taheri F, Kazemi T, Bijari B. The Prevalence of Diabetes and Prediabetes Among Elementary School Children in Birjand. Iran J Pediatr. 2015; 25(1): e183.	Total diabetes
Chen HD, Shaw CK, Tseng WP, Chen HI, Lee ML. Prevalence of diabetes mellitus and impaired glucose tolerance in Aborigines and Chinese in eastern Taiwan. Diabetes Res Clin Pract. 1997; 38(3): 199-205.	Total diabetes
Cherubini V, Carle F, Gesuita R, Iannilli A, Tuomilehto J, Prisco F, Iafusco D, Altobelli E, Chiarelli F, De Giorgi G, Falorni A. Large incidence variation of Type I diabetes in central-southern Italy 1990-1995: lower risk in rural areas. Diabetologia. 1999; 42(7): 789-92.	Total diabetes, type 1 diabetes
Chhetri JK, Zheng Z, Xu X, Ma C, Chan P. The prevalence and incidence of frailty in Pre-diabetic and diabetic community-dwelling older population: results from Beijing longitudinal study of aging II (BLSA-II). BMC Geriatr. 2017; 17(1): 47.	Total diabetes
Chhetri MR, Chapman RS. Prevalence and determinants of diabetes among the elderly population in the Kathmandu Valley of Nepal. Nepal Med Coll J. 2009; 11(1): 34-8.	Total diabetes
Chiang P-H, Liu C-L, Lin M-H, Peng L-N, Chen L-K, Chen J-D, Hou S-M. Survival benefits of metabolic syndrome among older men aged 75 years and over in Taiwan. J Nutr Health Aging. 2012; 16(6): 520–4.	Total diabetes
Chin CY, Pengal S. Cardiovascular disease risk in a semirural community in Malaysia. Asia Pac J Public Health. 2009; 21(4): 410-20.	Total diabetes
Chinese Center for Disease Control and Prevention (CCDC). China Chronic Disease and Risk Factor Surveillance 2010.	Total diabetes
Chinese Center for Disease Control and Prevention (CCDC). China Chronic Disease and Risk Factor Surveillance 2013.	Total diabetes
Chong JW, Craig ME, Cameron FJ, Clarke CF, Rodda CP, Donath SM, Werther GA. Marked increase in type 1 diabetes mellitus incidence in children aged 0-14 yr in Victoria, Australia, from 1999 to 2002. Pediatr Diabetes. 2007; 8(2): 67-73.	Total diabetes, type 1 diabetes
Chou P, Liao MJ, Kuo HS, Hsiao KJ, Tsai ST. A population survey on the prevalence of diabetes in Kin-Hu, Kinmen. Diabetes Care. 1994; 17(9): 1055-8.	Total diabetes
Christian Medical College and Hospital Ludhiana (India), Department of Health and Family Welfare, Government of Punjab (India), Government Medical College, Amritsar (India), Government Medical College, Patiala (India), Guru Gobind Singh Medical College, Faridkot (India), Postgraduate Institute of Medical Education & Research (PGIMER) (Chandigarh), University of Michigan. India - Punjab Noncommunicable Disease Risk Factors Survey 2014-2015.	Total diabetes
Christian Medical College, Vellore (India), MRC Epidemiology Resource Center, University of Southampton. India - Vellore Birth Cohort Study 1998-2002.	Total diabetes
Chronic Disease Research Centre, University of the West Indies, Ministry of Health (Barbados). Barbados Health of the Nation Survey 2011.	Total diabetes
Chueca M, Oyarzabal M, Reparaz F, Garagorri JM, Sola A. Incidence of type I diabetes mellitus in Navarre, Spain (1975-91). Acta Paediatr. 1997; 86(6): 632-7.	Total diabetes, type 1 diabetes

Chuuk Department of Health Services (Micronesia), Department of Health and Social Affairs (Micronesia), World Health Organization (WHO). Micronesia - Chuuk STEPS Noncommunicable Disease Risk Factors Survey 2006.	Total diabetes
Chuuk Department of Health Services (Micronesia), Department of Health and Social Affairs (Micronesia), World Health Organization (WHO). Micronesia - Chuuk STEPS Noncommunicable Disease Risk Factors Survey 2016. Geneva, Switzerland: World Health Organization (WHO), 2019.	Total diabetes
Cinek O, Sumnik Z, Vavrinec J. Continuing increase in incidence of childhood-onset type 1 diabetes in the Czech Republic 1990-2001. Eur J Pediatr. 2003; 162(6): 428-9.	Total diabetes, type 1 diabetes
Cockram CS, Woo J, Lau E, Chan JC, Chan AY, Lau J, Swaminathan R, Donnan SP. The prevalence of diabetes mellitus and impaired glucose tolerance among Hong Kong Chinese adults of working age. Diabetes Res Clin Pract. 1993; 21(1): 67-73.	Total diabetes
College of Family Physicians in Poland (CFPiP), Częstochowa University of Technology, Polish Lipid Association, Silesian Analytical Laboratories. Poland LIPIDOGRAM 2015-2016 Study.	Total diabetes
College of Medicine, Nursing and Health Sciences, Fiji National University, Ministry of Health (Cook Islands), World Health Organization (WHO). Cook Islands STEPS Noncommunicable Disease Risk Factors Survey 2003-2004.	Total diabetes
Collins VR, Dowse GK, Toelupe PM, Imo TT, Aloaina FL, Spark RA, Zimmet PZ. Increasing prevalence of NIDDM in the Pacific island population of Western Samoa over a 13-year period. Diabetes Care. 1994; 17(4): 288-96.	Total diabetes
Cook-Huynh M, Ansong D, Steckelberg RC, Boakye I, Seligman K, Appiah L, Kumar N, Amuasi JH. Prevalence of hypertension and diabetes mellitus in adults from a rural community in Ghana. Ethn Dis. 2012; 22(3): 347-52.	Total diabetes
Cortez-Dias N, Martins S, Belo A, Fiuza M. Prevalence, management and control of diabetes mellitus and associated risk factors in primary health care in Portugal. Rev Port Cardiol. 2010; 29(4): 509-37.	Total diabetes
Cotellessa M, Barbieri P, Mazzella M, Bonassi S, Minicucci L, Lorini R. High incidence of childhood type 1 diabetes in Liguria, Italy, from 1989 to 1998. Diabetes Care. 2003; 26(6): 1786-9.	Total diabetes, type 1 diabetes
Craig ME, Femia G, Broyda V, Lloyd M, Howard NJ. Type 2 diabetes in Indigenous and non-Indigenous children and adolescents in New South Wales. Med J Aust. 2007; 186(10): 497-9.	Type 1 diabetes
Craig ME, Howard NJ, Silink M, Chan A. The rising incidence of childhood type 1 diabetes in New South Wales, Australia. J Pediatr Endocrinol Metab. 2000; 13(4): 363-72.	Total diabetes, type 1 diabetes
Croxson SC, Burden AC, Bodington M, Botha JL. The prevalence of diabetes in elderly people. Diabet Med. 1991; 8(1): 28-31.	Total diabetes
Cuong TQ, Dibley MJ, Bowe S, Hanh TT, Loan TT. Obesity in adults: an emerging problem in urban areas of Ho Chi Minh City, Vietnam. Eur J Clin Nutr. 2007; 61(5): 673-81.	Total diabetes
Dahiru T, Jibo A, Hassan AA, Mande AT. Prevalence of diabetes in a semi-urban community in Northern Nigeria. Niger J Med. 2008; 17(4): 414-6.	Total diabetes
Dahlquist GG, Nystrom L, Patterson CC. Incidence of type 1 diabetes in Sweden among individuals aged 0-34 years, 1983-2007: an analysis of time trends. Diabetes Care. 2011; 34(8): 1754-9.	Total diabetes, type 1 diabetes
Daniel CR, Prabhakaran D, Kapur K, Graubard BI, Devasenapathy N, Ramakrishnan L, George PS, Shetty H, Ferrucci LM, Yurgalevitch S, Chatterjee N, Reddy KS, Rastogi T, Gupta PC, Mathew A, Sinha R. A cross-sectional investigation of regional patterns of diet and cardio-metabolic risk in India. Open Nutr J. 2011; 12.	Total diabetes
Davies TT, Graue M, Igland J, Tell GS, Birkeland KI, Peyrot M, Haltbakk J. Diabetes prevalence among older people receiving care at home: associations with symptoms, health status and psychological well-being. Diabet Med. 2019; 36(1): 96-104.	Total diabetes
De Beaufort CE, Cecchi-Tenerini R, Clerc R, Hilaire M, Schwartz F, Manciaux M. [Incidence of insulin-dependent diabetes in six to sixteen year old school children in Lorraine]. Arch Fr Pediatr. 1991; 48(3): 228.	Total diabetes
De Pablos-Velasco PL, Martínez-Martín FJ, Rodríguez-Pérez F, Anía BJ, Losada A, Betancor P. Prevalence and determinants of diabetes mellitus and glucose intolerance in a Canarian Caucasian population - comparison of the 1997 ADA and the 1985 WHO criteria. The Guía Study. Diabet Med. 2001; 18(3): 235-41.	Total diabetes
De Sereday MS, Gonzalez C, Giorgini D, De Loredo L, Braguinsky J, Cobeñas C, Libman C, Tesone C. Prevalence of diabetes, obesity, hypertension and hyperlipidemia in the central area of Argentina. Diabetes Metab. 2004; 30(4): 335-9.	Total diabetes
de Souza LJ, Chalita FE, Reis AF, Teixeira CL, Gicovate Neto C, Bastos DA, De Souza TF, Côrtes VA, D. S. Filho JT. Prevalência de diabetes mellitus e fatores de risco em Campos dos Goytacazes, RJ. Arq Bras Endocrinol Metabol. 2003; 47(1): 69-74 .	Total diabetes
Dedov II, Shestakova MV, Vikulova OK. Epidemiology of Diabetes Mellitus in Russian Federation: Clinical and Statistical Report According to the Federal Diabetes Registry. Diabetes Mellit. 2017; 20(1): 13–41.	Type 1 diabetes
Deedwania PC, Gupta R, Sharma KK, Achari V, Gupta B, Maheshwari A, Gupta A. High prevalence of metabolic syndrome among urban subjects in India: a multisite study. Diabetes Metab Syndr. 2014; 8(3): 156–61.	Total diabetes
Defay R, Delcourt C, Ranvier M, Lacroux A, Papoz L. Relationships between physical activity, obesity and diabetes mellitus in a French elderly population: the POLA study. Pathologies Oculaires lieés á l' Age. Int J Obes Relat Metab Disord. 2001; 25(4): 512-8.	Total diabetes
Department of Census and Statistics (Sri Lanka), Ministry of Health (Sri Lanka), World Health Organization (WHO), World Health Organization Regional Office for South-East Asia (SEARO). Sri Lanka STEPS Noncommunicable Disease Risk Factors Survey 2014-2015.	Total diabetes
Department of Health (American Samoa), Monash University (Australia), World Health Organization (WHO). American Samoa STEPS Noncommunicable Disease Risk Factors Survey 2004.	Total diabetes
Derakhshan A, Sardarinia M, Khalili D, Momenan AA, Azizi F, Hadaegh F. Sex specific incidence rates of type 2 diabetes and its risk factors over 9 years of follow-up: Tehran Lipid and Glucose Study. PLoS One. 2014; 9(7): e102563.	Total diabetes
Derraik JGB, Reed PW, Jefferies C, Cutfield SW, Hofman PL, Cutfield WS. Increasing incidence and age at diagnosis among children with type 1 diabetes mellitus over a 20-year period in Auckland (New Zealand). PLoS One. 2012; 7(2): e32640.	Total diabetes, type 1 diabetes
Digestive Diseases Research Institute (DDRI) (Iran), Shiraz University of Medical Sciences. Iran - Pars Cohort Study.	Total diabetes
Dionadji M, Boy B, Mouanodji M, Batakao G. Prevalence of diabetes mellitus in rural areas in Chad. Med Trop (Mars). 2010; 70(4): 414-5.	Total diabetes
Directorate General of Health-Duhok (Iraq), Kurdistan Regional Government (Iraq), Ministry of Health (Iraq), World Health Organization (WHO). Iraq - Dahūk STEPS Noncommunicable Disease Risk Factors Survey 2003-2004.	Total diabetes
Divison Garrote JA, Masso Orozco J, Carrion Valero L, Lopez Abril J, Carbayo Herencia JA, Artigao Rodenas LM, Gil Guillen V, Grupo de Enfermedades Vasculares de Albacete (GEVA). [Trends in prevalence of risk factors and global cardiovascular risk in general population of albacete, Spain (1992-94 a 2004-06)]. Rev Esp Salud Publica. 2011; 85(3): 275–84.	Total diabetes
Dogan N, Toprak D, Demir S. Hypertension prevalence and risk factors among adult population in Afyonkarahisar region: a cross-sectional research. Anadolu Kardiyol Derg. 2012; 12(1): 47–52.	Total diabetes

Dong Y, Gao W, Nan H, Yu H, Li F, Duan W, Wang Y, Sun B, Qian R, Tuomilehto J, Qiao Q. Prevalence of Type 2 diabetes in urban and rural Chinese populations in Qingdao, China. Diabet Med. 2005; 22(10): 1427-33.	Total diabetes
Dorynska A, Polak M, Kozela M, Szafraniec K, Piotrowski W, Bielecki W, Drygas W, Kozakiewicz K, Piwonski J, Tykarski A, Zdrojewski T, Pajak A. Cardiovascular disease (CVD) risk factors in Krakow and in the whole Poland adult population. Results from the WOBASZ study and Polish arm of the HAPIEE project. Przegl Epidemiol. 2015; 69(1): 79–86.	Total diabetes
Dowse GK, Gareeboo H, Zimmet PZ, Alberti KG, Tuomilehto J, Fareed D, Brissonnette LG, Finch CF. High prevalence of NIDDM and impaired glucose tolerance in Indian, Creole, and Chinese Mauritians. Mauritius Noncommunicable Disease Study Group. Diabetes. 1990; 39(3): 390-6.	Total diabetes
Drivsholm T, Ibsen H, Schroll M, Davidsen M, Borch-Johnsen K. Increasing prevalence of diabetes mellitus and impaired glucose tolerance among 60-year-old Danes. Diabet Med. 2001; 18(2): 126-32.	Total diabetes
Droumaguet C, Balkau B, Simon D, Caces E, Tichet J, Charles MA, Eschwege E, DESIR Study Group. Use of HbA1c in predicting progression to diabetes in French men and women: data from an Epidemiological Study on the Insulin Resistance Syndrome (DESIR). Diabetes Care. 2006; 29(7): 1619–25.	Total diabetes
Duboz P, Chapuis-Lucciani N, Boëtsch G, Gueye L. Prevalence of diabetes and associated risk factors in a Senegalese urban (Dakar) population. Diabetes Metab. 2012; 38(4): 332-6.	Total diabetes
Dziatkowiak H, Ciechanowska M, Wasikowa R, Symonides-ławecka A, Bieniasz J, Trippenbach-Dulska H, Korniszewski L, Szybiński Z. Increase in the incidence of type 1 diabetes mellitus in children in three cities in Poland, 1987-1999. J Pediatr Endocrinol Metab. 2002; 15(8): 1153-60.	Total diabetes, type 1 diabetes
Ebrahimi H, Emamian MH, Hashemi H, Fotouhi A. High Incidence of Diabetes Mellitus Among a Middle-Aged Population in Iran: A Longitudinal Study. Can J Diabetes. 2016; 40(6): 570–5.	Total diabetes
Echouffo-Tcheugui JB, Dzudie A, Epacka ME, Choukem SP, Doualla MS, Luma H, Kengne AP. Prevalence and determinants of undiagnosed diabetes in an urban sub-Saharan African population. Prim Care Diabetes. 2012; 6(3): 229–34.	Total diabetes
Eggertsen R, Lapidus L, Lindstedt G, Nilsson T, Nyström E. [A study of 56-65 years old persons in Mölnlycke. No association between Helicobacter and heart disease or thyroid disorder]. Lakartidningen. 2002; 99(6): 508-9.	Total diabetes
Ehehalt S, Blumenstock G, Willasch AM, Hub R, Ranke MB, Neu A. Continuous rise in incidence of childhood Type 1 diabetes in Germany. Diabet Med. 2008; 25(6): 755-7.	Total diabetes, type 1 diabetes
Ejim EC, Onwubere BJ, Okafor CI, Ulasi II, Emehel A, Onyia U, Akabueze J, Mendis S. Cardiovascular risk factors in middle-aged and elderly residents in South-East Nigeria: the influence of urbanization. Niger J Med. 2013; 22(4): 286–91.	Total diabetes
Elbagir MN, Eltom MA, Elmahadi EM, Kadam IM, Berne C. A high prevalence of diabetes mellitus and impaired glucose tolerance in the Danagla community in northern Sudan. Diabet Med. 1998; 15(2): 164-9.	Total diabetes
Elbagir MN, Eltom MA, Elmahadi EM, Kadam IM, Berne C. A population-based study of the prevalence of diabetes and impaired glucose tolerance in adults in northern Sudan. Diabetes Care. 1996; 19(10): 1126-8.	Total diabetes
El-Hazmi MA, Al-Swailem A, Warsy AS, Al-Sudairy F, Sulaimani R, Al-Swailem A, Al-Meshari A. The prevalence of diabetes mellitus and impaired glucose tolerance in the population of Riyadh. Ann Saudi Med. 1995; 15(6): 598-601.	Type 1 diabetes
El-Hazmi MA, Warsy AS, Al-Swailem AR, Al-Swailem AM, Sulaimani R, Al-Meshari AA. Diabetes mellitus and impaired glucose tolerance in Saudi Arabia. Ann Saudi Med. 1996; 16(4): 381-5.	Total diabetes, type 1 diabetes
El-Ziny MA, Salem NA, El-Hawary AK, Chalaby NM, Elsharkawy AA. Epidemiology of childhood type 1 diabetes mellitus in Nile Delta, northern Egypt - a retrospective study. J Clin Res Pediatr Endocrinol. 2014; 6(1): 9-15.	Total diabetes, type 1 diabetes
Erem C, Yildiz R, Kavgaci H, Karahan C, Deger O, Can G, Telatar M. Prevalence of diabetes, obesity and hypertension in a Turkish population (Trabzon city). Diabetes Res Clin Pract. 2001; 54(3): 203-8.	Total diabetes
Espelt A, Goday A, Franch J, Borrell C. Validity of self-reported diabetes in health interview surveys for measuring social inequalities in the prevalence of diabetes. J Epidemiol Community Health. 2012; 66(7): e15.	Total diabetes
Ethiopian Public Health Institute (EPHI), World Health Organization (WHO). Ethiopia STEPS Noncommunicable Disease Risk Factors Survey 2015.	Total diabetes
Evaristo-Neto AD, Foss-Freitas MC, Foss MC. Prevalence of diabetes mellitus and impaired glucose tolerance in a rural community of Angola. Diabetol Metab Syndr. 2010; 63.	Total diabetes
Evertsen J, Alemzadeh R, Wang X. Increasing incidence of pediatric type 1 diabetes mellitus in Southeastern Wisconsin: relationship with body weight at diagnosis. PLoS One. 2009; 4(9): e6873.	Total diabetes, type 1 diabetes
Félix-Redondo FJ, Baena-Díez JM, Grau M, Tormo MÁ, Fernández-Bergés D. Prevalence of obesity and cardiovascular risk in the general population of a health area in Extremadura (Spain): the Hermex study. Endocrinol Nutr. 2012; 59(3): 160–8.	Total diabetes
Faculty of Public Health, Medical University of Varna (Bulgaria). Bulgaria - Dolni Chiflik Blood Pressure Study 2007.	Total diabetes
Fava C, Sjogren M, Montagnana M, Danese E, Almgren P, Engstrom G, Nilsson P, Hedblad B, Guidi GC, Minuz P, Melander O. Prediction of blood pressure changes over time and incidence of hypertension by a genetic risk score in Swedes. Hypertension. 2013; 61(2): 319–26.	Total diabetes
Fawwad A, Alvi SFD, Basit A, Ahmed K, Ahmedani MY, Hakeem R. Changing pattern in the risk factors for diabetes in young adults from the rural area of Baluchistan. J Pak Med Assoc. 2013; 63(9): 1089–93.	Total diabetes
Federal Ministry of Health (Sudan), World Health Organization (WHO). Sudan - Khartoum STEPS Noncommunicable Disease Risk Factors Survey 2005-2006.	Total diabetes
Federal Ministry of Health (Sudan), World Health Organization (WHO). Sudan STEPS Noncommunicable Disease Risk Factors Survey 2016. Geneva, Switzerland: World Health Organization (WHO), 2019.	Total diabetes
Ferguson TS, Younger NOM, Tulloch-Reid MK, Wright MBL, Ward EM, Ashley DE, Wilks RJ. Prevalence of prehypertension and its relationship to risk factors for cardiovascular disease in Jamaica: analysis from a cross-sectional survey. BMC Cardiovasc Disord. 2008; 8: 20.	Total diabetes
Fernandez-Ramos C, Arana-Arri E, Jimenez-Huertas P, Vela A, Rica I. Incidence of childhood-onset type 1 diabetes in Biscay, Spain, 1990-2013. Pediatr Diabetes. 2017; 18(1): 71–6.	Total diabetes, type 1 diabetes
Fernando DJ, Siribaddana S, de Silva D. Impaired glucose tolerance and diabetes mellitus in a suburban Sri Lankan community. Postgrad Med J. 1994; 70(823): 347-9.	Total diabetes
Ferrer A, Padrós G, Formiga F, Rojas-Farreras S, Perez JM, Pujol R. Diabetes mellitus: prevalence and effect of morbidities in the oldest old. The Octabaix study. J Am Geriatr Soc. 2012; 60(3): 462-7.	Total diabetes
Fiji School of Medicine, Menzies Center for Population Health Research, University of Tasmania (Australia), Ministry of Health (Marshall Islands), World Health Organization (WHO). Marshall Islands STEPS Noncommunicable Disease Risk Factors Survey 2002.	Total diabetes
Fiji School of Medicine, Ministry of Health and Medical Services (Solomon Islands), World Health Organization (WHO). Solomon Islands STEPS Noncommunicable Disease Risk Factors Survey 2005-2006.	Total diabetes

Florez H, Silva E, Fernández V, Ryder E, Sulbarán T, Campos G, Calmón G, Clavel E, Castillo-Florez S, Goldberg R. Prevalence and risk factors associated with the metabolic syndrome and dyslipidemia in White, Black, Amerindian and Mixed Hispanics in Zulia State, Venezuela. Diabetes Res Clin Pract. 2005; 69(1): 63-77.	Total diabetes
Food and Nutrition Research Institute, Department of Science and Technology (Philippines). Philippines National Nutrition Survey 2013-2014.	Total diabetes
Forga Llenas L, Goni Iriarte MJ, Cambra Contin K, Ibanez Beroiz B, Chueca Guendulain M, Berrade Zubiri S. Incidence and temporal trends of childhood type 1 diabetes between 1975 and 2012 in Navarre (Spain). Gac Sanit. 2015; 29(1): 51-4.	Total diabetes, type 1 diabetes
Formosa N, Calleja N, Torpiano J. Incidence and modes of presentation of childhood type 1 diabetes mellitus in Malta between 2006 and 2010. Pediatr Diabetes. 2012; 13(6): 484-8.	Total diabetes, type 1 diabetes
Fortunato F, Cappelli MG, Vece MM, Caputi G, Delvecchio M, Prato R, Martinelli D, Apulian Childhood-Onset Diabetes Registry Workgroup. Incidence of Type 1 Diabetes among Children and Adolescents in Italy between 2009 and 2013: The Role of a Regional Childhood Diabetes Registry. J Diabetes Res. 2016; 2016: 7239692.	Total diabetes, type 1 diabetes
Foster C, Rotimi C, Fraser H, Sundarum C, Liao Y, Gibson E, Holder Y, Hoyos M, Mellanson-King R. Hypertension, diabetes, and obesity in Barbados: findings from a recent population-based survey. Ethn Dis. 1993; 3(4): 404-12.	Total diabetes
Freitas MPD, Loyola Filho AI de, Lima-Costa MF. Birth cohort differences in cardiovascular risk factors in a Brazilian population of older elderly: the Bambuí Cohort Study of Aging (1997 and 2008). Cad Saude Publica. 2011; S409-417.	Total diabetes
French Institute for Public Health Surveillance (INVS), French National Health Insurance Fund for Salaried Workers (CNAMTS), National Conservatory of Arts and Trades (CNAM) (France), University of Paris 13. France National Nutrition and Health Survey 2006-2007 - Public Health France.	Total diabetes
French Institute of Health and Medical Research (INSERM), University of Victor Segalen Bordeaux (France). France Three-City Cohort Study 1999-2001.	Total diabetes
Gamlath L, Nandasena S, Hennadige Padmal de Silva S, Linhart C, Ngo A, Morrell S, Nathan S, Sharpe A, Taylor R. Differentials in Cardiovascular Risk Factors and Diabetes by Socioeconomic Status and Sex in Kalutara, Sri Lanka. Asia Pac J Public Health. 2017; 29(5): 401-410.	Total diabetes
Gao WG, Dong YH, Pang ZC, Nan HR, Zhang L, Wang SJ, Ren J, Ning F, Qiao Q. Increasing trend in the prevalence of Type 2 diabetes and pre-diabetes in the Chinese rural and urban population in Qingdao, China. Diabet Med. 2009; 26(12): 1220-7.	Total diabetes
Garancini MP, Calori G, Manara E, Izzo A, Ebbli E, Galli L, Boari L, Gallus G. An Italian population-based study of the prevalence of diabetes: some methodological aspects. Diabete Metab. 1993; 19(1 Pt 2): 116-20.	Total diabetes
Gardete-Correia L, Boavida JM, Raposo JF, Mesquita AC, Fona C, Carvalho R, Massano-Cardoso S. First diabetes prevalence study in Portugal: PREVADIAB study. Diabet Med. 2010; 27(8): 879-81.	Total diabetes
Gardner SG, Bingley PJ, Sawtell PA, Weeks S, Gale EA. Rising incidence of insulin dependent diabetes in children aged under 5 years in the Oxford region: time trend analysis. The Bart's-Oxford Study Group. BMJ. 1997; 315(7110): 713-7.	Total diabetes, type 1 diabetes
Gatling W, Budd S, Walters D, Mullee MA, Goddard JR, Hill RD. Evidence of an increasing prevalence of diagnosed diabetes mellitus in the Poole area from 1983 to 1996. Diabet Med. 1998; 15(12): 1015-21.	Type 1 diabetes
Gause-Nilsson I, Gherman S, Kumar Dey D, Kennerfalk A, Steen B. Prevalence of metabolic syndrome in an elderly Swedish population. Acta Diabetol. 2006; 43(4): 120-6.	Total diabetes
Gelaye B, Revilla L, Lopez T, Sanchez S, Williams MA. Prevalence of metabolic syndrome and its relationship with leisure time physical activity among Peruvian adults. Eur J Clin Invest. 2009; 39(10): 891–8.	Total diabetes
George Institute for Global Health (Australia), International Council for the Control of Iodine Deficiency Disorders (ICCIDD), Ministry of Health (Samoa), Samoa Bureau of Statistics, World Health Organization (WHO). Samoa STEPS Noncommunicable Disease Risk Factors Survey 2013.	Total diabetes
Ghana Health Service, World Health Organization (WHO). Ghana - Greater Accra Region STEPS Noncommunicable Disease Risk Factors Survey 2006.	Total diabetes
Giri BR, Sharma KP, Chapagai RN, Palzom D. Diabetes and hypertension in urban bhutanese men and women. Indian J Community Med. 2013; 38(3): 138-43.	Total diabetes
Goday A, Castell C, Tresserras R, Canela J, Taberner JL, Lloveras G. Incidence of type 1 (insulin-dependent) diabetes mellitus in Catalonia, Spain. The Catalan Epidemiology Diabetes Study Group. Diabetologia. 1992; 35(3): 267-71.	Total diabetes, type 1 diabetes
Gokcel A, Ozsahin AK, Sezgin N, Karakose H, Ertorer ME, Akbaba M, Baklaci N, Sengul A, Guvener N. High prevalence of diabetes in Adana, a southern province of Turkey. Diabetes Care. 2003; 26(11): 3031-4.	Total diabetes
Gomez-Huelgas R, Mancera-Romero J, Bernal-Lopez MR, Jansen-Chaparro S, Baca-Osorio AJ, Toledo E, Perez-Gonzalez R, Guijarro-Merino R, Tinahones FJ, Martinez-Gonzalez MA. Prevalence of cardiovascular risk factors in an urban adult population from southern Spain. IMAP Study. Int J Clin Pract. 2011; 65(1): 35–40.	Total diabetes
Gong C, Meng X, Jiang Y, Wang X, Cui H, Chen X. Trends in childhood type 1 diabetes mellitus incidence in Beijing from 1995 to 2010: a retrospective multicenter study based on hospitalization data. Diabetes Technol Ther. 2015; 17(3): 159-65.	Total diabetes, type 1 diabetes
González-Villalpando C, Dávila-Cervantes CA, Zamora-Macorra M, Trejo-Valdivia B, González-Villalpando ME. Incidence of type 2 diabetes in Mexico: results of the Mexico City Diabetes Study after 18 years of follow-up. Salud Publica Mex. 2014; 56(1): 11-7.	Total diabetes
Gopinath S, Ortqvist E, Norgren S, Green A, Sanjeevi CB. Variations in incidence of type 1 diabetes in different municipalities of stockholm. Ann N Y Acad Sci. 2008; 200-7.	Total diabetes, type 1 diabetes
Goswami AK, Gupta SK, Kalaivani M, Nongkynrih B, Pandav CS. Burden of Hypertension and Diabetes among Urban Population Aged ≥ 60 years in South Delhi: A Community Based Study. J Clin Diagn Res. 2016; 10(3): LC01-05.	Total diabetes
Gourdy P, Ruidavets JB, Ferrieres J, Ducimetiere P, Amouyel P, Arveiler D, Cottel D, Lamamy N, Bingham A, Hanaire-Broutin H; MONICA Study. Prevalence of type 2 diabetes and impaired fasting glucose in the middle-aged population of three French regions - the MONICA study 1995-97. Diabetes Metab. 2001; 27(3): 347-58.	Total diabetes
Gouveri ET, Tzavara C, Drakopanagiotakis F, Tsaoussoglou M, Marakomichelakis GE, Tountas Y, Diamantopoulos EJ. Mediterranean diet and metabolic syndrome in an urban population: the Athens Study. Nutr Clin Pract. 2011; 26(5): 598–606.	Total diabetes
Government of Uruguay, Ministry of Public Health (Uruguay). Uruguay STEPS Noncommunicable Disease Risk Factors Survey 2013-2014. Geneva, Switzerland: World Health Organization (WHO).	Total diabetes
Graciani A, Leon-Munoz LM, Guallar-Castillon P, Rodriguez-Artalejo F, Banegas JR. Cardiovascular health in a southern Mediterranean European country: a nationwide population-based study. Circ Cardiovasc Qual Outcomes. 2013; 6(1): 90–8.	Total diabetes
Grandinetti A, Kaholokula JK, Theriault AG, Mor JM, Chang HK, Waslien C. Prevalence of diabetes and glucose intolerance in an ethnically diverse rural community of Hawaii. Ethn Dis. 2007; 17(2): 250-5.	Total diabetes

Gregory CO, Dai J, Ramirez-Zea M, Stein AD. Occupation is more important than rural or urban residence in explaining the prevalence of metabolic and cardiovascular disease risk in Guatemalan adults. J Nutr. 2007; 137(5): 1314-9.	Total diabetes
Guerrero-Romero F, Violante R, Rodriguez-Moran M. Distribution of fasting plasma glucose and prevalence of impaired fasting glucose, impaired glucose tolerance and type 2 diabetes in the Mexican paediatric population. Paediatr Perinat Epidemiol. 2009; 23(4): 363-9.	Total diabetes
Gunaid AA, Assabri AM. Prevalence of type 2 diabetes and other cardiovascular risk factors in a semirural area in Yemen. East Mediterr Health J. 2008; 14(1): 42-56.	Total diabetes
Gundogan K, Bayram F, Capak M, Tanriverdi F, Karaman A, Ozturk A, Altunbas H, Gokce C, Kalkan A, Yazici C. Prevalence of metabolic syndrome in the Mediterranean region of Turkey: evaluation of hypertension, diabetes mellitus, obesity, and dyslipidemia. Metab Syndr Relat Disord. 2009; 7(5): 427–34.	Total diabetes
Gupta R, Deedwania PC, Achari V, Asirvatham AJ, Bhansali A, Gupta A, Gupta B, Gupta S, Jali MV, Mahanta TG, Maheshwari A, Saboo B, Singh J. India Heart Watch Study 2005-2009. [Unpublished].	Total diabetes
Gupta R, Misra A, Vikram NK, Kondal D, Gupta SS, Agrawal A, Pandey RM. Younger age of escalation of cardiovascular risk factors in Asian Indian subjects. BMC Cardiovasc Disord. 2009; 28.	Total diabetes
Gupta R, Sharma KK, Gupta BK, Gupta A, Saboo B, Maheshwari A, Mahanta T, Deedwania PC. Geographic epidemiology of cardiometabolic risk factors in middle class urban residents in India: cross–sectional study. J Glob Health. 2015; 5(1): 010411.	Total diabetes
Gyaneshwar R, Naidu S, Raban MZ, Naidu S, Linhart C, Morrell S, Tukana I, Taylor R. Absolute cardiovascular risk in a Fiji medical zone. BMC Public Health. 2016; 16: 128.	Total diabetes
Gyurus EK, Patterson C, Soltesz G. Twenty-one years of prospective incidence of childhood type 1 diabetes in Hungary--the rising trend continues (or peaks and highlands?). Pediatr Diabetes. 2012; 13(1): 21-5.	Total diabetes, type 1 diabetes
Haba-Rubio J, Marques-Vidal P, Andries D, Tobback N, Preisig M, Vollenweider P, Waeber G, Luca G, Tafti M, Heinzer R. Objective sleep structure and cardiovascular risk factors in the general population: the HypnoLaus Study. Sleep. 2015; 38(3): 391–400.	Total diabetes
Habeb AM, Al-Magamsi MS, Halabi S, Eid IM, Shalaby S, Bakoush O. High incidence of childhood type 1 diabetes in Al-Madinah, North West Saudi Arabia (2004-2009). Pediatr Diabetes. 2011; 12(8): 676-81.	Total diabetes, type 1 diabetes
Hammami S, Mehri S, Hajem S, Koubaa N, Souid H, Hammami M. Prevalence of diabetes mellitus among non institutionalized elderly in Monastir City. BMC Endocr Disord. 2012; 15.	Total diabetes
Hanninen M-RA, Niiranen TJ, Puukka PJ, Johansson J, Jula AM. Prognostic significance of masked and white-coat hypertension in the general population: the Finn-Home Study. J Hypertens. 2012; 30(4): 705–12.	Total diabetes
Hansen TW, Staessen JA, Torp-Pedersen C, Rasmussen S, Li Y, Dolan E, Thijs L, Wang J-G, O Brien E, Ibsen H. Ambulatory arterial stiffness index predicts stroke in a general population. J Hypertens. 2006; 24(11): 2247-54.	Total diabetes
Harjutsalo V, Forsblom C, Groop PH. Time trends in mortality in patients with type 1 diabetes: nationwide population based cohort study. BMJ. 2011; 343(7824): d5364.	Type 1 diabetes
Harjutsalo V, Sjberg L, Tuomilehto J. Time trends in the incidence of type 1 diabetes in Finnish children: a cohort study. Lancet. 2008; 371(9626): 1777-82.	Total diabetes, type 1 diabetes
Harjutsalo V, Sund R, Knip M, Groop PH. Incidence of type 1 diabetes in Finland. JAMA. 2013; 310(4): 427-8.	Total diabetes, type 1 diabetes
Haynes A, Bower C, Bulsara MK, Jones TW, Davis EA. Continued increase in the incidence of childhood Type 1 diabetes in a population-based Australian sample (1985-2002). Diabetologia. 2004; 47(5): 866-70.	Total diabetes, type 1 diabetes
He Y-H, Jiang G-X, Yang Y, Huang H-E, Li R, Li X-Y, Ning G, Cheng Q. Obesity and its associations with hypertension and type 2 diabetes among Chinese adults age 40 years and over. Nutrition. 2009; 25(11-12): 1143–9.	Total diabetes
Health Canada, Public Health Agency of Canada, Statistics Canada (StatCan). Canada Health Measures Survey 2007-2009. Ottawa, Canada: Statistics Canada (StatCan).	Total diabetes
Health Canada, Public Health Agency of Canada, Statistics Canada (StatCan). Canada Health Measures Survey 2009-2011. Ottawa, Canada: Statistics Canada (StatCan).	Total diabetes
Health of Populations in Transition (HoPiT) Research Group (Cameroon), Ministry of Public Health (Cameroon), World Diabetes Foundation (WDF), World Health Organization (WHO). Cameroon STEPS Noncommunicable Disease Risk Factors Survey 2003.	Total diabetes
Health Promotion Administration, Ministry of Health and Welfare (Taiwan). Taiwan National Nutrition and Health Survey 2013-2016.	Total diabetes
Helgason T, Danielsen R, Thorsson AV. Incidence and prevalence of type 1 (insulin-dependent) diabetes mellitus in Icelandic children 1970-1989. Diabetologia. 1992; 35(9): 880-3.	Total diabetes, type 1 diabetes
Herings RM, de Boer A, Stricker BH, Bakker A, Sturmans F. A rapid method to estimate the incidence rate and prevalence of insulin-dependent diabetes mellitus in children 0-19 years of age. Pharm World Sci. 1995; 17(1): 17-9.	Total diabetes
Hiltunen L, Luukinen H, Koski K, Kivel,, SL. Prevalence of diabetes mellitus in an elderly Finnish population. Diabet Med. 1994; 11(3): 241-9.	Total diabetes
Ho SC, Chen YM, Woo JL, Leung SS, Lam TH, Janus ED. Association between simple anthropometric indices and cardiovascular risk factors. Int J Obes Relat Metab Disord. 2001; 25(11): 1689-97.	Total diabetes
Hodge AM, Dowse GK, Zimmet PZ, Gareeboo H, Westerman RA, Tuomilehto J, Alberti KG. Factors associated with impaired vibration perception in Mauritians with normal and abnormal glucose tolerance. Mauritius NCD Study Group. J Diabet Complications. 1995; 9(3): 149-57.	Total diabetes
Hollman G, Kristenson M. The prevalence of the metabolic syndrome and its risk factors in a middle-aged Swedish population--mainly a function of overweight?. Eur J Cardiovasc Nurs. 2008; 7(1): 21-6.	Total diabetes
Ho-Pham LT, Nguyen UDT, Tran TX, Nguyen TV. Discordance in the diagnosis of diabetes: Comparison between HbA1c and fasting plasma glucose. PLoS One. 2017; 12(8): e0182192.	Total diabetes
Hu Y, Teng W, Liu L, Chen K, Liu L, Hua R, Chen J, Zhou Y, Chen L. Prevalence and risk factors of diabetes and diabetic retinopathy in Liaoning province, China: a population-based cross-sectional study. PLoS One. 2015; 10(3): e0121477.	Total diabetes
Huffman MD, Prabhakaran D, Osmond C, Fall CHD, Tandon N, Lakshmy R, Ramji S, Khalil A, Gera T, Prabhakaran P, Biswas SKD, Reddy KS, Bhargava SK, Sachdev HS, New Delhi Birth Cohort. Incidence of cardiovascular risk factors in an Indian urban cohort results from the New Delhi birth cohort. J Am Coll Cardiol. 2011; 57(17): 1765-74.	Total diabetes
Hussain A, Rahim MA, Azad Khan AK, Ali SM, Vaaler S. Type 2 diabetes in rural and urban population: diverse prevalence and associated risk factors in Bangladesh. Diabet Med. 2005; 22(7): 931-6.	Total diabetes
Hussain A, Vaaler S, Sayeed MA, Mahtab H, Ali SM, Khan AK. Type 2 diabetes and impaired fasting blood glucose in rural Bangladesh: a population-based study. Eur J Public Health. 2007; 17(3): 291-6.	Total diabetes

Husseini A, Abdul-Rahim H, Awartani F, Jervell J, Bjertness E. Prevalence of diabetes mellitus and impaired glucose tolerance in a rural Palestinian population. East Mediterr Health J. 2000; 6(5-6): 1039-45.	Total diabetes
ICF International, Mitra and Associates, National Institute of Population Research and Training (NIPORT). Bangladesh Demographic and Health Survey 2017-2018. Fairfax, United States of America: ICF International, 2020.	Total diabetes
ICF Macro, Mitra and Associates, National Institute of Population Research and Training (NIPORT). Bangladesh Demographic and Health Survey 2011-2012. Calverton, United States of America: ICF Macro.	Total diabetes
Illangasekera U, Nugegoda DB, Perera LS. Prevalence of diabetes mellitus and impaired glucose tolerance in a rural Sri Lankan community. Ceylon Med J. 1993; 38(3): 123-6.	Total diabetes
Ilow R, Regulska-Ilow B, Rozanska D, Kowalisko A, Biernat J. Prevalence of metabolic syndrome among 40- and 50-year-old inhabitants of Wroclaw, Poland. Ann Agric Environ Med. 2012; 19(3): 551–6.	Total diabetes
Imamura Y, Uto H, Hiramine Y, Hosoyamada K, Ijuin S, Yoshifuku S, Miyahara H, Maenohara S, Oketani M, Ido A, Tsubouchi H. Increasing prevalence of diabetes mellitus in association with fatty liver in a Japanese population. J Gastroenterol. 2013.	Total diabetes
India Prevalence of Risk Factors for Non-Communicable Diseases in Rural and Urban Tamil Nadu 2010-2012.	Total diabetes
Indian Council of Medical Research (ICMR), Madras Diabetes Research Foundation. Indian Council of Medical Research India Diabetes Study (ICMR-INDIAB) 2008-2010.	Total diabetes
Indian Council of Medical Research (ICMR), National Institute of Health and Family Welfare (India), Nutrition Foundation of India, Office of the Registrar General and Census Commissioner (India). India Clinical, Anthropometric and Bio-chemical Survey 2014.	Total diabetes
Indian Council of Medical Research (ICMR). Indian Council of Medical Research India Diabetes Study (ICMR-INDIAB) - North East 2012-2013.	Total diabetes
Indian Council of Medical Research (ICMR). Indian Council of Medical Research India Diabetes Study (ICMR-INDIAB) 2012-2013.	Total diabetes
International Diabetes Institute (IDI). Australia Diabetes, Obesity and Lifestyle Study 1999-2000. Melbourne, Australia: International Diabetes Institute (IDI).	Total diabetes
Ionescu-Tîrgovişte C, Guja C, Cãlin A, Moţa M. An increasing trend in the incidence of type 1 diabetes mellitus in children aged 0-14 years in Romania--ten years (1988-1997) EURODIAB study experience. J Pediatr Endocrinol Metab. 2004; 17(7): 983-91.	Total diabetes, type 1 diabetes
Ionescu-Tirgoviste C, Paterache E, Cheta D, Farcasiu E, Serafinceanu C, Mincu I. Epidemiology of diabetes in Bucharest. Diabet Med. 1994; 11(4): 413-7.	Total diabetes, type 1 diabetes
Isezuo SA, Sabir AA, Ohwovorilole AE, Fasanmade OA. Prevalence, associated factors and relationship between prehypertension and hypertension: a study of two ethnic African populations in Northern Nigeria. J Hum Hypertens. 2011; 25(4): 224–30.	Total diabetes
Islam FM, Chakrabarti R, Islam MT, Wahab M, Lamoureux E, Finger RP, Shaw JE. Prediabetes, diagnosed and undiagnosed diabetes, their risk factors and association with knowledge of diabetes in rural Bangladesh: The Bangladesh Population-based Diabetes and Eye Study. J Diabetes. 2015; 8(2): nan.	Total diabetes
Israel Center for Disease Control (ICDC). Israel Juvenile Diabetes Registry Report 2005-2007. Jerusalem, Israel: Ministry of Health (Israel), 2010.	Total diabetes, type 1 diabetes
Israel Center for Disease Control (ICDC). Israel Juvenile Diabetes Registry Report 2008-2009. Jerusalem, Israel: Ministry of Health (Israel), 2011.	Total diabetes, type 1 diabetes
Israel Center for Disease Control (ICDC). Israel Juvenile Diabetes Registry Report 2010. Jerusalem, Israel: Ministry of Health (Israel), 2012.	Total diabetes, type 1 diabetes
Israel Center for Disease Control (ICDC). Israel Juvenile Diabetes Registry Report 2011. Jerusalem, Israel: Ministry of Health (Israel), 2013.	Total diabetes, type 1 diabetes
Israel Center for Disease Control (ICDC). Israel Juvenile Diabetes Registry Report 2012. Jerusalem, Israel: Ministry of Health (Israel), 2014.	Total diabetes, type 1 diabetes
Israel Center for Disease Control (ICDC). Israel Juvenile Diabetes Registry Report 2013. Jerusalem, Israel: Ministry of Health (Israel), 2015.	Total diabetes, type 1 diabetes
Israel Center for Disease Control (ICDC). Israel Juvenile Diabetes Registry Report 2014. Jerusalem, Israel: Ministry of Health (Israel), 2016.	Total diabetes, type 1 diabetes
Israel Center for Disease Control (ICDC). Israel Juvenile Diabetes Registry Report 2015. Jerusalem, Israel: Ministry of Health (Israel), 2017.	Total diabetes, type 1 diabetes
Jørgensen ME, Borch-Johnsen K, Witte DR, Bjerregaard P. Diabetes in Greenland and its relationship with urbanization. Diabet Med. 2012; 29(6): 755-60.	Total diabetes
Jacobs JM, Stessman J, Ein-Mor E, Bursztyn M. Hypertension and 5-year mortality among 85-year-olds: the Jerusalem Longitudinal Study. J Am Med Dir Assoc. 2012; 13(8): 759e1–6.	Total diabetes
Jarosz-Chobot P, Otto-Buczkowska E, Koehler B, Matlakiewicz E, Green A. Increased trend of type 1 diabetes mellitus in children's population (0-14 years) in Upper Silesia region (Poland). Med Sci Monit. 2000; 6(3): 573-80.	Total diabetes, type 1 diabetes
Jarosz-Chobot P, Polanska J, Szadkowska A, Kretowski A, Bandurska-Stankiewicz E, Ciechanowska M, Deja G, Mysliwiec M, Peczynska J, Rutkowska J, Sobel-Maruniak A, Fichna P, Chobot A, Rewers M. Rapid increase in the incidence of type 1 diabetes in Polish children from 1989 to 2004, and predictions for 2010 to 2025. Diabetologia. 2011; 54(3): 508-15.	Total diabetes, type 1 diabetes
Jeenduang N, Whanmasae S, Seepawin P, Kullabootr S. The prevalence of dyslipidemia among a rural Thai population in the Nakhon Si Thammarat province. J Med Assoc Thai. 2013; 96(8): 992–1000.	Total diabetes
Jermendy G, Nadas J, Szigethy E, Szeles G, Nagy A, Hidvegi T, Paragh G, Adany R. Prevalence rate of diabetes mellitus and impaired fasting glycemia in Hungary: cross-sectional study on nationally representative sample of people aged 20-69 years. Croat Med J. 2010; 51(2): 151-6.	Total diabetes
Joint Health Surveys Unit of Social and Community Planning Research and University College London, Health Survey for England, 1994 [computer file]. 4th ed. Colchester, Essex: UK Data Archive [distributor], 26 March 2001. SN: 3640.	Total diabetes
Joint Health Surveys Unit, University College London and Medical Research Council. Social and Public Health Sciences Unit, Scottish Health Survey, 2003 [computer file]. Colchester, Essex: UK Data Archive [distributor], February 2006. SN: 5318.	Total diabetes
Joner G, Stene LC, S?vik O. Nationwide, prospective registration of type 1 diabetes in children aged Diabetes Care. 2004; 27(7): 1618-22.	Total diabetes, type 1 diabetes
Jordan University of Science and Technology, National Center for Diabetes, Endocrinology, and Genetics (NCDEG) (Jordan). Jordan Cardiovascular Disease Risk Factor Survey 2009.	Total diabetes

Jordan University of Science and Technology, National Center for Diabetes, Endocrinology, and Genetics (NCDEG) (Jordan). Jordan Cardiovascular Disease Risk Factor Survey 2017.	Total diabetes
Kadiki OA, Moawad SE. Incidence and prevalence of type 1 diabetes in children and adolescents in Benghazi, Libya. Diabet Med. 1993; 10(9): 866-9.	Total diabetes
Kadiki OA, Reddy MR, Marzouk AA. Incidence of insulin-dependent diabetes (IDDM) and non-insulin-dependent diabetes (NIDDM) (0-34 years at onset) in Benghazi, Libya. Diabetes Res Clin Pract. 1996; 32(3): 165-73.	Total diabetes, type 1 diabetes
Kadiki OA, Roaed RB. Epidemiological and clinical patterns of diabetes mellitus in Benghazi, Libyan Arab Jamahiriya. East Mediterr Health J. 1999; 5(1): 6-13.	Total diabetes
Kadiki OA, Roaeid RB, Bhairi AM, Elamari IM. Incidence of insulin-dependent diabetes mellitus in Benghazi, Libya (1991-1995). Diabetes Metab. 1998; 24(5): 424-7.	Total diabetes, type 1 diabetes
Kadiki OA, Roaeid RB. Prevalence of diabetes mellitus and impaired glucose tolerance in Benghazi Libya. Diabetes Metab. 2001; 27(6): 647-54.	Total diabetes
Kailuan General Hospital, Hebei United University. China - KaiLuan Study 2008-2009.	Total diabetes
Kailuan General Hospital, Hebei United University. China - KaiLuan Study 2010-2011.	Total diabetes
Kailuan General Hospital, Hebei United University. China - KaiLuan Study 2012-2014.	Total diabetes
Kailuan General Hospital, Hebei United University. China - KaiLuan Study 2014-2016.	Total diabetes
Kailuan General Hospital, Hebei United University. China - KaiLuan Study Baseline 2006-2007.	Total diabetes
Kaiser A, Vollenweider P, Waeber G, Marques-Vidal P. Prevalence, awareness and treatment of type 2 diabetes mellitus in Switzerland: the CoLaus study. Diabet Med. 2012; 29(2): 190-7.	Total diabetes
Kalits I, Podar T. Incidence and prevalence of type 1 (insulin-dependent) diabetes in Estonia in 1988. Diabetologia. 1990; 33(6): 346-9.	Total diabetes, type 1 diabetes
Kalra S, Kalra B, Sharma A. Prevalence of type 1 diabetes mellitus in Karnal district, Haryana state, India. Diabetol Metab Syndr. 2010; 14.	Total diabetes, type 1 diabetes
Kalter-Leibovici O, Chetrit A, Lubin F, Atamna A, Alpert G, Ziv A, Abu-Saad K, Murad H, Eilat-Adar S, Goldbourt U. Adult-onset diabetes among Arabs and Jews in Israel: a population-based study. Diabet Med. 2012; 29(6): 748-54.	Total diabetes
Kamble P, Deshmukh PR, Garg N. Metabolic syndrome in adult population of rural Wardha, central India. Indian J Med Res. 2010; 132: 701–5.	Total diabetes
Kanerva N, Rissanen H, Knekt P, Havulinna AS, Eriksson JG, Mannisto S. The healthy Nordic diet and incidence of Type 2 Diabetes--10-year follow-up. Diabetes Res Clin Pract. 2014; 106(2): e34-7.	Total diabetes
Karalis IK, Alegakis AK, Kafatos AG, Koutis AD, Vardas PE, Lionis CD. Risk factors for ischaemic heart disease in a Cretan rural population: a twelve year follow-up study. BMC Public Health. 2007; 351.	Total diabetes
Karolinska Institute (Sweden), Statistics Sweden, Stockholm County Council. Sweden - Stockholm Public Health Survey 2006-2007.	Total diabetes
Karvonen M, Viik-Kajander M, Moltchanova E, Libman I, LaPorte R, Tuomilehto J. Incidence of childhood type 1 diabetes worldwide. Diabetes Mondiale (DiaMond) Project Group. Diabetes Care. 2000; 23(10): 1516–26.	Total diabetes, type 1 diabetes
Katte J-C, Dzudie A, Sobngwi E, Mbong EN, Fetse GT, Kouam CK, Kengne A-P. Coincidence of diabetes mellitus and hypertension in a semi-urban Cameroonian population: a cross-sectional study. BMC Public Health. 2014; 14: 696.	Total diabetes
Kazakh Academy of Nutrition, Ministry of Education and Science (Kazakhstan), Republican State Enterprise (Kazakhstan). Kazakhstan Complex Prevention of the Epidemic of Excessive Body Mass and Obesity 2012.	Total diabetes
Kelestimur F, Cetin M, Paşaoğlu H, Coksevim B, Cetinkaya F, Unlühizarci K, Unal S, Köker AH. The prevalence and identification of risk factors for type 2 diabetes mellitus and impaired glucose tolerance in Kayseri, central Anatolia, Turkey. Acta Diabetol. 1999; 36(1-2): 85-91.	Total diabetes
Kelly HA, Russell MT, Jones TW, Byrne GC. Dramatic increase in incidence of insulin dependent diabetes mellitus in Western Australia. Med J Aust. 1994; 161(7): 426-9.	Total diabetes, type 1 diabetes
Kenya National Bureau of Statistics, Ministry of Health (Kenya), World Health Organization (WHO). Kenya STEPS Noncommunicable Disease Risk Factors Survey 2015.	Total diabetes
Khader Y, Batieha A, Ajlouni H, El-Khateeb M, Ajlouni K. Obesity in Jordan: prevalence, associated factors, comorbidities, and change in prevalence over ten years. Metab Syndr Relat Disord. 2008; 6(2): 113-20.	Total diabetes
Khan FS, Lotia-Farrukh I, Khan AJ, Siddiqui ST, Sajun SZ, Malik AA, Burfat A, Arshad MH, Codlin AJ, Reininger BM, McCormick JB, Afridi N, Fisher-Hoch SP. The burden of non-communicable disease in transition communities in an Asian megacity: baseline findings from a cohort study in Karachi, Pakistan. PLoS One. 2013; 8(2): e56008.	Total diabetes
Khebir BV, Osman A, Khalid BA. Changing prevalence of diabetes mellitus amongst rural Malays in Kuala Selangor over a 10-year period. Med J Malaysia. 1996; 51(1): 41-7.	Total diabetes
Khedr EM, Fawi G, Allah Abbas MA, El-Fetoh NA, Al Attar G, Zaki AF, Gamea A. Prevalence of Diabetes and Diabetic Neuropathy in Qena Governorate: Population-Based Survey. Neuroepidemiology. 2016; 46(3): 173–81.	Type 1 diabetes
Kheirandish M, Asgari S, Lotfaliany M, Bozorgmanesh M, Saadat N, Tohidi M, Azizi F, Hadaegh F. Secular trends in serum lipid levels of a Middle Eastern adult population; 10 years follow up in Tehran lipid and glucose study. Lipids Health Dis. 2014; 13: 20.	Total diabetes
Kida K, Mimura G, Ito T, Murakami K, Ashkenazi I, Laron Z. Incidence of Type 1 diabetes mellitus in children aged 0-14 in Japan, 1986-1990, including an analysis for seasonality of onset and month of birth: JDS study. The Data Committee for Childhood Diabetes of the Japan Diabetes Society (JDS). Diabet Med. 2000; 17(1): 59-63.	Total diabetes, type 1 diabetes
Kim G, Lee YH, Lee BW, Kang ES, Lee IK, Cha BS, Kim DJ. Diabetes self-assessment score and the development of diabetes: A 10-year prospective study. Medicine (Baltimore). 2017; 96(23): e7067.	Total diabetes
Kim JH, Lee CG, Lee YA, Yang SW, Shin CH. Increasing incidence of type 1 diabetes among Korean children and adolescents: analysis of data from a nationwide registry in Korea. Pediatr Diabetes. 2016; 17(7): 519–24.	Total diabetes, type 1 diabetes
Kim JM. Vascular disease/risk and late-life depression in a Korean community population. Br J Psychiatry. 2004; 185(2): 102-7.	Total diabetes
Kim SG, Yang SW, Jang AS, Seo JP, Han SW, Yeom CH, Kim YC, Oh SH, Kim JS, Nam HS, Chung DJ, Chung MY. Prevalence of diabetes mellitus in the elderly of Namwon County, South Korea. Korean J Intern Med. 2002; 17(3): 180-90.	Total diabetes
King H, Abdullaev B, Djumaeva S, Nikitin V, Ashworth L, Dobo MG. Glucose intolerance and associated factors in the Fergana Valley, Uzbekistan. Diabet Med. 1998; 15(12): 1052–62.	Total diabetes

King H, Taylor R, Koteka G, Nemaia H, Zimmet P, Bennett PH, Raper LR. Glucose tolerance in Polynesia. Population-based surveys in Rarotonga and Niue. Med J Aust. 1986; 145(10): 505-10.	Total diabetes
Kobayashi J, Nishimura K, Matoba M, Maekawa N, Mabuchi H. Generation and Gender Differences in the Components Contributing to the Diagnosis of the Metabolic Syndrome According to the Japanese Criteria. Circ J. 2007; 71(11): 1734-7.	Total diabetes
Kondrashova A, Reunanen A, Romanov A, Karvonen A, Viskari H, Vesikari T, Ilonen J, Knip M, Hyöty H. A six-fold gradient in the incidence of type 1 diabetes at the eastern border of Finland. Ann Med. 2005; 37(1): 67-72.	Total diabetes, type 1 diabetes
Korea Centers for Disease Control and Prevention (KCDC). South Korea National Health and Nutrition Examination Survey 1998.	Total diabetes
Korea Centers for Disease Control and Prevention (KCDC). South Korea National Health and Nutrition Examination Survey 2001.	Total diabetes
Korea Centers for Disease Control and Prevention (KCDC). South Korea National Health and Nutrition Examination Survey 2005.	Total diabetes
Korea Centers for Disease Control and Prevention (KCDC). South Korea National Health and Nutrition Examination Survey 2007.	Total diabetes
Korea Centers for Disease Control and Prevention (KCDC). South Korea National Health and Nutrition Examination Survey 2008.	Total diabetes
Korea Centers for Disease Control and Prevention (KCDC). South Korea National Health and Nutrition Examination Survey 2009.	Total diabetes
Korea Centers for Disease Control and Prevention (KCDC). South Korea National Health and Nutrition Examination Survey 2010.	Total diabetes
Korea Centers for Disease Control and Prevention (KCDC). South Korea National Health and Nutrition Examination Survey 2011.	Total diabetes
Korea Centers for Disease Control and Prevention (KCDC). South Korea National Health and Nutrition Examination Survey 2012.	Total diabetes
Korea Centers for Disease Control and Prevention (KCDC). South Korea National Health and Nutrition Examination Survey 2013. Osong-eup, Republic of Korea: Korea Centers for Disease Control and Prevention (KCDC).	Total diabetes
Korea Centers for Disease Control and Prevention (KCDC). South Korea National Health and Nutrition Examination Survey 2014.	Total diabetes
Korea Centers for Disease Control and Prevention (KCDC). South Korea National Health and Nutrition Examination Survey 2015.	Total diabetes
Korea Centers for Disease Control and Prevention (KCDC). South Korea National Health and Nutrition Examination Survey 2016. Osong-eup, Republic of Korea: Korea Centers for Disease Control and Prevention (KCDC).	Total diabetes
Korea Centers for Disease Control and Prevention (KCDC). South Korea National Health and Nutrition Examination Survey 2017. Osong-eup, Republic of Korea: Korea Centers for Disease Control and Prevention (KCDC).	Total diabetes
Korea Centers for Disease Control and Prevention (KCDC). South Korea National Health and Nutrition Examination Survey 2018. Osong-eup, Republic of Korea: Korea Centers for Disease Control and Prevention (KCDC).	Total diabetes
Korea Centers for Disease Control and Prevention (KCDC). South Korea National Health and Nutrition Examination Survey 2019. Osong-eup, Republic of Korea: Korea Centers for Disease Control and Prevention (KCDC).	Total diabetes
Kretowski A, Kowalska I, Peczyñska J, Urban M, Green A, Kinalska I. The large increase in incidence of Type I diabetes mellitus in Poland. Diabetologia. 2001; B48-50.	Total diabetes, type 1 diabetes
Krishnadath ISK, Nahar-van Venrooij LM, Jaddoe VWV, Toelsie JR. Ethnic differences in prediabetes and diabetes in the Suriname Health Study. BMJ Open Diabetes Res Care. 2016; 4(1): e000186.	Total diabetes
Kumar P, Krishna P, Reddy SC, Gurappa M, Aravind SR, Munichoodappa C. Incidence of type 1 diabetes mellitus and associated complications among children and young adults: results from Karnataka Diabetes Registry 1995-2008. J Indian Med Assoc. 2008; 106(11): 708–11.	Total diabetes, type 1 diabetes
Kuo R-J, Wu Y-H, Chen L-K. Inability of waist-to-height ratio to predict new onset diabetes mellitus among older adults in Taiwan: a five-year observational cohort study. Arch Gerontol Geriatr. 2011; 53(1): e1–4.	Total diabetes
Kweon S-S, Shin M-H, Park K-S, Nam H-S, Jeong S-K, Ryu S-Y, Chung E-K, Choi J-S. Distribution of the ankle-brachial index and associated cardiovascular risk factors in a population of middle-aged and elderly koreans. J Korean Med Sci. 2005; 20(3): 373-8.	Total diabetes
López-Siguero JP, Del Pino-De la Fuente A, Martínez-Aedo MJ, Moreno-Molina JA. Increased incidence of type 1 diabetes in the south of Spain. Diabetes Care. 2002; 25(6): 1099.	Total diabetes, type 1 diabetes
Lai S-W, Tan C-K. Epidemiology of Hyperglycemia in Elderly Persons. J Gerontol A Biol Sci Med Sci. 2000; 55A(5): M257-9.	Total diabetes
Laing SP, Swerdlow AJ, Slater SD, Botha JL, Burden AC, Waugh NR, Smith AW, Hill RD, Bingley PJ, Patterson CC, Qiao Z, Keen H. The British Diabetic Association Cohort Study, I: all-cause mortality in patients with insulin-treated diabetes mellitus. Diabet Med. 1999; 16(6): 459-65.	Type 1 diabetes
Lapertosa S GC, Benítez J, Céspedes MS, Bordón C, de Loredo L, Santoro S, de Sereday M. Prevalencia de Diabetes Mellitus tipo 2 en población adulta de Gobernador Virasoro, Provincia de Corrientes. Rev Asoc Latinoam Diabetes. 2009; 89-96.	Total diabetes
Larenas G, Montecinos A, Manosalva M, Barthou M, Vidal T. Incidence of insulin-dependent diabetes mellitus in the IX region of Chile: ethnic differences. Diabetes Res Clin Pract. 1996; S147-51.	Total diabetes, type 1 diabetes
Laron-Kenet T, Shamis I, Weitzman S, Rosen S, Laron ZV. Mortality of patients with childhood onset (0-17 years) Type I diabetes in Israel: a population-based study. Diabetologia. 2001; B81-6.	Type 1 diabetes
Le C, Jun D, Zhankun S, Yichun L, Jie T. Socioeconomic differences in diabetes prevalence, awareness, and treatment in rural southwest China. Trop Med Int Health. 2011; 16(9): 1070-6.	Total diabetes
Lee CH, Shih AZL, Woo YC, Fong CHY, Leung OY, Janus E, Cheung BMY, Lam KSL. Optimal Cut-Offs of Homeostasis Model Assessment of Insulin Resistance (HOMA-IR) to Identify Dysglycemia and Type 2 Diabetes Mellitus: A 15-Year Prospective Study in Chinese. PLoS One. 2016; 11(9): e0163424.	Total diabetes
Lee H-S, Park Y-M, Kwon H-S, Lee J-H, Park YJ, Lim SY, Lee S-H, Yoon K-H, Son H-Y, Kim DS, Yim HW, Lee W-C. Prevalence, awareness, treatment, and control of hypertension among people over 40 years old in a rural area of South Korea: The Chungju Metabolic Disease Cohort (CMC) Study. Clin Exp Hypertens. 2010; 32(3): 166–78.	Total diabetes
Lee HY, Won JC, Kang YJ, Yoon SH, Choi EO, Bae JY, Sung MH, Kim H-R, Yang JH, Oh J, Lee YM, Park NH, Ko KS, Rhee BD. Type 2 diabetes in urban and rural districts in Korea: factors associated with prevalence difference. J Korean Med Sci. 2010; 25(12): 1777-83.	Total diabetes
Lessa I, Magalhães L, Araújo MJ, de Almeida Filho N, Aquino E, Oliveira MM. Arterial hypertension in the adult population of Salvador (BA)--Brazil. Arq Bras Cardiol. 2006; 87(6): 747-56.	Total diabetes
Levitt NS, Katzenellenbogen JM, Bradshaw D, Hoffman MN, Bonnici F. The prevalence and identification of risk factors for NIDDM in urban Africans in Cape Town, South Africa. Diabetes Care. 1993; 16(4): 601-7.	Total diabetes

Levy-Marchal C, Czernichow P. Heterogeneity of type 1 diabetes at onset in children: results from the French Incidence Study. Diabete Metab. 1993; 19(3): 296-303.	Total diabetes, type 1 diabetes
Levy-Marchal C, Papoz L, de Beaufort C, Doutreix J, Froment V, Voirin J, Collignon A, Garros B, Schleret Y, Czernichow P. Incidence of juvenile type 1 (insulin-dependent) diabetes mellitus in France. Diabetologia. 1990; 33(8): 465-9.	Total diabetes, type 1 diabetes
Li XH, Li TL, Yang Z, Liu ZY, Wei YD, Jin SX, Hong C, Qin RL, Li YQ, Dorman JS, Laporte RE, Wang KA. A nine-year prospective study on the incidence of childhood type 1 diabetes mellitus in China. Biomed Environ Sci. 2000; 13(4): 263-70.	Type 1 diabetes
Liang J, Zhang Y, Xue A, Sun J, Song X, Xue B, Ji F, Gao W, He L, Pang Z, Qiao Q, Ning F. Association between fruit, vegetable, seafood, and dairy intake and a reduction in the prevalence of type 2 diabetes in Qingdao, China. Asia Pac J Clin Nutr. 2017; 26(2): 255-261.	Total diabetes
Lim N-K, Park S-H, Choi S-J, Lee K-S, Park H-Y. A risk score for predicting the incidence of type 2 diabetes in a middle-aged Korean cohort: the Korean genome and epidemiology study. Circ J. 2012; 76(8): 1904–10.	Total diabetes
Lin C-C, Liu C-S, Lai M-M, Li C-I, Chen C-C, Chang P-C, Lin W-Y, Lee Y-D, Lin T, Li T-C. Metabolic syndrome in a Taiwanese metropolitan adult population. BMC Public Health. 2007; 239.	Total diabetes
Lin C-C, Liu C-S, Li T-C, Chen C-C, Li C-I, Lin W-Y. Microalbuminuria and the metabolic syndrome and its components in the Chinese population. Eur J Clin Invest. 2007; 37(10): 783-90.	Total diabetes
Lin L, Chen G, Zou X, Zhao J, Zhu F, Tu M, Xu S, Lin W, Yang S, Zhang Y, Lin M, Chen N, Huang H, Liang J, Li L, Yao J. Diabetes, pre-diabetes and associated risks on Minnesota code-indicated major electrocardiogram abnormality among Chinese: a cross-sectional diabetic study in Fujian province, southeast China. Obes Rev. 2009; 10(4): 420-30.	Total diabetes
Lin S, Cheng TO, Liu X, Mai J, Rao X, Gao X, Deng H, Shi M. Impact of dysglycemia, body mass index, and waist-to-hip ratio on the prevalence of systemic hypertension in a lean Chinese population. Am J Cardiol. 2006; 97(6): 839-42.	Total diabetes
Lin WH, Wang MC, Wang WM, Yang DC, Lam CF, Roan JN, Li CY. Incidence of and mortality from Type I diabetes in Taiwan from 1999 through 2010: a nationwide cohort study. PLoS One. 2014; 9(1): e86172.	Total diabetes, type 1 diabetes
Lind H, Nilsson P, Holthuis N, Lindholm L. Non-obese men with high lipoprotein(a) values-- a cardiovascular risk group different from those with the metabolic syndrome?. Scand J Clin Lab Invest. 1994; 54(2): 177-83.	Total diabetes
Lindblad U, Ek J, Eckner J, Larsson CA, Shan G, Rastam L. Prevalence, awareness, treatment, and control of hypertension: rule of thirds in the Skaraborg project. Scand J Prim Health Care. 2012; 30(2): 88–94.	Total diabetes
Lipman TH, Chang Y, Murphy KM. The epidemiology of type 1 diabetes in children in Philadelphia 1990-1994: evidence of an epidemic. Diabetes Care. 2002; 25(11): 1969-75.	Total diabetes, type 1 diabetes
Lipman TH, Jawad AF, Murphy KM, Tuttle A, Thompson RL, Ratcliffe SJ, Levitt Katz LE. Incidence of type 1 diabetes in Philadelphia is higher in black than white children from 1995 to 1999: epidemic or misclassification?. Diabetes Care. 2006; 29(11): 2391-5.	Total diabetes, type 1 diabetes
Liu F, Ma Y-T, Yang Y-N, Zhen Y-J, Xie X, Li X-M, Ma X, Chen B-D, Huang Y, Shan C-F, Gao X-M. The prevalence of isolated systolic hypertension in adult populations from the Han, Uygur and Kazakh ethnic groups in Xinjiang, China. Blood Press. 2014; 23(3): 154–9.	Total diabetes
Liu L, Zhou C, Du H, Zhang K, Huang D, Wu J. The prevalences of impaired fasting glucose and diabetes mellitus in working age men of North China: Anshan Worker Health Survey. Sci Rep. 2014; 4835.	Total diabetes
Liu S, Wang W, Zhang J, He Y, Yao C, Zeng Z, Piao J, Howard BV, Fabsitz RR, Best L, Yang X, Lee ET. Prevalence of diabetes and impaired fasting glucose in Chinese adults, China National Nutrition and Health Survey, 2002. Prev Chronic Dis. 2011; 8(1): A13.	Total diabetes
Lora-Gómez RE, Morales-Pérez FM, Arroyo-Díez FJ, Barquero-Romero J. Incidence of Type 1 diabetes in children in Cáceres, Spain, during 1988-1999. Diabetes Res Clin Pract. 2005; 69(2): 169-74.	Total diabetes, type 1 diabetes
Lu K, Chen J, Wu S, Chen J, Hu D. Interaction of Sleep Duration and Sleep Quality on Hypertension Prevalence in Adult Chinese Males. J Epidemiol. 2015; 25(6): 415–22.	Total diabetes
Lv Y-B, Yin Z-X, Chei C-L, Qian H-Z, Kraus VB, Zhang J, Brasher MS, Shi X-M, Matchar DB, Zeng Y. Low-density lipoprotein cholesterol was inversely associated with 3-year all-cause mortality among Chinese oldest old: data from the Chinese Longitudinal Healthy Longevity Survey. Atherosclerosis. 2015; 239(1): 137–42.	Total diabetes
M.V. Hospital for Diabetes (India). India - Chennai Urban Population Study Blood Glucose, Cholesterol, BMI, and Diabetes Incidence Measurements, 1996-2006. [Unpublished].	Total diabetes
Möller CS, Zethelius B, Sundström J, Lind L. Impact of follow-up time and re-measurement of the electrocardiogram and conventional cardiovascular risk factors on their predictive value for myocardial infarction. J Intern Med. 2006; 260(1): 22-30.	Total diabetes
Macedo A, Jorge Z, Lacerda Nobre E, Pratas S, J come de Castro J. Prevalence of Type 1 diabetes mellitus in Portugal, 1995-1999:cohort of young men. Diabet Med. 2003; 20(5): 418-9.	Total diabetes
Malavige GN, de Alwis NM, Weerasooriya N, Fernando DJ, Siribaddana SH. Increasing diabetes and vascular risk factors in a sub-urban Sri Lankan population. Diabetes Res Clin Pract. 2002; 57(2): 143-5.	Total diabetes
Malerbi DA, Franco LJ. Multicenter study of the prevalence of diabetes mellitus and impaired glucose tolerance in the urban Brazilian population aged 30-69 yr. The Brazilian Cooperative Group on the Study of Diabetes Prevalence. Diabetes Care. 1992; 15(11): 1509-16.	Total diabetes
Malik M, Bakir A, Saab BA, Roglic G, King H. Glucose intolerance and associated factors in the multi-ethnic population of the United Arab Emirates results of a national survey. Diabetes Res Clin Pract. 2005; 69(2): 188-95.	Total diabetes
Mamoulakis D, Galanakis E, Bicouvarakis S, Paraskakis E, Sbyrakis S. Epidemiology of childhood type I diabetes in Crete, 1990-2001. Acta Paediatr. 2003; 92(6): 737-9.	Total diabetes, type 1 diabetes
Mannami T, Baba S, Ogata J. Potential of Carotid Enlargement as a Useful Indicator Affected by High Blood Pressure in a Large General Population of a Japanese City The Suita Study. Stroke. 2000; 31(12): 2958-65.	Total diabetes
Maral I, Tütüncü NB, Bakar C, Durukan E, Budakoğlu II, Ozkan S, Aycan S, Aygün R, Bumin MA. The 5-year incidence of type 2 diabetes mellitus in women older than 15 years in Ankara, Turkey: a population-based study. J Investig Med. 2010; 58(6): 796-800.	Total diabetes
Marcopito LF, Rodrigues SSF, Pacheco MA, Shirassu MM, Goldfeder AJ, de Moraes MAOL. Prevalence of a set of risk factors for chronic diseases in the city of Sao Paulo, Brazil. Rev Saude Publica. 2005; 39(5): 738-45.	Total diabetes
Marigliano M, Tadiotto E, Morandi A, Sabbion A, Contreas G, Avossa F, Fedeli U, Maffeis C. Epidemiology of type 1 diabetes mellitus in the pediatric population in Veneto Region, Italy. Diabetes Res Clin Pract. 2015; 107(3): e19-21.	Total diabetes, type 1 diabetes
Marques-Vidal P, Vollenweider P, Waeber G. Alcohol consumption and incidence of type 2 diabetes. Results from the CoLaus study. Nutr Metab Cardiovasc Dis. 2015; 25(1): 75–84.	Total diabetes
Martinez-Hervas S, Carmena R, Ascaso JF, Real JT, Masana L, Catala M, Vendrell J, Vázquez JA, Valdes S, Urrutia I, Soriguer F, Serrano-Rios M, Rojo-Martínez G, Pascual-Manich G, Ortega E, Mora-Peces I, Menendez E, Martínez-Larrad MT, Lopez-Alba A, Gomis R, Goday A, Girbes J, Gaztambide S, Franch J, Delgado E, Castell C, Castano L, Casamitjana R, Calle-Pascual A, Bordiu E. Prevalence of plasma lipid abnormalities and its association with glucose metabolism in Spain: the diabetes study. Clin Investig Arterioscler. 2014; 26(3): 107–14.	Total diabetes

Martinucci ME, Curradi G, Fasulo A, Medici A, Toni S, Osovik G, Lapistkaya E, Sherbitskaya E. Incidence of childhood type 1 diabetes mellitus in Gomel, Belarus. J Pediatr Endocrinol Metab. 2002; 15(1): 53-7.	Total diabetes, type 1 diabetes
Marzouk D, Sass J, Bakr I, El Hosseiny M, Abdel-Hamid M, Rekacewicz C, Chaturvedi N, Mohamed MK, Fontanet A. Metabolic and cardiovascular risk profiles and hepatitis C virus infection in rural Egypt. Gut. 2007; 56(8): 1105-10.	Total diabetes
Matshipi M, Monyeki KD, Kemper H. The Relationship between Physical Activity and Plasma Glucose Level amongst Ellisras Rural Young Adult Males and Females: Ellisras Longitudinal Study. Int J Environ Res Public Health. 2017; 14(2).	Total diabetes
Mauny F, Grandmottet M, Lestradet C, Guitard J, Crenn D, Floret N, Olivier-Koehret M, Viel JF. Increasing trend of childhood type 1 diabetes in Franche-Comt, (France): analysis of age and period effects from 1980 to 1998. Eur J Epidemiol. 2005; 20(4): 325-9.	Total diabetes, type 1 diabetes
Mayega RW, Guwatudde D, Makumbi F, Nakwagala FN, Peterson S, Tomson G, Ostenson C-G. Diabetes and pre-diabetes among persons aged 35 to 60 years in eastern Uganda: prevalence and associated factors. PLoS One. 2013; 8(8): e72554.	Total diabetes
Mazzella M, Cotellessa M, Bonassi S, Mulas R, Caratozzolo A, Gaber S, Romano C. Incidence of type I diabetes in the Liguria Region, Italy. Results of a prospective study in a 0- to 14-year age-group. Diabetes Care. 1994; 17(10): 1193-6.	Total diabetes, type 1 diabetes
Mbanya JC, Ngogang J, Salah JN, Minkoulou E, Balkau B. Prevalence of NIDDM and impaired glucose tolerance in a rural and an urban population in Cameroon. Diabetologia. 1997; 40(7): 824-9.	Total diabetes
Meiloud G, Arfa I, Kefi R, Abdelhamid I, Veten F, Lasram K, Ben Halim N, Sidi Mhamed A, Samb A, Abdelhak S, Houmeida AO. Type 2 diabetes in Mauritania: prevalence of the undiagnosed diabetes, influence of family history and maternal effect. Prim Care Diabetes. 2013; 7(1): 19-24.	Total diabetes
Meisinger C, Strassburger K, Heier M, Thorand B, Baumeister SE, Giani G, Rathmann W. Prevalence of undiagnosed diabetes and impaired glucose regulation in 35-59-year-old individuals in Southern Germany: the KORA F4 Study. Diabet Med. 2010; 27(3): 360–2.	Total diabetes
Melander O, Maisel AS, Almgren P, Manjer J, Belting M, Hedblad B, Engstrom G, Kilger U, Nilsson P, Bergmann A, Orho-Melander M. Plasma proneurotensin and incidence of diabetes, cardiovascular disease, breast cancer, and mortality. JAMA. 2012; 308(14): 1469–75.	Total diabetes
Menotti A, Lanti M, Angeletti M, Panarelli W, Scavizzi P, Botta G, Cirillo M, Laurenzi M, Mancini M, Terradura-Vagnarelli O, Zanchetti A. Twenty-year cardiovascular and all-cause mortality trends and changes in cardiovascular risk factors in Gubbio, Italy: The role of blood pressure changes. J Hypertens. 2009; 27(2): 266-74.	Total diabetes
Menzies Research Institute Tasmania, Ministry of Health (Vietnam). Vietnam STEPS Noncommunicable Disease Risk Factors Survey 2009.	Total diabetes
Metcalfe MA, Baum JD. Incidence of insulin dependent diabetes in children aged under 15 years in the British Isles during 1988. BMJ. 1991; 302(6774): 443-7.	Total diabetes, type 1 diabetes
Metelskaya VA, Shkolnikova MA, Shalnova SA, Andreev EM, Deev AD, Jdanov DA, Shkolnikov VM, Vaupel JW. Prevalence, components, and correlates of metabolic syndrome (MetS) among elderly Muscovites. Arch Gerontol Geriatr. 2012; 55(2): 231–7.	Total diabetes
Michalkov DM, Cernay J, Dankov A, Rusn k M, Fand kov K. Incidence and prevalence of childhood diabetes in Slovakia (1985-1992). Slovak Childhood Diabetes Epidemiology Study Group. Diabetes Care. 1995; 18(3): 315-20.	Total diabetes, type 1 diabetes
Miettola J, Nykanen I, Kumpusalo E. Health views and metabolic syndrome in a Finnish rural community: a cross-sectional population study. Can J Rural Med. 2012; 17(1): 10–6.	Total diabetes
Mihardja L, Delima, Manz HS, Ghani L, Soegondo S. Prevalence and determinants of diabetes mellitus and impaired glucose tolerance in Indonesia (a part of basic health research/Riskesdas). Acta Med Indones. 2009; 41(4): 169–74.	Total diabetes
Miller GJ, Maude GH, Beckles GL. Incidence of hypertension and non-insulin dependent diabetes mellitus and associated risk factors in a rapidly developing Caribbean community: the St James survey, Trinidad. J Epidemiol Community Health. 1996; 50(5): 497-504.	Total diabetes
Ministry of Health (Barbados). Barbados STEPS Noncommunicable Disease Risk Factors Survey 2007.	Total diabetes
Ministry of Health (Benin), World Health Organization (WHO). Benin - Littoral STEPS Noncommunicable Disease Risk Factors Survey 2007.	Total diabetes
Ministry of Health (Benin), World Health Organization (WHO). Benin STEPS Noncommunicable Disease Risk Factors Survey 2008.	Total diabetes
Ministry of Health (Benin), World Health Organization (WHO). Benin STEPS Noncommunicable Disease Risk Factors Survey 2015.	Total diabetes
Ministry of Health (Bhutan), World Health Organization (WHO). Bhutan - Thimphu STEPS Noncommunicable Disease Risk Factors Survey 2007.	Total diabetes
Ministry of Health (Bhutan), World Health Organization (WHO). Bhutan STEPS Noncommunicable Disease Risk Factors Survey 2014.	Total diabetes
Ministry of Health (Bhutan), World Health Organization (WHO). Bhutan STEPS Noncommunicable Disease Risk Factors Survey 2019. WHO NCD Microdata Repository, 2021.	Total diabetes
Ministry of Health (Botswana), World Health Organization (WHO). Botswana STEPS Noncommunicable Disease Risk Factors Survey 2014.	Total diabetes
Ministry of Health (Burkina Faso), West African Health Organization, World Health Organization (WHO). Burkina Faso STEPS Noncommunicable Disease Risk Factors Survey 2013.	Total diabetes
Ministry of Health (Cambodia), University of Health Sciences (Cambodia), World Health Organization (WHO). Cambodia STEPS Noncommunicable Disease Risk Factors Survey 2010.	Total diabetes
Ministry of Health (Congo, Rep.), World Health Organization (WHO). Congo - Brazzaville STEPS Noncommunicable Disease Risk Factors Survey 2004.	Total diabetes
Ministry of Health (Cook Islands), World Health Organization (WHO). Cook Islands STEPS Noncommunicable Disease Risk Factors Survey 2013-2015.	Total diabetes
Ministry of Health (Guyana), World Health Organization (WHO). Guyana STEPS Noncommunicable Disease Risk Factors Survey 2016.	Total diabetes
Ministry of Health (Indonesia), World Health Organization (WHO). Indonesia - Jawa Barat STEPS Noncommunicable Disease Risk Factors Survey 2003.	Total diabetes
Ministry of Health (Indonesia), World Health Organization (WHO). Indonesia - Jawa Barat STEPS Noncommunicable Disease Risk Factors Survey 2006.	Total diabetes
Ministry of Health (Indonesia), World Health Organization (WHO). Indonesia STEPS Noncommunicable Disease Risk Factors Survey 2001.	Total diabetes
Ministry of Health (Iraq), Ministry of Planning (Iraq), World Health Organization (WHO). Iraq STEPS Noncommunicable Disease Risk Factors Survey 2015.	Total diabetes
Ministry of Health (Italy), National Association of Hospital Cardiologists (Italy), National Center for Epidemiology, Surveillance, and Health Promotion (Italy), National Institute of Health (Italy). Italy Cardiovascular Epidemiological Observatory/ Health Examination Survey 2008-2012.	Total diabetes

Ministry of Health (Kuwait), World Health Organization (WHO). Kuwait STEPS Noncommunicable Disease Risk Factors Survey 2006.	Total diabetes
Ministry of Health (Kuwait), World Health Organization (WHO). Kuwait STEPS Noncommunicable Disease Risk Factors Survey 2014.	Total diabetes
Ministry of Health (Kyrgyzstan), World Health Organization (WHO). Kyrgyzstan STEPS Noncommunicable Disease Risk Factors Survey 2013.	Total diabetes
Ministry of Health (Malawi), World Health Organization (WHO). Malawi STEPS Noncommunicable Disease Risk Factors Survey 2009.	Total diabetes
Ministry of Health (Malaysia), World Health Organization (WHO). Malaysia STEPS Noncommunicable Disease Risk Factors Survey 2005-2006.	Total diabetes
Ministry of Health (Mauritania), World Health Organization (WHO). Mauritania - Nouakchott STEPS Noncommunicable Disease Risk Factors Survey 2006.	Total diabetes
Ministry of Health (Mexico), National Institute of Public Health (Mexico). Mexico National Survey of Health and Nutrition Mid-way 2016.	Total diabetes
Ministry of Health (Moldova), National Bureau of Statistics (Moldova), National Center of Public Health (Moldova), World Health Organization (WHO). Moldova STEPS Noncommunicable Disease Risk Factors Survey 2013.	Total diabetes
Ministry of Health (Morocco), World Health Organization (WHO). Morocco STEPS Noncommunicable Disease Risk Factors Survey 2017.	Total diabetes
Ministry of Health (Mozambique), World Health Organization (WHO). Mozambique STEPS Noncommunicable Disease Risk Factors Survey 2005.	Total diabetes
Ministry of Health (Myanmar), Myanmar Medical Association, World Health Organization (WHO). Myanmar STEPS Noncommunicable Disease Risk Factors Survey 2014. Geneva, Switzerland: World Health Organization (WHO).	Total diabetes
Ministry of Health (Myanmar), World Health Organization (WHO). Myanmar - Yangon STEPS Noncommunicable Disease Risk Factors Survey 2003.	Total diabetes
Ministry of Health (Nauru), World Health Organization (WHO). Nauru STEPS Noncommunicable Disease Risk Factors Survey 2015-2016. Geneva, Switzerland: World Health Organization (WHO).	Total diabetes
Ministry of Health (New Zealand). New Zealand Virtual Diabetes Register 2005.	Total diabetes
Ministry of Health (New Zealand). New Zealand Virtual Diabetes Register 2006.	Total diabetes
Ministry of Health (New Zealand). New Zealand Virtual Diabetes Register 2007.	Total diabetes
Ministry of Health (New Zealand). New Zealand Virtual Diabetes Register 2008.	Total diabetes
Ministry of Health (New Zealand). New Zealand Virtual Diabetes Register 2009.	Total diabetes
Ministry of Health (New Zealand). New Zealand Virtual Diabetes Register 2010.	Total diabetes
Ministry of Health (New Zealand). New Zealand Virtual Diabetes Register 2011.	Total diabetes
Ministry of Health (New Zealand). New Zealand Virtual Diabetes Register 2012.	Total diabetes
Ministry of Health (New Zealand). New Zealand Virtual Diabetes Register 2013.	Total diabetes
Ministry of Health (New Zealand). New Zealand Virtual Diabetes Register 2014.	Total diabetes
Ministry of Health (New Zealand). New Zealand Virtual Diabetes Register 2015.	Total diabetes
Ministry of Health (New Zealand). New Zealand Virtual Diabetes Register 2016.	Total diabetes
Ministry of Health (New Zealand). New Zealand Virtual Diabetes Register 2017. Wellington, New Zealand: Ministry of Health (New Zealand), 2018.	Total diabetes
Ministry of Health (New Zealand). New Zealand Virtual Diabetes Register 2018. Wellington, New Zealand: Ministry of Health (New Zealand).	Total diabetes
Ministry of Health (New Zealand). New Zealand Virtual Diabetes Register 2019. Wellington, New Zealand: Ministry of Health (New Zealand).	Total diabetes
Ministry of Health (New Zealand). New Zealand Virtual Diabetes Register 2020. Wellington, New Zealand: Ministry of Health (New Zealand).	Total diabetes
Ministry of Health (Niger), World Health Organization (WHO). Niger STEPS Noncommunicable Disease Risk Factors Survey 2007.	Total diabetes
Ministry of Health (Oman), World Health Organization (WHO). Oman STEPS Noncommunicable Disease Risk Factors Survey 2017.	Total diabetes
Ministry of Health (Oman), World Health Organization (WHO). Oman World Health Survey 2007-2008.	Total diabetes
Ministry of Health (Palau), World Health Organization (WHO). Palau STEPS Noncommunicable Disease Risk Factors Survey 2011-2013. Geneva, Switzerland: World Health Organization (WHO).	Total diabetes
Ministry of Health (Palau), World Health Organization (WHO). Palau STEPS Noncommunicable Disease Risk Factors Survey 2016. Geneva, Switzerland: World Health Organization (WHO).	Total diabetes
Ministry of Health (Palestine), World Health Organization (WHO). Palestine STEPS Noncommunicable Disease Risk Factors Survey 2010-2011. Geneva, Switzerland: World Health Organization (WHO).	Total diabetes
Ministry of Health (Peru), National Center for Food and Nutrition, National Institute of Health (Peru), National Institute of Health (Peru). Peru National Survey on Nutritional, Biochemical, Socioeconomic Indicators Related to Chronic Degenerative Diseases 2004-2005.	Total diabetes
Ministry of Health (Saint Lucia), World Health Organization (WHO). Saint Lucia STEPS Noncommunicable Disease Risk Factors Survey 2012.	Total diabetes
Ministry of Health (Samoa). Samoa STEPS Noncommunicable Disease Risk Factors Survey 2002.	Total diabetes
Ministry of Health (Sao Tome and Principe), World Health Organization (WHO). São Tomé and Príncipe STEPS Noncommunicable Disease Risk Factors Survey 2019. WHO NCD Microdata Repository, 2022.	Total diabetes
Ministry of Health (Singapore). Singapore National Health Survey 1992.	Total diabetes

Ministry of Health (Singapore). Singapore National Health Survey 1998.	Total diabetes
Ministry of Health (Singapore). Singapore National Health Survey 2004.	Total diabetes
Ministry of Health (Singapore). Singapore National Health Survey 2010.	Total diabetes
Ministry of Health (Singapore). Singapore National Population Health Survey 2016-2017. Singapore, Singapore: Ministry of Health (Singapore).	Total diabetes
Ministry of Health (Solomon Islands), World Health Organization (WHO). Solomon Islands STEPS Noncommunicable Disease Risk Factors Survey 2015.	Total diabetes
Ministry of Health (Swaziland), World Health Organization (WHO). Swaziland STEPS Noncommunicable Disease Risk Factors Survey 2007.	Total diabetes
Ministry of Health (Swaziland), World Health Organization (WHO). Swaziland STEPS Noncommunicable Disease Risk Factors Survey 2014.	Total diabetes
Ministry of Health (Tajikistan), World Health Organization (WHO). Tajikistan STEPS Noncommunicable Disease Risk Factors Survey 2016.	Total diabetes
Ministry of Health (Timor-Leste), National University of East Timor, World Health Organization (WHO). Timor-Leste STEPS Noncommunicable Disease Risk Factors Survey 2014.	Total diabetes
Ministry of Health (Togo), West African Health Organization, World Health Organization (WHO). Togo STEPS Noncommunicable Disease Risk Factors Survey 2010-2011.	Total diabetes
Ministry of Health (Tonga), World Health Organization (WHO). Tonga STEPS Noncommunicable Disease Risk Factors Survey 2004.	Total diabetes
Ministry of Health (Tonga), World Health Organization (WHO). Tonga STEPS Noncommunicable Disease Risk Factors Survey 2011-2012. 2014.	Total diabetes
Ministry of Health (Turkey). Turkey Chronic Diseases and Risk Factors Study 2011.	Total diabetes
Ministry of Health (Tuvalu), World Health Organization (WHO). Tuvalu STEPS Noncommunicable Disease Risk Factors Survey 2015. Geneva, Switzerland: World Health Organization (WHO), 2020.	Total diabetes
Ministry of Health (Uganda), World Health Organization (WHO). Uganda STEPS Noncommunicable Disease Risk Factors Survey 2014. Geneva, Switzerland: World Health Organization (WHO).	Total diabetes
Ministry of Health (Uzbekistan), World Bank, World Health Organization (WHO). Uzbekistan STEPS Noncommunicable Disease Risk Factors Survey 2014.	Total diabetes
Ministry of Health (Vanuatu), World Health Organization (WHO). Vanuatu STEPS Noncommunicable Disease Risk Factors Survey 2011.	Total diabetes
Ministry of Health (Vietnam). Vietnam STEPS Noncommunicable Disease Risk Factors Survey 2015. Geneva, Switzerland: World Health Organization (WHO), 2016.	Total diabetes
Ministry of Health (Zambia), World Health Organization (WHO). Zambia - Lusaka STEPS Noncommunicable Disease Risk Factors Survey 2008.	Total diabetes
Ministry of Health (Zanzibar), World Health Organization (WHO). Tanzania - Zanzibar STEPS Noncommunicable Disease Risk Factors Survey 2011.	Total diabetes
Ministry of Health and Family Welfare (Bangladesh), National Institute of Preventive and Social Medicine, University of Dhaka (Bangladesh), World Health Organization (WHO). Bangladesh STEPS Noncommunicable Disease Risk Factors Survey 2018. Geneva, Switzerland: World Health Organization (WHO).	Total diabetes
Ministry of Health and Human Services (Marshall Islands), World Health Organization (WHO). Marshall Islands STEPS Noncommunicable Disease Risk Factors Survey 2017-2018 . Geneva, Switzerland: World Health Organization (WHO), 2020.	Total diabetes
Ministry of Health and Medical Education (Iran). Iran STEPS Noncommunicable Disease Risk Factors Survey 2011.	Total diabetes
Ministry of Health and Medical Industry (Turkmenistan), State Committee on Statistics of Turkmenistan, World Health Organization (WHO). Turkmenistan STEPS Noncommunicable Disease Risk Factors Survey 2018. WHO NCD Microdata Repository, 2021.	Total diabetes
Ministry of Health and Medical Services (Kiribati), World Health Organization (WHO). Kiribati STEPS Noncommunicable Disease Risk Factors Survey 2004-2006.	Total diabetes
Ministry of Health and Medical Services (Kiribati), World Health Organization (WHO). Kiribati STEPS Noncommunicable Disease Risk Factors Survey 2016.	Total diabetes
Ministry of Health and Population (Egypt), United States Agency for International Development (USAID), World Health Organization (WHO). Egypt STEPS Noncommunicable Disease Risk Factors Survey 2011-2012.	Total diabetes
Ministry of Health and Population (Nepal), Nepal Health Research Council (NHRC), World Health Organization (WHO). Nepal STEPS Noncommunicable Disease Risk Factors Survey 2012-2013.	Total diabetes
Ministry of Health and Population (Nepal), Nepal Health Research Council (NHRC), World Health Organization (WHO). Nepal STEPS Noncommunicable Disease Risk Factors Survey 2019. Geneva, Switzerland: World Health Organization (WHO).	Total diabetes
Ministry of Health and Social Protection (Colombia), National Consulting Center (Colombia), University of Caldas (Colombia), University of Valle (Colombia). Colombia Health, Well-Being and Aging Survey 2015.	Total diabetes
Ministry of Health and Social Welfare (Lesotho), World Health Organization (WHO). Lesotho STEPS Noncommunicable Disease Risk Factors Survey 2012 .	Total diabetes
Ministry of Health and Welfare (Japan), University of Tokyo. Japan National Health and Nutrition Survey Diabetes Status by Age, Sex, and Prefecture 1990-2017.	Total diabetes
Ministry of Health and Welfare (Taiwan). Taiwan National Health Insurance Claims Data 2016.	Total diabetes, type 1 diabetes
Ministry of Health, Population and Hospital Reform (Algeria), World Health Organization (WHO). Algeria - Sétif and Mostaganem STEPS Noncommunicable Disease Risk Factors Survey 2003.	Total diabetes
Ministry of Health, Population and Hospital Reform (Algeria), World Health Organization (WHO). Algeria STEPS Noncommunicable Disease Risk Factors Survey 2016-2017.	Total diabetes
Ministry of Public Health (Afghanistan), World Health Organization (WHO). Afghanistan STEPS Noncommunicable Disease Risk Factors Survey 2018. Geneva, Switzerland: World Health Organization (WHO).	Total diabetes
Ministry of Public Health (Congo, DR), World Health Organization (WHO). Democratic Republic of the Congo - Kinshasa STEPS Noncommunicable Disease Risk Factors Survey 2005. 2021.	Total diabetes

Ministry of Public Health (Ecuador), National Institute of Statistics and Censuses (Ecuador). Ecuador National Health and Nutrition Survey 2012.	Total diabetes
Ministry of Public Health (Ecuador), World Health Organization (WHO). Ecuador STEPS Noncommunicable Disease Risk Factors Survey 2018. Geneva, Switzerland: World Health Organization (WHO).	Total diabetes
Ministry of Public Health (Lebanon), World Health Organization (WHO). Lebanon STEPS Noncommunicable Disease Risk Factors Survey 2016-2017.	Total diabetes
Ministry of Public Health (Uruguay), World Health Organization (WHO). Uruguay STEPS Noncommunicable Disease Risk Factors Survey 2006.	Total diabetes
Miranda AM, Steluti J, Fisberg RM, Marchioni DM. Association between Coffee Consumption and Its Polyphenols with Cardiovascular Risk Factors: A Population-Based Study. Nutrients. 2017; 9(3).	Total diabetes
Modesti PA, Bamoshmoosh M, Rapi S, Massetti L, Bianchi S, Al-Hidabi D, Al Goshae H. Relationship between hypertension, diabetes and proteinuria in rural and urban households in Yemen. J Hum Hypertens. 2013; 27(9): 572-9.	Total diabetes
Moon H-W, Park CM, Hong SN, Park S, Hur M, Yun Y-M. Assessment of apoB dyslipoproteinemia in Korean population. Clin Biochem. 2013; 46(12): 1041–6.	Total diabetes
Morales DD, Punzalan FER, Paz-Pacheco E, Sy RG, Duante CA. Metabolic syndrome in the Philippine general population: prevalence and risk for atherosclerotic cardiovascular disease and diabetes mellitus. Diab Vasc Dis Res. 2008; 5(1): 36-43.	Total diabetes
Morales-Pérez FM, Barquero-Romero J, Pérez-Miranda M. Incidence of type I diabetes among children and young adults (0-29 years) in the province of Badajoz, Spain during 1992 to 1996. Acta Paediatr. 2000; 89(1): 101-4.	Total diabetes, type 1 diabetes
Motala AA, Esterhuizen T, Gouws E, Pirie FJ, Omar MA. Diabetes and other disorders of glycemia in a rural South African community: prevalence and associated risk factors. Diabetes Care. 2008; 31(9): 1783-8.	Total diabetes
Motta M, Bennati E, Capri M, Ferlito L, Malaguarnera M. Diabetes mellitus in the extreme longevity. Exp Gerontol. 2008; 43(2): 102-5.	Total diabetes
Moussa MA, Alsaeid M, Abdella N, Refai TM, Al-Sheikh N, Gomez JE. Prevalence of type 1 diabetes among 6- to 18-year-old Kuwaiti children. Med Princ Pract. 2005; 14(2): 87-91.	Total diabetes
Muiña PG, Herrera MJ, Atance EP, Donado JJ, Sánchez G, Ferrer LS. [Epidemiological study of type 1 diabetes in children under 15 years-old in Castilla-La Mancha (Spain)]. An Pediatr (Barc). 2012; 76(2): 83-91.	Total diabetes, type 1 diabetes
Muiesan ML, Padovani A, Salvetti M, Monteduro C, Poisa P, Bonzi B, Paini A, Cottini E, Agosti C, Castellano M, Rizzoni D, Vignolo A, Agabiti-Rosei E. Headache: Prevalence and relationship with office or ambulatory blood pressure in a general population sample (the Vobarno Study). Blood Press. 2006; 15(1): 14-9.	Total diabetes
Muniz J, Hervada J, Juane R, Lopez-Rodriguez I, Castro-Beiras A. Prevalence of diabetes mellitus in the population aged 40-69 years in Galicia, northwest Spain. Diabetes Res Clin Pract. 1995; 30(2): 137-42.	Total diabetes
Murdock D, Salit J, Stoffel M, Friedman JM, Pe'er I, Breslow JL, Bonnen PE. Longitudinal study shows increasing obesity and hyperglycemia in micronesia. Obesity (Silver Spring). 2013; 21(9): E421–7.	Total diabetes
Muyer MT, Muls E, Mapatano MA, Makulo JR, Mvitu M, Kimenyembo W, Mandja BA, Kimbondo P, Bieleli CB, Kaimbo Wa Kaimbo D, Buntinx F. Diabetes and intermediate hyperglycaemia in Kisantu, DR Congo: a cross-sectional prevalence study. BMJ Open. 2012; 2(6): nan.	Total diabetes
Mykkänen L, Laakso M, Uusitupa M, Pyörälä K. Prevalence of diabetes and impaired glucose tolerance in elderly subjects and their association with obesity and family history of diabetes. Diabetes Care. 1990; 13(11): 1099-105.	Total diabetes
Nakagami T, Tominaga M, Nishimura R, Yoshiike N, Daimon M, Oizumi T, Tajima N. Is the measurement of glycated hemoglobin A1c alone an efficient screening test for undiagnosed diabetes? Japan National Diabetes Survey. Diabetes Res Clin Pract. 2007; 76(2): 251-6.	Total diabetes
Nangia V, Jonas JB, Sinha A, Matin A, Kulkarni M, Panda-Jonas S. Ocular axial length and its associations in an adult population of Central Rural India. The Central India Eye and Medical Study [Unpublished data]. Ophthalmology 2010;117(7):1360-6.	Total diabetes
Napoli N, Mottini G, Arigliani M, Creta A, Giua R, Incammisa A, Carotti S, Sihom F, Yimagou I, Alombah R, Mbanya JC, Pozzilli P. Unexpectedly high rates of obesity and dysglycemia among villagers in Cameroon. Diabetes Metab Res Rev. 2010; 26(1): 10–12.	Total diabetes
NatCen Social Research and Royal Free and University College Medical School. Department of Epidemiology and Public Health, Health Survey for England, 2010 [computer file]. 2nd Edition. Colchester, Essex: UK Data Archive [distributor], July 2012. SN: 6986, http://dx.doi.org/10.5255/UKDA-SN-6986-2	Total diabetes
NatCen Social Research and University College London. Department of Epidemiology and Public Health, Health Survey for England, 2011 [computer file]. Colchester, Essex: UK Data Archive [distributor], April 2013. SN: 7260, http://dx.doi.org/10.5255/UKDA-SN-7260-1	Total diabetes
NatCen Social Research and University College London. Department of Epidemiology and Public Health, Health Survey for England, 2012 [computer file]. Colchester, Essex: UK Data Archive [distributor], April 2014. SN: 7480, http://dx.doi.org/10.5255/UKDA-SN-7480-1	Total diabetes
NatCen Social Research and University College London. Department of Epidemiology and Public Health, Health Survey for England, 2013 [computer file]. Colchester, Essex: UK Data Archive [distributor], January 2015. SN: 7649, http://dx.doi.org/10.5255/UKDA-SN-7649-1	Total diabetes
National Center for Disease Control and Public Health (Georgia). Georgia STEPS Noncommunicable Disease Risk Factors Survey 2016.	Total diabetes
National Center for Health Statistics (NCHS), Centers for Disease Control and Prevention (CDC). United States National Health and Nutrition Examination Survey 1976-1980. Hyattsville, United States: National Center for Health Statistics (NCHS), Centers for Disease Control and Prevention (CDC).	Total diabetes
National Center for Health Statistics (NCHS), Centers for Disease Control and Prevention (CDC). United States National Health and Nutrition Examination Survey 1988-1994. Hyattsville, United States: National Center for Health Statistics (NCHS), Centers for Disease Control and Prevention (CDC).	Total diabetes
National Center for Health Statistics (NCHS), Centers for Disease Control and Prevention (CDC). United States National Health and Nutrition Examination Survey 1999-2000. Hyattsville, United States: National Center for Health Statistics (NCHS), Centers for Disease Control and Prevention (CDC).	Total diabetes
National Center for Health Statistics (NCHS), Centers for Disease Control and Prevention (CDC). United States National Health and Nutrition Examination Survey 2001-2002. Hyattsville, United States: National Center for Health Statistics (NCHS), Centers for Disease Control and Prevention (CDC).	Total diabetes
National Center for Health Statistics (NCHS), Centers for Disease Control and Prevention (CDC). United States National Health and Nutrition Examination Survey 2003-2004. Hyattsville, United States: National Center for Health Statistics (NCHS), Centers for Disease Control and Prevention (CDC).	Total diabetes
National Center for Health Statistics (NCHS), Centers for Disease Control and Prevention (CDC). United States National Health and Nutrition Examination Survey 2005-2006. Hyattsville, United States: National Center for Health Statistics (NCHS), Centers for Disease Control and Prevention (CDC), 2007.	Total diabetes

National Center for Health Statistics (NCHS), Centers for Disease Control and Prevention (CDC). United States National Health and Nutrition Examination Survey 2007-2008. Hyattsville, United States: National Center for Health Statistics (NCHS), Centers for Disease Control and Prevention (CDC), 2009.	Total diabetes
National Center for Health Statistics (NCHS), Centers for Disease Control and Prevention (CDC). United States National Health and Nutrition Examination Survey 2009-2010. Hyattsville, United States: National Center for Health Statistics (NCHS), Centers for Disease Control and Prevention (CDC), 2011.	Total diabetes
National Center for Health Statistics, Centers for Disease Control and Prevention. United States National Health and Nutrition Examination Survey 2011-2012. Hyattsville, United States of America: National Center for Health Statistics, Centers for Disease Control and Prevention, 2013.	Total diabetes
National Center for Health Statistics, Centers for Disease Control and Prevention. United States National Health and Nutrition Examination Survey 2013-2014. Hyattsville, United States of America: National Center for Health Statistics, Centers for Disease Control and Prevention.	Total diabetes
National Center for Health Statistics, Centers for Disease Control and Prevention. United States National Health and Nutrition Examination Survey 2015-2016. Hyattsville, United States of America: National Center for Health Statistics, Centers for Disease Control and Prevention, 2017.	Total diabetes
National Center for Health Statistics, Centers for Disease Control and Prevention. United States National Health and Nutrition Examination Survey 2017-2018. Hyattsville, United States of America: National Center for Health Statistics, Centers for Disease Control and Prevention.	Total diabetes
National Centre for Social Research and University College London. Department of Epidemiology and Public Health, Health Survey for England, 2003 [computer file]. Colchester, Essex: UK Data Archive [distributor], March 2005. SN: 5098.	Total diabetes
National Centre for Social Research and University College London. Department of Epidemiology and Public Health, Health Survey for England, 2006 [computer file]. 4th Edition. Colchester, Essex: UK Data Archive [distributor], July 2011. SN: 5809, http://dx.doi.org/10.5255/UKDA-SN-5809-1	Total diabetes
National Centre for Social Research and University College London. Department of Epidemiology and Public Health, Health Survey for England, 2008 [computer file]. 3rd Edition. Colchester, Essex: UK Data Archive [distributor], July 2011. SN: 6397, http://dx.doi.org/10.5255/UKDA-SN-6397-1	Total diabetes
National Centre for Social Research and University College London. Department of Epidemiology and Public Health, Health Survey for England, 2009 [computer file]. 2nd Edition. Colchester, Essex: UK Data Archive [distributor], July 2011. SN: 6732, http://dx.doi.org/10.5255/UKDA-SN-6732-1	Total diabetes
National Centre for Social Research and University College London. Department of Epidemiology and Public Health, Health Survey for England, 2005 [computer file]. Colchester, Essex: UK Data Archive [distributor], July 2007. SN: 5675.	Total diabetes
National Health Examination Survey Office (Thailand). Thailand National Health and Examination Survey 2008-2009.	Total diabetes
National Institute for Medical Research (Tanzania), World Health Organization (WHO). Tanzania STEPS Noncommunicable Disease Risk Factors Survey 2012.	Total diabetes
National Institute of Public Health (Mexico), National Institute of Statistics and Geography (INEGI) (Mexico). Mexico National Survey of Health and Nutrition (ENSANUT) 2018-2019. Cuernavaca, Mexico: National Institute of Public Health (Mexico).	Total diabetes
National Institute of Public Health (Mexico). Mexico National Health Survey 1999-2000.	Total diabetes
National Institute of Public Health (Mexico). Mexico National Survey of Health and Nutrition 2005-2006. Cuernavaca, Mexico: National Institute of Public Health (Mexico).	Total diabetes
National Institute of Public Health (Mexico). Mexico National Survey of Health and Nutrition 2011-2012. Cuernavaca, Mexico: National Institute of Public Health (Mexico).	Total diabetes
Nazir A, Papita R, Anbalagan VP, Anjana RM, Deepa M, Mohan V. Prevalence of diabetes in Asian Indians based on glycated hemoglobin and fasting and 2-H post-load (75-g) plasma glucose (CURES-120). Diabetes Technol Ther. 2012; 14(8): 665–8.	Total diabetes
Negrato CA, Dias JPL, Teixeira MF, Dias A, Salgado MH, Lauris JR, Montenegro RM Jr, Gomes MB, Jovanovic L. Temporal trends in incidence of Type 1 diabetes between 1986 and 2006 in Brazil. J Endocrinol Invest. 2010; 33(6): 373-7.	Total diabetes, type 1 diabetes
Newhook LA, Grant M, Sloka S, Hoque M, Paterson AD, Hagerty D, Curtis J. Very high and increasing incidence of type 1 diabetes mellitus in Newfoundland and Labrador, Canada. Pediatr Diabetes. 2008; 9(3 Pt 2): 62-8.	Total diabetes, type 1 diabetes
Newhook LA, Penney S, Fiander J, Dowden J. Recent incidence of type 1 diabetes mellitus in children 0-14 years in Newfoundland and Labrador, Canada climbs to over 45/100,000: a retrospective time trend study. BMC Res Notes. 2012; 628.	Total diabetes, type 1 diabetes
Nilsson PM, Møller L, Solstad K. Adverse effects of psychosocial stress on gonadal function and insulin levels in middle-aged males. J Intern Med. 1995; 237(5): 479-86.	Total diabetes
Nirmalan PK, Tielsch JM, Katz J, Thulasiraj RD, Krishnadas R, Ramakrishnan R, Robin AL. Relationship between vision impairment and eye disease to vision-specific quality of life and function in rural India: the Aravind Comprehensive Eye Survey. Invest Ophthalmol Vis Sci. 2005; 46(7): 2308-12.	Total diabetes
Norwegian Institute of Public Health, Tromsø Municipal Council (Norway), University Hospital of North Norway, University of Tromsø - The Arctic University of Norway. Norway Tromso Study 1994-2016.	Total diabetes
Norwegian Institute of Public Health, University of Bergen, University of Oslo, University of Tromsø - The Arctic University of Norway, University of Trondheim (Norway). Cohort of Norway 1994-2003.	Total diabetes
Norwegian University of Science and Technology (NTNU). Norway - Nord-Trøndelag Health Study Wave 3 2006-2008. Trondheim, Norway: Norwegian University of Science and Technology (NTNU).	Total diabetes
Nyenwe EA, Odia OJ, Ihekwaba AE, Ojule A, Babatunde S. Type 2 diabetes in adult Nigerians: a study of its prevalence and risk factors in Port Harcourt, Nigeria. Diabetes Res Clin Pract. 2003; 62(3): 177-85.	Total diabetes
Nystrom L, Dahlquist G, Rewers M, Wall S. The Swedish childhood diabetes study. An analysis of the temporal variation in diabetes incidence 1978-1987. Int J Epidemiol. 1990; 19(1): 141-6.	Total diabetes, type 1 diabetes
O Connor JM, Millar SR, Buckley CM, Kearney PM, Perry IJ. The prevalence and determinants of undiagnosed and diagnosed type 2 diabetes in middle-aged irish adults. PLoS One. 2013; 8(11): e80504.	Total diabetes
Office of Population Censuses and Surveys. Social Survey Division, Health Survey for England, 1993 [Computer file]. Colchester, Essex: UK Data Archive [distributor], April 1995. SN: 3316, http://dx.doi.org/10.5255/UKDA-SN-3316-1	Total diabetes
Ogle GD, Lesley J, Sine P, McMaster P. Type 1 diabetes mellitus in children in Papua New Guinea. P N G Med J. 2001; 44(3-4): 96-100.	Type 1 diabetes
Ohmura T, Ueda K, Kiyohara Y, Kato I, Iwamoto H, Nakayama K, Nomiyama K, Ohmori S, Yoshitake T, Shinkawu A. Prevalence of type 2 (non-insulin-dependent) diabetes mellitus and impaired glucose tolerance in the Japanese general population: the Hisayama Study. Diabetologia. 1993; 36(11): 1198-203.	Total diabetes

Okada K, Furusyo N, Sawayama Y, Kanamoto Y, Murata M, Hayashi J. Prevalence and risk factors for diabetes: a ten year follow-up study of the Yaeyama district of Okinawa. Hukuoka Acta Med. 2010; 101(10): 215-24.	Total diabetes
Okesina AB, Oparinde DP, Akindoyin KA, Erasmus RT. Prevalence of some risk factors of coronary heart disease in a rural Nigerian population. East Afr Med J. 1999; 76(4): 212-6.	Total diabetes
Oladapo OO, Salako L, Sodiq O, Shoyinka K, Adedapo K, Falase AO. A prevalence of cardiometabolic risk factors among a rural Yoruba south-western Nigerian population: a population-based survey. Cardiovasc J Afr. 2010; 21(1): 26–31.	Total diabetes
Omar MA, Seedat MA, Motala AA, Dyer RB, Becker P. The prevalence of diabetes mellitus and impaired glucose tolerance in a group of urban South African blacks. S Afr Med J. 1993; 83(9): 641-3.	Total diabetes
Omboni S, Carabelli G, Ghirardi E, Carugo S. Awareness, treatment, and control of major cardiovascular risk factors in a small-scale Italian community: results of a screening campaign. Vasc Health Risk Manag. 2013; 9: 177–85.	Total diabetes
Onat A, Hergenc G, Bulur S, Ugur M, Kucukdurmaz Z, Can G. The paradox of high apolipoprotein A-I levels independently predicting incident type-2 diabetes among Turks. Int J Cardiol. 2010; 142(1): 72–9.	Total diabetes
Ono K, Limbu YR, Rai SK, Kurokawa M, Yanagida J, Rai G, Gurung N, Sharma M, Rai CK. The prevalence of type 2 diabetes mellitus and impaired fasting glucose in semi-urban population of Nepal. Nepal Med Coll J. 2007; 9(3): 154-6.	Total diabetes
Ostman J, Lonnberg G, Arnqvist HJ, Blohm, G, Bolinder J, Ekbom Schnell A, Eriksson JW, Gudbjornsdottir S, Sundkvist G, Nystrom L. Gender differences and temporal variation in the incidence of type 1 diabetes: results of 8012 cases in the nationwide Diabetes Incidence Study in Sweden 1983-2002. J Intern Med. 2008; 263(4): 386-94.	Type 1 diabetes
Ostovaneh MR, Zamani F, Sharafkhah M, Ansari-Moghaddam A, Akhavan Khaleghi N, Saeedian FS, Rohani Z, Motamed N, Maadi M, Malekzadeh R, Poustchi H. Prevalence of metabolic syndrome in Amol and Zahedan, Iran: a population based study. Arch Iran Med. 2014; 17(7): 477–82.	Total diabetes
Ostrauskas R, Zalinkevicius R, Jurgeviciene N, Radzeviciene L, Lasaite L. The incidence of type 1 diabetes mellitus among 15-34 years aged Lithuanian population: 18-year incidence study based on prospective databases. BMC Public Health. 2011; 813.	Type 1 diabetes
Ostrauskas R, Zalinkevicius R. Incidence in young adulthood-onset Type 1 diabetes mellitus in Lithuania during 1991-1997. Lithuanian Epidemiology Diabetes Study Group. Diabetes Nutr Metab. 2000; 13(2): 68-74.	Type 1 diabetes
Ostrauskas R. The prevalence of type 1 diabetes mellitus among adolescents and adults in Lithuania during 1991-2004. Medicina (Kaunas). 2007; 43(3): 242-50.	Total diabetes
Owoaje EE, Rotimi CN, Kaufman JS, Tracy J, Cooper RS. Prevalence of adult diabetes in Ibadan, Nigeria. East Afr Med J. 1997; 74(5): 299-302.	Total diabetes
Pérez CM, Guzmán M, Ortiz AP, Estrella M, Valle Y, Pérez N, Haddock L, Suárez E. Prevalence of the metabolic syndrome in San Juan, Puerto Rico. Ethn Dis. 2008; 18(4): 434-41.	Total diabetes
Padaiga Z, Tuomilehto J, Karvonen M, Dahlquist G, Podar T, Adojaan B, Urbonaite B, Zalinkevicius R, Brigis G, Virtala E, Kohtam„ki K, Cepaitis Z, Tuomilehto-Wolf E. Seasonal variation in the incidence of Type 1 diabetes mellitus during 1983 to 1992 in the countries around the Baltic Sea. Diabet Med. 1999; 16(9): 736-43.	Total diabetes, type 1 diabetes
Palacios C, Pérez CM, Guzmán M, Ortiz AP, Ayala A, Suárez E. Association between adiposity indices and cardiometabolic risk factors among adults living in Puerto Rico. Public Health Nutr. 2011; 14(10): 1714–23.	Total diabetes
Palomo G I, Icaza N G, Mujica E V, Núñez F L, Leiva M E, Vásquez R M, Alarcón L M, Moyano D E. Prevalencia de factores de riesgo cardiovascular clásicos en población adulta de Talca, Chile, 2005. Rev Med Chil. 2007; 135(7): 904-12.	Total diabetes
Pan American Health Organization (PAHO). Central America Diabetes Initiative (CAMDI): Survey of Diabetes, Hypertension and Chronic Disease Risk Factors. Belize, San José, San Salvador, Guatemala City, Managua and Tegucigalpa. Washington, D.C., United States: Pan American Health Organization (PAHO), 2011.	Total diabetes
Pan American Health Organization (PAHO). Honduras - Tegucigalpa Diabetes, Hypertension, and Non-Communicable Disease Risk Factors Survey 2003-2004.	Total diabetes
Pan W-H, Wu H-J, Yeh C-J, Chuang S-Y, Chang H-Y, Yeh N-H, Hsieh Y-T. Diet and health trends in Taiwan: comparison of two nutrition and health surveys from 1993-1996 and 2005-2008. Asia Pac J Clin Nutr. 2011; 20(2): 238–50.	Total diabetes
Pan X, Yang W, Liu J. Prevalence of diabetes and its risk factors in China 1994. National Diabetes Prevention and Control Cooperative Group. Chin J Intern Med. 1997; 36(6): 384-9.	Total diabetes
Panamonta O, Laopaiboon M, Tuchinda C. Incidence of childhood type 1 (insulin dependent) diabetes mellitus in northeastern Thailand. J Med Assoc Thai. 2000; 83(8): 821-4.	Total diabetes, type 1 diabetes
Papantoniou K, Fito M, Covas M-I, Munoz D, Schroder H. trans Fatty acid consumption, lifestyle and type 2 diabetes prevalence in a Spanish population. Eur J Nutr. 2010; 49(6): 357–64.	Total diabetes
Papazoglou N, Manes C, Chatzimitrofanous P, Papadeli E, Tzounas K, Scaragas G, Kontogiannis I, Alexiades D. Epidemiology of diabetes mellitus in the elderly in northern Greece: a population study. Diabet Med. 1995; 12(5): 397-400.	Total diabetes
Papoz L, Ben Khalifa F, Eschwege E, Ben Ayed H. Diabetes mellitus in Tunisia: description in urban and rural populations. Int J Epidemiol. 1988; 17(2): 419-22.	Total diabetes
Park Y, Lee H, Koh CS, Min H, Yoo K, Kim Y, Shin Y. Prevalence of diabetes and IGT in Yonchon County, South Korea. Diabetes Care. 1995; 18(4): 545-8.	Total diabetes
Park Y, Lee H, Koh CS, Min H. Community-based epidemiologic study on atherosclerotic cardiovascular risk factors. Diabetes Res Clin Pract. 1996; S65-72.	Total diabetes
Passos VM de A, Barreto SM, Diniz LM, Lima-Costa MF. Type 2 diabetes: prevalence and associated factors in a Brazilian community--the Bambuí health and aging study. Sao Paulo Med J. 2005; 123(2): 66-71.	Total diabetes
Patandin S, Bots ML, Abel R, Valkenburg HA. Impaired glucose tolerance and diabetes mellitus in a rural population in south India. Diabetes Res Clin Pract. 1994; 24(1): 47-53.	Total diabetes
Patterson CC, Carson DJ, Hadden DR. Epidemiology of childhood IDDM in Northern Ireland 1989-1994: low incidence in areas with highest population density and most household crowding. Northern Ireland Diabetes Study Group. Diabetologia. 1996; 39(9): 1063-9.	Total diabetes, type 1 diabetes
Patterson CC, Smith PG, Webb J, Heasman MA, Mann JI. Geographical variation in the incidence of diabetes mellitus in Scottish children during the period 1977-1983. Diabet Med. 1988; 5(2): 160-5.	Total diabetes, type 1 diabetes
Pedro JM, Brito M, Barros H. Prevalence, awareness, treatment and control of hypertension, diabetes and hypercholesterolaemia among adults in Dande municipality, Angola. Cardiovasc J Afr. 2017; 28: 1–10.	Total diabetes
Pessinaba S, Mbaye A, Yabeta G-A-D, Kane A, Ndao CT, Ndiaye MB, Harouna H, Bodian M, Diao M, Mbaye MN, Diagne MN, Diack B, Kane M, Niang K, Mathieu J-BS, Kane A. Prevalence and determinants of hypertension and associated cardiovascular risk factors: data from a population-based, cross-sectional survey in Saint Louis, Senegal. Cardiovasc J Afr. 2013; 24(5): 180–3.	Total diabetes

Pinelli L, Beretta F, Dalla Bernardina P, Gonfiantini E, Groff P. Incidence of insulin dependent diabetes mellitus in children 0-14 years old in the Veneto Region, Italy. J Pediatr Endocrinol Metab. 1998; 11(3): 447-50.	Total diabetes, type 1 diabetes
Pishdad GR. Low incidence of type 1 diabetes in Iran. Diabetes Care. 2005; 28(4): 927-8.	Total diabetes, type 1 diabetes
Podar T, Laporte RE. Incidence of childhood diabetes did not increase in Estonia during 1980-89. Diabete Metab. 1993; 19(4): 361-3.	Total diabetes, type 1 diabetes
Podar T, Solntsev A, Karvonen M, Padaiga Z, Brigis G, Urbonaite B, Viik-Kajander M, Reunanen A, Tuomilehto J. Increasing incidence of childhood-onset type I diabetes in 3 Baltic countries and Finland 1983-1998. Diabetologia. 2001; B17-20.	Total diabetes, type 1 diabetes
Prasad DS, Kabir Z, Dash AK, Das BC. Prevalence and risk factors for diabetes and impaired glucose tolerance in Asian Indians: a community survey from urban eastern India. Diabetes Metab Syndr. 2012; 6(2): 96-101.	Total diabetes
Pronina EA, Petraikina EE, Antsiferov MB, Duchareva OV, Petrone A, Buzzetti R, Pozzilli P. A 10-year (1996-2005) prospective study of the incidence of Type 1 diabetes in Moscow in the age group 0-14 years. Diabet Med. 2008; 25(8): 956-9.	Total diabetes, type 1 diabetes
Public Health Institute, Ministry of Health (Mongolia), World Health Organization (WHO). Mongolia STEPS Noncommunicable Disease Risk Factors Survey 2013.	Total diabetes
Public Health Institute, Ministry of Health (Mongolia), World Health Organization (WHO). Mongolia STEPS Noncommunicable Disease Risk Factors Survey 2019. Geneva, Switzerland: World Health Organization (WHO).	Total diabetes
Pubudu De Silva A, Padmal De Silva SH, Liyanage IK, Rajapakse LC, Jayasinghe KSA, Katulanda P, Wijeratne CN, Wijeratne S. Social, cultural and economical determinants of diabetes mellitus in Kalutara district, Sri Lanka: a cross sectional descriptive study. Int J Equity Health. 2012; 11: 76.	Total diabetes
Pundziute-Lycka A, Urbonaite B, Ostrauskas R, Zalinkevicius R, Dahlquist GG. Incidence of type 1 diabetes in Lithuanians aged 0-39 years varies by the urban-rural setting, and the time change differs for men and women during 1991-2000. Diabetes Care. 2003; 26(3): 671-6.	Total diabetes, type 1 diabetes
Pundziute-Lyckå A, Dahlquist G, Urbonaite B, Žalinkevičius R. Time trend of childhood type 1 diabetes incidence in Lithuania and Sweden, 1983-2000. Acta Paediatr. 2004; 93(11): 1519-24.	Total diabetes, type 1 diabetes
Qatar Statistics Authority, Supreme Council of Health (Qatar), World Health Organization (WHO). Qatar STEPS Noncommunicable Disease Risk Factors Survey 2012. Geneva, Switzerland: World Health Organization (WHO).	Total diabetes
Qi L, Feng L, Ding X, Mao D, Wang Y, Xiong H. Prevalence of diabetes and impaired fasting glucose among residents in the Three Gorges Reservoir Region, China. BMC Public Health. 2014; 14: 1152.	Total diabetes
Qin Y, Wang R, Ma X, Zhao Y, Lu J, Wu C, He J. Prevalence, Awareness, Treatment and Control of Diabetes Mellitus-A Population Based Study in Shanghai, China. Int J Environ Res Public Health. 2016; 13(5).	Total diabetes
Quoc PS, Charles MA, Cuong NH, Lieu LH, Tuan NA, Thomas M, Balkau B, Simon D. Blood glucose distribution and prevalence of diabetes in Hanoi (Vietnam). Am J Epidemiol. 1994; 139(7): 713-22.	Total diabetes
Radosevic B, Bukara-Radujkovic G, Miljkovic V, Pejicic S, Bratina N, Battelino T. The incidence of type 1 diabetes in Republic of Srpska (Bosnia and Herzegovina) and Slovenia in the period 1998-2010. Pediatr Diabetes. 2013; 14(4): 273-9.	Total diabetes, type 1 diabetes
Rahim MA, Azad Khan AK, Nahar Q, Ali SMK, Hussain A. Impaired fasting glucose and impaired glucose tolerance in rural population of Bangladesh. Bangladesh Med Res Counc Bull. 2010; 36(2): 47–51.	Total diabetes
Rahim MA, Hussain A, Azad Khan AK, Sayeed MA, Keramat Ali SM, Vaaler S. Rising prevalence of type 2 diabetes in rural Bangladesh: a population based study. Diabetes Res Clin Pract. 2007; 77(2): 300-5.	Total diabetes
Rahman MM, Rahim MA, Nahar Q. Prevalence and risk factors of type 2 diabetes in an urbanizing rural community of Bangladesh. Bangladesh Med Res Counc Bull. 2007; 33(2): 48-54.	Total diabetes
Raiko JRH, Viikari JSA, Ilmanen A, Hutri-Kahonen N, Taittonen L, Jokinen E, Pietikainen M, Jula A, Loo B-M, Marniemi J, Lehtimaki T, Kahonen M, Ronnemaa T, Raitakari OT, Juonala M. Follow-ups of the Cardiovascular Risk in Young Finns Study in 2001 and 2007: levels and 6-year changes in risk factors. J Intern Med. 2010; 267(4): 370–84.	Total diabetes, type 1 diabetes
Ramachandran A, Mary S, Yamuna A, Murugesan N, Snehalatha C. High prevalence of diabetes and cardiovascular risk factors associated with urbanization in India. Diabetes Care. 2008; 31(5): 893-8.	Total diabetes
Ramachandran A, Snehalatha C, Krishnaswamy CV. Incidence of IDDM in children in urban population in southern India. Madras IDDM Registry Group Madras, South India. Diabetes Res Clin Pract. 1996; 34(2): 79–82.	Total diabetes, type 1 diabetes
Ramachandran A, Snehalatha C, Latha E, Manoharan M, Vijay V. Impacts of urbanisation on the lifestyle and on the prevalence of diabetes in native Asian Indian population. Diabetes Res Clin Pract. 1999; 44(3): 207-13.	Total diabetes
Raman Kutty V, Joseph A, Soman CR. High prevalence of type 2 diabetes in an urban settlement in Kerala, India. Ethn Health. 1999; 4(4): 231-9.	Total diabetes
Rami B, Waldhor T, Schober E. Incidence of Type I diabetes mellitus in children and young adults in the province of Upper Austria, 1994-1996. Diabetologia. 2001; B45-7.	Total diabetes, type 1 diabetes
Rampal S, Mahadeva S, Guallar E, Bulgiba A, Mohamed R, Rahmat R, Arif MT, Rampal L. Ethnic differences in the prevalence of metabolic syndrome: results from a multi-ethnic population-based survey in Malaysia. PLoS One. 2012; 7(9): e46365.	Total diabetes
Rampal S, Rampal L, Rahmat R, Zain AM, Yap YG, Mohamed M, Taha M. Variation in the prevalence, awareness, and control of diabetes in a multiethnic population: a nationwide population study in Malaysia. Asia Pac J Public Health. 2010; 22(2): 194-202.	Total diabetes
Rangasami JJ, Greenwood DC, McSporran B, Smail PJ, Patterson CC, Waugh NR. Rising incidence of type 1 diabetes in Scottish children, 1984-93. The Scottish Study Group for the Care of Young Diabetics. Arch Dis Child. 1997; 77(3): 210-3.	Total diabetes, type 1 diabetes
Rao CR, Kamath VG, Shetty A, Kamath A. A study on the prevalence of type 2 diabetes in coastal Karnataka. Int J Diabetes Dev Ctries. 2010; 30(2): 80-5.	Total diabetes
Rathmann W, Haastert B, Icks A, Löwel H, Meisinger C, Holle R, Giani G. High prevalence of undiagnosed diabetes mellitus in Southern Germany: target populations for efficient screening. The KORA survey 2000. Diabetologia. 2003; 46(2): 182-9.	Total diabetes
Ravikumar P, Bhansali A, Ravikiran M, Bhansali S, Walia R, Shanmugasundar G, Thakur JS, Kumar Bhadada S, Dutta P. Prevalence and risk factors of diabetes in a community-based study in North India: the Chandigarh Urban Diabetes Study (CUDS). Diabetes Metab. 2011; 37(3): 216-21.	Total diabetes
Republican Scientific and Practical Center of Medical Technologies, Informatization, Management and Economics of Public Health (Belarus), World Health Organization (WHO). Belarus STEPS Noncommunicable Disease Risk Factors Survey 2016-2017.	Total diabetes
Rigo Carratala F, Frontera Juan G, Canaves Llobera J, Rodriguez Ruiz T, Borras Bosch T, Fuentespina Vidal E, . Prevalencia de factores de riesgo cardiovascular en IslasBaleares (estudio CORSAIB). Rev Esp Cardiol. 2005; 58(12): 1411-9.	Total diabetes
Rigo JC, Vieira JL, Dalacorte RR, Reichert CL. Prevalence of metabolic syndrome in an elderly community: comparison between three diagnostic methods. Arq Bras Cardiol. 2009; 93(2): 85-91.	Total diabetes

Roche EF, McKenna AM, Ryder KJ, Brennan AA, O'Regan M, Hoey HM. Is the incidence of type 1 diabetes in children and adolescents stabilising? The first 6 years of a National Register. Eur J Pediatr. 2016; 175(12): 1913–9.	Total diabetes, type 1 diabetes
Roche EF, Menon A, Gill D, Hoey HM. Incidence of type 1 diabetes mellitis in children aged under 15 years in the Republic of Ireland. J Pediatr Endocrinol Metab. 2002; 15(8): 1191-4.	Total diabetes, type 1 diabetes
Rodrigues Junior W, Gaban SCN, Pontes ERJC, Souza CC, Gimenes LP, Lacerda PF, Cunha MLMN, Stefanello II JVL, Brum LM, Oliveira LA, Silva CR, Ribeiro ALD. Diabetes mellitus and impaired glucose tolerance in urban adult population. Rev Assoc Med Bras. 2014; 60(2): 118-24.	Total diabetes
Rojnic Putarek N, Ille J, Spehar Uroic A, Skrabic V, Stipancic G, Krnic N, Radica A, Marjanac I, Severinski S, Svigir A, Bogdanic A, Dumic M. Incidence of type 1 diabetes mellitus in 0 to 14-yr-old children in Croatia - 2004 to 2012 study. Pediatr Diabetes. 2015; 16(6): 448–53.	Total diabetes, type 1 diabetes
Rolandsson O, Hägg E, Nilsson M, Hallmans G, Mincheva-Nilsson L, Lernmark A. Prediction of diabetes with body mass index, oral glucose tolerance test and islet cell autoantibodies in a regional population. J Intern Med. 2001; 249(4): 279-88.	Total diabetes
Rosado Martín J, Martínez López MÁ, Mantilla Morató T, Dujovne Kohan I, Palau Cuevas FJ, Torres Jiménez R, García Puig J. [Prevalence of diabetes in an adult population in the region of Madrid (Spain). The Madrid Cardiovascular Risk study]. Gac Sanit. 2012; 26(3): 243-50.	Total diabetes
Rosenbauer J, Herzig P, von Kries R, Neu A, Giani G. Temporal, seasonal, and geographical incidence patterns of type I diabetes mellitus in children under 5 years of age in Germany. Diabetologia. 1999; 42(9): 1055-9.	Total diabetes, type 1 diabetes
Rosero-Bixby, Luis , Xinia Fernández, and William H. Dow. CRELES: Costa Rican Longevity and Healthy Aging Study, 2005 (Costa Rica Estudio de Longevidad y Envejecimiento Saludable) [Computer file]. ICPSR26681-v1. Ann Arbor, MI: Inter-university Consortium for Political and Social Research [distributor], 2010-07-21. doi:10.3886/ICPSR26681	Total diabetes
Rosmond R, Björntorp P. Blood pressure in relation to obesity, insulin and the hypothalamic-pituitary-adrenal axis in Swedish men. J Hypertens. 1998; 16(12): 1721-6.	Total diabetes
Rosso D, Campagna S, Di Stefano F, Romano G, Maugeri D, Maggi S, Motta M, Catanzaro S, Carnazzo G. Prevalence of diabetes mellitus in a sample of the elderly population of the city of Catania. Arch Gerontol Geriatr. 1998; 27(3): 223-35.	Total diabetes, type 1 diabetes
Rouvre M, Vol S, Gusto G, Born C, Lantieri O, Tichet J, Lecomte P. Low high density lipoprotein cholesterol: prevalence and associated risk-factors in a large French population. Ann Epidemiol. 2011; 21(2): 118–27.	Total diabetes
Söderberg S, Zimmet P, Tuomilehto J, de Courten M, Dowse GK, Chitson P, Gareeboo H, Alberti KGMM, Shaw JE. Increasing prevalence of Type 2 diabetes mellitus in all ethnic groups in Mauritius. Diabet Med. 2005; 22(1): 61-8.	Total diabetes
Saadi H, Carruthers SG, Nagelkerke N, Al-Maskari F, Afandi B, Reed R, Lukic M, Nicholls MG, Kazam E, Algawi K, Al-Kaabi J, Leduc C, Sabri S, El-Sadig M, Elkhumaidi S, Agarwal M, Benedict S. Prevalence of diabetes mellitus and its complications in a population-based sample in Al Ain, United Arab Emirates. Diabetes Res Clin Pract. 2007; 78(3): 369-77.	Total diabetes
Sabir A, Ohwovoriole A, Isezuo S, Fasanmade O, Abubakar S, Iwuala S. Type 2 diabetes mellitus and its risk factors among the rural Fulanis of Northern Nigeria. Ann Afr Med. 2013; 12(4): 217-22.	Total diabetes
Sadikot SM, Nigam A, Das S, Bajaj S, Zargar AH, Prasannakumar KM, Sosale A, Munichoodappa C, Seshiah V, Singh SK, Jamal A, Sai K, Sadasivrao Y, Murthy SS, Hazra DK, Jain S, Mukherjee S, Bandyopadhay S, Sinha NK, Mishra R, Dora M, Jena B, Patra P, Goenka K. The burden of diabetes and impaired glucose tolerance in India using the WHO 1999 criteria: prevalence of diabetes in India study (PODIS). Diabetes Res Clin Pract. 2004; 66(3): 301-7.	Total diabetes
Salazar MR, Carbajal HA, Espeche WG, Aizpurua M, Leiva Sisnieguez CE, March CE, Balbin E, Stavile RN, Reaven GM. Identifying cardiovascular disease risk and outcome: use of the plasma triglyceride/high-density lipoprotein cholesterol concentration ratio versus metabolic syndrome criteria. J Intern Med. 2013; 273(6): 595–601.	Total diabetes
Salti IS, Khogali M, Alam S, Haidar NA, Masri A. Epidemiology of diabetes mellitus in relation to other cardiovascular risk factors in Lebanon. East Mediterr Health J. 1997; 3(3): 462-71.	Total diabetes
Samardzic M, Marinkovic J, Kocev N, Curovic N, Terzic N. Increasing incidence of childhood type 1 diabetes in Montenegro from 1997 to 2006. Pediatr Diabetes. 2010; 11(6): 412-6.	Total diabetes, type 1 diabetes
Samuel P, Antonisamy B, Raghupathy P, Richard J, Fall CHD. Socio-economic status and cardiovascular risk factors in rural and urban areas of Vellore, Tamilnadu, South India. Int J Epidemiol. 2012; 41(5): 1315-27.	Total diabetes
Sanisoglu SY, Oktenli C, Hasimi A, Yokusoglu M, Ugurlu M. Prevalence of metabolic syndrome-related disorders in a large adult population in Turkey. BMC Public Health. 2006; 92.	Total diabetes
Santander Center for Public Health (Colombia), Santander Ministry of Health (Colombia), World Health Organization (WHO). Colombia - Santander STEPS Noncommunicable Disease Risk Factors Survey 2010.	Total diabetes
Saquib N, Khanam MA, Saquib J, Anand S, Chertow GM, Barry M, Ahmed T, Cullen MR. High prevalence of type 2 diabetes among the urban middle class in Bangladesh. BMC Public Health. 2013; 1032.	Total diabetes
Sasaki H, Kawasaki T, Ogaki T, Kobayashi S, Itoh K, Yoshimizu Y, Sharma S, Acharya GP. The prevalence of diabetes mellitus and impaired fasting glucose/glycaemia (IFG) in suburban and rural Nepal-the communities--based cross-sectional study during the democratic movements in 1990. Diabetes Res Clin Pract. 2005; 67(2): 167-74.	Total diabetes
Sayeed MA, Hussain MZ, Banu A, Rumi MA, Azad Khan AK. Prevalence of diabetes in a suburban population of Bangladesh. Diabetes Res Clin Pract. 1997; 34(3): 149-55.	Total diabetes
Schöttker B, Raum E, Rothenbacher D, Müller H, Brenner H. Prognostic value of haemoglobin A1c and fasting plasma glucose for incident diabetes and implications for screening. Eur J Epidemiol. 2011; 26(10): 779–87.	Total diabetes
Schaan BD, Harzheim E, Gus I. [Cardiac risk profile in diabetes mellitus and impaired fasting glucose]. Rev Saude Publica. 2004; 38(4): 529-36.	Total diabetes
Scheltens T, Bots ML, Numans ME, Grobbee DE, Hoes AW. Awareness, treatment and control of hypertension: the "rule of halves" in an era of risk-based treatment of hypertension. J Hum Hypertens. 2007; 21(2): 99-106.	Total diabetes
Schober E, Rami B, Waldhoer T. Steep increase of incidence of childhood diabetes since 1999 in Austria. Time trend analysis 1979-2005. A nationwide study. Eur J Pediatr. 2008; 167(3): 293-7.	Total diabetes, type 1 diabetes
Schober E, Waldhoer T, Rami B, Hofer S. Incidence and time trend of type 1 and type 2 diabetes in Austrian children 1999-2007. J Pediatr. 2009; 155(2): 190-3e1.	Total diabetes, type 1 diabetes
Schranz AG. Abnormal glucose tolerance in the Maltese. A population-based longitudinal study of the natural history of NIDDM and IGT in Malta. Diabetes Res Clin Pract. 1989; 7(1): 7-16.	Total diabetes
ScotCen Social Research and University College London. Department of Epidemiology and Public Health, Scottish Health Survey, 2010 [computer file]. Colchester, Essex: UK Data Archive [distributor], April 2012. SN: 6987, http://dx.doi.org/10.5255/UKDA-SN-6987-1	Total diabetes
ScotCen Social Research, University College London. Department of Epidemiology and Public Health and University of Glasgow. MRC/CSO Social and Public Health Sciences Unit, Scottish Health Survey, 2011 [computer file]. 2nd Edition. Colchester, Essex: UK Data Archive [distributor], August 2013. SN: 7247, http://dx.doi.org/10.5255/UKDA-SN-7247-2	Total diabetes

Scott RS, Brown LJ. Prevalence and incidence of insulin-treated diabetes mellitus in adults in Canterbury, New Zealand. Diabet Med. 1991; 8(5): 443-7.	Type 1 diabetes
Scottish Centre for Social Research and University College London. Department of Epidemiology and Public Health, Scottish Health Survey, 2008 [computer file]. 2nd Edition. Colchester, Essex: UK Data Archive [distributor], April 2013. SN: 6383, http://dx.doi.org/10.5255/UKDA-SN-6383-2	Total diabetes
Scottish Centre for Social Research and University College London. Department of Epidemiology and Public Health, Scottish Health Survey, 2009 [computer file]. 4th Edition. Colchester, Essex: UK Data Archive [distributor], November 2011. SN: 6713, http://dx.doi.org/10.5255/UKDA-SN-6713-2	Total diabetes
Scuteri A, Najjar SS, Orru' M, Albai G, Strait J, Tarasov KV, Piras MG, Cao A, Schlessinger D, Uda M, Lakatta EG. Age- and gender-specific awareness, treatment, and control of cardiovascular risk factors and subclinical vascular lesions in a founder population: the SardiNIA Study. Nutr Metab Cardiovasc Dis. 2009; 19(8): 532–41.	Type 1 diabetes
Secretariat of Health and Environment (Libya), World Health Organization (WHO). Libya STEPS Noncommunicable Disease Risk Factors Survey 2009.	Total diabetes
Sekikawa A, Tominaga M, Takahashi K, Eguchi H, Igarashi M, Ohnuma H, Sugiyama K, Manaka H, Sasaki H, Fukuyama H. Prevalence of diabetes and impaired glucose tolerance in Funagata area, Japan. Diabetes Care. 1993; 16(4): 570-4.	Total diabetes
Sekita A, Arima H, Ninomiya T, Ohara T, Doi Y, Hirakawa Y, Fukuhara M, Hata J, Yonemoto K, Ga Y, Kitazono T, Kanba S, Kiyohara Y. Elevated depressive symptoms in metabolic syndrome in a general population of Japanese men: a cross-sectional study. BMC Public Health. 2013; 13: 862.	Total diabetes
Serban V, Brink S, Timar B, Sima A, Vlad M, Timar R, Vlad A. An increasing incidence of type 1 diabetes mellitus in Romanian children aged 0 to 17 years. J Pediatr Endocrinol Metab. 2015; 28(3-4): 293-8.	Total diabetes, type 1 diabetes
Serban V, Timar R, Dabelea D, Green A, McKinney P, Law G. The epidemiology of childhood-onset type 1 diabetes mellitus in Romania. ONROCAD Study Group. National Romanian Organisation for the Care of Diabetic Children and Adolescents. J Pediatr Endocrinol Metab. 2001; 14(5): 535-41.	Total diabetes, type 1 diabetes
Shah A, Afzal M. Prevalence of diabetes and hypertension and association with various risk factors among different Muslim populations of Manipur, India. J Diabetes Metab Disord. 2013; 12(1): 52.	Total diabetes
Shah C, Sheth NR, Solanki B, Shah N. To assess the prevalence of impaired glucose tolerance and impaired fasting glucose in Western Indian population. J Assoc Physicians India. 2013; 61(3): 179–84.	Total diabetes
Shaltout AA, Qabazard MA, Abdella NA, LaPorte RE, al Arouj M, Ben Nekhi A, Moussa MA, al Khawari MA. High incidence of childhood-onset IDDM in Kuwait. Kuwait Study Group of Diabetes in Childhood. Diabetes Care. 1995; 18(7): 923-7.	Total diabetes, type 1 diabetes
Shaltout AA, Wake D, Thanaraj TA, Omar DM, Al-AbdulRazzaq D, Channanath A, AlKandari H, Abdulrasoul M, Miller S, Conway N, Tuomilehto J, Davidsson L, on behalf of the Steering Group for the Study of Childhood Diabetes in Kuwait. Incidence of type 1 diabetes has doubled in Kuwaiti children 0-14 years over the last 20 years. Pediatr Diabetes. 2017; 18(8): 761–6.	Total diabetes, type 1 diabetes
Shan Y, Zhang Q, Liu Z, Hu X, Liu D. Prevalence and risk factors associated with chronic kidney disease in adults over 40 years: a population study from Central China. Nephrology (Carlton). 2010; 15(3): 354-61.	Total diabetes
Sharma SK, Ghimire A, Radhakrishnan J, Thapa L, Shrestha NR, Paudel N, Gurung K, R M, Budathoki A, Baral N, Brodie D. Prevalence of hypertension, obesity, diabetes, and metabolic syndrome in Nepal. Int J Hypertens. 2011; 821971.	Total diabetes
Shen SX, Wang HB, Chen ZW, Shen YE, Fu H, Wu CE, Ye TT, Wang JJ, Wang KA, Li TL, Yang Z, LaPorte RE, Dorman JS. The incidence of insulin-dependent diabetes mellitus in urban districts of Shanghai (1989-1993). J Pediatr Endocrinol Metab. 1996; 9(4): 469-73.	Total diabetes, type 1 diabetes
Sheng C-S, Liu M, Kang Y-Y, Wei F-F, Zhang L, Li G-L, Dong Q, Huang Q-F, Li Y, Wang J-G. Prevalence, awareness, treatment and control of hypertension in elderly Chinese. Hypertens Res. 2013; 36(9): 824–8.	Total diabetes
Shera AS, Rafique G, Khawaja IA, Ara J, Baqai S, King H. Pakistan national diabetes survey: prevalence of glucose intolerance and associated factors in Shikarpur, Sindh Province. Diabet Med. 1995; 12(12): 1116-21.	Total diabetes
Shera AS, Rafique G, Khawaja IA, Baqai S, King H. Pakistan National Diabetes Survey: prevalence of glucose intolerance and associated factors in Baluchistan province. Diabetes Res Clin Pract. 1999; 44(1): 49-58.	Total diabetes
Shrestha UK, Singh DL, Bhattarai MD. The prevalence of hypertension and diabetes defined by fasting and 2-h plasma glucose criteria in urban Nepal. Diabet Med. 2006; 23(10): 1130-5.	Total diabetes
Shrivastava SR, Ghorpade AG. High prevalence of type 2 diabetes melitus and its risk factors among the rural population of Pondicherry, South India. J Res Health Sci. 2014; 14(4): 258-63.	Total diabetes
Silva H, Hernandez-Hernandez R, Vinueza R, Velasco M, Boissonnet CP, Escobedo J, Silva HE, Pramparo P, Wilson E, CARMELA Study Investigators. Cardiovascular risk awareness, treatment, and control in urban Latin America. Am J Ther. 2010; 17(2): 159–66.	Total diabetes
Simmons D, McKenzie A, Eaton S, Shaw J, Zimmet P. Prevalence of diabetes in rural Victoria. Diabetes Res Clin Pract. 2005; 70(3): 287-90.	Total diabetes, type 1 diabetes
Singh AK, Mani K, Krishnan A, Aggarwal P, Gupta SK. Prevalence, awareness, treatment and control of diabetes among elderly persons in an urban slum of delhi. Indian J Community Med. 2012; 37(4): 236-9.	Total diabetes
Singh DL, Bhattarai MD. High prevalence of diabetes and impaired fasting glycaemia in urban Nepal. Diabet Med. 2003; 20(2): 170-1.	Total diabetes
Sipetic S, Maksimovic J, Vlajinac H, Ratkov I, Sajic S, Zdravkovic D, Sipetic T. Rising incidence of Type 1 diabetes in Belgrade children aged 0-14 years in the period from 1982 to 2005. J Endocrinol Invest. 2013; 36(5): 307-12.	Total diabetes, type 1 diabetes
Skordis N, Efstathiou E, Kyriakides TC, Savvidou A, Savva SC, Phylactou LA, Shammas C, Neocleous V. Epidemiology of type 1 diabetes mellitus in Cyprus: rising incidence at the dawn of the 21st century. Hormones (Athens). 2012; 11(1): 86-93.	Total diabetes, type 1 diabetes
Skordis N, Hadjiloizou S. Incidence of insulin dependent diabetes mellitus in Greek Cypriot children and adolescents, 1990-1994. J Pediatr Endocrinol Metab. 1997; 10(2): 203-7.	Total diabetes, type 1 diabetes
Skordis N, Theodorou S, Apsiotou T, Stavrou S, Herakleous E, Savva SC. The incidence of type 1 diabetes mellitus in Greek-Cypriot children and adolescents in 1990-2000. Pediatr Diabetes. 2002; 3(4): 200-4.	Total diabetes, type 1 diabetes
Skrivarhaug T, Stene LC, Drivvoll AK, Str?m H, Joner G. Incidence of type 1 diabetes in Norway among children aged 0-14 years between 1989 and 2012: has the incidence stopped rising? Results from the Norwegian Childhood Diabetes Registry. Diabetologia. 2014; 57(1): 57-62.	Total diabetes, type 1 diabetes
Smith KJ, Blizzard L, McNaughton SA, Gall SL, Dwyer T, Venn AJ. Daily eating frequency and cardiometabolic risk factors in young Australian adults: cross-sectional analyses. Br J Nutr. 2012; 108(6): 1086–94.	Total diabetes
Sobel-Maruniak A, Grzywa M, Orłowska-Florek R, Staniszewski A. The rising incidence of type 1 diabetes in south-eastern Poland. A study of the 0-29 year-old age group, 1980-1999. Endokrynol Pol. 2006; 57(2): 127-30.	Total diabetes, type 1 diabetes

Soegondo S, Widyahening IS, Istantho R, Yunir E. Prevalence of diabetes among suburban population of Ternate--a small remote island in the eastern part of Indonesia. Acta Med Indones. 2011; 43(2): 99-104.	Total diabetes
Solet J-L, Baroux N, Pochet M, Benoit-Cattin T, De Montera A-M, Sissoko D, Favier F, Fagot-Campagna A. Prevalence of type 2 diabetes and other cardiovascular risk factors in Mayotte in 2008: the MAYDIA study. Diabetes Metab. 2011; 37(3): 201-7.	Total diabetes
Solfrizzi V, Panza F, Colacicco AM, D'Introno A, Capurso C, Torres F, Grigoletto F, Maggi S, Del Parigi A, Reiman EM, Caselli RJ, Scafato E, Farchi G, Capurso A, Italian Longitudinal Study on Aging Working Group. Vascular risk factors, incidence of MCI, and rates of progression to dementia. Neurology. 2004; 63(10): 1882-91.	Total diabetes
Song K-H, Nam-Goomg IS, Han S-M, Kim M-S, Lee E-J, Lee YS, Lee MS, Yoon S, Lee K-U, Park J-Y. Change in prevalence and 6-year incidence of diabetes and impaired fasting glucose in Korean subjects living in a rural area. Diabetes Res Clin Pract. 2007; 78(3): 378-84.	Total diabetes
Songini M, Muntoni S. High incidence of type 1 diabetes in Sardinia. Gruppo Collaborativo per l'Epidemiologia dell'IDDM in Sardegna. Ann Ig. 1992; 4(3): 179-90.	Total diabetes, type 1 diabetes
Soria MLB, Sy RG, Vega BS, Ty-Willing T, Abenir-Gallardo A, Velandria F, Punzalan FE. The incidence of type 2 diabetes mellitus in the Philippines: a 9-year cohort study. Diabetes Res Clin Pract. 2009; 86(2): 130-3.	Total diabetes
Soriguer F, Goday A, Bosch-Comas A, Bordiú E, Calle-Pascual A, Carmena R, Casamitjana R, Castaño L, Castell C, Catalá M, Delgado E, Franch J, Gaztambide S, Girbés J, Gomis R, Gutiérrez G, López-Alba A, Martínez-Larrad MT, Menéndez E, Mora-Peces I, Ortega E, Pascual-Manich G, Rojo-Martínez G, Serrano-Rios M, Valdés S, Vázquez JA, Vendrell J. Prevalence of diabetes mellitus and impaired glucose regulation in Spain: the Di@bet.es Study. Diabetologia. 2012; 55(1): 88-93.	Total diabetes
Soriguer F, Rojo-Martínez G, Almaraz MC, Esteva I, Ruiz de Adana MS, Morcillo S, Valdés S, García-Fuentes E, García-Escobar E, Cardona I, Gomez-Zumaquero JM, Olveira-Fuster G. Incidence of type 2 diabetes in southern Spain (Pizarra Study). Eur J Clin Invest. 2008; 38(2): 126-33.	Total diabetes
Sossa C, Delisle H, Agueh V, Sodjinou R, Ntandou G, Makoutode M. Lifestyle and dietary factors associated with the evolution of cardiometabolic risk over four years in West-African adults: the Benin study. J Obes. 2013; 2013: 298024.	Total diabetes
Soysal A, Demiral Y, Soysal D, Uçku R, Köseoglu M, Aksakoglu G. The prevalence of metabolic syndrome among young adults in Izmir, Turkey. Anadolu Kardiyol Derg. 2005; 5(3): 196-201.	Total diabetes
Spaans EA, Gusdorf LM, Groenier KH, Brand PL, Veeze HJ, Reeser HM, Bilo HJ, Kleefstra N. The incidence of type 1 diabetes is still increasing in the Netherlands, but has stabilised in children under five (Young DUDEs-1). Acta Paediatr. 2015; 104(6): 626-9.	Total diabetes, type 1 diabetes
Staines A, Bodansky HJ, Lilley HE, Stephenson C, McNally RJ, Cartwright RA. The epidemiology of diabetes mellitus in the United Kingdom: the Yorkshire Regional Childhood Diabetes Register. Diabetologia. 1993; 36(12): 1282-7.	Total diabetes, type 1 diabetes
Stipancić G, La Grasta Sabolić L, Pozgaj Sepec M, Radica A, Skrabić V, Severinski S, Kujundzić Tiljak M. Regional differences in incidence and clinical presentation of type 1 diabetes in children aged under 15 years in Croatia. Croat Med J. 2012; 53(2): 141-8.	Total diabetes, type 1 diabetes
Stipancic G, La Grasta Sabolic L, Malenica M, Radica A, Skrabic V, Tiljak MK. Incidence and trends of childhood Type 1 diabetes in Croatia from 1995 to 2003. Diabetes Res Clin Pract. 2008; 80(1): 122-7.	Total diabetes, type 1 diabetes
Stolk RP, van Splunder IP, Schouten JS, Witteman JC, Hofman A, Grobbee DE. High blood pressure and the incidence of non-insulin dependent diabetes mellitus: findings in a 11.5 year follow-up study in The Netherlands. Eur J Epidemiol. 1993; 9(2): 134-9.	Total diabetes
Sutton DL, Lyle DM, Pierce JP. Incidence and prevalence of insulin-dependent diabetes mellitus in the zero- to 19-years' age-group in Sydney. Med J Aust. 1989; 151(3): 140-6.	Total diabetes, type 1 diabetes
Suvd J, Gerel B, Otgooloi H, Purevsuren D, Zolzaya H, Roglic G, King H. Glucose intolerance and associated factors in Mongolia: results of a national survey. Diabet Med. 2002; 19(6): 502–8.	Total diabetes
Svensson J, Carstensen B, Molbak A, Christau B, Mortensen HB, Nerup J, Borch-Johnsen K. Increased risk of childhood type 1 diabetes in children born after 1985. Diabetes Care. 2002; 25(12): 2197-201.	Total diabetes, type 1 diabetes
Swai AB, Mclarty DG, Mtinangi BL, Tatala S, Kitange HM, Mlingi N, Rosling H, Howlett WP, Brubaker GR, Alberti KG. Diabetes is not caused by cassava toxicity. A study in a Tanzanian community. Diabetes Care. 1992; 15(10): 1378-85.	Total diabetes
Swedish Association for Diabetology, Vastra Gotaland Registry Center. Sweden National Diabetes Register Tables 2008-2012.	Total diabetes, type 1 diabetes
Sy RG, Morales DD, Dans AL, Paz-Pacheco E, Punzalan FER, Abelardo NS, Duante CA. Prevalence of atherosclerosis-related risk factors and diseases in the Philippines. J Epidemiol. 2012; 22(5): 440-7.	Total diabetes
Tahirovic H, Toromanovic A. Incidence of type 1 diabetes mellitus in children in Tuzla Canton between 1995 and 2004. Eur J Pediatr. 2007; 166(5): 491-2.	Total diabetes, type 1 diabetes
Tamil Nadu Dr. M.G.R. Medical University. India - Chennai Urban Rural Epidemiology Study Blood Glucose, Cholesterol, BMI, and Diabetes Incidence Measurements, 2001-2013. [Unpublished].	Total diabetes
Tan WS, Ng CJ, Khoo E-M, Low W-Y, Tan HM. The triad of erectile dysfunction, testosterone deficiency syndrome and metabolic syndrome: findings from a multi-ethnic Asian men study (The Subang Men's Health Study). Aging Male. 2011; 14(4): 231–6.	Total diabetes
Taplin CE, Craig ME, Lloyd M, Taylor C, Crock P, Silink M, Howard NJ. The rising incidence of childhood type 1 diabetes in New South Wales, 1990-2002. Med J Aust. 2005; 183(5): 243-6.	Total diabetes, type 1 diabetes
Teeaar T, Liivak N, Heilman K, Kool P, Sor R, Paal M, Einberg U, Tillmann V. Increasing incidence of childhood-onset type 1 diabetes mellitus among Estonian children in 1999-2006. Time trend analysis 1983-2006. Pediatr Diabetes. 2010; 11(2): 107-10.	Total diabetes, type 1 diabetes
Temmar M, Labat C, Benkhedda S, Charifi M, Thomas F, Bouafia MT, Bean K, Darne B, Safar ME, Benetos A. Prevalence and determinants of hypertension in the Algerian Sahara. J Hypertens. 2007; 25(11): 2218-26.	Total diabetes
Thomas W, Birgit R, Edith S. Changing geographical distribution of diabetes mellitus type 1 incidence in Austrian children 1989--2005. Eur J Epidemiol. 2008; 23(3): 213-8.	Total diabetes, type 1 diabetes
Thuesen BH, Cerqueira C, Aadahl M, Ebstrup JF, Toft U, Thyssen JP, Fenger RV, Hersoug L-G, Elberling J, Pedersen O, Hansen T, Johansen JD, Jørgensen T, Linneberg A. Cohort Profile: the Health2006 cohort, research centre for prevention and health. Int J Epidemiol. 2014; 43(2): 568–75.	Total diabetes
Thunander M, Petersson C, Jonzon K, Fornander J, Ossiansson B, Torn C, Edvardsson S, Landin-Olsson M. Incidence of type 1 and type 2 diabetes in adults and children in Kronoberg, Sweden. Diabetes Res Clin Pract. 2008; 82(2): 247-55.	Type 1 diabetes
Tian H, Song G, Xie H, Zhang H, Tuomilehto J, Hu G. Prevalence of diabetes and impaired fasting glucose among 769,792 rural Chinese adults. Diabetes Res Clin Pract. 2009; 84(3): 273-8.	Total diabetes
Torquato MT, Montenegro Junior RM, Viana LA, de Souza RA, Lanna CM, Lucas JC, Bidurin C, Foss MC. Prevalence of diabetes mellitus and impaired glucose tolerance in the urban population aged 30-69 years in Ribeirao Preto (Sao Paulo), Brazil. Sao Paulo Med J. 2003; 121(6): 224-30.	Total diabetes
Tran F, Stone M, Huang CY, Lloyd M, Woodhead HJ, Elliott KD, Crock PA, Howard NJ, Craig ME. Population-based incidence of diabetes in Australian youth aged 10-18?yr: increase in type 1 diabetes but not type 2 diabetes. Pediatr Diabetes. 2014; 15(8): 585-90.	Total diabetes, type 1 diabetes

Truven Health Analytics. United States MarketScan Commercial Claims and Encounters Database 2000. Ann Arbor, United States of America: Truven Health Analytics.	Total diabetes, type 1 diabetes
Truven Health Analytics. United States MarketScan Commercial Claims and Encounters Database 2010. Ann Arbor, United States of America: Truven Health Analytics.	Total diabetes, type 1 diabetes
Truven Health Analytics. United States MarketScan Commercial Claims and Encounters Database 2011. Ann Arbor, United States of America: Truven Health Analytics.	Total diabetes, type 1 diabetes
Truven Health Analytics. United States MarketScan Commercial Claims and Encounters Database 2012. Ann Arbor, United States of America: Truven Health Analytics.	Total diabetes, type 1 diabetes
Truven Health Analytics. United States MarketScan Commercial Claims and Encounters Database 2013. Ann Arbor, United States of America: Truven Health Analytics.	Total diabetes, type 1 diabetes
Truven Health Analytics. United States MarketScan Commercial Claims and Encounters Database 2014. Ann Arbor, United States of America: Truven Health Analytics.	Total diabetes, type 1 diabetes
Truven Health Analytics. United States MarketScan Medicare Supplemental and Coordination of Benefits Database 2000. Ann Arbor, United States of America: Truven Health Analytics.	Total diabetes, type 1 diabetes
Truven Health Analytics. United States MarketScan Medicare Supplemental and Coordination of Benefits Database 2010. Ann Arbor, United States of America: Truven Health Analytics.	Total diabetes, type 1 diabetes
Truven Health Analytics. United States MarketScan Medicare Supplemental and Coordination of Benefits Database 2011. Ann Arbor, United States of America: Truven Health Analytics.	Total diabetes, type 1 diabetes
Truven Health Analytics. United States MarketScan Medicare Supplemental and Coordination of Benefits Database 2012. Ann Arbor, United States of America: Truven Health Analytics.	Total diabetes, type 1 diabetes
Truven Health Analytics. United States MarketScan Medicare Supplemental and Coordination of Benefits Database 2013. Ann Arbor, United States of America: Truven Health Analytics.	Total diabetes, type 1 diabetes
Truven Health Analytics. United States MarketScan Medicare Supplemental and Coordination of Benefits Database 2014. Ann Arbor, United States of America: Truven Health Analytics.	Total diabetes, type 1 diabetes
Tsirona S, Katsaros F, Bargiota A, Polyzos SA, Arapoglou G, Koukoulis GN. Prevalence and determinants of type 2 diabetes mellitus in a Greek adult population. Hormones (Athens). 2016; 15(1): 88–98.	Total diabetes
Tufton N, Chowdhury T. Prevalence of Diabetes on Santa Cruz Island in Galapagos Archipelago. Prev Chronic Dis. 2015; E94.	Total diabetes
Tuomilehto J, Karvonen M, Pitkaniemi J, Virtala E, Kohtamaki K, Toivanen L, Tuomilehto-Wolf E. Record-high incidence of Type I (insulin-dependent) diabetes mellitus in Finnish children. The Finnish Childhood Type I Diabetes Registry Group. Diabetologia. 1999; 42(6): 655-60.	Total diabetes, type 1 diabetes
Tuomilehto J, Nissinen A, Kivelä SL, Pekkanen J, Kaarsalo E, Wolf E, Aro A, Punsar S, Karvonen MJ. Prevalence of diabetes mellitus in elderly men aged 65 to 84 years in eastern and western Finland. Diabetologia. 1986; 29(9): 611-5.	Total diabetes
Tuomilehto J, Podar T, Brigis G, Urbonaite B, Rewers M, Adojaan B, Cepaitis Z, Kalits I, King H, LaPorte R. Comparison of the incidence of insulin-dependent diabetes mellitus in childhood among five Baltic populations during 1983-1988. Int J Epidemiol. 1992; 21(3): 518-27.	Total diabetes, type 1 diabetes
Tuomilehto J, Rewers M, Reunanen A, Lounamaa P, Lounamaa R, Tuomilehto-Wolf E, Akerblom HK. Increasing trend in type 1 (insulin-dependent) diabetes mellitus in childhood in Finland. Analysis of age, calendar time and birth cohort effects during 1965 to 1984. Diabetologia. 1991; 34(4): 282-7.	Total diabetes, type 1 diabetes
Tuomilehto J, Virtala E, Karvonen M, Lounamaa R, Pitk„niemi J, Reunanen A, Tuomilehto-Wolf E, Toivanen L. Increase in incidence of insulin-dependent diabetes mellitus among children in Finland. Int J Epidemiol. 1995; 24(5): 984-92.	Total diabetes, type 1 diabetes
Ufa Eye Research Institute. Russia - Ural Eye and Medical Study 2015-2017.	Total diabetes
Ufa Eye Research Institute. Russia Ural Very Old Study 2017-2020.	Total diabetes
Ulmer H, Kelleher CC, Fitz-Simon N, Diem G, Concin H. Secular trends in cardiovascular risk factors: an age-period cohort analysis of 698,954 health examinations in 181,350 Austrian men and women. J Intern Med. 2007; 261(6): 566-76.	Total diabetes
Unachak K, Tuchinda C. Incidence of type 1 diabetes in children under 15 years in northern Thailand, from 1991 to 1997. J Med Assoc Thai. 2001; 84(7): 923-8.	Type 1 diabetes
Unal B, Sozmen K, Ucku R, Ergor G, Soysal A, Baydur H, Meseri R, Simsek H, Gerceklioglu G, Doganay S, Budak R, Kilic B, Gunay T, Ergor A, Demiral Y, Aslan O, Cimrin D, Akvardar Y, Tuncel P. High prevalence of cardiovascular risk factors in a Western urban Turkish population: a community-based study. Anadolu Kardiyol Derg. 2013; 13(1): 9–17.	Total diabetes
University of the West Indies. Jamaica Health and Lifestyle Survey 2007-2008.	Total diabetes
Upmeier E, Vire J, Korhonen MJ, Isoaho H, Lehtonen A, Arve S, Wuorela M, Viitanen M. Cardiovascular risk profile and use of statins at the age of 70 years: a comparison of two Finnish birth cohorts born 20 years apart. Age Ageing. 2016; 45(1): 84-90.	Total diabetes
Urbonaite B, Zalinkevicius R, Green A. Incidence, prevalence, and mortality of insulin-dependent (type 1) diabetes mellitus in Lithuanian children during 1983-98. Pediatr Diabetes. 2002; 3(1): 23-30.	Total diabetes, type 1 diabetes
Valdes S, Botas P, Delgado E, Alvarez F, Cadorniga FD. Population-based incidence of type 2 diabetes in northern Spain: the Asturias Study. Diabetes Care. 2007; 30(9): 2258-63.	Total diabetes
Valent F, Candido R, Faleschini E, Tonutti L, Tortul C, Zanatta M, Zanette G, Zanier L. The incidence rate and prevalence of pediatric type 1 diabetes mellitus (age 0-18) in the Italian region Friuli Venezia Giulia: population-based estimates through the analysis of health administrative databases. Acta Diabetol. 2016; 53(4): 629–35.	Total diabetes, type 1 diabetes
Verrillo A, de Teresa A, La Rocca S, Giarrusso PC. Prevalence of diabetes mellitus and impaired glucose tolerance in a rural area of Italy. Diabetes Res. 1985; 2(6): 301-6.	Total diabetes
Vijayakumar G, Arun R, Kutty VR. High prevalence of type 2 diabetes mellitus and other metabolic disorders in rural Central Kerala. J Assoc Physicians India. 2009; 563-7.	Total diabetes
Wadsworth E, Shield J, Hunt L, Baum D. Insulin dependent diabetes in children under 5: incidence and ascertainment validation for 1992. BMJ. 1995; 310(6981): 700-3.	Total diabetes, type 1 diabetes
Wandell PE, Carlsson AC, de Faire U, Hellenius M-L. Prevalence of blood lipid disturbances in Swedish and foreign-born 60-year-old men and women in Stockholm, Sweden. Nutr Metab Cardiovasc Dis. 2011; 21(3): 173–81.	Total diabetes
Wang C, Hou X, Bao Y, Pan J, Zuo Y, Zhong W, Jia W, Xiang K. The metabolic syndrome increased risk of cardiovascular events in Chinese--a community based study. Int J Cardiol. 2010; 139(2): 159–65.	Total diabetes

Wang F, Ye P, Luo L, Xiao W, Qi L, Bian S, Wu H, Sheng L, Xiao T, Xu R. Association of serum lipids with arterial stiffness in a population-based study in Beijing. Eur J Clin Invest. 2011; 41(9): 929–36.	Total diabetes
Wang H, Qiu Q, Tan LL, Liu T, Deng XQ, Chen YM, Chen W, Yu XQ, Hu BJ, Chen WQ. Prevalence and determinants of diabetes and impaired fasting glucose among urban community-dwelling adults in Guangzhou, China. Diabetes Metab. 2009; 35(5): 378-84.	Total diabetes
Wang R, Zhang P, Lv X, Jiang L, Gao C, Song Y, Yu Y, Li B. Situation of Diabetes and Related Disease Surveillance in Rural Areas of Jilin Province, Northeast China. Int J Environ Res Public Health. 2016; 13(6).	Total diabetes
Wang S, Ma W, Yuan Z, Wang S-M, Yi X, Jia H, Xue F. Association between obesity indices and type 2 diabetes mellitus among middle-aged and elderly people in Jinan, China: a cross-sectional study. BMJ Open. 2016; 6(11): e012742.	Total diabetes
Wang T, Bi Y, Xu M, Huang Y, Xu Y, Li X, Wang W, Ning G. Serum uric acid associates with the incidence of type 2 diabetes in a prospective cohort of middle-aged and elderly Chinese. Endocrine. 2011; 40(1): 109–16.	Total diabetes
Warsy AS, el-Hazmi MA. Diabetes mellitus, hypertension and obesity--common multifactorial disorders in Saudis. East Mediterr Health J. 1999; 5(6): 1236-42.	Total diabetes
Washington RE, Orchard TJ, Arena VC, Laporte RE, Tull ES. Incidence of type 1 and type 2 diabetes in youth in the U.S. Virgin Islands, 2001-2010. Pediatr Diabetes. 2013; 14(4): 280-7.	Total diabetes, type 1 diabetes
Waspadji S, Ranakusuma AB, Suyono S, Supartondo S, Sukaton U. Diabetes mellitus in an urban population in Jakarta, Indonesia. Tohoku J Exp Med. 1983; 141(Suppl): 219-28.	Total diabetes
Waugh NR. Insulin-dependent diabetes in a Scottish region: incidence and urban/rural differences. J Epidemiol Community Health. 1986; 40(3): 240-3.	Total diabetes, type 1 diabetes
Weets I, De Leeuw IH, Du Caju MV, Rooman R, Keymeulen B, Mathieu C, Rottiers R, Daubresse JC, Rocour-Brumioul D, Pipeleers DG, Gorus FK. The incidence of type 1 diabetes in the age group 0-39 years has not increased in Antwerp (Belgium) between 1989 and 2000: evidence for earlier disease manifestation. Diabetes Care. 2002; 25(5): 840-6.	Total diabetes, type 1 diabetes
Wei Q, Sun J, Huang J, Zhou HY, Ding YM, Tao YC, He SM, Liu YL, Niu JQ. Prevalence of hypertension and associated risk factors in Dehui City of Jilin Province in China. J Hum Hypertens. 2015; 29(1): 64–8.	Total diabetes
Weinstein, Maxine, Noreen Goldman, Ming-Cheng Chang, Hui-Sheng Lin, Yi-Li Chuang, Christine E. Peterson, Dana A. Glei, Baai-Shyun Hurng, Yu-Hsuan Lin, Shu-Hui Lin, I-Wen Liu, Hsia-Yuan Liu, Shio-Jean Lin, Chun-Ming Wu, Mei-Ling Hsiao, and Shiow-lng Wu. Social Environment and Biomarkers of Aging Study (SEBAS) in Taiwan, 2000 and 2006 /Computer file/. ICPSR 3792.vS. Ann Arbor, MI: Inter-university Consortium/or Political and Social Research /distributor/, 2011-06-17. doi: 10.3886/ICPSR03792	Total diabetes
Welin L, Eriksson H, Larsson B, Ohlson LO, Svärdsudd K, Tibblin G. Hyperinsulinaemia is not a major coronary risk factor in elderly men. The study of men born in 1913. Diabetologia. 1992; 35(8): 766-70.	Total diabetes
Weng J, Zhou Z, Guo L, Zhu D, Ji L, Luo X, Mu Y, Jia W. Incidence of type 1 diabetes in China, 2010-13: population based study. BMJ. 2018; 360: j5295.	Total diabetes, type 1 diabetes
Wijewardene K, Mohideen MR, Mendis S, Fernando DS, Kulathilaka T, Weerasekara D, Uluwitta P. Prevalence of hypertension, diabetes and obesity: baseline findings of a population based survey in four provinces in Sri Lanka. Ceylon Med J. 2005; 50(2): 62-70.	Total diabetes
Wittek A, Sokalski B, Grzeszczak W, Strojek K. Prevalence of diabetes and cardiovascular risk factors of industrial area in southern Poland. Exp Clin Endocrinol Diabetes. 2009; 117(7): 350-3.	Total diabetes
World Health Organization (WHO), Ministry of Health (Mongolia), National Medical Research Institute (Mongolia), Health Sciences University (Mongolia), National Oncology Center of Mongolia (Mongolia). Mongolia STEPS Noncommunicable Disease Risk Factors Survey 2005.	Total diabetes
World Health Organization (WHO), Ministry of Health (Mongolia), National Medical Research Institute (Mongolia), Health Sciences University (Mongolia), National Oncology Center of Mongolia (Mongolia). Mongolia STEPS Noncommunicable Disease Risk Factors Survey 2009.	Total diabetes
World Health Organization (WHO), Ministry of Health (Seychelles), Institute of Social and Preventive Medicine, University of Lausanne (Switzerland), University Hospital Center (Switzerland). Seychelles STEPS Noncommunicable Disease Risk Factors Survey 2004.	Total diabetes
World Health Organization (WHO), Ministry of Health and Medical Education (Iran), Center for Non-Communicable Diseases Control (Iran). Iran STEPS Noncommunicable Disease Risk Factors Survey 2005.	Total diabetes
World Health Organization (WHO). Armenia STEPS Noncommunicable Disease Risk Factors Survey 2016.	Total diabetes
World Health Organization (WHO). Brunei STEPS Noncommunicable Disease Risk Factors Survey 2015-2016.	Total diabetes
World Health Organization (WHO). Cape Verde STEPS Noncommunicable Disease Risk Factors Survey 2007.	Total diabetes
World Health Organization (WHO). Central African Republic - Bangui and Ombella M'Poko STEPS Noncommunicable Disease Risk Factors Survey 2017.	Total diabetes
World Health Organization (WHO). Central African Republic - Bangui STEPS Noncommunicable Disease Risk Factors Survey 2010.	Total diabetes
World Health Organization (WHO). Comoros STEPS Noncommunicable Disease Risk Factors Survey 2011.	Total diabetes
World Health Organization (WHO). Eritrea STEPS Noncommunicable Disease Risk Factors Survey 2010.	Total diabetes
World Health Organization (WHO). Fiji STEPS Noncommunicable Disease Risk Factors Survey 2011.	Total diabetes
World Health Organization (WHO). Georgia STEPS Noncommunicable Disease Risk Factors Survey 2010.	Total diabetes
World Health Organization (WHO). Guinea STEPS Noncommunicable Disease Risk Factors Survey 2009.	Total diabetes
World Health Organization (WHO). Kazakhstan - Aktobe Oblast STEPS Noncommunicable Disease Risk Factors Survey 2016.	Total diabetes
World Health Organization (WHO). Kazakhstan - Almaty STEPS Noncommunicable Disease Risk Factors Survey 2015.	Total diabetes
World Health Organization (WHO). Kazakhstan - Shymkent STEPS Noncommunicable Disease Risk Factors Survey 2015.	Total diabetes
World Health Organization (WHO). Laos STEPS Noncommunicable Disease Risk Factors Survey 2013.	Total diabetes
World Health Organization (WHO). Liberia STEPS Noncommunicable Disease Risk Factors Survey 2011.	Total diabetes
World Health Organization (WHO). Malawi STEPS Noncommunicable Disease Risk Factors Survey 2017.	Total diabetes

World Health Organization (WHO). Mali STEPS Noncommunicable Disease Risk Factors Survey 2007.	Total diabetes
World Health Organization (WHO). Micronesia - Kosrae STEPS Noncommunicable Disease Risk Factors Survey 2009.	Total diabetes
World Health Organization (WHO). Micronesia - Pohnpei STEPS Noncommunicable Disease Risk Factors Survey 2008 .	Total diabetes
World Health Organization (WHO). Micronesia - Yap STEPS Noncommunicable Disease Risk Factors Survey 2009.	Total diabetes
World Health Organization (WHO). Niue STEPS Noncommunicable Disease Risk Factors Survey 2011-2012.	Total diabetes
World Health Organization (WHO). Papua New Guinea STEPS Noncommunicable Disease Risk Factors Survey 2007-2008.	Total diabetes
World Health Organization (WHO). Sao Tome and Principe STEPS Noncommunicable Disease Risk Factors Survey 2008.	Total diabetes
World Health Organization (WHO). Vietnam - Cần Thơ STEPS Noncommunicable Disease Risk Factors Survey 2005.	Total diabetes
World Health Organization (WHO). Zambia STEPS Noncommunicable Disease Risk Factors Survey 2017. Geneva, Switzerland: World Health Organization (WHO).	Total diabetes
Wu D, Kendall D, Lunt H, Willis J, Darlow B, Frampton C. Prevalence of Type 1 diabetes in New Zealanders aged 0-24 years. N Z Med J. 2005; 118(1218): U1557.	Type 1 diabetes
Wu HB, Zhong JM, Hu RY, Wang H, Gong WW, Pan J, Fei FR, Wang M, Guo LH, Yang L, Yu M. Rapidly rising incidence of Type 1 diabetes in children and adolescents aged 0-19 years in Zhejiang, China, 2007 to 2013. Diabet Med. 2016; 33(10): 1339–46.	Type 1 diabetes
Wyka J, Biernat J, Kiedik D. Nutritional determination of the health status in Polish elderly people from an urban environment. J Nutr Health Aging. 2010; 14(1): 67-71.	Total diabetes
Xie J, Guan F, Wang J-H, Hu D-Y. Reasons for the upsetting cholesterol level during the community investigation from residents, physicians, and social aspects: the China Cholesterol Education Program (CCEP). Chin Med J (Engl). 2011; 124(19): 3030–4.	Total diabetes
Xin Z, Yuan M-X, Li H-X, Hua L, Feng J-P, Shi J, Zhu X-R, Cao X, Yang J-K. Evaluation for fasting and 2-hour glucose and HbA1c for diagnosing diabetes based on prevalence of retinopathy in a Chinese population. PLoS One. 2012; 7(7): e40610.	Total diabetes
Xu J, Xu L, Du KF, Shao L, Chen CX, Zhou JQ, Wang YX, You QS, Jonas JB, Wei WB. Subfoveal choroidal thickness in diabetes and diabetic retinopathy [Unpublished data]. Ophthalmology. 2013 Oct;120(10):2023-8.	Total diabetes
Xu S, Ming J, Xing Y, Gao B, Yang C, Ji Q, Chen G. Regional differences in diabetes prevalence and awareness between coastal and interior provinces in China: a population-based cross-sectional study. BMC Public Health. 2013; 299.	Total diabetes
Xu WL, Qiu CX, Wahlin A, Winblad B, Fratiglioni L. Diabetes mellitus and risk of dementia in the Kungsholmen project: a 6-year follow-up study. Neurology. 2004; 63(7): 1181–6.	Total diabetes
Xu Y, Wang L, He J, Bi Y, Li M, Wang T, Wang L, Jiang Y, Dai M, Lu J, Xu M, Li Y, Hu N, Li J, Mi S, Chen CS, Li G, Mu Y, Zhao J, Kong L, Chen J, Lai S, Wang W, Zhao W, Ning G. Prevalence and control of diabetes in Chinese adults. JAMA. 2013; 310(9): 948-59.	Total diabetes
Yamamoto Kimura L, Zamora Gonzalez J, Garcia de la Torre G, Cardoso Saldana G, Fajardo Gutierrez A, Ayala Barajas C, Posadas Romero C. Prevalence of high blood pressure and associated coronary risk factors in an adult population of Mexico city. Arch Med Res. 1998; 29(4): 341-9.	Total diabetes
Yang WS, Chen PC, Lin HJ, Su TC, Hsu HC, Chen MF, Lee YT, Chien KL. Association between type 2 diabetes and cancer incidence in Taiwan: data from a prospective community-based cohort study. Acta Diabetol. 2017; 54(5): 455-461.	Total diabetes
Ylihärsilä H, Lindström J, Eriksson JG, Jousilahti P, Valle TT, Sundvall J, Tuomilehto J. Prevalence of diabetes and impaired glucose regulation in 45- to 64-year-old individuals in three areas of Finland. Diabet Med. 2005; 22(1): 88-91.	Total diabetes
Yu S, Guo X, Yang H, Zheng L, Sun Y. An update on the prevalence of metabolic syndrome and its associated factors in rural northeast China. BMC Public Health. 2014; 14: 877.	Total diabetes
Zafar J, Bhatti F, Akhtar N, Rasheed U, Bashir R, Humayun S, Waheed A, Younus F, Nazar M, Umaimato. Prevalence and risk factors for diabetes mellitus in a selected urban population of a city in Punjab. J Pak Med Assoc. 2011; 61(1): 40-7.	Total diabetes
Zaman FA, Borang A. Prevalence of diabetes mellitus amongst rural hilly population of North Eastern India and its relationship with associated risk factors and related co-morbidities. J Nat Sci Biol Med. 2014; 5(2): 383–8.	Total diabetes
Zargar AH, Khan AK, Masoodi SR, Laway BA, Wani AI, Bashir MI, Dar FA. Prevalence of type 2 diabetes mellitus and impaired glucose tolerance in the Kashmir Valley of the Indian subcontinent. Diabetes Res Clin Pract. 2000; 47(2): 135-46.	Total diabetes
Zatońska K, Ilow R, Regulska-Ilow B, Różańska D, Szuba A, Wołyniec M, Einhorn J, Vatten L, Asvold BO, Mańczuk M, Zatoński WA. Prevalence of diabetes mellitus and IFG in the prospective cohort 'PONS' study - baseline assessment. Ann Agric Environ Med. 2011; 18(2): 265-9.	Total diabetes
Zhan Y, Zhuang J, Dong Y, Xu H, Hu D, Yu J. Predicting the prevalence of peripheral arterial diseases: modelling and validation in different cohorts. VASA. 2016; 45(1): 31-9.	Total diabetes
Zhang H, Xu W, Dahl AK, Xu Z, Wang H-X, Qi X. Relation of socio-economic status to impaired fasting glucose and Type 2 diabetes: findings based on a large population-based cross-sectional study in Tianjin, China. Diabet Med. 2013; 30(5): e157–62.	Total diabetes
Zhang L, Qin L-Q, Liu A-P, Wang P-Y. Prevalence of risk factors for cardiovascular disease and their associations with diet and physical activity in suburban Beijing, China. J Epidemiol. 2010; 20(3): 237–43.	Total diabetes
Zhang L, Zhang W-H, Zhang L, Wang P-Y. Prevalence of overweight/obesity and its associations with hypertension, diabetes, dyslipidemia, and metabolic syndrome: a survey in the suburban area of Beijing, 2007. Obes Facts. 2011; 4(4): 284-9.	Total diabetes
Zhang S, Tong W, Xu T, Wu B, Zhang Y. Diabetes and impaired fasting glucose in Mongolian population, Inner Mongolia, China. Diabetes Res Clin Pract. 2009; 86(2): 124-9.	Total diabetes
Zhang YH, Ma WJ, Thomas GN, Xu YJ, Lao XQ, Xu XJ, Song XL, Xu HF, Cai QM, Xia L, Nie SP, Deng HH, Yu IT. Diabetes and pre-diabetes as determined by glycated haemoglobin A1c and glucose levels in a developing southern Chinese population. PLoS One. 2012; 7(5): e37260.	Total diabetes
Zhao Y, Yang K, Wang F, Liang Y, Peng Y, Shen R, Wong T, Wang N. Associations between metabolic syndrome and syndrome components and retinal microvascular signs in a rural Chinese population: the Handan Eye Study. Graefes Arch Clin Exp Ophthalmol. 2012; 250(12): 1755–63.	Total diabetes
Zhao Z, Sun C, Wang C, Li P, Wang W, Ye J, Gu X, Wang X, Shen S, Zhi D, Lu Z, Ye R, Cheng R, Xi L, Li X, Zheng Z, Zhang M, Luo F. Rapidly rising incidence of childhood type 1 diabetes in Chinese population: epidemiology in Shanghai during 1997-2011. Acta Diabetol. 2014; 51(6): 947-53.	Total diabetes, type 1 diabetes
Zhou X, Guan H, Zheng L, Li Z, Guo X, Yang H, Yu S, Sun G, Li W, Hu W, Guo L, Pan G, Xing L, Zhang Y, Sun Y. Prevalence and awareness of diabetes mellitus among a rural population in China: results from Liaoning Province. Diabet Med. 2015; 32(3): 332-42.	Total diabetes

Zhou X, Ji L, Luo Y, Han X, Zhang X, Sun X, Ren Q, Qiao Q. Risk factors associated with the presence of diabetes in Chinese communities in Beijing. Diabetes Res Clin Pract. 2009; 86(3): 233–8.	Total diabetes
Zhuang J, Zhan Y, Zhang F, Tang Z, Wang J, Sun Y, Ding R, Hu D, Yu J. Self-reported insomnia and coronary heart disease in the elderly. Clin Exp Hypertens. 2016; 38(1): 51–5.	Total diabetes
Zung A, Blumenfeld O, Shehadeh N, Dally Gottfried O, Tenenbaum Rakover Y, Hershkovitz E, Gillis D, Zangen D, Pinhas-Hamiel O, Hanukoglu A, Rachmiel M, Shalitin S. Increase in the incidence of type 1 diabetes in Israeli children following the Second Lebanon War. Pediatr Diabetes. 2012; 13(4): 326-33.	Total diabetes, type 1 diabetes

Table S26. Diabetes relative risk input data source citations

Citation	Model
Abbasi A, Corpeleijn E, Gansevoort RT, Gans RO, Hillege HL, Stolk RP, Navis G, Bakker SJ, Dullaart RP. Role of HDL cholesterol and estimates of HDL particle composition in future development of type 2 diabetes in the general population: the PREVEND study. J Clin Endocrinol Metab. 2013; 98(8): E1352-9.	High alcohol use
Agardh EE, Lundin A, Lager A, Allebeck P, Koupil I, Andreasson S, Östenson CG, Danielsson AK. Alcohol and type 2 diabetes: The role of socioeconomic, lifestyle and psychosocial factors. Scand J Public Health. 2019; 47(4): 408-416.	High alcohol use
Ahmed A, Lager A, Fredlund P, Elinder LS. Consumption of fruit and vegetables and the risk of type 2 diabetes: a 4-year longitudinal study among Swedish adults. J Nutr Sci. 2020; 9: e14.	Diet low in vegetables
Akter S, Okazaki H, Kuwahara K, Miyamoto T, Murakami T, Shimizu C, Shimizu M, Tomita K, Nagahama S, Eguchi M, Kochi T, Imai T, Nishihara A, Sasaki N, Nakagawa T, Yamamoto S, Honda T, Uehara A, Yamamoto M, Hori A, Sakamoto N, Nishiura C, Totsuzaki T, Kato N, Fukasawa K, Pham NM, Kurotani K, Nanri A, Kabe I, Mizoue T, Sone T, Dohi S, Group the JEC on OHS. Smoking, Smoking Cessation, and the Risk of Type 2 Diabetes among Japanese Adults: Japan Epidemiology Collaboration on Occupational Health Study. PLoS One. 2015; 10(7): e0132166.	Smoking
Baan CA, Stolk RP, Grobbee DE, Witteman JC, Feskens EJ. Physical activity in elderly subjects with impaired glucose tolerance and newly diagnosed diabetes mellitus. Am J Epidemiol. 1999; 149(3): 219-27.	Low physical activity
Barclay AW, Flood VM, Rochtchina E, Mitchell P, Brand-Miller JC. Glycemic index, dietary fiber, and risk of type 2 diabetes in a cohort of older Australians. Diabetes Care. 2007; 30(11): 2811-3.	Diet low in fiber
Barouti AA, Tynelius P, Lager A, Björklund A. Fruit and vegetable intake and risk of prediabetes and type 2 diabetes: results from a 20-year long prospective cohort study in Swedish men and women. Eur J Nutr. 2022.	Diet low in vegetables
Bazzano LA, Li TY, Joshipura KJ, Hu FB. Intake of fruit, vegetables, and fruit juices and risk of diabetes in women. Diabetes Care. 2008; 31(7): 1311–7.	Diet low in fruits, Diet low in vegetables
Beulens JW, Stolk RP, van der Schouw YT, Grobbee DE, Hendriks HF, Bots ML. Alcohol consumption and risk of type 2 diabetes among older women. Diabetes Care. 2005; 28(12): 2933-8.	High alcohol use
Beulens JW, van der Schouw YT, Bergmann MM, Rohrmann S, Schulze MB, Buijsse B, Grobbee DE, Arriola L, Cauchi S, Tormo MJ, Allen NE, van der A DL, Balkau B, Boeing H, Clavel-Chapelon F, de Lauzon-Guillan B, Franks P, Froguel P, Gonzales C, Halkjaer J, Huerta JM, Kaaks R, Key TJ, Khaw KT, Krogh V, Molina-Montes E, Nilsson P, Overvad K, Palli D, Panico S, Ramon Quiros J, Rolandsson O, Ronaldsson O, Romieu I, Romaguera D, Sacerdote C, Sanchez MJ, Spijkerman AM, Teucher B, Tjonneland A, Tumino R, Sharp S, Forouhi NG, Langenberg C, Feskens EJ, Riboli E, Wareham NJ. Alcohol consumption and risk of type 2 diabetes in European men and women: influence of beverage type and body size The EPIC-InterAct study. J Intern Med. 2012; 272(4): 358-70.	High alcohol use
Bhaskaran K, Dos-Santos-Silva I, Leon DA, Douglas IJ, Smeeth L. Association of BMI with overall and cause-specific mortality: a population-based cohort study of 36 million adults in the UK. Lancet Diabetes Endocrinol. 2018; 6(12): 944-953.	High body-mass index
Bhupathiraju SN, Pan A, Malik VS, Manson JE, Willett WC, van Dam RM, Hu FB. Caffeinated and caffeine-free beverages and risk of type 2 diabetes. Am J Clin Nutr. 2013; 97(1): 155-66.	Diet high in sugar-sweetened beverages
Boggs DA, Rosenberg L, Ruiz-Narvaez EA, Palmer JR. Coffee, tea, and alcohol intake in relation to risk of type 2 diabetes in African American women. Am J Clin Nutr. 2010; 92(4): 960-6.	High alcohol use

Borné Y, Nilsson PM, Melander O, Hedblad B, Engström G. Multiple anthropometric measures in relation to incidence of diabetes: a Swedish population-based cohort study. Eur J Public Health. 2015; 25(6): 1100-5.	High body-mass index
Bowe B, Xie Y, Li T, Yan Y, Xian H, Al-Aly Z. The 2016 global and national burden of diabetes mellitus attributable to PM2·5 air pollution. Lancet Planet Health. 2018; 2(7): e301–12.	Ambient particulate matter pollution and household air pollution
Burnett RT. Cox Proportional Survival Model Hazard Ratios from Census Year to 2011 for Adults Aged 25 to 89 in CanCHEC Cohort.	Ambient particulate matter pollution and household air pollution
Carlsson S, Hammar N, Grill V, Kaprio J. Alcohol consumption and the incidence of type 2 diabetes: a 20-year follow-up of the Finnish twin cohort study. Diabetes Care. 2003; 26(10): 2785-90.	High alcohol use
Carlsson S, Midthjell K, Grill V, Nord-Trondelag study. Smoking is associated with an increased risk of type 2 diabetes but a decreased risk of autoimmune diabetes in adults: an 11-year follow-up of incidence of diabetes in the Nord-Trondelag study. Diabetologia. 2004; 47(11): 1953-6.	Smoking
Carlsson S, Midthjell K, Tesfamarian MY, Grill V. Age, overweight and physical inactivity increase the risk of latent autoimmune diabetes in adults: results from the Nord-Trøndelag health study. Diabetologia. 2007; 50(1): 55-8.	Low physical activity
Chen GC, Koh WP, Yuan JM, Qin LQ, van Dam RM. Green leafy and cruciferous vegetable consumption and risk of type 2 diabetes: results from the Singapore Chinese Health Study and meta-analysis. Br J Nutr. 2018; 119(9): 1057-1067.	Diet low in vegetables
Chen H, Burnett RT, Kwong JC, Villeneuve PJ, Goldberg MS, Brook RD, van Donkelaar A, Jerrett M, Martin RV, Brook JR, Copes R. Risk of incident diabetes in relation to long-term exposure to fine particulate matter in Ontario, Canada. Environ Health Perspect. 2013; 121(7): 804–10.	Ambient particulate matter pollution and household air pollution
Chen Y, Zhang XP, Yuan J, Cai B, Wang XL, Wu XL, Zhang YH, Zhang XY, Yin T, Zhu XH, Gu YJ, Cui SW, Lu ZQ, Li XY. Association of body mass index and age with incident diabetes in Chinese adults: a population-based cohort study. BMJ Open. 2018; 8(9): e021768.	High body-mass index
Chinese Center for Disease Control and Prevention (CCDC), Ministry of Health (China). China Vital Registration - Death Counts by Cause, County, Age, and Daily Temperature 2015.	High and low temperature
Chinese Center for Disease Control and Prevention (CCDC), Ministry of Health (China). China Vital Registration - Death Counts by Cause, County, Age, and Daily Temperature 2016.	High and low temperature
Clark C, Sbihi H, Tamburic L, Brauer M, Frank LD, Davies HW. Association of Long-Term Exposure to Transportation Noise and Traffic-Related Air Pollution with the Incidence of Diabetes: A Prospective Cohort Study. Environ Health Perspect. 2017; 125(8): 087025.	Ambient particulate matter pollution and household air pollution
Colditz GA, Willett WC, Rotnitzky A, Manson JE. Weight gain as a risk factor for clinical diabetes mellitus in women. Ann Intern Med. 1995; 122(7): 481-6.	High body-mass index
Consortium TI, Spijkerman AMW, A DL van der, Nilsson PM, Ardanaz E, Gavrila D, Agudo A, Arriola L, Balkau B, Beulens JW, Boeing H, Lauzon-Guillain B de, Fagherazzi G, Feskens EJM, Franks PW, Grioni S, Huerta JM, Kaaks R, Key TJ, Overvad K, Palli D, Panico S, Redondo ML, Rolandsson O, Roswall N, Sacerdote C, Sánchez M-J, Schulze MB, Slimani N, Teucher B, Tjonneland A, Tumino R, Schouw YT van der, Langenberg C, Sharp SJ, Forouhi NG, Riboli E, Wareham NJ. Smoking and Long-Term Risk of Type 2 Diabetes: The EPIC-InterAct Study in European Populations. Diabetes Care. 2014; 37(12): 3164–71.	Smoking
Conway BN, Han X, Munro HM, Gross AL, Shu XO, Hargreaves MK, Zheng W, Powers AC, Blot WJ. The obesity epidemic and rising diabetes incidence in a low-income racially diverse southern US cohort. PLoS One. 2018; 13(1): e0190993.	High alcohol use, High body-mass index
Coogan PF, White LF, Yu J, Burnett RT, Seto E, Brook RD, Palmer JR, Rosenberg L, Jerrett M. PM2.5 and Diabetes and Hypertension Incidence in the Black Women's Health Study. Epidemiology. 2016; 27(2): 202–10.	Ambient particulate matter pollution and household air pollution

Cooper AJ, Forouhi NG, Ye Z, Buijsse B, Arriola L, Balkau B, Barricarte A, Beulens JW, Boeing H, Büchner FL, Dahm CC, de Lauzon-Guillain B, Fagherazzi G, Franks PW, Gonzalez C, Grioni S, Kaaks R, Key TJ, Masala G, Navarro C, Nilsson P, Overvad K, Panico S, Ramón Quirós J, Rolandsson O, Roswall N, Sacerdote C, Sánchez MJ, Slimani N, Sluijs I, Spijkerman AM, Teucher B, Tjonneland A, Tumino R, Sharp SJ, Langenberg C, Feskens EJ, Riboli E, Wareham NJ, InterAct Consortium. Fruit and vegetable intake and type 2 diabetes: EPIC-InterAct prospective study and meta-analysis. Eur J Clin Nutr. 2012; 66(10): 1082-92.	Diet low in fruits, Diet low in vegetables
Cooper AJ, Sharp SJ, Lentjes MAH, Luben RN, Khaw K-T, Wareham NJ, Forouhi NG. A Prospective Study of the Association Between Quantity and Variety of Fruit and Vegetable Intake and Incident Type 2 Diabetes. Diabetes Care. 2012; 35(6): 1293–300.	Diet low in fruits
Cullmann M, Hilding A, Ostenson CG. Alcohol consumption and risk of pre-diabetes and type 2 diabetes development in a Swedish population. Diabet Med. 2012; 29(4): 441-52.	High alcohol use
de Koning L, Malik VS, Rimm EB, Willett WC, Hu FB. Sugar-sweetened and artificially sweetened beverage consumption and risk of type 2 diabetes in men. Am J Clin Nutr. 2011; 93(6): 1321-7.	Diet high in sugar-sweetened beverages
de Vegt F, Dekker JM, Groeneveld WJ, Nijpels G, Stehouwer CD, Bouter LM, Heine RJ. Moderate alcohol consumption is associated with lower risk for incident diabetes and mortality: the Hoorn Study. Diabetes Res Clin Pract. 2002; 57(1): 53-60.	High alcohol use
Demakakos P, Hamer M, Stamatakis E, Steptoe A. Low-intensity physical activity is associated with reduced risk of incident type 2 diabetes in older adults: evidence from the English Longitudinal Study of Ageing. Diabetologia. 2010; 53(9): 1877–1885.	Low physical activity
Ding D, Chong S, Jalaludin B, Comino E, Bauman AE. Risk factors of incident type 2-diabetes mellitus over a 3-year follow-up: Results from a large Australian sample. Diabetes Res Clin Pract. 2015; 108(2): 306-15.	High body-mass index, Low physical activity
Djousse L, Biggs ML, Mukamal KJ, Siscovick DS. Alcohol consumption and type 2 diabetes among older adults: the Cardiovascular Health Study. Obesity (Silver Spring). 2007; 15(7): 1758-65.	High alcohol use
Doi Y, Ninomiya T, Hata J, Hirakawa Y, Mukai N, Iwase M, Kiyohara Y. Two risk score models for predicting incident Type 2 diabetes in Japan. Diabet Med. 2012; 29(1): 107-14.	Low physical activity, Smoking
Dow C, Balkau B, Bonnet F, Mancini F, Rajaobelina K, Shaw J, Magliano DJ, Fagherazzi G. Strong adherence to dietary and lifestyle recommendations is associated with decreased type 2 diabetes risk in the AusDiab cohort study. Prev Med. 2019; 123: 208-216.	High alcohol use
Ericson U, Hellstrand S, Brunkwall L, Schulz CA, Sonestedt E, Wallström P, Gullberg B, Wirfält E, Orho-Melander M. Food sources of fat may clarify the inconsistent role of dietary fat intake for incidence of type 2 diabetes. Am J Clin Nutr. 2015; 101(5): 1065-80.	Diet high in processed meat, Diet high in red meat
Ericson U, Sonestedt E, Gullberg B, Hellstrand S, Hindy G, Wirfält E, Orho-Melander M. High intakes of protein and processed meat associate with increased incidence of type 2 diabetes. Br J Nutr. 2013; 109(6): 1143–53.	Diet low in whole grains
Eshak ES, Iso H, Mizoue T, Inoue M, Noda M, Tsugane S. Soft drink, 100% fruit juice, and vegetable juice intakes and risk of diabetes mellitus. Clin Nutr. 2013; 32(2): 300–8.	Diet high in sugar-sweetened beverages
Etemadi A, Sinha R, Ward MH, Graubard BI, Inoue-Choi M, Dawsey SM, Abnet CC. Mortality from different causes associated with meat, heme iron, nitrates, and nitrites in the NIH-AARP Diet and Health Study: population based cohort study. BMJ. 2017; 357: j1957.	Diet high in red meat
Fagherazzi G, Vilier A, Saes Sartorelli D, Lajous M, Balkau B, Clavel-Chapelon F. Consumption of artificially and sugar-sweetened beverages and incident type 2 diabetes in the Etude Epidemiologique aupres des femmes de la Mutuelle Generale de l'Education Nationale-European Prospective Investigation into Cancer and Nutrition cohort. Am J Clin Nutr. 2013; 97(3): 517-23.	Diet high in sugar-sweetened beverages
Feskens EJ, Kromhout D. Cardiovascular risk factors and the 25-year incidence of diabetes mellitus in middle-aged men. The Zutphen Study. Am J Epidemiol. 1989; 130(6): 1101-8.	High alcohol use

Folsom AR, Kushi LH, Anderson KE, Mink PJ, Olson JE, Hong CP, Sellers TA, Lazovich D, Prineas RJ. Associations of general and abdominal obesity with multiple health outcomes in older women: the Iowa Women's Health Study. Arch Intern Med. 2000; 160(14): 2117-28.	High body-mass index
Folsom AR, Kushi LH, Hong CP. Physical activity and incident diabetes mellitus in postmenopausal women. Am J Public Health. 2000; 90(1): 134-8.	Low physical activity
Ford ES, Williamson DF, Liu S. Weight change and diabetes incidence: findings from a national cohort of US adults. Am J Epidemiol. 1997; 146(3): 214-22.	High body-mass index
Fretts AM, Howard BV, Kriska AM, Smith NL, Lumley T, Lee ET, Russell M, Siscovick D. Physical activity and incident diabetes in American Indians: the Strong Heart Study. Am J Epidemiol. 2009; 170(5): 632-9.	Low physical activity
Fretts AM, Howard BV, McKnight B, Duncan GE, Beresford SA, Mete M, Eilat-Adar S, Zhang Y, Siscovick DS. Associations of processed meat and unprocessed red meat intake with incident diabetes: the Strong Heart Family Study. Am J Clin Nutr. 2012; 95(3): 752-8.	Diet high in processed meat, Diet high in red meat
Fujita M, Ueno K, Hata A. Effect of obesity on incidence of type 2 diabetes declines with age among Japanese women. Exp Biol Med (Maywood). 2009; 234(7): 750-7.	High body-mass index
Fung TT, Hu FB, Pereira MA, Liu S, Stampfer MJ, Colditz GA, Willett WC. Whole-grain intake and the risk of type 2 diabetes: a prospective study in men. Am J Clin Nutr. 2002; 76(3): 535-40.	Diet low in whole grains
Golozar A, Khalili D, Etemadi A, Poustchi H, Fazeltabar A, Hosseini F, Kamangar F, Khoshnia M, Islami F, Hadaegh F, Brennan P, Boffetta P, Abnet CC, Dawsey SM, Azizi F, Malekzadeh R, Danaei G. White rice intake and incidence of type-2 diabetes: analysis of two prospective cohort studies from Iran. BMC Public Health. 2017; 17(1): 133.	Diet low in whole grains
Grøntved A, Pan A, Mekary RA, Stampfer M, Willett WC, Manson JE, Hu FB. Muscle-strengthening and conditioning activities and risk of type 2 diabetes: a prospective study in two cohorts of US women. PLoS Med. 2014; 11(1): e1001587.	Low physical activity
Grøntved A, Rimm EB, Willett WC, Andersen LB, Hu FB. A prospective study of weight training and risk of type 2 diabetes mellitus in men. Arch Intern Med. 2012; 172(17): 1306-12.	Low physical activity
Gurwitz JH, Field TS, Glynn RJ, Manson JE, Avorn J, Taylor JO, Hennekens CH. Risk factors for non-insulin-dependent diabetes mellitus requiring treatment in the elderly. J Am Geriatr Soc. 1994; 42(12): 1235-40.	High alcohol use
Hadaegh F, Bozorgmanesh M, Safarkhani M, Khalili D, Azizi F. Predictability of body mass index for diabetes: affected by the presence of metabolic syndrome?. BMC Public Health. 2011; 11: 383.	High body-mass index
Han C, Liu Y, Sun X, Luo X, Zhang L, Wang B, Ren Y, Zhou J, Zhao Y, Zhang D, Liu X, Zhang M, Hu D. Prediction of a new body shape index and body adiposity estimator for development of type 2 diabetes mellitus: The Rural Chinese Cohort Study. Br J Nutr. 2017; 118(10): 771-776.	High body-mass index
Han T, Zhang S, Duan W, Ren X, Wei C, Sun C, Li Y. Eighteen-year alcohol consumption trajectories and their association with risk of type 2 diabetes and its related factors: the China Health and Nutrition Survey. Diabetologia. 2019; 62(6): 970-980.	High alcohol use
Hansen AB, Ravnskjær L, Loft S, Andersen KK, Bräuner EV, Baastrup R, Yao C, Ketzel M, Becker T, Brandt J, Hertel O, Andersen ZJ. Long-term exposure to fine particulate matter and incidence of diabetes in the Danish Nurse Cohort. Environ Int. 2016; 91: 243–50.	Ambient particulate matter pollution and household air pollution
Hart CL, Hole DJ, Lawlor DA, Davey Smith G. How many cases of Type 2 diabetes mellitus are due to being overweight in middle age? Evidence from the Midspan prospective cohort studies using mention of diabetes mellitus on hospital discharge or death records. Diabet Med. 2007; 24(1): 73-80.	High body-mass index
Hayashino Y, Fukuhara S, Okamura T, Yamato H, Tanaka H, Tanaka T, Kadowaki T, Ueshima H; HIPOP-OHP Research Group. A prospective study of passive smoking and risk of diabetes in a cohort of workers: the High-Risk and Population Strategy for Occupational Health Promotion (HIPOP-OHP) study. Diabetes Care. 2008; 31(4): 732-4.	Second-hand smoke

He X, Rebholz CM, Daya N, Lazo M, Selvin E. Alcohol consumption and incident diabetes: The Atherosclerosis Risk in Communities (ARIC) study. Diabetologia. 2019; 62(5): 770-778.	High alcohol use
Heianza Y, Arase Y, Saito K, Tsuji H, Fujihara K, Hsieh SD, Kodama S, Shimano H, Yamada N, Hara S, Sone H. Role of alcohol drinking pattern in type 2 diabetes in Japanese men: the Toranomon Hospital Health Management Center Study 11 (TOPICS 11). Am J Clin Nutr. 2013; 97(3): 561-8.	High alcohol use
Hilawe EH, Yatsuya H, Li Y, Uemura M, Wang C, Chiang C, Toyoshima H, Tamakoshi K, Zhang Y, Kawazoe N, Aoyama A. Smoking and diabetes: is the association mediated by adiponectin, leptin, or C-reactive protein?. J Epidemiol. 2015; 25(2): 99–109.	Smoking
Hinnouho GM, Czernichow S, Dugravot A, Nabi H, Brunner EJ, Kivimaki M, Singh-Manoux A. Metabolically healthy obesity and the risk of cardiovascular disease and type 2 diabetes: the Whitehall II cohort study. Eur Heart J. 2015; 36(9): 551-9.	High body-mass index
Hodge AM, Dowse GK, Zimmet PZ. Diet does not predict incidence or prevalence of non-insulin-dependent diabetes in Nauruans. Asia Pac J Clin Nutr. 1993; 2(1): 35-41.	Diet low in fiber
Hodge AM, English DR, O'Dea K, Giles GG. Alcohol intake, consumption pattern and beverage type, and the risk of Type 2 diabetes. Diabet Med. 2006; 23(6): 690-7.	High alcohol use
Hodge AM, English DR, O'Dea K, Giles GG. Glycemic index and dietary fiber and the risk of type 2 diabetes. Diabetes Care. 2004; 27(11): 2701-6.	Diet low in fiber, Diet low in fruits, Diet low in vegetables, Diet low in whole grains
Honda T, Kuwahara K, Nakagawa T, Yamamoto S, Hayashi T, Mizoue T. Leisure-time, occupational, and commuting physical activity and risk of type 2 diabetes in Japanese workers: a cohort study. BMC Public Health. 2015; 15: 1004.	Low physical activity
Honda T, Pun VC, Manjourides J, Suh H. Associations between long-term exposure to air pollution, glycosylated hemoglobin and diabetes. Int J Hyg Environ Health. 2017; 220(7): 1124–32.	Ambient particulate matter pollution and household air pollution
Hopping BN, Erber E, Grandinetti A, Verheus M, Kolonel LN, Maskarinec G. Dietary fiber, magnesium, and glycemic load alter risk of type 2 diabetes in a multiethnic cohort in Hawaii. J Nutr. 2010; 140(1): 68-74.	Diet low in fiber
Hou X, Qiu J, Chen P, Lu J, Ma X, Lu J, Weng J, Ji L, Shan Z, Liu J, Tian H, Ji Q, Zhu D, Ge J, Lin L, Chen L, Guo X, Zhao Z, Li Q, Zhou Z, Yang W, Jia W, Group CNDMDS. Cigarette Smoking Is Associated with a Lower Prevalence of Newly Diagnosed Diabetes Screened by OGTT than Non-Smoking in Chinese Men with Normal Weight. PLoS One. 2016; 11(3): e0149234.	Smoking
Houston TK, Person SD, Pletcher MJ, Liu K, Iribarren C, Kiefe CI. Active and passive smoking and development of glucose intolerance among young adults in a prospective cohort: CARDIA study. BMJ. 2006; 332(7549): 1064-9.	Second-hand smoke
Hsia J, Wu L, Allen C, Oberman A, Lawson WE, Torréns J, Safford M, Limacher MC, Howard BV, Women's Health Initiative Research Group. Physical activity and diabetes risk in postmenopausal women. Am J Prev Med. 2005; 28(1): 19-25.	Low physical activity
Hu FB, Manson JE, Stampfer MJ, Colditz G, Liu S, Solomon CG, Willett WC. Diet, lifestyle, and the risk of type 2 diabetes mellitus in women. N Engl J Med. 2001; 345(11): 790-7.	High alcohol use, Smoking
Hu FB, Sigal RJ, Rich-Edwards JW, Colditz GA, Solomon CG, Willett WC, Speizer FE, Manson JE. Walking compared with vigorous physical activity and risk of type 2 diabetes in women: a prospective study. JAMA. 1999; 282(15): 1433-9.	Low physical activity
Hu G, Jousilahti P, Peltonen M, Bidel S, Tuomilehto J. Joint association of coffee consumption and other factors to the risk of type 2 diabetes: a prospective study in Finland. Int J Obes (Lond). 2006; 30(12): 1742-9.	High alcohol use
Hu G, Lindström J, Valle TT, Eriksson JG, Jousilahti P, Silventoinen K, Qiao Q, Tuomilehto J. Physical activity, body mass index, and risk of type 2 diabetes in patients with normal or impaired glucose regulation. Arch Intern Med. 2004; 164(8): 892-6.	High body-mass index

Hwang LC, Bai CH, Sun CA, Chen CJ. Prevalence of metabolically healthy obesity and its impacts on incidences of hypertension, diabetes and the metabolic syndrome in Taiwan. Asia Pac J Clin Nutr. 2012; 21(2): 227-33.	High body-mass index
Hwang LC, Chen SC, Chen CJ. Increased risk of mortality from overweight and obesity in middle-aged individuals from six communities in Taiwan. J Formos Med Assoc. 2011; 110(5): 290-8.	High body-mass index
Hystad P, Duong M, Brauer M, Larkin A, Arku R, Kurmi OP, Fan WQ, Avezum A, Azam I, Chifamba J, Dans A, du Plessis JL, Gupta R, Kumar R, Lanas F, Liu Z, Lu Y, Lopez-Jaramillo P, Mony P, Mohan V, Mohan D, Nair S, Puoane T, Rahman O, Lap AT, Wang Y, Wei L, Yeates K, Rangarajan S, Teo K, Yusuf S, on behalf of Prospective Urban and Rural Epidemiological (PURE) Study investigators. Health Effects of Household Solid Fuel Use: Findings from 11 Countries within the Prospective Urban and Rural Epidemiology Study [Unpublished]. Environ Health Perspect. 2019; 127(5): 57003.	Ambient particulate matter pollution and household air pollution
Imamura F, Lichtenstein AH, Dallal GE, Meigs JB, Jacques PF. Confounding by dietary patterns of the inverse association between alcohol consumption and type 2 diabetes risk. Am J Epidemiol. 2009; 170(1): 37-45.	High alcohol use
InterAct Consortium, Bendinelli B, Palli D, Masala G, Sharp SJ, Schulze MB, Guevara M, van der AD, Sera F, Amiano P, Balkau B, Barricarte A, Boeing H, Crowe FL, Dahm CC, Dalmeijer G, de Lauzon-Guillain B, Egeberg R, Fagherazzi G, Franks PW, Krogh V, Huerta JM, Jakszyn P, Khaw KT, Li K, Mattiello A, Nilsson PM, Overvad K, Ricceri F, Rolandsson O, Sánchez MJ, Slimani N, Sluijs I, Spijkerman AM, Teucher B, Tjonneland A, Tumino R, van den Berg SW, Forouhi NG, Langeberg C, Feskens EJ, Riboli E, Wareham NJ. Association between dietary meat consumption and incident type 2 diabetes: the EPIC-InterAct study. Diabetologia. 2013; 56(1): 47-59.	Diet high in processed meat, Diet high in red meat
InterAct Consortium, Langenberg C, Sharp SJ, Schulze MB, Rolandsson O, Overvad K, Forouhi NG, Spranger J, Drogan D, Huerta JM, Arriola L, de Lauzon-Guillan B, Tormo MJ, Ardanaz E, Balkau B, Beulens JW, Boeing H, Bueno-de-Mesquita HB, Clavel-Chapelon F, Crowe FL, Franks PW, Gonzalez CA, Grioni S, Halkjaer J, Hallmans G, Kaaks R, Kerrison ND, Key TJ, Khaw KT, Mattiello A, Nilsson P, Norat T, Palla L, Palli D, Panico S, Quirós JR, Romaguera D, Romieu I, Sacerdote C, Sánchez MJ, Slimani N, Sluijs I, Spijkerman AM, Teucher B, Tjonneland A, Tumino R, van der A DL, van der Schouw YT, Feskens EJ, Riboli E, Wareham NJ. Long-term risk of incident type 2 diabetes and measures of overall and regional obesity: the EPIC-InterAct case-cohort study. PLoS Med. 2012; 9(6): e1001230.	High body-mass index
InterAct Consortium. Dietary fibre and incidence of type 2 diabetes in eight European countries: the EPIC-InterAct Study and a meta-analysis of prospective studies. Diabetologia. 2015; 58(7): 1394-408.	Diet low in fiber
InterAct Consortium., Romaguera D, Norat T, Wark PA, Vergnaud AC, Schulze MB, van Woudenbergh GJ, Drogan D, Amiano P, Molina-Montes E, Sánchez MJ, Balkau B, Barricarte A, Beulens JW, Clavel-Chapelon F, Crispim SP, Fagherazzi G, Franks PW, Grote VA, Huybrechts I, Kaaks R, Key TJ, Khaw KT, Nilsson P, Overvad K, Palli D, Panico S, Quirós JR, Rolandsson O, Sacerdote C, Sieri S, Slimani N, Spijkerman AM, Tjonneland A, Tormo MJ, Tumino R, van den Berg SW, Wermeling PR, Zamara-Ros R, Feskens EJ, Langenberg C, Sharp SJ, Forouhi NG, Riboli E, Wareham NJ. Consumption of sweet beverages and type 2 diabetes incidence in European adults: results from EPIC-InterAct. Diabetologia. 2013; 56(7): 1520-30.	Diet high in sugar-sweetened beverages
Ishikawa-Takata K, Ohta T, Moritaki K, Gotou T, Inoue S. Obesity, weight change and risks for hypertension, diabetes and hypercholesterolemia in Japanese men. Eur J Clin Nutr. 2002; 56(7): 601-7.	High body-mass index
Janssen I. Morbidity and mortality risk associated with an overweight BMI in older men and women. Obesity (Silver Spring). 2007; 15(7): 1827-40.	High body-mass index
Jee SH, Foong AW, Hur NW, Samet JM. Smoking and risk for diabetes incidence and mortality in Korean men and women. Diabetes Care. 2010; 33(12): 2567-72.	High alcohol use, Smoking
Joosten MM, Chiuve SE, Mukamal KJ, Hu FB, Hendriks HF, Rimm EB. Changes in alcohol consumption and subsequent risk of type 2 diabetes in men. Diabetes. 2011; 60(1): 74-9.	High alcohol use
Joosten MM, Grobbee DE, van der A DL, Verschuren WM, Hendriks HF, Beulens JW. Combined effect of alcohol consumption and lifestyle behaviors on risk of type 2 diabetes. Am J Clin Nutr. 2010; 91(6): 1777-83.	High alcohol use
Joseph J, Svartberg J, Njolstad I, Schirmer H. Incidence of and risk factors for type-2 diabetes in a general population: the Tromso Study. Scand J Public Health. 2010; 38(7): 768-75.	Low physical activity

Joseph JJ, Echouffo-Tcheugui JB, Carnethon MR, Bertoni AG, Shay CM, Ahmed HM, Blumenthal RS, Cushman M, Golden SH. The association of ideal cardiovascular health with incident type 2 diabetes mellitus: the Multi-Ethnic Study of Atherosclerosis. Diabetologia. 2016; 59(9): 1893-903.	Low physical activity
Jung HS, Chang Y, Eun Yun K, Kim CW, Choi ES, Kwon MJ, Cho J, Zhang Y, Rampal S, Zhao D, Soo Kim H, Shin H, Guallar E, Ryu S. Impact of body mass index, metabolic health and weight change on incident diabetes in a Korean population. Obesity (Silver Spring). 2014; 22(8): 1880-7.	High body-mass index
Kawakami N, Takatsuka N, Shimizu H, Ishibashi H. Effects of smoking on the incidence of non-insulin-dependent diabetes mellitus. Replication and extension in a Japanese cohort of male employees. Am J Epidemiol. 1997; 145(2): 103-9.	High alcohol use, Smoking
Kerr WC, Williams E, Li L, Lui CK, Ye Y, Greenfield TK, Lown EA. Alcohol use patterns and risk of diabetes onset in the 1979 National Longitudinal Survey of Youth Cohort. Prev Med. 2018; 109: 22-27.	High alcohol use
Kim C, Seow WJ, Shu X-O, Bassig BA, Rothman N, Chen BE, Xiang Y-B, Hosgood HD, Ji B-T, Hu W, Wen C, Chow W-H, Cai Q, Yang G, Gao Y-T, Zheng W, Lan Q. Cooking Coal Use and All-Cause and Cause-Specific Mortality in a Prospective Cohort Study of Women in Shanghai, China. Environ Health Perspect. 2016; 124(9): 1384–9.	Ambient particulate matter pollution and household air pollution
Ko KP, Min H, Ahn Y, Park SJ, Kim CS, Park JK, Kim SS. A prospective study investigating the association between environmental tobacco smoke exposure and the incidence of type 2 diabetes in never smokers. Ann Epidemiol. 2011; 21(1): 42-7.	Second-hand smoke
Koloverou E, Panagiotakos DB, Pitsavos C, Chrysohoou C, Georgousopoulou EN, Metaxa V, Stefanadis C. Effects of alcohol consumption and the metabolic syndrome on 10-year incidence of diabetes: the ATTICA study. Diabetes Metab. 2015; 41(2): 152-9.	High alcohol use
Kotronen A, Laaksonen MA, Heliövaara M, Reunanen A, Tuomilehto J, Yki-Järvinen H, Peltonen M, Knekt P. Fatty liver score and 15-year incidence of type 2 diabetes. Hepatol Int. 2013; 7(2): 610-21.	High alcohol use
Kowall B, Rathmann W, Strassburger K, Heier M, Holle R, Thorand B, Giani G, Peters A, Meisinger C. Association of passive and active smoking with incident type 2 diabetes mellitus in the elderly population: the KORA S4/F4 cohort study. Eur J Epidemiol. 2010; 25(6): 393-402.	Second-hand smoke
Krishnan S, Rosenberg L, Djoussé L, Cupples LA, Palmer JR. Overall and central obesity and risk of type 2 diabetes in U.S. black women. Obesity (Silver Spring). 2007; 15(7): 1860-6.	High body-mass index
Krishnan S, Rosenberg L, Palmer JR. Physical activity and television watching in relation to risk of type 2 diabetes: the Black Women's Health Study. Am J Epidemiol. 2009; 169(4): 428-34.	Low physical activity
Krishnan S, Rosenberg L, Singer M, Hu FB, Djoussé L, Cupples LA, Palmer JR. Glycemic index, glycemic load, and cereal fiber intake and risk of type 2 diabetes in US black women. Arch Intern Med. 2007; 167(21): 2304-9.	Diet low in fiber
Kurotani K, Nanri A, Goto A, Mizoue T, Noda M, Kato M, Inoue M, Tsugane S, Group for the JPHCPS. Vegetable and fruit intake and risk of type 2 diabetes: Japan Public Health Center-based Prospective Study. Br J Nutr. 2013; 109(4): 709–17.	Diet low in fruits, Diet low in vegetables
Kurotani K, Nanri A, Goto A, Mizoue T, Noda M, Oba S, Kato M, Matsushita Y, Inoue M, Tsugane S, Japan Public Health Center-based Prospective Study Group. Red meat consumption is associated with the risk of type 2 diabetes in men but not in women: a Japan Public Health Center-based Prospective Study. Br J Nutr. 2013; 110(10): 1910-8.	Diet high in processed meat, Diet high in red meat
Laaksonen MA, Knekt P, Rissanen H, Härkänen T, Virtala E, Marniemi J, Aromaa A, Heliövaara M, Reunanen A. The relative importance of modifiable potential risk factors of type 2 diabetes: a meta-analysis of two cohorts. Eur J Epidemiol. 2010; 25(2): 115-24.	Low physical activity
Lacoppidan SA, Kyrø C, Loft S, Helnæs A, Christensen J, Hansen CP, Dahm CC, Overvad K, Tjønneland A, Olsen A. Adherence to a Healthy Nordic Food Index Is Associated with a Lower Risk of Type-2 Diabetes-The Danish Diet, Cancer and Health Cohort Study. Nutrients. 2015; 7(10): 8633-44.	Diet low in fruits

Lajous M, Tondeur L, Fagherazzi G, de Lauzon-Guillain B, Boutron-Ruaualt MC, Clavel-Chapelon F. Processed and unprocessed red meat consumption and incident type 2 diabetes among French women. Diabetes Care. 2012; 35(1): 128-30.	Diet high in red meat
Lee D, Park I, Jun TW, Nam BH, Cho S, Blair SN, Kim YS. Physical activity and body mass index and their associations with the development of type 2 diabetes in korean men. Am J Epidemiol. 2012; 176(1): 43–51.	Low physical activity
Lee DH, Ha MH, Kim JH, Christiani DC, Gross MD, Steffes M, Blomhoff R, Jacobs DR. Gamma-glutamyltransferase and diabetes--a 4 year follow-up study. Diabetologia. 2003; 46(3): 359-64.	High alcohol use
Lee DY, Yoo MG, Kim HJ, Jang HB, Kim JH, Lee HJ, Park SI. Association between alcohol consumption pattern and the incidence risk of type 2 diabetes in Korean men: A 12-years follow-up study. Sci Rep. 2017; 7(1): 7322.	High alcohol use
Lim CC, Hayes RB, Ahn J, Shao Y, Silverman DT, Jones RR, Garcia C, Thurston GD. Association between long-term exposure to ambient air pollution and diabetes mortality in the US. Environ Res. 2018; 165: 330-336.	Ambient particulate matter pollution and household air pollution
Lindström J, Peltonen M, Eriksson JG, Louheranta A, Fogelholm M, Uusitupa M, Tuomilehto J. High-fibre, low-fat diet predicts long-term weight loss and decreased type 2 diabetes risk: the Finnish Diabetes Prevention Study. Diabetologia. 2006; 49(5): 912-20.	Diet low in fiber
Liu M, Liu C, Zhang Z, Zhou C, Li Q, He P, Zhang Y, Li H, Qin X. Quantity and variety of food groups consumption and the risk of diabetes in adults: A prospective cohort study. Clin Nutr. 2021; 40(12): 5710-5717.	Diet low in vegetables
Liu M, Tang R, Wang J, He Y. Distribution of metabolic/obese phenotypes and association with diabetes: 5 years' cohort based on 22,276 elderly. Endocrine. 2018; 62(1): 107-115.	High body-mass index
Liu S, Manson JE, Stampfer MJ, Hu FB, Giovannucci E, Colditz GA, Hennekens CH, Willett WC. A prospective study of whole-grain intake and risk of type 2 diabetes mellitus in US women. Am J Public Health. 2000; 90(9): 1409-15.	Diet low in whole grains
Liu S, Serdula M, Janket S-J, Cook NR, Sesso HD, Willett WC, Manson JE, Buring JE. A Prospective Study of Fruit and Vegetable Intake and the Risk of Type 2 Diabetes in Women. Diabetes Care. 2004; 27(12): 2993–6.	Diet low in fruits, Diet low in vegetables
Lv J, Yu C, Guo Y, Bian Z, Yang L, Chen Y, Hu X, Hou W, Chen J, Chen Z, Qi L, Li L, China Kadoorie Biobank Collaborative Group. Adherence to a Healthy Lifestyle and the Risk of Type 2 Diabetes in Chinese Adults. Int J Epidemiol. 2017; 46(5): 1410-20.	High alcohol use, Smoking
Magliano DJ, Barr EL, Zimmet PZ, Cameron AJ, Dunstan DW, Colagiuri S, Jolley D, Owen N, Phillips P, Tapp RJ, Welborn TA, Shaw JE. Glucose indices, health behaviors, and incidence of diabetes in Australia: the Australian Diabetes, Obesity and Lifestyle Study. Diabetes Care. 2008; 31(2): 267-72.	Diet high in processed meat, Diet high in red meat
Mamluk L, O'Doherty MG, Orfanos P, Saitakis G, Woodside JV, Liao LM, Sinha R, Boffetta P, Trichopoulou A, Kee F. Fruit and vegetable intake and risk of incident of type 2 diabetes: results from the consortium on health and ageing network of cohorts in Europe and the United States (CHANCES). Eur J Clin Nutr. 2017; 71(1): 83-91.	Low physical activity
Männistö S, Kontto J, Kataja-Tuomola M, Albanes D, Virtamo J. High processed meat consumption is a risk factor of type 2 diabetes in the Alpha-Tocopherol, Beta-Carotene Cancer Prevention study. Br J Nutr. 2010; 103(12): 1817-22.	Diet low in fruits, Diet low in vegetables
Manson JE, Ajani UA, Liu S, Nathan DM, Hennekens CH. A prospective study of cigarette smoking and the incidence of diabetes mellitus among US male physicians. Am J Med. 2000; 109(7): 538-42.	Smoking
Manson JE, Rimm EB, Stampfer MJ, Colditz GA, Willett WC, Krolewski AS, Rosner B, Hennekens CH, Speizer FE. Physical activity and incidence of non-insulin-dependent diabetes mellitus in women. Lancet. 1991; 338(8770): 774-8.	Low physical activity
Mari-Sanchis A, Gea A, Basterra-Gortari FJ, Martinez-Gonzalez MA, Beunza JJ, Bes-Rastrollo M. Meat Consumption and Risk of Developing Type 2 Diabetes in the SUN Project: A Highly Educated Middle-Class Population. PLoS One. 2016; 11(7): e0157990.	Diet high in processed meat
Marques-Vidal P, Vollenweider P, Waeber G. Alcohol consumption and incidence of type 2 diabetes. Results from the CoLaus study. Nutr Metab Cardiovasc Dis. 2015; 25(1): 75–84.	High alcohol use

Maty SC, James SA, Kaplan GA. Life-course socioeconomic position and incidence of diabetes mellitus among blacks and whites: the Alameda County Study, 1965-1999. 2010; 100(1): 137-45.	High alcohol use
Maty SC, Lynch JW, Raghunathan TE, Kaplan GA. Childhood socioeconomic position, gender, adult body mass index, and incidence of type 2 diabetes mellitus over 34 years in the Alameda County Study. Am J Public Health. 2008; 98(8): 1486-94.	High alcohol use
Meisinger C, Döring A, Thorand B, Heier M, Löwel H. Body fat distribution and risk of type 2 diabetes in the general population: are there differences between men and women? The MONICA/KORA Augsburg cohort study. Am J Clin Nutr. 2006; 84(3): 483-9.	High body-mass index
Meisinger C, Doring A, Thorand B, Lowel H. Association of cigarette smoking and tar and nicotine intake with development of type 2 diabetes mellitus in men and women from the general population: the MONICA/KORA Augsburg Cohort Study. Diabetologia. 2006; 49(8): 1770-6.	Smoking
Meisinger C, Löwel H, Thorand B, Döring A. Leisure time physical activity and the risk of type 2 diabetes in men and women from the general population. Diabetologia. 2005; 48(1): 27-34.	Low physical activity
Meisinger C, Thorand B, Schneider A, Stieber J, Doring A, Lowel H. Sex differences in risk factors for incident type 2 diabetes mellitus: the MONICA Augsburg cohort study. Arch Intern Med. 2002; 162(1): 82-9.	High alcohol use
Meyer KA, Kushi LH, Jacobs DR, Slavin J, Sellers TA, Folsom AR. Carbohydrates, dietary fiber, and incident type 2 diabetes in older women. Am J Clin Nutr. 2000; 71(4): 921–30.	Diet low in fiber, Diet low in fruits, Diet low in vegetables, Diet low in whole grains
Mishra GD, Carrigan G, Brown WJ, Barnett AG, Dobson AJ. Short-term weight change and the incidence of diabetes in midlife: results from the Australian Longitudinal Study on Women's Health. Diabetes Care. 2007; 30(6): 1418-24.	High body-mass index
Montonen J, Järvinen R, Heliövaara M, Reunanen A, Aromaa A, Knekt P. Food consumption and the incidence of type II diabetes mellitus. Eur J Clin Nutr. 2005; 59(3): 441–8.	Diet low in fruits, Diet high in processed meat, Diet high in red meat, Diet low in vegetables
Montonen J, Järvinen R, Knekt P, Heliövaara M, Reunanen A. Consumption of sweetened beverages and intakes of fructose and glucose predict type 2 diabetes occurrence. J Nutr. 2007; 137(6): 1447–54.	Diet high in sugar-sweetened beverages
Montonen J, Knekt P, Järvinen R, Aromaa A, Reunanen A. Whole-grain and fiber intake and the incidence of type 2 diabetes. Am J Clin Nutr. 2003; 77(3): 622–9.	Diet low in fiber, Diet low in whole grains
Morimoto A, Ohno Y, Tatsumi Y, Nishigaki Y, Maejima F, Mizuno S, Watanabe S. Impact of smoking cessation on incidence of diabetes mellitus among overweight or normal-weight Japanese men. Diabetes Res Clin Pract. 2012; 96(3): 407–13.	Smoking
Muraki I, Imamura F, Manson JE, Hu FB, Willett WC, Dam RM van, Sun Q. Fruit consumption and risk of type 2 diabetes: results from three prospective longitudinal cohort studies. BMJ. 2013; 347: f5001.	Diet low in fruits
Mursu J, Virtanen JK, Tuomainen TP, Nurmi T, Voutilainen S. Intake of fruit, berries, and vegetables and risk of type 2 diabetes in Finnish men: the Kuopio Ischaemic Heart Disease Risk Factor Study. Am J Clin Nutr. 2014; 99(2): 328-33.	Diet low in fruits
Nagaya T, Yoshida H, Takahashi H, Kawai M. Increases in body mass index, even within non-obese levels, raise the risk for Type 2 diabetes mellitus: a follow-up study in a Japanese population. Diabet Med. 2005; 22(8): 1107-11.	High body-mass index
Nagaya T, Yoshida H, Takahashi H, Kawai M. Resting heart rate and blood pressure, independent of each other, proportionally raise the risk for type-2 diabetes mellitus. Int J Epidemiol. 2010; 39(1): 215-22.	High alcohol use
Nakanishi N, Nakamura K, Matsuo Y, Suzuki K, Tatara K. Cigarette smoking and risk for impaired fasting glucose and type 2 diabetes in middle-aged Japanese men. Ann Intern Med. 2000; 133(3): 183-91.	Smoking
Nanri A, Mizoue T, Noda M, Takahashi Y, Kato M, Inoue M, Tsugane S, Japan Public Health Center-based Prospective Study Group. Rice intake and type 2 diabetes in Japanese men and women: the Japan Public Health Center-based Prospective Study. Am J Clin Nutr. 2010; 92(6): 1468-77.	Diet low in whole grains

Nanri A, Nakagawa T, Kuwahara K, Yamamoto S, Honda T, Okazaki H, Uehara A, Yamamoto M, Miyamoto T, Kochi T, Eguchi M, Murakami T, Shimizu C, Shimizu M, Tomita K, Nagahama S, Imai T, Nishihara A, Sasaki N, Hori A, Sakamoto N, Nishiura C, Totsuzaki T, Kato N, Fukasawa K, Huanhuan H, Akter S, Kurotani K, Kabe I, Mizoue T, Sone T, Dohi S, Japan Epidemiology Collaboration on Occupational Health Study Group. Development of Risk Score for Predicting 3-Year Incidence of Type 2 Diabetes: Japan Epidemiology Collaboration on Occupational Health Study. PLoS One. 2015; 10(11): e0142779.	High body-mass index
Nemesure B, Wu SY, Hennis A, Leske MC, BES Study Group. The relationship of body mass index and waist-hip ratio on the 9-year incidence of diabetes and hypertension in a predominantly African-origin population. Ann Epidemiol. 2008; 18(8): 657-63.	High body-mass index
Ng R, Sutradhar R, Yao Z, Wodchis WP, Rosella LC. Smoking, drinking, diet and physical activity-modifiable lifestyle risk factors and their associations with age to first chronic disease. Int J Epidemiol. 2019.	High alcohol use
Nguyen B, Bauman A, Ding D. Incident Type 2 Diabetes in a Large Australian Cohort Study: Does Physical Activity or Sitting Time Alter the Risk Associated With Body Mass Index?. J Phys Act Health. 2017; 14(1): 13-19.	High body-mass index
Njølstad I, Arnesen E, Lund-Larsen PG. Sex differences in risk factors for clinical diabetes mellitus in a general population: a 12-year follow-up of the Finnmark Study. Am J Epidemiol. 1998; 147(1): 49-58.	High body-mass index
Oba S, Noda M, Waki K, Nanri A, Kato M, Takahashi Y, Poudel-Tandukar K, Matsushita Y, Inoue M, Mizoue T, Tsugane S, Japan Public Health Center-Based Prospective Study Group. Smoking Cessation Increases Short-Term Risk of Type 2 Diabetes Irrespective of Weight Gain: The Japan Public Health Center-Based Prospective Study. PLoS One. 2012; 7(2): e17061.	Smoking
Odegaard AO, Koh W-P, Arakawa K, Yu MC, Pereira MA. Soft drink and juice consumption and risk of physician-diagnosed incident type 2 diabetes: the Singapore Chinese Health Study. Am J Epidemiol. 2010; 171(6): 701–8.	Diet high in sugar-sweetened beverages
Odegaard AO, Koh WP, Vazquez G, Arakawa K, Lee HP, Yu MC, Pereira MA. BMI and diabetes risk in Singaporean Chinese. Diabetes Care. 2009; 32(6): 1104-6.	High body-mass index
Oguma Y, Sesso HD, Paffenbarger RS Jr, Lee IM. Weight change and risk of developing type 2 diabetes. Obes Res. 2005; 13(5): 945-51.	High body-mass index
Okada K, Hayashi T, Tsumura K, Suematsu C, Endo G, Fujii S. Leisure-time physical activity at weekends and the risk of Type 2 diabetes mellitus in Japanese men: the Osaka Health Survey. Diabet Med. 2000; 17(1): 53-8.	Low physical activity
Olsson K, Ramne S, González-Padilla E, Ericson U, Sonestedt E. Associations of carbohydrates and carbohydrate-rich foods with incidence of type 2 diabetes. Br J Nutr. 2021; 126(7): 1065-1075.	Diet low in vegetables
Onat A, Hergenc G, Kucukdurmaz Z, Ugur M, Kaya Z, Can G, Yuksel H. Moderate and heavy alcohol consumption among Turks: long-term impact on mortality and cardiometabolic risk. Turk Kardiyol Dern Ars. 2009; 37(2): 83-90.	High alcohol use
Pan A, Sun Q, Bernstein AM, Schulze MB, Manson JE, Willett WC, Hu FB. Red meat consumption and risk of type 2 diabetes: 3 cohorts of US adults and an updated meta-analysis. Am J Clin Nutr. 2011; 94(4): 1088-96.	Diet high in processed meat, Diet high in red meat
Panagiotakos DB, Pitsavos C, Skoumas Y, Lentzas Y, Stefanadis C. Five-year incidence of type 2 diabetes mellitus among cardiovascular disease-free Greek adults: findings from the ATTICA study. Vasc Health Risk Manag. 2008; 4(3): 691–8.	Low physical activity
Papier K, Fensom GK, Knuppel A, Appleby PN, Tong TYN, Schmidt JA, Travis RC, Key TJ, Perez-Cornago A. Meat consumption and risk of 25 common conditions: outcome-wide analyses in 475,000 men and women in the UK Biobank study. BMC Med. 2021; 19(1): 53.	Diet high in red meat
Papier K, Jordan S, D'Este C, Bain C, Peungson J, Banwell C, Yiengprugsawan V, Seubsman SA, Sleigh A. Incidence and risk factors for type 2 diabetes mellitus in transitional Thailand: results from the Thai cohort study. BMJ Open. 2016; 6(12): e014102.	High body-mass index
Park CH, Ga H, Leem JH, Kwak SM, Kim HC, Choi JH. [The effect of smoking status upon occurrence of impaired fasting glucose or type 2 diabetes in Korean men]. J Prev Med Public Health. 2008; 41(4): 249-54.	Smoking

Park SK, Adar SD, O'Neill MS, Auchincloss AH, Szpiro A, Bertoni AG, Navas-Acien A, Kaufman JD, Diez-Roux AV. Long-term exposure to air pollution and type 2 diabetes mellitus in a multiethnic cohort. Am J Epidemiol. 2015; 181(5): 327–36.	Ambient particulate matter pollution and household air pollution
Parker ED, Liu S, Van Horn L, Tinker LF, Shikany JM, Eaton CB, Margolis KL. The association of whole grain consumption with incident type 2 diabetes: the Women's Health Initiative Observational Study. Ann Epidemiol. 2013; 23(6): 321–7.	Diet low in whole grains
Patja K, Jousilahti P, Hu G, Valle T, Qiao Q, Tuomilehto J. Effects of smoking, obesity and physical activity on the risk of type 2 diabetes in middle-aged Finnish men and women. J Intern Med. 2005; 258(4): 356-62.	High body-mass index, Smoking
Paynter NP, Yeh H-C, Voutilainen S, Schmidt MI, Heiss G, Folsom AR, Brancati FL, Kao WHL. Coffee and sweetened beverage consumption and the risk of type 2 diabetes mellitus: the atherosclerosis risk in communities study. Am J Epidemiol. 2006; 164(11): 1075–84.	Diet high in sugar-sweetened beverages
Pirie K, Peto R, Reeves GK, Green J, Beral V, Million Women Study Collaborators. The 21st century hazards of smoking and benefits of stopping: a prospective study of one million women in the UK. Lancet. 2013; 381(9861): 133–41.	Smoking
Qiao Y, Tinker L, Olendzki BC, Hébert JR, Balasubramanian R, Rosal MC, Hingle M, Song Y, Schneider KL, Liu S, Sims S, Ockene JK, Sepavich DM, Shikany JM, Persuitte G, Ma Y. Racial/ethnic disparities in association between dietary quality and incident diabetes in postmenopausal women in the United States: the Women's Health Initiative 1993-2005. Ethn Health. 2014; 19(3): 328-47.	Diet low in fiber, Diet low in fruits, Diet low in vegetables
Qiu H, Schooling CM, Sun S, Tsang H, Yang Y, Lee RS, Wong CM, Tian L. Long-term exposure to fine particulate matter air pollution and type 2 diabetes mellitus in elderly: A cohort study in Hong Kong. Environ Int. 2018; 113: 350-356.	Ambient particulate matter pollution and household air pollution
Radzeviciene L, Ostrauskas R. Smoking habits and type 2 diabetes mellitus in women. Women Health. 2018; 58(8): 884-897.	Smoking
Rasouli B, Ahlbom A, Andersson T, Grill V, Midthjell K, Olsson L, Carlsson S. Alcohol consumption is associated with reduced risk of Type 2 diabetes and autoimmune diabetes in adults: results from the Nord-Trondelag health study. Diabet Med. 2013; 30(1): 56-64.	High alcohol use
Rasouli B, Andersson T, Carlsson P-O, Grill V, Groop L, Martinell M, Storm P, Tuomi T, Carlsson S. Smoking and the Risk of LADA: Results From a Swedish Population-Based Case-Control Study. Diabetes Care. 2016; 39(5): 794–800.	Smoking
Rathmann W, Strassburger K, Heier M, Holle R, Thorand B, Giani G, Meisinger C. Incidence of Type 2 diabetes in the elderly German population and the effect of clinical and lifestyle risk factors: KORA S4/F4 cohort study. Diabet Med. 2009; 26(12): 1212-9.	High body-mass index, Low physical activity
Rayner J, D'Arcy E, Ross LJ, Hodge A, Schoenaker DAJM. Carbohydrate restriction in midlife is associated with higher risk of type 2 diabetes among Australian women: A cohort study. Nutr Metab Cardiovasc Dis. 2020; 30(3): 400-409.	Diet low in vegetables
Reis JP, Loria CM, Sorlie PD, Park Y, Hollenbeck A, Schatzkin A. Lifestyle factors and risk for new-onset diabetes: a population-based cohort study. Ann Intern Med. 2011; 155(5): 292-9.	Low physical activity
Renzi M, Cerza F, Gariazzo C, Agabiti N, Cascini S, Di Domenicantonio R, Davoli M, Forastiere F, Cesaroni G. Air pollution and occurrence of type 2 diabetes in a large cohort study. Environ Int. 2018; 112: 68-76.	Ambient particulate matter pollution and household air pollution
Rimm EB, Chan J, Stampfer MJ, Colditz GA, Willett WC. Prospective study of cigarette smoking, alcohol use, and the risk of diabetes in men. BMJ. 1995; 310(6979): 555-9.	Smoking
Roediger MA, Marucci MFN, Gobbo LA, Dourado DAQS, Santos JLF, Duarte YAO, Lebrão ML. Reported diabetes mellitus: incidence and determinants in cohort of community dwelling elderly people in São Paulo City, Brazil: SABE study, health, wellness and aging. Ciênc Saúde Colet. 2018; 23(11): 3913-22.	High body-mass index

Rolandsson O, Hägg E, Nilsson M, Hallmans G, Mincheva-Nilsson L, Lernmark A. Prediction of diabetes with body mass index, oral glucose tolerance test and islet cell autoantibodies in a regional population. J Intern Med. 2001; 249(4): 279-88.	High body-mass index
Sairenchi T, Iso H, Nishimura A, Hosoda T, Irie F, Saito Y, Murakami A, Fukutomi H. Cigarette smoking and risk of type 2 diabetes mellitus among middle-aged and elderly Japanese men and women. Am J Epidemiol. 2004; 160(2): 158-62.	Smoking
Sakurai M, Nakamura K, Miura K, Takamura T, Yoshita K, Morikawa Y, Ishizaki M, Kido T, Naruse Y, Suwazono Y, Kaneko S, Sasaki S, Nakagawa H. Dietary glycemic index and risk of type 2 diabetes mellitus in middle-aged Japanese men. Metab Clin Exp. 2012; 61(1): 47-55.	Diet low in fiber
Sakurai M, Nakamura K, Miura K, Takamura T, Yoshita K, Nagasawa SY, Morikawa Y, Ishizaki M, Kido T, Naruse Y, Suwazono Y, Sasaki S, Nakagawa H. Sugar-sweetened beverage and diet soda consumption and the 7-year risk for type 2 diabetes mellitus in middle-aged Japanese men. Eur J Nutr. 2014; 53(1): 251-8.	Diet high in sugar-sweetened beverages
Salmeron J, Ascherio A, Rimm EB, Colditz GA, Spiegelman D, Jenkins DJ, Stampfer MJ, Wing AL, Willett WC. Dietary fiber, glycemic load, and risk of NIDDM in men. Diabetes Care. 1997; 20(4): 545–50.	Diet low in fiber
Salmerón J, Manson JE, Stampfer MJ, Colditz GA, Wing AL, Willett WC. Dietary fiber, glycemic load, and risk of non-insulin-dependent diabetes mellitus in women. JAMA. 1997; 277(6): 472-7.	Diet low in fiber
Sanada H, Yokokawa H, Yoneda M, Yatabe J, Sasaki Yatabe M, Williams SM, Felder RA, Jose PA. High body mass index is an important risk factor for the development of type 2 diabetes. Intern Med. 2012; 51(14): 1821-6.	High body-mass index
Saremi A, Hanson RL, Tulloch-Reid M, Williams DE, Knowler WC. Alcohol consumption predicts hypertension but not diabetes. J Stud Alcohol. 2004; 65(2): 184-90.	High alcohol use
Sasai H, Sairenchi T, Iso H, Irie F, Otaka E, Tanaka K, Ota H, Muto T. Relationship between obesity and incident diabetes in middle-aged and older Japanese adults: the Ibaraki Prefectural Health Study. Mayo Clin Proc. 2010; 85(1): 36-40.	High body-mass index
Sato KK, Hayashi T, Harita N, Koh H, Maeda I, Endo G, Nakamura Y, Kambe H, Kiyotaki C. Relationship between drinking patterns and the risk of type 2 diabetes: the Kansai Healthcare Study. J Epidemiol Community Health. 2012; 66(6): 507-11.	High alcohol use
Sauvaget C, Ramadas K, Thomas G, Vinoda J, Thara S, Sankaranarayanan R. Body mass index, weight change and mortality risk in a prospective study in India. Int J Epidemiol. 2008; 37(5): 990-1004.	High body-mass index
Sawada SS, Lee IM, Muto T, Matuszaki K, Blair SN. Cardiorespiratory fitness and the incidence of type 2 diabetes: prospective study of Japanese men. Diabetes Care. 2003; 26(10): 2918-22.	Smoking
Sawada SS, Lee IM, Naito H, Noguchi J, Tsukamoto K, Muto T, Higaki Y, Tanaka H, Blair SN. Long-term trends in cardiorespiratory fitness and the incidence of type 2 diabetes. Diabetes Care. 2010; 33(6): 1353-7.	High alcohol use
Schmidt M, Johannesdottir SA, Lemeshow S, Lash TL, Ulrichsen SP, Bøtker HE, Sørensen HT. Obesity in young men, and individual and combined risks of type 2 diabetes, cardiovascular morbidity and death before 55 years of age: a Danish 33-year follow-up study. BMJ Open. 2013; 3(4).	High body-mass index
Schulze MB, Liu S, Rimm EB, Manson JE, Willett WC, Hu FB. Glycemic index, glycemic load, and dietary fiber intake and incidence of type 2 diabetes in younger and middle-aged women. Am J Clin Nutr. 2004; 80(2): 348-56.	Diet low in fiber
Schulze MB, Schulz M, Heidemann C, Schienkiewitz A, Hoffmann K, Boeing H. Fiber and magnesium intake and incidence of type 2 diabetes: a prospective study and meta-analysis. Arch Intern Med. 2007; 167(9): 956-65.	Diet low in fiber
Shang X, Li J, Tao Q, Li J, Li X, Zhang L, Liu X, Wang Q, Shi X, Zhao Y, Hu S, Jiang L, Yang Y. educational level, obesity and incidence of diabetes among Chinese adult men and women aged 18-59 years old: an 11-year follow-up study. PLoS One. 2013; 8(6): e66479.	High body-mass index

Shaper AG, Wannamethee SG, Walker M. Body weight: implications for the prevention of coronary heart disease, stroke, and diabetes mellitus in a cohort study of middle aged men. BMJ. 1997; 314(7090): 1311-7.	High body-mass index
Shi L, Shu XO, Li H, Cai H, Liu Q, Zheng W, Xiang YB, Villegas R. Physical activity, smoking, and alcohol consumption in association with incidence of type 2 diabetes among middle-aged and elderly Chinese men. PLoS One. 2013; 8(11): e77919.	High alcohol use, Low physical activity
Siegel LC, Sesso HD, Bowman TS, Lee IM, Manson JE, Gaziano JM. Physical activity, body mass index, and diabetes risk in men: a prospective study. Am J Med. 2009; 122(22): 1115-21.	Low physical activity
Sluijs I, van der Schouw YT, van der A DL, Spijkerman AM, Hu FB, Grobbee DE, Beulens JW. Carbohydrate quantity and quality and risk of type 2 diabetes in the European Prospective Investigation into Cancer and Nutrition-Netherlands (EPIC-NL) study. Am J Clin Nutr. 2010; 92(4): 905–11.	Diet low in fiber
Someya Y, Tamura Y, Kohmura Y, Aoki K, Kawai S, Daida H, Naito H. A body mass index over 22 kg/m2 at college age is a risk factor for future diabetes in Japanese men. PLoS One. 2019; 14(1): e0211067.	High body-mass index
Song Y, Manson JE, Buring JE, Liu S. A prospective study of red meat consumption and type 2 diabetes in middle-aged and elderly women: the women's health study. Diabetes Care. 2004; 27(9): 2108–15.	Diet high in processed meat
Soriguer F, Colomo N, Olveira G, Garcia-Fuentes E, Esteva I, Ruiz de Adana MS, Morcillo S, Porras N, Valdes S, Rojo-Martinez G. White rice consumption and risk of type 2 diabetes. Clin Nutr. 2013; 32(3): 481–4.	Diet low in whole grains
Steele CJ, Schöttker B, Marshall AH, Kouvonen A, O'Doherty MG, Mons U, Saum KU, Boffetta P, Trichopoulou A, Brenner H, Kee F. Education achievement and type 2 diabetes-what mediates the relationship in older adults? Data from the ESTHER study: a population-based cohort study. BMJ Open. 2017; 7(4): e013569.	High alcohol use
Steinbrecher A, Erber E, Grandinetti A, Kolonel LN, Maskarinec G. Meat consumption and risk of type 2 diabetes: the Multiethnic Cohort. Public Health Nutr. 2011; 14(4): 568–74.	Diet high in processed meat, Diet high in red meat
Steinbrecher A, Erber E, Grandinetti A, Nigg C, Kolonel LN, Maskarinec G. Physical activity and risk of type 2 diabetes among Native Hawaiians, Japanese Americans, and Caucasians: the Multiethnic Cohort. J Phys Act Health. 2012; 9(5): 634-41.	Low physical activity
Steinbrecher A, Morimoto Y, Heak S, Ollberding NJ, Geller KS, Grandinetti A, Kolonel LN, Maskarinec G. The preventable proportion of type 2 diabetes by ethnicity: the multiethnic cohort. Ann Epidemiol. 2011; 21(7): 526-35.	High alcohol use
Stringhini S, Tabak A, Akbaraly TN, Sabia S, Shipley MJ, Marmot MG, Brunner EJ, Batty GD, Bovet P, Kivimäki M. Contribution of modifiable risk factors to social inequalities in type 2 diabetes: prospective Whitehall II cohort study. BMJ. 2012; 345: e5452.	High alcohol use, Low physical activity
Strodl E, Kenardy J. Psychosocial and non-psychosocial risk factors for the new diagnosis of diabetes in elderly women. Diabetes Res Clin Pract. 2006; 74(1): 57-65.	High alcohol use
Sui X, Hooker SP, Lee IM, Church TS, Colabianchi N, Lee CD, Blair SN. A prospective study of cardiorespiratory fitness and risk of type 2 diabetes in women. Diabetes Care. 2008; 31(3): 550-5.	High body-mass index
Sun Q, Spiegelman D, van Dam RM, Holmes MD, Malik VS, Willett WC, Hu FB. White rice, brown rice, and risk of type 2 diabetes in US men and women. Arch Intern Med. 2010; 170(11): 961–9.	Diet low in whole grains
Sung EJ, Sunwoo S, Kim SW, Kim YS. Obesity as a risk factor for non-insulin-dependent diabetes mellitus in Korea. J Korean Med Sci. 2001; 16(4): 391-6.	High body-mass index
Talaei M, Wang YL, Yuan JM, Pan A, Koh WP. Meat, Dietary Heme Iron, and Risk of Type 2 Diabetes Mellitus: The Singapore Chinese Health Study. Am J Epidemiol. 2017; 186(7): 824-833.	Diet high in red meat
Tatsumi Y, Ohno Y, Morimoto A, Nishigaki Y, Mizuno S, Watanabe S. Age differences in the risk of diabetes incidence according to body mass index level in Japanese women. Obes Res Clin Pract. 2013; 7(6): e455-63.	High body-mass index

Teratani T, Morimoto H, Sakata K, Oishi M, Tanaka K, Nakada S, Nogawa K, Suwazono Y. Dose-response relationship between tobacco or alcohol consumption and the development of diabetes mellitus in Japanese male workers. Drug Alcohol Depend. 2012; 125(3): 276-82.	High alcohol use, Smoking
To T, Zhu J, Villeneuve PJ, Simatovic J, Feldman L, Gao C, Williams D, Chen H, Weichenthal S, Wall C, Miller AB. Chronic disease prevalence in women and air pollution--A 30-year longitudinal cohort study. Environ Int. 2015; 80: 26–32.	Ambient particulate matter pollution and household air pollution
Tsai AC, Lee SH. Determinants of new-onset diabetes in older adults–Results of a national cohort study. Clin Nutr. 2015; 34(5): 937-42.	High body-mass index, Low physical activity
Tsai SP, Donnelly RP, Wendt JK. Obesity and mortality in a prospective study of a middle-aged industrial population. J Occup Environ Med. 2006; 48(1): 22-7.	High body-mass index
Tsumura K, Hayashi T, Suematsu C, Endo G, Fujii S, Okada K. Daily alcohol consumption and the risk of type 2 diabetes in Japanese men: the Osaka Health Survey. Diabetes Care. 1999; 22(9): 1432-7.	High alcohol use
Turner MC,Jerrett M, Pope CA 3rd, Krewski D, Gapstur SM, Diver WR, Beckerman BS, Marshall JD, Su J, Crouse DL, Burnett RT. Long-term ozone exposure and mortality in a large prospective study . Am J Respir Crit Care Med. 2016; 193(10): 1134-42.	Ambient particulate matter pollution and household air pollution
Uchimoto S, Tsumura K, Hayashi T, Suematsu C, Endo G, Fujii S, Okada K. Impact of cigarette smoking on the incidence of Type 2 diabetes mellitus in middle-aged Japanese men: the Osaka Health Survey. Diabet Med. 1999; 16(11): 951-5.	Smoking
van Dam RM, Hu FB, Rosenberg L, Krishnan S, Palmer JR. Dietary calcium and magnesium, major food sources, and risk of type 2 diabetes in U.S. black women. Diabetes Care. 2006; 29(10): 2238-43.	Diet low in whole grains
van Woudenbergh GJ, Kuijsten A, Tigcheler B, Sijbrands EJ, van Rooij FJ, Hofman A, Witteman JC, Feskens EJ. Meat consumption and its association with C-reactive protein and incident type 2 diabetes: the Rotterdam Study. Diabetes Care. 2012; 35(7): 1499-505.	Diet high in processed meat, Diet high in red meat
Villegas R, Shu XO, Gao Y-T, Yang G, Cai H, Li H, Zheng W. The association of meat intake and the risk of type 2 diabetes may be modified by body weight. Int J Med Sci. 2006; 3(4): 152–9.	Diet high in processed meat, Diet high in red meat
Villegas R, Shu XO, Gao Y-T, Yang G, Elasy T, Li H, Zheng W. Vegetable but Not Fruit Consumption Reduces the Risk of Type 2 Diabetes in Chinese Women. J Nutr. 2008; 138(3): 574–80.	Diet low in fruits, Diet low in vegetables
Villegas R, Shu XO, Li H, Yang G, Matthews CE, Leitzmann M, Li Q, Cai H, Gao YT, Zheng W. Physical activity and the incidence of type 2 diabetes in the Shanghai women's health study. Int J Epidemiol. 2006; 35(6): 1553–62.	Low physical activity
Virtanen HEK, Koskinen TT, Voutilainen S, Mursu J, Tuomainen TP, Kokko P, Virtanen JK. Intake of different dietary proteins and risk of type 2 diabetes in men: the Kuopio Ischaemic Heart Disease Risk Factor Study. Br J Nutr. 2017; 117(6): 882-893.	Diet high in red meat
Waki K, Noda M, Sasaki S, Matsumura Y, Takahashi Y, Isogawa A, Ohashi Y, Kadowaki T, Tsugane S, JPHC Study Group. Alcohol consumption and other risk factors for self-reported diabetes among middle-aged Japanese: a population-based prospective study in the JPHC study cohort I. Diabet Med. 2005; 22(3): 323-31.	High alcohol use, Low physical activity, Smoking
Waller K, Kaprio J, Lehtovirta M, Silventoinen K, Koskenvuo M, Kujala UM. Leisure-time physical activity and type 2 diabetes during a 28 year follow-up in twins. Diabetologia. 2010; 53(12): 2531–7.	Low physical activity
Wang CS, Chang TT, Yao WJ, Wang ST, Chou P. The impact of smoking on incident type 2 diabetes in a cohort with hepatitis B but not hepatitis C infection. J Viral Hepat. 2017; 24(12): 1114-1120.	High alcohol use
Wannamethee SG, Camargo CA, Manson JE, Willett WC, Rimm EB. Alcohol drinking patterns and risk of type 2 diabetes mellitus among younger women. Arch Intern Med. 2003; 163(11): 1329-36.	High alcohol use
Wannamethee SG, Shaper AG, Perry IJ, Alberti KG. Alcohol consumption and the incidence of type II diabetes. J Epidemiol Community Health. 2002; 56(7): 542-8.	High alcohol use

Wannamethee SG, Shaper AG, Perry IJ, British Regional Heart Study. Smoking as a modifiable risk factor for type 2 diabetes in middle-aged men. Diabetes Care. 2001; 24(9): 1590-5.	Smoking
Wannamethee SG, Whincup PH, Thomas MC, Sattar N. Associations between dietary fiber and inflammation, hepatic function, and risk of type 2 diabetes in older men: potential mechanisms for the benefits of fiber on diabetes risk. Diabetes Care. 2009; 32(10): 1823-5.	Diet low in fiber
Waring ME, Eaton CB, Lasater TM, Lapane KL. Incident diabetes in relation to weight patterns during middle age. Am J Epidemiol. 2010; 171(5): 550-6.	High body-mass index
Wei M, Gibbons LW, Mitchell TL, Kampert JB, Blair SN. Alcohol intake and incidence of type 2 diabetes in men. Diabetes Care. 2000; 23(1): 18-22.	High alcohol use
Weinmayr G, Hennig F, Fuks K, Nonnemacher M, Jakobs H, Möhlenkamp S, Erbel R, Jöckel K-H, Hoffmann B, Moebus S, Heinz Nixdorf Recall Investigator Group. Long-term exposure to fine particulate matter and incidence of type 2 diabetes mellitus in a cohort study: effects of total and traffic-specific air pollution. Environ Health. 2015; 14: 53.	Ambient particulate matter pollution and household air pollution
Weinstein AR, Sesso HD, Lee IM, Cook NR, Manson JE, Buring JE, Gaziano JM. Relationship of physical activity vs body mass index with type 2 diabetes in women. JAMA. 2004; 292(10): 1188-94.	High body-mass index
Weng LC, Lee NJ, Yeh WT, Ho LT, Pan WH. Lower intake of magnesium and dietary fiber increases the incidence of type 2 diabetes in Taiwanese. J Formos Med Assoc. 2012; 111(11): 651-9.	Diet low in fiber
Will JC, Galuska DA, Ford ES, Mokdad A, Calle EE. Cigarette smoking and diabetes mellitus: evidence of a positive association from a large prospective cohort study. Int J Epidemiol. 2001; 30(3): 540–6.	Smoking
Wirström T, Hilding A, Gu HF, Östenson C-G, Björklund A. Consumption of whole grain reduces risk of deteriorating glucose tolerance, including progression to prediabetes. Am J Clin Nutr. 2013; 97(1): 179–87.	Diet low in whole grains
Xu F, Ware RS, Tse LA, Wang Y, Wang Z, Hong X, Chan EY, Dunstan DW, Owen N. Joint associations of physical activity and hypertension with the development of type 2 diabetes among urban men and women in Mainland China. PLoS One. 2014; 9(2): e88719.	Low physical activity
Yatsuya H, Li Y, Hirakawa Y, Ota A, Matsunaga M, Haregot HE, Chiang C, Zhang Y, Tamakoshi K, Toyoshima H, Aoyama A. A Point System for Predicting 10-Year Risk of Developing Type 2 Diabetes Mellitus in Japanese Men: Aichi Workers' Cohort Study. J Epidemiol. 2018; 28(8): 347-352.	High alcohol use
Yeh HC, Duncan BB, Schmidt MI, Wang NY, Brancati FL. Smoking, smoking cessation, and risk for type 2 diabetes mellitus: a cohort study. Ann Intern Med. 2010; 152(1): 10-7.	Smoking
Yin P, Brauer M, Cohen A, Burnett RT, Liu J, Liu Y, Liang R, Wang W, Qi J, Wang L, Zhou M. Long-term Fine Particulate Matter Exposure and Nonaccidental and Cause-specific Mortality in a Large National Cohort of Chinese Men [Unpublished]. Environ Health Perspect. 2017; 125(11): 117002.	Ambient particulate matter pollution and household air pollution
Zelle DM, Agarwal PK, Ramirez JL, van der Heide JJ, Corpeleijn E, Gans RO, Navis G, Bakker SJ. Alcohol consumption, new onset of diabetes after transplantation, and all-cause mortality in renal transplant recipients. Transplantation. 2011; 92(2): 203-9.	High alcohol use
Zhang L, Curhan GC, Hu FB, Rimm EB, Forman JP. Association between passive and active smoking and incident type 2 diabetes in women. Diabetes Care. 2011; 34(4): 892-7.	Second-hand smoke

Section 8. Contributions

Managing the overall research enterprise

Hailey Hagins, Simon I Hay, Paulina A Lindstedt, Kanyin Liane Ong, and Amanda E Smith.

Writing the first draft of the manuscript
Susan A McLaughlin, Kanyin Liane Ong, and Lauryn K Stafford.

Primary responsibility for applying analytical methods to produce estimates
Jessica A Cruz, Bronte E Dalton, Joe Duprey, Kanyin Liane Ong, and Lauryn K Stafford.

Primary responsibility for seeking, cataloguing, extracting, or cleaning data; designing or coding figures and tables
Kanyin Liane Ong and Lauryn K Stafford.

Providing data or critical feedback on data sources
Yohannes Habtegiorgis Abate, Mohammadreza Abbasian, Rami Abd-Rabu, Abu Yousuf Md Abdullah, Hassan Abidi, Richard Gyan Aboagye, Hassan Abolhassani, Denberu Eshetie Adane, Tigist Demssew Adane, Victor Adekanmbi, Qorinah Estiningtyas Sakilah Adnani, Marcela Agudelo-Botero, Bright Opoku Ahinkorah, Rizwan Ahmad, Sajjad Ahmad, Ali Ahmadi, Ali Ahmed, Ayman Ahmed, Hanadi Al Hamad, Tareq Mohammed Ali AL-Ahdal, Mohammad T AlBataineh, Jacqueline Elizabeth Alcalde-Rabanal, Hassam Ali, Syed Mohamed Aljunid, Sami Almustanyir, Nelson Alvis-Guzman, Ganiyu Adeniyi Amusa, Jalal Arabloo, Hidayat Arifin, Benedetta Armocida, Anton A Artamonov, Judie Arulappan, Mulu Tiruneh Asemu, Seyyed Shamsadin Athari, Ahmed Awaisu, Ashish D Badiye, Sara Bagherieh, Saeed Bahadory, Ruhai Bai, Mainak Bardhan, Till Winfried Bärnighausen, Amadou Barrow, Afisu Basiru, Sanjay Basu, Abdul-Monim Mohammad Batiha, Kavita Batra, Mulat Tirfie Bayih, Nebiyou Simegnew Bayileyegn, Alehegn Bekele Bekele, Melaku Ashagrie Belete, Sonu Bhaskar, Ajay Nagesh Bhat, Jasvinder Singh Bhatti, Boris Bikbov, Faiq Bilal, Bagas Suryo Bintoro, Archith Boloor, Michael Brauer, Luciana Aparecida Campos, Ismael R Campos-Nonato, Chao Cao, Carlos A Castañeda-Orjuela, Ferrán Catalá-López, Gashaw Sisay Chanie, Vijay Kumar Chattu, Endeshaw Chekol Abebe, William C S Cho, Rajiv Chowdhury, Dinh-Toi Chu, Alyssa Columbus, Ewerton Cousin, Michael H Criqui, Natália Cruz-Martins, Xiaochen Dai, Albertino Antonio Moura Damasceno, Lalit Dandona, Rakhi Dandona, Saswati Das, Fitsum Wolde Demisse, Hardik Dineshbhai Desai, Anteneh Mengist Dessie, Meghnath Dhimal, Linh Phuong Doan, Francisco Winter dos Santos Figueiredo, Bruce B Duncan, Michael Ekholuenetale, Temitope Cyrus Ekundayo, Islam Y Elgendy, Hawi Leul Esayas, Adeniyi Francis Fagbamigbe, Ayesha Fahim, Shahab Falahi, Hossein Farrokhpour, Farshad Farzadfar, Ali Fatehizadeh, David Flood, Roham Foroumadi, Masoumeh Foroutan Koudehi, Santosh Gaihre, Yaseen Galali, Balasankar Ganesan, Teferi Gebru Gebremeskel, Lemma Getacher, Fariba Ghassemi, Mahaveer Golechha, Pouya Goleij, Rajeev Gupta, Vivek Kumar Gupta, Teklehaimanot Gereziher Haile, Arvin Haj-Mirzaian, Rabih Halwani, Shafiul Haque, Netanja I Harlianto, Soheil Hassanipour, Johannes Haubold, Kamran Hessami, Hassan Hosseinzadeh, Mehdi Hosseinzadeh, Md Nazmul Huda, Salman Hussain, Hong-Han Huynh, Segun Emmanuel Ibitoye, Nayu Ikeda, Rakibul M Islam, Sheikh Mohammed Shariful Islam, Nahlah Elkudssiah Ismail, Gaetano Isola, Haitham Jahrami, Rajesh Jain, Balamurugan Janakiraman, Sathish Kumar Jayapal, Shubha Jayaram, Jost B Jonas, Abel Joseph, Charity Ehimwenma Joshua, Farahnaz Joukar, Jacek Jerzy Jozwiak, Billingsley Kaambwa, Vidya Kadashetti, Rohollah Kalhor, Himal Kandel, Samad Karkhah, Patrick DMC Katoto, Yousef Saleh Khader, Himanshu Khajuria, Maseer Khan, Moien AB Khan, Amir M Khater, Sorour Khateri, Jagdish Khubchandani, Adnan Kisa, Oleksii Korzh, Kewal Krishan, Yuvaraj Krishnamoorthy, Barthelemy Kuate Defo, Harish Kumar, Maria Dyah Kurniasari, Thao Thi Thu Le, Munjae Lee, Sang-woong Lee, Seung Won Lee, Shaun Wen Huey Lee, Yongze Li, Lee-Ling Lim, Stephen S Lim, Xuefeng Liu, Stefan Lorkowski, Rafael Lozano, Azzam A Maghazachi, Dianna J Magliano, Soleiman Mahjoub, Anh Tuan Mai, Azeem Majeed, Pantea Majma Sanaye, Kashish Malhotra, Deborah Carvalho Malta, Abdullah A Mamun, Borhan Mansouri, Roy Rillera Marzo, Reza Masoudi, Andrea Maugeri, Mahboobeh Meshkat, Irmina Maria Michalek, Le Huu Nhat Minh, Erkin M Mirrakhimov, Awoke Misganaw, Babak Moazen, Mohsen Mohammadi, Abdollah Mohammadian-Hafshejani, Alireza Mohseni, Ali H Mokdad, Sara Momtazmanesh, Lorenzo Monasta, Maryam Moradi, Yousef Moradi, Jakub Morze, Elias Mossialos, Ebrahim Mostafavi, Ulrich Otto Mueller, Admir Mulita, Francesk Mulita, Efrén Murillo-Zamora, Christopher J L Murray, Kamarul Imran Musa, Julius C Mwita, Shankar Prasad Nagaraju, Mohsen Naghavi, Firzan Nainu, Tapas Sadasivan Nair, Shumaila Nargus, Zuhair S Natto, Biswa Prakash Nayak, Ruxandra Irina Negoi, Dang H Nguyen, Hien Quang Nguyen, Phat Tuan Nguyen, Van Thanh Nguyen, Robina Khan Niazi, Muhammad A Nizam, Lawrence Achilles Nnyanzi, Jean Jacques Noubiap, Chimezie Igwegbe Nzoputam, Bogdan Oancea, Nkechi Martina Odogwu, Vivek Anand Ojha, Osaretin Christabel Okonji, Patrick Godwin Okwute, Isaac Iyinoluwa Olufadewa, Kanyin Liane Ong, Obinna E Onwujekwe, Alberto Ortiz, Uchechukwu Levi Osuagwu, Mayowa O Owolabi, Jagadish Rao Padubidri, Raffaele Palladino, Demosthenes Panagiotakos, Songhomitra Panda-Jonas, Anamika Pandey, Ashok Pandey, Shahina Pardhan, Maja Pasovic, Jay Patel, Jenil R Patel, Uttam Paudel, Simone Perna, Maarten J Postma, Naeimeh Pourtaheri, Elton Junio Sady Prates, Mirza Muhammad Fahd Qadir, Ibrar Rafique, Mehran Rahimi, Mahban Rahimifard, Vafa Rahimi-Movaghar, Shayan Rahmani, Bibek Rajbhandari, Pradhum Ram, Sheena Ramazanu, Juwel Rana, Nemanja Rancic, Chythra R Rao, Sina Rashedi, Vahid Rashedi, Ahmed Mustafa Rashid, Zubair Ahmed Ratan, Salman Rawaf, Lal Rawal, Elrashdy Moustafa Mohamed Redwan, Kannan RR Rengasamy, Andre M N Renzaho, Luis Felipe Reyes, Nima Rezaei, Hossein Rezazadeh, Seyed Mohammad Riahi, Jefferson Antonio Buendia Rodriguez, Leonardo Roever, Peter Rohloff, Abazar Roustazadeh, Godfrey M Rwegerera, Aly M A Saad, Siamak Sabour, Basema Saddik, Umar Saeed, KM Saif-Ur-Rahman, Mirza Rizwan Sajid, Mohamed A Saleh, Mohammad Amin

Salehi, Juan Sanabria, Milena M Santric-Milicevic, Saman Sargazi, Brijesh Sathian, Monika Sawhney, Maria Inês Schmidt, Abdul-Aziz Seidu, Yashendra Sethi, Tahereh Shafaghat, Samiah Shahid, Masood Ali Shaikh, Mohd Shanawaz, Mohammed Shannawaz, Aminu Shittu, K M Shivakumar, Parnian Shobeiri, Luís Manuel Lopes Rodrigues Silva, Jasvinder A Singh, Paramdeep Singh, Anna Aleksandrovna Skryabina, Lauryn K Stafford, Muhammad Suleman, Jing Sun, Johan Sundström, Rafael Tabarés-Seisdedos, Mohammad Tabish, Ker-Kan Tan, Worku Animaw Temesgen, Pugazhenthan Thangaraju, Nihal Thomas, Amir Tiyuri, Marcello Tonelli, Marcos Roberto Tovani-Palone, Domenico Trico, Indang Trihandini, Jaya Prasad Tripathy, Samuel Joseph Tromans, Guesh Mebrahtom Tsegay, Abdul Rohim Tualeka, Sana Ullah, Era Upadhyay, Shoban Babu Varthya, Tommi Juhani Vasankari, Georgios-Ioannis Verras, Danh Cao Vo, Theo Vos, Yasir Waheed, Melissa Y Wei Wei, Abrha Hailay Weldemariam, Ronny Westerman, Hong Xiao, Suowen Xu, Dereje Y Yada, Siyan Yi, Naohiro Yonemoto, Iman Zare, Mikhail Sergeevich Zastrozhin, Zhi-Jiang Zhang, Jingjing Zhou, Magdalena Zielińska, Yossef Teshome Zikarg, Mohammad Zoladl, Alimuddin Zumla, and Yves Miel H Zuniga.

Developing methods or computational machinery

Qorinah Estiningtyas Sakilah Adnani, Ali Ahmadi, Syed Anees Ahmed, Tareq Mohammed Ali AL-Ahdal, Aleksandr Y Aravkin, Zahra Aryan, Mulu Tiruneh Asemu, Kavita Batra, Ajay Nagesh Bhat, Faiq Bilal, Michael Brauer, Eeshwar K Chandrasekar, Kaleb Coberly, Jessica A Cruz, Xiaochen Dai, Hardik Dineshbhai Desai, Linh Phuong Doan, Ali Fatehizadeh, Rasool Haddadi, Mohammad Heidari, Hassan Hosseinzadeh, Mehdi Hosseinzadeh, Hong-Han Huynh, Gaetano Isola, Sathish Kumar Jayapal, Vidya Kadashetti, Farima Kahe, Samad Karkhah, Amir M Khater, Sorour Khateri, Adnan Kisa, Thao Thi Thu Le, Sang-woong Lee, Seung Won Lee, Soleiman Mahjoub, Razzagh Mahmoudi, Borhan Mansouri, Reza Masoudi, Mahboobeh Meshkat, Le Huu Nhat Minh, Mohsen Mohammadi, Ali H Mokdad, Yousef Moradi, Admir Mulita, Francesk Mulita, Christopher J L Murray, Mohsen Naghavi, Abdallah Y Naser, Dang H Nguyen, Hien Quang Nguyen, Phat Tuan Nguyen, Van Thanh Nguyen, Robina Khan Niazi, Kanyin Liane Ong, Michal Ordak, Mirza Muhammad Fahd Qadir, Bibek Rajbhandari, Hossein Rezazadeh, Seyed Mohammad Riahi, Mónica Rodrigues, Umar Saeed, Reed J D Sorensen, Lauryn K Stafford, Muhammad Suleman, Shoban Babu Varthya, Danh Cao Vo, Stein Emil Vollset, Theo Vos, Ronny Westerman, Naohiro Yonemoto, and Mikhail Sergeevich Zastrozhin.

Providing critical feedback on methods or results

Amirali Aali, Melsew Dagne Abate, Yohannes Habtegiorgis Abate, Mohammadreza Abbasian, Samar Abd ElHafeez, Deldar Morad Abdulah, Abu Yousuf Md Abdullah, Vida Abedi, Hassan Abidi, Richard Gyan Aboagye, Hassan Abolhassani, Eman Abu-Gharbieh, Ahmed Abu-Zaid, Denberu Eshetie Adane, Tigist Demssew Adane, Isaac Yeboah Addo, Oyelola A Adegboye, Victor Adekanmbi, Abiola Victor Adepoju, Qorinah Estiningtyas Sakilah Adnani, Rotimi Felix Afolabi, Gina Agarwal, Marcela Agudelo-Botero, Constanza Elizabeth Aguilera Arriagada, Williams Agyemang-Duah, Bright Opoku Ahinkorah, Aqeel Ahmad, Danish Ahmad, Rizwan Ahmad, Sajjad Ahmad, Ali Ahmadi, Keivan Ahmadi, Ali Ahmed, Ayman Ahmed, Luai A Ahmed, Syed Anees Ahmed, Rufus Olusola Akinyemi, Hanadi Al Hamad, Syed Mahfuz Al Hasan, Tareq Mohammed Ali AL-Ahdal, Tariq A Alalwan, Ziyad Al-Aly, Mohammad T AlBataineh, Jacqueline Elizabeth Alcalde-Rabanal, Sharifullah Alemi, Hassam Ali, Tahereh Alinia, Syed Mohamed Aljunid, Sami Almustanyir, Rajaa M Al-Raddadi, Nelson Alvis-Guzman, Edward Kwabena Ameyaw, Sohrab Amiri, Ganiyu Adeniyi Amusa, Catalina Liliana Andrei, Adnan Ansar, Golnoosh Ansari, Alireza Ansari-Moghaddam, Anayochukwu Edward Anyasodor, Jalal Arabloo, Demelash Areda, Hidayat Arifin, Mesay Arkew, Johan Ärnlöv, Anton A Artamonov, Judie Arulappan, Raphael Taiwo Aruleba, Ashokan Arumugam, Zahra Aryan, Mulu Tiruneh Asemu, Mohammad Asghari-Jafarabadi, Daniel Asmelash, Thomas Astell-Burt, Mohammad Athar, Seyyed Shamsadin Athari, Maha Moh'd Wahbi Atout, Leticia Avila-Burgos, Ahmed Awaisu, Sina Azadnajafabad, Darshan B B, Hassan Babamohamadi, Muhammad Badar, Alaa Badawi, Ashish D Badiye, Nayereh Baghcheghi, Nasser Bagheri, Sara Bagherieh, Sulaiman Bah, Ruhai Bai, Atif Amin Baig, Ovidiu Constantin Baltatu, Hamid Reza Baradaran, Martina Barchitta, Mainak Bardhan, Noel C Barengo, Till Winfried Bärnighausen, Mark Thomaz Ugliara Barone, Amadou Barrow, Afisu Basiru, Sanjay Basu, Saurav Basu, Abdul-Monim Mohammad Batiha, Kavita Batra, Mulat Tirfie Bayih, Nebiyou Simegnew Bayileyegn, Amir Hossein Behnoush, Alehegn Bekele Bekele, Melaku Ashagrie Belete, Uzma Iqbal Belgaumi, Derrick A Bennett, Isabela M Bensenor, Alemshet Yirga Berhie, Sonu Bhaskar, Ajay Nagesh Bhat, Jasvinder Singh Bhatti, Boris Bikbov, Faiq Bilal, Bagas Suryo Bintoro, Veera R Bitra, Vesna Bjegovic-Mikanovic, Virginia Bodolica, Archith Boloor, Edward J Boyko, Javier Brazo-Sayavera, Hermann Brenner, Zahid A Butt, Luciana Aparecida Campos, Ismael R Campos-Nonato, Chao Cao, Yin Cao, Josip Car, Márcia Carvalho, Carlos A Castañeda-Orjuela, Ferrán Catalá-López, Ester Cerin, Joshua Chadwick, Eeshwar K Chandrasekar, Gashaw Sisay Chanie, Jaykaran Charan, Vijay Kumar Chattu, Kirti Chauhan, Huzaifa Ahmad Cheema, Endeshaw Chekol Abebe, Nicolas Cherbuin, Fatemeh Chichagi, Saravana Babu Chidambaram, William C S Cho, Sonali Gajanan Choudhari, Enayet Karim Chowdhury, Rajiv Chowdhury, Dinh-Toi Chu, Isaac Sunday Chukwu, Sheng-Chia Chung, Alyssa Columbus, Daniela Contreras, Ewerton Cousin, Michael H Criqui, Natália Cruz-Martins, Sarah Cuschieri, Bashir Dabo, Omid Dadras, Xiaochen Dai, Lalit Dandona, Rakhi Dandona, Ana Maria Dascalu, Nihar Ranjan Dash, Mohsen Dashti, Gebiso Roba Debele, Kourosh Delpasand, Fitsum Wolde Demisse, Getu Debalkie Demissie, Xinlei Deng, Salil V Deo, Emina Dervišević, Hardik Dineshbhai Desai, Aragaw Tesfaw Desale, Anteneh Mengist Dessie, Fikreab Desta, Sourav Dey, Kuldeep Dhama, Meghnath Dhimal, Nancy Diao, Daniel Diaz, Monica Dinu, Mengistie Diress, Shirin Djalalinia, Deepa Dongarwar, Siddhartha Dutta, Arkadiusz Marian Dziedzic, Hisham Atan Edinur, Michael Ekholuenetale, Temitope Cyrus Ekundayo, Muhammed Elhadi, Waseem El-Huneidi, Omar Abdelsadek Abdou Elmeligy, Mohamed A Elmonem, Destaw Endeshaw, Hawi Leul Esayas, Habitu Birhan Eshetu, Farshid Etaee, Ibtihal Fadhil, Adeniyi Francis Fagbamigbe,

Ayesha Fahim, Shahab Falahi, MoezAlIslam Ezzat Mahmoud Faris, Hossein Farrokhpour, Farshad Farzadfar, Ali Fatehizadeh, Ghazal Fazli, Xiaoqi Feng, Florian Fischer, David Flood, Ali Forouhari, Roham Foroumadi, Masoumeh Foroutan Koudehi, Abhay Motiramji Gaidhane, Santosh Gaihre, Abduzhappar Gaipov, Yaseen Galali, Balasankar Ganesan, Rupesh K Gautam, Mesfin Gebrehiwot, Kahsu Gebrekirstos Gebrekidan, Teferi Gebru Gebremeskel, Lemma Getacher, Mohammad Ghasemi Nour, Mahaveer Golechha, Davide Golinelli, Sameer Vali Gopalani, Habtamu Alganeh Guadie, Shi-Yang Guan, Temesgen Worku Gudayu, Rafael Alves Guimarães, Rashid Abdi Guled, Kartik Gupta, Rajeev Gupta, Vivek Kumar Gupta, Bishal Gyawali, Rasool Haddadi, Najah R Hadi, Teklehaimanot Gereziher Haile, Ramtin Hajibeygi, Rabih Halwani, Samer Hamidi, Md Abdul Hannan, Shafiul Haque, Hamid Harandi, Netanja I Harlianto, S M Mahmudul Hasan, Syed Shahzad Hasan, Hamidreza Hasani, Soheil Hassanipour, Mohammed Bheser Hassen, Johannes Haubold, Simon I Hay, Khezar Hayat, Golnaz Heidari, Mohammad Heidari, Kamran Hessami, Yuta Hiraike, Ramesh Holla, Md Shakhaoat Hossain, Sahadat Hossain, Mohammad-Salar Hosseini, Hassan Hosseinzadeh, Mehdi Hosseinzadeh, Md Nazmul Huda, Salman Hussain, Hong-Han Huynh, Bing-Fang Hwang, Segun Emmanuel Ibitoye, Nayu Ikeda, Irena M Ilic, Milena D Ilic, Leeberk Raja Inbaraj, Afrin Iqbal, Rakibul M Islam, Sheikh Mohammed Shariful Islam, Nahlah Elkudssiah Ismail, Gaetano Isola, Ramaiah Itumalla, Masao Iwagami, Chidozie C D Iwu, Ihoghosa Osamuyi Iyamu, Louis Jacob, Abdollah Jafarzadeh, Haitham Jahrami, Chinwe Jaja, Zahra Jamalpoor, Elham Jamshidi, Balamurugan Janakiraman, Krishnamurthy Jayanna, Sathish Kumar Jayapal, Shubha Jayaram, Ranil Jayawardena, Rime Jebai, Wonjeong Jeong, Yinzi Jin, Mohammad Jokar, Jost B Jonas, Abel Joseph, Nitin Joseph, Charity Ehimwenma Joshua, Farahnaz Joukar, Jacek Jerzy Jozwiak, Billingsley Kaambwa, Ali Kabir, Robel Hussen Kabthymer, Vidya Kadashetti, Farima Kahe, Rohollah Kalhor, Himal Kandel, Ibraheem M Karaye, Samad Karkhah, Patrick DMC Katoto, Navjot Kaur, Sina Kazemian, Sewnet Adem Kebede, Yousef Saleh Khader, Himanshu Khajuria, Amirmohammad Khalaji, Ajmal Khan, Maseer Khan, Moien AB Khan, Saval Khanal, Moawiah Mohammad Khatatbeh, Amir M Khater, Sorour Khateri, Fatemeh khorashadizadeh, Biruk Getahun Kibret, Min Seo Kim, Ruth W Kimokoti, Adnan Kisa, Mika Kivimäki, Ali-Asghar Kolahi, Somayeh Komaki, Farzad Kompani, Hamid Reza Koohestani, Oleksii Korzh, Karel Kostev, Nikhil Kothari, Ai Koyanagi, Kewal Krishan, Yuvaraj Krishnamoorthy, Barthelemy Kuate Defo, Md Abdul Kuddus, Mohammed Kuddus, Satyajit Kundu, Maria Dyah Kurniasari, Ambily Kuttikkattu, Carlo La Vecchia, Tea Lallukka, Anders O Larsson, Kamaluddin Latief, Basira Kankia Lawal, Thao Thi Thu Le, Trang Thi Bich Le, Munjae Lee, Sang-woong Lee, Seung Won Lee, Shaun Wen Huey Lee, Wei-Chen Lee, Samson Mideksa Legesse, Jacopo Lenzi, Ming-Chieh Li, Yongze Li, Lee-Ling Lim, Stephen S Lim, Chaojie Liu, Xuefeng Liu, Chun-Han Lo, Graciliana Lopes, Stefan Lorkowski, Rafael Lozano, Giancarlo Lucchetti, Azzam A Maghazachi, Dianna J Magliano, Phetole Walter Mahasha, Soleiman Mahjoub, Mansour Adam Mahmoud, Razzagh Mahmoudi, Marzieh Mahmoudimanesh, Anh Tuan Mai, Azeem Majeed, Konstantinos Christos Makris, Kashish Malhotra, Ahmad Azam Malik, Iram Malik, Tauqeer Hussain Mallhi, Deborah Carvalho Malta, Abdullah A Mamun, Borhan Mansouri, Hamid Reza Marateb, Parham Mardi, Santi Martini, Miquel Martorell, Roy Rillera Marzo, Reza Masoudi, Sahar Masoudi, Elezebeth Mathews, Andrea Maugeri, Giampiero Mazzaglia, Teferi Mekonnen, Mahboobeh Meshkat, Tomislav Mestrovic, Junmei Miao Jonasson, Tomasz Miazgowski, Irmina Maria Michalek, Le Huu Nhat Minh, GK Mini, Reza Mirfakhraie, Erkin M Mirrakhimov, Mohammad Mirza-Aghazadeh-Attari, Awoke Misganaw, Kebede Haile Misgina, Manish Mishra, Babak Moazen, Nouh Saad Mohamed, Esmaeil Mohammadi, Mohsen Mohammadi, Abdollah Mohammadian-Hafshejani, Marita Mohammadshahi, Alireza Mohseni, Ali H Mokdad, Sara Momtazmanesh, Md Moniruzzaman, Ute Mons, Fateme Montazeri, AmirAli Moodi Ghalibaf, Maryam Moradi, Yousef Moradi, Mostafa Moradi Sarabi, Negar Morovatdar, Shane Douglas Morrison, Jakub Morze, Elias Mossialos, Ebrahim Mostafavi, Ulrich Otto Mueller, Admir Mulita, Francesk Mulita, Efrén Murillo-Zamora, Christopher J L Murray, Kamarul Imran Musa, Julius C Mwita, Shankar Prasad Nagaraju, Mohsen Naghavi, Firzan Nainu, Tapas Sadasivan Nair, Hastyar Hama Rashid Najmuldeen, Vinay Nangia, Shumaila Nargus, Abdallah Y Naser, Hasan Nassereldine, Zuhair S Natto, Javaid Nauman, Biswa Prakash Nayak, Hadush Negash, Ruxandra Irina Negoi, Dang H Nguyen, Hau Thi Hien Nguyen, Hien Quang Nguyen, Phat Tuan Nguyen, Van Thanh Nguyen, Robina Khan Niazi, Yeshambel T Nigatu, Dina Nur Anggraini Ningrum, Muhammad A Nizam, Lawrence Achilles Nnyanzi, Jean Jacques Noubiap, Chimezie Igwegbe Nzoputam, Ogochukwu Janet Nzoputam, Bogdan Oancea, Nkechi Martina Odogwu, Oluwakemi Ololade Odukoya, Vivek Anand Ojha, Hassan Okati-Aliabad, Akinkunmi Paul Okekunle, Osaretin Christabel Okonji, Patrick Godwin Okwute, Isaac Iyinoluwa Olufadewa, Kanyin Liane Ong, Obinna E Onwujekwe, Michal Ordak, Alberto Ortiz, Uchechukwu Levi Osuagwu, Abderrahim Oulhaj, Mayowa O Owolabi, Alicia Padron-Monedero, Jagadish Rao Padubidri, Raffaele Palladino, Demosthenes Panagiotakos, Songhomitra Panda-Jonas, Anamika Pandey, Ashok Pandey, Seithikurippu R Pandi-Perumal, Shahina Pardhan, Tarang Parekh, Utsav Parekh, Maja Pasovic, Jay Patel, Jenil R Patel, Uttam Paudel, Veincent Christian Filipino Pepito, Marcos Pereira, Ionela-Roxana Petcu, Fanny Emily Petermann-Rocha, Vivek Podder, Maarten J Postma, Ghazaleh Pourali, Naeimeh Pourtaheri, Elton Junio Sady Prates, Mirza Muhammad Fahd Qadir, Ibrahim Qattea, Pourya Raee, Ibrar Rafique, Mehran Rahimi, Mahban Rahimifard, Vafa Rahimi-Movaghar, Md Mosfequr Rahman, Md Obaidur Rahman, Mosiur Rahman, Muhammad Aziz Rahman, Mohamed Rahmani, Shayan Rahmani, Vahid Rahmanian, Setyaningrum Rahmawaty, Niloufar Rahnavard, Bibek Rajbhandari, Pradhum Ram, Sheena Ramazanu, Juwel Rana, Nemanja Rancic, Muhammad Modassar Ali Nawaz Ranjha, Chythra R Rao, Deepthi Rapaka, Drona Prakash Rasali, Sina Rashedi, Vahid Rashedi, Ahmed Mustafa Rashid, Mohammad-Mahdi Rashidi, Zubair Ahmed Ratan, Salman Rawaf, Lal Rawal, Elrashdy Moustafa Mohamed Redwan, Kannan RR Rengasamy, Andre M N Renzaho, Luis Felipe Reyes, Nazila Rezaei, Nima Rezaei, Mohsen Rezaeian, Hossein Rezazadeh, Seyed Mohammad Riahi, Yohanes Andy Rias, Daniela Ribeiro, Mónica Rodrigues, Jefferson Antonio Buendia Rodriguez, Leonardo Roever, Peter Rohloff, Gholamreza Roshandel, Abazar Roustazadeh, Godfrey M Rwegerera, Aly M A Saad, Maha Mohamed Saber-Ayad, Siamak Sabour, Leila Sabzmakan, Basema Saddik, Erfan Sadeghi, Umar Saeed, Sahar Saeedi Moghaddam, Sare Safi, Sher Zaman Safi, Amene Saghazadeh, Fatemeh Saheb Sharif-Askari, Narjes Saheb Sharif-Askari, Harihar Sahoo, Soumya Swaroop Sahoo, KM Saif-Ur-Rahman, Mirza Rizwan Sajid, Mohamed A Saleh, Mohammad Amin Salehi, Joshua A Salomon, Juan Sanabria, Rama Krishna Sanjeev, Francesco Sanmarchi, Milena M Santric-Milicevic, Made Ary Sarasmita, Saman

Sargazi, Brijesh Sathian, Thirunavukkarasu Sathish, Monika Sawhney, Markus P Schlaich, Art Schuermans, Abdul-Aziz Seidu, Nachimuthu Senthil Kumar, Sadaf G Sepanlou, Yashendra Sethi, Tahereh Shafaghat, Melika Shafeghat, Mahan Shafie, Nilay S Shah, Samiah Shahid, Masood Ali Shaikh, Mohd Shanawaz, Mohammed Shannawaz, Sadaf Sharfaei, Bereket Beyene Shashamo, Rahman Shiri, Aminu Shittu, K M Shivakumar, Siddharudha Shivalli, Parnian Shobeiri, Fereshteh Shokri, Kerem Shuval, Migbar Mekonnen Sibhat, Luís Manuel Lopes Rodrigues Silva, Colin R Simpson, Jasvinder A Singh, Paramdeep Singh, Surjit Singh, Md Shahjahan Siraj, Anna Aleksandrovna Skryabina, Amanda E Smith, Abdullah Al Mamun Sohag, Solikhah Solikhah, Mohammad Sadegh Soltani-Zangbar, Ranjani Somayaji, Reed J D Sorensen, Lauryn K Stafford, Antonina V Starodubova, Sujata Sujata, Muhammad Suleman, Jing Sun, Rafael Tabarés-Seisdedos, Seyyed Mohammad Tabatabaei, Seyed-Amir Tabatabaeizadeh, Mohammad Tabish, Ensiyeh Taheri, Majid Taheri, Elahe Taki, Ker-Kan Tan, Birhan Tsegaw Taye, Worku Animaw Temesgen, Mohamad-Hani Temsah, Pugazhenthan Thangaraju, Rajshree Thapa, Samar Tharwat, Nihal Thomas, Jansje Henny Vera Ticoalu, Amir Tiyuri, Marcello Tonelli, Marcos Roberto Tovani-Palone, Domenico Trico, Indang Trihandini, Jaya Prasad Tripathy, Samuel Joseph Tromans, Guesh Mebrahtom Tsegay, Derara Girma Tufa, Stefanos Tyrovolas, Sana Ullah, Era Upadhyay, Seyed Mohammad Vahabi, Asokan Govindaraj Vaithinathan, Rohollah Valizadeh, Kim Robin van Daalen, Priya Vart, Shoban Babu Varthya, Siavash Vaziri, Madhur verma Verma, Georgios-Ioannis Verras, Danh Cao Vo, Stein Emil Vollset, Theo Vos, Birhanu Wagaye, Yasir Waheed, Cong Wang, Yanqing Wang, Ziyue Wang, Gizachew Tadesse Wassie, Melissa Y Wei Wei, Abrha Hailay Weldemariam, Ronny Westerman, Nuwan Darshana Wickramasinghe, YiFan Wu, Ratna DWI Wulandari, Juan Xia, Hong Xiao, Xiaoyue Xu, Lin Yang, Hiroshi Yatsuya, Metin Yesiltepe, Siyan Yi, Hunachew Kibret Yohannis, Yuyi You, Sojib Bin Zaman, Nelson Zamora, Kourosh Zarea, Armin Zarrintan, Mikhail Sergeevich Zastrozhin, Naod Gebrekrstos Zeru, Zhi-Jiang Zhang, Chenwen Zhong, Jingjing Zhou, Magdalena Zielińska, Yossef Teshome Zikarg, Sanjay Zodpey, Mohammad Zoladl, Zhiyong Zou, and Yves Miel H Zuniga.

Drafting the work or revising it critically for important intellectual content

Amirali Aali, Melsew Dagne Abate, Yohannes Habtegiorgis Abate, Mohammadreza Abbasian, Mohsen Abbasi-Kangevari, Zeinab Abbasi-Kangevari, Rami Abd-Rabu, Abu Yousuf Md Abdullah, Hassan Abidi, Hassan Abolhassani, Eman Abu-Gharbieh, Ahmed Abu-Zaid, Denberu Eshetie Adane, Isaac Yeboah Addo, Oyelola A Adegboye, Victor Adekanmbi, Abiola Victor Adepoju, Qorinah Estiningtyas Sakilah Adnani, Gina Agarwal, Zahra Babaei Aghdam, Constanza Elizabeth Aguilera Arriagada, Bright Opoku Ahinkorah, Danish Ahmad, Ali Ahmadi, Ali Ahmed, Ayman Ahmed, Luai A Ahmed, Marjan Ajami, Rufus Olusola Akinyemi, Syed Mahfuz Al Hasan, Tariq A Alalwan, Mohammad T AlBataineh, Jacqueline Elizabeth Alcalde-Rabanal, Tahereh Alinia, Sami Almustanyir, Nelson Alvis-Guzman, Firehiwot Amare, Sohrab Amiri, Ganiyu Adeniyi Amusa, Catalina Liliana Andrei, Golnoosh Ansari, Anayochukwu Edward Anyasodor, Jalal Arabloo, Mesay Arkew, Benedetta Armocida, Johan Ärnlöv, Judie Arulappan, Raphael Taiwo Aruleba, Ashokan Arumugam, Zahra Aryan, Mulu Tiruneh Asemu, Elaheh Askari, Mohammad Athar, Seyyed Shamsadin Athari, Maha Moh'd Wahbi Atout, Ahmed Awaisu, Sina Azadnajafabad, Darshan B B, Muhammad Badar, Alaa Badawi, Ashish D Badiye, Sara Bagherieh, Ruhai Bai, Atif Amin Baig, Ovidiu Constantin Baltatu, Hamid Reza Baradaran, Martina Barchitta, Mainak Bardhan, Noel C Barengo, Till Winfried Bärnighausen, Mark Thomaz Ugliara Barone, Francesco Barone-Adesi, Amadou Barrow, Hamideh Bashiri, Afisu Basiru, Sanjay Basu, Saurav Basu, Abdul-Monim Mohammad Batiha, Kavita Batra, Amir Hossein Behnoush, Alehegn Bekele Bekele, Melaku Ashagrie Belete, Uzma Iqbal Belgaumi, Luis Belo, Derrick A Bennett, Isabela M Bensenor, Kidanemaryam Berhe, Sonu Bhaskar, Ajay Nagesh Bhat, Jasvinder Singh Bhatti, Boris Bikbov, Saeid Bitaraf, Veera R Bitra, Vesna Bjegovic-Mikanovic, Virginia Bodolica, Edward J Boyko, Javier Brazo-Sayavera, Hermann Brenner, Daniela Calina, Chao Cao, Yin Cao, Josip Car, Márcia Carvalho, Carlos A Castañeda-Orjuela, Ferrán Catalá-López, Ester Cerin, Joshua Chadwick, Eeshwar K Chandrasekar, Vijay Kumar Chattu, Huzaifa Ahmad Cheema, Simiao Chen, Nicolas Cherbuin, Fatemeh Chichagi, William C S Cho, Rajiv Chowdhury, Dinh-Toi Chu, Alyssa Columbus, Daniela Contreras, Ewerton Cousin, Michael H Criqui, Natália Cruz-Martins, Sarah Cuschieri, Bashir Dabo, Albertino Antonio Moura Damasceno, Ana Maria Dascalu, Nihar Ranjan Dash, Claudio Alberto Dávila-Cervantes, Vanessa De la Cruz-Góngora, Gebiso Roba Debele, Getu Debalkie Demissie, Edgar Denova-Gutiérrez, Emina Dervišević, Hardik Dineshbhai Desai, Anteneh Mengist Dessie, Fikreab Desta, Syed Masudur Rahman Dewan, Sourav Dey, Meghnath Dhimal, Nancy Diao, Daniel Diaz, Monica Dinu, Linh Phuong Doan, Deepa Dongarwar, Francisco Winter dos Santos Figueiredo, Bruce B Duncan, Siddhartha Dutta, Arkadiusz Marian Dziedzic, Islam Y Elgendy, Muhammed Elhadi, Omar Abdelsadek Abdou Elmeligy, Mohamed A Elmonem, Destaw Endeshaw, Habitu Birhan Eshetu, Farshid Etaee, Adeniyi Francis Fagbamigbe, Ayesha Fahim, MoezAlIslam Ezzat Mahmoud Faris, Ali Fatehizadeh, Tomas Y Ferede, Florian Fischer, Ali Forouhari, Masoumeh Foroutan Koudehi, Santosh Gaihre, Yaseen Galali, Balasankar Ganesan, MA Garcia-Gordillo, Rupesh K Gautam, Mesfin Gebrehiwot, Teferi Gebru Gebremeskel, Lemma Getacher, Fataneh Ghadirian, Seyyed-Hadi Ghamari, Mohammad Ghasemi Nour, Fariba Ghassemi, Davide Golinelli, Sameer Vali Gopalani, Habtamu Alganeh Guadie, Shi-Yang Guan, Temesgen Worku Gudayu, Rafael Alves Guimarães, Kartik Gupta, Rajeev Gupta, Veer Bala Gupta, Vivek Kumar Gupta, Bishal Gyawali, Rasool Haddadi, Najah R Hadi, Teklehaimanot Gereziher Haile, Ramtin Hajibeygi, Arvin Haj-Mirzaian, Rabih Halwani, Graeme J Hankey, Md Abdul Hannan, Shafiul Haque, Hamid Harandi, S M Mahmudul Hasan, Syed Shahzad Hasan, Hamidreza Hasani, Johannes Haubold, Simon I Hay, Golnaz Heidari, Kamran Hessami, Yuta Hiraike, Ramesh Holla, Md Shakhaoat Hossain, Mohammad-Salar Hosseini, Hassan Hosseinzadeh, Junjie Huang, Md Nazmul Huda, Salman Hussain, Hong-Han Huynh, Segun Emmanuel Ibitoye, Nayu Ikeda, Irena M Ilic, Milena D Ilic, Rakibul M Islam, Sheikh Mohammed Shariful Islam, Nahlah Elkudssiah Ismail, Hiroyasu Iso, Gaetano Isola, Ramaiah Itumalla, Chidozie C D Iwu, Ihoghosa Osamuyi Iyamu, Assefa N Iyasu, Louis Jacob, Haitham Jahrami, Rajesh Jain, Chinwe Jaja, Balamurugan Janakiraman, Sathish Kumar Jayapal, Shubha Jayaram, Rime Jebai, Wonjeong Jeong, Jost B Jonas, Abel Joseph,

Nitin Joseph, Jacek Jerzy Jozwiak, Ali Kabir, Robel Hussen Kabthymer, Vidya Kadashetti, Farima Kahe, Himal Kandel, Shama D Karanth, Samad Karkhah, Patrick DMC Katoto, Navjot Kaur, Yousef Saleh Khader, Himanshu Khajuria, Amirmohammad Khalaji, Ajmal Khan, Maseer Khan, Moien AB Khan, Saval Khanal, Moawiah Mohammad Khatatbeh, Amir M Khater, Fatemeh khorashadizadeh, Jagdish Khubchandani, Biruk Getahun Kibret, Min Seo Kim, Adnan Kisa, Mika Kivimäki, Farzad Kompani, Oleksii Korzh, Nikhil Kothari, Ai Koyanagi, Kewal Krishan, Barthelemy Kuate Defo, Md Abdul Kuddus, Mohammed Kuddus, Rakesh Kumar, Satyajit Kundu, Maria Dyah Kurniasari, Carlo La Vecchia, Tea Lallukka, Bagher Larijani, Anders O Larsson, Kamaluddin Latief, Basira Kankia Lawal, Thao Thi Thu Le, Trang Thi Bich Le, Munjae Lee, Paul H Lee, Seung Won Lee, Samson Mideksa Legesse, Jacopo Lenzi, Lee-Ling Lim, Chaojie Liu, Chun-Han Lo, Stefan Lorkowski, Giancarlo Lucchetti, Dianna J Magliano, Soleiman Mahjoub, Mansour Adam Mahmoud, Razzagh Mahmoudi, Anh Tuan Mai, Azeem Majeed, Konstantinos Christos Makris, Ahmad Azam Malik, Iram Malik, Tauqeer Hussain Mallhi, Deborah Carvalho Malta, Abdullah A Mamun, Borhan Mansouri, Hamid Reza Marateb, Parham Mardi, Santi Martini, Miquel Martorell, Roy Rillera Marzo, Reza Masoudi, Elezebeth Mathews, Andrea Maugeri, Giampiero Mazzaglia, Susan A McLaughlin, Mahboobeh Meshkat, Tomislav Mestrovic, Tomasz Miazgowski, Irmina Maria Michalek, Le Huu Nhat Minh, J Jaime Miranda, Reza Mirfakhraie, Mohammad Mirza-Aghazadeh-Attari, Awoke Misganaw, Kebede Haile Misgina, Manish Mishra, Babak Moazen, Esmaeil Mohammadi, Mohsen Mohammadi, Abdollah Mohammadian-Hafshejani, Alireza Mohseni, Hoda Mojiri-forushani, Ali H Mokdad, Sara Momtazmanesh, Lorenzo Monasta, Ute Mons, Fateme Montazeri, AmirAli Moodi Ghalibaf, Maryam Moradi, Mostafa Moradi Sarabi, Negar Morovatdar, Shane Douglas Morrison, Jakub Morze, Ebrahim Mostafavi, Ulrich Otto Mueller, Christopher J L Murray, Kamarul Imran Musa, Julius C Mwita, Shankar Prasad Nagaraju, Mohsen Naghavi, Firzan Nainu, Hastyar Hama Rashid Najmuldeen, Shumaila Nargus, Abdallah Y Naser, Hasan Nassereldine, Zuhair S Natto, Javaid Nauman, Biswa Prakash Nayak, Hadush Negash, Ruxandra Irina Negoi, Dang H Nguyen, Hau Thi Hien Nguyen, Hien Quang Nguyen, Phat Tuan Nguyen, Van Thanh Nguyen, Robina Khan Niazi, Muhammad A Nizam, Lawrence Achilles Nnyanzi, Mamoona Noreen, Jean Jacques Noubiap, Chimezie Igwegbe Nzoputam, Ogochukwu Janet Nzoputam, Bogdan Oancea, Nkechi Martina Odogwu, Oluwakemi Ololade Odukoya, Akinkunmi Paul Okekunle, Osaretin Christabel Okonji, Patrick Godwin Okwute, Kanyin Liane Ong, Obinna E Onwujekwe, Michal Ordak, Alberto Ortiz, Uchechukwu Levi Osuagwu, Mayowa O Owolabi, Alicia Padron-Monedero, Jagadish Rao Padubidri, Raffaele Palladino, Songhomitra Panda-Jonas, Seithikurippu R Pandi-Perumal, Anca Mihaela Pantea Stoian, Shahina Pardhan, Jay Patel, Jenil R Patel, Uttam Paudel, Veincent Christian Filipino Pepito, Marcos Pereira, Norberto Perico, Ionela-Roxana Petcu, Fanny Emily Petermann-Rocha, Vivek Podder, Maarten J Postma, Ghazaleh Pourali, Elton Junio Sady Prates, Mehran Rahimi, Vafa Rahimi-Movaghar, Md Mosfequr Rahman, Mohammad Hifz Ur Rahman, Shayan Rahmani, Niloufar Rahnavard, Bibek Rajbhandari, Pradhum Ram, Sheena Ramazanu, Nemanja Rancic, Muhammad Modassar Ali Nawaz Ranjha, Chythra R Rao, Deepthi Rapaka, Ahmed Mustafa Rashid, Zubair Ahmed Ratan, Salman Rawaf, Lal Rawal, Elrashdy Moustafa Mohamed Redwan, Giuseppe Remuzzi, Luis Felipe Reyes, Nazila Rezaei, Nima Rezaei, Hossein Rezazadeh, Seyed Mohammad Riahi, Yohanes Andy Rias, Muhammad Riaz, Daniela Ribeiro, Mónica Rodrigues, Jefferson Antonio Buendia Rodriguez, Leonardo Roever, Peter Rohloff, Gholamreza Roshandel, Abazar Roustazadeh, Godfrey M Rwegerera, Aly M A Saad, Maha Mohamed Saber-Ayad, Siamak Sabour, Leila Sabzmakan, Basema Saddik, Umar Saeed, Sahar Saeedi Moghaddam, Sher Zaman Safi, Fatemeh Saheb Sharif-Askari, Narjes Saheb Sharif-Askari, Amirhossein Sahebkar, Harihar Sahoo, Soumya Swaroop Sahoo, KM Saif-Ur-Rahman, Mirza Rizwan Sajid, Saina Salahi, Sarvenaz Salahi, Mohammad Amin Salehi, Juan Sanabria, Francesco Sanmarchi, Milena M Santric-Milicevic, Thirunavukkarasu Sathish, Maria Inês Schmidt, Art Schuermans, Nachimuthu Senthil Kumar, Sadaf G Sepanlou, Yashendra Sethi, Allen Seylani, Maryam Shabany, Tahereh Shafaghat, Melika Shafeghat, Mahan Shafie, Nilay S Shah, Samiah Shahid, Mohd Shanawaz, Mohammed Shannawaz, Bereket Beyene Shashamo, Aminu Shittu, K M Shivakumar, Siddharudha Shivalli, Parnian Shobeiri, Fereshteh Shokri, Kerem Shuval, Migbar Mekonnen Sibhat, Colin R Simpson, Jasvinder A Singh, Paramdeep Singh, Anna Aleksandrovna Skryabina, Amanda E Smith, Abdullah Al Mamun Sohag, Hamidreza Soleimani, Solikhah Solikhah, Ranjani Somayaji, Lauryn K Stafford, Antonina V Starodubova, Muhammad Suleman, Johan Sundström, Seyyed Mohammad Tabatabaei, Seyed-Amir Tabatabaeizadeh, Mohammad Tabish, Ensiyeh Taheri, Majid Taheri, Jacques JL Lukenze Tamuzi, Ker-Kan Tan, Nathan Y Tat, Worku Animaw Temesgen, Mohamad-Hani Temsah, Riki Tesler, Pugazhenthan Thangaraju, Kavumpurathu Raman Thankappan, Samar Tharwat, Jansje Henny Vera Ticoalu, Marcello Tonelli, Marcos Roberto Tovani-Palone, Domenico Trico, Jaya Prasad Tripathy, Samuel Joseph Tromans, Guesh Mebrahtom Tsegay, Stefanos Tyrovolas, Era Upadhyay, Asokan Govindaraj Vaithinathan, Kim Robin van Daalen, Priya Vart, Shoban Babu Varthya, Tommi Juhani Vasankari, Madhur verma Verma, Georgios-Ioannis Verras, Danh Cao Vo, Stein Emil Vollset, Theo Vos, Birhanu Wagaye, Yasir Waheed, Fang Wang, Ziyue Wang, Gizachew Tadesse Wassie, Melissa Y Wei Wei, Ronny Westerman, Nuwan Darshana Wickramasinghe, Juan Xia, Hong Xiao, Lin Yang, Hunachew Kibret Yohannis, Naohiro Yonemoto, Yuyi You, Sojib Bin Zaman, Iman Zare, Kourosh Zarea, Armin Zarrintan, Mikhail Sergeevich Zastrozhin, Chenwen Zhong, Magdalena Zielińska, Yossef Teshome Zikarg, Mohammad Zoladl, Zhiyong Zou, and Alimuddin Zumla.

Managing the estimation or publications process

Denberu Eshetie Adane, Ali Ahmadi, Mulu Tiruneh Asemu, Dinh-Toi Chu, Ali Fatehizadeh, Hailey Hagins, Simon I Hay, Hong-Han Huynh, Haitham Jahrami, Samad Karkhah, Fatemeh khorashadizadeh, Thao Thi Thu Le, Samson Mideksa Legesse, Soleiman Mahjoub, Borhan Mansouri, Reza Masoudi, Susan A McLaughlin, Le Huu Nhat Minh, Mohsen Mohammadi, Ali H Mokdad, Christopher J L Murray, Mohsen Naghavi, Dang H Nguyen, Hien Quang Nguyen, Phat Tuan Nguyen, Van Thanh Nguyen, Kanyin

Liane Ong, Maja Pasovic, Bibek Rajbhandari, Nemanja Rancic, Hossein Rezazadeh, Yohanes Andy Rias, Aly M A Saad, Amanda E Smith, Shoban Babu Varthya, Danh Cao Vo, Theo Vos, and Mikhail Sergeevich Zastrozhin.
